# Supplementary material for: Activation of glucocorticoid receptor signaling inhibits KSHV-induced inflammation and tumorigenesis
Source: mBio. 2023 Dec 20;15(1):e03011-23. doi: 10.1128/mbio.03011-23 (PMC10790708; doi:10.1128/mbio.03011-23)
Supplement: Supplemental Figures and Tables — Figures S1 and S2, and Tables S1-S6. [file mbio.03011-23-s0001.pdf]

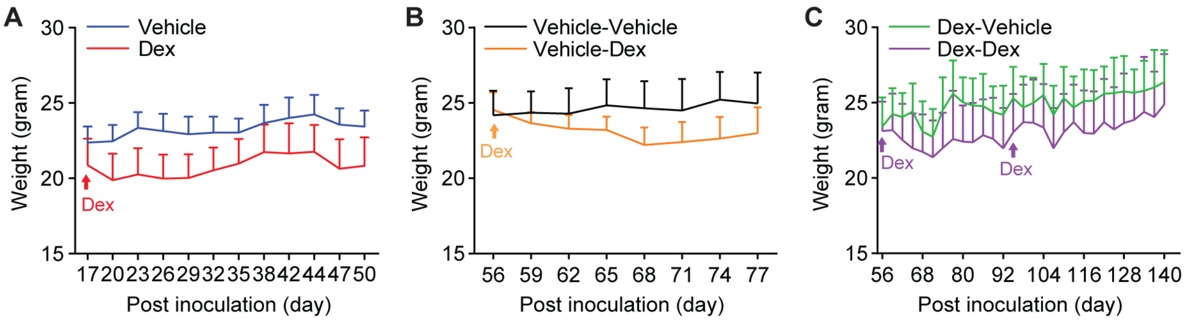

FIG S1 Body weights of mice treated with vehicle and dexamethasone (Dex) described in Fig 2. (A) Body weights of mice in Stage I experiment. (B) Body weights of mice in Stage II tumor regression experiment. (C) Body weights of mice in Stage II maintenance experiment.

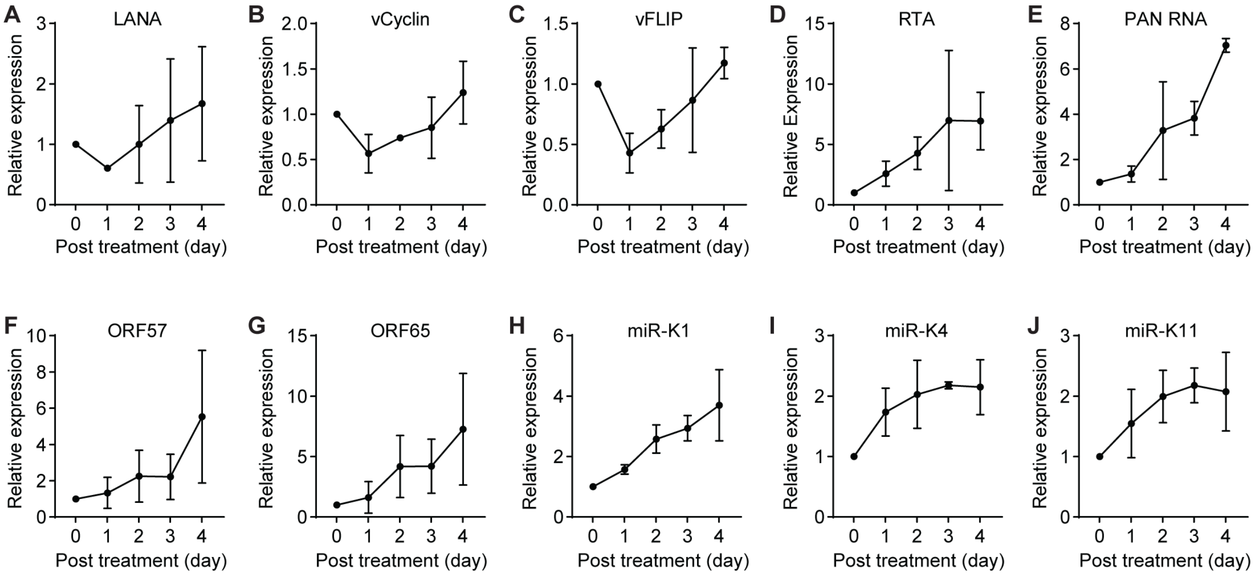

FIG S2 Expression levels of KSHV genes treated with dexamethasone. (A) LANA. (B) vCyclin. (C) vFLIP. (D) RTA. (E) PAN RNA. (F) ORF57. (G) ORF65. (H) miR-K1. (I) miR-K4. (J) miR-K11.

Table S1. Differentially expressed genes in KMM cells compared to MM cells

Cutoffs for differentially expressed genes: Fold change>2.5, P-value<0.05, FDR<0.05

Name	Log_2 fold change	Fold change	P-value	FDR p-value	Bonferroni	ENSEMBL	Biotype
Vom2r6	3.24542	9.48353	0.01803	0.03786	1	ENSRNO	protein_coding
Zc3h12d	1.64908	3.13633	0.0118	0.02607	1	ENSRNO	protein_coding
Ust	-1.8149	-3.5182	1.4E-07	7E-07	0.00272	ENSRNO	protein_coding
Samd5	1.77648	3.4259	7.2E-18	7.8E-17	1.4E-13	ENSRNO	protein_coding
Adgb	3.4496	10.9253	7.5E-08	3.8E-07	0.00142	ENSRNO	protein_coding
Grm1	1.42084	2.67741	0.00013	0.00043	1	ENSRNO	protein_coding
Plagl1	-6.7993	-111.37	3E-131	3E-129	5E-127	ENSRNO	protein_coding
Phactr2	-7.208	-147.85	2.3E-41	5.9E-40	4.4E-37	ENSRNO	protein_coding
Fuca2	-6.6821	-102.69	2.9E-79	1.5E-77	5.5E-75	ENSRNO	protein_coding
Aig1	-6.236	-75.372	2E-235	5E-233	3E-231	ENSRNO	protein_coding
Adgrg6	-8.1604	-286.11	3E-191	6E-189	6E-187	ENSRNO	protein_coding
Tnfaip3	2.00523	4.01453	4.4E-33	9E-32	8.3E-29	ENSRNO	protein_coding
Pde7b	5.47377	44.4396	0.02294	0.04683	1	ENSRNO	protein_coding
Myb	2.08537	4.24385	6.1E-10	3.9E-09	1.2E-05	ENSRNO	protein_coding
Aldh8a1	1.93913	3.83474	0.0074	0.01723	1	ENSRNO	protein_coding
Themis	8.33547	323.018	3.2E-11	2.3E-10	6.1E-07	ENSRNO	protein_coding
Enpp1	-1.3289	-2.5122	4.2E-39	1E-37	8E-35	ENSRNO	protein_coding
Ctgf	-2.1366	-4.3972	2E-100	1.4E-98	3.3E-96	ENSRNO	protein_coding
Moxd1	5.47383	44.4412	0.02266	0.04637	1	ENSRNO	protein_coding
Taar7b	7.28916	156.407	5.2E-14	4.5E-13	1E-09	ENSRNO	protein_coding
Vnn3	6.79414	110.978	0.00426	0.0105	1	ENSRNO	protein_coding
AABR070	5.19198	36.5547	9.7E-12	7.1E-11	1.8E-07	ENSRNO	protein_coding
LOC5014	3.68268	12.841	1.4E-05	5.3E-05	0.26427	ENSRNO	protein_coding
RGD1562	4.47024	22.1655	5.6E-27	9.3E-26	1.1E-22	ENSRNO	protein_coding
Trdn	-1.4543	-2.7402	2.2E-07	1.1E-06	0.00421	ENSRNO	protein_coding
Ahrr	-4.6544	-25.183	2E-270	1E-267	5E-266	ENSRNO	protein_coding
Tppp	-6.2949	-78.516	0.01093	0.02433	1	ENSRNO	protein_coding
Nkd2	-3.2322	-9.3971	1.4E-42	3.7E-41	2.6E-38	ENSRNO	protein_coding
Slc12a7	-1.3741	-2.592	4.4E-30	8.1E-29	8.3E-26	ENSRNO	protein_coding
Irx4	4.13501	17.5696	4.2E-08	2.2E-07	0.00079	ENSRNO	protein_coding
Irx2	2.38483	5.22283	8.1E-14	6.9E-13	1.5E-09	ENSRNO	protein_coding
LOC5014	4.25519	19.0959	4.7E-05	0.00017	0.89998	ENSRNO	protein_coding
AABR070	2.58747	6.01045	0.00052	0.00154	1	ENSRNO	lincRNA
Plekhg1	4.07128	16.8103	3.1E-77	1.5E-75	5.8E-73	ENSRNO	protein_coding
Akap12	2.94279	7.68897	2.8E-81	1.5E-79	5.2E-77	ENSRNO	protein_coding
Myct1	-7.759	-216.62	1.6E-20	2E-19	3E-16	ENSRNO	protein_coding
Ipcef1	5.83574	57.1126	3.4E-17	3.6E-16	6.5E-13	ENSRNO	protein_coding
Zdhhc14	2.77806	6.85929	6.7E-25	1E-23	1.3E-20	ENSRNO	protein_coding
Tagap	3.13326	8.77413	0.02275	0.04652	1	ENSRNO	protein_coding
Fndc1	-3.2143	-9.2809	1E-106	9E-105	2E-102	ENSRNO	protein_coding
Igf2r	1.43988	2.71297	1.5E-37	3.4E-36	2.8E-33	ENSRNO	protein_coding
Slc22a3	6.18161	72.5854	0.0098	0.02209	1	ENSRNO	protein_coding
AABR070	1.79886	3.47944	0.00106	0.00295	1	ENSRNO	lincRNA
Rps6ka2	7.17224	144.231	2E-136	2E-134	3E-132	ENSRNO	protein_coding

AABR070	-1.6836	-3.2122	0.01788	0.0376	1	ENSRNO	lincRNA
Dact2	-3.5678	-11.858	6.5E-08	3.3E-07	0.00123	ENSRNO	protein_coding
Thbs2	-3.6639	-12.675	4E-277	2E-274	9E-273	ENSRNO	protein_coding
Dll1	3.18827	9.11518	1.8E-43	5E-42	3.5E-39	ENSRNO	protein_coding
Lix1	3.95285	15.4855	9.9E-06	3.9E-05	0.18818	ENSRNO	protein_coding
AABR070	-3.3827	-10.43	2.6E-08	1.4E-07	0.0005	ENSRNO	protein_coding
Vom2r16	2.33311	5.03891	0.01985	0.04122	1	ENSRNO	protein_coding
Zik1	8.51928	366.91	0.00053	0.00155	1	ENSRNO	protein_coding
Lilrb3l	2.47344	5.55368	0.00045	0.00135	1	ENSRNO	protein_coding
Mboat7	-8.2183	-297.82	0.00175	0.00466	1	ENSRNO	protein_coding
LOC2925	1.79676	3.4744	0.00258	0.00666	1	ENSRNO	protein_coding
LOC1025	5.64018	49.8726	0.02124	0.04375	1	ENSRNO	protein_coding
Zfp580	1.58013	2.98997	0.00027	0.00085	1	ENSRNO	protein_coding
Zfp579	3.52055	11.476	7.1E-10	4.4E-09	1.3E-05	ENSRNO	protein_coding
Ssc5d	-2.2721	-4.8302	4.2E-05	0.00015	0.80633	ENSRNO	protein_coding
Shisa7	-2.508	-5.6883	0.00352	0.00883	1	ENSRNO	protein_coding
AABR070	-1.4931	-2.8149	0.00232	0.00603	1	ENSRNO	pseudogene
Tmem190	-1.3587	-2.5645	0.00013	0.00043	1	ENSRNO	protein_coding
Il11	-2.107	-4.3078	0.0025	0.00646	1	ENSRNO	protein_coding
Ptprh	-2.6801	-6.4088	0.00537	0.01294	1	ENSRNO	protein_coding
Dnaaf3	2.17411	4.51308	3.3E-31	6.3E-30	6.2E-27	ENSRNO	protein_coding
Tnni3	4.65744	25.2365	5.6E-21	7.1E-20	1.1E-16	ENSRNO	protein_coding
Eps8l1	-3.7474	-13.43	4E-05	0.00014	0.76822	ENSRNO	protein_coding
Lilrb4	4.35426	20.4533	3.5E-33	7.2E-32	6.6E-29	ENSRNO	protein_coding
Lilrb3	4.42039	21.4126	0.00111	0.00307	1	ENSRNO	protein_coding
Lilra5	6.11589	69.3533	0.01119	0.02483	1	ENSRNO	protein_coding
Slc1a5	1.38737	2.61602	5.6E-40	1.4E-38	1.1E-35	ENSRNO	protein_coding
Psgb1	4.64885	25.0867	0.00051	0.00151	1	ENSRNO	protein_coding
Mill1	3.02834	8.15869	1.1E-05	4.4E-05	0.21675	ENSRNO	protein_coding
Eml2	-2.3155	-4.9777	4.4E-92	2.9E-90	8.5E-88	ENSRNO	protein_coding
Klc3	2.84385	7.17934	0.00032	0.00098	1	ENSRNO	protein_coding
Ckm	3.92207	15.1586	0.00391	0.0097	1	ENSRNO	protein_coding
Exoc3l2	1.59068	3.01192	1.4E-07	7E-07	0.00271	ENSRNO	protein_coding
Zfp296	-1.4569	-2.7452	0.00011	0.00036	1	ENSRNO	protein_coding
Relb	1.44523	2.72306	7.6E-12	5.6E-11	1.4E-07	ENSRNO	protein_coding
Nectin2	-1.5231	-2.8742	7.6E-11	5.2E-10	1.4E-06	ENSRNO	protein_coding
Zfp112	-3.6631	-12.668	6.7E-13	5.3E-12	1.3E-08	ENSRNO	protein_coding
Kcnn4	2.22471	4.67418	2E-105	1E-103	3E-101	ENSRNO	protein_coding
Cadm4	-5.3232	-40.034	2.2E-25	3.4E-24	4.2E-21	ENSRNO	protein_coding
Lypd3	6.72607	105.864	0.00476	0.01161	1	ENSRNO	protein_coding
LOC1009	11.1075	2206.49	2.6E-06	1.1E-05	0.04857	ENSRNO	protein_coding
LOC1009	7.12017	139.118	0.00265	0.00682	1	ENSRNO	protein_coding
Cd79a	3.6197	12.2924	8.1E-05	0.00028	1	ENSRNO	protein_coding
Ceacam1	1.35073	2.55041	4.4E-19	5E-18	8.3E-15	ENSRNO	protein_coding
Tgfb1	1.69585	3.23968	2.9E-23	4.1E-22	5.5E-19	ENSRNO	protein_coding
Cyp2s1	5.02347	32.5248	6.8E-25	1E-23	1.3E-20	ENSRNO	protein_coding
LOC1003	3.10744	8.61851	7.9E-40	2E-38	1.5E-35	ENSRNO	protein_coding
LOC1009	6.69068	103.299	0.00494	0.01202	1	ENSRNO	protein_coding
LOC1025	5.94598	61.648	1E-178	2E-176	2E-174	ENSRNO	protein_coding

LOC1009	6.69055	103.29	0.005	0.01214	1	ENSRNO	protein_coding
RGD1565	6.46535	88.362	5.8E-07	2.7E-06	0.011	ENSRNO	protein_coding
Cd177	6.2985	78.7112	0.00872	0.01991	1	ENSRNO	protein_coding
Mia	2.72637	6.61787	0.00094	0.00265	1	ENSRNO	protein_coding
Sptbn4	2.94964	7.72558	0.00013	0.00042	1	ENSRNO	protein_coding
AABR070	-2.6374	-6.2219	0.00021	0.00067	1	ENSRNO	protein_coding
ENSRNO	-1.6862	-3.2181	0.00808	0.0186	1	ENSRNO	protein_coding
Fcgbp	1.85758	3.62399	9.5E-06	3.7E-05	0.18091	ENSRNO	protein_coding
Plekhg2	-2.1616	-4.4741	3.6E-51	1.1E-49	6.8E-47	ENSRNO	protein_coding
Eid2	1.32669	2.50827	0.02016	0.04173	1	ENSRNO	protein_coding
Rinl	4.43189	21.584	2.3E-09	1.4E-08	4.4E-05	ENSRNO	protein_coding
Lgals4	6.09555	68.382	2.8E-06	1.2E-05	0.05384	ENSRNO	protein_coding
Spint2	6.9282	121.785	3E-137	3E-135	5E-133	ENSRNO	protein_coding
Ryr1	2.81667	7.04535	9.5E-22	1.2E-20	1.8E-17	ENSRNO	protein_coding
Ggn	1.48251	2.79434	0.00044	0.00132	1	ENSRNO	protein_coding
Yif1b	7.76673	217.781	0.00113	0.00312	1	ENSRNO	protein_coding
AABR070	1.81277	3.51316	0.01914	0.03991	1	ENSRNO	lincRNA
AABR070	-3.7192	-13.171	8.5E-07	3.8E-06	0.0161	ENSRNO	protein_coding
Lrfn3	3.56944	11.8716	3.7E-08	1.9E-07	0.0007	ENSRNO	protein_coding
Nfkbid	2.3337	5.04095	2.8E-12	2.1E-11	5.3E-08	ENSRNO	protein_coding
Kirrel2	6.23219	75.1756	0.00971	0.0219	1	ENSRNO	protein_coding
Nphs1	4.43259	21.5944	2.9E-16	2.9E-15	5.5E-12	ENSRNO	protein_coding
Prodh2	4.28732	19.526	5.5E-07	2.5E-06	0.01048	ENSRNO	protein_coding
Lgi4	-6.913	-120.51	0.00457	0.0112	1	ENSRNO	protein_coding
Scn1b	-2.4218	-5.3585	3.6E-15	3.3E-14	6.8E-11	ENSRNO	protein_coding
RGD1308	-1.4839	-2.797	1.9E-46	5.5E-45	3.6E-42	ENSRNO	protein_coding
Kctd15	4.54409	23.3297	1.8E-88	1.1E-86	3.5E-84	ENSRNO	protein_coding
LOC1083	-3.5352	-11.593	6.1E-06	2.5E-05	0.11642	ENSRNO	protein_coding
Clip3	-6.4723	-88.787	2.5E-35	5.4E-34	4.7E-31	ENSRNO	protein_coding
NEWGEN	6.87426	117.316	0.00741	0.01726	1	ENSRNO	protein_coding
Rhpn2	1.34244	2.5358	1.7E-28	2.9E-27	3.2E-24	ENSRNO	protein_coding
Tshz3	-6.7701	-109.15	2.1E-64	9.1E-63	4.1E-60	ENSRNO	protein_coding
RGD1562	4.43482	21.6278	9.6E-06	3.8E-05	0.1834	ENSRNO	protein_coding
Rn50_1_(	4.56927	23.7403	1.5E-24	2.2E-23	2.8E-20	ENSRNO	unprocessed_pseudogene
AABR070	-1.3261	-2.5072	0.00767	0.01779	1	ENSRNO	lincRNA
Siglec10	-2.4525	-5.4735	3.2E-27	5.4E-26	6.2E-23	ENSRNO	protein_coding
Klk14	2.72424	6.60811	8.4E-06	3.3E-05	0.16024	ENSRNO	protein_coding
Klk6	-2.1094	-4.3152	0.00103	0.00287	1	ENSRNO	protein_coding
Spib	3.62322	12.3225	0.00762	0.01769	1	ENSRNO	protein_coding
Napsa	1.4643	2.75929	0.0001	0.00034	1	ENSRNO	protein_coding
Myh14	2.09737	4.2793	0.00118	0.00325	1	ENSRNO	protein_coding
Zfp473	-2.0812	-4.2317	7.1E-08	3.6E-07	0.00135	ENSRNO	protein_coding
Rcn3	-5.6807	-51.294	5E-180	9E-178	9E-176	ENSRNO	protein_coding
Slc17a7	-5.5034	-45.362	6.7E-08	3.4E-07	0.00127	ENSRNO	protein_coding
Ccdc155	1.83575	3.56957	0.00739	0.01722	1	ENSRNO	protein_coding
Ppfia3	-1.3859	-2.6134	0.00173	0.00462	1	ENSRNO	protein_coding
Lhb	1.38858	2.61821	0.00654	0.01543	1	ENSRNO	protein_coding
Car11	-4.0885	-17.012	8.8E-22	1.2E-20	1.7E-17	ENSRNO	protein_coding
Grin2d	-3.0711	-8.4044	3.4E-13	2.7E-12	6.4E-09	ENSRNO	protein_coding

AC12080	5.37207	41.4147	3E-86	1.8E-84	5.8E-82	ENSRNO	lincRNA
Otog	5.9429	61.5165	2.2E-09	1.3E-08	4.1E-05	ENSRNO	protein_coding
Tph1	2.52116	5.74042	6.8E-12	5E-11	1.3E-07	ENSRNO	protein_coding
Slc6a5	2.35084	5.10122	0.00141	0.00382	1	ENSRNO	protein_coding
Fancf	1.99411	3.9837	0.00349	0.00876	1	ENSRNO	protein_coding
Gas2	6.65414	100.715	0.00528	0.01276	1	ENSRNO	protein_coding
LOC1083	3.32716	10.0363	0.00209	0.00548	1	ENSRNO	lincRNA
AABR070	2.0502	4.14164	0.00344	0.00866	1	ENSRNO	lincRNA
Apba2	1.7583	3.38299	6.4E-09	3.6E-08	0.00012	ENSRNO	protein_coding
Pcsk6	-7.6982	-207.67	8E-101	6E-99	1.5E-96	ENSRNO	protein_coding
Aldh1a3	1.90942	3.75658	0.00198	0.00522	1	ENSRNO	protein_coding
Adamts17	2.2616	4.79523	0.00817	0.0188	1	ENSRNO	protein_coding
Arrdc4	-5.5475	-46.771	1.4E-15	1.4E-14	2.8E-11	ENSRNO	protein_coding
Nr2f2	-1.6277	-3.0902	3.2E-27	5.4E-26	6.2E-23	ENSRNO	protein_coding
Mctp2	3.07049	8.40061	0.00044	0.00132	1	ENSRNO	protein_coding
Rgma	-1.6772	-3.198	4.2E-23	5.9E-22	8E-19	ENSRNO	protein_coding
Acan	-3.8104	-14.03	5E-297	3E-294	9E-293	ENSRNO	protein_coding
Mfge8	-2.0749	-4.2133	1.2E-95	8.6E-94	2.3E-91	ENSRNO	protein_coding
Mesp2	2.45603	5.48703	6.3E-06	2.5E-05	0.11893	ENSRNO	protein_coding
Fes	1.96201	3.89603	0.01645	0.03492	1	ENSRNO	protein_coding
Alpk3	4.12842	17.4896	4.8E-12	3.6E-11	9.1E-08	ENSRNO	protein_coding
Slc28a1	2.84712	7.1956	4.1E-12	3.1E-11	7.8E-08	ENSRNO	protein_coding
Cpeb1	-7.6423	-199.79	1.2E-58	4.7E-57	2.4E-54	ENSRNO	protein_coding
Ap3b2	-2.1008	-4.2894	9.2E-23	1.3E-21	1.8E-18	ENSRNO	protein_coding
Homer2	-6.3084	-79.256	4.8E-79	2.5E-77	9.1E-75	ENSRNO	protein_coding
Cemip	-4.0603	-16.683	7.7E-32	1.5E-30	1.5E-27	ENSRNO	protein_coding
Arnt2	-2.1721	-4.5067	8.9E-96	6.3E-94	1.7E-91	ENSRNO	protein_coding
LOC1003	5.33429	40.3443	5E-137	6E-135	1E-132	ENSRNO	protein_coding
LOC1009	5.1645	35.8649	1.3E-42	3.6E-41	2.6E-38	ENSRNO	protein_coding
Xlr3a	4.36357	20.5856	2.3E-41	5.9E-40	4.3E-37	ENSRNO	protein_coding
LOC1003	-5.8246	-56.673	0.01805	0.0379	1	ENSRNO	protein_coding
Nox4	-6.3211	-79.956	7.6E-23	1.1E-21	1.4E-18	ENSRNO	protein_coding
Prss23	-4.1761	-18.077	1.7E-83	9.5E-82	3.2E-79	ENSRNO	protein_coding
Me3	3.17036	9.00274	2.6E-05	9.6E-05	0.49935	ENSRNO	protein_coding
Ccdc81	-6.8788	-117.69	2.3E-12	1.8E-11	4.4E-08	ENSRNO	protein_coding
Sytl2_2	6.62822	98.9221	0.00962	0.02171	1	ENSRNO	protein_coding
Tenm4	3.04097	8.23043	0.00099	0.00277	1	ENSRNO	protein_coding
Aamdc	1.61483	3.06276	0.00025	0.00078	1	ENSRNO	protein_coding
Pak1	-2.8725	-7.3231	8E-148	1E-145	1E-143	ENSRNO	protein_coding
Myo7a	-2.7565	-6.7576	1.6E-05	6E-05	0.29946	ENSRNO	protein_coding
Tsku	2.42567	5.37278	6E-115	6E-113	1E-110	ENSRNO	protein_coding
Mogat2	-2.5263	-5.761	0.02373	0.04824	1	ENSRNO	protein_coding
Gdpd5	-1.7277	-3.3119	1.3E-20	1.6E-19	2.5E-16	ENSRNO	protein_coding
Arrb1	-1.6343	-3.1044	2E-32	4E-31	3.8E-28	ENSRNO	protein_coding
P4ha3	-2.1022	-4.2935	1.8E-91	1.2E-89	3.5E-87	ENSRNO	protein_coding
Trim21	2.34693	5.08741	6.1E-41	1.6E-39	1.2E-36	ENSRNO	protein_coding
Hpx	2.44052	5.42837	9.4E-09	5.2E-08	0.00018	ENSRNO	protein_coding
Cyb5r2	3.17634	9.04013	0.00017	0.00056	1	ENSRNO	protein_coding
LOC4992	2.20576	4.61316	4E-08	2.1E-07	0.00076	ENSRNO	protein_coding

Trim66	1.68366	3.21243	0.0046	0.01126	1	ENSRNO	protein_coding
Nrip3	-3.8902	-14.828	3.9E-49	1.2E-47	7.5E-45	ENSRNO	protein_coding
Scube2	-2.0777	-4.2214	0.0093	0.02107	1	ENSRNO	protein_coding
AABR070	-1.5707	-2.9704	0.00071	0.00206	1	ENSRNO	pseudogene
Ampd3	5.10289	34.3654	3E-92	2E-90	5.8E-88	ENSRNO	protein_coding
Mrvi1	-3.5055	-11.357	0.0012	0.00329	1	ENSRNO	protein_coding
Micalcl	2.0731	4.2079	9.6E-05	0.00032	1	ENSRNO	protein_coding
Rras2	-1.4341	-2.7021	1.1E-44	3.1E-43	2.1E-40	ENSRNO	protein_coding
Cyp2r1	-7.7279	-211.99	1.1E-09	6.7E-09	2.1E-05	ENSRNO	protein_coding
Plekha7	-3.2431	-9.4685	7E-123	7E-121	1E-118	ENSRNO	protein_coding
Sox6	2.5826	5.9902	8.2E-10	5.1E-09	1.6E-05	ENSRNO	protein_coding
Acsm1	-4.3196	-19.967	4.3E-05	0.00015	0.82416	ENSRNO	protein_coding
AABR070	-1.7733	-3.4184	0.00105	0.00293	1	ENSRNO	lincRNA
Zp2	5.63112	49.5605	0.01883	0.03936	1	ENSRNO	protein_coding
Anks4b	1.74034	3.34113	0.00115	0.00319	1	ENSRNO	protein_coding
Abca16	-2.5018	-5.6639	0.00142	0.00386	1	ENSRNO	protein_coding
Aqp8	5.00845	32.188	0.00013	0.00044	1	ENSRNO	protein_coding
Snrpn	-2.2385	-4.7189	0.00047	0.00139	1	ENSRNO	protein_coding
Snurf	-1.9937	-3.9825	4.9E-07	2.3E-06	0.0093	ENSRNO	protein_coding
Il4r	1.91102	3.76074	2.3E-32	4.6E-31	4.4E-28	ENSRNO	protein_coding
Cln3	-2.3429	-5.0733	3E-14	2.7E-13	5.8E-10	ENSRNO	protein_coding
Apobr	5.83605	57.1251	0.01523	0.03268	1	ENSRNO	protein_coding
Sult1a1	-1.6926	-3.2324	0.00145	0.00394	1	ENSRNO	protein_coding
Fam57b	3.44085	10.8593	0.0013	0.00354	1	ENSRNO	protein_coding
Kctd13	1.97188	3.9228	8.6E-29	1.5E-27	1.6E-24	ENSRNO	protein_coding
Prrt2	5.96252	62.3589	0.01269	0.02782	1	ENSRNO	protein_coding
Mylpf	1.74928	3.36192	0.00724	0.0169	1	ENSRNO	protein_coding
Sept1	1.59168	3.014	0.00021	0.00068	1	ENSRNO	protein_coding
Itgal	1.78566	3.44775	0.00533	0.01286	1	ENSRNO	protein_coding
Stx1b	1.75381	3.37249	0.02115	0.04359	1	ENSRNO	protein_coding
Pycard	1.34403	2.5386	5.7E-06	2.3E-05	0.10793	ENSRNO	protein_coding
Itgad	2.88755	7.40011	0.00371	0.00925	1	ENSRNO	protein_coding
Itgax	4.22778	18.7365	3E-238	9E-236	6E-234	ENSRNO	protein_coding
Cox6a2	2.69468	6.47412	2.1E-14	1.9E-13	4E-10	ENSRNO	protein_coding
Slc5a2	2.79967	6.96281	6E-05	0.00021	1	ENSRNO	protein_coding
Ahsp	3.14566	8.84987	5E-19	5.8E-18	9.6E-15	ENSRNO	protein_coding
Fgfr2	-7.1421	-141.25	1.4E-86	8.4E-85	2.7E-82	ENSRNO	protein_coding
RGD1560	2.55543	5.87842	0.01113	0.02471	1	ENSRNO	protein_coding
Cpxm2	-1.4166	-2.6696	0.01222	0.02689	1	ENSRNO	protein_coding
Chst15	-1.9551	-3.8775	1E-31	2E-30	1.9E-27	ENSRNO	protein_coding
Ptpre	1.64897	3.1361	7.2E-20	8.5E-19	1.4E-15	ENSRNO	protein_coding
Stk32c	-7.2924	-156.76	0.0026	0.0067	1	ENSRNO	protein_coding
RGD1309	6.49539	90.2211	4E-71	1.9E-69	7.7E-67	ENSRNO	protein_coding
Ifitm1	-3.5311	-11.56	2.7E-25	4.1E-24	5.1E-21	ENSRNO	protein_coding
B4galnt4	-4.8488	-28.815	6.3E-15	5.7E-14	1.2E-10	ENSRNO	protein_coding
Eps8l2	2.88735	7.39908	1E-171	2E-169	2E-167	ENSRNO	protein_coding
Polr2l	2.7354	6.65945	0.00017	0.00055	1	ENSRNO	protein_coding
ENSRNO	4.59152	24.1094	0.00089	0.00251	1	ENSRNO	protein_coding
Lsp1	-2.6659	-6.3463	1.6E-13	1.3E-12	3E-09	ENSRNO	protein_coding

Th	4.82817	28.4069	1.9E-13	1.5E-12	3.5E-09	ENSRNO	protein_coding
Slc22a18	3.5752	11.919	2.2E-24	3.2E-23	4.1E-20	ENSRNO	protein_coding
Lrp5	1.38322	2.60849	2.1E-40	5.2E-39	4E-36	ENSRNO	protein_coding
RGD1311	-1.8072	-3.4997	8.2E-61	3.2E-59	1.6E-56	ENSRNO	protein_coding
Cabp4	1.37532	2.59425	0.02211	0.04534	1	ENSRNO	protein_coding
Pold4	-1.5235	-2.8749	3.2E-08	1.7E-07	0.00061	ENSRNO	protein_coding
LOC6890	4.35727	20.496	7.3E-09	4.1E-08	0.00014	ENSRNO	protein_coding
Ctsf	-3.3844	-10.443	1.3E-80	7.2E-79	2.6E-76	ENSRNO	protein_coding
Peli3	-1.7868	-3.4505	5.5E-09	3.2E-08	0.00011	ENSRNO	protein_coding
Cst6	4.19183	18.2754	9.2E-06	3.6E-05	0.17409	ENSRNO	protein_coding
Ctsw	-5.3256	-40.101	1.1E-20	1.4E-19	2.1E-16	ENSRNO	protein_coding
Ap5b1	1.68479	3.21493	1.3E-34	2.8E-33	2.5E-30	ENSRNO	protein_coding
Slc25a45	3.71841	13.1629	4.2E-05	0.00015	0.80435	ENSRNO	protein_coding
Tigd3	1.52254	2.87296	4.1E-05	0.00015	0.78383	ENSRNO	protein_coding
Majin	7.09369	136.588	0.0028	0.00716	1	ENSRNO	protein_coding
Cdc42bpg	2.31891	4.98955	1.6E-05	6.1E-05	0.30862	ENSRNO	protein_coding
Map4k2	-1.5845	-2.999	3.4E-13	2.8E-12	6.5E-09	ENSRNO	protein_coding
Rasgrp2	3.8185	14.1086	7.3E-07	3.3E-06	0.0138	ENSRNO	protein_coding
Nrxn2	-3.8876	-14.801	0.00027	0.00085	1	ENSRNO	protein_coding
Esrra	1.3935	2.62716	1.5E-32	3E-31	2.8E-28	ENSRNO	protein_coding
Tex40	3.28299	9.73371	0.00016	0.00053	1	ENSRNO	protein_coding
Kcnk4	2.93533	7.64934	0.00179	0.00475	1	ENSRNO	protein_coding
Nudt22	-2.6468	-6.2628	1.3E-08	7.3E-08	0.00025	ENSRNO	protein_coding
Trpt1	-4.6302	-24.765	1.4E-09	8.5E-09	2.6E-05	ENSRNO	protein_coding
Fermt3	3.38563	10.4514	0.00073	0.00211	1	ENSRNO	protein_coding
Macrod1	-5.9922	-63.656	5.1E-06	2.1E-05	0.09743	ENSRNO	protein_coding
Flrt1	-3.5196	-11.468	0.01044	0.02336	1	ENSRNO	protein_coding
Rcor2	1.50946	2.84703	8.9E-05	0.0003	1	ENSRNO	protein_coding
RGD1560	1.48966	2.80824	8.8E-38	2E-36	1.7E-33	ENSRNO	protein_coding
UST4r	5.76309	54.308	0.01711	0.03617	1	ENSRNO	protein_coding
Ust5r	6.98727	126.876	3.5E-30	6.4E-29	6.6E-26	ENSRNO	protein_coding
Lrrn4cl	1.5739	2.97708	3.1E-06	1.3E-05	0.05964	ENSRNO	protein_coding
Dagla	4.93961	30.6882	2E-138	3E-136	4E-134	ENSRNO	protein_coding
Syt7	6.43938	86.7851	0.0075	0.01744	1	ENSRNO	protein_coding
Ms4a18	3.51903	11.4639	0.01402	0.03043	1	ENSRNO	protein_coding
Ms4a6a	4.36343	20.5837	1.4E-26	2.3E-25	2.7E-22	ENSRNO	protein_coding
Dtx4	-1.6259	-3.0864	2.1E-08	1.1E-07	0.00039	ENSRNO	protein_coding
LOC1009	6.80275	111.643	8.8E-08	4.4E-07	0.00167	ENSRNO	protein_coding
Tle4	3.9794	15.7732	4.3E-67	1.9E-65	8.2E-63	ENSRNO	protein_coding
Trpm6	-2.4005	-5.2797	2.6E-09	1.5E-08	4.9E-05	ENSRNO	protein_coding
Gna14	-2.8295	-7.1084	2E-05	7.4E-05	0.37606	ENSRNO	protein_coding
Prune2	-3.5923	-12.061	1.6E-06	6.8E-06	0.02972	ENSRNO	protein_coding
AABR070	-6.8807	-117.84	0.00469	0.01145	1	ENSRNO	protein_coding
Mamdc2	-4.947	-30.846	1.3E-37	3.1E-36	2.5E-33	ENSRNO	protein_coding
Tmem252	-1.6168	-3.0669	0.00241	0.00625	1	ENSRNO	protein_coding
Pgm5	-5.0734	-33.669	4.9E-65	2.1E-63	9.4E-61	ENSRNO	protein_coding
Dmrt2	-7.1439	-141.42	9.4E-18	1E-16	1.8E-13	ENSRNO	protein_coding
Vldlr	-2.5816	-5.986	4.9E-35	1.1E-33	9.3E-31	ENSRNO	protein_coding
Slc1a1	-4.3502	-20.396	3.7E-05	0.00013	0.71003	ENSRNO	protein_coding

Spata6l	1.53635	2.9006	0.00672	0.01583	1	ENSRNO	protein_coding
Mlana	5.84692	57.5569	1.3E-14	1.2E-13	2.5E-10	ENSRNO	protein_coding
Il33	2.15605	4.45692	0.01137	0.02521	1	ENSRNO	protein_coding
Uhrf2	-1.5255	-2.879	1.6E-41	4.1E-40	3E-37	ENSRNO	protein_coding
Asah2	-5.4822	-44.699	1.6E-17	1.7E-16	3.1E-13	ENSRNO	protein_coding
Papss2	-3.9421	-15.37	1.3E-58	5.1E-57	2.6E-54	ENSRNO	protein_coding
Acta2	-6.0197	-64.88	0	0	0	ENSRNO	protein_coding
Fas	-3.4644	-11.038	8.8E-13	7E-12	1.7E-08	ENSRNO	protein_coding
Ifit2	2.97697	7.8733	3E-18	3.3E-17	5.7E-14	ENSRNO	protein_coding
Ifit3	1.87478	3.66745	0.00029	0.0009	1	ENSRNO	protein_coding
Slc16a12	-5.8472	-57.567	9.8E-58	3.6E-56	1.9E-53	ENSRNO	protein_coding
Ankrd1	-6.281	-77.764	0	0	0	ENSRNO	protein_coding
Hhex	5.61378	48.9684	0.02105	0.04341	1	ENSRNO	protein_coding
Cyp26c1	-5.516	-45.76	2.9E-05	0.00011	0.55834	ENSRNO	protein_coding
Plce1	4.66873	25.4347	7.7E-55	2.7E-53	1.5E-50	ENSRNO	protein_coding
AABR070	-1.6355	-3.1069	0.00023	0.00072	1	ENSRNO	protein_coding
Aldh18a1	4.80402	27.9353	1.3E-11	9.7E-11	2.5E-07	ENSRNO	protein_coding
Entpd1	2.65028	6.2779	1.7E-08	9.5E-08	0.00033	ENSRNO	protein_coding
Zfp518a	-5.795	-55.522	4.4E-57	1.6E-55	8.4E-53	ENSRNO	protein_coding
Blnk	2.07633	4.21734	4.9E-08	2.5E-07	0.00093	ENSRNO	protein_coding
Golga7b	2.54914	5.85286	3.2E-09	1.9E-08	6E-05	ENSRNO	protein_coding
Cnnm1	6.47723	89.0923	7.3E-32	1.4E-30	1.4E-27	ENSRNO	protein_coding
Nkx2-3	-1.8037	-3.4913	4.5E-14	3.9E-13	8.6E-10	ENSRNO	protein_coding
Abcc2	-4.6543	-25.181	1.8E-12	1.4E-11	3.4E-08	ENSRNO	protein_coding
Scd	-2.6527	-6.2883	2E-120	2E-118	4E-116	ENSRNO	protein_coding
Lbx1	2.07292	4.20738	0.00841	0.01927	1	ENSRNO	protein_coding
LOC1025	1.87686	3.67276	0.0068	0.016	1	ENSRNO	lincRNA
Mfsd13a	2.0605	4.1713	6.3E-22	8.3E-21	1.2E-17	ENSRNO	protein_coding
As3mt	7.8211	226.144	0.00097	0.00272	1	ENSRNO	protein_coding
Neurl1	-4.6643	-25.356	4.5E-14	3.9E-13	8.6E-10	ENSRNO	protein_coding
Col17a1	1.95436	3.87545	0.00883	0.02013	1	ENSRNO	protein_coding
Sorcs1	5.28433	38.9711	1.5E-43	4.2E-42	2.9E-39	ENSRNO	protein_coding
LOC1009	1.64725	3.13236	2.3E-07	1.1E-06	0.00436	ENSRNO	lincRNA
RGD1561	1.48935	2.80762	0.01337	0.02915	1	ENSRNO	protein_coding
Add3	7.19645	146.672	5.9E-22	7.8E-21	1.1E-17	ENSRNO	protein_coding
Dusp5	1.49192	2.81262	3.4E-27	5.7E-26	6.5E-23	ENSRNO	protein_coding
Rbm20	1.93791	3.8315	6.6E-11	4.6E-10	1.3E-06	ENSRNO	protein_coding
Vwa2	3.06832	8.38798	0.00477	0.01163	1	ENSRNO	protein_coding
Afap1l2	-5.205	-36.886	3.1E-98	2.4E-96	6E-94	ENSRNO	protein_coding
Rn50_1_2	5.77302	54.6828	0.01635	0.03473	1	ENSRNO	lincRNA
AABR070	-8.9703	-501.55	0.00019	0.0006	1	ENSRNO	lincRNA
Gfra1	-8.6243	-394.63	4E-34	8.4E-33	7.6E-30	ENSRNO	protein_coding
Ccdc172	-1.5691	-2.9673	0.00104	0.0029	1	ENSRNO	protein_coding
AABR070	3.24548	9.48389	0.01993	0.04136	1	ENSRNO	lincRNA
Ces2c	-3.3358	-10.097	4.3E-16	4.2E-15	8.2E-12	ENSRNO	protein_coding
LOC1036	-1.4053	-2.6486	9.6E-05	0.00032	1	ENSRNO	lincRNA
AC11788	1.34701	2.54385	4.2E-11	3E-10	8.1E-07	ENSRNO	protein_coding
ENSRNO	1.96223	3.89664	0.02452	0.04967	1	ENSRNO	protein_coding
Rsl1d1	1.36168	2.56985	0.00039	0.00117	1	ENSRNO	protein_coding

Emp2	3.87834	14.706	3E-249	9E-247	5E-245	ENSRNO	protein_coding
Rpl39l	-4.7562	-27.025	2.1E-11	1.5E-10	4E-07	ENSRNO	protein_coding
Ppl	4.60875	24.3989	1E-300	9E-298	3E-296	ENSRNO	protein_coding
Adcy9	5.76005	54.1936	1.6E-51	5E-50	3E-47	ENSRNO	protein_coding
Hcfc1r1	1.82662	3.54705	9.9E-20	1.2E-18	1.9E-15	ENSRNO	protein_coding
RGD1561	1.96525	3.9048	2.9E-07	1.4E-06	0.00552	ENSRNO	protein_coding
NEWGEN	-7.188	-145.82	0.00318	0.00805	1	ENSRNO	protein_coding
Prss22	2.24749	4.74856	0.02347	0.04774	1	ENSRNO	protein_coding
Ntn3	1.42104	2.67779	0.00037	0.00111	1	ENSRNO	protein_coding
Eci1	1.56763	2.96417	1.2E-18	1.3E-17	2.3E-14	ENSRNO	protein_coding
Caskin1	-1.6638	-3.1685	3.8E-08	2E-07	0.00072	ENSRNO	protein_coding
Npw	-4.4673	-22.12	1.7E-05	6.3E-05	0.31661	ENSRNO	protein_coding
Rnf151	1.52986	2.88758	0.00027	0.00085	1	ENSRNO	protein_coding
Tmem204	-4.0149	-16.166	3.1E-14	2.7E-13	5.8E-10	ENSRNO	protein_coding
Cacna1h	2.67953	6.40648	0.02339	0.04761	1	ENSRNO	protein_coding
Mslnl	-1.9144	-3.7695	0.01999	0.04145	1	ENSRNO	protein_coding
Msln	-3.3331	-10.078	2E-196	4E-194	4E-192	ENSRNO	protein_coding
Fbxl16	-2.967	-7.8191	2.8E-07	1.4E-06	0.00538	ENSRNO	protein_coding
Jmjd8	1.50504	2.83833	1E-19	1.2E-18	1.9E-15	ENSRNO	protein_coding
Il9r	3.59551	12.088	0.01591	0.03392	1	ENSRNO	protein_coding
Nsg2	6.57933	95.626	0.00576	0.0138	1	ENSRNO	protein_coding
Neurl1b	-1.5053	-2.8389	9.7E-14	8.3E-13	1.9E-09	ENSRNO	protein_coding
Ebf1	-7.4899	-179.75	9.7E-46	2.8E-44	1.8E-41	ENSRNO	protein_coding
Gabra1	-2.2509	-4.7599	0.01652	0.03503	1	ENSRNO	protein_coding
C1qtnf2	-2.9562	-7.7609	4.5E-19	5.1E-18	8.5E-15	ENSRNO	protein_coding
Adam19	-3.5351	-11.592	1E-220	3E-218	3E-216	ENSRNO	protein_coding
Cyfip2	-3.7173	-13.153	8E-06	3.2E-05	0.15148	ENSRNO	protein_coding
Sgcd	-2.5165	-5.722	1.4E-31	2.7E-30	2.6E-27	ENSRNO	protein_coding
Col23a1_	-2.1961	-4.5824	0.00029	0.0009	1	ENSRNO	protein_coding
Nhp2	1.34986	2.54887	3E-12	2.3E-11	5.7E-08	ENSRNO	protein_coding
LOC1036	1.48778	2.80457	3.1E-38	7.5E-37	6E-34	ENSRNO	protein_coding
Flt4	-8.7958	-444.42	3.5E-96	2.5E-94	6.7E-92	ENSRNO	protein_coding
Gfpt2	2.20972	4.62584	0.0061	0.01452	1	ENSRNO	protein_coding
Sqstm1	1.39876	2.63675	9.3E-43	2.5E-41	1.8E-38	ENSRNO	protein_coding
Adamts2	-8.6866	-412.04	5.8E-27	9.6E-26	1.1E-22	ENSRNO	protein_coding
Zfp354c	-5.6171	-49.081	7.9E-33	1.6E-31	1.5E-28	ENSRNO	protein_coding
Zfp879	-8.081	-270.78	0.00086	0.00245	1	ENSRNO	protein_coding
Zfp2	-3.3011	-9.8567	9.8E-13	7.7E-12	1.9E-08	ENSRNO	protein_coding
Znf354b	-2.5809	-5.9832	0.00088	0.00249	1	ENSRNO	protein_coding
Col23a1_	-3.2278	-9.3687	1.5E-19	1.7E-18	2.8E-15	ENSRNO	protein_coding
Phykpl	6.66438	101.433	0.00924	0.02096	1	ENSRNO	protein_coding
N4bp3	1.56144	2.95149	0.00022	0.00068	1	ENSRNO	protein_coding
Tcf7	1.45633	2.74409	0.00151	0.00407	1	ENSRNO	protein_coding
Acsl6	2.3331	5.03888	0.01754	0.03697	1	ENSRNO	protein_coding
Gpx3	-3.575	-11.917	0.00013	0.00043	1	ENSRNO	protein_coding
Fat2	-3.6522	-12.572	8.8E-12	6.4E-11	1.7E-07	ENSRNO	protein_coding
Glra1	-2.1715	-4.505	0.00012	0.00039	1	ENSRNO	protein_coding
Lypd8	7.64832	200.62	1.7E-09	1E-08	3.1E-05	ENSRNO	protein_coding
Zfp39	-5.5778	-47.761	5.1E-21	6.4E-20	9.6E-17	ENSRNO	protein_coding

Obscn	4.50317	22.6772	2.9E-26	4.7E-25	5.6E-22	ENSRNO	protein_coding
RGD1304	1.60188	3.03539	3.8E-16	3.7E-15	7.2E-12	ENSRNO	protein_coding
Nlrp3	-6.1794	-72.474	5E-118	4E-116	9E-114	ENSRNO	protein_coding
Pemt	1.39756	2.63456	0.00022	0.0007	1	ENSRNO	protein_coding
Slc47a2	-6.5789	-95.595	0.00686	0.01612	1	ENSRNO	protein_coding
Zfp287	-9.8945	-951.82	3.7E-05	0.00013	0.70797	ENSRNO	protein_coding
Pmp22	-2.394	-5.2561	3E-110	3E-108	6E-106	ENSRNO	protein_coding
Hs3st3a1	-6.1471	-70.871	0.01303	0.02846	1	ENSRNO	protein_coding
Arhgap44	-6.7376	-106.71	0.00563	0.01351	1	ENSRNO	protein_coding
AABR070	6.2398	75.5732	0.00964	0.02177	1	ENSRNO	pseudogene
Myh2	-5.4668	-44.225	3E-194	6E-192	5E-190	ENSRNO	protein_coding
Myh8	-4.433	-21.601	5.7E-07	2.6E-06	0.01088	ENSRNO	protein_coding
Gas7	-7.5157	-183	3.8E-09	2.2E-08	7.2E-05	ENSRNO	protein_coding
Usp43	-2.9173	-7.5543	0.00077	0.00221	1	ENSRNO	protein_coding
Pik3r5	3.24549	9.48395	0.01822	0.03823	1	ENSRNO	protein_coding
Myh10	-1.7864	-3.4496	4.7E-70	2.2E-68	9E-66	ENSRNO	protein_coding
Rangrf	-1.7661	-3.4014	5.7E-07	2.6E-06	0.0109	ENSRNO	protein_coding
Kcnab3	-2.2586	-4.7852	0.00122	0.00336	1	ENSRNO	protein_coding
Shbg	-1.4439	-2.7205	0.01449	0.03131	1	ENSRNO	protein_coding
Sox15	2.17494	4.51567	0.01541	0.03299	1	ENSRNO	protein_coding
Cd68	1.56674	2.96235	8.8E-07	3.9E-06	0.01667	ENSRNO	protein_coding
Tnfsf13	1.83568	3.56941	2.8E-07	1.3E-06	0.00528	ENSRNO	protein_coding
Tnfsf12	-2.1711	-4.5036	1.5E-11	1.1E-10	2.8E-07	ENSRNO	protein_coding
Fgf11	-2.6417	-6.2405	4.3E-05	0.00015	0.81437	ENSRNO	protein_coding
Tnk1	1.56428	2.95731	0.00579	0.01386	1	ENSRNO	protein_coding
Acap1	4.55698	23.539	1.5E-19	1.8E-18	2.9E-15	ENSRNO	protein_coding
LOC4979	-1.5116	-2.8512	8.1E-10	5.1E-09	1.5E-05	ENSRNO	protein_coding
Ybx2	-2.3583	-5.1277	3E-05	0.00011	0.57587	ENSRNO	protein_coding
Dlg4	-2.5732	-5.9513	8.6E-16	8.2E-15	1.6E-11	ENSRNO	protein_coding
Slc16a11	-5.5181	-45.826	7E-145	9E-143	1E-140	ENSRNO	protein_coding
Alox15	2.06034	4.17086	0.0007	0.00202	1	ENSRNO	protein_coding
Gltpd2	-1.5304	-2.8887	0.00126	0.00345	1	ENSRNO	protein_coding
Zfp3	5.00861	32.1916	0.00015	0.00048	1	ENSRNO	protein_coding
LOC6919	2.4684	5.53429	3.1E-06	1.3E-05	0.05874	ENSRNO	protein_coding
Fam64a	-1.6426	-3.1223	8.5E-23	1.2E-21	1.6E-18	ENSRNO	protein_coding
Atp2a3	3.4703	11.0832	1.6E-71	7.7E-70	3.1E-67	ENSRNO	protein_coding
P2rx5	-2.7241	-6.6074	3.2E-17	3.4E-16	6.1E-13	ENSRNO	protein_coding
Trpv1	5.4739	44.4436	0.02317	0.04721	1	ENSRNO	protein_coding
Aspa	-2.3192	-4.9905	7.3E-14	6.2E-13	1.4E-09	ENSRNO	protein_coding
Rap1gap2	2.08484	4.24228	1.2E-37	2.7E-36	2.2E-33	ENSRNO	protein_coding
Rn60_10_	-5.5634	-47.289	1.3E-19	1.5E-18	2.5E-15	ENSRNO	unprocessed_pseudogene
Serpinf1	-6.0921	-68.217	2E-147	3E-145	4E-143	ENSRNO	protein_coding
Serpinf2	2.55818	5.88966	6.7E-05	0.00023	1	ENSRNO	protein_coding
Coro6	-4.4362	-21.649	2E-26	3.2E-25	3.8E-22	ENSRNO	protein_coding
Rilp	4.40305	21.1568	2E-05	7.5E-05	0.37974	ENSRNO	protein_coding
Lgals9	4.02181	16.2438	4E-14	3.5E-13	7.6E-10	ENSRNO	protein_coding
Rn60_10_	2.40121	5.28244	0.00024	0.00076	1	ENSRNO	unprocessed_pseudogene
Aldoc	3.96769	15.6456	1.5E-17	1.6E-16	2.8E-13	ENSRNO	protein_coding
Slc46a1	-2.0983	-4.2821	1.4E-18	1.6E-17	2.8E-14	ENSRNO	protein_coding

Sarm1	-4.7384	-26.694	7.6E-11	5.2E-10	1.4E-06	ENSRNO	protein_coding
Fam58b	1.55054	2.92926	4E-14	3.5E-13	7.7E-10	ENSRNO	protein_coding
LOC4979	1.456	2.74347	3.6E-06	1.5E-05	0.06931	ENSRNO	protein_coding
Ksr1	2.27639	4.84463	8.8E-23	1.2E-21	1.7E-18	ENSRNO	protein_coding
AABR070	2.30644	4.94663	2.2E-05	8.3E-05	0.42366	ENSRNO	lincRNA
Evi2b	3.05739	8.32463	2.3E-11	1.7E-10	4.4E-07	ENSRNO	protein_coding
Rhbdl3	-3.1092	-8.6292	8.9E-12	6.5E-11	1.7E-07	ENSRNO	protein_coding
Ccl7	-2.0002	-4.0005	5.8E-09	3.3E-08	0.00011	ENSRNO	protein_coding
Slfn5	-2.164	-4.4816	2.1E-09	1.3E-08	4E-05	ENSRNO	protein_coding
LOC6890	1.93894	3.83424	9.5E-10	5.8E-09	1.8E-05	ENSRNO	protein_coding
Mmp28	1.4012	2.64122	1.2E-09	7.4E-09	2.3E-05	ENSRNO	protein_coding
Dusp14	-1.729	-3.3149	4.3E-37	9.7E-36	8.1E-33	ENSRNO	protein_coding
AABR070	3.68626	12.8729	4.9E-05	0.00017	0.93653	ENSRNO	pseudogene
AABR070	1.56903	2.96705	0.01911	0.03986	1	ENSRNO	protein_coding
Tbx2	-3.8328	-14.249	5.3E-52	1.7E-50	1E-47	ENSRNO	protein_coding
Dhx40	-2.135	-4.3924	1.5E-57	5.4E-56	2.8E-53	ENSRNO	protein_coding
Tex14	4.77552	27.3889	2E-137	2E-135	4E-133	ENSRNO	protein_coding
Sept4	2.31396	4.97244	1.1E-05	4.3E-05	0.21186	ENSRNO	protein_coding
Rnf43	-2.6683	-6.3568	3.5E-07	1.7E-06	0.00673	ENSRNO	protein_coding
Tspoap1	-7.1261	-139.69	2.1E-08	1.1E-07	0.00039	ENSRNO	protein_coding
Mks1	-3.3304	-10.059	4.6E-20	5.5E-19	8.7E-16	ENSRNO	protein_coding
Dgke	-2.1426	-4.4154	5.9E-26	9.4E-25	1.1E-21	ENSRNO	protein_coding
Tom1l1	4.30788	19.8062	2.4E-77	1.2E-75	4.6E-73	ENSRNO	protein_coding
Nme1	1.44101	2.71512	3.2E-45	9E-44	6.1E-41	ENSRNO	protein_coding
Cacna1g	-1.5111	-2.8504	0.00423	0.01043	1	ENSRNO	protein_coding
LOC1003	3.23029	9.3846	0.00158	0.00425	1	ENSRNO	protein_coding
AABR070	1.86171	3.63438	3.2E-06	1.3E-05	0.06132	ENSRNO	lincRNA
AABR070	-3.5307	-11.557	4.1E-15	3.8E-14	7.8E-11	ENSRNO	lincRNA
Col1a1	-3.6255	-12.342	1E-221	3E-219	3E-217	ENSRNO	protein_coding
Itga3	1.74722	3.35712	2.8E-68	1.3E-66	5.4E-64	ENSRNO	protein_coding
Dlx3	-3.0072	-8.0403	1.4E-18	1.6E-17	2.7E-14	ENSRNO	protein_coding
Spop	1.73115	3.31992	1.3E-49	4.1E-48	2.6E-45	ENSRNO	protein_coding
Igf2bp1	-3.065	-8.3685	1.6E-07	8E-07	0.0031	ENSRNO	protein_coding
Atp5g1	1.32201	2.50014	6.5E-19	7.4E-18	1.2E-14	ENSRNO	protein_coding
Hoxb7	2.98719	7.9293	4E-112	4E-110	8E-108	ENSRNO	protein_coding
Copz2	-1.7962	-3.4731	1.7E-19	2E-18	3.2E-15	ENSRNO	protein_coding
Sp6	-4.9144	-30.156	3.2E-31	6.1E-30	6E-27	ENSRNO	protein_coding
Scrn2	2.02727	4.07634	1.2E-14	1.1E-13	2.3E-10	ENSRNO	protein_coding
Lrrc46	2.04543	4.12795	2.8E-11	2E-10	5.4E-07	ENSRNO	protein_coding
Osbpl7	1.8649	3.64244	5E-45	1.4E-43	9.4E-41	ENSRNO	protein_coding
Tbkbp1	5.91476	60.3283	3.6E-49	1.1E-47	6.9E-45	ENSRNO	protein_coding
Mrpl45	1.38394	2.60981	1.8E-41	4.7E-40	3.5E-37	ENSRNO	protein_coding
LOC6911	1.34761	2.54491	0.00021	0.00065	1	ENSRNO	protein_coding
Cisd3	1.6103	3.05314	5.3E-27	8.7E-26	1E-22	ENSRNO	protein_coding
Ppp1r1b	1.37921	2.60126	0.00527	0.01274	1	ENSRNO	protein_coding
Thra	1.48508	2.79932	1.1E-44	3.1E-43	2.1E-40	ENSRNO	protein_coding
Nr1d1	2.02056	4.0574	7.2E-41	1.8E-39	1.4E-36	ENSRNO	protein_coding
Cdc6	1.32772	2.51006	4.6E-38	1.1E-36	8.7E-34	ENSRNO	protein_coding
Igfbp4	1.34774	2.54513	6.6E-36	1.5E-34	1.3E-31	ENSRNO	protein_coding

Tns4	5.19091	36.5276	8E-26	1.3E-24	1.5E-21	ENSRNO	protein_coding
Krt26	5.51384	45.6911	2E-155	3E-153	4E-151	ENSRNO	protein_coding
Krt28	-2.7772	-6.855	0.00114	0.00316	1	ENSRNO	protein_coding
Krtap1-5	-2.9039	-7.4847	0.01461	0.03152	1	ENSRNO	protein_coding
Krtap17-1	6.62678	98.8236	0.00603	0.01438	1	ENSRNO	protein_coding
Krt14	4.65341	25.166	5.7E-09	3.3E-08	0.00011	ENSRNO	protein_coding
Krt16	7.5917	192.899	0.00132	0.0036	1	ENSRNO	protein_coding
Jup	5.78625	55.1867	0	0	2E-307	ENSRNO	protein_coding
Dhx58	2.25981	4.78927	3.6E-08	1.9E-07	0.00068	ENSRNO	protein_coding
Stat5a	3.07164	8.40729	2E-102	1E-100	3.4E-98	ENSRNO	protein_coding
Atp6v0a1	1.42991	2.69429	1.8E-44	4.9E-43	3.3E-40	ENSRNO	protein_coding
Hsd17b1	1.57821	2.986	1.3E-05	4.9E-05	0.24451	ENSRNO	protein_coding
Mlx	1.4942	2.81707	1.9E-47	5.5E-46	3.5E-43	ENSRNO	protein_coding
Tubg2	1.52485	2.87757	0.01936	0.04032	1	ENSRNO	protein_coding
Vps25	1.3819	2.60611	1.5E-40	3.7E-39	2.8E-36	ENSRNO	protein_coding
Aoc3	1.92517	3.79783	0.00477	0.01163	1	ENSRNO	protein_coding
Tmem106	4.82073	28.2608	1.1E-16	1.1E-15	2.1E-12	ENSRNO	protein_coding
Arl4d	2.37162	5.1752	5.7E-29	1E-27	1.1E-24	ENSRNO	protein_coding
Asb16	2.06246	4.17697	0.01113	0.02471	1	ENSRNO	protein_coding
Fam171a	-6.6851	-102.9	1.9E-11	1.4E-10	3.6E-07	ENSRNO	protein_coding
Fzd2	-1.7239	-3.3033	6.1E-24	8.7E-23	1.2E-19	ENSRNO	protein_coding
Gjc1	-9.0099	-515.52	6.5E-37	1.5E-35	1.2E-32	ENSRNO	protein_coding
Rn60_10_	1.38367	2.6093	0.0205	0.04238	1	ENSRNO	antisense_RNA
Fmnl1	3.70397	13.0318	0.00668	0.01574	1	ENSRNO	protein_coding
Map3k14	1.45293	2.73764	1.1E-27	1.9E-26	2.1E-23	ENSRNO	protein_coding
Rn60_10_	2.81239	7.02448	0.00112	0.00312	1	ENSRNO	antisense_RNA
Rn60_10_	3.27515	9.68098	0.0006	0.00175	1	ENSRNO	antisense_RNA
Myl4	-1.3282	-2.511	0.00013	0.00043	1	ENSRNO	protein_coding
Itgb3	-3.6549	-12.596	0.00014	0.00047	1	ENSRNO	protein_coding
AABR070	-4.0606	-16.687	0.00259	0.00668	1	ENSRNO	protein_coding
AABR070	5.9108	60.1627	0.01403	0.03044	1	ENSRNO	lincRNA
AABR070	6.83255	113.973	0.00446	0.01096	1	ENSRNO	lincRNA
Cyb561	1.35519	2.55831	3.2E-10	2.1E-09	6.1E-06	ENSRNO	protein_coding
Kcnh6	2.70435	6.51763	3E-101	3E-99	6.6E-97	ENSRNO	protein_coding
Milr1	4.47614	22.2563	0.00085	0.00242	1	ENSRNO	protein_coding
Cep95	1.74065	3.34186	1.3E-32	2.7E-31	2.6E-28	ENSRNO	protein_coding
Slc16a6	2.08987	4.2571	0.00078	0.00223	1	ENSRNO	protein_coding
Abca9	-5.9703	-62.697	9.6E-43	2.6E-41	1.8E-38	ENSRNO	protein_coding
Abca5	-7.2831	-155.75	8.5E-39	2.1E-37	1.6E-34	ENSRNO	protein_coding
Kcnj16	-3.2359	-9.4212	0.00022	0.00069	1	ENSRNO	protein_coding
Kcnj2	-6.0053	-64.233	1E-18	1.2E-17	1.9E-14	ENSRNO	protein_coding
Sdk2	-4.1218	-17.41	0.00013	0.00042	1	ENSRNO	protein_coding
Kif19	-7.015	-129.34	4.1E-08	2.1E-07	0.00077	ENSRNO	protein_coding
Gprc5c	-5.9523	-61.921	6.5E-90	4.1E-88	1.2E-85	ENSRNO	protein_coding
Cd300lb	4.15955	17.8711	6.9E-06	2.8E-05	0.13134	ENSRNO	protein_coding
Fads6	2.15125	4.44213	1.2E-06	5.4E-06	0.02344	ENSRNO	protein_coding
Armc7	1.73186	3.32157	1.1E-18	1.2E-17	2.1E-14	ENSRNO	protein_coding
Nt5c	3.93124	15.2553	2.3E-38	5.5E-37	4.4E-34	ENSRNO	protein_coding
Gga3	1.39072	2.62209	8.5E-39	2.1E-37	1.6E-34	ENSRNO	protein_coding

Llgl2	3.16114	8.94535	2E-21	2.6E-20	3.8E-17	ENSRNO	protein_coding
Unc13d	3.602	12.1425	1.2E-08	6.8E-08	0.00023	ENSRNO	protein_coding
Trim65	1.35513	2.5582	5.8E-32	1.2E-30	1.1E-27	ENSRNO	protein_coding
Mrpl38	1.42037	2.67654	9.3E-30	1.7E-28	1.8E-25	ENSRNO	protein_coding
Evpl	4.8356	28.5536	1.1E-92	7.5E-91	2.1E-88	ENSRNO	protein_coding
Rnf157	-1.9564	-3.8809	3.5E-23	4.9E-22	6.6E-19	ENSRNO	protein_coding
Rhbdf2	1.46328	2.75735	2.8E-17	3E-16	5.4E-13	ENSRNO	protein_coding
Mxra7	-5.206	-36.912	1.1E-21	1.4E-20	2E-17	ENSRNO	protein_coding
ENSRNO	1.46334	2.75746	2.1E-23	3E-22	4E-19	ENSRNO	protein_coding
Tmc8	1.46515	2.76092	0.01823	0.03823	1	ENSRNO	protein_coding
Afmid	1.7235	3.30236	2.1E-10	1.4E-09	4.1E-06	ENSRNO	protein_coding
Dnah17	1.89945	3.7307	0.00019	0.0006	1	ENSRNO	protein_coding
Lgals3bp	2.10667	4.30696	2.1E-93	1.4E-91	4E-89	ENSRNO	protein_coding
C1qtnf1	3.79943	13.9233	2E-116	2E-114	4E-112	ENSRNO	protein_coding
Enpp7	6.45852	87.9446	0.00672	0.01583	1	ENSRNO	protein_coding
AABR070	2.0782	4.22281	0.00171	0.00456	1	ENSRNO	lincRNA
AABR070	5.48059	44.6501	0.02376	0.04828	1	ENSRNO	lincRNA
Baiap2	1.54903	2.92621	8.4E-51	2.7E-49	1.6E-46	ENSRNO	protein_coding
Fscn2	2.62178	6.15509	2.8E-05	0.0001	0.53056	ENSRNO	protein_coding
Faap100	1.53248	2.89283	2.1E-27	3.6E-26	4.1E-23	ENSRNO	protein_coding
Oxld1	1.68247	3.20976	3E-05	0.00011	0.57474	ENSRNO	protein_coding
Pcyt2	1.56516	2.95911	9E-39	2.2E-37	1.7E-34	ENSRNO	protein_coding
Sirt7	1.81176	3.51072	1.8E-51	5.7E-50	3.3E-47	ENSRNO	protein_coding
Notum	-2.6693	-6.3611	1.6E-11	1.2E-10	3E-07	ENSRNO	protein_coding
ENSRNO	1.36758	2.58037	2.8E-06	1.2E-05	0.0535	ENSRNO	protein_coding
Dcxr	-2.8781	-7.352	1.3E-05	5E-05	0.24817	ENSRNO	protein_coding
LOC1025	1.99688	3.99136	2.2E-07	1.1E-06	0.00417	ENSRNO	lincRNA
Slc16a3	1.452	2.73588	0.0041	0.01014	1	ENSRNO	protein_coding
Sectm1a	6.12992	70.031	0.01026	0.02301	1	ENSRNO	protein_coding
Hexdc	1.89517	3.71965	2.7E-26	4.4E-25	5.2E-22	ENSRNO	protein_coding
LOC6195	1.33942	2.53049	9.2E-23	1.3E-21	1.8E-18	ENSRNO	protein_coding
Rab40b	1.32345	2.50263	0.0184	0.03856	1	ENSRNO	protein_coding
LOC1009	3.4448	10.889	0.01415	0.03066	1	ENSRNO	protein_coding
Epha3	-5.1449	-35.38	1.4E-13	1.2E-12	2.6E-09	ENSRNO	protein_coding
Chmp2b	-1.34	-2.5315	2E-37	4.6E-36	3.9E-33	ENSRNO	protein_coding
Vgll3	-1.3568	-2.5612	2.2E-38	5.3E-37	4.2E-34	ENSRNO	protein_coding
Robo1	-4.7261	-26.467	6.3E-53	2.1E-51	1.2E-48	ENSRNO	protein_coding
Nrip1	-1.3873	-2.6159	8.5E-26	1.3E-24	1.6E-21	ENSRNO	protein_coding
Cxadr	-3.5383	-11.618	1.2E-66	5.1E-65	2.2E-62	ENSRNO	protein_coding
Btg3	-2.0139	-4.0386	7.3E-75	3.6E-73	1.4E-70	ENSRNO	protein_coding
Jam2	1.53354	2.89496	0.00014	0.00046	1	ENSRNO	protein_coding
App	1.92153	3.78826	1.1E-79	5.9E-78	2.1E-75	ENSRNO	protein_coding
Adamts1	-1.8651	-3.6429	9.3E-72	4.4E-70	1.8E-67	ENSRNO	protein_coding
N6amt1	-1.6072	-3.0465	1.8E-18	2E-17	3.5E-14	ENSRNO	protein_coding
Map3k7cl	-2.4398	-5.4258	1.7E-09	1E-08	3.1E-05	ENSRNO	protein_coding
RGD1562	6.4796	89.2387	2.9E-21	3.7E-20	5.4E-17	ENSRNO	protein_coding
Kcne2	3.16308	8.9574	2E-06	8.5E-06	0.03784	ENSRNO	protein_coding
AABR070	6.82712	113.545	0.0041	0.01014	1	ENSRNO	lincRNA
Sim2	7.47181	177.517	0.00162	0.00435	1	ENSRNO	protein_coding

Erg	3.22686	9.36226	4E-42	1.1E-40	7.6E-38	ENSRNO	protein_coding
LOC1009	-8.2043	-294.94	0.00163	0.00438	1	ENSRNO	protein_coding
Igsf5	3.46334	11.0298	1.5E-06	6.7E-06	0.02916	ENSRNO	protein_coding
Mx1	2.68248	6.4196	4.2E-05	0.00015	0.8046	ENSRNO	protein_coding
Fam3b	4.77781	27.4324	6E-82	3.3E-80	1.1E-77	ENSRNO	protein_coding
Tmprss2	4.66754	25.4138	4.3E-11	3E-10	8.1E-07	ENSRNO	protein_coding
Riox2	1.72728	3.31104	9.7E-28	1.7E-26	1.8E-23	ENSRNO	protein_coding
AC14113(	2.5241	5.75216	5.2E-07	2.4E-06	0.00981	ENSRNO	lincRNA
Gabrr3	2.17354	4.5113	0.00194	0.00514	1	ENSRNO	protein_coding
Tmem45a	1.53058	2.88902	7E-15	6.4E-14	1.3E-10	ENSRNO	protein_coding
Abi3bp	-3.8272	-14.194	2.8E-13	2.3E-12	5.3E-09	ENSRNO	protein_coding
AABR070	4.09767	17.1208	0.00016	0.0005	1	ENSRNO	pseudogene
Phldb2	-1.3628	-2.5719	4.4E-40	1.1E-38	8.3E-36	ENSRNO	protein_coding
Gap43	3.78053	13.7421	0.00036	0.00109	1	ENSRNO	protein_coding
Tmprss7	2.65923	6.31696	0.00163	0.00436	1	ENSRNO	protein_coding
Cd200	-5.9727	-62.802	8.2E-66	3.6E-64	1.6E-61	ENSRNO	protein_coding
Cd200r1	6.49523	90.2107	2.3E-94	1.6E-92	4.3E-90	ENSRNO	protein_coding
Boc	-2.5994	-6.0603	1.4E-06	6.3E-06	0.02746	ENSRNO	protein_coding
Igsf11	2.56364	5.91199	0.0036	0.00901	1	ENSRNO	protein_coding
Upk1b	-3.3365	-10.102	0.00039	0.00119	1	ENSRNO	protein_coding
B4galt4	-2.6355	-6.2138	1.6E-34	3.3E-33	3E-30	ENSRNO	protein_coding
Poglut1	-1.3347	-2.5222	4.9E-38	1.2E-36	9.4E-34	ENSRNO	protein_coding
Cd80	2.00622	4.01728	4.3E-11	3E-10	8.1E-07	ENSRNO	protein_coding
Pla1a	-2.8651	-7.2859	0.0003	0.00093	1	ENSRNO	protein_coding
Gpr156	2.64766	6.26649	0.00094	0.00266	1	ENSRNO	protein_coding
Stxbp5l	4.37967	20.8167	8E-11	5.5E-10	1.5E-06	ENSRNO	protein_coding
Hcls1	5.70371	52.1181	0.01738	0.03666	1	ENSRNO	protein_coding
Pdia5	-1.5123	-2.8527	1.5E-20	1.8E-19	2.8E-16	ENSRNO	protein_coding
Mylk	-1.6992	-3.2471	0.00286	0.00732	1	ENSRNO	protein_coding
Ccdc14	-5.0875	-34	3.1E-71	1.4E-69	5.8E-67	ENSRNO	protein_coding
Pigz_1	5.04493	33.0123	0.00014	0.00045	1	ENSRNO	protein_coding
LOC1009	5.68026	51.2778	0.00048	0.00141	1	ENSRNO	protein_coding
Pigz_2	3.90939	15.026	0.00028	0.00086	1	ENSRNO	protein_coding
Fam43a	2.49674	5.64407	1.6E-22	2.1E-21	3E-18	ENSRNO	protein_coding
Tmem44	4.93592	30.6098	4.4E-70	2.1E-68	8.5E-66	ENSRNO	protein_coding
Lrrc15	4.70283	26.0431	6.8E-06	2.7E-05	0.12857	ENSRNO	protein_coding
Atp13a4	3.14792	8.86378	1E-105	9E-104	2E-101	ENSRNO	protein_coding
Il13ra1	-6.6451	-100.09	2E-120	2E-118	4E-116	ENSRNO	protein_coding
Tp63	5.40178	42.2764	4E-183	8E-181	8E-179	ENSRNO	protein_coding
AABR070	1.80965	3.50557	0.01858	0.03889	1	ENSRNO	pseudogene
Lpp	-1.3757	-2.595	5.7E-14	4.9E-13	1.1E-09	ENSRNO	protein_coding
Rtp4	3.77588	13.6979	6.6E-06	2.6E-05	0.12561	ENSRNO	protein_coding
Adipoq	3.65453	12.5929	3.2E-09	1.9E-08	6.1E-05	ENSRNO	protein_coding
Kng1	-3.5013	-11.324	6E-24	8.6E-23	1.1E-19	ENSRNO	protein_coding
LOC1009	7.66288	202.655	5E-11	3.5E-10	9.5E-07	ENSRNO	protein_coding
Ephb3	-1.6685	-3.1789	1.8E-37	4.1E-36	3.4E-33	ENSRNO	protein_coding
Ece2	-3.3844	-10.442	6.9E-47	2E-45	1.3E-42	ENSRNO	protein_coding
Gp1bb_1	-1.3246	-2.5046	6.6E-25	9.9E-24	1.3E-20	ENSRNO	protein_coding
Txnrd2	-1.611	-3.0546	5.6E-25	8.4E-24	1.1E-20	ENSRNO	protein_coding

Rtn4r	-2.3576	-5.1251	1.6E-16	1.6E-15	3.1E-12	ENSRNO	protein_coding
Slc7a4	-6.8807	-117.84	0.00461	0.01129	1	ENSRNO	protein_coding
RGD1308	2.74367	6.6977	2.2E-16	2.2E-15	4.1E-12	ENSRNO	protein_coding
Ypel1	-2.1349	-4.392	4.5E-07	2.1E-06	0.00856	ENSRNO	protein_coding
Fgd4	-4.4206	-21.416	3E-07	1.4E-06	0.00574	ENSRNO	protein_coding
ENSRNO	-1.3788	-2.6005	2.7E-33	5.5E-32	5.1E-29	ENSRNO	protein_coding
LOC1025	-3.6609	-12.649	0.00056	0.00163	1	ENSRNO	lincRNA
Snai2	-1.3399	-2.5313	6.7E-26	1.1E-24	1.3E-21	ENSRNO	protein_coding
Pex11g	-1.9747	-3.9305	0.00114	0.00316	1	ENSRNO	protein_coding
Stxbp2	2.40284	5.28844	1.5E-46	4.5E-45	2.9E-42	ENSRNO	protein_coding
Mcemp1	1.74411	3.34987	0.00152	0.0041	1	ENSRNO	protein_coding
Fcer2	2.9193	7.56477	0.00887	0.02021	1	ENSRNO	protein_coding
Prr36	-3.3779	-10.396	2.1E-07	1E-06	0.00397	ENSRNO	protein_coding
AABR070	4.6893	25.8001	0.00044	0.00131	1	ENSRNO	lincRNA
AABR070	6.15887	71.4505	0.01091	0.02429	1	ENSRNO	protein_coding
Zfp958	1.77601	3.42477	2.1E-36	4.7E-35	4E-32	ENSRNO	protein_coding
Fry	3.74581	13.4153	1E-204	3E-202	2E-200	ENSRNO	protein_coding
Wdr95	5.83888	57.237	0.01479	0.03182	1	ENSRNO	protein_coding
AABR070	1.99311	3.98093	1.6E-06	7E-06	0.03069	ENSRNO	protein_coding
Alox5ap	4.22549	18.7068	0.00152	0.0041	1	ENSRNO	protein_coding
Katnal1	-2.0023	-4.0064	0.00238	0.00618	1	ENSRNO	protein_coding
Mtus2	-3.8403	-14.323	7.7E-19	8.7E-18	1.5E-14	ENSRNO	protein_coding
Pomp	1.45273	2.73726	8.7E-06	3.4E-05	0.16581	ENSRNO	protein_coding
Flt1	3.49993	11.3131	5.2E-14	4.5E-13	1E-09	ENSRNO	protein_coding
Cdx2	1.34873	2.54687	5.9E-38	1.4E-36	1.1E-33	ENSRNO	protein_coding
Pdx1	1.79214	3.46328	7.6E-08	3.9E-07	0.00145	ENSRNO	protein_coding
Wasf3	2.44326	5.43869	1.5E-13	1.3E-12	2.9E-09	ENSRNO	protein_coding
RGD1565	1.90493	3.74491	2.4E-05	8.8E-05	0.45358	ENSRNO	processed_pseudogene
Ocm2	6.12882	69.9773	0	0	0	ENSRNO	protein_coding
Fscn1	-7.8319	-227.85	2E-166	3E-164	4E-162	ENSRNO	protein_coding
Slc29a4	1.90898	3.75545	1.9E-10	1.3E-09	3.7E-06	ENSRNO	protein_coding
Tmem184	4.33093	20.1252	0.00117	0.00323	1	ENSRNO	protein_coding
Gper1	5.19462	36.6216	2.1E-17	2.2E-16	3.9E-13	ENSRNO	protein_coding
Asmt	2.18936	4.56103	0.01176	0.02599	1	ENSRNO	protein_coding
Il3ra	2.89362	7.43134	1E-25	1.6E-24	2E-21	ENSRNO	protein_coding
Cyp3a9_1	2.47336	5.55335	1.4E-05	5.3E-05	0.26478	ENSRNO	protein_coding
Gal3st4	2.75302	6.74126	0.0153	0.0328	1	ENSRNO	protein_coding
Vom2r62	6.45851	87.9436	0.0068	0.016	1	ENSRNO	protein_coding
LOC6809	7.38545	167.202	0.00193	0.0051	1	ENSRNO	protein_coding
AABR070	-1.9892	-3.9703	0.00017	0.00055	1	ENSRNO	lincRNA
AABR070	-4.1236	-17.431	1.1E-39	2.8E-38	2.2E-35	ENSRNO	lincRNA
AABR070	-3.5589	-11.785	3E-08	1.6E-07	0.00057	ENSRNO	lincRNA
AABR070	-5.3747	-41.489	2.2E-07	1.1E-06	0.00414	ENSRNO	lincRNA
AABR070	-4.0485	-16.547	1.8E-16	1.8E-15	3.5E-12	ENSRNO	lincRNA
AABR070	-3.7091	-13.078	1.8E-07	8.8E-07	0.00344	ENSRNO	lincRNA
AABR070	-2.7388	-6.6753	2.3E-05	8.6E-05	0.44029	ENSRNO	lincRNA
AABR070	-2.6844	-6.4283	2E-10	1.3E-09	3.8E-06	ENSRNO	lincRNA
AABR070	-3.3237	-10.012	6.4E-13	5.1E-12	1.2E-08	ENSRNO	lincRNA
AABR070	-1.556	-2.9404	3.3E-05	0.00012	0.6224	ENSRNO	lincRNA

Ufsp1	1.83966	3.57926	6.3E-06	2.5E-05	0.11992	ENSRNO	protein_coding
Orai2	1.73965	3.33953	7.8E-56	2.8E-54	1.5E-51	ENSRNO	protein_coding
Rasa4	4.43271	21.5962	9.9E-25	1.5E-23	1.9E-20	ENSRNO	protein_coding
Upk3b	-4.1888	-18.238	8.3E-05	0.00028	1	ENSRNO	protein_coding
Cldn4	5.66199	50.6326	1.3E-05	5.1E-05	0.2506	ENSRNO	protein_coding
Wbscr27	3.04241	8.23865	2.1E-19	2.5E-18	4.1E-15	ENSRNO	protein_coding
Lat2	2.14932	4.4362	0.0053	0.0128	1	ENSRNO	protein_coding
Wbscr17	-7.5124	-182.59	1.2E-40	2.9E-39	2.2E-36	ENSRNO	protein_coding
Scarb1	1.70338	3.25662	1.7E-59	6.5E-58	3.2E-55	ENSRNO	protein_coding
Rflna	3.24546	9.48376	0.01853	0.03879	1	ENSRNO	protein_coding
Pitpnm2	-1.9508	-3.866	6.7E-75	3.3E-73	1.3E-70	ENSRNO	protein_coding
Hcar2	3.30973	9.91582	8.9E-12	6.6E-11	1.7E-07	ENSRNO	protein_coding
Bcl7a	-2.938	-7.6635	1.7E-19	2E-18	3.2E-15	ENSRNO	protein_coding
AABR070	-1.6247	-3.0837	0.01415	0.03067	1	ENSRNO	pseudogene
Hvcn1	7.3722	165.673	0.00196	0.00517	1	ENSRNO	protein_coding
Cux2	5.55793	47.1091	2.3E-05	8.4E-05	0.43457	ENSRNO	protein_coding
LOC1003	5.8429	57.3971	0.01534	0.03286	1	ENSRNO	protein_coding
Tmem116	2.06075	4.17203	0.00036	0.00108	1	ENSRNO	protein_coding
Oas1g	1.51853	2.86498	1.8E-07	9E-07	0.00352	ENSRNO	protein_coding
Oas1a	2.61547	6.12823	2.8E-05	0.0001	0.53051	ENSRNO	protein_coding
Iqcd	3.97419	15.7163	7.8E-55	2.7E-53	1.5E-50	ENSRNO	protein_coding
Slc8b1	1.39732	2.63412	1.3E-40	3.3E-39	2.5E-36	ENSRNO	protein_coding
Med13l	-1.3947	-2.6293	6E-43	1.6E-41	1.1E-38	ENSRNO	protein_coding
Hspb8	4.35447	20.4562	1.4E-06	6.3E-06	0.02736	ENSRNO	protein_coding
Pla2g1b	5.77275	54.6728	0.01656	0.0351	1	ENSRNO	protein_coding
Hnf1a	3.34965	10.194	0.01668	0.03533	1	ENSRNO	protein_coding
Oasl	2.29638	4.91224	4.6E-13	3.7E-12	8.7E-09	ENSRNO	protein_coding
Oasl2	3.09216	8.5277	1.9E-44	5.3E-43	3.7E-40	ENSRNO	protein_coding
AABR070	3.3176	9.97007	0.00045	0.00134	1	ENSRNO	lincRNA
Acacb	1.96754	3.911	0.00128	0.00349	1	ENSRNO	protein_coding
Tmem119	-3.8819	-14.743	6E-279	3E-276	1E-274	ENSRNO	protein_coding
Sgsm1	3.69637	12.9634	2.4E-87	1.4E-85	4.5E-83	ENSRNO	protein_coding
RGD1306	-1.5467	-2.9215	2.3E-36	5.1E-35	4.4E-32	ENSRNO	protein_coding
Crybb1	-6.4062	-84.811	0.00912	0.02072	1	ENSRNO	protein_coding
Cryba4	-3.5098	-11.391	5E-07	2.3E-06	0.00956	ENSRNO	protein_coding
Ttc28	-1.3405	-2.5324	7.2E-38	1.7E-36	1.4E-33	ENSRNO	protein_coding
Dsel	-5.114	-34.631	3.2E-45	9.1E-44	6.1E-41	ENSRNO	protein_coding
Rnf152	3.45319	10.9525	2.9E-07	1.4E-06	0.0055	ENSRNO	protein_coding
Serpinb11	5.55997	47.1756	0.02191	0.04496	1	ENSRNO	protein_coding
Serpinb7	4.10892	17.2547	2.9E-51	9.2E-50	5.5E-47	ENSRNO	protein_coding
Serpinb2	5.54927	46.8271	2.5E-54	8.4E-53	4.7E-50	ENSRNO	protein_coding
Serpinb8	6.37991	83.2806	3E-251	1E-248	7E-247	ENSRNO	protein_coding
Tfcp2l1	-4.5621	-23.623	2.7E-24	3.9E-23	5E-20	ENSRNO	protein_coding
Gli2	-4.9889	-31.755	1.6E-80	8.3E-79	3E-76	ENSRNO	protein_coding
Tmem185	1.33091	2.51561	1E-34	2.2E-33	2E-30	ENSRNO	protein_coding
Htr5b	5.2365	37.7002	1.8E-94	1.2E-92	3.4E-90	ENSRNO	protein_coding
Nckap5	5.71105	52.3838	1.5E-05	5.8E-05	0.29171	ENSRNO	protein_coding
LOC1009	-6.8438	-114.86	0.0088	0.02007	1	ENSRNO	protein_coding
Tmem163	4.8039	27.9329	0.00026	0.00082	1	ENSRNO	protein_coding

Map3k19	-1.8515	-3.6088	4.6E-06	1.9E-05	0.0882	ENSRNO	protein_coding
Dyrk3	1.40275	2.64404	2.3E-10	1.5E-09	4.4E-06	ENSRNO	protein_coding
Rab7b	4.13894	17.6175	2.8E-13	2.3E-12	5.3E-09	ENSRNO	protein_coding
Slc26a9	2.8493	7.20648	1.1E-05	4.3E-05	0.21127	ENSRNO	protein_coding
Cdk18	3.41594	10.6734	7.7E-22	1E-20	1.5E-17	ENSRNO	protein_coding
Nfasc	7.35191	163.36	1.3E-23	1.9E-22	2.5E-19	ENSRNO	protein_coding
Lrrn2	3.73144	13.2823	8.3E-05	0.00028	1	ENSRNO	protein_coding
Pik3c2b	2.44062	5.42876	2.1E-38	5.1E-37	4.1E-34	ENSRNO	protein_coding
AABR070	7.77146	218.496	0.00106	0.00294	1	ENSRNO	lincRNA
Atp2b4	2.81373	7.031	1E-130	1E-128	2E-126	ENSRNO	protein_coding
Sox13	6.25953	76.6138	1.2E-50	3.7E-49	2.2E-46	ENSRNO	protein_coding
Plekha6	5.30041	39.4078	6.6E-92	4.2E-90	1.2E-87	ENSRNO	processed_transcript
Fmod	2.03233	4.09065	9.2E-70	4.2E-68	1.8E-65	ENSRNO	protein_coding
Chit1	5.8429	57.3971	0.01532	0.03283	1	ENSRNO	protein_coding
Adora1	2.30671	4.94753	0.00023	0.00072	1	ENSRNO	protein_coding
Lgr6	3.17344	9.02197	8.1E-10	5E-09	1.5E-05	ENSRNO	protein_coding
Ptprv	3.73441	13.3098	8E-55	2.8E-53	1.5E-50	ENSRNO	protein_coding
Ptpn7	5.94302	61.5216	2E-193	4E-191	4E-189	ENSRNO	protein_coding
Lmod1	-5.5647	-47.33	3.3E-26	5.2E-25	6.2E-22	ENSRNO	protein_coding
Tnnt2	-1.6242	-3.0828	0.01194	0.02634	1	ENSRNO	protein_coding
Cacna1s	4.52955	23.0957	1.7E-05	6.3E-05	0.31781	ENSRNO	protein_coding
Kif21b	1.42483	2.68483	1.2E-12	9.6E-12	2.3E-08	ENSRNO	protein_coding
Lhx9	1.76349	3.39518	0.0047	0.01149	1	ENSRNO	protein_coding
Crb1	5.23709	37.7156	8.3E-16	8E-15	1.6E-11	ENSRNO	protein_coding
Rgs2	2.2346	4.70631	5E-09	2.9E-08	9.5E-05	ENSRNO	protein_coding
Rgs21	5.11303	34.6079	0.00014	0.00045	1	ENSRNO	protein_coding
AABR070	7.01004	128.894	0.00311	0.0079	1	ENSRNO	lincRNA
Rgs18	5.71499	52.5269	2.2E-49	6.8E-48	4.2E-45	ENSRNO	protein_coding
Prg4	2.81116	7.01848	1.7E-05	6.5E-05	0.32857	ENSRNO	protein_coding
Hmcn1	1.59596	3.02295	0.00683	0.01605	1	ENSRNO	protein_coding
Colgalt2	7.01008	128.898	0.0032	0.0081	1	ENSRNO	protein_coding
Ncf2	4.50163	22.6531	1.5E-13	1.3E-12	2.8E-09	ENSRNO	processed_transcript
Nmnat2	1.75418	3.37335	1.5E-07	7.3E-07	0.00284	ENSRNO	protein_coding
Lamc2	-1.9196	-3.7833	1.3E-70	6.2E-69	2.5E-66	ENSRNO	protein_coding
Npl	2.23951	4.72235	1.4E-14	1.2E-13	2.6E-10	ENSRNO	protein_coding
Rnasel	1.93282	3.81801	2.6E-27	4.4E-26	5E-23	ENSRNO	protein_coding
Cacna1e	3.62315	12.3219	0.00729	0.01702	1	ENSRNO	protein_coding
Mr1	1.95131	3.86725	0.00029	0.0009	1	ENSRNO	protein_coding
Qsox1	-6.3213	-79.966	2.8E-40	6.9E-39	5.3E-36	ENSRNO	protein_coding
Tdrd5	3.98818	15.8695	0.00273	0.00701	1	ENSRNO	protein_coding
Tnr	1.52454	2.87695	0.00558	0.01342	1	ENSRNO	protein_coding
Tnn	-5.0834	-33.903	1E-192	2E-190	2E-188	ENSRNO	protein_coding
Rn50_13_	-2.9487	-7.7204	7.5E-05	0.00025	1	ENSRNO	lincRNA
Tnfsf18	-2.2431	-4.7341	2E-10	1.3E-09	3.8E-06	ENSRNO	protein_coding
Dnm3	1.7916	3.46198	4E-08	2.1E-07	0.00077	ENSRNO	protein_coding
Rn50_13_	4.08685	16.9928	9E-16	8.6E-15	1.7E-11	ENSRNO	antisense_RNA
Mir199a2_	-1.7393	-3.3386	0.00137	0.00372	1	ENSRNO	protein_coding
Myoc	3.24549	9.48395	0.01818	0.03813	1	ENSRNO	protein_coding
Fmo1	-2.6666	-6.3494	2.6E-05	9.5E-05	0.49332	ENSRNO	protein_coding

Fmo3	-1.3785	-2.6	0.00419	0.01034	1	ENSRNO	protein_coding
F5	5.39241	42.0027	2.4E-27	4E-26	4.6E-23	ENSRNO	protein_coding
Gpr161	-1.917	-3.7764	2.2E-20	2.7E-19	4.2E-16	ENSRNO	protein_coding
Adcy10	6.59696	96.802	2.5E-24	3.7E-23	4.8E-20	ENSRNO	protein_coding
Creg1	3.38971	10.481	0.00223	0.00583	1	ENSRNO	protein_coding
Dusp27	-4.1119	-17.29	1.5E-64	6.6E-63	2.9E-60	ENSRNO	protein_coding
Fmo9	1.37311	2.59029	1.6E-06	6.9E-06	0.03047	ENSRNO	protein_coding
Rn50_13_	-3.3348	-10.09	0.00327	0.00828	1	ENSRNO	lincRNA
Mgst3	2.07758	4.221	5.1E-32	1E-30	9.7E-28	ENSRNO	protein_coding
Rxrg	2.79934	6.96124	0.0007	0.00202	1	ENSRNO	protein_coding
AABR070	1.95795	3.88511	3.9E-08	2E-07	0.00073	ENSRNO	lincRNA
Sh2d1b2	5.15841	35.7139	8.5E-20	1E-18	1.6E-15	ENSRNO	protein_coding
Sh2d1b	1.50233	2.83299	2.5E-12	1.9E-11	4.7E-08	ENSRNO	protein_coding
Olfml2b	-1.9461	-3.8533	3.1E-64	1.3E-62	6E-60	ENSRNO	protein_coding
Fcrlb	2.31061	4.96091	0.0007	0.00202	1	ENSRNO	protein_coding
Fcrla	4.12984	17.5067	2.7E-47	8.1E-46	5.2E-43	ENSRNO	protein_coding
Pcp4l1	1.9993	3.99807	6.2E-05	0.00021	1	ENSRNO	protein_coding
Klhdc9	4.35174	20.4176	8.7E-07	3.9E-06	0.0166	ENSRNO	protein_coding
Arhgap30	3.37138	10.3487	1.6E-19	1.9E-18	3E-15	ENSRNO	protein_coding
F11r	5.06359	33.442	5.2E-85	3E-83	9.9E-81	ENSRNO	protein_coding
Itln1	5.77298	54.6814	0.01632	0.03468	1	ENSRNO	protein_coding
Cd244	3.63504	12.4239	2.5E-13	2.1E-12	4.8E-09	ENSRNO	protein_coding
Ly9	2.33879	5.05879	2.7E-13	2.3E-12	5.2E-09	ENSRNO	protein_coding
AABR070	1.58216	2.99418	9.5E-05	0.00032	1	ENSRNO	lincRNA
Vangl2	4.38094	20.8351	0.00101	0.00283	1	ENSRNO	protein_coding
Atp1a4	1.52512	2.87811	0.00288	0.00737	1	ENSRNO	protein_coding
Dusp23	1.60892	3.05023	0.01751	0.03692	1	ENSRNO	protein_coding
LOC1009	-2.6085	-6.0986	2E-127	2E-125	4E-123	ENSRNO	protein_coding
LOC1083	-3.696	-12.96	2.9E-97	2.1E-95	5.5E-93	ENSRNO	protein_coding
Mnda	2.37359	5.18228	1.1E-38	2.7E-37	2.2E-34	ENSRNO	protein_coding
Spta1	1.68731	3.22055	5.1E-09	2.9E-08	9.7E-05	ENSRNO	protein_coding
Rgs7	5.09352	34.1431	1.1E-08	6.1E-08	0.00021	ENSRNO	protein_coding
Wdr64	5.60117	48.5421	1.2E-28	2.1E-27	2.3E-24	ENSRNO	protein_coding
Pld5	6.57924	95.6202	0.00582	0.01392	1	ENSRNO	protein_coding
Itpkb	-3.1256	-8.7275	1.1E-20	1.4E-19	2.1E-16	ENSRNO	protein_coding
Capn8	6.46847	88.5533	7E-152	1E-149	1E-147	ENSRNO	protein_coding
Susd4	7.23017	150.14	6E-141	7E-139	1E-136	ENSRNO	protein_coding
Tlr5	5.34843	40.7416	1.3E-29	2.3E-28	2.5E-25	ENSRNO	protein_coding
AABR070	1.38524	2.61216	0.01702	0.03599	1	ENSRNO	lincRNA
Mark1	-2.0171	-4.0476	1.7E-27	2.9E-26	3.2E-23	ENSRNO	protein_coding
Dusp10	2.3787	5.20069	3E-33	6.1E-32	5.6E-29	ENSRNO	protein_coding
Tgfb2	-2.0075	-4.0208	1.3E-85	7.3E-84	2.4E-81	ENSRNO	protein_coding
Kcnk2	3.32137	9.99611	8.1E-36	1.8E-34	1.5E-31	ENSRNO	protein_coding
Atf3	3.45899	10.9966	2.3E-13	1.9E-12	4.4E-09	ENSRNO	protein_coding
Traf3ip3	5.70382	52.1219	0.01733	0.03658	1	ENSRNO	protein_coding
Hsd11b1	-1.4057	-2.6494	2E-07	9.5E-07	0.00372	ENSRNO	protein_coding
Lamb3	4.44861	21.8355	0	0	0	ENSRNO	protein_coding
AABR070	3.73727	13.3361	0.00064	0.00186	1	ENSRNO	lincRNA
Rnf212	-5.8854	-59.114	6.4E-09	3.7E-08	0.00012	ENSRNO	protein_coding

Fgfrl1	-1.8313	-3.5587	7.7E-22	1E-20	1.5E-17	ENSRNO	protein_coding
Cplx1	5.63112	49.5605	0.01884	0.03938	1	ENSRNO	protein_coding
Mfsd7	1.57718	2.98387	6.2E-07	2.8E-06	0.01178	ENSRNO	protein_coding
Gfi1	2.19367	4.57467	1.3E-11	9.2E-11	2.4E-07	ENSRNO	protein_coding
Brdt	8.30924	317.199	1.7E-49	5.4E-48	3.3E-45	ENSRNO	protein_coding
Lrrc8d	1.91268	3.76507	9.7E-40	2.4E-38	1.8E-35	ENSRNO	protein_coding
Mpa2l	-1.6338	-3.1032	0.00092	0.00261	1	ENSRNO	protein_coding
LOC1003	-1.3579	-2.563	0.00015	0.00048	1	ENSRNO	protein_coding
LOC6798	1.36144	2.56941	1.4E-26	2.3E-25	2.7E-22	ENSRNO	protein_coding
Mepe	5.49093	44.9714	3E-257	1E-254	7E-253	ENSRNO	protein_coding
Dmp1	-1.952	-3.8692	0.01769	0.03724	1	ENSRNO	protein_coding
AABR070	1.96205	3.89616	0.02258	0.04623	1	ENSRNO	pseudogene
Mapk10	2.13444	4.39065	2E-08	1.1E-07	0.00038	ENSRNO	protein_coding
Arhgap24	4.06135	16.695	6.5E-23	9E-22	1.2E-18	ENSRNO	protein_coding
Cds1	-2.0189	-4.0528	7.8E-09	4.4E-08	0.00015	ENSRNO	protein_coding
Hpse	5.21364	37.1074	2.1E-97	1.6E-95	4E-93	ENSRNO	protein_coding
Prkg2	7.38117	166.707	6.7E-30	1.2E-28	1.3E-25	ENSRNO	protein_coding
Bmp3	5.65861	50.5138	4.1E-16	4E-15	7.9E-12	ENSRNO	protein_coding
Prdm8	2.19381	4.57513	1.9E-33	4E-32	3.7E-29	ENSRNO	protein_coding
Anxa3	-4.2183	-18.614	2E-238	5E-236	4E-234	ENSRNO	protein_coding
Sowahb	5.0771	33.7566	1.2E-93	8.4E-92	2.3E-89	ENSRNO	protein_coding
Shroom3	5.23367	37.6264	1.3E-34	2.7E-33	2.4E-30	ENSRNO	protein_coding
Art3	1.87679	3.67257	0.0078	0.01803	1	ENSRNO	protein_coding
Cxcl11	2.79573	6.94382	1.4E-05	5.3E-05	0.26097	ENSRNO	protein_coding
Cxcl10	1.33894	2.52965	3.3E-26	5.4E-25	6.4E-22	ENSRNO	protein_coding
Naaa	2.63193	6.19856	1.6E-05	5.9E-05	0.29595	ENSRNO	protein_coding
Parm1	1.90385	3.74211	2.1E-46	6.1E-45	4E-42	ENSRNO	protein_coding
Areg	-1.9639	-3.9012	1.2E-69	5.4E-68	2.2E-65	ENSRNO	protein_coding
Epgn	-3.5015	-11.326	1.2E-49	3.7E-48	2.3E-45	ENSRNO	protein_coding
Mthfd2l	-1.3506	-2.5502	8.7E-11	5.9E-10	1.7E-06	ENSRNO	protein_coding
Cxcl2	3.984	15.8236	0.00345	0.00868	1	ENSRNO	protein_coding
Cxcl3	-1.5522	-2.9326	6.7E-07	3.1E-06	0.01275	ENSRNO	protein_coding
Afm	4.01402	16.1563	0.00018	0.00057	1	ENSRNO	protein_coding
Afp	7.05491	132.965	2.6E-08	1.4E-07	0.00049	ENSRNO	protein_coding
Sult1d1	6.76054	108.424	0.00444	0.01093	1	ENSRNO	protein_coding
Sult1b1	3.57305	11.9013	3.9E-13	3.1E-12	7.4E-09	ENSRNO	protein_coding
Ugt2a1	2.98917	7.94018	0.00122	0.00336	1	ENSRNO	protein_coding
RGD1559	4.4772	22.2726	0.00088	0.00249	1	ENSRNO	protein_coding
Hopx	5.68911	51.5934	2.5E-23	3.5E-22	4.8E-19	ENSRNO	protein_coding
Thegl	3.73121	13.2803	1.5E-07	7.4E-07	0.00284	ENSRNO	protein_coding
RGD1311	6.98978	127.097	2E-106	2E-104	4E-102	ENSRNO	protein_coding
Pdcl2	-4.549	-23.41	1E-05	3.9E-05	0.19217	ENSRNO	protein_coding
Kdr	7.01006	128.896	0.00311	0.00791	1	ENSRNO	protein_coding
Kit	5.33198	40.2798	1E-30	2E-29	2E-26	ENSRNO	protein_coding
Spata18	-2.0468	-4.1318	0.00022	0.00071	1	ENSRNO	protein_coding
Txk	2.20788	4.61997	1.1E-07	5.6E-07	0.00214	ENSRNO	protein_coding
Nipal1	4.88422	29.5322	1.2E-59	4.8E-58	2.4E-55	ENSRNO	protein_coding
Atp8a1_1	3.02196	8.12272	4.8E-06	2E-05	0.0909	ENSRNO	protein_coding
Atp8a1_2	2.7418	6.68906	9.2E-48	2.8E-46	1.7E-43	ENSRNO	protein_coding

Limch1	6.51744	91.6104	1.5E-80	8E-79	2.8E-76	ENSRNO	protein_coding
Uchl1	3.69433	12.9451	1E-148	2E-146	2E-144	ENSRNO	protein_coding
AC11262	2.66158	6.32727	0.00474	0.01156	1	ENSRNO	protein_coding
LOC1003	6.51033	91.16	0.00867	0.01981	1	ENSRNO	protein_coding
Rpl9	1.50343	2.83516	2.3E-06	9.7E-06	0.04325	ENSRNO	protein_coding
AABR070	-3.4666	-11.055	2.2E-05	8.2E-05	0.42272	ENSRNO	pseudogene
Tmem156	3.704	13.0321	0.00673	0.01584	1	ENSRNO	protein_coding
Fam114a	-1.8732	-3.6634	2.8E-67	1.2E-65	5.2E-63	ENSRNO	protein_coding
Tlr10	3.64867	12.5418	0.01064	0.02376	1	ENSRNO	protein_coding
LOC4983	4.5373	23.2201	4.2E-50	1.3E-48	8E-46	ENSRNO	protein_coding
Arap2	3.48249	11.1772	7.2E-23	1E-21	1.4E-18	ENSRNO	protein_coding
Pcdh7	-7.5891	-192.55	0.00178	0.00473	1	ENSRNO	protein_coding
LOC1003	5.95524	62.0449	0.0167	0.03537	1	ENSRNO	protein_coding
Gnpda2	-4.2578	-19.131	3.1E-26	5E-25	5.9E-22	ENSRNO	protein_coding
Ppargc1a	5.36788	41.2946	3.8E-62	1.5E-60	7.1E-58	ENSRNO	protein_coding
Bst1	-3.0579	-8.3276	3.6E-19	4.2E-18	6.9E-15	ENSRNO	protein_coding
Hs3st1	4.70031	25.9976	2.1E-86	1.2E-84	4E-82	ENSRNO	protein_coding
Slc2a9	-1.8405	-3.5813	1.1E-09	6.5E-09	2E-05	ENSRNO	protein_coding
Msx1	-8.3635	-329.35	3.2E-25	4.9E-24	6.1E-21	ENSRNO	protein_coding
Evc2	-1.5013	-2.831	3.9E-20	4.8E-19	7.5E-16	ENSRNO	protein_coding
Crmp1	5.46639	44.2127	1.4E-10	9.6E-10	2.7E-06	ENSRNO	protein_coding
Jakmip1	3.98826	15.8703	0.00298	0.00759	1	ENSRNO	protein_coding
Sorcs2	1.39704	2.6336	7.8E-41	2E-39	1.5E-36	ENSRNO	protein_coding
Msantd1	1.38337	2.60878	0.00078	0.00224	1	ENSRNO	protein_coding
Nat8l	5.8721	58.5703	9.5E-09	5.3E-08	0.00018	ENSRNO	protein_coding
Slc5a1	3.4464	10.9011	0.01391	0.03019	1	ENSRNO	protein_coding
Patz1	1.76213	3.39199	7.9E-09	4.5E-08	0.00015	ENSRNO	protein_coding
Inpp5j	3.74156	13.3758	4.4E-47	1.3E-45	8.3E-43	ENSRNO	protein_coding
Selenom	-1.549	-2.9261	4.1E-31	7.8E-30	7.8E-27	ENSRNO	protein_coding
Osbp2	-6.1607	-71.542	1.1E-24	1.6E-23	2.1E-20	ENSRNO	protein_coding
Sec14l4	5.89676	59.58	0.01414	0.03064	1	ENSRNO	protein_coding
Gatsl3	-4.0239	-16.267	1.5E-17	1.6E-16	2.8E-13	ENSRNO	protein_coding
Lif	1.40733	2.65247	1E-12	7.9E-12	1.9E-08	ENSRNO	protein_coding
Nipsnap1	-5.7757	-54.784	5E-91	3.2E-89	9.4E-87	ENSRNO	protein_coding
Nefh	2.44819	5.45731	0.00554	0.01332	1	ENSRNO	protein_coding
Ankrd36	1.77966	3.43346	3.4E-09	2E-08	6.4E-05	ENSRNO	protein_coding
AABR070	-4.6397	-24.928	3E-07	1.4E-06	0.00577	ENSRNO	lincRNA
Aebp1	-6.8678	-116.79	6E-202	1E-199	1E-197	ENSRNO	protein_coding
Gck	5.89129	59.3548	0.01476	0.03178	1	ENSRNO	protein_coding
Camk2b	2.08253	4.23549	0.0003	0.00091	1	ENSRNO	protein_coding
Nacad	-2.4444	-5.4428	2.9E-16	2.8E-15	5.5E-12	ENSRNO	protein_coding
Ramp3	-4.6209	-24.605	1.8E-17	1.9E-16	3.4E-13	ENSRNO	protein_coding
Pkd1l1	-3.4864	-11.208	1.1E-33	2.2E-32	2E-29	ENSRNO	protein_coding
LOC6885	2.94404	7.69564	0.00066	0.00192	1	ENSRNO	protein_coding
Abca13	7.40349	169.307	0.00183	0.00486	1	ENSRNO	protein_coding
Vwc2	4.43806	21.6764	6E-11	4.1E-10	1.1E-06	ENSRNO	protein_coding
Zpbp	5.00965	32.2147	2.1E-28	3.7E-27	4.1E-24	ENSRNO	protein_coding
RGD1309	8.89787	477.007	0.00016	0.00052	1	ENSRNO	protein_coding
Grb10	-3.845	-14.37	0.0003	0.00093	1	ENSRNO	protein_coding

Cobl	-2.0591	-4.1672	2.2E-81	1.2E-79	4.1E-77	ENSRNO	protein_coding
Meis1	-2.0406	-4.1141	5E-79	2.6E-77	9.5E-75	ENSRNO	protein_coding
AABR070	4.30083	19.7096	3.8E-89	2.4E-87	7.2E-85	ENSRNO	lincRNA
AABR070	1.8182	3.5264	0.00083	0.00238	1	ENSRNO	lincRNA
Tmem17	-5.9933	-63.702	2.5E-23	3.6E-22	4.8E-19	ENSRNO	protein_coding
Zrsr1	4.98472	31.663	3.8E-55	1.3E-53	7.3E-51	ENSRNO	protein_coding
Fam161a	5.26737	38.5156	9.4E-25	1.4E-23	1.8E-20	ENSRNO	protein_coding
Clhc1	2.11513	4.3323	0.00033	0.001	1	ENSRNO	protein_coding
Gpr75	-5.4326	-43.19	3.3E-27	5.4E-26	6.2E-23	ENSRNO	protein_coding
Zfp503	1.5527	2.93366	1.3E-12	1.1E-11	2.5E-08	ENSRNO	protein_coding
Dusp13_1	1.95952	3.88934	0.01954	0.04066	1	ENSRNO	protein_coding
Cfap70	3.61951	12.2908	0.00012	0.00039	1	ENSRNO	protein_coding
Gng2	1.49127	2.81137	1.5E-18	1.7E-17	2.9E-14	ENSRNO	protein_coding
Nid2	1.63344	3.10252	8.3E-51	2.6E-49	1.6E-46	ENSRNO	protein_coding
AABR070	4.56528	23.6748	0.00057	0.00168	1	ENSRNO	lincRNA
AABR070	6.22285	74.6901	2.6E-10	1.7E-09	5E-06	ENSRNO	protein_coding
AABR070	4.24881	19.0117	1.3E-08	7E-08	0.00024	ENSRNO	lincRNA
LOC1003	3.25864	9.57077	0.00031	0.00095	1	ENSRNO	protein_coding
Bmp4	5.83776	57.1928	1E-33	2.2E-32	2E-29	ENSRNO	protein_coding
Ccnb1ip1	6.91852	120.971	4E-105	3E-103	8E-101	ENSRNO	protein_coding
Rnase4	1.63683	3.10982	3.4E-23	4.8E-22	6.4E-19	ENSRNO	protein_coding
AC11434:	3.04093	8.23023	0.00171	0.00458	1	ENSRNO	protein_coding
Rnase1l1	5.80664	55.9724	1E-250	5E-248	3E-246	ENSRNO	protein_coding
LOC1036	5.89735	59.6047	0	0	0	ENSRNO	protein_coding
LOC1036	5.8159	56.3328	0	0	0	ENSRNO	protein_coding
Sall2	-4.1311	-17.522	3.8E-26	6.1E-25	7.2E-22	ENSRNO	protein_coding
Rem2	-3.9343	-15.288	2E-06	8.6E-06	0.03808	ENSRNO	protein_coding
Efs	1.39405	2.62815	2.3E-09	1.4E-08	4.5E-05	ENSRNO	protein_coding
Ap1g2	3.16306	8.95729	9.6E-52	3.1E-50	1.8E-47	ENSRNO	protein_coding
Dhrs2	4.23545	18.8364	0.00206	0.00543	1	ENSRNO	protein_coding
Carmil3	-5.6802	-51.277	0.02196	0.04504	1	ENSRNO	protein_coding
Psme1	1.32749	2.50966	2.1E-36	4.7E-35	4E-32	ENSRNO	protein_coding
Emc9	1.68458	3.21446	2E-05	7.4E-05	0.37835	ENSRNO	protein_coding
Mdp1	1.59614	3.02333	3.8E-17	4E-16	7.3E-13	ENSRNO	protein_coding
Nfatc4	-3.9122	-15.055	1.2E-55	4.4E-54	2.3E-51	ENSRNO	protein_coding
Nynrin	-2.4121	-5.3225	4.8E-21	6.1E-20	9.1E-17	ENSRNO	protein_coding
Gzmc	-2.5285	-5.7696	4.1E-05	0.00015	0.78409	ENSRNO	protein_coding
LOC1009	3.13742	8.79951	0.00374	0.00931	1	ENSRNO	protein_coding
LOC6916	-5.7426	-53.541	0.02275	0.04651	1	ENSRNO	protein_coding
Rnf17	7.47174	177.508	0.00161	0.00433	1	ENSRNO	protein_coding
Gja3	-8.1941	-292.87	0.00072	0.00206	1	ENSRNO	protein_coding
Fgf9	7.34021	162.04	6.6E-09	3.8E-08	0.00013	ENSRNO	protein_coding
Phf11b	4.8653	29.1474	1.1E-60	4.4E-59	2.1E-56	ENSRNO	protein_coding
Phf11	3.49539	11.2776	2.6E-05	9.6E-05	0.49775	ENSRNO	protein_coding
Atp8a2	-7.4606	-176.14	5.3E-09	3E-08	0.0001	ENSRNO	protein_coding
Sgcg	-3.9423	-15.373	3E-256	1E-253	5E-252	ENSRNO	protein_coding
Pbk	-1.3368	-2.5259	1.3E-38	3E-37	2.4E-34	ENSRNO	protein_coding
Ephx2	-2.1093	-4.3148	0.00075	0.00214	1	ENSRNO	protein_coding
Chrna2	5.96256	62.3604	0.01272	0.02789	1	ENSRNO	protein_coding

Ptk2b	4.33989	20.2505	3E-267	1E-264	6E-263	ENSRNO	protein_coding
Fam167a	7.25283	152.518	6.2E-14	5.3E-13	1.2E-09	ENSRNO	protein_coding
Xkr6	-1.3782	-2.5994	8.8E-05	0.0003	1	ENSRNO	protein_coding
AABR070	2.88614	7.39292	0.01816	0.0381	1	ENSRNO	protein_coding
Scara5	-3.3973	-10.536	1E-177	2E-175	2E-173	ENSRNO	protein_coding
Adamdec	4.73725	26.6718	1.8E-24	2.7E-23	3.5E-20	ENSRNO	protein_coding
AABR070	2.45371	5.47824	0.00019	0.0006	1	ENSRNO	lincRNA
AABR070	3.52413	11.5046	2.1E-05	8E-05	0.40763	ENSRNO	lincRNA
AABR070	2.59895	6.05846	2.1E-14	1.9E-13	4.1E-10	ENSRNO	lincRNA
Adam28	2.71952	6.58651	1.5E-15	1.5E-14	2.9E-11	ENSRNO	protein_coding
Stc1	4.78888	27.6437	1.2E-07	5.8E-07	0.0022	ENSRNO	protein_coding
RGD1308	-1.5412	-2.9103	8.8E-11	6E-10	1.7E-06	ENSRNO	protein_coding
Piwil2	5.78591	55.1737	3E-301	2E-298	6E-297	ENSRNO	protein_coding
Hr	2.29386	4.90366	7.3E-14	6.3E-13	1.4E-09	ENSRNO	protein_coding
Dmtn	6.28007	77.7121	0.00828	0.01901	1	ENSRNO	protein_coding
Gfra2	4.87393	29.3223	2.4E-17	2.5E-16	4.5E-13	ENSRNO	protein_coding
Lpar6	-1.6101	-3.0527	2.2E-18	2.5E-17	4.2E-14	ENSRNO	protein_coding
Rubcnl	3.6981	12.9789	0.01056	0.0236	1	ENSRNO	protein_coding
Lcp1	2.96325	7.79881	7.7E-63	3.1E-61	1.5E-58	ENSRNO	protein_coding
Siah3	2.09526	4.27302	3.2E-05	0.00011	0.60092	ENSRNO	protein_coding
Kctd4	4.11096	17.2791	4E-09	2.4E-08	7.7E-05	ENSRNO	protein_coding
Tsc22d1	-1.758	-3.3822	2.2E-67	9.8E-66	4.1E-63	ENSRNO	protein_coding
Serp2	-2.6975	-6.4866	1.3E-11	9.6E-11	2.5E-07	ENSRNO	protein_coding
Kbtbd6_1	2.53146	5.78157	2.3E-05	8.5E-05	0.43977	ENSRNO	protein_coding
Kbtbd6_2	6.07246	67.2967	1.3E-15	1.2E-14	2.5E-11	ENSRNO	protein_coding
Pcdh20	3.24665	9.49161	1.3E-47	3.9E-46	2.5E-43	ENSRNO	protein_coding
AABR070	5.98649	63.4033	4.1E-06	1.7E-05	0.07828	ENSRNO	protein_coding
Pcdh9	-7.4553	-175.49	4.9E-09	2.8E-08	9.3E-05	ENSRNO	protein_coding
RGD1306	6.0183	64.8172	9.4E-13	7.4E-12	1.8E-08	ENSRNO	protein_coding
Dach1	2.85641	7.24213	9.8E-17	1E-15	1.9E-12	ENSRNO	protein_coding
AABR070	-6.3521	-81.693	0.00914	0.02076	1	ENSRNO	pseudogene
AABR070	4.43683	21.6581	2.4E-05	8.8E-05	0.45448	ENSRNO	lincRNA
Scel	5.34133	40.5417	4.5E-35	9.6E-34	8.5E-31	ENSRNO	protein_coding
Slitrk6	5.36813	41.3018	3.6E-10	2.3E-09	6.8E-06	ENSRNO	protein_coding
Dzip1	-1.8964	-3.7227	2.7E-68	1.2E-66	5.2E-64	ENSRNO	protein_coding
Hs6st3	6.45782	87.9018	8.3E-07	3.7E-06	0.0157	ENSRNO	processed_transcript
Clybl	2.76585	6.80148	3.6E-12	2.7E-11	6.8E-08	ENSRNO	protein_coding
Itgbl1	4.00622	16.0691	1.1E-72	5.1E-71	2E-68	ENSRNO	protein_coding
Zcchc24	-2.7575	-6.7622	1E-141	2E-139	2E-137	ENSRNO	protein_coding
Dnah12	2.6529	6.2893	1.8E-06	7.7E-06	0.03396	ENSRNO	protein_coding
Arhgef3	-6.2335	-75.243	2.4E-88	1.5E-86	4.6E-84	ENSRNO	protein_coding
Fam208a	-7.7006	-208.02	2E-179	3E-177	3E-175	ENSRNO	protein_coding
Erc2	-7.4383	-173.44	0.00218	0.0057	1	ENSRNO	protein_coding
Cacna1d	2.63214	6.19945	0.0053	0.0128	1	ENSRNO	protein_coding
Prkcd	2.15119	4.44194	2.1E-64	9.1E-63	4.1E-60	ENSRNO	protein_coding
Tnnc1	-3.6546	-12.593	4E-20	4.8E-19	7.6E-16	ENSRNO	protein_coding
Sema3g	-1.7296	-3.3164	4.7E-05	0.00017	0.89929	ENSRNO	protein_coding
Dnah1	-5.1448	-35.379	2.5E-13	2E-12	4.7E-09	ENSRNO	protein_coding
Sh3bp5	-2.2944	-4.9054	9.5E-74	4.6E-72	1.8E-69	ENSRNO	protein_coding

Colq	3.98189	15.8004	0.00016	0.0005	1	ENSRNO	protein_coding
Vstm4	6.41595	85.387	0.00705	0.0165	1	ENSRNO	protein_coding
Arhgap22	-2.4617	-5.5085	3E-119	3E-117	6E-115	ENSRNO	protein_coding
Ptpn20	6.23155	75.1424	0.00923	0.02093	1	ENSRNO	protein_coding
Anxa8	-1.803	-3.4893	9.4E-63	3.8E-61	1.8E-58	ENSRNO	protein_coding
Fam25a	6.56349	94.5816	4E-238	1E-235	8E-234	ENSRNO	protein_coding
Sncg	-4.3325	-20.146	7E-131	7E-129	1E-126	ENSRNO	protein_coding
Mmrn2	-5.5666	-47.394	3.7E-71	1.7E-69	7.1E-67	ENSRNO	protein_coding
AABR070	2.59557	6.04429	2.1E-07	1E-06	0.00398	ENSRNO	pseudogene
Mat1a	1.53241	2.8927	1.6E-12	1.3E-11	3.1E-08	ENSRNO	protein_coding
Klf2	-1.6767	-3.1969	4.9E-10	3.1E-09	9.2E-06	ENSRNO	protein_coding
Kcnn1	3.13339	8.77496	3.4E-11	2.4E-10	6.5E-07	ENSRNO	protein_coding
Pde4c	-2.6302	-6.1912	0.01965	0.04086	1	ENSRNO	protein_coding
Gdf15	6.97826	126.085	1.7E-70	7.7E-69	3.2E-66	ENSRNO	protein_coding
Ssbp4	2.55395	5.8724	1.3E-45	3.6E-44	2.4E-41	ENSRNO	protein_coding
Isyna1	1.56657	2.962	0.00734	0.01712	1	ENSRNO	protein_coding
Klhl26	-3.8155	-14.079	1E-19	1.2E-18	2E-15	ENSRNO	protein_coding
Comp	-6.5051	-90.831	7.9E-19	9E-18	1.5E-14	ENSRNO	protein_coding
Mef2b	2.3437	5.07603	5.7E-05	0.0002	1	ENSRNO	protein_coding
Ncan	2.02807	4.07858	0.01174	0.02594	1	ENSRNO	protein_coding
Hapln4	-1.7647	-3.3979	4.9E-31	9.2E-30	9.2E-27	ENSRNO	protein_coding
Tssk6	2.61038	6.10663	6.8E-09	3.9E-08	0.00013	ENSRNO	protein_coding
RGD1563	3.50099	11.3215	0.00169	0.00452	1	ENSRNO	protein_coding
Lzts1	-5.7784	-54.889	7.3E-12	5.4E-11	1.4E-07	ENSRNO	protein_coding
Lpl	-5.6071	-48.741	1.9E-05	7E-05	0.35277	ENSRNO	protein_coding
Ccdc94_1	1.59622	3.02349	0.00404	0.00999	1	ENSRNO	protein_coding
Sh2d4a	-1.4022	-2.6431	3.6E-16	3.6E-15	6.9E-12	ENSRNO	protein_coding
AABR070	7.78804	221.022	0.00102	0.00284	1	ENSRNO	pseudogene
Tll1	-4.204	-18.43	4E-12	3E-11	7.6E-08	ENSRNO	protein_coding
ENSRNO	6.18009	72.5092	0.01018	0.02286	1	ENSRNO	protein_coding
Palld_2	-1.7303	-3.3181	6.6E-60	2.6E-58	1.2E-55	ENSRNO	protein_coding
Mfap3l	3.9298	15.24	4E-110	4E-108	8E-106	ENSRNO	protein_coding
Aadat	-1.5773	-2.9841	1.6E-06	6.8E-06	0.02979	ENSRNO	protein_coding
AABR070	2.67204	6.3733	1E-06	4.6E-06	0.01973	ENSRNO	lincRNA
Asb5	-4.3411	-20.268	5E-40	1.2E-38	9.5E-36	ENSRNO	protein_coding
Vegfc	-7.4642	-176.59	2.3E-30	4.3E-29	4.4E-26	ENSRNO	protein_coding
Tenm3	-4.0611	-16.692	7E-292	4E-289	1E-287	ENSRNO	protein_coding
Stox2	5.73859	53.3936	1.6E-11	1.1E-10	3E-07	ENSRNO	protein_coding
AABR070	6.61724	98.172	0.00548	0.01319	1	ENSRNO	pseudogene
Ankrd37	-1.4944	-2.8175	1.8E-05	6.9E-05	0.34814	ENSRNO	protein_coding
LOC1009	-3.3397	-10.124	0.00198	0.00522	1	ENSRNO	protein_coding
Sorbs2	-2.1028	-4.2953	3.3E-72	1.6E-70	6.2E-68	ENSRNO	protein_coding
Mtus1	-2.1825	-4.5394	4E-101	3E-99	6.7E-97	ENSRNO	protein_coding
Pdgfrl	-3.8701	-14.623	4.7E-56	1.7E-54	8.9E-52	ENSRNO	protein_coding
AABR070	-2.8011	-6.9699	0.00486	0.01184	1	ENSRNO	protein_coding
Slc7a2	-1.5745	-2.9784	3.4E-18	3.8E-17	6.5E-14	ENSRNO	protein_coding
AABR070	3.58522	12.0021	9.7E-05	0.00033	1	ENSRNO	lincRNA
AABR070	3.70384	13.0306	0.00692	0.01623	1	ENSRNO	lincRNA
Ppp1r3b	5.0296	32.6633	1.8E-14	1.6E-13	3.3E-10	ENSRNO	protein_coding

Dusp4	3.49814	11.2991	3.9E-86	2.2E-84	7.3E-82	ENSRNO	protein_coding
Rbpms	2.26066	4.79212	7.8E-13	6.2E-12	1.5E-08	ENSRNO	protein_coding
Tex15	-3.2006	-9.1937	0.00054	0.00158	1	ENSRNO	protein_coding
Nrg1	3.55947	11.7898	8E-99	6.3E-97	1.6E-94	ENSRNO	protein_coding
Chrnb3	5.03984	32.896	0.00012	0.00041	1	ENSRNO	protein_coding
Adrb3	-9.7899	-885.21	4.6E-05	0.00016	0.86552	ENSRNO	protein_coding
Got1l1	3.90413	14.9713	4E-08	2.1E-07	0.00075	ENSRNO	protein_coding
Zfp703	-1.3345	-2.5219	1.5E-14	1.4E-13	2.9E-10	ENSRNO	protein_coding
Fgfr1	6.29941	78.7609	5E-249	2E-246	1E-244	ENSRNO	protein_coding
Htra4	1.34624	2.54249	0.00074	0.00213	1	ENSRNO	protein_coding
Adam32	6.13975	70.5097	5.2E-42	1.4E-40	9.8E-38	ENSRNO	protein_coding
LOC1009	4.69954	25.9837	4.5E-20	5.5E-19	8.6E-16	ENSRNO	protein_coding
Ank1	4.61342	24.4781	3.6E-07	1.7E-06	0.00676	ENSRNO	protein_coding
LOC1009	1.57894	2.9875	3.7E-05	0.00013	0.70567	ENSRNO	protein_coding
Ap3m2	6.05384	66.4337	3.4E-19	3.9E-18	6.4E-15	ENSRNO	protein_coding
Plat	2.20411	4.60791	5.9E-96	4.2E-94	1.1E-91	ENSRNO	protein_coding
Thsd1	1.70036	3.24982	2.5E-05	9.2E-05	0.47788	ENSRNO	protein_coding
Defb37	5.39839	42.1771	3E-10	2E-09	5.8E-06	ENSRNO	protein_coding
Csmd1	3.78044	13.7413	0.00542	0.01306	1	ENSRNO	protein_coding
Myom2	4.93437	30.5769	9.8E-59	3.7E-57	1.9E-54	ENSRNO	protein_coding
Kbtbd11	1.49585	2.8203	1.4E-06	6E-06	0.02625	ENSRNO	protein_coding
Dlgap2	1.71328	3.27905	0.00478	0.01166	1	ENSRNO	protein_coding
Tmco3	2.87912	7.35702	7E-10	4.4E-09	1.3E-05	ENSRNO	protein_coding
Gas6	-5.3891	-41.906	3E-86	1.8E-84	5.8E-82	ENSRNO	protein_coding
Adprhl1	2.47964	5.57758	1.3E-18	1.5E-17	2.5E-14	ENSRNO	protein_coding
Grtp1_1	2.13983	4.4071	4.3E-23	6E-22	8.2E-19	ENSRNO	protein_coding
Rab20	2.42431	5.36774	6.6E-41	1.7E-39	1.3E-36	ENSRNO	protein_coding
Col4a2	-3.4938	-11.265	6E-226	2E-223	1E-221	ENSRNO	protein_coding
Col4a1	-3.4052	-10.594	2E-222	4E-220	3E-218	ENSRNO	protein_coding
Myo16	5.46778	44.2553	3E-05	0.00011	0.58	ENSRNO	protein_coding
Fbp2	9.33466	645.673	7E-05	0.00024	1	ENSRNO	protein_coding
Aaed1	-1.5557	-2.9397	4.1E-07	1.9E-06	0.00785	ENSRNO	protein_coding
Cntnap3b	5.69971	51.9739	0.01857	0.03887	1	ENSRNO	protein_coding
Dapk1	5.96249	62.3575	0.013	0.02841	1	ENSRNO	protein_coding
Spata31d	1.72699	3.31036	0.00702	0.01644	1	ENSRNO	protein_coding
Golm1	3.4552	10.9678	7.8E-11	5.3E-10	1.5E-06	ENSRNO	protein_coding
AABR070	2.66191	6.32871	0.01634	0.03471	1	ENSRNO	lincRNA
Ntrk2	2.44336	5.43906	0.00122	0.00335	1	ENSRNO	protein_coding
Slc28a3	3.87354	14.6573	1.1E-09	6.7E-09	2.1E-05	ENSRNO	protein_coding
LOC1025	2.33325	5.03941	1.2E-37	2.8E-36	2.3E-33	ENSRNO	lincRNA
Spock1	4.72864	26.5133	1.8E-07	8.7E-07	0.00338	ENSRNO	protein_coding
Trpc7	-1.7341	-3.3267	0.01286	0.02817	1	ENSRNO	protein_coding
AABR070	-2.0226	-4.063	0.00653	0.01541	1	ENSRNO	lincRNA
Slc25a48	-2.4993	-5.6542	3.4E-42	9.2E-41	6.6E-38	ENSRNO	protein_coding
Pitx1	6.01625	64.7251	0.01252	0.0275	1	ENSRNO	protein_coding
Dbn1	-3.3086	-9.9078	4.4E-92	2.9E-90	8.4E-88	ENSRNO	protein_coding
Prr7	-2.9978	-7.9878	2.2E-13	1.8E-12	4.2E-09	ENSRNO	protein_coding
Hk3	3.25295	9.5331	3.3E-23	4.6E-22	6.2E-19	ENSRNO	protein_coding
Tspan17	-4.7975	-27.81	2E-199	3E-197	3E-195	ENSRNO	protein_coding

Cdhr2	1.9123	3.76409	0.01531	0.03281	1	ENSRNO	protein_coding
Drd1	7.6034	194.47	2.1E-09	1.3E-08	4.1E-05	ENSRNO	protein_coding
AABR070	3.13499	8.78466	8.2E-06	3.2E-05	0.15516	ENSRNO	lincRNA
Ror2	-3.3414	-10.136	1.9E-66	8.4E-65	3.6E-62	ENSRNO	protein_coding
Syk	3.44325	10.8773	2E-11	1.5E-10	3.9E-07	ENSRNO	protein_coding
Diras2	4.49986	22.6252	9.5E-64	3.9E-62	1.8E-59	ENSRNO	protein_coding
Gadd45g	-1.665	-3.1712	7.1E-41	1.8E-39	1.3E-36	ENSRNO	protein_coding
Sema4d	2.33004	5.0282	0.0229	0.04678	1	ENSRNO	protein_coding
S1pr3	-2.5265	-5.7617	6.5E-10	4.1E-09	1.2E-05	ENSRNO	protein_coding
LOC1036	-8.7453	-429.14	0.00051	0.00152	1	ENSRNO	protein_coding
NEWGEN	-2.7408	-6.6845	2.8E-61	1.1E-59	5.3E-57	ENSRNO	protein_coding
Ogn	-2.8396	-7.1582	5.2E-44	1.4E-42	9.8E-40	ENSRNO	protein_coding
Omd	-1.5441	-2.9163	1.5E-05	5.6E-05	0.27931	ENSRNO	protein_coding
Fgd3	5.19809	36.7097	6.9E-64	2.9E-62	1.3E-59	ENSRNO	protein_coding
Id4	3.59015	12.0433	2.1E-19	2.4E-18	4E-15	ENSRNO	protein_coding
Nhlrc1	1.79691	3.47475	5.3E-07	2.4E-06	0.00999	ENSRNO	protein_coding
Cap2	4.97618	31.476	0.00016	0.00052	1	ENSRNO	protein_coding
Rbm24	6.07626	67.4741	0.01091	0.02428	1	ENSRNO	protein_coding
Edn1	-4.6924	-25.855	6.8E-27	1.1E-25	1.3E-22	ENSRNO	protein_coding
Phactr1	3.18489	9.09385	1.2E-21	1.6E-20	2.3E-17	ENSRNO	protein_coding
Rnf182	3.97853	15.7636	3.1E-11	2.2E-10	5.9E-07	ENSRNO	protein_coding
Cd83	3.62324	12.3226	0.00852	0.01951	1	ENSRNO	protein_coding
Tfap2a	2.54596	5.83998	4E-48	1.2E-46	7.6E-44	ENSRNO	protein_coding
F13a1	2.03184	4.08927	2.4E-33	5E-32	4.6E-29	ENSRNO	protein_coding
Fam217a	1.56747	2.96384	0.0106	0.02368	1	ENSRNO	protein_coding
Slc22a23	2.07468	4.21252	5.5E-59	2.1E-57	1E-54	ENSRNO	protein_coding
Tubb2a	-6.8087	-112.11	4E-142	5E-140	7E-138	ENSRNO	protein_coding
Serpinb6b	3.16718	8.98291	4.6E-13	3.7E-12	8.8E-09	ENSRNO	protein_coding
Serpinb1a	1.762	3.39167	2.7E-20	3.3E-19	5.2E-16	ENSRNO	protein_coding
Foxq1	1.41146	2.66006	4.6E-16	4.5E-15	8.8E-12	ENSRNO	pseudogene
Dusp22	7.06336	133.747	4.6E-13	3.7E-12	8.8E-09	ENSRNO	protein_coding
Prl5a1	4.80395	27.934	0.00027	0.00085	1	ENSRNO	protein_coding
Prl7a3	7.06627	134.017	0.00286	0.0073	1	ENSRNO	protein_coding
Dcdc2	-4.4163	-21.352	3.9E-05	0.00014	0.74962	ENSRNO	protein_coding
Prl6a1	5.97634	62.9589	1E-48	3.2E-47	2E-44	ENSRNO	protein_coding
Carmil1	-1.4979	-2.8242	5.6E-46	1.6E-44	1.1E-41	ENSRNO	protein_coding
Slc17a4	1.33468	2.52219	0.01155	0.02555	1	ENSRNO	protein_coding
Hist1h1a	3.24552	9.48415	0.0183	0.03838	1	ENSRNO	protein_coding
Hist2h3c2	-1.514	-2.856	0.0069	0.01618	1	ENSRNO	protein_coding
Hfe	2.72464	6.60993	6.7E-31	1.3E-29	1.3E-26	ENSRNO	protein_coding
Hist1h1d	-1.4724	-2.7749	0.00556	0.01338	1	ENSRNO	protein_coding
Btn2a2	-1.829	-3.5528	2.4E-58	9.1E-57	4.6E-54	ENSRNO	protein_coding
hist1h2ail	2.06596	4.18713	0.01546	0.03309	1	ENSRNO	protein_coding
RGD1562	6.02226	64.9953	1.4E-73	6.8E-72	2.7E-69	ENSRNO	protein_coding
Hist1h2bk	1.82703	3.54805	0.0002	0.00064	1	ENSRNO	protein_coding
LOC6803	-5.5948	-48.33	0.02258	0.04623	1	ENSRNO	protein_coding
RGD1562	-2.3237	-5.0061	0.00559	0.01344	1	ENSRNO	protein_coding
Hist1h3b_	3.49439	11.2698	0.01981	0.04115	1	ENSRNO	protein_coding
Gpr141	6.22929	75.0248	0.00928	0.02104	1	ENSRNO	protein_coding

LOC4987	3.42176	10.7165	0.0212	0.04368	1	ENSRNO	protein_coding
LOC1009	-1.3979	-2.6352	7.2E-43	1.9E-41	1.4E-38	ENSRNO	protein_coding
ENSRNO	-2.1935	-4.5742	2.7E-08	1.4E-07	0.00051	ENSRNO	protein_coding
Svil	-1.5653	-2.9593	1.1E-52	3.7E-51	2.1E-48	ENSRNO	protein_coding
RGD1562	-4.0721	-16.819	1.4E-81	7.7E-80	2.7E-77	ENSRNO	protein_coding
Mpp7	-1.8258	-3.545	1.5E-09	9.2E-09	2.9E-05	ENSRNO	protein_coding
Fzd8	-1.3348	-2.5225	4.6E-11	3.2E-10	8.8E-07	ENSRNO	protein_coding
Chrm3	-8.8109	-449.1	3.6E-12	2.7E-11	6.9E-08	ENSRNO	protein_coding
ENSRNO	2.88911	7.40813	4.2E-06	1.7E-05	0.07971	ENSRNO	protein_coding
AABR070	5.12923	34.9986	0.00011	0.00037	1	ENSRNO	pseudogene
Akr1c13	3.82731	14.195	2.6E-16	2.6E-15	4.9E-12	ENSRNO	protein_coding
Akr1c19	2.16269	4.4775	2E-08	1.1E-07	0.00037	ENSRNO	protein_coding
Akr1c3	-3.3297	-10.054	0.00267	0.00687	1	ENSRNO	protein_coding
Akr1c14	-2.4595	-5.5002	5E-33	1E-31	9.5E-29	ENSRNO	protein_coding
Il15ra	2.72831	6.62678	5.3E-11	3.7E-10	1E-06	ENSRNO	protein_coding
Prkcq	2.43656	5.41351	2.3E-06	9.8E-06	0.04385	ENSRNO	protein_coding
Gata3	1.37019	2.58504	1.1E-15	1.1E-14	2.2E-11	ENSRNO	protein_coding
Echdc3	-1.7674	-3.4045	5.8E-17	5.9E-16	1.1E-12	ENSRNO	protein_coding
Frmd4a	-2.0772	-4.22	2E-34	4.2E-33	3.8E-30	ENSRNO	protein_coding
Fam171a	1.32227	2.50059	2.4E-14	2.1E-13	4.5E-10	ENSRNO	protein_coding
Itga8	1.85248	3.6112	4.5E-42	1.2E-40	8.5E-38	ENSRNO	protein_coding
Cubn	-2.0757	-4.2155	3.6E-10	2.3E-09	6.8E-06	ENSRNO	protein_coding
Hacd1	-1.4923	-2.8133	2.6E-32	5.1E-31	4.9E-28	ENSRNO	protein_coding
Plxdc2	-5.1984	-36.717	0	0	0	ENSRNO	protein_coding
Nebl	2.89494	7.43814	4.5E-05	0.00016	0.85302	ENSRNO	protein_coding
Msrb2	-7.5918	-192.91	0.00173	0.00462	1	ENSRNO	protein_coding
Otud1	-1.7749	-3.4221	3.9E-16	3.9E-15	7.5E-12	ENSRNO	protein_coding
Myo3a	6.02038	64.9107	0.01192	0.0263	1	ENSRNO	protein_coding
Nid1	-2.846	-7.1898	9.5E-18	1E-16	1.8E-13	ENSRNO	protein_coding
Gpr137b	-2.2328	-4.7004	2.8E-43	7.4E-42	5.2E-39	ENSRNO	protein_coding
Ero1b	-1.6075	-3.0472	5.9E-21	7.4E-20	1.1E-16	ENSRNO	protein_coding
Mtcp1	1.47355	2.77705	0.00234	0.00609	1	ENSRNO	protein_coding
Zfp136	-1.4879	-2.8047	0.00025	0.00079	1	ENSRNO	protein_coding
Gata6	1.51458	2.85715	5.8E-11	4E-10	1.1E-06	ENSRNO	protein_coding
Kctd1	1.93247	3.81707	1.3E-25	2E-24	2.4E-21	ENSRNO	protein_coding
Cdh2	6.43667	86.6223	1E-156	2E-154	3E-152	ENSRNO	protein_coding
Klhl14	2.83542	7.13752	0.0133	0.02902	1	ENSRNO	protein_coding
Dtna	6.36412	82.3741	4.2E-43	1.1E-41	8.1E-39	ENSRNO	protein_coding
Rnf125	1.43987	2.71296	9.9E-05	0.00033	1	ENSRNO	protein_coding
Dsg2	7.02392	130.14	0	0	0	ENSRNO	protein_coding
Celf4	6.37207	82.8291	0.00764	0.01773	1	ENSRNO	protein_coding
Myo7b	1.41708	2.67044	2E-05	7.5E-05	0.38348	ENSRNO	protein_coding
Epb41l4a	-3.6089	-12.201	3.1E-77	1.6E-75	5.9E-73	ENSRNO	protein_coding
Nme5	-2.2383	-4.7183	0.00083	0.00236	1	ENSRNO	protein_coding
Egr1	-1.7765	-3.4259	1.2E-21	1.6E-20	2.3E-17	ENSRNO	protein_coding
AC126530	1.5656	2.96	0.0091	0.02069	1	ENSRNO	pseudogene
Slc23a1	-1.9209	-3.7866	0.00969	0.02186	1	ENSRNO	protein_coding
Pcdhb5	3.02846	8.15937	3.4E-13	2.8E-12	6.5E-09	ENSRNO	protein_coding
Pcdhb10	-3.3187	-9.9775	0.01879	0.03929	1	ENSRNO	protein_coding

Pcdhb12	1.46546	2.76151	2.8E-06	1.2E-05	0.0531	ENSRNO	protein_coding
LOC1083	2.81539	7.03908	7.4E-09	4.2E-08	0.00014	ENSRNO	protein_coding
LOC1083	2.09525	4.27301	1.4E-29	2.6E-28	2.7E-25	ENSRNO	protein_coding
AC13136(	1.6836	3.21229	2.2E-17	2.3E-16	4.1E-13	ENSRNO	pseudogene
LOC1083	2.03402	4.09544	4.7E-12	3.5E-11	8.9E-08	ENSRNO	protein_coding
Pcdhb19	1.67464	3.19241	4.7E-06	1.9E-05	0.0886	ENSRNO	protein_coding
Pcdhb21	1.47982	2.78914	5.1E-07	2.4E-06	0.00971	ENSRNO	protein_coding
Pcdhb22	1.33372	2.52051	3.1E-05	0.00011	0.58489	ENSRNO	protein_coding
Slc25a2	1.76489	3.39847	1.3E-06	5.8E-06	0.02508	ENSRNO	protein_coding
Taf7	1.32339	2.50254	2.3E-31	4.5E-30	4.5E-27	ENSRNO	protein_coding
RGD1563	9.49188	720.014	5.3E-05	0.00018	1	ENSRNO	protein_coding
Pcdhga1	4.14051	17.6367	1.7E-51	5.4E-50	3.2E-47	ENSRNO	protein_coding
Pcdhga2	4.2281	18.7407	1.4E-25	2.1E-24	2.6E-21	ENSRNO	protein_coding
Pcdhga3	4.87046	29.2519	6.5E-18	7.1E-17	1.2E-13	ENSRNO	protein_coding
ENSRNO	3.31205	9.93176	4.1E-14	3.6E-13	7.8E-10	ENSRNO	protein_coding
Pcdhga4	2.61033	6.10643	5.1E-05	0.00018	0.96725	ENSRNO	protein_coding
AABR070	3.16357	8.96042	4.1E-48	1.2E-46	7.7E-44	ENSRNO	pseudogene
Pcdhga5	4.42251	21.4442	7.8E-08	4E-07	0.00148	ENSRNO	protein_coding
Pcdhga6	2.44128	5.43123	3.2E-05	0.00012	0.61382	ENSRNO	protein_coding
Pcdhgb4	3.58989	12.0411	2.3E-05	8.6E-05	0.44224	ENSRNO	protein_coding
Pcdhga8	4.09421	17.0797	1.4E-05	5.4E-05	0.26864	ENSRNO	protein_coding
Pcdhgb5	4.5332	23.1542	1E-06	4.5E-06	0.01936	ENSRNO	protein_coding
Pcdhga9	3.35558	10.236	2.8E-07	1.3E-06	0.00536	ENSRNO	protein_coding
Pcdhgb6	2.59281	6.03271	1.7E-06	7.3E-06	0.03211	ENSRNO	protein_coding
Pcdhga1(	4.44194	21.7348	2.8E-11	2E-10	5.3E-07	ENSRNO	protein_coding
Pcdhgb7	5.32707	40.1428	6.5E-10	4.1E-09	1.2E-05	ENSRNO	protein_coding
Pcdhga11	3.88718	14.7965	1E-09	6.3E-09	1.9E-05	ENSRNO	protein_coding
Rell2	-2.083	-4.237	9E-35	1.9E-33	1.7E-30	ENSRNO	protein_coding
Arhgap26	-2.5921	-6.0297	1.5E-10	1E-09	2.9E-06	ENSRNO	protein_coding
LOC1036	-8.2531	-305.1	0.00166	0.00443	1	ENSRNO	protein_coding
Dpysl3	-1.6412	-3.1193	2.2E-59	8.6E-58	4.3E-55	ENSRNO	protein_coding
Prr16	6.30363	78.9915	1.2E-06	5.4E-06	0.02322	ENSRNO	protein_coding
Lox	-3.5099	-11.392	5E-267	2E-264	9E-263	ENSRNO	protein_coding
Zfp474	-4.2223	-18.665	4E-13	3.3E-12	7.6E-09	ENSRNO	protein_coding
Prdm6	-3.8054	-13.981	8.8E-08	4.5E-07	0.00168	ENSRNO	protein_coding
Megf10	-5.3936	-42.038	2E-42	5.2E-41	3.7E-38	ENSRNO	protein_coding
RGD1312	7.18141	145.151	1.5E-13	1.2E-12	2.8E-09	ENSRNO	protein_coding
LOC1009	-6.5371	-92.867	0.00822	0.01889	1	ENSRNO	protein_coding
LOC1009	1.53246	2.89278	1.8E-09	1.1E-08	3.5E-05	ENSRNO	protein_coding
MGC1055	2.93459	7.64537	6.7E-16	6.4E-15	1.3E-11	ENSRNO	protein_coding
Cd74	3.97799	15.7578	1.8E-08	9.6E-08	0.00034	ENSRNO	protein_coding
Arsi	-2.9035	-7.4826	0.00844	0.01933	1	ENSRNO	protein_coding
Slc6a7	-1.9639	-3.9012	0.01548	0.03311	1	ENSRNO	protein_coding
Pdgfrb	-1.7128	-3.278	9.9E-62	3.9E-60	1.9E-57	ENSRNO	protein_coding
Slc26a2	1.78225	3.43963	0.0202	0.0418	1	ENSRNO	protein_coding
Arhgef37	-3.4226	-10.723	0.01313	0.02868	1	ENSRNO	protein_coding
Afap1l1	3.69281	12.9314	9.3E-07	4.2E-06	0.01776	ENSRNO	protein_coding
Ablim3	-6.0226	-65.013	0.01361	0.02962	1	ENSRNO	protein_coding
Sh3tc2	1.59972	3.03085	3.3E-18	3.7E-17	6.4E-14	ENSRNO	protein_coding

Piezo2	-3.6846	-12.858	2.2E-12	1.7E-11	4.2E-08	ENSRNO	protein_coding
Fech	-1.7229	-3.3009	1.6E-54	5.5E-53	3.1E-50	ENSRNO	protein_coding
Alpk2	2.86409	7.28077	3E-07	1.4E-06	0.00566	ENSRNO	protein_coding
Oacyl	3.68026	12.8194	0.00083	0.00237	1	ENSRNO	protein_coding
Ccbe1	1.32346	2.50266	1.5E-14	1.3E-13	2.8E-10	ENSRNO	protein_coding
Cidea	-3.4618	-11.018	5.3E-05	0.00019	1	ENSRNO	protein_coding
Rab27b	9.79426	887.902	3E-05	0.00011	0.5761	ENSRNO	protein_coding
Stard6	-1.4164	-2.6692	4.5E-07	2.1E-06	0.00861	ENSRNO	protein_coding
Lipg	3.07861	8.448	5.8E-07	2.7E-06	0.01101	ENSRNO	protein_coding
Ctif	-1.5408	-2.9096	3.7E-49	1.1E-47	7E-45	ENSRNO	protein_coding
Zbtb7c	1.52047	2.86884	1.3E-14	1.2E-13	2.5E-10	ENSRNO	protein_coding
Katnal2	1.90584	3.74728	0.0153	0.0328	1	ENSRNO	protein_coding
Loxhd1	6.02038	64.9107	0.01192	0.0263	1	ENSRNO	protein_coding
Pstpip2	2.26608	4.81015	2.7E-07	1.3E-06	0.0051	ENSRNO	protein_coding
Pard6g	-2.9556	-7.7574	3.3E-11	2.3E-10	6.3E-07	ENSRNO	protein_coding
Pqlc1	1.43397	2.70189	5.9E-18	6.4E-17	1.1E-13	ENSRNO	protein_coding
Nfatc1	1.4753	2.78041	2.9E-12	2.2E-11	5.5E-08	ENSRNO	protein_coding
Mbp	1.54389	2.9158	2.6E-14	2.3E-13	4.9E-10	ENSRNO	protein_coding
Cndp1	-2.5231	-5.748	2.2E-16	2.2E-15	4.1E-12	ENSRNO	protein_coding
Fam69c	-1.8069	-3.4989	3.8E-07	1.8E-06	0.00717	ENSRNO	protein_coding
Cbln2	5.88897	59.2595	5.2E-24	7.5E-23	9.9E-20	ENSRNO	protein_coding
LOC1083	6.23163	75.1461	0.00904	0.02055	1	ENSRNO	protein_coding
AABR070	5.48649	44.833	9.9E-60	3.8E-58	1.9E-55	ENSRNO	protein_coding
Rrad	-2.3328	-5.0377	0.00048	0.00142	1	ENSRNO	protein_coding
Cdh16	1.62418	3.08267	0.01552	0.03319	1	ENSRNO	protein_coding
Terb1	2.07844	4.2235	0.00306	0.00779	1	ENSRNO	protein_coding
Cklf	-1.7238	-3.3031	0.00304	0.00775	1	ENSRNO	protein_coding
Cdh5	6.35373	81.7831	9.6E-07	4.3E-06	0.0183	ENSRNO	protein_coding
Cdh11	-7.0191	-129.71	4E-224	1E-221	8E-220	ENSRNO	protein_coding
AABR070	4.28002	19.4273	0.00114	0.00316	1	ENSRNO	protein_coding
AABR070	6.453	87.6083	0.00756	0.01757	1	ENSRNO	lincRNA
Ndrg4	-1.6256	-3.0856	1.9E-36	4.3E-35	3.7E-32	ENSRNO	protein_coding
Mmp15	-4.4401	-21.707	8.6E-18	9.2E-17	1.6E-13	ENSRNO	protein_coding
Tepp	-2.0219	-4.0612	0.00854	0.01955	1	ENSRNO	protein_coding
Adgrg1	2.75287	6.74058	0.01286	0.02817	1	ENSRNO	protein_coding
Cx3cl1	1.86085	3.63221	3E-09	1.8E-08	5.8E-05	ENSRNO	protein_coding
Mt2A	6.71261	104.881	1E-100	8E-99	1.9E-96	ENSRNO	protein_coding
Ces5a	-2.1678	-4.4934	5.7E-12	4.2E-11	1.1E-07	ENSRNO	protein_coding
Large1	-1.3424	-2.5358	4.8E-40	1.2E-38	9.2E-36	ENSRNO	protein_coding
Zfp82_2	-8.3129	-318	0.00059	0.00171	1	ENSRNO	protein_coding
Ces1d	-3.4096	-10.627	0.01875	0.03921	1	ENSRNO	protein_coding
Slc6a2	4.83652	28.5718	2.2E-06	9.5E-06	0.04253	ENSRNO	protein_coding
Lpcat2	-6.3478	-81.446	2.8E-74	1.3E-72	5.2E-70	ENSRNO	protein_coding
Mmp2	-1.944	-3.8477	0.0007	0.00202	1	ENSRNO	protein_coding
Irx5	-3.5388	-11.622	1E-21	1.4E-20	2E-17	ENSRNO	protein_coding
Snx20	5.59197	48.2337	1.7E-13	1.4E-12	3.2E-09	ENSRNO	protein_coding
Adcy7	-3.3839	-10.439	3E-121	3E-119	5E-117	ENSRNO	protein_coding
Zfp423	4.76743	27.2358	8.3E-06	3.3E-05	0.15833	ENSRNO	protein_coding
Neto2	8.75532	432.129	1.1E-41	2.9E-40	2.1E-37	ENSRNO	protein_coding

Sall1	6.23176	75.1529	0.00888	0.02024	1	ENSRNO	protein_coding
Rnf150	-2.3803	-5.2064	1.3E-68	6.1E-67	2.5E-64	ENSRNO	protein_coding
Tbc1d9	-2.2561	-4.7771	7E-99	5E-97	1.2E-94	ENSRNO	protein_coding
Adgrl1	5.22461	37.3909	9E-242	3E-239	2E-237	ENSRNO	protein_coding
Palm3	2.69599	6.47997	3.7E-16	3.6E-15	7E-12	ENSRNO	protein_coding
Il27ra	2.31027	4.95977	5.8E-11	4E-10	1.1E-06	ENSRNO	protein_coding
Podnl1	-5.2208	-37.292	2.1E-63	8.7E-62	4.1E-59	ENSRNO	protein_coding
Cacna1a	-1.9011	-3.7349	2.2E-05	8.2E-05	0.4213	ENSRNO	protein_coding
AABR070	6.40088	84.4999	3E-06	1.2E-05	0.05623	ENSRNO	lincRNA
AABR070	9.54534	747.196	4.8E-05	0.00017	0.9195	ENSRNO	protein_coding
AABR070	5.55466	47.0023	0.02089	0.04311	1	ENSRNO	protein_coding
AABR070	2.70185	6.50633	5.8E-08	3E-07	0.00109	ENSRNO	lincRNA
AABR070	5.0739	33.682	5.8E-10	3.6E-09	1.1E-05	ENSRNO	protein_coding
Mylk3	2.56083	5.90047	0.00088	0.0025	1	ENSRNO	protein_coding
AABR070	2.76256	6.786	1.2E-10	7.8E-10	2.2E-06	ENSRNO	lincRNA
AABR070	5.30563	39.5507	8.3E-93	5.7E-91	1.6E-88	ENSRNO	lincRNA
LOC3633	5.19898	36.7323	2.5E-90	1.6E-88	4.8E-86	ENSRNO	protein_coding
AABR070	5.13217	35.0701	3.4E-57	1.2E-55	6.4E-53	ENSRNO	lincRNA
AABR070	4.24419	18.9509	1.1E-13	9.7E-13	2.2E-09	ENSRNO	protein_coding
LOC5014	-2.5654	-5.9194	9.5E-13	7.5E-12	1.8E-08	ENSRNO	protein_coding
AABR070	3.30944	9.91383	0.0006	0.00175	1	ENSRNO	lincRNA
AABR070	2.69803	6.48916	0.00497	0.01207	1	ENSRNO	lincRNA
AABR070	6.54311	93.2553	6.9E-07	3.1E-06	0.01304	ENSRNO	protein_coding
AABR070	3.62926	12.3742	3.3E-22	4.4E-21	6.3E-18	ENSRNO	lincRNA
AABR070	9.53255	740.601	5.5E-05	0.00019	1	ENSRNO	protein_coding
AABR070	5.55354	46.9659	0.02148	0.04419	1	ENSRNO	lincRNA
ENSRNO	4.85384	28.9169	6.7E-26	1.1E-24	1.3E-21	ENSRNO	protein_coding
Inpp4b	1.6564	3.1523	2.8E-06	1.2E-05	0.0541	ENSRNO	protein_coding
Frem3	7.46713	176.942	3.3E-09	1.9E-08	6.2E-05	ENSRNO	protein_coding
Hhip	7.90021	238.892	0.00086	0.00243	1	ENSRNO	protein_coding
Ednra	2.62096	6.15161	5.2E-75	2.6E-73	9.9E-71	ENSRNO	protein_coding
Nr3c2	-1.7538	-3.3725	1.5E-20	1.9E-19	2.9E-16	ENSRNO	protein_coding
AABR070	6.09869	68.5312	0.01774	0.03732	1	ENSRNO	protein_coding
AABR070	5.69233	51.7083	0.01885	0.03941	1	ENSRNO	lincRNA
AABR070	-1.916	-3.7738	0.02335	0.04755	1	ENSRNO	protein_coding
Ces2g	6.32953	80.4227	1E-06	4.5E-06	0.0191	ENSRNO	protein_coding
B3gnt9	1.4672	2.76485	9.6E-10	6E-09	1.8E-05	ENSRNO	protein_coding
Plekhg4	-3.2548	-9.5454	5E-192	1E-189	9E-188	ENSRNO	protein_coding
Kctd19	-2.9097	-7.5144	1.4E-66	6.2E-65	2.7E-62	ENSRNO	protein_coding
Zdhhc1	-1.3446	-2.5395	2.9E-11	2.1E-10	5.6E-07	ENSRNO	protein_coding
Rn60_19_	1.59701	3.02516	7.8E-05	0.00027	1	ENSRNO	antisense_RNA
Dpep3	3.66462	12.6812	7.7E-08	3.9E-07	0.00146	ENSRNO	protein_coding
Dpep2	2.5303	5.77694	1.4E-31	2.8E-30	2.7E-27	ENSRNO	protein_coding
Esrp2	-5.2308	-37.553	4.9E-30	8.9E-29	9.3E-26	ENSRNO	protein_coding
Mlkl	2.21023	4.62748	2.3E-49	7E-48	4.3E-45	ENSRNO	protein_coding
AABR070	1.86143	3.63367	6.5E-10	4.1E-09	1.2E-05	ENSRNO	lincRNA
Fa2h	2.27382	4.836	9E-06	3.5E-05	0.17107	ENSRNO	protein_coding
Ctrb1	2.43896	5.4225	6.6E-13	5.3E-12	1.3E-08	ENSRNO	protein_coding
Chst5	-10.294	-1255.1	1.8E-05	6.6E-05	0.33425	ENSRNO	protein_coding

Tmem231	-2.7932	-6.9318	6.9E-83	3.8E-81	1.3E-78	ENSRNO	protein_coding
Nudt7	-5.9008	-59.748	5.5E-21	7E-20	1E-16	ENSRNO	protein_coding
Pkd1l2	-2.0045	-4.0124	2.9E-07	1.4E-06	0.00556	ENSRNO	protein_coding
Cdh13	1.89878	3.72899	1.9E-74	9.6E-73	3.7E-70	ENSRNO	protein_coding
Osgin1	1.72752	3.31159	2.7E-16	2.7E-15	5.1E-12	ENSRNO	protein_coding
Atp2c2	3.46075	11.0101	2.8E-22	3.8E-21	5.4E-18	ENSRNO	protein_coding
AABR070	-1.789	-3.4557	0.01555	0.03326	1	ENSRNO	lincRNA
Fendrr	-3.8578	-14.499	3.2E-07	1.5E-06	0.006	ENSRNO	lincRNA
Foxf1	-2.3322	-5.0356	1.4E-29	2.6E-28	2.7E-25	ENSRNO	protein_coding
Foxc2	-4.514	-22.848	1.2E-56	4.4E-55	2.3E-52	ENSRNO	protein_coding
Foxl1	-2.9842	-7.9129	0.00903	0.02055	1	ENSRNO	protein_coding
AABR070	2.5601	5.89748	0.00013	0.00041	1	ENSRNO	lincRNA
Mlnr	2.30402	4.93831	0.00123	0.00337	1	ENSRNO	protein_coding
Dpep1	-3.5389	-11.623	0.00016	0.00052	1	ENSRNO	protein_coding
Spire2	-5.3922	-41.997	4.5E-05	0.00016	0.84695	ENSRNO	protein_coding
Acta1	-2.4201	-5.3519	2E-08	1.1E-07	0.00038	ENSRNO	protein_coding
Agt	1.54047	2.90888	0.00446	0.01096	1	ENSRNO	protein_coding
Sipa1l2	1.68099	3.20649	7.4E-26	1.2E-24	1.4E-21	ENSRNO	protein_coding
Pcnx2	-3.8515	-14.435	1.2E-07	5.8E-07	0.0022	ENSRNO	protein_coding
Kcnk1	-6.1726	-72.135	2.8E-13	2.3E-12	5.4E-09	ENSRNO	protein_coding
Rhobtb3	-1.772	-3.4152	1.4E-62	5.4E-61	2.6E-58	ENSRNO	protein_coding
Gpr150	-6.2504	-76.13	0.01071	0.02389	1	ENSRNO	protein_coding
Fam81b	3.13333	8.7746	0.02297	0.04687	1	ENSRNO	protein_coding
Adgrv1	3.06842	8.38853	0.00499	0.01211	1	ENSRNO	protein_coding
Edil3	-5.5522	-46.921	1.1E-24	1.6E-23	2.1E-20	ENSRNO	protein_coding
Atp6ap1l	-2.317	-4.9828	4.9E-11	3.4E-10	9.3E-07	ENSRNO	protein_coding
Pde8b	-2.1771	-4.5223	0.01799	0.03778	1	ENSRNO	protein_coding
F2rl1	1.60845	3.04923	2.4E-51	7.7E-50	4.6E-47	ENSRNO	protein_coding
Iqgap2	4.76401	27.1713	1.4E-14	1.3E-13	2.7E-10	ENSRNO	protein_coding
F2rl2	-3.9679	-15.648	1.4E-41	3.7E-40	2.7E-37	ENSRNO	protein_coding
Ndufs6	2.65554	6.3008	0.01692	0.0358	1	ENSRNO	processed_pseudogene
Sv2c	6.32674	80.2674	0.00787	0.01818	1	ENSRNO	protein_coding
Arhgef26	-5.6002	-48.509	2.6E-55	9.3E-54	5E-51	ENSRNO	protein_coding
Gcnt4	-2.291	-4.8939	2.7E-15	2.5E-14	5.2E-11	ENSRNO	protein_coding
Naip6	-2.8266	-7.0938	4.1E-12	3.1E-11	7.8E-08	ENSRNO	protein_coding
Ccdc125	-5.2243	-37.382	9.6E-57	3.5E-55	1.8E-52	ENSRNO	protein_coding
Mast4	-9.1831	-581.27	0.00016	0.00052	1	ENSRNO	protein_coding
Adamts6	-1.7137	-3.28	2.2E-28	3.9E-27	4.3E-24	ENSRNO	protein_coding
LOC1009	2.09226	4.26416	0.0143	0.03095	1	ENSRNO	protein_coding
Olr1280	2.33298	5.03844	0.01577	0.03366	1	ENSRNO	protein_coding
LOC1083	5.72538	52.9067	1.1E-11	8E-11	2.1E-07	ENSRNO	protein_coding
AABR070	-6.3522	-81.695	0.00921	0.0209	1	ENSRNO	lincRNA
Elovl7	-8.1946	-292.98	0.00071	0.00205	1	ENSRNO	protein_coding
AABR070	-1.3334	-2.52	0.01291	0.02826	1	ENSRNO	protein_coding
LOC1083	-3.0507	-8.2861	0.00115	0.00318	1	ENSRNO	lincRNA
Plk2	-2.3664	-5.1566	3E-110	2E-108	5E-106	ENSRNO	protein_coding
Ccno	-3.9343	-15.287	2.2E-05	8E-05	0.40992	ENSRNO	protein_coding
Fst	-2.2032	-4.6049	5.5E-39	1.3E-37	1E-34	ENSRNO	protein_coding
Hcn1	-1.7899	-3.4579	8.5E-07	3.8E-06	0.01616	ENSRNO	protein_coding

Fgf10	-1.9569	-3.8823	0.00043	0.00129	1	ENSRNO	protein_coding
Ccdc152	-2.5633	-5.9106	4.3E-10	2.8E-09	8.2E-06	ENSRNO	protein_coding
Ghr	-4.6488	-25.086	1.3E-13	1.1E-12	2.5E-09	ENSRNO	protein_coding
LOC1009	-6.9703	-125.39	0.00765	0.01775	1	ENSRNO	protein_coding
Fyb	-2.2966	-4.9129	0.00013	0.00042	1	ENSRNO	protein_coding
Osmr	-5.095	-34.178	5E-178	9E-176	1E-173	ENSRNO	protein_coding
Lifr	-3.2351	-9.4157	2.9E-65	1.2E-63	5.5E-61	ENSRNO	protein_coding
Ranbp3l	2.38788	5.23389	0.00077	0.00221	1	ENSRNO	protein_coding
Nadk2	1.51938	2.86668	1.1E-16	1.1E-15	2.2E-12	ENSRNO	protein_coding
Capsl	-4.948	-30.866	0.00022	0.00069	1	ENSRNO	protein_coding
Adamts12	-2.9624	-7.7942	1E-103	1E-101	3E-99	ENSRNO	protein_coding
Pdzd2	4.38084	20.8336	6.1E-16	5.9E-15	1.2E-11	ENSRNO	protein_coding
Cdh6	-5.5949	-48.331	0.02312	0.04712	1	ENSRNO	protein_coding
Cdh9	5.47396	44.4453	0.02293	0.04682	1	ENSRNO	protein_coding
Cdh10	4.51552	22.8721	2.8E-14	2.5E-13	5.4E-10	ENSRNO	protein_coding
Cdh18	1.55401	2.93631	4.2E-07	2E-06	0.00803	ENSRNO	protein_coding
Fam105a	1.80526	3.49492	4.7E-26	7.4E-25	8.9E-22	ENSRNO	protein_coding
Ctnnd2	-6.1328	-70.17	3E-269	1E-266	5E-265	ENSRNO	protein_coding
Sema5a	-7.8639	-232.96	0	0	0	ENSRNO	protein_coding
Car3	-2.864	-7.2802	9E-165	1E-162	2E-160	ENSRNO	protein_coding
Car13	-6.3363	-80.801	9.1E-37	2.1E-35	1.7E-32	ENSRNO	protein_coding
LOC6883	3.57118	11.8859	3.8E-08	2E-07	0.00073	ENSRNO	protein_coding
Chmp4c	-1.6722	-3.1869	1.4E-55	5.1E-54	2.7E-51	ENSRNO	protein_coding
Fabp12	-8.5238	-368.05	0.00042	0.00126	1	ENSRNO	protein_coding
Fabp5	-6.9948	-127.54	9.4E-61	3.7E-59	1.8E-56	ENSRNO	protein_coding
Hnf4g	6.69062	103.295	0.0049	0.01192	1	ENSRNO	protein_coding
Cp	-2.3863	-5.2282	2.3E-40	5.7E-39	4.4E-36	ENSRNO	protein_coding
Agtr1b	5.34603	40.6737	2.2E-21	2.9E-20	4.2E-17	ENSRNO	protein_coding
Nlgn1	-6.9783	-126.09	1.6E-54	5.6E-53	3.1E-50	ENSRNO	protein_coding
Tnik	5.26137	38.3557	4.9E-14	4.2E-13	9.3E-10	ENSRNO	protein_coding
LOC1009	2.79559	6.94316	0.00232	0.00604	1	ENSRNO	protein_coding
Eif5a2	-5.372	-41.413	1E-173	2E-171	2E-169	ENSRNO	protein_coding
Kcnmb2	2.42303	5.36298	1E-13	8.8E-13	2E-09	ENSRNO	protein_coding
Gnb4	6.27141	77.2474	2.4E-55	8.4E-54	4.6E-51	ENSRNO	protein_coding
Usp13	-1.7254	-3.3067	1.4E-58	5.4E-57	2.7E-54	ENSRNO	protein_coding
Spry1	1.43557	2.70489	1.3E-06	5.5E-06	0.02401	ENSRNO	protein_coding
Ankrd50	-1.7949	-3.47	6.5E-62	2.6E-60	1.2E-57	ENSRNO	protein_coding
LOC1083	1.37572	2.59498	2.4E-05	8.9E-05	0.46031	ENSRNO	lincRNA
AABR070	2.08092	4.23076	7.5E-25	1.1E-23	1.4E-20	ENSRNO	pseudogene
Slc7a11	3.7997	13.9259	2E-121	2E-119	4E-117	ENSRNO	protein_coding
Mgarp	-4.0009	-16.01	2.7E-15	2.5E-14	5.1E-11	ENSRNO	protein_coding
Foxo1	1.38732	2.61592	8.8E-11	6E-10	1.7E-06	ENSRNO	protein_coding
Postn	5.63266	49.6133	0	0	0	ENSRNO	protein_coding
LOC1036	-2.6614	-6.3264	5.8E-05	0.0002	1	ENSRNO	protein_coding
Smad9	-1.3633	-2.5727	1.4E-38	3.5E-37	2.7E-34	ENSRNO	protein_coding
Ccna1	-6.5937	-96.585	2.3E-11	1.7E-10	4.5E-07	ENSRNO	protein_coding
AC129365	-1.6391	-3.1147	0.00014	0.00046	1	ENSRNO	pseudogene
Tm4sf4	-3.1553	-8.909	2.2E-10	1.4E-09	4.2E-06	ENSRNO	protein_coding
Med12l	-8.1493	-283.92	6.9E-30	1.3E-28	1.3E-25	ENSRNO	protein_coding

Igsf10	-5.6105	-48.859	3.5E-18	3.9E-17	6.7E-14	ENSRNO	protein_coding
Mme	-3.3689	-10.331	3E-181	6E-179	7E-177	ENSRNO	protein_coding
AABR070	4.10106	17.161	0.00288	0.00736	1	ENSRNO	pseudogene
AABR070	2.56295	5.90916	0.00784	0.01812	1	ENSRNO	lincRNA
AABR070	4.381	20.8359	0.00098	0.00274	1	ENSRNO	lincRNA
AABR070	-1.9233	-3.793	0.0142	0.03076	1	ENSRNO	protein_coding
Ptx3	-1.7901	-3.4585	0.0027	0.00695	1	ENSRNO	protein_coding
Ppm1l	-1.4935	-2.8156	2.2E-46	6.5E-45	4.2E-42	ENSRNO	protein_coding
AABR070	2.03821	4.10734	2.9E-05	0.00011	0.54945	ENSRNO	pseudogene
AABR070	2.83956	7.15803	5.2E-05	0.00018	0.98741	ENSRNO	protein_coding
Bche	7.09435	136.651	3.3E-21	4.2E-20	6.2E-17	ENSRNO	protein_coding
Serpini1	2.6638	6.33702	1.5E-41	3.9E-40	2.8E-37	ENSRNO	protein_coding
Rxfp1	-2.382	-5.2127	7.8E-37	1.8E-35	1.5E-32	ENSRNO	protein_coding
Fam198b	-3.226	-9.357	2E-141	2E-139	3E-137	ENSRNO	protein_coding
Gria2	3.3282	10.0436	2.3E-13	1.9E-12	4.4E-09	ENSRNO	protein_coding
AABR070	2.25157	4.76202	1E-22	1.4E-21	2E-18	ENSRNO	lincRNA
Gucy1b3	3.73803	13.3432	1.6E-08	8.9E-08	0.00031	ENSRNO	protein_coding
Fgg	-4.8131	-28.112	8E-33	1.6E-31	1.5E-28	ENSRNO	protein_coding
Sfrp2	6.29093	78.2995	1.1E-06	5E-06	0.02163	ENSRNO	protein_coding
Tlr2	-4.468	-22.131	2.2E-05	8.2E-05	0.42239	ENSRNO	protein_coding
Tigd4	-7.292	-156.71	0.00267	0.00687	1	ENSRNO	protein_coding
Tmem154	6.08056	67.6753	3.1E-06	1.3E-05	0.05901	ENSRNO	protein_coding
Rn60_2_1	-1.4843	-2.7979	0.00025	0.00077	1	ENSRNO	processed_pseudogene
Fam160a	-1.4305	-2.6955	3.3E-16	3.3E-15	6.3E-12	ENSRNO	protein_coding
Rn60_2_1	2.01335	4.03719	0.00647	0.01528	1	ENSRNO	antisense_RNA
Dclk2	-1.6024	-3.0365	1.3E-07	6.5E-07	0.0025	ENSRNO	protein_coding
Lrrc71	2.56479	5.91668	0.02119	0.04367	1	ENSRNO	protein_coding
Pear1	7.37177	165.625	6.3E-24	9.1E-23	1.2E-19	ENSRNO	protein_coding
Nes	5.78983	55.3238	5E-170	7E-168	9E-166	ENSRNO	protein_coding
Paqr6	2.11517	4.3324	2.8E-07	1.4E-06	0.00539	ENSRNO	protein_coding
Sema4a	-1.5336	-2.8951	0.00189	0.005	1	ENSRNO	protein_coding
Mex3a	-1.6329	-3.1013	9.3E-05	0.00031	1	ENSRNO	protein_coding
Adam15	3.85788	14.4989	7E-220	2E-217	1E-215	ENSRNO	protein_coding
Chrnb2	1.70142	3.25221	1.3E-05	4.9E-05	0.23967	ENSRNO	protein_coding
S100a1	1.5514	2.93102	9.2E-15	8.4E-14	1.8E-10	ENSRNO	protein_coding
S100a13	1.5886	3.00758	1.5E-11	1.1E-10	2.8E-07	ENSRNO	protein_coding
S100a5	2.0538	4.15198	0.00088	0.0025	1	ENSRNO	protein_coding
S100a7a	5.77277	54.6734	0.01659	0.03517	1	ENSRNO	protein_coding
Sprr1a	3.90375	14.9674	8.1E-74	4E-72	1.5E-69	ENSRNO	protein_coding
Kprp	-3.0206	-8.1148	7E-10	4.4E-09	1.3E-05	ENSRNO	protein_coding
Crnn	7.4205	171.315	1E-225	3E-223	2E-221	ENSRNO	protein_coding
Flg2	4.11174	17.2885	0.00206	0.00543	1	ENSRNO	protein_coding
AABR070	2.32958	5.02657	1E-10	6.8E-10	1.9E-06	ENSRNO	protein_coding
Tchh	6.19539	73.2822	2E-79	1E-77	3.7E-75	ENSRNO	protein_coding
Tdrkh	-6.6036	-97.248	2.6E-15	2.4E-14	4.9E-11	ENSRNO	protein_coding
Cgn	2.24796	4.75012	7.3E-08	3.7E-07	0.00138	ENSRNO	protein_coding
Tnfaip8l2	1.79114	3.46088	0.00672	0.01582	1	ENSRNO	protein_coding
Ciart	2.38545	5.22509	2.1E-35	4.7E-34	4.1E-31	ENSRNO	protein_coding
Itga10	3.97319	15.7054	4.6E-81	2.5E-79	8.7E-77	ENSRNO	protein_coding

Gja5	3.13326	8.77413	0.02418	0.04909	1	ENSRNO	protein_coding
Tbx15	-2.0247	-4.0691	5.8E-48	1.7E-46	1.1E-43	ENSRNO	protein_coding
Fam46c	3.06479	8.36745	2E-11	1.4E-10	3.8E-07	ENSRNO	protein_coding
Vtcn1	-1.9339	-3.8208	0.0077	0.01784	1	ENSRNO	protein_coding
Ptgfrn	-1.6308	-3.0969	3E-59	1.2E-57	5.8E-55	ENSRNO	protein_coding
Igsf3	-2.2595	-4.7884	1E-108	1E-106	2E-104	ENSRNO	protein_coding
Mab21l3	2.09519	4.27282	0.00011	0.00036	1	ENSRNO	protein_coding
Ngf	4.2591	19.1478	5.1E-53	1.7E-51	9.8E-49	ENSRNO	protein_coding
Sycp1	5.54771	46.7765	5.4E-24	7.8E-23	1E-19	ENSRNO	protein_coding
Nr1h5	-5.8204	-56.508	0.01889	0.03947	1	ENSRNO	protein_coding
Fam212b	3.95754	15.536	8.6E-07	3.9E-06	0.0164	ENSRNO	protein_coding
Cd53	-2.1858	-4.5498	1.7E-07	8.1E-07	0.00314	ENSRNO	protein_coding
Slc16a4	-2.0423	-4.1189	6E-12	4.5E-11	1.1E-07	ENSRNO	protein_coding
Gnat2	-3.1337	-8.7771	3.3E-21	4.3E-20	6.4E-17	ENSRNO	protein_coding
Mybphl	-4.1741	-18.052	3E-07	1.4E-06	0.00579	ENSRNO	protein_coding
Celsr2	-2.6243	-6.166	2.7E-33	5.6E-32	5.1E-29	ENSRNO	protein_coding
RGD1310	5.00473	32.1051	7.1E-11	4.9E-10	1.3E-06	ENSRNO	protein_coding
RGD1309	2.66941	6.36168	0.00026	0.0008	1	ENSRNO	protein_coding
Aknad1	-3.5834	-11.987	1.2E-05	4.8E-05	0.2349	ENSRNO	protein_coding
Vav3	6.02776	65.2432	3.4E-65	1.5E-63	6.5E-61	ENSRNO	protein_coding
Col11a1	-5.6219	-49.244	5E-146	6E-144	9E-142	ENSRNO	protein_coding
Vcam1	2.86721	7.29652	5.5E-19	6.3E-18	1E-14	ENSRNO	protein_coding
Gpr88	2.11954	4.34555	5.7E-06	2.3E-05	0.1082	ENSRNO	protein_coding
Dpyd	4.3921	20.9968	5.5E-07	2.6E-06	0.01055	ENSRNO	protein_coding
F3	3.79952	13.9241	5.9E-77	3E-75	1.1E-72	ENSRNO	protein_coding
Pde5a	-1.8691	-3.653	3.1E-24	4.5E-23	5.8E-20	ENSRNO	protein_coding
Synpo2	1.86101	3.63262	1.8E-08	9.6E-08	0.00034	ENSRNO	protein_coding
Prss12	-5.1686	-35.966	4.2E-19	4.8E-18	8E-15	ENSRNO	protein_coding
Tram1l1	-9.7881	-884.15	4.6E-05	0.00016	0.87666	ENSRNO	protein_coding
Ndst4	6.10519	68.8409	5.6E-13	4.5E-12	1.1E-08	ENSRNO	protein_coding
Casp6	-2.1979	-4.588	2.4E-12	1.8E-11	4.5E-08	ENSRNO	protein_coding
Mcub	-1.5121	-2.8523	2.5E-15	2.3E-14	4.7E-11	ENSRNO	protein_coding
Pitx2	2.2012	4.59861	1.9E-26	3.1E-25	3.7E-22	ENSRNO	protein_coding
Etnppl	3.75684	13.5183	2.6E-08	1.4E-07	0.00049	ENSRNO	protein_coding
AABR070	-1.486	-2.8011	0.00026	0.00082	1	ENSRNO	lincRNA
Dkk2	7.33509	161.466	1E-154	2E-152	3E-150	ENSRNO	protein_coding
Npnt	-2.2256	-4.6771	2E-103	1E-101	3E-99	ENSRNO	protein_coding
Cxxc4	3.8493	14.413	2.5E-06	1.1E-05	0.04721	ENSRNO	protein_coding
Manba	3.13364	8.77644	3.4E-61	1.3E-59	6.4E-57	ENSRNO	protein_coding
Ddit4l	-5.4189	-42.781	1.6E-07	7.7E-07	0.00298	ENSRNO	protein_coding
Dapp1	3.22752	9.36654	2.2E-43	6.1E-42	4.3E-39	ENSRNO	protein_coding
AABR070	6.41608	85.3948	0.00716	0.01675	1	ENSRNO	lincRNA
Adh1	4.91471	30.1631	3.2E-59	1.2E-57	6.2E-55	ENSRNO	protein_coding
Bmpr1b	6.85345	115.636	1.7E-61	6.7E-60	3.2E-57	ENSRNO	protein_coding
Gbp5	2.09126	4.26119	0.01109	0.02464	1	ENSRNO	protein_coding
Gbp4	4.37204	20.7069	6.3E-07	2.9E-06	0.01206	ENSRNO	protein_coding
Gbp3	2.06011	4.17018	0.00468	0.01143	1	ENSRNO	protein_coding
Clca5	1.9064	3.74873	0.00163	0.00438	1	ENSRNO	protein_coding
Clca4l	1.78287	3.4411	1E-08	5.8E-08	0.0002	ENSRNO	protein_coding

Col24a1	2.14955	4.43688	0.00359	0.00899	1	ENSRNO	protein_coding
Cyr61	-2.2074	-4.6186	6E-80	3.2E-78	1.1E-75	ENSRNO	protein_coding
LOC1025	-2.097	-4.2782	1.3E-07	6.5E-07	0.00248	ENSRNO	lincRNA
Ddah1	-3.3616	-10.279	2E-101	1E-99	3E-97	ENSRNO	protein_coding
Mcoln3	3.82968	14.2183	1.7E-52	5.5E-51	3.2E-48	ENSRNO	protein_coding
Ctbs	-4.7074	-26.125	1E-245	4E-243	3E-241	ENSRNO	protein_coding
Ifi44l	2.56121	5.90204	0.00114	0.00316	1	ENSRNO	protein_coding
Ptgfr	-6.7742	-109.46	3.2E-41	8.2E-40	6.1E-37	ENSRNO	protein_coding
Nexn	-3.2703	-9.6487	7.7E-88	4.7E-86	1.5E-83	ENSRNO	protein_coding
Ak5	-7.5459	-186.88	4.8E-52	1.6E-50	9.1E-48	ENSRNO	protein_coding
St6galnac	7.73713	213.358	0.0011	0.00306	1	ENSRNO	protein_coding
Slc44a5	-6.4005	-84.476	0.00866	0.01978	1	ENSRNO	protein_coding
AABR070	5.21947	37.2577	6.7E-12	5E-11	1.3E-07	ENSRNO	pseudogene
LOC1036	-3.4756	-11.124	9.2E-26	1.5E-24	1.8E-21	ENSRNO	protein_coding
Clic2	1.68994	3.22643	3.6E-23	5.1E-22	6.9E-19	ENSRNO	protein_coding
AABR070	2.91997	7.56833	0.00947	0.02141	1	ENSRNO	pseudogene
RT1-M3-1	1.72232	3.29967	3.7E-16	3.6E-15	7E-12	ENSRNO	protein_coding
Rn50_20_	-1.7151	-3.2832	2E-05	7.5E-05	0.38384	ENSRNO	antisense_RNA
Rnf39	2.98261	7.90417	6E-20	7.2E-19	1.1E-15	ENSRNO	protein_coding
Hspa1a	-1.9638	-3.901	3.9E-05	0.00014	0.73759	ENSRNO	protein_coding
LOC1003	1.67138	3.18519	2E-15	1.9E-14	3.8E-11	ENSRNO	protein_coding
Rn60_20_	2.44613	5.44951	0.00184	0.00487	1	ENSRNO	processed_pseudogene
RT1-N2	1.5669	2.96268	7.2E-19	8.2E-18	1.4E-14	ENSRNO	protein_coding
Rn50_20_	2.98766	7.93188	0.00945	0.02137	1	ENSRNO	unprocessed_pseudogene
Rn60_20_	1.89506	3.71937	0.00319	0.00808	1	ENSRNO	antisense_RNA
Flot1	1.35031	2.54966	3.2E-29	5.6E-28	6E-25	ENSRNO	protein_coding
Rn50_20_	-1.5169	-2.8618	3.4E-06	1.4E-05	0.06402	ENSRNO	lincRNA
RT1-DMb	5.4008	42.2477	4.2E-05	0.00015	0.79791	ENSRNO	protein_coding
Psmb9	2.35272	5.10787	6.1E-35	1.3E-33	1.2E-30	ENSRNO	protein_coding
RT1-Bb	5.75704	54.0805	8.2E-12	6E-11	1.6E-07	ENSRNO	protein_coding
RT1-Ba	6.04287	65.9303	2.4E-38	5.7E-37	4.6E-34	ENSRNO	protein_coding
RT1-Db1	6.16634	71.8215	4.9E-20	5.9E-19	9.4E-16	ENSRNO	protein_coding
RT1-Db2	6.98111	126.335	0.00328	0.00829	1	ENSRNO	protein_coding
RT1-Da	6.20715	73.8818	2.7E-22	3.6E-21	5E-18	ENSRNO	protein_coding
Egfl8	1.53766	2.90324	0.0026	0.00671	1	ENSRNO	protein_coding
Ppt2	1.38679	2.61496	1.3E-13	1.1E-12	2.4E-09	ENSRNO	protein_coding
Tnxb	4.3099	19.8339	3E-280	2E-277	7E-276	ENSRNO	processed_transcript
RT1-CE6	4.00566	16.0629	8.6E-06	3.4E-05	0.16272	ENSRNO	protein_coding
RT1-CE7	2.44117	5.43083	3.8E-38	8.9E-37	7.2E-34	ENSRNO	protein_coding
Hspa1b	-1.694	-3.2356	9.9E-05	0.00033	1	ENSRNO	protein_coding
Rn60_20_	2.48348	5.59243	0.00147	0.00399	1	ENSRNO	lincRNA
Vwa7	3.02565	8.1435	3.5E-14	3E-13	6.6E-10	ENSRNO	protein_coding
Sapcd1	4.16985	17.9991	0.00202	0.00533	1	ENSRNO	protein_coding
Rn60_20_	6.06199	66.8099	0.01453	0.03137	1	ENSRNO	protein_coding
G6b	2.11685	4.33746	0.01895	0.03958	1	ENSRNO	protein_coding
RT1-DMb	3.79297	13.8611	1.8E-06	7.8E-06	0.03441	ENSRNO	protein_coding
RT1-DMa	-3.3697	-10.336	0.00998	0.02245	1	ENSRNO	protein_coding
Rn60_20_	3.07325	8.4167	2.6E-06	1.1E-05	0.05012	ENSRNO	lincRNA
RT1-CE9-	1.50918	2.84648	5.1E-14	4.4E-13	9.6E-10	ENSRNO	unprocessed_pseudogene

RT1-A2	1.6187	3.07099	1.8E-24	2.7E-23	3.5E-20	ENSRNO	protein_coding
B3galt4	5.61902	49.1467	3.4E-11	2.4E-10	6.5E-07	ENSRNO	protein_coding
Ggnbp1	1.9101	3.75836	6.8E-05	0.00023	1	ENSRNO	protein_coding
Pxt1	2.4893	5.61506	0.01365	0.0297	1	ENSRNO	protein_coding
Grm4	2.50861	5.69071	1.3E-07	6.7E-07	0.00256	ENSRNO	processed_transcript
Rn60_20_	1.56058	2.94972	4.5E-07	2.1E-06	0.00861	ENSRNO	sense_intronic
RGD1564	1.74431	3.35035	2.2E-19	2.5E-18	4.1E-15	ENSRNO	protein_coding
Pacsin1	2.35129	5.1028	1.7E-08	9.5E-08	0.00033	ENSRNO	protein_coding
Scube3	3.35935	10.2628	1.9E-11	1.4E-10	3.7E-07	ENSRNO	protein_coding
Tead3	-1.6757	-3.1947	4.4E-55	1.5E-53	8.4E-51	ENSRNO	protein_coding
Dnah8	-2.2903	-4.8914	4.2E-13	3.4E-12	7.9E-09	ENSRNO	protein_coding
Ubash3a	-6.4924	-90.034	0.00779	0.01801	1	ENSRNO	protein_coding
Rsph1	6.95164	123.78	0.0034	0.00856	1	ENSRNO	protein_coding
Slc37a1	5.72145	52.763	9.2E-06	3.6E-05	0.17547	ENSRNO	protein_coding
Pde9a	6.06367	66.8877	3.5E-36	7.6E-35	6.6E-32	ENSRNO	protein_coding
Hsf2bp	5.02931	32.6567	6.4E-92	4.2E-90	1.2E-87	ENSRNO	protein_coding
Icoslg	5.71445	52.5075	1E-27	1.8E-26	2E-23	ENSRNO	protein_coding
Trpm2	4.94398	30.7813	4.5E-08	2.4E-07	0.00086	ENSRNO	protein_coding
Col18a1	3.51628	11.4421	7E-234	2E-231	1E-229	ENSRNO	protein_coding
Col6a1	2.29117	4.89454	1E-114	1E-112	2E-110	ENSRNO	protein_coding
Col6a2	1.78634	3.44938	4.4E-71	2.1E-69	8.4E-67	ENSRNO	protein_coding
RGD1563	-6.2504	-76.132	0.01044	0.02337	1	ENSRNO	protein_coding
LOC1036	3.39941	10.5517	0.00024	0.00074	1	ENSRNO	protein_coding
Ggt5	1.50917	2.84647	5.3E-07	2.4E-06	0.00999	ENSRNO	protein_coding
Ggt1	-2.576	-5.9627	7E-126	7E-124	1E-121	ENSRNO	protein_coding
Rn60_20_	-2.3087	-4.9542	1.2E-19	1.5E-18	2.3E-15	ENSRNO	lincRNA
Adora2a	1.96387	3.90107	0.01316	0.02874	1	ENSRNO	protein_coding
Rab36	-4.242	-18.923	0.00166	0.00444	1	ENSRNO	protein_coding
Pcdh15	2.35934	5.13135	0.00175	0.00466	1	ENSRNO	protein_coding
Bicc1	-6.6947	-103.59	5E-129	6E-127	1E-124	ENSRNO	protein_coding
Fam13c	2.16471	4.48378	9E-09	5E-08	0.00017	ENSRNO	protein_coding
ENSRNO	5.28369	38.9538	3.5E-12	2.7E-11	6.7E-08	ENSRNO	protein_coding
Zfp365	1.50791	2.84397	1E-13	8.8E-13	2E-09	ENSRNO	protein_coding
Lrrtm3	6.72384	105.7	0.00486	0.01183	1	ENSRNO	protein_coding
Mypn	3.44655	10.9022	0.01269	0.02783	1	ENSRNO	protein_coding
Tet1	-2.6238	-6.1639	0.01286	0.02817	1	ENSRNO	protein_coding
Sowahc	1.86294	3.63747	4E-64	1.7E-62	7.6E-60	ENSRNO	protein_coding
Ddit4	1.6587	3.15732	8.2E-09	4.6E-08	0.00016	ENSRNO	protein_coding
Vsir	3.16312	8.95766	5.7E-47	1.7E-45	1.1E-42	ENSRNO	protein_coding
Adamts14	-1.952	-3.8692	0.01744	0.03678	1	ENSRNO	protein_coding
Pald1	-11.295	-2511.9	2.3E-06	1E-05	0.04463	ENSRNO	protein_coding
Tysnd1	7.14746	141.775	1.1E-17	1.2E-16	2.2E-13	ENSRNO	protein_coding
Aifm2	-4.6194	-24.58	9E-93	6.1E-91	1.7E-88	ENSRNO	protein_coding
H2afy2	-6.395	-84.158	7.5E-50	2.4E-48	1.4E-45	ENSRNO	protein_coding
Col13a1	5.56468	47.3299	0.02164	0.04448	1	ENSRNO	protein_coding
RGD1305	2.70606	6.52537	3.4E-25	5.2E-24	6.5E-21	ENSRNO	protein_coding
Vgll2	-3.6104	-12.214	0.00836	0.01918	1	ENSRNO	protein_coding
Slc35f1	4.7668	27.2239	0.00034	0.00104	1	ENSRNO	protein_coding
Pln	-4.1612	-17.891	1.2E-19	1.4E-18	2.3E-15	ENSRNO	protein_coding

Man1a1	7.12387	139.476	9E-122	9E-120	2E-117	ENSRNO	protein_coding
Msl3l2	6.56094	94.415	3E-15	2.8E-14	5.8E-11	ENSRNO	protein_coding
Smpdl3a	-5.1062	-34.444	2E-16	2E-15	3.9E-12	ENSRNO	protein_coding
Tspyl4	-3.01	-8.0558	0.00074	0.00212	1	ENSRNO	protein_coding
Lama4	-7.0571	-133.16	5E-127	5E-125	9E-123	ENSRNO	protein_coding
Fyn	-2.12	-4.347	0.00015	0.00048	1	ENSRNO	protein_coding
Ak9	1.49743	2.82339	0.01053	0.02354	1	ENSRNO	protein_coding
Armc2	-2.3185	-4.9882	2.8E-27	4.7E-26	5.4E-23	ENSRNO	protein_coding
AC13275	-6.4004	-84.472	0.00859	0.01965	1	ENSRNO	pseudogene
Sobp	-5.5764	-47.716	5.7E-16	5.6E-15	1.1E-11	ENSRNO	protein_coding
Cd24	3.65124	12.5641	6E-258	2E-255	1E-253	ENSRNO	protein_coding
Ddo	1.64463	3.12667	0.00943	0.02132	1	ENSRNO	protein_coding
Aim1	6.31995	79.8905	2.1E-28	3.6E-27	3.9E-24	ENSRNO	protein_coding
Prdm1	3.6198	12.2933	2.7E-11	1.9E-10	5.2E-07	ENSRNO	protein_coding
Popdc3	-2.7273	-6.6222	0.01577	0.03367	1	ENSRNO	protein_coding
AABR070	-4.8197	-28.242	0.00285	0.00729	1	ENSRNO	pseudogene
Sim1	3.50797	11.3763	1.1E-18	1.3E-17	2.1E-14	ENSRNO	protein_coding
Il1rn	-4.5526	-23.468	4.2E-88	2.6E-86	8E-84	ENSRNO	protein_coding
Nsmf	-1.8188	-3.5278	2.8E-28	4.8E-27	5.3E-24	ENSRNO	protein_coding
Tor4a	2.64958	6.27484	6.2E-80	3.3E-78	1.2E-75	ENSRNO	protein_coding
Mamdc4	1.34561	2.54138	0.00203	0.00535	1	ENSRNO	protein_coding
Tmem141	-6.5899	-96.331	4.1E-07	1.9E-06	0.00773	ENSRNO	protein_coding
Fcna	3.73153	13.2832	1.4E-07	7E-07	0.00271	ENSRNO	protein_coding
Bmyc	6.13959	70.5018	2.1E-06	8.8E-06	0.03925	ENSRNO	protein_coding
Sohlh1	1.94342	3.84616	5.2E-09	3E-08	9.8E-05	ENSRNO	protein_coding
Nacc2	-1.9083	-3.7537	5.1E-67	2.3E-65	9.7E-63	ENSRNO	protein_coding
Card9	3.53968	11.6292	7.6E-12	5.6E-11	1.4E-07	ENSRNO	protein_coding
Fam69b	-2.5268	-5.7628	2.1E-33	4.3E-32	4E-29	ENSRNO	protein_coding
Abo	-3.9502	-15.458	9.9E-23	1.4E-21	1.9E-18	ENSRNO	protein_coding
Abo3	-7.0375	-131.37	3.1E-21	4E-20	5.9E-17	ENSRNO	protein_coding
Abo2	-6.8806	-117.83	0.00464	0.01135	1	ENSRNO	protein_coding
Adamtsl2	5.12791	34.9668	2.5E-15	2.3E-14	4.7E-11	ENSRNO	protein_coding
Dbh	-3.0872	-8.4982	1.2E-05	4.6E-05	0.22644	ENSRNO	protein_coding
Sardh	-1.6463	-3.1302	3.3E-16	3.3E-15	6.3E-12	ENSRNO	protein_coding
Vav2	-1.7162	-3.2856	3.9E-48	1.2E-46	7.3E-44	ENSRNO	protein_coding
Olfm1	7.11789	138.899	7.8E-65	3.3E-63	1.5E-60	ENSRNO	protein_coding
Ak8	5.63107	49.5588	0.01893	0.03954	1	ENSRNO	protein_coding
Aif1l	-4.7671	-27.23	1.1E-96	7.7E-95	2E-92	ENSRNO	protein_coding
Ass1	4.547	23.3766	1.4E-26	2.4E-25	2.8E-22	ENSRNO	protein_coding
Hmcn2	3.19404	9.15167	0.00037	0.00113	1	ENSRNO	processed_transcript
Ncs1	-1.548	-2.9241	9E-47	2.7E-45	1.7E-42	ENSRNO	protein_coding
AABR070	2.01843	4.05143	0.00041	0.00124	1	ENSRNO	lincRNA
Plpp7	-3.2574	-9.5624	9.1E-14	7.7E-13	1.7E-09	ENSRNO	protein_coding
Dnm1	-1.325	-2.5054	9.4E-32	1.8E-30	1.8E-27	ENSRNO	protein_coding
RGD1561	-7.786	-220.71	0.00129	0.00352	1	ENSRNO	protein_coding
Lcn2	-1.3558	-2.5594	0.00038	0.00114	1	ENSRNO	protein_coding
St6galnac	-1.3471	-2.544	1.2E-39	2.8E-38	2.2E-35	ENSRNO	protein_coding
Eng	-5.8792	-58.859	3E-243	9E-241	6E-239	ENSRNO	protein_coding
Mvb12b	-1.4243	-2.6838	1.1E-34	2.4E-33	2.2E-30	ENSRNO	protein_coding

Rabepk	-4.1558	-17.825	4.3E-29	7.7E-28	8.3E-25	ENSRNO	protein_coding
Traf1	3.03139	8.17597	1.4E-41	3.8E-40	2.7E-37	ENSRNO	protein_coding
Rn60_3_(	6.25406	76.3239	0.01436	0.03106	1	ENSRNO	protein_coding
Stom	-7.5994	-193.94	2E-283	1E-280	4E-279	ENSRNO	protein_coding
Ggta1	-8.5543	-375.93	7.9E-70	3.6E-68	1.5E-65	ENSRNO	protein_coding
AABR070	-7.0412	-131.71	0.00383	0.00951	1	ENSRNO	lincRNA
Ptgs1	-2.1124	-4.3241	9.9E-97	7.3E-95	1.9E-92	ENSRNO	protein_coding
ENSRNO	5.29326	39.213	4.2E-18	4.6E-17	8E-14	ENSRNO	protein_coding
ENSRNO	8.29147	313.316	0.00046	0.00136	1	ENSRNO	protein_coding
Crb2	-5.8246	-56.672	0.01753	0.03695	1	ENSRNO	protein_coding
Nr5a1	-6.1836	-72.684	3.3E-41	8.5E-40	6.3E-37	ENSRNO	protein_coding
Olfml2a	-3.0082	-8.0457	1.3E-14	1.2E-13	2.5E-10	ENSRNO	protein_coding
AABR070	-2.2814	-4.8616	5.7E-11	4E-10	1.1E-06	ENSRNO	pseudogene
Zeb2	-2.4318	-5.3958	4E-107	3E-105	7E-103	ENSRNO	protein_coding
Lypd6b	-6.3852	-83.584	3.2E-21	4.2E-20	6.2E-17	ENSRNO	protein_coding
Rnd3	-1.6155	-3.0642	9.9E-57	3.6E-55	1.9E-52	ENSRNO	protein_coding
Rbm43	-2.117	-4.3379	2.5E-12	1.9E-11	4.7E-08	ENSRNO	protein_coding
Nmi	-1.6154	-3.0641	1.2E-06	5.5E-06	0.02361	ENSRNO	protein_coding
Tnfaip6	-6.2399	-75.579	1.6E-06	6.9E-06	0.0301	ENSRNO	protein_coding
Neb	-2.8269	-7.0956	1.6E-17	1.7E-16	3E-13	ENSRNO	protein_coding
Fmnl2	-2.6177	-6.1379	3.8E-81	2.1E-79	7.3E-77	ENSRNO	protein_coding
Cytip	-2.7698	-6.8201	3.1E-15	2.9E-14	6E-11	ENSRNO	protein_coding
Baz2b	-1.5321	-2.8922	5.7E-32	1.1E-30	1.1E-27	ENSRNO	protein_coding
Ly75	-6.3363	-80.798	9.7E-07	4.3E-06	0.01845	ENSRNO	protein_coding
Dpp4	1.8272	3.54847	0.00097	0.00273	1	ENSRNO	protein_coding
Ifih1	-6.9828	-126.48	6.4E-30	1.2E-28	1.2E-25	ENSRNO	protein_coding
Fign	-5.3487	-40.75	3.5E-36	7.8E-35	6.7E-32	ENSRNO	protein_coding
Grb14	-1.4109	-2.659	6.1E-20	7.3E-19	1.2E-15	ENSRNO	protein_coding
Galnt3	-6.7591	-108.32	3E-148	4E-146	5E-144	ENSRNO	protein_coding
B3galt1	-7.3912	-167.87	0.00234	0.00608	1	ENSRNO	protein_coding
Stk39	-5.0556	-33.257	6.4E-89	4E-87	1.2E-84	ENSRNO	protein_coding
Nostrin	2.69968	6.49658	3.1E-13	2.5E-12	5.8E-09	ENSRNO	protein_coding
Lrp2	-1.5296	-2.887	0.0109	0.02428	1	ENSRNO	protein_coding
Klhl23	-4.049	-16.552	3.1E-82	1.7E-80	5.9E-78	ENSRNO	protein_coding
Mettl5	-1.661	-3.1624	1.4E-12	1.1E-11	2.6E-08	ENSRNO	protein_coding
Itga6	-6.3447	-81.274	0	0	0	ENSRNO	protein_coding
Gpr155	1.60516	3.04229	1.3E-07	6.3E-07	0.00243	ENSRNO	protein_coding
Wipf1	-6.6304	-99.071	1E-198	3E-196	3E-194	ENSRNO	protein_coding
Chn1	-1.8237	-3.5398	2.3E-64	9.5E-63	4.3E-60	ENSRNO	protein_coding
Hoxd13	-2.6335	-6.2054	7E-114	7E-112	1E-109	ENSRNO	protein_coding
Hoxd11	-5.9595	-62.23	0.01521	0.03263	1	ENSRNO	protein_coding
Hoxd10	-4.2779	-19.399	1.4E-78	7.3E-77	2.7E-74	ENSRNO	protein_coding
Hoxd9	-2.2059	-4.6136	1.1E-43	3.1E-42	2.1E-39	ENSRNO	protein_coding
Hoxd8	-2.0064	-4.0179	5.3E-23	7.4E-22	1E-18	ENSRNO	protein_coding
Hoxd4	-1.4849	-2.799	1.3E-06	5.9E-06	0.02557	ENSRNO	protein_coding
Ttc30b	-2.0292	-4.0817	1.1E-31	2.1E-30	2E-27	ENSRNO	protein_coding
Ttc30a1	-3.4408	-10.859	4.9E-17	5E-16	9.3E-13	ENSRNO	protein_coding
Osbpl6	-1.9326	-3.8174	7.3E-28	1.2E-26	1.4E-23	ENSRNO	protein_coding
Fkbp7	-2.323	-5.0036	3.2E-30	5.9E-29	6.1E-26	ENSRNO	protein_coding

Ccdc141	2.01952	4.05449	0.00932	0.0211	1	ENSRNO	protein_coding
Pde1a	-3.8636	-14.556	2.3E-14	2E-13	4.4E-10	ENSRNO	protein_coding
AABR070	2.3329	5.03816	0.01977	0.04108	1	ENSRNO	pseudogene
Zfp804a	-4.8902	-29.655	8.1E-12	6E-11	1.5E-07	ENSRNO	protein_coding
Fam171b	-5.0402	-32.905	9E-100	7.3E-98	1.8E-95	ENSRNO	protein_coding
Ypel4	-1.3446	-2.5396	2.6E-05	9.7E-05	0.50263	ENSRNO	protein_coding
Ube2l6	-3.6237	-12.326	1.9E-46	5.6E-45	3.6E-42	ENSRNO	protein_coding
Smtnl1	-2.4758	-5.5628	0.01363	0.02965	1	ENSRNO	protein_coding
Slc43a1	-6.5112	-91.216	1.1E-21	1.5E-20	2.1E-17	ENSRNO	protein_coding
Rtn4rl2	-4.143	-17.667	5.3E-06	2.1E-05	0.10021	ENSRNO	protein_coding
Slc43a3	-5.9601	-62.254	0.01565	0.03345	1	ENSRNO	protein_coding
AC11163	1.92231	3.79028	0.00515	0.01246	1	ENSRNO	protein_coding
Ptprj	-5.8447	-57.468	2.9E-55	1E-53	5.5E-51	ENSRNO	protein_coding
Rapsn	-3.4231	-10.726	1.4E-39	3.3E-38	2.6E-35	ENSRNO	protein_coding
Lrp4	-2.9294	-7.6179	1.3E-60	5.2E-59	2.5E-56	ENSRNO	protein_coding
Creb3l1	-4.9185	-30.243	1E-149	2E-147	3E-145	ENSRNO	protein_coding
Syt13	-2.0066	-4.0184	1.4E-74	7.1E-73	2.8E-70	ENSRNO	protein_coding
Prdm11	-3.7676	-13.619	6E-06	2.4E-05	0.11319	ENSRNO	protein_coding
LOC1025	-3.9916	-15.907	0.00535	0.01291	1	ENSRNO	protein_coding
Tspan18	-7.3795	-166.52	1E-43	2.9E-42	2E-39	ENSRNO	protein_coding
AABR070	4.9069	30.0002	2.5E-08	1.4E-07	0.00048	ENSRNO	lincRNA
Prr5l	-6.7439	-107.18	1.1E-19	1.4E-18	2.2E-15	ENSRNO	protein_coding
Ldlrad3	-2.0724	-4.2058	7.2E-12	5.3E-11	1.4E-07	ENSRNO	protein_coding
Tcp11l1	-2.138	-4.4016	2.1E-44	5.9E-43	4.1E-40	ENSRNO	protein_coding
Depdc7	-2.0705	-4.2002	3.4E-16	3.3E-15	6.4E-12	ENSRNO	protein_coding
Ccdc73	1.77077	3.41235	0.0012	0.00329	1	ENSRNO	protein_coding
Wt1	-8.1414	-282.36	8E-136	9E-134	1E-131	ENSRNO	protein_coding
LOC6910	-2.4686	-5.535	0.00017	0.00056	1	ENSRNO	protein_coding
AABR070	2.8447	7.18359	0.00085	0.00241	1	ENSRNO	protein_coding
Lgr4	-1.784	-3.4437	5.6E-63	2.3E-61	1.1E-58	ENSRNO	protein_coding
Fibin	-3.637	-12.441	1.3E-18	1.5E-17	2.6E-14	ENSRNO	protein_coding
Aven	-1.5223	-2.8725	9.4E-21	1.2E-19	1.8E-16	ENSRNO	protein_coding
Fmn1	-2.0117	-4.0327	1.7E-40	4.3E-39	3.3E-36	ENSRNO	protein_coding
Grem1	-7.725	-211.57	6E-299	4E-296	1E-294	ENSRNO	protein_coding
Rasgrp1	-2.6819	-6.4168	6.2E-07	2.8E-06	0.01176	ENSRNO	protein_coding
Thbs1	-1.7219	-3.2988	1.7E-66	7.5E-65	3.2E-62	ENSRNO	protein_coding
Fsip1	-6.5271	-92.228	5.9E-19	6.8E-18	1.1E-14	ENSRNO	protein_coding
Disp2	2.77944	6.86584	0.00156	0.0042	1	ENSRNO	protein_coding
Chst14	3.2327	9.40027	6.8E-11	4.7E-10	1.3E-06	ENSRNO	protein_coding
Tyro3	-1.6343	-3.1044	4.9E-41	1.2E-39	9.3E-37	ENSRNO	protein_coding
AABR070	2.1436	4.41862	0.00226	0.00589	1	ENSRNO	protein_coding
LOC1009	6.7341	106.455	0.00854	0.01955	1	ENSRNO	protein_coding
Epb42	-2.2105	-4.6285	0.00735	0.01714	1	ENSRNO	protein_coding
Tgm7l1_1	-3.4644	-11.038	3E-15	2.8E-14	5.7E-11	ENSRNO	protein_coding
Ckmt1	1.84859	3.60147	0.01737	0.03665	1	ENSRNO	protein_coding
Frmd5	-6.6622	-101.28	1.5E-29	2.7E-28	2.9E-25	ENSRNO	protein_coding
Casc4	-5.5027	-45.338	1E-243	4E-241	2E-239	ENSRNO	protein_coding
Trim69	-3.2736	-9.6703	2.2E-06	9.2E-06	0.04097	ENSRNO	protein_coding
Duox2	-4.96	-31.125	1.2E-25	1.9E-24	2.3E-21	ENSRNO	protein_coding

Duoxa2	-2.6303	-6.1917	0.01832	0.03841	1	ENSRNO	protein_coding
Duoxa1	-5.4119	-42.574	1.6E-20	2E-19	3.1E-16	ENSRNO	protein_coding
Duox1	-5.8376	-57.188	2.4E-20	2.9E-19	4.5E-16	ENSRNO	protein_coding
LOC1025	-3.9947	-15.941	0.00339	0.00854	1	ENSRNO	lincRNA
Slc24a5	-6.5836	-95.906	3.1E-96	2.2E-94	5.8E-92	ENSRNO	protein_coding
Myef2	-7.8111	-224.58	3E-203	6E-201	5E-199	ENSRNO	protein_coding
Slc12a1	2.20896	4.62343	0.0003	0.00093	1	ENSRNO	protein_coding
Fbn1	-5.8358	-57.113	3.8E-29	6.8E-28	7.3E-25	ENSRNO	protein_coding
Prom2	2.41862	5.34659	4.4E-14	3.9E-13	8.4E-10	ENSRNO	protein_coding
Mal	2.84581	7.1891	6.1E-08	3.1E-07	0.00115	ENSRNO	protein_coding
Mertk	4.23807	18.8706	8.9E-14	7.6E-13	1.7E-09	ENSRNO	protein_coding
Fbln7	-4.9817	-31.597	0.00017	0.00056	1	ENSRNO	protein_coding
Il1a	2.41735	5.3419	1.1E-06	4.9E-06	0.02104	ENSRNO	protein_coding
Sirpa	2.1668	4.49026	1.8E-87	1.1E-85	3.4E-83	ENSRNO	protein_coding
Cpxm1	1.61557	3.06433	0.01918	0.03997	1	ENSRNO	protein_coding
Adam33	-5.1049	-34.412	2.6E-63	1E-61	4.9E-59	ENSRNO	protein_coding
Rnf24	-1.3961	-2.6318	0.00027	0.00083	1	ENSRNO	protein_coding
Smox	-5.2892	-39.103	4.2E-16	4.1E-15	8.1E-12	ENSRNO	protein_coding
Rassf2	-1.438	-2.7094	0.00125	0.00341	1	ENSRNO	protein_coding
Chgb	5.63042	49.5367	1.6E-84	9.4E-83	3.1E-80	ENSRNO	protein_coding
Lrrn4	-1.9253	-3.7981	0.0022	0.00575	1	ENSRNO	protein_coding
Plcb1	-1.6567	-3.1531	8E-18	8.7E-17	1.5E-13	ENSRNO	protein_coding
Sptlc3	-4.7574	-27.047	1.2E-07	6E-07	0.00228	ENSRNO	protein_coding
Ovol2	3.24544	9.48366	0.02087	0.04309	1	ENSRNO	protein_coding
Slc24a3	-2.1688	-4.4966	2.5E-34	5.4E-33	4.8E-30	ENSRNO	protein_coding
Nkx2-2	-6.6604	-101.15	0.00624	0.01483	1	ENSRNO	protein_coding
Entpd6	-1.6883	-3.2228	1.4E-09	8.8E-09	2.7E-05	ENSRNO	protein_coding
Sdcbp2	1.74123	3.34319	0.01024	0.02297	1	ENSRNO	protein_coding
Rad21l1	1.96588	3.9065	0.01434	0.03103	1	ENSRNO	protein_coding
Angpt4	-6.457	-87.85	2E-224	5E-222	4E-220	ENSRNO	protein_coding
Trib3	2.09756	4.27985	4.4E-31	8.4E-30	8.4E-27	ENSRNO	protein_coding
LOC5026	-2.5263	-5.761	0.02283	0.04667	1	ENSRNO	protein_coding
Defb29	8.69622	414.786	0.00022	0.0007	1	ENSRNO	protein_coding
Rem1	-3.0992	-8.5695	2.3E-06	9.9E-06	0.04436	ENSRNO	protein_coding
Foxs1	-5.7402	-53.451	8.8E-87	5.3E-85	1.7E-82	ENSRNO	protein_coding
Dusp15	-2.052	-4.1467	0.00212	0.00556	1	ENSRNO	protein_coding
Ttll9	-1.9152	-3.7716	0.00357	0.00893	1	ENSRNO	protein_coding
Nol4l	-4.9818	-31.598	0.00019	0.0006	1	ENSRNO	protein_coding
Bpifb5	4.08976	17.0271	0.00014	0.00047	1	ENSRNO	protein_coding
Asip	6.92133	121.207	0.00358	0.00897	1	ENSRNO	protein_coding
Ggt7	-2.0458	-4.129	1.4E-26	2.2E-25	2.6E-22	ENSRNO	protein_coding
Romo1	-3.9066	-14.997	7.3E-07	3.3E-06	0.01391	ENSRNO	protein_coding
LOC1009	-1.7512	-3.3665	0.0038	0.00945	1	ENSRNO	protein_coding
Src	-3.3624	-10.284	1E-182	2E-180	2E-178	ENSRNO	protein_coding
Tgm2	2.0476	4.13416	3.7E-24	5.4E-23	7.1E-20	ENSRNO	protein_coding
Lbp	-3.8163	-14.088	2.1E-23	2.9E-22	4E-19	ENSRNO	protein_coding
Jph2	-7.2938	-156.91	8E-125	8E-123	2E-120	ENSRNO	protein_coding
Gdap1l1	3.78049	13.7417	0.00535	0.01291	1	ENSRNO	protein_coding
Hnf4a	7.40926	169.985	0.00181	0.0048	1	ENSRNO	protein_coding

Ada	-5.0077	-32.17	3E-34	6.4E-33	5.8E-30	ENSRNO	protein_coding	
Slpil2	6.82167	113.117	0.00431	0.01062	1	ENSRNO	protein_coding	
Slpi	2.23518	4.70822	0.00048	0.00142	1	ENSRNO	protein_coding	
Rbpjl	1.58439	2.9988	0.00339	0.00855	1	ENSRNO	protein_coding	
Ube2c	-1.339	-2.5298	7.9E-18	8.6E-17	1.5E-13	ENSRNO	protein_coding	
Tnnc2	3.7803	13.7399	0.00582	0.01392	1	ENSRNO	protein_coding	
Pltp	-3.0847	-8.484	9.7E-42	2.6E-40	1.8E-37	ENSRNO	protein_coding	
Mmp9	1.56451	2.95777	6.9E-08	3.5E-07	0.00132	ENSRNO	protein_coding	
Slc12a5	1.83567	3.56938	1E-05	4E-05	0.19386	ENSRNO	protein_coding	
Zfp334	-2.0809	-4.2307	4.4E-11	3.1E-10	8.3E-07	ENSRNO	protein_coding	
Slc2a10	-3.2474	-9.4965	0.00048	0.00142	1	ENSRNO	protein_coding	
Eya2	2.19206	4.56958	0.00324	0.00819	1	ENSRNO	protein_coding	
Sulf2	2.23932	4.72174	3.7E-09	2.1E-08	7E-05	ENSRNO	protein_coding	
Ptgis	-4.2503	-19.031	6.2E-19	7E-18	1.2E-14	ENSRNO	protein_coding	
Snai1	-1.3729	-2.59	1.4E-16	1.4E-15	2.7E-12	ENSRNO	protein_coding	
Fam65c	-1.6744	-3.1919	3.3E-05	0.00012	0.6268	ENSRNO	protein_coding	
Atp9a	-1.3829	-2.608	7.1E-06	2.8E-05	0.13449	ENSRNO	protein_coding	
Dok5	-7.0938	-136.6	1.2E-82	6.4E-81	2.2E-78	ENSRNO	protein_coding	
Bmp7	-8.0955	-273.52	0.00084	0.0024	1	ENSRNO	protein_coding	
Npepl1	-7.3251	-160.35	1.9E-97	1.4E-95	3.6E-93	ENSRNO	protein_coding	
Lama5	1.61909	3.07181	7.9E-58	2.9E-56	1.5E-53	ENSRNO	protein_coding	
Slco4a1	-3.4607	-11.009	0.00019	0.00059	1	ENSRNO	protein_coding	
Mrgbp	-8.2649	-307.61	0.00098	0.00276	1	ENSRNO	protein_coding	
Slc17a9	-2.2789	-4.8529	1.6E-08	8.5E-08	0.0003	ENSRNO	protein_coding	
Col20a1	2.14654	4.42766	0.00026	0.0008	1	ENSRNO	protein_coding	
Stmn3	-2.3951	-5.2603	0.01889	0.03947	1	ENSRNO	protein_coding	
Oprl1	3.49456	11.2711	0.00099	0.00277	1	ENSRNO	protein_coding	
Smarcd3	-1.4492	-2.7305	3E-18	3.3E-17	5.7E-14	ENSRNO	protein_coding	
Iqca1l	-2.0298	-4.0835	0.01475	0.03176	1	ENSRNO	protein_coding	
Atg9b	1.49374	2.81617	0.00078	0.00223	1	ENSRNO	protein_coding	
Kcnh2	-1.7618	-3.3913	1.9E-10	1.2E-09	3.6E-06	ENSRNO	protein_coding	
Reln	8.8096	448.697	0.00018	0.00057	1	ENSRNO	protein_coding	
Ccdc146	-2.3779	-5.1977	0.00418	0.01032	1	ENSRNO	protein_coding	
Fgl2	-1.4895	-2.808	0.00476	0.0116	1	ENSRNO	protein_coding	
Gsap	-4.252	-19.054	1E-08	5.7E-08	0.00019	ENSRNO	protein_coding	
Magi2	-2.0528	-4.1492	1.2E-10	8.2E-10	2.3E-06	ENSRNO	protein_coding	
AABR070	-1.4353	-2.7044	0.00702	0.01644	1	ENSRNO	lincRNA	
Gnai1	-4.6412	-24.954	3.6E-84	2.1E-82	6.9E-80	ENSRNO	protein_coding	
Cacna2d1	-2.4158	-5.3363	1.3E-41	3.4E-40	2.4E-37	ENSRNO	protein_coding	
Pclo	4.441	21.7207	1.5E-06	6.6E-06	0.02881	ENSRNO	protein_coding	
AABR070	3.94233	15.373	6.9E-07	3.1E-06	0.01306	ENSRNO	lincRNA	
Sema3e	3.09585	8.54956	5.5E-44	1.5E-42	1.1E-39	ENSRNO	protein_coding	
Sema3d	-5.8117	-56.171	1.7E-92	1.2E-90	3.3E-88	ENSRNO	protein_coding	
Abcb1a	5.50432	45.3906	0	0	0	ENSRNO	protein_coding	
Rundc3b	3.19619	9.16535	0.00048	0.00142	1	ENSRNO	protein_coding	
Zfp804b	7.25515	152.763	1E-08	5.8E-08	0.0002	ENSRNO	protein_coding	
Cfap69	-1.9532	-3.8723	8.9E-09	5E-08	0.00017	ENSRNO	protein_coding	
Fzd1	4.49468	22.5442	5E-151	6E-149	9E-147	ENSRNO	protein_coding	
Gng11	-2.1107	-4.3189	1.9E-18	2.1E-17	3.5E-14	ENSRNO	protein_coding	

NEWGEN	-4.0298	-16.333	3E-105	3E-103	6E-101	ENSRNO	protein_coding
Casd1	-9.8122	-899.04	4.3E-05	0.00015	0.82628	ENSRNO	protein_coding
Ppp1r9a	-8.8777	-470.39	0.00022	0.00071	1	ENSRNO	protein_coding
Pon2	-1.6526	-3.144	6.8E-56	2.4E-54	1.3E-51	ENSRNO	protein_coding
Pdk4	-5.0503	-33.136	6.6E-27	1.1E-25	1.3E-22	ENSRNO	protein_coding
Dync1i1	-5.4497	-43.704	4.6E-30	8.5E-29	8.8E-26	ENSRNO	protein_coding
LOC1009	-1.776	-3.4246	5.1E-07	2.4E-06	0.00978	ENSRNO	protein_coding
NEWGEN	-3.7987	-13.916	1E-252	5E-250	3E-248	ENSRNO	protein_coding
Dlx6as	-1.5883	-3.0069	0.00787	0.01818	1	ENSRNO	antisense_RNA
Dlx6	-4.4756	-22.248	8.6E-09	4.8E-08	0.00016	ENSRNO	protein_coding
Dlx5	-3.9497	-15.452	1.2E-40	3E-39	2.2E-36	ENSRNO	protein_coding
C1galt1	-1.4323	-2.6987	9.4E-46	2.7E-44	1.8E-41	ENSRNO	protein_coding
Nxph1	-7.0712	-134.48	2.5E-08	1.3E-07	0.00047	ENSRNO	protein_coding
Thsd7a	-5.8312	-56.934	1.5E-51	4.9E-50	2.9E-47	ENSRNO	protein_coding
Foxp2	4.35861	20.515	8.5E-86	4.9E-84	1.6E-81	ENSRNO	protein_coding
Cttnbp2	-2.8335	-7.1278	2.2E-64	9.1E-63	4.1E-60	ENSRNO	protein_coding
Tfec	2.39594	5.26321	8E-37	1.8E-35	1.5E-32	ENSRNO	protein_coding
Cav2	3.55645	11.7652	4E-149	5E-147	7E-145	ENSRNO	protein_coding
Asb15	-3.7334	-13.301	1.3E-73	6.4E-72	2.5E-69	ENSRNO	protein_coding
Prrt4	-2.3136	-4.9711	0.00018	0.00057	1	ENSRNO	protein_coding
Impdh1	-1.587	-3.0042	3.5E-53	1.2E-51	6.7E-49	ENSRNO	protein_coding
Fam71f1	-3.5868	-12.015	1.6E-25	2.5E-24	3.1E-21	ENSRNO	protein_coding
Ccdc136	-1.7872	-3.4513	1.3E-28	2.3E-27	2.5E-24	ENSRNO	protein_coding
Tspan33	-3.4806	-11.162	1.1E-12	8.6E-12	2.1E-08	ENSRNO	protein_coding
Smo	-4.7441	-26.798	2E-282	1E-279	5E-278	ENSRNO	protein_coding
Strip2	-1.3432	-2.5371	0.00012	0.00041	1	ENSRNO	protein_coding
Mest	2.6411	6.23808	4.6E-55	1.6E-53	8.7E-51	ENSRNO	protein_coding
Podxl	2.83833	7.15192	0.01201	0.02647	1	ENSRNO	protein_coding
Plxna4	6.07621	67.4716	0.01091	0.02429	1	ENSRNO	protein_coding
Akr1b10	2.60059	6.06534	3E-145	4E-143	5E-141	ENSRNO	protein_coding
LOC1009	2.58223	5.98863	2.1E-47	6.3E-46	4E-43	ENSRNO	protein_coding
Cald1	-2.0495	-4.1396	1.1E-92	7.1E-91	2E-88	ENSRNO	protein_coding
Agbl3	1.53822	2.90436	4.5E-07	2.1E-06	0.00855	ENSRNO	protein_coding
Fam180a	-2.0323	-4.0906	5.8E-08	3E-07	0.0011	ENSRNO	protein_coding
Chrm2	6.45854	87.9458	0.00684	0.01607	1	ENSRNO	protein_coding
Creb3l2	-1.4792	-2.7878	2.8E-30	5.2E-29	5.3E-26	ENSRNO	protein_coding
Klrg2	2.41588	5.33646	1.2E-07	5.8E-07	0.00223	ENSRNO	protein_coding
Dennd2a	-2.5513	-5.8617	7.7E-16	7.4E-15	1.5E-11	ENSRNO	protein_coding
Tmem178	1.43877	2.7109	0.00231	0.00601	1	ENSRNO	protein_coding
Chl1	2.33399	5.04198	0.02398	0.04871	1	ENSRNO	protein_coding
Gstk1	-5.7228	-52.812	4.4E-14	3.8E-13	8.3E-10	ENSRNO	protein_coding
Tmem139	-2.7197	-6.5872	2.6E-06	1.1E-05	0.04942	ENSRNO	protein_coding
Fam131b	-2.7013	-6.5037	1.8E-10	1.2E-09	3.5E-06	ENSRNO	protein_coding
Rn50_4_1	-2.6926	-6.4647	0.00011	0.00036	1	ENSRNO	unprocessed_pseudogene
Zfp862	-1.4156	-2.6676	1.8E-13	1.5E-12	3.4E-09	ENSRNO	protein_coding
Gimap6	2.60853	6.09882	9.1E-37	2.1E-35	1.7E-32	ENSRNO	protein_coding
Gimap5	3.46474	11.0405	8.9E-21	1.1E-19	1.7E-16	ENSRNO	protein_coding
Tmem176	-3.6808	-12.825	6.7E-05	0.00023	1	ENSRNO	protein_coding
Igf2bp3	-1.7184	-3.2906	8.7E-57	3.2E-55	1.7E-52	ENSRNO	protein_coding

Fam221a	-6.656	-100.85	1.6E-15	1.5E-14	3E-11	ENSRNO	protein_coding
LOC1009	8.38651	334.65	0.0004	0.00121	1	ENSRNO	protein_coding
Snx10	2.46673	5.52788	5.5E-96	4E-94	1.1E-91	ENSRNO	protein_coding
Hoxa7	-2.615	-6.126	6.5E-05	0.00023	1	ENSRNO	protein_coding
Hoxa11-a	1.41154	2.6602	5.2E-13	4.2E-12	9.9E-09	ENSRNO	protein_coding
Jazf1	-2.1315	-4.3816	6.4E-12	4.7E-11	1.2E-07	ENSRNO	protein_coding
Creb5	1.87286	3.66258	2.2E-05	8.2E-05	0.42059	ENSRNO	protein_coding
Chn2	-3.0471	-8.2658	7.8E-29	1.4E-27	1.5E-24	ENSRNO	protein_coding
Wipf3	-3.1813	-9.0714	7E-13	5.5E-12	1.3E-08	ENSRNO	protein_coding
Fkbp14	-1.4639	-2.7586	6.8E-20	8.1E-19	1.3E-15	ENSRNO	protein_coding
AABR070	7.48622	179.298	0.00235	0.00611	1	ENSRNO	pseudogene
Aqp1	-1.3611	-2.5688	5.1E-38	1.2E-36	9.6E-34	ENSRNO	protein_coding
Neurod6	-5.283	-38.936	4.2E-18	4.6E-17	8E-14	ENSRNO	protein_coding
Pde1c	5.6416	49.9219	1.4E-05	5.2E-05	0.2595	ENSRNO	protein_coding
Fkbp9	-2.4026	-5.2877	2E-122	2E-120	3E-118	ENSRNO	protein_coding
Ppm1k	-1.4416	-2.7163	3.9E-13	3.2E-12	7.4E-09	ENSRNO	protein_coding
Gprin3	-1.5519	-2.9319	2.3E-18	2.5E-17	4.3E-14	ENSRNO	protein_coding
Ndnf	-4.9045	-29.95	9.1E-10	5.6E-09	1.7E-05	ENSRNO	protein_coding
Prdm5	-5.0817	-33.865	3.3E-86	1.9E-84	6.3E-82	ENSRNO	protein_coding
Thnsl2	-5.6719	-50.981	1.5E-05	5.7E-05	0.28696	ENSRNO	protein_coding
Reep1	1.83021	3.55588	0.00865	0.01977	1	ENSRNO	protein_coding
Tmem150	2.17979	4.53086	2.4E-36	5.3E-35	4.6E-32	ENSRNO	protein_coding
Tcf7l1	-2.5476	-5.8466	2.2E-44	6.1E-43	4.2E-40	ENSRNO	protein_coding
AABR070	5.81079	56.1337	0.01741	0.03673	1	ENSRNO	lincRNA
Dnah6	2.80194	6.97376	1.2E-35	2.5E-34	2.2E-31	ENSRNO	protein_coding
Eva1a	-3.755	-13.501	1.8E-24	2.7E-23	3.4E-20	ENSRNO	protein_coding
Sema4f	-4.2152	-18.574	4.8E-06	2E-05	0.09127	ENSRNO	protein_coding
LOC1036	-1.9876	-3.9659	1.9E-06	8.2E-06	0.03645	ENSRNO	protein_coding
Wdr54	-11.053	-2125.1	4E-06	1.7E-05	0.07646	ENSRNO	protein_coding
LOC5002	-3.139	-8.8088	0.00011	0.00036	1	ENSRNO	protein_coding
Cd207	-5.1256	-34.911	8.1E-09	4.6E-08	0.00015	ENSRNO	protein_coding
Atp6v1b1	1.40942	2.65631	3.3E-05	0.00012	0.6297	ENSRNO	protein_coding
Dysf	-1.977	-3.9366	1E-83	5.7E-82	1.9E-79	ENSRNO	protein_coding
LOC1036	6.65901	101.056	0.0095	0.02146	1	ENSRNO	protein_coding
Tprkb	-1.8147	-3.5179	0.00786	0.01816	1	ENSRNO	protein_coding
H1fx	-1.755	-3.3753	2.3E-09	1.4E-08	4.4E-05	ENSRNO	protein_coding
AABR070	-5.6507	-50.237	1.6E-05	6.2E-05	0.31047	ENSRNO	lincRNA
Gata2	-2.301	-4.9281	4.1E-32	8.2E-31	7.8E-28	ENSRNO	protein_coding
Podxl2	1.40618	2.65034	0.00142	0.00385	1	ENSRNO	protein_coding
Vom1r90	5.76711	54.4596	0.01778	0.0374	1	ENSRNO	protein_coding
Cfap100	-3.4117	-10.642	1.2E-23	1.6E-22	2.2E-19	ENSRNO	protein_coding
Nup210	-6.4213	-85.703	5E-161	8E-159	1E-156	ENSRNO	protein_coding
Fbln2	-2.8842	-7.3831	3.4E-09	2E-08	6.4E-05	ENSRNO	protein_coding
Wnt7a	4.32843	20.0904	5.1E-05	0.00018	0.96328	ENSRNO	protein_coding
Slc41a3	-3.3533	-10.22	1.8E-62	7.4E-61	3.5E-58	ENSRNO	protein_coding
Aldh1l1	2.93359	7.64008	1.8E-07	8.8E-07	0.00344	ENSRNO	protein_coding
Grip2	6.07623	67.4725	0.01113	0.02472	1	ENSRNO	protein_coding
Fgd5	4.64926	25.0938	0.00046	0.00137	1	ENSRNO	protein_coding
Prickle2	-3.6669	-12.701	3E-44	8.1E-43	5.6E-40	ENSRNO	protein_coding

Gxylt2	-7.6529	-201.26	0.00158	0.00425	1	ENSRNO	protein_coding
Itpr1	-1.9075	-3.7515	6.4E-78	3.3E-76	1.2E-73	ENSRNO	protein_coding
Cav3	-4.0868	-16.992	1.2E-07	6.2E-07	0.00235	ENSRNO	protein_coding
Srgap3	-1.3807	-2.6039	5.8E-25	8.8E-24	1.1E-20	ENSRNO	protein_coding
Il17re	-1.9148	-3.7705	1.2E-28	2.1E-27	2.2E-24	ENSRNO	protein_coding
Creld1	1.56661	2.96208	9.3E-17	9.5E-16	1.8E-12	ENSRNO	protein_coding
Slc6a1	1.66767	3.17701	0.01925	0.0401	1	ENSRNO	protein_coding
Hrh1	-2.9843	-7.9132	0.00777	0.01798	1	ENSRNO	protein_coding
Pparg	1.52141	2.87071	1.4E-16	1.4E-15	2.6E-12	ENSRNO	protein_coding
Cand2	-7.0115	-129.03	1.9E-17	2E-16	3.7E-13	ENSRNO	protein_coding
Efcab12	1.7597	3.38627	0.00858	0.01963	1	ENSRNO	protein_coding
Plxnd1	-1.498	-2.8244	4.6E-14	4E-13	8.8E-10	ENSRNO	protein_coding
Rassf4	4.34567	20.3319	2.2E-05	8.1E-05	0.4173	ENSRNO	protein_coding
Cxcl12	-6.8975	-119.22	0	0	0	ENSRNO	protein_coding
Zfp637	-1.4234	-2.6823	1E-36	2.3E-35	2E-32	ENSRNO	protein_coding
Zfp9	-3.4096	-10.626	1.6E-23	2.3E-22	3E-19	ENSRNO	protein_coding
Cacna1c	-6.1522	-71.12	1E-115	1E-113	2E-111	ENSRNO	protein_coding
Dcp1b	-8.583	-383.47	1.2E-11	8.8E-11	2.3E-07	ENSRNO	protein_coding
Wnt5b	-5.4573	-43.934	3.1E-46	9.1E-45	5.9E-42	ENSRNO	protein_coding
AABR070	-2.5522	-5.8652	0.00866	0.01978	1	ENSRNO	lincRNA
Cecr2	3.13327	8.77424	0.02297	0.04687	1	ENSRNO	protein_coding
Usp18	1.5092	2.84652	1.8E-30	3.3E-29	3.4E-26	ENSRNO	protein_coding
Iqsec3	10.4229	1372.82	8.9E-06	3.5E-05	0.16862	ENSRNO	protein_coding
A2m	-4.2897	-19.558	9.5E-13	7.5E-12	1.8E-08	ENSRNO	protein_coding
LOC1009	-4.1432	-17.669	1.3E-19	1.6E-18	2.6E-15	ENSRNO	protein_coding
Mfap5	-2.9036	-7.4827	0.00956	0.02159	1	ENSRNO	protein_coding
Slc2a3	-3.1716	-9.0104	5E-196	1E-193	9E-192	ENSRNO	protein_coding
Eno2	2.1197	4.34603	1.2E-84	6.7E-83	2.2E-80	ENSRNO	protein_coding
P3h3	-4.1669	-17.962	6E-234	2E-231	1E-229	ENSRNO	protein_coding
AC11542(	-4.118	-17.363	7.6E-06	3E-05	0.14393	ENSRNO	protein_coding
Gpr162	-2.7368	-6.6659	5.7E-12	4.3E-11	1.1E-07	ENSRNO	protein_coding
Pianp	-3.2197	-9.3161	2.3E-51	7.4E-50	4.4E-47	ENSRNO	protein_coding
Scnn1a	-2.2385	-4.7191	0.00017	0.00055	1	ENSRNO	protein_coding
Cd9	-2.8019	-6.9738	8E-157	1E-154	1E-152	ENSRNO	protein_coding
Ano2	-9.6337	-794.36	3.5E-24	5.1E-23	6.7E-20	ENSRNO	protein_coding
Kcna6	-6.2146	-74.265	6E-185	1E-182	1E-180	ENSRNO	protein_coding
AABR070	-4.4498	-21.854	0.00114	0.00315	1	ENSRNO	lincRNA
Ccnd2	-4.4566	-21.957	2E-153	2E-151	3E-149	ENSRNO	protein_coding
Cracr2a	6.94285	123.029	0.00361	0.00903	1	ENSRNO	protein_coding
AABR070	4.32481	20.04	0.00138	0.00376	1	ENSRNO	lincRNA
Tspan9	-1.4624	-2.7556	4.1E-41	1E-39	7.7E-37	ENSRNO	protein_coding
Tead4	-2.3072	-4.9492	4E-101	3E-99	8.3E-97	ENSRNO	protein_coding
LOC6897	3.41496	10.6661	2.5E-25	3.9E-24	4.8E-21	ENSRNO	protein_coding
Klra5_1	5.64221	49.943	0.02263	0.04633	1	ENSRNO	protein_coding
Clec12a	4.22237	18.6664	8.6E-09	4.8E-08	0.00016	ENSRNO	protein_coding
Olr1	3.24627	9.48909	9.1E-08	4.6E-07	0.00174	ENSRNO	protein_coding
Klrc3	6.91274	120.487	0.00378	0.00941	1	ENSRNO	protein_coding
Ly49i5	9.87272	937.529	2.6E-05	9.5E-05	0.49351	ENSRNO	protein_coding
Ly49s6	4.85061	28.8523	6.3E-12	4.7E-11	1.2E-07	ENSRNO	protein_coding

Ly49s4	6.53225	92.5559	0.00644	0.01522	1	ENSRNO	protein_coding
Ly49i2	6.94041	122.821	2E-20	2.4E-19	3.8E-16	ENSRNO	protein_coding
Klra2	3.66312	12.668	3.3E-07	1.6E-06	0.00629	ENSRNO	protein_coding
Styk1	1.83084	3.55744	1.4E-09	8.4E-09	2.6E-05	ENSRNO	protein_coding
Etv6	-1.5416	-2.9111	1.6E-21	2.1E-20	3E-17	ENSRNO	protein_coding
Bcl2l14	-2.391	-5.2452	0.0003	0.00093	1	ENSRNO	protein_coding
Gprc5d	-2.6303	-6.1914	0.01907	0.0398	1	ENSRNO	protein_coding
Gucy2c	-2.4758	-5.5627	0.01401	0.03041	1	ENSRNO	protein_coding
Mgp	-1.502	-2.8324	1.2E-45	3.4E-44	2.3E-41	ENSRNO	protein_coding
Arhgdib	5.80806	56.0275	1.3E-54	4.4E-53	2.5E-50	ENSRNO	protein_coding
Rerg	4.64938	25.0959	0.0005	0.00149	1	ENSRNO	protein_coding
Eps8	-2.1533	-4.4483	6.7E-94	4.6E-92	1.3E-89	ENSRNO	protein_coding
Mgst1	-1.7499	-3.3634	2.6E-15	2.4E-14	5E-11	ENSRNO	protein_coding
Lmo3	3.13523	8.78613	8.5E-12	6.2E-11	1.6E-07	ENSRNO	protein_coding
Abcc9	-8.7424	-428.27	2E-156	3E-154	3E-152	ENSRNO	protein_coding
Sox5	4.62231	24.6295	7.9E-18	8.6E-17	1.5E-13	ENSRNO	protein_coding
AABR070	3.15745	8.92253	1.5E-06	6.7E-06	0.02919	ENSRNO	lincRNA
Bhlhe41	1.35984	2.56657	2.1E-11	1.5E-10	4E-07	ENSRNO	protein_coding
Smco2	-7.7704	-218.33	0.00136	0.00369	1	ENSRNO	protein_coding
Rps4y2	-7.6636	-202.75	1.8E-87	1.1E-85	3.4E-83	ENSRNO	protein_coding
Caprin2	-1.7339	-3.3262	3.4E-26	5.5E-25	6.6E-22	ENSRNO	protein_coding
Trpa1	3.10114	8.58095	4.2E-13	3.4E-12	8E-09	ENSRNO	protein_coding
Eya1	-1.7676	-3.4048	7.3E-33	1.5E-31	1.4E-28	ENSRNO	protein_coding
Sulf1	1.99171	3.97707	8.7E-77	4.4E-75	1.7E-72	ENSRNO	protein_coding
Adhfe1	1.33169	2.51697	0.00178	0.00474	1	ENSRNO	protein_coding
Rgs20	6.43807	86.7066	2.2E-21	2.8E-20	4.2E-17	ENSRNO	protein_coding
Rp1	2.03565	4.10007	0.0208	0.04295	1	ENSRNO	protein_coding
Plag1	1.57622	2.98187	2.6E-07	1.3E-06	0.00501	ENSRNO	protein_coding
Tox	5.52892	46.1712	7.4E-10	4.6E-09	1.4E-05	ENSRNO	protein_coding
Car8	4.84772	28.7946	1.7E-07	8.3E-07	0.00325	ENSRNO	protein_coding
Chd7	-2.2911	-4.8944	4.6E-11	3.2E-10	8.8E-07	ENSRNO	protein_coding
Gdf6	1.90803	3.75297	0.00129	0.00353	1	ENSRNO	protein_coding
Gem	6.92924	121.874	1E-20	1.3E-19	2E-16	ENSRNO	protein_coding
Cdh17	-3.0795	-8.4535	2.3E-38	5.4E-37	4.3E-34	ENSRNO	protein_coding
Slc26a7	-2.2977	-4.9168	0.0007	0.00201	1	ENSRNO	protein_coding
LOC1009	7.52903	184.698	3.1E-46	9.1E-45	5.9E-42	ENSRNO	protein_coding
Necab1	5.5985	48.4526	1.1E-13	9.5E-13	2.1E-09	ENSRNO	protein_coding
Mmp16	1.39545	2.6307	1E-23	1.5E-22	2E-19	ENSRNO	protein_coding
Ttpa	4.38918	20.9543	1.2E-06	5.4E-06	0.02317	ENSRNO	protein_coding
Klhl32	4.94759	30.8583	2.1E-08	1.1E-07	0.0004	ENSRNO	protein_coding
Gpr63	5.91664	60.4067	3.8E-12	2.9E-11	7.2E-08	ENSRNO	protein_coding
AABR070	2.82151	7.06904	0.00352	0.00884	1	ENSRNO	pseudogene
Bach2	-2.5208	-5.7391	9E-08	4.5E-07	0.00171	ENSRNO	protein_coding
Ankrd6	2.4395	5.42455	1.5E-28	2.7E-27	2.9E-24	ENSRNO	protein_coding
RGD1563	2.17511	4.51619	0.00421	0.0104	1	ENSRNO	protein_coding
RGD1359	1.49691	2.82238	3.7E-14	3.2E-13	7E-10	ENSRNO	protein_coding
Ddx58	1.82442	3.54165	1.7E-12	1.3E-11	3.2E-08	ENSRNO	protein_coding
Fam219a	1.47523	2.78027	9.3E-31	1.8E-29	1.8E-26	ENSRNO	protein_coding
Phf24	-6.2483	-76.017	1.1E-22	1.5E-21	2.1E-18	ENSRNO	protein_coding

Unc13b	3.65705	12.6148	1.6E-24	2.3E-23	3E-20	ENSRNO	protein_coding
Atp8b5p	2.70372	6.51478	0.00024	0.00075	1	ENSRNO	protein_coding
Sit1	3.39933	10.5511	0.00022	0.0007	1	ENSRNO	protein_coding
Npr2	-5.2534	-38.145	8E-120	7E-118	1E-115	ENSRNO	protein_coding
Reck	1.62852	3.09196	1.5E-51	4.8E-50	2.8E-47	ENSRNO	protein_coding
Fbxo10	-1.6147	-3.0625	3.2E-12	2.4E-11	6.1E-08	ENSRNO	protein_coding
Frmpd1	3.681	12.826	1E-49	3.2E-48	1.9E-45	ENSRNO	protein_coding
Igfbpl1	-3.2575	-9.5634	2.3E-05	8.4E-05	0.43148	ENSRNO	protein_coding
RGD1305	5.83883	57.2351	0.01472	0.03171	1	ENSRNO	protein_coding
Tmod1	2.69754	6.48696	3.2E-52	1E-50	6E-48	ENSRNO	protein_coding
Trim14	1.38156	2.6055	1.8E-05	6.8E-05	0.34264	ENSRNO	protein_coding
Coro2a	-1.6961	-3.2403	7E-06	2.8E-05	0.13266	ENSRNO	protein_coding
Tbc1d2	-1.3405	-2.5325	4.1E-37	9.4E-36	7.8E-33	ENSRNO	protein_coding
Galnt12	1.87567	3.66973	3.3E-08	1.8E-07	0.00063	ENSRNO	protein_coding
Murc	-8.5886	-384.96	8.4E-64	3.5E-62	1.6E-59	ENSRNO	protein_coding
Baat	4.48796	22.4393	0.00093	0.00263	1	ENSRNO	protein_coding
Aldob	6.8593	116.106	0.0039	0.00968	1	ENSRNO	protein_coding
Slc44a1	1.5439	2.91581	5.5E-52	1.8E-50	1E-47	ENSRNO	protein_coding
Tal2	5.56468	47.3299	0.02163	0.04446	1	ENSRNO	protein_coding
Epb41l4b	4.00444	16.0494	5.4E-12	4E-11	1E-07	ENSRNO	protein_coding
Palm2	-4.4233	-21.456	5.9E-29	1E-27	1.1E-24	ENSRNO	protein_coding
ENSRNO	-4.1624	-17.907	3.7E-28	6.3E-27	7E-24	ENSRNO	protein_coding
Akap2	-2.4393	-5.4239	4E-110	3E-108	7E-106	ENSRNO	protein_coding
Svep1	-9.2126	-593.28	0.00013	0.00041	1	ENSRNO	protein_coding
Lpar1	-3.1493	-8.8725	9E-166	1E-163	2E-161	ENSRNO	protein_coding
Zfp483	-7.3833	-166.95	0.00256	0.00661	1	ENSRNO	protein_coding
Susd1	1.8953	3.71999	0.00042	0.00125	1	ENSRNO	protein_coding
Zfp37_1	-5.187	-36.427	4.5E-09	2.6E-08	8.6E-05	ENSRNO	protein_coding
Rn50_5_(	-8.1389	-281.88	0.00078	0.00222	1	ENSRNO	unprocessed_pseudogene
Rn50_5_(	-6.5789	-95.595	0.00686	0.01611	1	ENSRNO	unprocessed_pseudogene
Zfp37_2	-5.8936	-59.451	0.01593	0.03395	1	ENSRNO	protein_coding
Zfp618	-3.0422	-8.2373	2E-65	8.5E-64	3.7E-61	ENSRNO	protein_coding
Orm1	-1.7997	-3.4816	0.00099	0.00279	1	ENSRNO	protein_coding
AC22994!	1.60882	3.05003	0.01188	0.02622	1	ENSRNO	protein_coding
Tlr4	5.25726	38.2467	5E-142	6E-140	1E-137	ENSRNO	protein_coding
AABR070	2.96477	7.807	1.2E-08	6.5E-08	0.00022	ENSRNO	pseudogene
AABR070	3.62321	12.3224	0.00777	0.01799	1	ENSRNO	pseudogene
Tyrp1	2.9551	7.75484	0.00027	0.00083	1	ENSRNO	protein_coding
Hspa8_1	2.64745	6.26557	9.4E-13	7.4E-12	1.8E-08	ENSRNO	protein_coding
Frem1	-1.8646	-3.6417	2.9E-21	3.8E-20	5.6E-17	ENSRNO	protein_coding
AABR070	6.18548	72.7804	4.5E-10	2.9E-09	8.5E-06	ENSRNO	protein_coding
Cntln	7.03151	130.826	1.5E-43	4.1E-42	2.9E-39	ENSRNO	protein_coding
Adamtsl1	-5.3525	-40.857	1.6E-31	3.1E-30	3E-27	ENSRNO	protein_coding
Hacd4	-2.6587	-6.3148	1.5E-16	1.5E-15	2.8E-12	ENSRNO	protein_coding
AABR070	6.71796	105.271	1.3E-07	6.3E-07	0.00239	ENSRNO	protein_coding
Elavl2	2.5314	5.78133	1E-37	2.4E-36	2E-33	ENSRNO	protein_coding
Tek	-2.6213	-6.153	3.1E-17	3.3E-16	6E-13	ENSRNO	protein_coding
Eqtn	3.15485	8.90647	3.5E-08	1.8E-07	0.00066	ENSRNO	protein_coding
LOC1009	5.07882	33.797	1.8E-28	3.2E-27	3.5E-24	ENSRNO	protein_coding

Kank4	-2.1803	-4.5326	2.6E-05	9.6E-05	0.49942	ENSRNO	protein_coding
LOC1003	2.66688	6.35054	0.01576	0.03366	1	ENSRNO	protein_coding
Raver2	-6.21	-74.027	3E-25	4.6E-24	5.7E-21	ENSRNO	protein_coding
Dnajc6	-4.2828	-19.465	2E-152	3E-150	4E-148	ENSRNO	protein_coding
RGD1562	3.78042	13.741	0.00527	0.01274	1	ENSRNO	protein_coding
Lexm	-1.6028	-3.0373	0.00037	0.00112	1	ENSRNO	protein_coding
Ttc22	-4.109	-17.256	3.8E-17	4E-16	7.2E-13	ENSRNO	protein_coding
Fam151a	-3.9014	-14.943	1.7E-05	6.3E-05	0.3163	ENSRNO	protein_coding
Acot11	-3.4043	-10.587	4.6E-05	0.00016	0.88407	ENSRNO	protein_coding
Ldlrad1	1.86937	3.65372	0.00185	0.00491	1	ENSRNO	protein_coding
Glis1	3.84959	14.416	2.9E-06	1.2E-05	0.05545	ENSRNO	protein_coding
Zyg11a	2.26563	4.80863	0.01447	0.03127	1	ENSRNO	protein_coding
Rab3b	-2.9673	-7.8207	4.9E-53	1.6E-51	9.3E-49	ENSRNO	protein_coding
AABR070	-1.4757	-2.7812	0.00014	0.00046	1	ENSRNO	pseudogene
Elavl4	5.51408	45.6987	1.2E-27	2E-26	2.3E-23	ENSRNO	protein_coding
Bend5	8.0976	273.919	0.00062	0.0018	1	ENSRNO	protein_coding
Cyp4b1	2.86864	7.30379	1.7E-05	6.5E-05	0.32874	ENSRNO	protein_coding
Lurap1	-1.6613	-3.1631	4E-06	1.6E-05	0.07578	ENSRNO	protein_coding
Zswim5	-7.4975	-180.7	3.7E-09	2.2E-08	7.1E-05	ENSRNO	protein_coding
Tmem53	-2.4221	-5.3596	5.8E-13	4.6E-12	1.1E-08	ENSRNO	protein_coding
Ccdc24	5.90201	59.7974	0.01376	0.02991	1	ENSRNO	protein_coding
B4galt2	6.93332	122.218	1.4E-54	4.8E-53	2.7E-50	ENSRNO	protein_coding
Artn	-2.794	-6.9354	2.6E-05	9.6E-05	0.49923	ENSRNO	protein_coding
Ccdc30	2.55554	5.87887	0.00483	0.01176	1	ENSRNO	protein_coding
Scmh1	1.35071	2.55038	3.3E-38	7.8E-37	6.3E-34	ENSRNO	protein_coding
Slfnl1	2.33466	5.04432	3.1E-25	4.8E-24	6E-21	ENSRNO	protein_coding
Mfsd2a	-5.6355	-49.712	4.7E-08	2.4E-07	0.00089	ENSRNO	protein_coding
LOC1036	1.64354	3.12433	8.5E-07	3.8E-06	0.01623	ENSRNO	lincRNA
Epha10	-2.6197	-6.1463	0.00099	0.00277	1	ENSRNO	protein_coding
Rspo1	-3.0554	-8.3134	4.1E-19	4.7E-18	7.8E-15	ENSRNO	protein_coding
Col8a2	-6.2896	-78.229	4E-250	1E-247	7E-246	ENSRNO	protein_coding
Tekt2	3.65095	12.5616	0.00078	0.00223	1	ENSRNO	protein_coding
Ago1	-3.2259	-9.3564	3.6E-22	4.8E-21	6.8E-18	ENSRNO	protein_coding
RGD1563	-1.5931	-3.0169	0.00015	0.00049	1	ENSRNO	protein_coding
Gja4	1.65831	3.15647	2.4E-22	3.2E-21	4.6E-18	ENSRNO	protein_coding
AABR070	-1.9108	-3.7601	0.00101	0.00283	1	ENSRNO	lincRNA
Fndc5	-4.5002	-22.63	0.00096	0.00271	1	ENSRNO	protein_coding
Sync	-2.8469	-7.1944	1.1E-26	1.8E-25	2.1E-22	ENSRNO	protein_coding
LOC1003	1.49831	2.82512	0.00858	0.01963	1	ENSRNO	protein_coding
Marcksl1	-4.0168	-16.188	9.3E-39	2.2E-37	1.8E-34	ENSRNO	protein_coding
Ccdc28b	1.92776	3.80463	0.00352	0.00884	1	ENSRNO	protein_coding
Adgrb2	5.77295	54.6802	0.01602	0.0341	1	ENSRNO	protein_coding
Tinagl1	2.50259	5.66701	6E-44	1.7E-42	1.1E-39	ENSRNO	protein_coding
Serinc2	2.7099	6.54276	9E-64	3.7E-62	1.7E-59	ENSRNO	protein_coding
Fabp3	1.67068	3.18365	3.6E-24	5.2E-23	6.8E-20	ENSRNO	protein_coding
Sdc3	-5.0902	-34.065	0	0	0	ENSRNO	protein_coding
Laptm5	6.42422	85.8782	0.00734	0.01712	1	ENSRNO	protein_coding
Sesn2	1.36816	2.58142	7.5E-22	9.9E-21	1.4E-17	ENSRNO	protein_coding
Ptafr	-3.9699	-15.67	2.6E-15	2.4E-14	4.9E-11	ENSRNO	protein_coding

Themis2	-1.9339	-3.8208	0.00051	0.0015	1	ENSRNO	protein_coding
Fam46b	-2.0045	-4.0125	1.2E-29	2.2E-28	2.3E-25	ENSRNO	protein_coding
Trnp1	-1.9848	-3.9581	0.0064	0.01515	1	ENSRNO	protein_coding
Sfn	3.30326	9.87142	4.2E-07	2E-06	0.00797	ENSRNO	protein_coding
Runx3	-6.3496	-81.549	9.4E-07	4.2E-06	0.01795	ENSRNO	protein_coding
Nipal3	3.10496	8.60369	3.2E-45	9.1E-44	6.1E-41	ENSRNO	protein_coding
Ifnlr1	1.51778	2.86351	2.3E-14	2.1E-13	4.4E-10	ENSRNO	protein_coding
Il22ra1	3.69139	12.9187	0.00052	0.00154	1	ENSRNO	protein_coding
Tcea3	-1.3664	-2.5782	4.4E-07	2.1E-06	0.00842	ENSRNO	protein_coding
Ephb2	4.57877	23.8971	1.6E-29	2.8E-28	3E-25	ENSRNO	protein_coding
Epha8	4.56531	23.6753	0.00063	0.00183	1	ENSRNO	protein_coding
Wnt4	-2.1827	-4.5402	1.6E-87	9.4E-86	3E-83	ENSRNO	protein_coding
AABR070	-1.4118	-2.6608	9.9E-45	2.8E-43	1.9E-40	ENSRNO	protein_coding
Rap1gap	-2.3316	-5.0335	2.4E-13	1.9E-12	4.5E-09	ENSRNO	protein_coding
Alpl	-4.2637	-19.208	1.5E-22	2E-21	2.8E-18	ENSRNO	protein_coding
LOC1009	3.13581	8.78965	5.1E-19	5.9E-18	9.8E-15	ENSRNO	protein_coding
Fam43b	-4.7683	-27.251	0.00047	0.0014	1	ENSRNO	protein_coding
Camk2n1	2.24091	4.72695	0.00097	0.00272	1	ENSRNO	protein_coding
Vwa5b1	7.01002	128.893	0.00311	0.0079	1	ENSRNO	protein_coding
Pla2g5	-3.437	-10.83	3.9E-26	6.2E-25	7.4E-22	ENSRNO	protein_coding
Padi2	2.87446	7.33326	0.00039	0.00116	1	ENSRNO	protein_coding
Rsg1	-1.5414	-2.9108	1.3E-10	9E-10	2.6E-06	ENSRNO	protein_coding
Clcnkb	3.22311	9.33798	3.6E-13	2.9E-12	6.8E-09	ENSRNO	protein_coding
Hspb7	-2.4867	-5.6051	8.2E-14	7E-13	1.6E-09	ENSRNO	protein_coding
Agmat	-2.4542	-5.4799	0.00021	0.00068	1	ENSRNO	protein_coding
Ctrc	3.78057	13.7425	3.5E-05	0.00012	0.65929	ENSRNO	protein_coding
Fhad1	1.53362	2.89511	0.01005	0.0226	1	ENSRNO	protein_coding
Pdpn	-6.2286	-74.987	4.4E-25	6.8E-24	8.5E-21	ENSRNO	protein_coding
Dhrs3	-2.261	-4.7933	5.8E-09	3.3E-08	0.00011	ENSRNO	protein_coding
Tnfrsf1b	2.07417	4.21101	2.2E-51	7.1E-50	4.2E-47	ENSRNO	protein_coding
Draxin	-4.8029	-27.915	9.1E-60	3.5E-58	1.7E-55	ENSRNO	protein_coding
Fbxo44	-3.2599	-9.5791	1.1E-16	1.1E-15	2.2E-12	ENSRNO	protein_coding
Fbxo2	-3.5621	-11.812	4.8E-24	7E-23	9.2E-20	ENSRNO	protein_coding
LOC1025	-1.3992	-2.6376	0.01646	0.03492	1	ENSRNO	protein_coding
Pik3cd	-3.3891	-10.477	5E-182	1E-179	1E-177	ENSRNO	protein_coding
AABR070	-3.5138	-11.422	6.9E-07	3.1E-06	0.01307	ENSRNO	lincRNA
Slc2a5	3.91353	15.0692	1.8E-52	5.8E-51	3.3E-48	ENSRNO	protein_coding
Plekhg5	-1.3608	-2.5683	3.3E-36	7.3E-35	6.3E-32	ENSRNO	protein_coding
Tnfrsf25	-1.9199	-3.784	1.5E-06	6.7E-06	0.02943	ENSRNO	protein_coding
Gpr153	-4.7963	-27.787	1.6E-11	1.1E-10	3E-07	ENSRNO	protein_coding
Hes3	3.78035	13.7404	0.00544	0.0131	1	ENSRNO	protein_coding
Rnf207	-1.6278	-3.0904	0.02115	0.0436	1	ENSRNO	protein_coding
Chd5	2.44808	5.45688	0.00413	0.01022	1	ENSRNO	protein_coding
Kcnab2	-1.6994	-3.2476	1.2E-24	1.8E-23	2.3E-20	ENSRNO	protein_coding
LOC1009	3.77599	13.6989	0.0064	0.01515	1	ENSRNO	protein_coding
Arhgef16	5.47396	44.4453	0.02294	0.04683	1	ENSRNO	protein_coding
Prkcz	-2.2304	-4.6927	1.7E-09	1E-08	3.2E-05	ENSRNO	protein_coding
Vwa1	-7.0988	-137.07	1E-124	1E-122	3E-120	ENSRNO	protein_coding
Crim1	-1.5263	-2.8805	7.6E-52	2.5E-50	1.4E-47	ENSRNO	protein_coding

LOC1036	-5.1422	-35.314	3.1E-11	2.2E-10	5.8E-07	ENSRNO	protein_coding
Qpct	-2.8515	-7.2173	2.8E-10	1.8E-09	5.4E-06	ENSRNO	protein_coding
Cyp1b1	-4.3424	-20.286	0	0	0	ENSRNO	protein_coding
Galm	-7.1666	-143.67	2E-134	2E-132	4E-130	ENSRNO	protein_coding
Map4k3	-2.2169	-4.6489	6.9E-96	4.9E-94	1.3E-91	ENSRNO	protein_coding
Slc8a1	-6.643	-99.943	5E-227	1E-224	9E-223	ENSRNO	protein_coding
Pkdcc	-2.8126	-7.0256	6.7E-18	7.3E-17	1.3E-13	ENSRNO	protein_coding
Dync2li1	-3.0556	-8.3141	2.9E-44	8.1E-43	5.6E-40	ENSRNO	protein_coding
AABR070	-1.8174	-3.5244	0.0008	0.00228	1	ENSRNO	protein_coding
Ston1	-1.478	-2.7856	6.6E-45	1.9E-43	1.3E-40	ENSRNO	protein_coding
Alk	5.90643	59.9808	3.1E-12	2.4E-11	5.9E-08	ENSRNO	protein_coding
Lbh	-1.6411	-3.119	1.7E-08	9.1E-08	0.00032	ENSRNO	protein_coding
Ehd3	-1.3231	-2.502	6E-35	1.3E-33	1.1E-30	ENSRNO	protein_coding
Fosl2	-1.5474	-2.9228	1.1E-16	1.1E-15	2E-12	ENSRNO	protein_coding
Fndc4	-5.1259	-34.919	2E-96	1.4E-94	3.7E-92	ENSRNO	protein_coding
Slc30a3	-7.1012	-137.3	0.00346	0.00871	1	ENSRNO	protein_coding
Cgref1	-6.7389	-106.81	2E-89	1.2E-87	3.8E-85	ENSRNO	protein_coding
Emilin1	-2.3981	-5.2711	4E-104	4E-102	8E-100	ENSRNO	protein_coding
Dpysl5	1.40295	2.64443	1.6E-11	1.2E-10	3.1E-07	ENSRNO	protein_coding
Kif3c	-1.7018	-3.2531	9E-56	3.2E-54	1.7E-51	ENSRNO	protein_coding
Dtnb	-1.7076	-3.2661	1.6E-58	6.1E-57	3.1E-54	ENSRNO	protein_coding
Efr3b	-6.5439	-93.306	6.7E-45	1.9E-43	1.3E-40	ENSRNO	protein_coding
Dnajc27	-7.4591	-175.97	5E-24	7.2E-23	9.5E-20	ENSRNO	protein_coding
Rhob	-1.3849	-2.6116	2E-41	5.3E-40	3.9E-37	ENSRNO	protein_coding
Osr1	-4.727	-26.483	1.3E-77	6.5E-76	2.4E-73	ENSRNO	protein_coding
Vsnl1	2.76766	6.81001	1.5E-36	3.3E-35	2.8E-32	ENSRNO	protein_coding
Fam49a	3.62618	12.3478	5.7E-23	7.9E-22	1.1E-18	ENSRNO	protein_coding
Mycn	-1.9201	-3.7846	0.0004	0.0012	1	ENSRNO	protein_coding
Nbas	-1.9504	-3.8648	2.1E-77	1.1E-75	4E-73	ENSRNO	protein_coding
Fam84a	-10.258	-1224.5	1.9E-05	7E-05	0.35752	ENSRNO	protein_coding
Trib2	-1.7865	-3.4499	8.4E-60	3.3E-58	1.6E-55	ENSRNO	protein_coding
E2f6	-1.3282	-2.5109	1.2E-36	2.7E-35	2.3E-32	ENSRNO	protein_coding
LOC6902	-1.7288	-3.3145	2.9E-06	1.2E-05	0.05503	ENSRNO	protein_coding
Kcnf1	-1.9849	-3.9582	7E-12	5.2E-11	1.3E-07	ENSRNO	protein_coding
Grhl1	-1.5071	-2.8424	1.8E-07	8.6E-07	0.00335	ENSRNO	protein_coding
Rnf144a	-3.522	-11.487	1E-133	2E-131	3E-129	ENSRNO	protein_coding
Rsad2	2.14721	4.42969	0.00152	0.0041	1	ENSRNO	protein_coding
Cmpk2	-1.3945	-2.6291	0.00406	0.01005	1	ENSRNO	protein_coding
Sox11	-6.7007	-104.02	2.2E-07	1.1E-06	0.00415	ENSRNO	protein_coding
Colec11	2.42017	5.35235	3.1E-08	1.6E-07	0.00058	ENSRNO	protein_coding
Adi1	-6.1524	-71.131	1.6E-22	2.1E-21	3E-18	ENSRNO	protein_coding
Pxdn	-1.9887	-3.9688	2.8E-85	1.6E-83	5.3E-81	ENSRNO	protein_coding
Fam110c	2.86174	7.2689	6.6E-15	6E-14	1.3E-10	ENSRNO	protein_coding
Cog5	-1.9112	-3.7613	8.1E-50	2.5E-48	1.5E-45	ENSRNO	protein_coding
Hbp1	-1.4673	-2.765	2E-41	5.3E-40	3.9E-37	ENSRNO	protein_coding
Pik3cg	8.4257	343.865	0.00036	0.00108	1	ENSRNO	protein_coding
AABR070	2.49494	5.63705	0.00687	0.01613	1	ENSRNO	protein_coding
Ahr	-2.3286	-5.0231	3E-99	2.4E-97	6.1E-95	ENSRNO	protein_coding
Meox2	-6.2832	-77.88	2.8E-31	5.5E-30	5.4E-27	ENSRNO	protein_coding

Lrrn3	1.79727	3.47561	1.2E-24	1.7E-23	2.2E-20	ENSRNO	protein_coding
Nrcam	3.13327	8.77422	0.0239	0.04855	1	ENSRNO	protein_coding
Stxbp6	-7.5989	-193.86	8E-47	2.4E-45	1.5E-42	ENSRNO	protein_coding
Nova1	-7.3913	-167.88	0.0023	0.00598	1	ENSRNO	protein_coding
Prkd1	-4.1515	-17.772	8E-253	3E-250	1E-248	ENSRNO	protein_coding
AABR070	3.44622	10.8997	0.01389	0.03017	1	ENSRNO	pseudogene
AABR070	1.4463	2.72508	0.0067	0.01578	1	ENSRNO	pseudogene
Akap6	2.19801	4.58847	6.1E-31	1.2E-29	1.2E-26	ENSRNO	protein_coding
Mipol1	-1.5442	-2.9165	4.3E-21	5.5E-20	8.2E-17	ENSRNO	protein_coding
Foxa1	-3.3087	-9.9089	1.4E-06	6E-06	0.02621	ENSRNO	protein_coding
Fbxo33	-1.5517	-2.9317	1.8E-42	4.8E-41	3.4E-38	ENSRNO	protein_coding
Mdga2	-3.8454	-14.374	1.2E-12	9.1E-12	2.2E-08	ENSRNO	protein_coding
LOC1009	-1.3894	-2.6197	3.7E-17	3.8E-16	7E-13	ENSRNO	protein_coding
Nin	-2.0577	-4.1633	1.4E-87	8.3E-86	2.6E-83	ENSRNO	protein_coding
Pygl	-6.4924	-90.037	2E-145	3E-143	4E-141	ENSRNO	protein_coding
ENSRNO	3.62317	12.3221	0.00895	0.02037	1	ENSRNO	protein_coding
Pcnx4	-2.2404	-4.7251	6E-25	9E-24	1.1E-20	ENSRNO	protein_coding
Dhrs7l1	-2.1517	-4.4435	3.5E-11	2.5E-10	6.7E-07	ENSRNO	protein_coding
Six1	-1.452	-2.7358	7.6E-41	1.9E-39	1.5E-36	ENSRNO	protein_coding
Prkch	-2.4438	-5.4405	0.00023	0.00073	1	ENSRNO	protein_coding
Rhoj	-7.3124	-158.94	1.5E-90	9.4E-89	2.8E-86	ENSRNO	protein_coding
Ppp1r36	-5.1068	-34.459	1E-26	1.7E-25	2E-22	ENSRNO	protein_coding
Rab15	-5.2777	-38.791	1E-221	3E-219	2E-217	ENSRNO	protein_coding
Fut8	-1.7619	-3.3913	2.3E-64	9.7E-63	4.4E-60	ENSRNO	protein_coding
Tmem229	-2.3347	-5.0445	0.00024	0.00075	1	ENSRNO	protein_coding
Plekhh1	-1.3615	-2.5694	1.2E-05	4.6E-05	0.22803	ENSRNO	protein_coding
Pigh	-1.493	-2.8147	2.5E-20	3.1E-19	4.8E-16	ENSRNO	protein_coding
Actn1	-1.6144	-3.0619	3E-58	1.1E-56	5.6E-54	ENSRNO	protein_coding
Galnt16	-4.7968	-27.797	0.00038	0.00114	1	ENSRNO	protein_coding
Plekhd1	1.96389	3.90112	0.01635	0.03472	1	ENSRNO	protein_coding
Smoc1	-1.8901	-3.7065	5.4E-29	9.6E-28	1E-24	ENSRNO	protein_coding
Slc8a3	1.84119	3.58305	0.00391	0.00969	1	ENSRNO	protein_coding
Ttc9	-5.7748	-54.75	1.2E-25	1.9E-24	2.3E-21	ENSRNO	protein_coding
AABR070	-6.2505	-76.133	0.01056	0.0236	1	ENSRNO	lincRNA
Sipa1l1	-1.5355	-2.8989	9.5E-51	3E-49	1.8E-46	ENSRNO	protein_coding
Pnma1	-3.795	-13.881	1.8E-08	9.9E-08	0.00035	ENSRNO	protein_coding
Abcd4	-1.8645	-3.6415	2.3E-64	9.6E-63	4.4E-60	ENSRNO	protein_coding
Zc2hc1c	-2.1996	-4.5934	0.00221	0.00578	1	ENSRNO	protein_coding
Batf	3.50849	11.3805	5.7E-09	3.3E-08	0.00011	ENSRNO	protein_coding
Tgfb3	-2.4508	-5.4672	5E-107	4E-105	9E-103	ENSRNO	protein_coding
Vash1	-6.8788	-117.69	2.3E-12	1.8E-11	4.4E-08	ENSRNO	protein_coding
Tmem63c	-3.8907	-14.833	3.6E-11	2.5E-10	6.8E-07	ENSRNO	protein_coding
Tmed8	-2.4589	-5.4981	8.8E-15	8E-14	1.7E-10	ENSRNO	protein_coding
Tshr	1.97855	3.94097	0.02225	0.04559	1	ENSRNO	protein_coding
Flrt2	-3.8452	-14.372	4E-143	5E-141	8E-139	ENSRNO	protein_coding
Ptpn21	-1.3235	-2.5027	5.6E-38	1.3E-36	1.1E-33	ENSRNO	protein_coding
Foxn3	-1.4462	-2.7248	1.6E-22	2.1E-21	3E-18	ENSRNO	protein_coding
Gpr68	5.33101	40.2527	2.8E-07	1.3E-06	0.00525	ENSRNO	protein_coding
Ccdc88c	1.60921	3.05086	1.9E-21	2.4E-20	3.5E-17	ENSRNO	protein_coding

Fbln5	-3.1617	-8.9489	9E-153	1E-150	2E-148	ENSRNO	protein_coding
Slc24a4	-2.7272	-6.6218	0.01584	0.03379	1	ENSRNO	protein_coding
Rin3	-7.0375	-131.37	6E-136	6E-134	1E-131	ENSRNO	protein_coding
Moap1	9.0669	536.301	0.00011	0.00038	1	ENSRNO	protein_coding
Asb2	3.704	13.0321	0.00674	0.01587	1	ENSRNO	protein_coding
Ifi27l2b	3.56567	11.8406	2E-24	3E-23	3.8E-20	ENSRNO	protein_coding
Serpina1	2.13916	4.40506	1.4E-06	6.1E-06	0.02637	ENSRNO	protein_coding
LOC5007	-1.552	-2.9323	7.1E-30	1.3E-28	1.4E-25	ENSRNO	protein_coding
Bdkrb2	-1.3987	-2.6367	1.2E-05	4.7E-05	0.22949	ENSRNO	protein_coding
Bdkrb1	-2.6182	-6.1398	3.2E-08	1.7E-07	0.00062	ENSRNO	protein_coding
Ak7	-3.2066	-9.2319	0.01937	0.04034	1	ENSRNO	protein_coding
LOC1009	9.50711	727.655	0.00011	0.00037	1	ENSRNO	protein_coding
ENSRNO	-2.2054	-4.6119	2.4E-11	1.7E-10	4.6E-07	ENSRNO	protein_coding
Hhipl1	-5.7908	-55.363	1.7E-41	4.4E-40	3.2E-37	ENSRNO	protein_coding
Cyp46a1	-6.3886	-83.786	9.1E-07	4.1E-06	0.01734	ENSRNO	protein_coding
Eml1	-1.5813	-2.9925	3.4E-48	1E-46	6.4E-44	ENSRNO	protein_coding
Evl	-2.9993	-7.996	5.5E-41	1.4E-39	1.1E-36	ENSRNO	protein_coding
Amn	-5.6755	-51.11	0.02071	0.04277	1	ENSRNO	protein_coding
Exoc3l4	2.19867	4.59057	3.2E-09	1.9E-08	6E-05	ENSRNO	protein_coding
Ckb	-2.0053	-4.0148	0.00027	0.00085	1	ENSRNO	protein_coding
Aspg	5.02136	32.4774	2.8E-76	1.4E-74	5.2E-72	ENSRNO	protein_coding
Adssl1	2.68095	6.41277	2.7E-05	9.8E-05	0.50723	ENSRNO	protein_coding
Cep170b	-2.298	-4.9176	2E-106	1E-104	3E-102	ENSRNO	protein_coding
Jag2	7.97596	251.769	5E-148	7E-146	1E-143	ENSRNO	protein_coding
Tmem121	-2.3114	-4.9635	0.00018	0.00057	1	ENSRNO	protein_coding
Igh-6	-6.5897	-96.312	4.2E-07	2E-06	0.00794	ENSRNO	protein_coding
Rapgef5	2.83386	7.12981	0.01465	0.03159	1	ENSRNO	protein_coding
Cdca7l	8.14342	282.756	8E-11	5.5E-10	1.5E-06	ENSRNO	protein_coding
Dnah11	3.34239	10.1428	9.5E-05	0.00032	1	ENSRNO	protein_coding
Itgb8	-7.8117	-224.67	7.3E-10	4.6E-09	1.4E-05	ENSRNO	protein_coding
Tmem196	-3.45	-10.928	0.00177	0.00472	1	ENSRNO	protein_coding
LOC1083	-4.409	-21.245	2.7E-05	9.8E-05	0.51162	ENSRNO	lincRNA
Erbb3	3.21539	9.28814	1.2E-21	1.5E-20	2.2E-17	ENSRNO	protein_coding
Pmel	-1.6601	-3.1604	1.7E-08	9.2E-08	0.00032	ENSRNO	protein_coding
AABR070	-2.2851	-4.8741	0.00024	0.00076	1	ENSRNO	lincRNA
ENSRNO	-1.9934	-3.9817	4E-31	7.6E-30	7.6E-27	ENSRNO	protein_coding
Gna15	-3.2545	-9.5436	1.3E-06	5.5E-06	0.02389	ENSRNO	protein_coding
Ncln	-1.3575	-2.5624	2.8E-41	7.3E-40	5.4E-37	ENSRNO	protein_coding
Nfic	-2.213	-4.6365	3.4E-45	9.6E-44	6.5E-41	ENSRNO	protein_coding
AC094643	-1.9978	-3.9939	3.6E-37	8.2E-36	6.8E-33	ENSRNO	protein_coding
Dohh	-1.8103	-3.5071	1.3E-70	6.2E-69	2.5E-66	ENSRNO	protein_coding
Fzr1	-2.4735	-5.5537	4E-127	5E-125	8E-123	ENSRNO	protein_coding
LOC6906	-1.7938	-3.4673	3E-11	2.1E-10	5.7E-07	ENSRNO	protein_coding
Mfsd12	-1.7067	-3.2642	2.4E-36	5.4E-35	4.7E-32	ENSRNO	protein_coding
Hmg20b	-2.1242	-4.3595	1E-92	6.7E-91	1.9E-88	ENSRNO	protein_coding
Gipc3	-6.0657	-66.983	4.1E-34	8.6E-33	7.7E-30	ENSRNO	protein_coding
Tbxa2r	-1.9254	-3.7983	7.4E-09	4.2E-08	0.00014	ENSRNO	protein_coding
Cactin	-1.7506	-3.365	3.6E-65	1.6E-63	6.9E-61	ENSRNO	protein_coding
Pip5k1c	-2.3828	-5.2153	2E-119	2E-117	4E-115	ENSRNO	protein_coding

Tjp3	-1.8851	-3.6938	4E-09	2.3E-08	7.7E-05	ENSRNO	protein_coding
Apba3	-2.489	-5.6138	7E-127	7E-125	1E-122	ENSRNO	protein_coding
Mrpl54	-1.9357	-3.8257	3.6E-76	1.8E-74	6.9E-72	ENSRNO	protein_coding
Matk	-4.1783	-18.104	4.1E-08	2.1E-07	0.00077	ENSRNO	protein_coding
Zfr2	-2.7343	-6.6543	2.1E-13	1.7E-12	3.9E-09	ENSRNO	protein_coding
Atcay	-5.4205	-42.829	2.8E-52	9.3E-51	5.4E-48	ENSRNO	protein_coding
Dapk3	-1.6124	-3.0576	1.1E-38	2.6E-37	2.1E-34	ENSRNO	protein_coding
Eef2	-1.5671	-2.963	1.8E-55	6.2E-54	3.3E-51	ENSRNO	protein_coding
Pias4	-2.4741	-5.5561	2.5E-66	1.1E-64	4.8E-62	ENSRNO	protein_coding
Zbtb7a	-2.2907	-4.8928	7.7E-66	3.4E-64	1.5E-61	ENSRNO	protein_coding
Map2k2	-2.5355	-5.7979	2E-135	2E-133	4E-131	ENSRNO	protein_coding
Thop1	-1.8471	-3.5977	9.9E-74	4.8E-72	1.9E-69	ENSRNO	protein_coding
Sgta	-2.0953	-4.273	2.6E-95	1.8E-93	4.9E-91	ENSRNO	protein_coding
Slc39a3	-2.3162	-4.9802	9E-102	7E-100	1.7E-97	ENSRNO	protein_coding
Diras1	-1.3845	-2.6109	0.0205	0.04238	1	ENSRNO	protein_coding
Adamtsl5	1.34605	2.54215	7.1E-13	5.6E-12	1.3E-08	ENSRNO	protein_coding
Efna2	-2.9555	-7.7568	5.3E-06	2.1E-05	0.1009	ENSRNO	protein_coding
Palm	-2.6856	-6.4334	1.1E-55	3.8E-54	2.1E-51	ENSRNO	protein_coding
Cnn2	-1.4827	-2.7946	4.4E-48	1.3E-46	8.5E-44	ENSRNO	protein_coding
Fgf22	1.45036	2.73277	5.7E-06	2.3E-05	0.10805	ENSRNO	protein_coding
Hcn2	-5.9761	-62.949	5.9E-06	2.4E-05	0.11279	ENSRNO	protein_coding
Cyp4f37_	3.13754	8.80021	0.00386	0.00958	1	ENSRNO	protein_coding
Casp14	3.92232	15.1613	0.00377	0.00939	1	ENSRNO	protein_coding
Notch3	-3.6982	-12.98	9.5E-26	1.5E-24	1.8E-21	ENSRNO	protein_coding
Cyp4f5	1.34986	2.54888	2.7E-05	9.8E-05	0.51	ENSRNO	protein_coding
Cyp4f37_	3.44407	10.8835	0.00184	0.00489	1	ENSRNO	protein_coding
AABR070	2.66186	6.32849	0.01731	0.03654	1	ENSRNO	lincRNA
Cyp4f6	-1.8261	-3.5457	4.4E-06	1.8E-05	0.08411	ENSRNO	protein_coding
Zfp563	-2.6689	-6.3596	2.9E-08	1.5E-07	0.00055	ENSRNO	protein_coding
Zfp955a	-4.6138	-24.485	1.1E-52	3.7E-51	2.1E-48	ENSRNO	protein_coding
Myo1f	1.97366	3.92762	0.0012	0.00331	1	ENSRNO	protein_coding
LOC1025	6.8907	118.661	0.00371	0.00926	1	ENSRNO	protein_coding
AC09802	2.50609	5.68077	0.00496	0.01206	1	ENSRNO	lincRNA
AABR070	-2.5806	-5.9818	0.00069	0.00198	1	ENSRNO	lincRNA
Syn3	4.22553	18.7073	0.00163	0.00438	1	ENSRNO	protein_coding
Nuak1	-1.9599	-3.8904	1.5E-80	7.8E-79	2.8E-76	ENSRNO	protein_coding
Rfx4	-7.8684	-233.68	7.4E-22	9.8E-21	1.4E-17	ENSRNO	protein_coding
Aldh1l2	-5.2958	-39.283	2.7E-12	2.1E-11	5.2E-08	ENSRNO	protein_coding
Ikbip	-1.3585	-2.5642	1.1E-37	2.6E-36	2.2E-33	ENSRNO	protein_coding
Cdk17	-1.5022	-2.8327	4.4E-46	1.3E-44	8.3E-42	ENSRNO	protein_coding
Ntn4	-2.1457	-4.425	3.6E-11	2.6E-10	6.9E-07	ENSRNO	protein_coding
Tmcc3	-6.5868	-96.119	5.7E-78	2.9E-76	1.1E-73	ENSRNO	protein_coding
Plxnc1	-5.1292	-34.999	3.3E-32	6.6E-31	6.3E-28	ENSRNO	protein_coding
Socs2	-3.6122	-12.228	2.4E-06	1E-05	0.04533	ENSRNO	protein_coding
Dcn	-1.9618	-3.8954	0.00243	0.00629	1	ENSRNO	protein_coding
Lum	-5.8678	-58.394	3E-168	5E-166	6E-164	ENSRNO	protein_coding
AABR070	3.06826	8.38762	0.00509	0.01234	1	ENSRNO	pseudogene
Tmtc2	4.58741	24.0407	1.3E-05	4.9E-05	0.24081	ENSRNO	protein_coding
Syt1	2.03221	4.0903	3.2E-67	1.4E-65	6.1E-63	ENSRNO	protein_coding

Caps2	-3.1476	-8.8616	2E-05	7.4E-05	0.37881	ENSRNO	protein_coding
Kcnc2	-4.5004	-22.634	0.00087	0.00247	1	ENSRNO	protein_coding
Ptprr	5.24568	37.9409	1.5E-56	5.4E-55	2.8E-52	ENSRNO	protein_coding
Ptprb	3.09089	8.52023	0.00094	0.00265	1	ENSRNO	protein_coding
Lyz2	5.8389	57.2379	0.01445	0.03124	1	ENSRNO	protein_coding
Dyrk2	-1.6715	-3.1855	9.8E-53	3.3E-51	1.9E-48	ENSRNO	protein_coding
Msrb3	-1.3254	-2.506	7.6E-39	1.8E-37	1.4E-34	ENSRNO	protein_coding
Tbc1d30	2.2988	4.92049	8.4E-09	4.7E-08	0.00016	ENSRNO	protein_coding
LOC1009	-8.2982	-314.78	0.00158	0.00424	1	ENSRNO	protein_coding
Srgap1	-2.9069	-7.5003	5.2E-18	5.7E-17	9.9E-14	ENSRNO	protein_coding
Tmem5	-1.7685	-3.4071	8.2E-21	1E-19	1.6E-16	ENSRNO	protein_coding
Grip1	-4.5132	-22.835	1.1E-86	6.4E-85	2E-82	ENSRNO	protein_coding
AABR070	3.34953	10.1932	0.01394	0.03026	1	ENSRNO	lincRNA
Fam19a2	4.47592	22.2529	0.00083	0.00236	1	ENSRNO	protein_coding
Ppm1h	-2.2236	-4.6705	3.4E-35	7.3E-34	6.4E-31	ENSRNO	protein_coding
Avpr1a	-7.5033	-181.44	9.2E-97	6.8E-95	1.8E-92	ENSRNO	protein_coding
Lrig3	-1.8818	-3.6854	8.7E-50	2.7E-48	1.7E-45	ENSRNO	protein_coding
Avil	1.99847	3.99576	4.4E-10	2.8E-09	8.3E-06	ENSRNO	protein_coding
Mettl21b	2.44808	5.4569	0.00455	0.01115	1	ENSRNO	protein_coding
Arhgef25	-2.971	-7.8408	2.2E-55	7.6E-54	4.1E-51	ENSRNO	protein_coding
Inhbe	-1.7998	-3.4817	0.00015	0.00048	1	ENSRNO	protein_coding
Rdh16	-4.8834	-29.516	1E-19	1.2E-18	1.9E-15	ENSRNO	protein_coding
Nipal2	3.47764	11.1397	1.7E-40	4.3E-39	3.2E-36	ENSRNO	protein_coding
Rgs22	-3.4485	-10.917	1.9E-23	2.7E-22	3.6E-19	ENSRNO	protein_coding
Spag1	-1.7086	-3.2684	8.3E-10	5.2E-09	1.6E-05	ENSRNO	protein_coding
Snx31	2.76115	6.77939	0.00389	0.00964	1	ENSRNO	protein_coding
Ncald	-1.3432	-2.5371	2.9E-07	1.4E-06	0.00554	ENSRNO	protein_coding
Rims2	-1.3925	-2.6253	5.6E-26	9E-25	1.1E-21	ENSRNO	protein_coding
Tmem74	-6.9502	-123.65	0.0044	0.01083	1	ENSRNO	protein_coding
Tnfrsf11b	-1.6237	-3.0816	4.4E-27	7.4E-26	8.4E-23	ENSRNO	protein_coding
Deptor	-2.1697	-4.4994	1.3E-31	2.5E-30	2.4E-27	ENSRNO	protein_coding
Col14a1	-4.1914	-18.271	4.3E-06	1.8E-05	0.08161	ENSRNO	protein_coding
Sntb1	-2.9896	-7.9426	7.7E-65	3.3E-63	1.5E-60	ENSRNO	protein_coding
Tmem65	-1.488	-2.805	1.8E-14	1.6E-13	3.4E-10	ENSRNO	protein_coding
LOC1083	3.62183	12.3106	1.1E-26	1.8E-25	2.1E-22	ENSRNO	protein_coding
Tmem71	1.69742	3.2432	5.5E-07	2.5E-06	0.01047	ENSRNO	protein_coding
Ndrg1	1.60073	3.03298	2.5E-52	8.2E-51	4.7E-48	ENSRNO	protein_coding
St3gal1	1.78471	3.44548	1.1E-66	5E-65	2.1E-62	ENSRNO	protein_coding
Khdrbs3	-7.933	-244.39	1.2E-56	4.5E-55	2.4E-52	ENSRNO	protein_coding
Ly6k	3.52365	11.5007	7.1E-11	4.9E-10	1.3E-06	ENSRNO	protein_coding
Ly6l	-1.7721	-3.4156	1.8E-09	1.1E-08	3.4E-05	ENSRNO	lincRNA
Gsdmd	-2.6791	-6.4047	2.4E-83	1.3E-81	4.5E-79	ENSRNO	protein_coding
Naprt	-2.0709	-4.2016	0.0002	0.00064	1	ENSRNO	protein_coding
Fam83h	-1.4419	-2.7168	2.6E-16	2.6E-15	4.9E-12	ENSRNO	protein_coding
Nrbp2	-1.9383	-3.8325	1.9E-10	1.2E-09	3.5E-06	ENSRNO	protein_coding
Scx	-1.5999	-3.0313	5.1E-21	6.4E-20	9.6E-17	ENSRNO	protein_coding
Mfsd3	1.34694	2.54371	1.3E-05	5.1E-05	0.25083	ENSRNO	protein_coding
AABR070	1.6776	3.19895	1.3E-19	1.5E-18	2.4E-15	ENSRNO	protein_coding
Apol3	4.24219	18.9246	2.4E-09	1.5E-08	4.6E-05	ENSRNO	protein_coding

LOC1009	1.41346	2.66375	2E-10	1.3E-09	3.8E-06	ENSRNO	protein_coding
Apol11a	1.59583	3.02269	0.00775	0.01794	1	ENSRNO	protein_coding
RGD1309	2.05864	4.16593	2.4E-17	2.5E-16	4.6E-13	ENSRNO	protein_coding
Apol9a	2.73842	6.67339	1.1E-39	2.7E-38	2.1E-35	ENSRNO	protein_coding
Foxred2	1.44727	2.72691	4.4E-14	3.8E-13	8.3E-10	ENSRNO	protein_coding
Tmprss6	-2.5521	-5.8648	0.01295	0.02834	1	ENSRNO	protein_coding
C1qtnf6	-3.086	-8.4911	7.7E-63	3.1E-61	1.5E-58	ENSRNO	protein_coding
AABR070	-2.9034	-7.4819	0.01185	0.02617	1	ENSRNO	lincRNA
Cyth4	2.87821	7.35235	0.00042	0.00126	1	ENSRNO	protein_coding
Elfn2	2.04449	4.12528	2.8E-09	1.6E-08	5.2E-05	ENSRNO	protein_coding
Lgals2	-3.6113	-12.221	1.2E-05	4.8E-05	0.23713	ENSRNO	protein_coding
Pdxp	-3.8062	-13.989	1.6E-24	2.3E-23	3E-20	ENSRNO	protein_coding
Gcat	-7.2814	-155.57	4.3E-34	9E-33	8.1E-30	ENSRNO	protein_coding
Fam227a	2.81234	7.02422	0.00123	0.00337	1	ENSRNO	protein_coding
Cbx7	-2.0149	-4.0416	1.4E-11	1E-10	2.6E-07	ENSRNO	protein_coding
Syngr1	4.53905	23.2482	2E-139	2E-137	3E-135	ENSRNO	protein_coding
Enthd1	1.77431	3.42075	0.00211	0.00553	1	ENSRNO	protein_coding
Fam83f	1.75962	3.3861	0.01142	0.02532	1	ENSRNO	protein_coding
Cyp2d1	1.82482	3.54264	0.00022	0.0007	1	ENSRNO	protein_coding
Bik	4.65565	25.2051	9.3E-18	1E-16	1.8E-13	ENSRNO	protein_coding
Tspo	1.38122	2.60489	8.2E-19	9.3E-18	1.6E-14	ENSRNO	protein_coding
Scube1	2.55952	5.8951	1.9E-06	8.1E-06	0.03568	ENSRNO	protein_coding
Efcab6	2.37093	5.17273	0.00341	0.00858	1	ENSRNO	protein_coding
Parvb	-2.7902	-6.9173	2.5E-37	5.6E-36	4.7E-33	ENSRNO	protein_coding
Phf21b	1.72804	3.31277	2.3E-06	9.7E-06	0.0432	ENSRNO	protein_coding
Fam118a	-5.8618	-58.155	1.2E-61	4.9E-60	2.4E-57	ENSRNO	protein_coding
Fbln1	-3.7619	-13.565	2.3E-25	3.5E-24	4.3E-21	ENSRNO	protein_coding
Wnt7b	-1.996	-3.9888	8.4E-05	0.00029	1	ENSRNO	protein_coding
Celsr1	-2.397	-5.2671	2.5E-11	1.7E-10	4.7E-07	ENSRNO	protein_coding
AABR070	-1.8362	-3.5707	0.00077	0.0022	1	ENSRNO	lincRNA
Panx2	-2.3863	-5.2281	0.00976	0.022	1	ENSRNO	protein_coding
Mapk12	7.38979	167.706	1.1E-49	3.3E-48	2E-45	ENSRNO	protein_coding
Kif21a	-1.8176	-3.525	2.2E-46	6.3E-45	4.1E-42	ENSRNO	protein_coding
Abcd2	-2.0319	-4.0895	4.3E-09	2.5E-08	8.2E-05	ENSRNO	protein_coding
Pdzrn4	2.98924	7.94057	0.00124	0.00339	1	ENSRNO	protein_coding
Adamts20	3.28497	9.74709	6E-08	3.1E-07	0.00114	ENSRNO	protein_coding
Slc38a4	-5.3641	-41.185	1E-163	2E-161	2E-159	ENSRNO	protein_coding
Rapgef3	-2.8989	-7.4587	4.1E-09	2.4E-08	7.7E-05	ENSRNO	protein_coding
Vdr	-2.2735	-4.8348	3E-14	2.7E-13	5.8E-10	ENSRNO	protein_coding
Tmem106	-1.5767	-2.9828	7.5E-17	7.7E-16	1.4E-12	ENSRNO	protein_coding
Ccdc65	-2.205	-4.6109	3.8E-09	2.2E-08	7.3E-05	ENSRNO	protein_coding
Wnt10b	-6.6449	-100.07	4.7E-25	7.1E-24	8.9E-21	ENSRNO	protein_coding
Prph	-2.4954	-5.639	9E-05	0.00031	1	ENSRNO	protein_coding
Fam186b	3.24552	9.48418	0.02014	0.04171	1	ENSRNO	protein_coding
Bcdin3d	1.54861	2.92534	2.6E-07	1.3E-06	0.00504	ENSRNO	protein_coding
Pou6f1	-1.5107	-2.8495	2.7E-15	2.5E-14	5.1E-11	ENSRNO	protein_coding
AABR070	-1.502	-2.8323	0.01562	0.0334	1	ENSRNO	lincRNA
Galnt6	-4.3858	-20.906	1.5E-57	5.6E-56	2.9E-53	ENSRNO	protein_coding
Slc4a8	4.69955	25.984	5.4E-14	4.6E-13	1E-09	ENSRNO	protein_coding

Acvrl1	-2.6303	-6.1916	0.02103	0.04338	1	ENSRNO	protein_coding
Krt7	-1.7648	-3.3982	3.7E-07	1.7E-06	0.00707	ENSRNO	protein_coding
Krt71	-3.3129	-9.9374	0.00449	0.01101	1	ENSRNO	protein_coding
Krt1	4.14275	17.6641	0.00438	0.01078	1	ENSRNO	protein_coding
Krt76	6.02556	65.1438	1.8E-21	2.4E-20	3.5E-17	ENSRNO	protein_coding
Krt8	1.37477	2.59326	2.3E-26	3.8E-25	4.5E-22	ENSRNO	protein_coding
Igfbp6	3.0003	8.00169	2E-54	6.7E-53	3.7E-50	ENSRNO	protein_coding
Itgb7	2.76861	6.81452	0.00045	0.00136	1	ENSRNO	protein_coding
Sp7	-6.1709	-72.047	4.2E-10	2.7E-09	7.9E-06	ENSRNO	protein_coding
Amhr2	1.83028	3.55606	0.00928	0.02104	1	ENSRNO	protein_coding
LOC1009	-1.4629	-2.7566	0.0032	0.00811	1	ENSRNO	protein_coding
Tbca_2	-6.4014	-84.532	0.00894	0.02036	1	ENSRNO	protein_coding
Hoxc13	-3.6476	-12.532	8.6E-14	7.3E-13	1.6E-09	ENSRNO	protein_coding
Nckap1l	4.52911	23.0886	2E-261	7E-259	3E-257	ENSRNO	protein_coding
Pde1b	2.19992	4.59453	0.00014	0.00046	1	ENSRNO	protein_coding
AABR070	-3.0669	-8.3795	1.9E-06	8.2E-06	0.03654	ENSRNO	lincRNA
Casp1	3.53201	11.5675	6.3E-51	2E-49	1.2E-46	ENSRNO	protein_coding
Mmp13	-2.3615	-5.139	7.6E-87	4.5E-85	1.4E-82	ENSRNO	protein_coding
Mmp3	4.75498	27.0017	0	0	0	ENSRNO	protein_coding
Mmp1	2.94205	7.68505	0.00245	0.00635	1	ENSRNO	protein_coding
Mmp10	4.20207	18.4056	2E-125	2E-123	4E-121	ENSRNO	protein_coding
Tmem123	-2.3182	-4.9869	1E-102	1E-100	2.7E-98	ENSRNO	protein_coding
Arhgap42	-1.405	-2.6482	1.5E-24	2.2E-23	2.8E-20	ENSRNO	protein_coding
Endod1	1.665	3.17113	3.4E-58	1.3E-56	6.5E-54	ENSRNO	protein_coding
Piwil4	4.14925	17.7439	2.6E-09	1.5E-08	4.9E-05	ENSRNO	protein_coding
Fut4	-4.4822	-22.35	1.2E-17	1.2E-16	2.2E-13	ENSRNO	protein_coding
Panx1	2.72923	6.63102	0.00054	0.00158	1	ENSRNO	protein_coding
Smco4	-2.8868	-7.3962	5.2E-19	5.9E-18	9.9E-15	ENSRNO	protein_coding
Deup1	5.26502	38.453	3.5E-07	1.7E-06	0.00674	ENSRNO	protein_coding
Fat3	-1.7218	-3.2984	6.9E-44	1.9E-42	1.3E-39	ENSRNO	protein_coding
Naalad2	-1.7536	-3.372	0.0181	0.03801	1	ENSRNO	protein_coding
Olfm2	-2.0821	-4.2341	3.4E-09	2E-08	6.5E-05	ENSRNO	protein_coding
Col5a3	-4.7423	-26.766	1E-251	4E-249	2E-247	ENSRNO	protein_coding
LOC5009	6.80042	111.463	0.00446	0.01095	1	ENSRNO	protein_coding
Icam5	-3.1039	-8.5973	5.2E-20	6.3E-19	1E-15	ENSRNO	protein_coding
Pde4a	3.15534	8.90949	8.7E-15	7.9E-14	1.6E-10	ENSRNO	protein_coding
LOC6911	1.82068	3.53247	0.00613	0.01459	1	ENSRNO	protein_coding
Dock6	-4.4795	-22.309	5E-245	2E-242	1E-240	ENSRNO	protein_coding
Plppr2	-2.4917	-5.6244	6.3E-19	7.2E-18	1.2E-14	ENSRNO	protein_coding
Cnn1	-4.7185	-26.328	1.2E-11	9.1E-11	2.4E-07	ENSRNO	protein_coding
ENSRNO	-4.5022	-22.663	7.9E-16	7.6E-15	1.5E-11	ENSRNO	protein_coding
AABR070	-1.4987	-2.8259	0.00059	0.00173	1	ENSRNO	lincRNA
Glb1l2	-1.6187	-3.0709	2E-53	6.7E-52	3.8E-49	ENSRNO	protein_coding
Jam3	-6.1296	-70.016	2.2E-24	3.2E-23	4.1E-20	ENSRNO	protein_coding
Igsf9b	2.72042	6.59066	5.5E-38	1.3E-36	1.1E-33	ENSRNO	protein_coding
Opcml	-6.5363	-92.816	0.00728	0.01698	1	ENSRNO	protein_coding
Ntm	-8.7394	-427.38	5E-116	5E-114	1E-111	ENSRNO	protein_coding
Adamts8	2.28367	4.86914	4E-101	3E-99	7E-97	ENSRNO	protein_coding
St14	2.92679	7.60418	1.4E-18	1.5E-17	2.6E-14	ENSRNO	protein_coding

Fli1	9.77361	875.288	3.1E-05	0.00011	0.59047	ENSRNO	protein_coding
Ddx25	1.49997	2.82837	7E-07	3.2E-06	0.01334	ENSRNO	protein_coding
Pate4	4.01916	16.2139	0.00016	0.00052	1	ENSRNO	protein_coding
Pknox2	6.92144	121.216	6.6E-54	2.2E-52	1.3E-49	ENSRNO	protein_coding
Spa17	-4.1549	-17.814	9.2E-05	0.00031	1	ENSRNO	protein_coding
LOC1715	3.44666	10.903	0.01232	0.02711	1	ENSRNO	protein_coding
LOC1009	1.82789	3.55017	0.01196	0.02636	1	ENSRNO	protein_coding
LOC1009	1.96388	3.90111	0.01049	0.02346	1	ENSRNO	protein_coding
RGD1311	6.63201	99.1822	4.5E-43	1.2E-41	8.6E-39	ENSRNO	protein_coding
Gramd1b	3.23281	9.40098	2.4E-14	2.1E-13	4.6E-10	ENSRNO	protein_coding
AC13326	-3.8805	-14.728	0.00063	0.00182	1	ENSRNO	protein_coding
Tbcel	-3.3991	-10.549	1.8E-17	1.9E-16	3.4E-13	ENSRNO	protein_coding
Grik4	6.28015	77.7168	0.00843	0.01933	1	ENSRNO	protein_coding
Trim29	2.36221	5.14157	2E-37	4.7E-36	3.9E-33	ENSRNO	protein_coding
AABR070	2.65892	6.31559	2.8E-05	0.0001	0.52447	ENSRNO	lincRNA
Nectin1	1.47858	2.78675	4.2E-46	1.2E-44	7.9E-42	ENSRNO	protein_coding
Thy1	-4.2631	-19.201	5E-147	6E-145	9E-143	ENSRNO	protein_coding
Usp2	-3.7013	-13.008	5.2E-55	1.8E-53	9.8E-51	ENSRNO	protein_coding
Mfrp	-4.1334	-17.55	6.8E-08	3.5E-07	0.0013	ENSRNO	protein_coding
C1qtnf5	-5.8333	-57.016	3E-164	5E-162	6E-160	ENSRNO	protein_coding
Tmem25	1.71252	3.27734	1.2E-05	4.6E-05	0.22801	ENSRNO	protein_coding
Fxyd6	5.28249	38.9214	2.1E-24	3.1E-23	4E-20	ENSRNO	protein_coding
Fxyd2	5.52468	46.0358	9E-55	3.1E-53	1.7E-50	ENSRNO	protein_coding
Tagln	-5.3225	-40.016	2E-285	1E-282	3E-281	ENSRNO	protein_coding
Apoa5	3.84019	14.3223	2.1E-05	8E-05	0.4088	ENSRNO	protein_coding
Cadm1	-4.4596	-22.002	0	0	0	ENSRNO	protein_coding
Zbtb16	2.0978	4.28056	7.6E-23	1E-21	1.4E-18	ENSRNO	protein_coding
Plet1	2.28835	4.88497	7.7E-09	4.3E-08	0.00015	ENSRNO	protein_coding
Pts	2.07414	4.21092	2.9E-15	2.7E-14	5.6E-11	ENSRNO	protein_coding
Hspb2	3.43253	10.7968	1.5E-07	7.5E-07	0.0029	ENSRNO	protein_coding
Cryab	1.73124	3.32013	8.9E-56	3.2E-54	1.7E-51	ENSRNO	protein_coding
Layn	-3.2609	-9.5859	2.1E-20	2.6E-19	4E-16	ENSRNO	protein_coding
Btg4	1.75709	3.38017	0.00134	0.00366	1	ENSRNO	protein_coding
Pou2af1	3.16498	8.96919	0.00032	0.00098	1	ENSRNO	protein_coding
Exph5	-2.2994	-4.9225	1.7E-96	1.3E-94	3.3E-92	ENSRNO	protein_coding
Rab39a	-2.6247	-6.1678	0.00598	0.01426	1	ENSRNO	protein_coding
Elmod1	4.70016	25.995	6E-90	3.8E-88	1.1E-85	ENSRNO	protein_coding
Tnfaip8l3	2.05072	4.14313	5.6E-30	1E-28	1.1E-25	ENSRNO	protein_coding
Cyp19a1	2.30588	4.94468	1.5E-29	2.7E-28	2.9E-25	ENSRNO	protein_coding
Sh2d7	4.45605	21.9486	0.00128	0.0035	1	ENSRNO	protein_coding
Hykk	-3.7604	-13.552	0.00059	0.00173	1	ENSRNO	protein_coding
Ube2q2	-2.5385	-5.8098	1.7E-54	5.7E-53	3.2E-50	ENSRNO	protein_coding
Rcn2	-2.608	-6.0965	1E-120	1E-118	2E-116	ENSRNO	protein_coding
Odf3l1	1.42861	2.69188	0.00036	0.00109	1	ENSRNO	protein_coding
Cspg4	3.85971	14.5174	0	5E-307	1E-305	ENSRNO	protein_coding
Neil1	-1.7646	-3.3978	2E-16	2E-15	3.9E-12	ENSRNO	protein_coding
Rpp25	3.3151	9.95281	6.3E-42	1.7E-40	1.2E-37	ENSRNO	protein_coding
Lman1l	2.7377	6.67006	0.00034	0.00105	1	ENSRNO	protein_coding
Cyp1a1	6.20729	73.889	1.1E-16	1.1E-15	2.1E-12	ENSRNO	protein_coding

Arid3b	-6.2724	-77.3	3.2E-17	3.3E-16	6.1E-13	ENSRNO	protein_coding
Sema7a	-2.5932	-6.0342	6E-124	6E-122	1E-119	ENSRNO	protein_coding
Islr	2.27025	4.82408	0.00388	0.00962	1	ENSRNO	protein_coding
RGD1562	2.69716	6.48523	2E-135	3E-133	5E-131	ENSRNO	protein_coding
AABR070	3.03567	8.20026	0.0006	0.00175	1	ENSRNO	protein_coding
Rec114	1.9779	3.93919	1.4E-40	3.5E-39	2.7E-36	ENSRNO	protein_coding
Hcn4	-2.1038	-4.2983	1.3E-07	6.5E-07	0.0025	ENSRNO	protein_coding
Neo1	-1.3491	-2.5476	3.9E-40	9.8E-39	7.5E-36	ENSRNO	protein_coding
Rn50_8_(	5.65807	50.495	5.6E-28	9.6E-27	1.1E-23	ENSRNO	processed_transcript
Rn50_8_(	5.3882	41.8802	2.9E-10	1.9E-09	5.5E-06	ENSRNO	processed_transcript
Rn50_8_(	5.78253	55.0448	1.5E-08	8.2E-08	0.00028	ENSRNO	processed_transcript
Larp6	-2.6715	-6.3709	1.7E-09	1E-08	3.2E-05	ENSRNO	protein_coding
Coro2b	-4.4042	-21.174	1.2E-49	3.6E-48	2.2E-45	ENSRNO	protein_coding
Itga11	-4.9482	-30.871	0	0	0	ENSRNO	protein_coding
Calml4	1.656	3.15143	9.1E-08	4.6E-07	0.00173	ENSRNO	protein_coding
Iqch	2.8258	7.09008	0.01557	0.03329	1	ENSRNO	protein_coding
Snapc5	2.24399	4.73705	3.7E-34	7.8E-33	7E-30	ENSRNO	protein_coding
Cilp	-4.9657	-31.248	2E-101	1E-99	3.1E-97	ENSRNO	protein_coding
Rasl12	-1.6399	-3.1165	0.00012	0.0004	1	ENSRNO	protein_coding
Pif1	-2.1473	-4.43	6.5E-15	5.9E-14	1.2E-10	ENSRNO	protein_coding
Dapk2	3.46662	11.0549	1.2E-45	3.5E-44	2.3E-41	ENSRNO	protein_coding
Tpm1	-1.4371	-2.7079	6.3E-46	1.8E-44	1.2E-41	ENSRNO	protein_coding
Gcnt3	1.6544	3.14792	0.00276	0.00707	1	ENSRNO	protein_coding
Pygo1	-3.1787	-9.0552	2.8E-41	7.2E-40	5.3E-37	ENSRNO	protein_coding
Unc13c	5.3417	40.5521	7.5E-26	1.2E-24	1.4E-21	ENSRNO	protein_coding
Onecut1	-7.2404	-151.21	0.00283	0.00725	1	ENSRNO	protein_coding
Myo5c	-1.682	-3.2087	4.5E-06	1.8E-05	0.08532	ENSRNO	protein_coding
Bmp5	-5.8872	-59.187	1.9E-56	6.9E-55	3.6E-52	ENSRNO	protein_coding
Hmgcll1	-6.8195	-112.95	3.6E-12	2.7E-11	6.9E-08	ENSRNO	protein_coding
Ick	-1.4657	-2.7619	4.8E-41	1.2E-39	9.1E-37	ENSRNO	protein_coding
Gsta4	2.36901	5.16588	5.3E-14	4.5E-13	1E-09	ENSRNO	protein_coding
Gsta5	-2.1688	-4.4965	5.1E-06	2.1E-05	0.09714	ENSRNO	protein_coding
Htr1b	1.38459	2.61097	0.006	0.0143	1	ENSRNO	protein_coding
Sh3bgrl2	3.52801	11.5355	4.5E-16	4.4E-15	8.6E-12	ENSRNO	protein_coding
Prss35	-5.671	-50.951	1.9E-11	1.4E-10	3.6E-07	ENSRNO	protein_coding
Snap91	6.24685	75.9433	2.8E-10	1.8E-09	5.3E-06	ENSRNO	protein_coding
Rasgrf1	-1.7089	-3.2692	4.5E-06	1.8E-05	0.08487	ENSRNO	protein_coding
Ctsh	-4.0607	-16.687	0.00251	0.0065	1	ENSRNO	protein_coding
Adamts7	2.42591	5.37367	9E-111	8E-109	2E-106	ENSRNO	protein_coding
Zic1	7.35513	163.724	8.1E-23	1.1E-21	1.5E-18	ENSRNO	protein_coding
Zic4	4.22312	18.676	5.4E-11	3.8E-10	1E-06	ENSRNO	protein_coding
PCOLCE2	2.15239	4.44565	9E-99	7.1E-97	1.8E-94	ENSRNO	protein_coding
Pls1	3.7945	13.8758	3E-240	9E-238	6E-236	ENSRNO	protein_coding
Spsb4	-1.976	-3.934	1.9E-31	3.6E-30	3.5E-27	ENSRNO	protein_coding
Nmnat3	-7.0389	-131.5	3.3E-08	1.7E-07	0.00063	ENSRNO	protein_coding
LOC1036	2.86118	7.2661	0.02373	0.04824	1	ENSRNO	protein_coding
Esyt3	-2.3892	-5.2385	2E-14	1.8E-13	3.9E-10	ENSRNO	protein_coding
Mras	-1.6666	-3.1746	4.6E-35	9.9E-34	8.8E-31	ENSRNO	protein_coding
Slc35g2	3.14038	8.81754	0.00478	0.01165	1	ENSRNO	protein_coding

Ky	6.22593	74.8498	1.5E-20	1.8E-19	2.8E-16	ENSRNO	protein_coding
Slco2a1	5.15895	35.7273	1.7E-27	2.9E-26	3.2E-23	ENSRNO	protein_coding
RGD1310	4.3058	19.7776	1.5E-27	2.5E-26	2.8E-23	ENSRNO	protein_coding
Tmem108	-3.9997	-15.996	1.3E-83	7.1E-82	2.4E-79	ENSRNO	protein_coding
Acpp	-1.4419	-2.7168	1.2E-37	2.7E-36	2.2E-33	ENSRNO	protein_coding
Cpne4	5.72583	52.9233	1.3E-11	9.4E-11	2.5E-07	ENSRNO	protein_coding
Nudt16	-6.2718	-77.266	9.9E-14	8.4E-13	1.9E-09	ENSRNO	protein_coding
Col6a6	4.46726	22.1197	3.4E-13	2.8E-12	6.4E-09	ENSRNO	protein_coding
Sema3f	1.42942	2.69338	9.6E-45	2.7E-43	1.8E-40	ENSRNO	protein_coding
Mst1r	6.39346	84.0664	1.3E-23	1.8E-22	2.4E-19	ENSRNO	protein_coding
Fam212a	-6.6256	-98.744	0.00698	0.01637	1	ENSRNO	protein_coding
Bsn	1.49548	2.81957	0.00203	0.00535	1	ENSRNO	protein_coding
Gpx1	1.565	2.95877	9.4E-52	3.1E-50	1.8E-47	ENSRNO	protein_coding
Klhdc8b	-2.4558	-5.4862	4.6E-30	8.5E-29	8.8E-26	ENSRNO	protein_coding
P4htm	-2.6155	-6.1283	1.4E-05	5.3E-05	0.26343	ENSRNO	protein_coding
Celsr3	-1.5456	-2.9192	0.01906	0.03977	1	ENSRNO	protein_coding
Col7a1	-1.4267	-2.6884	0.00013	0.00042	1	ENSRNO	protein_coding
Plxnb1	-4.8479	-28.799	5.8E-58	2.2E-56	1.1E-53	ENSRNO	protein_coding
Ngp	3.74783	13.4341	9.4E-09	5.3E-08	0.00018	ENSRNO	protein_coding
Als2cl	1.47596	2.78168	5E-19	5.8E-18	9.6E-15	ENSRNO	protein_coding
Rtp3	2.9238	7.58844	0.0001	0.00034	1	ENSRNO	protein_coding
Dclk3	-3.9947	-15.942	0.00328	0.00828	1	ENSRNO	protein_coding
Stac	-3.2403	-9.4498	3.3E-30	6.2E-29	6.3E-26	ENSRNO	protein_coding
Susd5	-5.0646	-33.466	2.2E-95	1.5E-93	4.2E-91	ENSRNO	protein_coding
Crtapl1_1	-3.6768	-12.789	9E-194	2E-191	2E-189	ENSRNO	protein_coding
Tgfbr2	-1.3586	-2.5644	2.7E-37	6.2E-36	5.2E-33	ENSRNO	protein_coding
AABR070	5.64783	50.1379	0.02256	0.04619	1	ENSRNO	lincRNA
Cmc1	2.48848	5.61185	2.7E-13	2.2E-12	5E-09	ENSRNO	protein_coding
Eomes	4.30137	19.717	1.3E-20	1.6E-19	2.5E-16	ENSRNO	protein_coding
Itga9	6.68827	103.127	0.00504	0.01222	1	ENSRNO	protein_coding
Scn5a	3.44704	10.9059	1.2E-08	6.8E-08	0.00024	ENSRNO	protein_coding
Scn11a	6.25142	76.1842	1.4E-06	6.2E-06	0.02706	ENSRNO	protein_coding
Entpd3	-3.0629	-8.3567	7.1E-20	8.4E-19	1.3E-15	ENSRNO	protein_coding
Cck	6.47442	88.9189	1.3E-10	8.7E-10	2.5E-06	ENSRNO	protein_coding
Klhl40	1.91379	3.76799	8.1E-10	5E-09	1.5E-05	ENSRNO	protein_coding
AABR070	2.00535	4.01487	0.00014	0.00045	1	ENSRNO	lincRNA
Zfp167	2.15515	4.45416	7.9E-10	4.9E-09	1.5E-05	ENSRNO	protein_coding
Ccr1	-2.8322	-7.1216	2.8E-10	1.8E-09	5.3E-06	ENSRNO	protein_coding
Satb1	-6.3453	-81.306	1.1E-22	1.5E-21	2.2E-18	ENSRNO	protein_coding
Sult1c2_1	-1.9887	-3.9688	0.0022	0.00574	1	ENSRNO	protein_coding
C3	-1.7054	-3.2612	4E-09	2.3E-08	7.7E-05	ENSRNO	protein_coding
Tubb4a	-1.4991	-2.8267	0.00545	0.01311	1	ENSRNO	protein_coding
Sema6b	-1.6474	-3.1326	1.3E-05	5E-05	0.24537	ENSRNO	protein_coding
Plin4	-1.7103	-3.2724	0.00101	0.00283	1	ENSRNO	protein_coding
Stap2	2.83652	7.14297	1.5E-16	1.6E-15	2.9E-12	ENSRNO	protein_coding
Rftn1	-7.6551	-201.56	3.5E-78	1.8E-76	6.7E-74	ENSRNO	protein_coding
Kif6	-8.3379	-323.57	0.00057	0.00166	1	ENSRNO	protein_coding
Daam2	-8.0249	-260.45	2.1E-10	1.4E-09	3.9E-06	ENSRNO	protein_coding
Mocs1	-1.7806	-3.4357	2.1E-15	1.9E-14	3.9E-11	ENSRNO	protein_coding

Trem3	5.89054	59.3239	0.01506	0.03236	1	ENSRNO	protein_coding
RGD1561	3.64899	12.5446	3.3E-20	3.9E-19	6.2E-16	ENSRNO	protein_coding
LOC1009	2.61561	6.12881	0.00234	0.00608	1	ENSRNO	protein_coding
Guca1b	4.34817	20.3671	0.00146	0.00395	1	ENSRNO	protein_coding
Ptk7	-6.6961	-103.69	2E-167	3E-165	3E-163	ENSRNO	protein_coding
Srf	-1.4931	-2.815	1.9E-47	5.7E-46	3.6E-43	ENSRNO	protein_coding
RGD1562	1.65503	3.14931	0.00046	0.00138	1	ENSRNO	protein_coding
Slc29a1	-1.4761	-2.7819	8E-38	1.9E-36	1.5E-33	ENSRNO	protein_coding
Tcte1	3.20361	9.21258	0.00338	0.00852	1	ENSRNO	protein_coding
Enpp4	-5.4371	-43.326	7.2E-46	2.1E-44	1.4E-41	ENSRNO	protein_coding
Enpp5	4.45621	21.9509	1.5E-55	5.4E-54	2.9E-51	ENSRNO	protein_coding
Rcan2	-3.3051	-9.8843	5.4E-73	2.6E-71	1E-68	ENSRNO	protein_coding
Cyp39a1	-2.1404	-4.4089	3.1E-08	1.6E-07	0.00058	ENSRNO	protein_coding
Slc25a27	-2.4927	-5.6282	2.8E-14	2.5E-13	5.3E-10	ENSRNO	protein_coding
Pla2g7	4.41172	21.2843	6.1E-07	2.8E-06	0.01152	ENSRNO	protein_coding
Adgrf5	4.34498	20.3221	5.3E-25	8E-24	1E-20	ENSRNO	protein_coding
Tnfrsf21	3.16208	8.95122	1.3E-69	6E-68	2.5E-65	ENSRNO	protein_coding
Crisp2	1.3969	2.63336	0.00783	0.01811	1	ENSRNO	protein_coding
Crisp3	-6.8215	-113.1	1E-73	5E-72	2E-69	ENSRNO	protein_coding
AABR070	-2.1813	-4.5357	0.0002	0.00064	1	ENSRNO	protein_coding
Tram2	-1.4852	-2.7996	2.5E-32	5.1E-31	4.8E-28	ENSRNO	protein_coding
Tmem14a	2.27407	4.83685	2E-22	2.7E-21	3.8E-18	ENSRNO	protein_coding
Gsta1	4.80673	27.9878	7.1E-21	9E-20	1.4E-16	ENSRNO	protein_coding
Ogfrl1	-4.1172	-17.354	4.2E-80	2.2E-78	8.1E-76	ENSRNO	protein_coding
AABR070	6.65204	100.569	0.0054	0.01301	1	ENSRNO	pseudogene
Hs6st1	-1.3643	-2.5745	1.7E-39	4.2E-38	3.3E-35	ENSRNO	protein_coding
Sema4c	-1.4313	-2.697	4E-14	3.5E-13	7.6E-10	ENSRNO	protein_coding
RGD1310	-6.5784	-95.566	4E-224	1E-221	8E-220	ENSRNO	protein_coding
Aff3	-7.3099	-158.67	2E-18	2.2E-17	3.8E-14	ENSRNO	protein_coding
Lonrf2	-6.1015	-68.664	5.3E-27	8.7E-26	1E-22	ENSRNO	protein_coding
Il1rl2	-2.7115	-6.5499	3.2E-10	2.1E-09	6.1E-06	ENSRNO	protein_coding
Il1rl1	-2.6407	-6.2363	2E-141	2E-139	3E-137	ENSRNO	protein_coding
Il18rap	-1.8792	-3.6787	1.9E-63	7.6E-62	3.5E-59	ENSRNO	protein_coding
AABR070	-1.9226	-3.7911	8.1E-16	7.8E-15	1.5E-11	ENSRNO	lincRNA
RGD1305	2.83863	7.15342	6.8E-06	2.7E-05	0.12912	ENSRNO	protein_coding
Col3a1	-4.3707	-20.688	0	0	0	ENSRNO	protein_coding
Col5a2	-2.0321	-4.0901	1E-91	6.4E-90	1.9E-87	ENSRNO	protein_coding
Mfsd6	-1.5616	-2.9518	2E-30	3.8E-29	3.8E-26	ENSRNO	protein_coding
Myo1b	-1.8564	-3.6211	1.2E-69	5.5E-68	2.3E-65	ENSRNO	protein_coding
Sdpr	-1.9716	-3.9219	6.7E-08	3.4E-07	0.00127	ENSRNO	protein_coding
Hecw2	-4.2512	-19.044	1.3E-92	8.8E-91	2.5E-88	ENSRNO	protein_coding
Rftn2	-2.8229	-7.0759	0.00317	0.00803	1	ENSRNO	protein_coding
Aox1	2.47657	5.56574	3.9E-74	1.9E-72	7.4E-70	ENSRNO	protein_coding
Aox2	2.38127	5.20997	6E-13	4.8E-12	1.1E-08	ENSRNO	protein_coding
Als2cr12	2.84464	7.18325	0.00057	0.00168	1	ENSRNO	protein_coding
Cd28	-2.6833	-6.4232	2.2E-49	6.8E-48	4.2E-45	ENSRNO	protein_coding
Zdbf2	-4.6128	-24.468	6.3E-83	3.5E-81	1.2E-78	ENSRNO	protein_coding
Adam23	-8.4905	-359.67	1.1E-32	2.2E-31	2.1E-28	ENSRNO	protein_coding
Klf7_1	-1.4504	-2.7328	6E-21	7.6E-20	1.1E-16	ENSRNO	protein_coding

Ccnyl1	-1.4965	-2.8215	8.3E-49	2.5E-47	1.6E-44	ENSRNO	protein_coding
Plekhm3	-1.3463	-2.5427	7.8E-06	3.1E-05	0.14865	ENSRNO	protein_coding
Kansl1l	-1.7924	-3.4639	2.8E-58	1.1E-56	5.4E-54	ENSRNO	protein_coding
Cps1	3.53756	11.6121	0.00982	0.02212	1	ENSRNO	protein_coding
Erbb4	3.54014	11.6329	1.8E-31	3.4E-30	3.3E-27	ENSRNO	protein_coding
Ikzf2	-1.7533	-3.3714	3.2E-07	1.5E-06	0.00606	ENSRNO	protein_coding
Abca12	3.68347	12.848	9.3E-06	3.6E-05	0.17734	ENSRNO	protein_coding
Tmem169	-3.2959	-9.8214	4.4E-06	1.8E-05	0.08276	ENSRNO	protein_coding
Vil1	1.88248	3.68709	0.00827	0.01898	1	ENSRNO	protein_coding
Stk36	-1.527	-2.8818	3.5E-16	3.4E-15	6.6E-12	ENSRNO	protein_coding
Cyp27a1	-5.1874	-36.438	1.1E-26	1.7E-25	2E-22	ENSRNO	protein_coding
Prkag3	-2.6527	-6.2884	9.5E-07	4.2E-06	0.0181	ENSRNO	protein_coding
Glb1l	-1.3556	-2.559	1.2E-37	2.7E-36	2.2E-33	ENSRNO	protein_coding
Ptprn	-4.8303	-28.449	6E-151	8E-149	1E-146	ENSRNO	protein_coding
Tmem198	-1.3795	-2.6017	0.00052	0.00155	1	ENSRNO	protein_coding
Slc4a3	-3.3355	-10.095	1.1E-63	4.4E-62	2E-59	ENSRNO	protein_coding
Serpine2	1.67826	3.20043	4.2E-63	1.7E-61	8E-59	ENSRNO	protein_coding
Dock10	-1.8519	-3.6098	4.1E-12	3.1E-11	7.8E-08	ENSRNO	processed_transcript
Col4a4	-1.957	-3.8825	2.4E-09	1.4E-08	4.5E-05	ENSRNO	protein_coding
Col4a3	-2.2261	-4.6786	1.4E-12	1.1E-11	2.7E-08	ENSRNO	protein_coding
Ccl20	-2.5646	-5.9158	1.9E-11	1.4E-10	3.7E-07	ENSRNO	protein_coding
Daw1	-2.233	-4.7012	0.00364	0.0091	1	ENSRNO	protein_coding
LOC1009	-3.2022	-9.2033	0.00395	0.00978	1	ENSRNO	lincRNA
LOC1009	-5.9393	-61.364	0.02346	0.04773	1	ENSRNO	protein_coding
Dner	-7.4732	-177.69	4E-186	8E-184	8E-182	ENSRNO	protein_coding
Sp100	-1.3668	-2.579	2.7E-17	2.8E-16	5.1E-13	ENSRNO	protein_coding
RGD1563	-2.4911	-5.6221	6.3E-06	2.5E-05	0.12061	ENSRNO	protein_coding
Htr2b	-2.9965	-7.9805	1.3E-08	7.3E-08	0.00025	ENSRNO	protein_coding
B3gnt7	-8.4992	-361.84	1.7E-11	1.2E-10	3.2E-07	ENSRNO	protein_coding
Pde6d	-1.3999	-2.6389	1.9E-19	2.2E-18	3.5E-15	ENSRNO	protein_coding
Efhd1	-4.0297	-16.332	2.2E-18	2.4E-17	4.1E-14	ENSRNO	protein_coding
Ngef	1.92002	3.78427	0.00041	0.00123	1	ENSRNO	protein_coding
Neu2	2.71209	6.55271	0.00735	0.01713	1	ENSRNO	protein_coding
AABR070	-2.5948	-6.041	1.4E-29	2.6E-28	2.7E-25	ENSRNO	pseudogene
Ackr3	-4.9571	-31.062	1.4E-66	6.1E-65	2.6E-62	ENSRNO	protein_coding
Col6a3	-4.0974	-17.117	0	0	0	ENSRNO	processed_transcript
Mlph	2.50612	5.68091	0.00517	0.01251	1	ENSRNO	protein_coding
Espnl	-2.0391	-4.1098	3E-11	2.1E-10	5.6E-07	ENSRNO	protein_coding
Klhl30	-1.4651	-2.7609	2.1E-45	5.9E-44	3.9E-41	ENSRNO	protein_coding
Ankmy1	-1.4569	-2.7451	0.01297	0.02837	1	ENSRNO	protein_coding
LOC1003	-2.3331	-5.0387	0.00058	0.0017	1	ENSRNO	protein_coding
Farp2	-1.4521	-2.736	2.6E-41	6.8E-40	5E-37	ENSRNO	protein_coding
Slco4c1	-3.5355	-11.595	0.00018	0.00058	1	ENSRNO	protein_coding
RGD1560	-3.2023	-9.2044	0.00364	0.00909	1	ENSRNO	protein_coding
Nudt12	-1.8122	-3.5118	1.6E-28	2.9E-27	3.1E-24	ENSRNO	protein_coding
AABR070	-1.4232	-2.6818	1.4E-05	5.3E-05	0.26521	ENSRNO	pseudogene
Efna5	-1.4588	-2.7489	5.1E-11	3.6E-10	9.8E-07	ENSRNO	protein_coding
Man2a1	-1.731	-3.3195	5.5E-66	2.4E-64	1E-61	ENSRNO	protein_coding
Arhgap28	-6.7374	-106.7	1.1E-19	1.3E-18	2E-15	ENSRNO	protein_coding

RGD1563	2.37334	5.1814	6.1E-05	0.00021	1	ENSRNO	protein_coding
Epb41l3	-2.3995	-5.2763	7.4E-08	3.8E-07	0.00141	ENSRNO	protein_coding
Emilin2	-7.6394	-199.38	2.3E-97	1.7E-95	4.4E-93	ENSRNO	protein_coding
Yes1	-1.3777	-2.5985	3E-21	3.8E-20	5.6E-17	ENSRNO	protein_coding
Zfp300	-7.2369	-150.84	0.00295	0.00753	1	ENSRNO	protein_coding
Zfp182	-2.631	-6.1945	0.00439	0.0108	1	ENSRNO	protein_coding
Syn1	-4.6518	-25.139	4.1E-27	6.8E-26	7.8E-23	ENSRNO	protein_coding
Efhc2	5.5516	46.9027	4E-180	7E-178	7E-176	ENSRNO	protein_coding
LOC1003	6.38947	83.8341	3E-104	3E-102	6E-100	ENSRNO	protein_coding
Ndp	3.78045	13.7414	0.00501	0.01216	1	ENSRNO	protein_coding
LOC1009	6.03419	65.5348	3.5E-06	1.4E-05	0.06634	ENSRNO	protein_coding
Cask	2.28675	4.87957	0.00611	0.01454	1	ENSRNO	protein_coding
Srpx	-5.4569	-43.921	1.2E-57	4.5E-56	2.3E-53	ENSRNO	protein_coding
Lancl3	5.83892	57.2387	0.0147	0.03168	1	ENSRNO	protein_coding
Porcn	-2.4883	-5.6113	3.8E-50	1.2E-48	7.2E-46	ENSRNO	protein_coding
Wdr13	-1.519	-2.866	9.6E-21	1.2E-19	1.8E-16	ENSRNO	protein_coding
Was	1.98888	3.96929	9.1E-09	5.1E-08	0.00017	ENSRNO	protein_coding
Glod5	3.70389	13.0311	0.00627	0.01488	1	ENSRNO	protein_coding
Kcnd1	3.85379	14.4579	5.4E-25	8.2E-24	1E-20	ENSRNO	protein_coding
Cacna1f	3.80778	14.0041	5E-06	2E-05	0.09568	ENSRNO	protein_coding
Usp27x	1.44545	2.72348	3.5E-13	2.9E-12	6.7E-09	ENSRNO	protein_coding
Shroom4	-6.2526	-76.249	2.5E-50	7.7E-49	4.7E-46	ENSRNO	protein_coding
LOC1009	-1.5915	-3.0137	0.01103	0.02452	1	ENSRNO	protein_coding
Spin2a	-5.8883	-59.234	0.02081	0.04296	1	ENSRNO	protein_coding
Usp51	7.57199	190.281	0.00137	0.00373	1	ENSRNO	protein_coding
AABR070	5.69274	51.7232	0.01872	0.03915	1	ENSRNO	protein_coding
Wnk3	-8.8788	-470.74	5.6E-51	1.8E-49	1.1E-46	ENSRNO	protein_coding
Gpr173_1	-6.3569	-81.963	1.5E-10	1E-09	2.9E-06	ENSRNO	protein_coding
Shroom2	-6.1411	-70.574	1.8E-24	2.6E-23	3.4E-20	ENSRNO	protein_coding
AABR070	3.88531	14.7773	9.8E-14	8.3E-13	1.9E-09	ENSRNO	lincRNA
Rab9a	-2.7434	-6.6965	7.3E-37	1.6E-35	1.4E-32	ENSRNO	protein_coding
Gpm6b	5.83954	57.2635	3.5E-12	2.6E-11	6.6E-08	ENSRNO	protein_coding
Asb9	3.95381	15.4959	1.3E-05	5.1E-05	0.25323	ENSRNO	protein_coding
Vegfd	2.12327	4.3568	8E-11	5.5E-10	1.5E-06	ENSRNO	protein_coding
Pir	1.46822	2.7668	7.9E-45	2.2E-43	1.5E-40	ENSRNO	protein_coding
Bmx	5.93344	61.1145	2.4E-09	1.4E-08	4.5E-05	ENSRNO	protein_coding
Grpr	-2.9453	-7.7025	2.6E-06	1.1E-05	0.04935	ENSRNO	protein_coding
Ctps2	5.1681	35.9546	1.2E-91	7.9E-90	2.4E-87	ENSRNO	protein_coding
Reps2	-2.7308	-6.6381	0.02222	0.04554	1	ENSRNO	protein_coding
Scml2	2.01352	4.03766	6.5E-26	1E-24	1.2E-21	ENSRNO	protein_coding
Sh3kbp1	1.40084	2.64056	1.3E-06	5.9E-06	0.02548	ENSRNO	protein_coding
Phex	-2.2732	-4.8341	3E-07	1.4E-06	0.00578	ENSRNO	protein_coding
RGD1563	3.7884	13.8173	0.00635	0.01504	1	ENSRNO	protein_coding
AABR070	2.40553	5.2983	0.02453	0.04968	1	ENSRNO	protein_coding
Fam47a_	6.32669	80.2644	0.00801	0.01845	1	ENSRNO	processed_transcript
Mageb16	2.82657	7.09385	6.3E-09	3.6E-08	0.00012	ENSRNO	protein_coding
AC11235	6.76059	108.428	0.00446	0.01095	1	ENSRNO	pseudogene
Mageb4	2.66828	6.3567	7E-17	7.2E-16	1.3E-12	ENSRNO	protein_coding
Mageb18	8.24301	302.966	0.00049	0.00146	1	ENSRNO	protein_coding

Pcyt1b	3.16625	8.97712	3.3E-21	4.2E-20	6.3E-17	ENSRNO	protein_coding
RGD1564	4.74109	26.7429	6.5E-16	6.2E-15	1.2E-11	ENSRNO	lincRNA
Rn50_X_(	5.25166	38.0984	8.7E-05	0.00029	1	ENSRNO	lincRNA
Rn50_X_(	6.66112	101.204	1.9E-07	9.4E-07	0.00366	ENSRNO	lincRNA
Arhgef9	-3.2639	-9.606	1.9E-22	2.6E-21	3.7E-18	ENSRNO	processed_transcript
Zc3h12b	-3.6378	-12.448	1.8E-05	6.7E-05	0.33643	ENSRNO	protein_coding
Rn50_X_(	2.74428	6.70054	1.1E-08	6E-08	0.00021	ENSRNO	lincRNA
Heph	2.81786	7.05115	5E-159	7E-157	9E-155	ENSRNO	protein_coding
Gpr165	8.14172	282.424	0.00057	0.00167	1	ENSRNO	protein_coding
AABR070	4.16964	17.9964	0.00201	0.00529	1	ENSRNO	protein_coding
Eda2r	6.23167	75.1483	0.00893	0.02035	1	ENSRNO	protein_coding
Stard8	-5.0186	-32.416	3.7E-16	3.6E-15	7E-12	ENSRNO	protein_coding
Eda	-4.5036	-22.684	1.7E-28	2.9E-27	3.2E-24	ENSRNO	protein_coding
P2ry4	2.87993	7.36115	4E-05	0.00014	0.7661	ENSRNO	protein_coding
Tex11	-8.0606	-266.97	1E-29	1.9E-28	2E-25	ENSRNO	protein_coding
Slc7a3	-1.9884	-3.9679	0.004	0.0099	1	ENSRNO	protein_coding
Il2rg	3.05292	8.29889	1.4E-18	1.6E-17	2.7E-14	ENSRNO	protein_coding
LOC1003	1.59466	3.02023	2.5E-52	8.4E-51	4.8E-48	ENSRNO	protein_coding
LOC1025	1.33098	2.51574	3.8E-38	9E-37	7.3E-34	ENSRNO	protein_coding
Nhsl2	-2.474	-5.5558	8E-30	1.5E-28	1.5E-25	ENSRNO	processed_transcript
Dmrtc1c1	2.11669	4.33696	0.02365	0.04809	1	ENSRNO	protein_coding
Rn50_X_(	2.24492	4.74012	1.2E-08	6.4E-08	0.00022	ENSRNO	processed_transcript
Tsx	5.55477	47.0057	0.02127	0.0438	1	ENSRNO	protein_coding
RGD1561	-3.9354	-15.3	4E-60	1.5E-58	7.5E-56	ENSRNO	protein_coding
Tlr13	3.56222	11.8123	1.1E-17	1.2E-16	2.1E-13	ENSRNO	protein_coding
Taf9b	-6.1636	-71.684	9.8E-25	1.5E-23	1.9E-20	ENSRNO	protein_coding
Zcchc5	5.5546	47.0003	0.02042	0.04223	1	ENSRNO	protein_coding
RGD1560	-1.3773	-2.5978	2.9E-39	7E-38	5.5E-35	ENSRNO	protein_coding
Rps6ka6	-7.6325	-198.43	4.7E-32	9.3E-31	8.9E-28	ENSRNO	protein_coding
AABR070	3.94367	15.3873	0.00021	0.00065	1	ENSRNO	pseudogene
Nap1l3	6.81292	112.433	1.1E-27	1.9E-26	2.2E-23	ENSRNO	protein_coding
Pcdh19	-8.3865	-334.64	3.5E-18	3.9E-17	6.7E-14	ENSRNO	protein_coding
Srpx2	-4.158	-17.852	0	0	0	ENSRNO	protein_coding
Tmem35	-5.9433	-61.535	2.7E-25	4.1E-24	5.1E-21	ENSRNO	protein_coding
Drp2	-1.6748	-3.1928	0.01969	0.04094	1	ENSRNO	protein_coding
Taf7l	1.54052	2.90899	0.0222	0.04552	1	ENSRNO	protein_coding
Armcx4	-3.4245	-10.737	4E-175	7E-173	7E-171	ENSRNO	protein_coding
Armcx6	-4.2416	-18.917	0.00188	0.00498	1	ENSRNO	protein_coding
Armcx2	-5.0621	-33.408	1.9E-30	3.6E-29	3.7E-26	ENSRNO	protein_coding
Zmat1	1.54369	2.91539	1.5E-12	1.1E-11	2.8E-08	ENSRNO	protein_coding
Nxf7	3.92812	15.2223	2.2E-53	7.5E-52	4.2E-49	ENSRNO	protein_coding
Gprasp2	2.06579	4.18662	2.2E-22	3E-21	4.2E-18	ENSRNO	protein_coding
Bhlhb9	5.06906	33.5691	2.6E-74	1.3E-72	4.9E-70	ENSRNO	protein_coding
Nxf3	5.18264	36.3188	3.1E-29	5.5E-28	5.8E-25	ENSRNO	protein_coding
Tceal1	2.98123	7.89659	6.6E-21	8.3E-20	1.3E-16	ENSRNO	protein_coding
Rab9b	2.98164	7.89886	6.5E-05	0.00022	1	ENSRNO	protein_coding
Fam199x	1.38558	2.61276	1.5E-09	9.1E-09	2.9E-05	ENSRNO	protein_coding
Mum1l1	-1.4689	-2.7682	1.2E-16	1.2E-15	2.3E-12	ENSRNO	protein_coding
Col4a6	-4.0893	-17.021	2.9E-09	1.7E-08	5.5E-05	ENSRNO	protein_coding

Col4a5	-1.3678	-2.5807	2E-40	5.1E-39	3.9E-36	ENSRNO	protein_coding
Pak3	-5.5138	-45.691	1E-86	6.1E-85	1.9E-82	ENSRNO	protein_coding
AABR070	-1.9052	-3.7455	1.8E-10	1.2E-09	3.3E-06	ENSRNO	protein_coding
Klhl13	-1.5948	-3.0205	2.9E-20	3.5E-19	5.5E-16	ENSRNO	protein_coding
Dock11	-6.6959	-103.68	9.9E-47	2.9E-45	1.9E-42	ENSRNO	protein_coding
Zcchc12	-3.2683	-9.635	0.00331	0.00836	1	ENSRNO	protein_coding
ENSRNO	6.5793	95.624	0.00576	0.01379	1	ENSRNO	protein_coding
Slc25a43	-5.5281	-46.145	7E-18	7.6E-17	1.3E-13	ENSRNO	protein_coding
LOC1083	2.37918	5.20241	0.00882	0.0201	1	ENSRNO	protein_coding
Rhox2	1.88335	3.68932	0.0037	0.00922	1	ENSRNO	protein_coding
Smarca1	-5.4305	-43.125	5.7E-46	1.6E-44	1.1E-41	ENSRNO	protein_coding
AABR070	-1.6417	-3.1203	0.00339	0.00855	1	ENSRNO	protein_coding
Stk26	-2.7864	-6.8992	1.5E-37	3.5E-36	2.9E-33	ENSRNO	protein_coding
Hs6st2	-5.9374	-61.282	2.6E-92	1.7E-90	4.9E-88	ENSRNO	protein_coding
MGC1144	8.80155	446.2	3E-58	1.1E-56	5.6E-54	ENSRNO	protein_coding
Gpc4	5.45112	43.7472	4.7E-13	3.8E-12	8.9E-09	ENSRNO	protein_coding
Zic3	-4.8383	-28.607	0.00031	0.00095	1	ENSRNO	protein_coding
Magea11	2.42633	5.37526	5E-115	4E-113	9E-111	ENSRNO	protein_coding
Mcf2	-1.5384	-2.9047	6.1E-05	0.00021	1	ENSRNO	protein_coding
Atp11c	-2.9104	-7.5183	1.7E-57	6.1E-56	3.1E-53	ENSRNO	protein_coding
NEWGEN	4.65941	25.271	7.9E-06	3.1E-05	0.15028	ENSRNO	protein_coding
LOC1025	7.68649	205.998	0.0012	0.00331	1	ENSRNO	protein_coding
MGC1144	4.23632	18.8477	2.3E-13	1.9E-12	4.5E-09	ENSRNO	protein_coding
AABR070	-1.4102	-2.6578	1.2E-09	7.1E-09	2.2E-05	ENSRNO	lincRNA
Gabra3	5.7089	52.3059	3.5E-08	1.8E-07	0.00066	ENSRNO	protein_coding
Cetn2	1.84767	3.59918	9.4E-36	2E-34	1.8E-31	ENSRNO	protein_coding
Xlr4a	1.72838	3.31356	1.1E-29	2E-28	2E-25	ENSRNO	protein_coding
LOC1036	1.92779	3.80471	4.9E-41	1.2E-39	9.2E-37	ENSRNO	protein_coding
LOC1009	9.8134	899.764	6.8E-05	0.00023	1	ENSRNO	protein_coding
LOC1025	-4.0452	-16.509	9.7E-10	6E-09	1.9E-05	ENSRNO	protein_coding
LOC1083	-4.7996	-27.851	2.1E-09	1.2E-08	3.9E-05	ENSRNO	protein_coding
Arhgap4	1.32266	2.50127	1.6E-06	7E-06	0.03096	ENSRNO	protein_coding
L1cam	2.06667	4.18917	1.9E-05	7.3E-05	0.36862	ENSRNO	protein_coding
Pdzd4	-2.2273	-4.6825	1.2E-25	1.8E-24	2.2E-21	ENSRNO	protein_coding
AABR070	1.48953	2.80797	0.00018	0.00058	1	ENSRNO	lincRNA
Haus7	1.45455	2.74072	6.6E-45	1.9E-43	1.3E-40	ENSRNO	protein_coding
Plac1	-4.5482	-23.396	0.00074	0.00212	1	ENSRNO	protein_coding
LOC1025	-3.5223	-11.49	0.01298	0.02838	1	ENSRNO	protein_coding
AABR070	-1.5946	-3.0201	2.4E-05	9E-05	0.46273	ENSRNO	lincRNA
Fhl1	-1.9509	-3.8663	3.2E-80	1.7E-78	6E-76	ENSRNO	protein_coding
Arhgef6	-7.3194	-159.72	4E-283	2E-280	7E-279	ENSRNO	protein_coding

Table S2. Genes in enriched pathways in KMM cells compared to MM cells analyzed using the GOBP database

Cutoffs for differentially expressed genes: Fold change>2.5, P-value<0.05, FDR<0.05

GO:0007155~cell adhesion			
**Gene**	**Fold change**	**P-value**	**FDR**
RELN	448.6971861	0.000177033	0.000568086
NFASC	163.3596957	1.30613E-23	1.86314E-22
DSG2	130.1397554	0	0
ITGA9	103.1265509	0.005039992	0.01221539
CDH2	86.62228472	1.36E-156	2.0064E-154
CD177	78.71123021	0.008724897	0.019909812
ADAM32	70.50970855	5.15562E-42	1.36332E-40
JUP	55.18666521	0	0
CLDN4	50.63260861	1.31769E-05	5.06073E-05
POSTN	49.61333295	0	0
PCDHGB7	40.14282482	6.5435E-10	4.12012E-09
F11R	33.44203593	5.22541E-85	2.98915E-83
PCDHGA3	29.25187868	6.50094E-18	7.07198E-17
EPHA8	23.67529332	0.000628559	0.001827645
PCDHGB5	23.15417775	1.01795E-06	4.5255E-06
PCDHGA10	21.73481622	2.76163E-11	1.95147E-10
NPHS1	21.59443181	2.91502E-16	2.88211E-15
PCDHGA5	21.4441567	7.77471E-08	3.9503E-07
PDZD2	20.83360551	6.07014E-16	5.86373E-15
PTK2B	20.25052503	3.1435E-267	1.3912E-264
PCDHGA2	18.74068902	1.36992E-25	2.12933E-24
PCDHGA1	17.63674535	1.68208E-51	5.41938E-50
FLG2	17.28849207	0.002064615	0.005427116
PCDHGA8	17.07965174	1.41257E-05	5.40045E-05
PCDHGA11	14.79645705	1.01967E-09	6.28852E-09
EMP2	14.70602773	2.619E-249	8.8093E-247
CD24	12.56412796	5.6624E-258	2.3227E-255
PCDHGB4	12.04107695	2.32538E-05	8.58768E-05
PCDHGA9	10.23597663	2.81625E-07	1.34365E-06
PCDH20	9.491614962	1.31641E-47	3.93941E-46
NRCAM	8.774218487	0.023895826	0.048553824
PCDHB5	8.159366905	3.4128E-13	2.78624E-12
ITGAD	7.400109986	0.003708433	0.009250731
VCAM1	7.296516753	5.50554E-19	6.27744E-18
PODXL	7.151921283	0.012012755	0.026474973
ADGRG1	6.740583999	0.012860255	0.028168297
PCDHGA4	6.106433923	5.08597E-05	0.000177792
PCDHGB6	6.032706049	1.6882E-06	7.28752E-06
PCDHGA6	5.43122508	3.22757E-05	0.000116384
PCDH15	5.131345621	0.001749721	0.004662781

CHL1	5.041978365	0.023980328	0.048707868
LGALS3BP	4.306964498	2.10722E-93	1.43479E-91
LOC108348201	4.273011071	1.42384E-29	2.56658E-28
NCAN	4.078579335	0.011736679	0.025941316
CCDC141	4.054489226	0.009316693	0.021102781
SULF1	3.977067432	8.74654E-77	4.39103E-75
FES	3.896034021	0.016454612	0.034922171
AOC3	3.797827942	0.004771268	0.011632819
APP	3.788256992	1.12742E-79	5.9253E-78
CDHR2	3.764088019	0.015311967	0.03281245
CX3CL1	3.63220813	3.03893E-09	1.79707E-08
DPP4	3.548465042	0.000971472	0.002729404
ITGA3	3.357115344	2.82153E-68	1.27219E-66
PCDHB19	3.192405391	4.659E-06	1.90413E-05
CDH16	3.082672495	0.015519062	0.033193385
TNR	2.876946007	0.005583217	0.013419828
PCDHB21	2.789136977	5.10543E-07	2.36277E-06
PCDHB12	2.761508184	2.79212E-06	1.17483E-05
EFS	2.628154609	2.34252E-09	1.39803E-08
CEACAM1	2.550409701	4.36808E-19	5.01108E-18
PCDHB22	2.520509198	3.07545E-05	0.00011128
NEO1	-2.547551275	3.93553E-40	9.76221E-39
RHOB	-2.611616323	2.03595E-41	5.28404E-40
FARP2	-2.736015159	2.62494E-41	6.78131E-40
OMD	-2.916310838	1.46865E-05	5.5996E-05
HAPLN4	-3.397937694	4.85084E-31	9.2496E-30
MYH10	-3.449550031	4.72243E-70	2.17594E-68
MFGE8	-4.21327375	1.2248E-95	8.58282E-94
PMP22	-5.256091097	3.2239E-110	2.8537E-108
SIGLEC10	-5.473491676	3.24261E-27	5.41901E-26
PARVB	-6.917326117	2.45067E-37	5.60754E-36
CD9	-6.973773329	7.8017E-157	1.1611E-154
ICAM5	-8.597297017	5.24952E-20	6.30618E-19
FBLN5	-8.94893209	8.8813E-153	1.2552E-150
NEXN	-9.648745477	7.66994E-88	4.69066E-86
PCDHB10	-9.977450219	0.018790737	0.039291635
SRC	-10.28414552	1.1583E-182	2.1407E-180
ITGB3	-12.59597777	0.000144452	0.000470175
THBS2	-12.67524431	4.4846E-277	2.1549E-274
ACAN	-14.03006631	4.6235E-297	2.8799E-294
NRXN2	-14.80101337	0.000274132	0.000851089
CYP1B1	-20.28604261	0	0
CADM1	-22.0022104	0	0
ACKR3	-31.06222833	1.38935E-66	6.13283E-65
ADA	-32.17015327	3.04304E-34	6.42938E-33
SUSD5	-33.46564099	2.19788E-95	1.53377E-93

SDC3	-34.06501019	0	0
CADM4	-40.03424109	2.1995E-25	3.39681E-24
EDIL3	-46.92076762	1.09726E-24	1.63018E-23
JAM3	-70.01632755	2.18179E-24	3.1935E-23
PDPN	-74.98670597	4.44924E-25	6.75336E-24
TNFAIP6	-75.5792774	1.58274E-06	6.85161E-06
COMP	-90.83052052	7.94545E-19	8.98028E-18
OPCML	-92.81571999	0.007275139	0.016979363
PTK7	-103.6878249	1.7944E-167	2.874E-165
CGREF1	-106.8087168	1.99325E-89	1.24619E-87
NLGN1	-126.0877023	1.63983E-54	5.59404E-53
LAMA4	-133.164988	4.5209E-127	4.7225E-125
PCDH9	-175.4948837	4.90504E-09	2.83092E-08
PCDH7	-192.5527824	0.001776355	0.004729261
PCDH19	-334.6425317	3.54229E-18	3.8912E-17
NTM	-427.382058	5.4578E-116	5.0712E-114
SVEP1	-593.2849125	0.000125155	0.000412233

GO:0016477~cell migration

Gene	Fold change	P-value	FDR
RELN	448.6971861	0.000177033	0.000568086
PIK3CG	343.8651175	0.000355495	0.00108036
KDR	128.8956701	0.003114572	0.007910128
CDH2	86.62228472	1.36E-156	2.0064E-154
MST1R	84.06641487	1.25205E-23	1.78752E-22
VAV3	65.24318394	3.43416E-65	1.47713E-63
JUP	55.18666521	0	0
GPC4	43.74721296	4.69199E-13	3.78283E-12
NCKAP1L	23.08861958	1.5624E-261	6.569E-259
LAMB3	21.83553268	0	0
MERTK	18.87064204	8.90091E-14	7.57186E-13
ITGBL1	16.06912672	1.07629E-72	5.12777E-71
EMP2	14.70602773	2.619E-249	8.8093E-247
FMNL1	13.03182758	0.00667983	0.015742907
CD24	12.56412796	5.6624E-258	2.3227E-255
NRG1	11.78979842	8.3204E-99	6.30327E-97
ERBB4	11.63291009	1.75241E-31	3.40716E-30
FLT1	11.31314812	5.24162E-14	4.52766E-13
ERG	9.362259254	3.99655E-42	1.06016E-40
IGFBP6	8.001690108	1.96589E-54	6.67927E-53
LCP1	7.798813791	7.65708E-63	3.10305E-61
PODXL	7.151921283	0.012012755	0.026474973
ITGB7	6.814517514	0.000454856	0.001357064
ADGRG1	6.740583999	0.012860255	0.028168297
FSCN2	6.155085563	2.7898E-05	0.000101798
PIK3C2B	5.428755208	2.13069E-38	5.09004E-37

PSTPIP2	4.810150868	2.67996E-07	1.2819E-06
SIRPA	4.490264887	1.80857E-87	1.0902E-85
L1CAM	4.189172955	1.93827E-05	7.25684E-05
TNFAIP3	4.014531502	4.37593E-33	8.97492E-32
KCTD13	3.922795372	8.55333E-29	1.50156E-27
TGFB1	3.239683624	2.88078E-23	4.0543E-22
LAMA5	3.071812695	7.87142E-58	2.92233E-56
SH3KBP1	2.640559161	1.33964E-06	5.8718E-06
EFS	2.628154609	2.34252E-09	1.39803E-08
SNAI2	-2.531268578	6.72592E-26	1.06413E-24
STRIP2	-2.537116808	0.000123538	0.000407466
SNAI1	-2.589992501	1.40879E-16	1.42301E-15
RHOB	-2.611616323	2.03595E-41	5.28404E-40
CARMIL1	-2.824228288	5.64576E-46	1.62865E-44
RND3	-3.064209445	9.90664E-57	3.59849E-55
TYRO3	-3.104432686	4.88753E-41	1.24732E-39
LURAP1	-3.163075471	3.98454E-06	1.64487E-05
EPHB3	-3.178922057	1.77951E-37	4.0885E-36
PDGFRB	-3.278026165	9.87446E-62	3.93528E-60
VAV2	-3.285613086	3.8587E-48	1.16719E-46
THBS1	-3.298804306	1.70196E-66	7.47353E-65
FUT8	-3.391344208	2.32853E-64	9.74161E-63
RHOBTB3	-3.415226711	1.3503E-62	5.43285E-61
MMP2	-3.847672007	0.000699408	0.002018992
TGFB2	-4.020839745	1.27254E-85	7.32933E-84
NTN4	-4.425011048	3.64101E-11	2.55143E-10
PRKCZ	-4.692663802	1.69505E-09	1.0225E-08
CLN3	-5.073324843	3.04227E-14	2.67459E-13
EMILIN1	-5.271134927	4.41E-104	3.6356E-102
FMNL2	-6.137872728	3.84777E-81	2.08748E-79
BDKRB1	-6.139780517	3.24337E-08	1.71964E-07
PAK1	-7.323128553	7.6741E-148	1.0005E-145
SRGAP1	-7.500293373	5.18459E-18	5.6583E-17
ADAMTS12	-7.794156838	1.4102E-103	1.1569E-101
SRC	-10.28414552	1.1583E-182	2.1407E-180
PIK3CD	-10.47677089	5.389E-182	9.8513E-180
CORO6	-21.64927327	1.98931E-26	3.21385E-25
SDC3	-34.06501019	0	0
EPHA3	-35.37988868	1.38668E-13	1.16489E-12
ENG	-58.85916311	2.9757E-243	9.099E-241
JAM3	-70.01632755	2.18179E-24	3.1935E-23
SHROOM2	-70.57422686	1.76452E-24	2.60312E-23
PTK7	-103.6878249	1.7944E-167	2.874E-165
CXCL12	-119.2246393	0	0
RHOJ	-158.9434365	1.47903E-90	9.38654E-89
ITGB8	-224.6733022	7.34897E-10	4.60488E-09

FSCN1	-227.8487598	1.9086E-166	3.0282E-164
GFRA1	-394.6276082	3.98466E-34	8.38724E-33

GO:0007411~axon guidance

Gene	Fold change	P-value	FDR
RELN	448.6971861	0.000177033	0.000568086
NFASC	163.3596957	1.30613E-23	1.86314E-22
PLXNA4	67.4716477	0.010910958	0.024287198
CRMP1	44.21273402	1.44492E-10	9.63927E-10
EPHB2	23.89713303	1.56057E-29	2.79507E-28
EPHA8	23.67529332	0.000628559	0.001827645
GAP43	13.74206381	0.000359821	0.001092323
LGR6	9.021966616	8.08433E-10	5.0375E-09
NRCAM	8.774218487	0.023895826	0.048553824
SEMA3E	8.549562274	5.53185E-44	1.51769E-42
EDNRA	6.151610242	5.21565E-75	2.59517E-73
CHL1	5.041978365	0.023980328	0.048707868
SEMA4D	5.028204743	0.022900879	0.046775199
L1CAM	4.189172955	1.93827E-05	7.25684E-05
CCDC141	4.054489226	0.009316693	0.021102781
LAMA5	3.071812695	7.87142E-58	2.92233E-56
NECTIN1	2.786745038	4.15322E-46	1.20222E-44
SEMA3F	2.693377241	9.58821E-45	2.67865E-43
NTN3	2.677791664	0.000366207	0.001111106
DPYSL5	2.644425488	1.61969E-11	1.16359E-10
GATA3	2.585040044	1.1389E-15	1.08337E-14
NEO1	-2.547551275	3.93553E-40	9.76221E-39
SEMA4C	-2.696981084	3.97414E-14	3.47386E-13
EFNA5	-2.748861062	5.1448E-11	3.571E-10
SEMA4A	-2.895054189	0.001887834	0.004999937
SEMA6B	-3.132611389	1.29018E-05	4.96415E-05
EPHB3	-3.178922057	1.77951E-37	4.0885E-36
SEMA3G	-3.316366247	4.72862E-05	0.00016606
LGR4	-3.443711104	5.63327E-63	2.28841E-61
MYH10	-3.449550031	4.72243E-70	2.17594E-68
TGFB2	-4.020839745	1.27254E-85	7.32933E-84
RTN4R	-5.125131641	1.60576E-16	1.61904E-15
SCN1B	-5.358506536	3.58847E-15	3.31053E-14
SEMA7A	-6.034206332	6.337E-124	6.3818E-122
BOC	-6.060306568	1.44383E-06	6.29403E-06
EPHA10	-6.146258395	0.000989423	0.002773816
ARTN	-6.935353465	2.62503E-05	9.62186E-05
EFNA2	-7.756779325	5.30532E-06	2.14689E-05
ARHGEF25	-7.840781846	2.166E-55	7.62087E-54
EVL	-7.996042861	5.52202E-41	1.40711E-39
NEXN	-9.648745477	7.66994E-88	4.69066E-86

NOTCH3	-12.97966901	9.51103E-26	1.49492E-24
FLRT2	-14.37228895	4.1445E-143	5.1252E-141
DLX5	-15.45190324	1.17526E-40	2.95892E-39
SEMA4F	-18.57409978	4.79924E-06	1.95717E-05
ROBO1	-26.46726172	6.2967E-53	2.10533E-51
DRAXIN	-27.91460411	9.12128E-60	3.5346E-58
GLI2	-31.75493013	1.5518E-80	8.28515E-79
EPHA3	-35.37988868	1.38668E-13	1.16489E-12
SEMA3D	-56.17072155	1.74924E-92	1.15822E-90
RUNX3	-81.54873273	9.43815E-07	4.21932E-06
CXCL12	-119.2246393	0	0
SEMA5A	-232.961647	0	0
BMP7	-273.5193165	0.0008423	0.002398137

GO:0042060~wound healing

Gene	Fold change	P-value	FDR
JAG2	251.7688048	5.0403E-148	6.6225E-146
ITGA9	103.1265509	0.005039992	0.01221539
FGFR1	78.76091653	5.2318E-249	1.7253E-246
BMP4	57.19284363	1.03762E-33	2.1678E-32
POSTN	49.61333295	0	0
SERPINB2	46.82706879	2.48325E-54	8.42002E-53
HPSE	37.10742175	2.12587E-97	1.58199E-95
EVPL	28.55360967	1.13026E-92	7.54311E-91
MMP3	27.00172742	0	0
PPL	24.39892227	1.3593E-300	9.1441E-298
VANGL2	20.83508187	0.001012924	0.002832618
WNT7A	20.09038454	5.06508E-05	0.000177099
EPB41L4B	16.0493676	5.4118E-12	4.03617E-11
CXCL2	15.82358009	0.003452014	0.008679319
NRG1	11.78979842	8.3204E-99	6.30327E-97
TSKU	5.372775221	6.2774E-115	5.7376E-113
IL1A	5.341904665	1.10648E-06	4.89062E-06
PLAT	4.607912234	5.9136E-96	4.19641E-94
IL33	4.45691807	0.011369524	0.025212565
ALOX15	4.170857095	0.000700306	0.002021238
FMOD	4.0906514	9.21035E-70	4.2207E-68
F13A1	4.089270135	2.43326E-33	5.03352E-32
CX3CL1	3.63220813	3.03893E-09	1.79707E-08
TGFB1	3.239683624	2.88078E-23	4.0543E-22
TGFBR2	-2.564435378	2.7325E-37	6.24391E-36
AQP1	-2.568842052	5.05866E-38	1.18988E-36
TPM1	-2.707853812	6.32281E-46	1.81773E-44
CNN2	-2.79462985	4.44812E-48	1.33826E-46
PDGFRB	-3.278026165	9.87446E-62	3.93528E-60
EGR1	-3.425863714	1.2279E-21	1.59712E-20

FGF10	-3.88229903	0.00043203	0.001293553
DCN	-3.895382864	0.002426153	0.006288032
TGFB2	-4.020839745	1.27254E-85	7.32933E-84
CELSR1	-5.26712106	2.46881E-11	1.74823E-10
TGFB3	-5.467156262	4.5731E-107	3.9442E-105
CCL20	-5.915846112	1.9432E-11	1.38713E-10
PAK1	-7.323128553	7.6741E-148	1.0005E-145
LOX	-11.39158609	4.8078E-267	2.0733E-264
COL1A1	-12.34216059	1.3344E-221	3.1168E-219
NOTCH3	-12.97966901	9.51103E-26	1.49492E-24
COL3A1	-20.68811594	0	0
WNT5B	-43.93372766	3.12227E-46	9.06599E-45
ENG	-58.85916311	2.9757E-243	9.099E-241
PTK7	-103.6878249	1.7944E-167	2.874E-165
FGFR2	-141.2525937	1.42922E-86	8.43389E-85

**GO:0030154~cell differentiation**			
**Gene**	**Fold change**	**P-value**	**FDR**
FLI1	875.287799	3.10478E-05	0.000112221
BRDT	317.198833	1.73736E-49	5.35144E-48
JAG2	251.7688048	5.0403E-148	6.6225E-146
SIM2	177.5167137	0.0016187	0.004347381
HNF4A	169.9847837	0.001806207	0.004801135
FGF9	162.0402506	6.61875E-09	3.76188E-08
BMPR1B	115.636428	1.69496E-61	6.72308E-60
HNF4G	103.2946295	0.004898999	0.0119166
SFRP2	78.29953978	1.13749E-06	5.02242E-06
LOC108348103	52.90668076	1.09742E-11	8.00364E-11
HHEX	48.96842577	0.021050806	0.043407608
TP63	42.27644375	4.2588E-183	7.9582E-181
F11R	33.44203593	5.22541E-85	2.98915E-83
ZFP423	27.23579794	8.32553E-06	3.2822E-05
PTK2B	20.25052503	3.1435E-267	1.3912E-264
EOMES	19.71695834	1.29269E-20	1.60328E-19
PIWIL4	17.74390089	2.5658E-09	1.52557E-08
FCRLA	17.5067123	2.71973E-47	8.08136E-46
ARHGAP24	16.69502445	6.49875E-23	9.00296E-22
SPIB	12.32249274	0.007621409	0.017689327
ID4	12.04326173	2.0778E-19	2.42166E-18
NRG1	11.78979842	8.3204E-99	6.30327E-97
CAV2	11.76516338	3.8015E-149	5.1147E-147
SIM1	11.37634676	1.11941E-18	1.26012E-17
SYK	10.87731699	2.04958E-11	1.46058E-10
ERG	9.362259254	3.99655E-42	1.06016E-40
RXRG	6.961236607	0.000698211	0.00201692
MEF2B	5.076028792	5.65246E-05	0.000196411

PLET1	4.884970435	7.65082E-09	4.31504E-08
EYA2	4.569577061	0.003236151	0.008187993
SOX15	4.515670274	0.015413638	0.032993071
SYT1	4.090297112	3.20312E-67	1.43271E-65
NR1D1	4.057403961	7.1971E-41	1.82291E-39
FES	3.896034021	0.016454612	0.034922171
SOHLH1	3.846162916	5.15551E-09	2.9653E-08
NGEF	3.78427383	0.000409332	0.001232833
GGNBP1	3.75835613	6.81779E-05	0.000233813
STYK1	3.557444476	1.37144E-09	8.35684E-09
PDX1	3.463282261	7.60425E-08	3.87071E-07
TNK1	2.9573067	0.005791747	0.013859648
PPARG	2.870706856	1.38003E-16	1.39479E-15
THRA	2.799320676	1.12312E-44	3.12208E-43
GGN	2.794341479	0.000440623	0.001317872
FGF22	2.732770405	5.68168E-06	2.28709E-05
NME1	2.715117223	3.18829E-45	9.04228E-44
RNF125	2.712955125	9.94494E-05	0.000333308
FOXQ1	2.660063344	4.63679E-16	4.5076E-15
SQSTM1	2.636745813	9.29158E-43	2.49228E-41
FOXO1	2.615919245	8.83388E-11	5.99548E-10
CDX2	2.546874493	5.88109E-38	1.37755E-36
SMAD9	-2.572677507	1.44263E-38	3.45614E-37
YES1	-2.598523395	2.95504E-21	3.78506E-20
RHOB	-2.611616323	2.03595E-41	5.28404E-40
RIMS2	-2.625323585	5.63118E-26	8.95133E-25
MGP	-2.832447216	1.19228E-45	3.40437E-44
UHRF2	-2.878956898	1.5718E-41	4.10475E-40
ETV6	-2.911096652	1.60098E-21	2.07277E-20
SCX	-3.031280999	5.07219E-21	6.42834E-20
NR2F2	-3.090189483	3.2447E-27	5.41901E-26
GADD45G	-3.171168981	7.06465E-41	1.79206E-39
CAPRIN2	-3.326165981	3.44717E-26	5.52138E-25
EYA1	-3.404785259	7.28651E-33	1.48359E-31
NKX2-3	-3.491250955	4.5331E-14	3.93587E-13
FHL1	-3.866259968	3.15568E-80	1.6795E-78
FGF10	-3.88229903	0.00043203	0.001293553
SPATA18	-4.131770026	0.000224174	0.000705759
FYN	-4.347000726	0.000148877	0.000483267
WNT4	-4.540178988	1.55144E-87	9.41951E-86
FST	-4.604881988	5.46275E-39	1.32572E-37
VDR	-4.834836339	3.04071E-14	2.67459E-13
GATA2	-4.928086576	4.11352E-32	8.16779E-31
RXFP1	-5.212680023	7.83382E-37	1.77082E-35
PMP22	-5.256091097	3.2239E-110	2.8537E-108
RASGRP1	-6.416840017	6.18344E-07	2.83129E-06

FOXL1	-7.912920706	0.009033486	0.020549866
DLX3	-8.040271073	1.42587E-18	1.59549E-17
WIPF3	-9.071381571	6.95078E-13	5.54809E-12
FOXA1	-9.908935836	1.37813E-06	6.02948E-06
ROR2	-10.13588218	1.91403E-66	8.38286E-65
SRC	-10.28414552	1.1583E-182	2.1407E-180
SLCO4C1	-11.5954141	0.000182606	0.000584554
TMEM176B	-12.82465801	6.65181E-05	0.000228633
DLX5	-15.45190324	1.17526E-40	2.95892E-39
CAV3	-16.99205039	1.23753E-07	6.15215E-07
PRKD1	-17.77213424	7.7595E-253	2.9659E-250
TLL1	-18.43005553	3.98725E-12	3.00303E-11
DLX6	-22.24810374	8.60588E-09	4.82446E-08
FOXC2	-22.84796664	1.21312E-56	4.39704E-55
OSR1	-26.48335924	1.27844E-77	6.53521E-76
SARM1	-26.69411612	7.56937E-11	5.17565E-10
GLI2	-31.75493013	1.5518E-80	8.28515E-79
IGSF10	-48.85856299	3.51642E-18	3.86531E-17
FOXS1	-53.45128441	8.84191E-87	5.27317E-85
NR1H5	-56.50829014	0.018886847	0.039468066
NR5A1	-72.68374135	3.29443E-41	8.4589E-40
NKX2-2	-101.1535756	0.006244129	0.014828264
SOX11	-104.0172067	2.18469E-07	1.0549E-06
ONECUT1	-151.2059529	0.002833636	0.007253474
SEMA5A	-232.961647	0	0

GO:0001525~angiogenesis

Gene	Fold change	P-value	FDR
FGF9	162.0402506	6.61875E-09	3.76188E-08
KDR	128.8956701	0.003114572	0.007910128
FGFR1	78.76091653	5.2318E-249	1.7253E-246
BMP4	57.19284363	1.03762E-33	2.1678E-32
EPHB2	23.89713303	1.56057E-29	2.79507E-28
PTK2B	20.25052503	3.1435E-267	1.3912E-264
WNT7A	20.09038454	5.06508E-05	0.000177099
ARHGAP24	16.69502445	6.49875E-23	9.00296E-22
CSPG4	14.51742309	0	4.939E-307
ADAM15	14.49893529	6.9741E-220	1.585E-217
COL18A1	11.44209715	7.2108E-234	1.9249E-231
FLT1	11.31314812	5.24162E-14	4.52766E-13
SYK	10.87731699	2.04958E-11	1.46058E-10
OVOL2	9.483659635	0.020872672	0.043093125
NRCAM	8.774218487	0.023895826	0.048553824
GJA5	8.774126372	0.024184036	0.049091988
PTPRB	8.520225181	0.000941154	0.0026522
ADGRG1	6.740583999	0.012860255	0.028168297

EDNRA	6.151610242	5.21565E-75	2.59517E-73
COL24A1	4.436881988	0.003593192	0.008989929
VEGFD	4.356802592	8.03575E-11	5.4759E-10
FZD8	-2.522463038	4.64197E-11	3.22865E-10
HS6ST1	-2.574524178	1.7096E-39	4.17302E-38
RHOB	-2.611616323	2.03595E-41	5.28404E-40
C1GALT1	-2.698692062	9.39159E-46	2.69076E-44
PLXND1	-2.824415289	4.62405E-14	4.01069E-13
EPHB3	-3.178922057	1.77951E-37	4.0885E-36
PDGFRB	-3.278026165	9.87446E-62	3.93528E-60
MMP2	-3.847672007	0.000699408	0.002018992
FGF10	-3.88229903	0.00043203	0.001293553
DYSF	-3.936614859	1.0128E-83	5.71588E-82
WNT7B	-3.988822154	8.43E-05	0.000285781
MEIS1	-4.114110188	5.00261E-79	2.59673E-77
CALD1	-4.139582404	1.06639E-92	7.14522E-91
MFGE8	-4.21327375	1.2248E-95	8.58282E-94
HOXA7	-6.126049998	6.54146E-05	0.000225116
TEK	-6.152991416	3.14036E-17	3.28653E-16
ACVRL1	-6.191550627	0.021034084	0.043378446
RAPGEF3	-7.45865654	4.05866E-09	2.36516E-08
EPGN	-11.32572408	1.18325E-49	3.65808E-48
PRKD1	-17.77213424	7.7595E-253	2.9659E-250
SRPX2	-17.85188858	0	0
THY1	-19.20148399	4.5966E-147	5.9012E-145
CYP1B1	-20.28604261	0	0
RAMP3	-24.60500789	1.81325E-17	1.91673E-16
NDNF	-29.95026004	9.09382E-10	5.63107E-09
ACKR3	-31.06222833	1.38935E-66	6.13283E-65
ENG	-58.85916311	2.9757E-243	9.099E-241
JAM3	-70.01632755	2.18179E-24	3.1935E-23
MEOX2	-77.87996946	2.83444E-31	5.4667E-30
ANGPT4	-87.85014729	2.0657E-224	5.1851E-222
VASH1	-117.6884051	2.29265E-12	1.76871E-11
FGFR2	-141.2525937	1.42922E-86	8.43389E-85
VEGFC	-176.5874263	2.29173E-30	4.27772E-29
GREM1	-211.5665067	6.2574E-299	4.0476E-296

GO:0008360~regulation of cell shape

Gene	Fold change	P-value	FDR
KDR	128.8956701	0.003114572	0.007910128
DMTN	77.71205026	0.008281727	0.019006834
PLXNA4	67.4716477	0.010910958	0.024287198
KIT	40.2797606	1.03714E-30	1.96425E-29
SHROOM3	37.62642251	1.25159E-34	2.66783E-33
F11R	33.44203593	5.22541E-85	2.98915E-83

PTK2B	20.25052503	3.1435E-267	1.3912E-264
FMNL1	13.03182758	0.00667983	0.015742907
SEMA3E	8.549562274	5.53185E-44	1.51769E-42
WASF3	5.438692848	1.5489E-13	1.29663E-12
SEMA4D	5.028204743	0.022900879	0.046775199
FES	3.896034021	0.016454612	0.034922171
VIL1	3.687085971	0.00826711	0.018978469
SPTA1	3.220548196	5.11926E-09	2.94646E-08
S100A13	3.007576927	1.47488E-11	1.06412E-10
BAIAP2	2.926210247	8.43812E-51	2.66752E-49
SH3KBP1	2.640559161	1.33964E-06	5.8718E-06
FAM171A1	2.500585018	2.38833E-14	2.11627E-13
STRIP2	-2.537116808	0.000123538	0.000407466
RHOB	-2.611616323	2.03595E-41	5.28404E-40
TPM1	-2.707853812	6.32281E-46	1.81773E-44
PLXND1	-2.824415289	4.62405E-14	4.01069E-13
SEMA4A	-2.895054189	0.001887834	0.004999937
DAPK3	-3.057563157	1.07857E-38	2.59504E-37
RND3	-3.064209445	9.90664E-57	3.59849E-55
RHOBTB3	-3.415226711	1.3503E-62	5.43285E-61
MYH10	-3.449550031	4.72243E-70	2.17594E-68
CCL7	-4.000530375	5.82763E-09	3.33773E-08
FYN	-4.347000726	0.000148877	0.000483267
EPS8	-4.44832523	6.65004E-94	4.56492E-92
EPB41L3	-5.27625697	7.38799E-08	3.76861E-07
FMNL2	-6.137872728	3.84777E-81	2.08748E-79
PALM	-6.43338209	1.07826E-55	3.83386E-54
LPAR1	-8.872464383	9.0763E-166	1.4266E-163
FGD4	-21.41588284	3.02021E-07	1.43283E-06
PALM2	-21.45553	5.88244E-29	1.03593E-27
PLXNB1	-28.79877068	5.83929E-58	2.17268E-56
PLXNC1	-34.99878246	3.30899E-32	6.59366E-31
PDPN	-74.98670597	4.44924E-25	6.75336E-24
RHOJ	-158.9434365	1.47903E-90	9.38654E-89
GAS7	-183.001569	3.77343E-09	2.20812E-08

GO:0043410~positive regulation of MAPK cascade

Gene	Fold change	P-value	FDR
FGF9	162.0402506	6.61875E-09	3.76188E-08
KDR	128.8956701	0.003114572	0.007910128
GDF15	126.0854887	1.66678E-70	7.72227E-69
CDH2	86.62228472	1.36E-156	2.0064E-154
LAPTM5	85.87823464	0.007340115	0.017116758
FGFR1	78.76091653	5.2318E-249	1.7253E-246
RT1-DB1	71.82150314	4.9466E-20	5.94653E-19
LILRA5	69.35328625	0.011186471	0.024829625

ALK	59.98077183	3.122E-12	2.37154E-11
BMP4	57.19284363	1.03762E-33	2.1678E-32
KIT	40.2797606	1.03714E-30	1.96425E-29
GPER1	36.62157844	2.06866E-17	2.17987E-16
TMEM106A	28.26083303	1.1138E-16	1.13047E-15
PLCE1	25.43471734	7.73599E-55	2.67703E-53
EPHA8	23.67529332	0.000628559	0.001827645
CD74	15.75778396	1.77463E-08	9.64012E-08
CSPG4	14.51742309	0	4.939E-307
C1QTNF1	13.92330565	1.9158E-116	1.79E-114
CAV2	11.76516338	3.8015E-149	5.1147E-147
FLT1	11.31314812	5.24162E-14	4.52766E-13
SYK	10.87731699	2.04958E-11	1.46058E-10
IGFBP6	8.001690108	1.96589E-54	6.67927E-53
GRM4	5.690708972	1.34412E-07	6.65059E-07
NTRK2	5.439056267	0.001220677	0.003354468
ADORA1	4.947532725	0.000229285	0.000720635
KSR1	4.844633869	8.84241E-23	1.21496E-21
PRKCD	4.441938876	2.14646E-64	9.07015E-63
APP	3.788256992	1.12742E-79	5.9253E-78
CX3CL1	3.63220813	3.03893E-09	1.79707E-08
AGT	2.90888258	0.004462768	0.010959497
GRM1	2.677413924	0.000129899	0.000426644
LIF	2.652466969	9.95695E-13	7.85441E-12
IGFBP4	2.545132511	6.64406E-36	1.45684E-34
ARRB1	-3.104365246	1.97882E-32	3.97609E-31
GADD45G	-3.171168981	7.06465E-41	1.79206E-39
FGF10	-3.88229903	0.00043203	0.001293553
IL11	-4.307802165	0.002497794	0.006464744
TGFB3	-5.467156262	4.5731E-107	3.9442E-105
MAP2K2	-5.797886055	1.8908E-135	2.106E-133
TEK	-6.152991416	3.14036E-17	3.28653E-16
C1QTNF2	-7.760856527	4.46654E-19	5.12053E-18
LPAR1	-8.872464383	9.0763E-166	1.4266E-163
ROR2	-10.13588218	1.91403E-66	8.38286E-65
PTPRJ	-57.46832266	2.88752E-55	1.00961E-53
DOK5	-136.5955392	1.1519E-82	6.3726E-81
FGFR2	-141.2525937	1.42922E-86	8.43389E-85
DNAJC27	-175.965629	4.99095E-24	7.22356E-23
FLT4	-444.4208615	3.54407E-96	2.53635E-94
ADRB3	-885.2112841	4.55104E-05	0.000160258

GO:0001666~response to hypoxia

Gene	Fold change	P-value	FDR
JAG2	251.7688048	5.0403E-148	6.6225E-146
KDR	128.8956701	0.003114572	0.007910128

CYP1A1	73.88901434	1.09158E-16	1.10859E-15
POSTN	49.61333295	0	0
ABCB1A	45.39057127	0	0
PPARGC1A	41.29456904	3.75061E-62	1.50185E-60
AGTR1B	40.6737387	2.22614E-21	2.86452E-20
TLR4	38.24672419	5.0351E-142	6.1362E-140
TH	28.40692277	1.85779E-13	1.54675E-12
MMP3	27.00172742	0	0
PTK2B	20.25052503	3.1435E-267	1.3912E-264
ALDOC	15.64560703	1.46165E-17	1.55386E-16
ADIPOQ	12.5928633	3.23261E-09	1.90624E-08
CD24	12.56412796	5.6624E-258	2.3227E-255
CASP1	11.56752975	6.3353E-51	2.01032E-49
FLT1	11.31314812	5.24162E-14	4.52766E-13
VCAM1	7.296516753	5.50554E-19	6.27744E-18
RYR1	7.045348034	9.51918E-22	1.24878E-20
EDNRA	6.151610242	5.21565E-75	2.59517E-73
PRKCQ	5.413507333	2.30551E-06	9.80878E-06
IL1A	5.341904665	1.10648E-06	4.89062E-06
ACSL6	5.038877682	0.017544622	0.036966356
ADORA1	4.947532725	0.000229285	0.000720635
PLAT	4.607912234	5.9136E-96	4.19641E-94
PRKCD	4.441938876	2.14646E-64	9.07015E-63
SERPINA1	4.40506247	1.38679E-06	6.06109E-06
VEGFD	4.356802592	8.03575E-11	5.4759E-10
MYB	4.243845569	6.14742E-10	3.88674E-09
CX3CL1	3.63220813	3.03893E-09	1.79707E-08
DPP4	3.548465042	0.000971472	0.002729404
CRYAB	3.320127859	8.86121E-56	3.16407E-54
CHRNB2	3.252214921	1.26023E-05	4.85556E-05
TGFB1	3.239683624	2.88078E-23	4.0543E-22
DDIT4	3.157315459	8.1629E-09	4.58529E-08
MMP9	2.957771215	6.92724E-08	3.54111E-07
TGFBR2	-2.564435378	2.7325E-37	6.24391E-36
SMAD9	-2.572677507	1.44263E-38	3.45614E-37
SRF	-2.814971338	1.89711E-47	5.65702E-46
FOSL2	-2.922806842	1.05463E-16	1.07301E-15
EGR1	-3.425863714	1.2279E-21	1.59712E-20
PDE5A	-3.652960806	3.06475E-24	4.45873E-23
ITPR1	-3.751463865	6.41147E-78	3.28744E-76
MMP2	-3.847672007	0.000699408	0.002018992
TGFB2	-4.020839745	1.27254E-85	7.32933E-84
ARNT2	-4.506734659	8.88426E-96	6.2517E-94
MMP13	-5.1390066	7.5531E-87	4.52057E-85
TGFB3	-5.467156262	4.5731E-107	3.9442E-105
VLDLR	-5.985985359	4.88269E-35	1.05009E-33

TEK	-6.152991416	3.14036E-17	3.28653E-16
ACVRL1	-6.191550627	0.021034084	0.043378446
PAK1	-7.323128553	7.6741E-148	1.0005E-145
FAS	-11.03817713	8.77406E-13	6.97363E-12
CAV3	-16.99205039	1.23753E-07	6.15215E-07
PTGIS	-19.03097991	6.18366E-19	7.02681E-18
TLR2	-22.13062658	2.22101E-05	8.23296E-05
EDN1	-25.85498871	6.83621E-27	1.12277E-25
ADA	-32.17015327	3.04304E-34	6.42938E-33
ENG	-58.85916311	2.9757E-243	9.099E-241
NOX4	-79.95595689	7.62377E-23	1.05182E-21
SLC8A1	-99.94256699	4.6304E-227	1.2168E-224
CXCL12	-119.2246393	0	0
VEGFC	-176.5874263	2.29173E-30	4.27772E-29

GO:0007229~integrin-mediated signaling pathway

Gene	Fold change	P-value	FDR
ITGA9	103.1265509	0.005039992	0.01221539
ADAM32	70.50970855	5.15562E-42	1.36332E-40
VAV3	65.24318394	3.43416E-65	1.47713E-63
PTK2B	20.25052503	3.1435E-267	1.3912E-264
ITGAX	18.73648598	3.2597E-238	9.2919E-236
ITGBL1	16.06912672	1.07629E-72	5.12777E-71
ITGA10	15.70544774	4.55078E-81	2.46094E-79
ADAM15	14.49893529	6.9741E-220	1.585E-217
SYK	10.87731699	2.04958E-11	1.46058E-10
FERMT3	10.45141497	0.000732884	0.002109109
ITGAD	7.400109986	0.003708433	0.009250731
ITGB7	6.814517514	0.000454856	0.001357064
TXK	4.619967255	1.12694E-07	5.62569E-07
ITGA8	3.611198444	4.49354E-42	1.19012E-40
ITGAL	3.447752354	0.005328313	0.01286229
ITGA3	3.357115344	2.82153E-68	1.27219E-66
LAMA5	3.071812695	7.87142E-58	2.92233E-56
FUT8	-3.391344208	2.32853E-64	9.74161E-63
ADAMTS1	-3.642879824	9.25219E-72	4.38319E-70
SEMA7A	-6.034206332	6.337E-124	6.3818E-122
CDH17	-8.453502771	2.25306E-38	5.36714E-37
SRC	-10.28414552	1.1583E-182	2.1407E-180
ITGB3	-12.59597777	0.000144452	0.000470175
THY1	-19.20148399	4.5966E-147	5.9012E-145
COL3A1	-20.68811594	0	0
ITGA11	-30.87084838	0	0
ITGA6	-81.27399127	0	0
ITGB8	-224.6733022	7.34897E-10	4.60488E-09
ADAMTS2	-412.0398473	5.81623E-27	9.58993E-26

GO:0042127~regulation of cell proliferation			
**Gene**	**Fold change**	**P-value**	**FDR**
JAG2	251.7688048	5.0403E-148	6.6225E-146
DUSP22	133.7466674	4.6041E-13	3.71554E-12
FGFR1	78.76091653	5.2318E-249	1.7253E-246
SFRP2	78.29953978	1.13749E-06	5.02242E-06
ALK	59.98077183	3.122E-12	2.37154E-11
JUP	55.18666521	0	0
HHEX	48.96842577	0.021050806	0.043407608
WNT7A	20.09038454	5.06508E-05	0.000177099
SLC7A11	13.92588088	2.3213E-121	2.283E-119
PRDM1	12.29330279	2.73409E-11	1.93282E-10
ERBB3	9.288144992	1.16007E-21	1.51475E-20
STAT5A	8.407293857	1.7681E-102	1.4296E-100
PRG4	7.018482465	1.72768E-05	6.51172E-05
CXCL11	6.943817155	1.37224E-05	5.25703E-05
TFAP2A	5.839976093	3.97931E-48	1.20151E-46
KSR1	4.844633869	8.84241E-23	1.21496E-21
FA2H	4.836003528	8.9951E-06	3.52469E-05
PITX2	4.598605843	1.92571E-26	3.11409E-25
FES	3.896034021	0.016454612	0.034922171
IL4R	3.760738647	2.30219E-32	4.62031E-31
PDX1	3.463282261	7.60425E-08	3.87071E-07
OSGIN1	3.311585766	2.69519E-16	2.67262E-15
TGFB1	3.239683624	2.88078E-23	4.0543E-22
LAMA5	3.071812695	7.87142E-58	2.92233E-56
NDRG1	3.032975478	2.4772E-52	8.18497E-51
TCF7	2.744086282	0.001506751	0.004073387
ESRRA	2.627159076	1.47929E-32	2.97949E-31
FOXO1	2.615919245	8.83388E-11	5.99548E-10
CXCL10	2.529647431	3.34949E-26	5.37517E-25
EID2	2.50827327	0.020158418	0.041730006
TGFBR2	-2.564435378	2.7325E-37	6.24391E-36
CNN2	-2.79462985	4.44812E-48	1.33826E-46
NR3C2	-3.372452395	1.5062E-20	1.8585E-19
NKX2-3	-3.491250955	4.5331E-14	3.93587E-13
TRNP1	-3.958141624	0.006402339	0.015152622
TGFB2	-4.020839745	1.27254E-85	7.32933E-84
DUSP15	-4.1466895	0.002120611	0.00555867
PTGS1	-4.324076409	9.9396E-97	7.26801E-95
TGFB3	-5.467156262	4.5731E-107	3.9442E-105
GUCY2C	-5.562738123	0.014014652	0.030408776
HOXD13	-6.205396407	7.3009E-114	6.6015E-112
GRPR	-7.70250748	2.5947E-06	1.09615E-05
DBH	-8.498164709	1.19064E-05	4.60432E-05

FAS	-11.03817713	8.77406E-13	6.97363E-12
CADM4	-40.03424109	2.1995E-25	3.39681E-24
FGFR2	-141.2525937	1.42922E-86	8.43389E-85

GO:0051897~positive regulation of protein kinase B signaling

Gene	Fold change	P-value	FDR
PIK3CG	343.8651175	0.000355495	0.00108036
GDF15	126.0854887	1.66678E-70	7.72227E-69
MST1R	84.06641487	1.25205E-23	1.78752E-22
FGFR1	78.76091653	5.2318E-249	1.7253E-246
HCLS1	52.11805983	0.017378135	0.036656902
HPSE	37.10742175	2.12587E-97	1.58199E-95
GPER1	36.62157844	2.06866E-17	2.17987E-16
F3	13.92413522	5.94733E-77	2.99468E-75
C1QTNF1	13.92330565	1.9158E-116	1.79E-114
NRG1	11.78979842	8.3204E-99	6.30327E-97
PIK3R5	9.483946143	0.018223833	0.038225046
MYOC	9.483946143	0.018176762	0.03813107
FAM110C	7.268897225	6.60125E-15	6.03697E-14
TNFAIP8L3	4.14312651	5.64513E-30	1.0342E-28
CX3CL1	3.63220813	3.03893E-09	1.79707E-08
TGFB1	3.239683624	2.88078E-23	4.0543E-22
GPX1	2.95876815	9.44265E-52	3.07169E-50
GATA3	2.585040044	1.1389E-15	1.08337E-14
LRP2	-2.886983942	0.010902343	0.024279079
THBS1	-3.298804306	1.70196E-66	7.47353E-65
TEK	-6.152991416	3.14036E-17	3.28653E-16
CD28	-6.42317603	2.21002E-49	6.79489E-48
AKR1C3	-10.0542414	0.00267267	0.006870829
SRC	-10.28414552	1.1583E-182	2.1407E-180
PIK3CD	-10.47677089	5.389E-182	9.8513E-180
RAMP3	-24.60500789	1.81325E-17	1.91673E-16
GAS6	-41.90614353	3.02503E-86	1.77265E-84
PTPRJ	-57.46832266	2.88752E-55	1.00961E-53
ENG	-58.85916311	2.9757E-243	9.099E-241
NOX4	-79.95595689	7.62377E-23	1.05182E-21
CXCL12	-119.2246393	0	0
SEMA5A	-232.961647	0	0

GO:0006954~inflammatory response

Gene	Fold change	P-value	FDR
KRT16	192.8990134	0.001320122	0.003602436
BMPR1B	115.636428	1.69496E-61	6.72308E-60
S100A7A	54.67344164	0.016589371	0.035165118
TRPV1	44.44362359	0.023165734	0.047207236
TLR5	40.74160583	1.29807E-29	2.34489E-28

AGTR1B	40.6737387	2.22614E-21	2.86452E-20
KIT	40.2797606	1.03714E-30	1.96425E-29
TLR4	38.24672419	5.0351E-142	6.1362E-140
HPSE	37.10742175	2.12587E-97	1.58199E-95
GPER1	36.62157844	2.06866E-17	2.17987E-16
NGF	19.14775912	5.13049E-53	1.71882E-51
LGALS9	16.24377043	3.97707E-14	3.47462E-13
CXCL2	15.82358009	0.003452014	0.008679319
CSPG4	14.51742309	0	4.939E-307
TLR10	12.54178094	0.010644505	0.023764672
TLR13	11.81233506	1.0781E-17	1.15413E-16
OLR1	9.489089175	9.13168E-08	4.60361E-07
CXCL11	6.943817155	1.37224E-05	5.25703E-05
NTRK2	5.439056267	0.001220677	0.003354468
PRKCQ	5.413507333	2.30551E-06	9.80878E-06
IL1A	5.341904665	1.10648E-06	4.89062E-06
SERPINA1	4.40506247	1.38679E-06	6.06109E-06
TNFRSF1B	4.211006607	2.22311E-51	7.13517E-50
IL4R	3.760738647	2.30219E-32	4.62031E-31
CX3CL1	3.63220813	3.03893E-09	1.79707E-08
SERPINB1A	3.391669535	2.71883E-20	3.31583E-19
TGFB1	3.239683624	2.88078E-23	4.0543E-22
F2RL1	3.049234632	2.41321E-51	7.71585E-50
GGT5	2.846467349	5.25471E-07	2.42518E-06
RELB	2.723064288	7.5782E-12	5.58992E-11
CYP4F5	2.548875246	2.68167E-05	9.8108E-05
IGFBP4	2.545132511	6.64406E-36	1.45684E-34
PYCARD	2.53860212	5.67539E-06	2.2851E-05
CXCL10	2.529647431	3.34949E-26	5.37517E-25
CXCL3	-2.93260462	6.70269E-07	3.05242E-06
C3	-3.261178346	4.02526E-09	2.34814E-08
THBS1	-3.298804306	1.70196E-66	7.47353E-65
HCN1	-3.457888924	8.49622E-07	3.82159E-06
CYP4F6	-3.545682162	4.42239E-06	1.81582E-05
IL17RE	-3.770491642	1.18031E-28	2.06775E-27
TBXA2R	-3.798302065	7.43693E-09	4.20418E-08
CCL7	-4.000530375	5.82763E-09	3.33773E-08
EPHX2	-4.314760305	0.000745972	0.002141646
PTGS1	-4.324076409	9.9396E-97	7.26801E-95
PRKCZ	-4.692663802	1.69505E-09	1.0225E-08
S1PR3	-5.761664073	6.54602E-10	4.12017E-09
CCL20	-5.915846112	1.9432E-11	1.38713E-10
BDKRB1	-6.139780517	3.24337E-08	1.71964E-07
NAIP6	-7.093784937	4.11944E-12	3.09565E-11
CCR1	-7.121586024	2.80824E-10	1.82351E-09
KNG1	-11.32388304	5.96057E-24	8.59733E-23

DPEP1	-11.62308056	0.000160997	0.0005206
PTAFR	-15.66977508	2.57312E-15	2.40683E-14
PRKD1	-17.77213424	7.7595E-253	2.9659E-250
TLR2	-22.13062658	2.22101E-05	8.23296E-05
IL1RN	-23.46797782	4.20702E-88	2.58225E-86
ABCC2	-25.18127774	1.7933E-12	1.39306E-11
AFAP1L2	-36.88591286	3.13209E-98	2.35158E-96
HCN2	-62.94872568	5.93048E-06	2.38382E-05
NLRP3	-72.47445778	4.7618E-118	4.474E-116
PDPN	-74.98670597	4.44924E-25	6.75336E-24
PTGFR	-109.4562853	3.1889E-41	8.20045E-40

GO:0071560~cellular response to transforming growth factor beta stimulus

Gene	Fold change	P-value	FDR
POSTN	49.61333295	0	0
PPARGC1A	41.29456904	3.75061E-62	1.50185E-60
SOX5	24.62947922	7.90649E-18	8.56223E-17
WNT7A	20.09038454	5.06508E-05	0.000177099
OVOL2	9.483659635	0.020872672	0.043093125
SOX6	5.990198731	8.20684E-10	5.11006E-09
L1CAM	4.189172955	1.93827E-05	7.25684E-05
TGFB1	3.239683624	2.88078E-23	4.0543E-22
ENPP1	-2.512184043	4.20598E-39	1.0222E-37
YES1	-2.598523395	2.95504E-21	3.78506E-20
SCX	-3.031280999	5.07219E-21	6.42834E-20
FYN	-4.347000726	0.000148877	0.000483267
WNT4	-4.540178988	1.55144E-87	9.41951E-86
ACVRL1	-6.191550627	0.021034084	0.043378446
RAPGEF3	-7.45865654	4.05866E-09	2.36516E-08
DBN1	-9.907819871	4.438E-92	2.89857E-90
AKR1C3	-10.0542414	0.00267267	0.006870829
SRC	-10.28414552	1.1583E-182	2.1407E-180
COL4A2	-11.26542586	6.1755E-226	1.5978E-223
COL1A1	-12.34216059	1.3344E-221	3.1168E-219
EDN1	-25.85498871	6.83621E-27	1.12277E-25
CILP	-31.24830909	1.653E-101	1.3175E-99
FBN1	-57.1134887	3.82546E-29	6.77941E-28
ANKRD1	-77.76368597	0	0
NOX4	-79.95595689	7.62377E-23	1.05182E-21
FGFR2	-141.2525937	1.42922E-86	8.43389E-85

GO:0010862~positive regulation of pathway-restricted SMAD protein phosphorylation

Gene	Fold change	P-value	FDR
GDF15	126.0854887	1.66678E-70	7.72227E-69
BMP4	57.19284363	1.03762E-33	2.1678E-32
BMP3	50.51377756	4.13881E-16	4.04689E-15

LGALS9	16.24377043	3.97707E-14	3.47462E-13
HFE	6.609931738	6.73284E-31	1.27802E-29
RBPMS	4.79212154	7.79476E-13	6.20702E-12
GDF6	3.752973178	0.001292061	0.003530443
TGFB1	3.239683624	2.88078E-23	4.0543E-22
PPARG	2.870706856	1.38003E-16	1.39479E-15
TGFBR2	-2.564435378	2.7325E-37	6.24391E-36
INHBE	-3.481725991	0.000149209	0.000484254
TGFB2	-4.020839745	1.27254E-85	7.32933E-84
TGFB3	-5.467156262	4.5731E-107	3.9442E-105
ACVRL1	-6.191550627	0.021034084	0.043378446
ENG	-58.85916311	2.9757E-243	9.099E-241
BMP5	-59.18689709	1.91007E-56	6.87872E-55
BMP7	-273.5193165	0.0008423	0.002398137

GO:0032757~positive regulation of interleukin-8 production

Gene	Fold change	P-value	FDR
PLA2G1B	54.67281472	0.01655655	0.035104395
TLR5	40.74160583	1.29807E-29	2.34489E-28
TLR4	38.24672419	5.0351E-142	6.1362E-140
LGALS9	16.24377043	3.97707E-14	3.47462E-13
CD74	15.75778396	1.77463E-08	9.64012E-08
F3	13.92413522	5.94733E-77	2.99468E-75
FCNA	13.28321188	1.42723E-07	7.03493E-07
ADIPOQ	12.5928633	3.23261E-09	1.90624E-08
CD244	12.42387568	2.49839E-13	2.0587E-12
SYK	10.87731699	2.04958E-11	1.46058E-10
DDX58	3.541654399	1.67437E-12	1.30308E-11
F2RL1	3.049234632	2.41321E-51	7.71585E-50
ZFP580	2.98997103	0.000274631	0.000852325
PYCARD	2.53860212	5.67539E-06	2.2851E-05
HSPA1B	-3.235574911	9.91704E-05	0.000332439
HSPA1A	-3.900972453	3.87836E-05	0.000138103
LBP	-14.08752436	2.08175E-23	2.94705E-22
TLR2	-22.13062658	2.22101E-05	8.23296E-05
AFAP1L2	-36.88591286	3.13209E-98	2.35158E-96

GO:0071347~cellular response to interleukin-1

Gene	Fold change	P-value	FDR
MMP3	27.00172742	0	0
CXCL2	15.82358009	0.003452014	0.008679319
AKAP12	7.688974317	2.75873E-81	1.5015E-79
MMP1	7.685048403	0.002450386	0.006345949
MYLK3	5.900468845	0.000883744	0.002504265
ADAMTS7	5.373667045	9.3918E-111	8.4017E-109
PSMB9	5.107872981	6.05233E-35	1.29832E-33

SIRPA	4.490264887	1.80857E-87	1.0902E-85
GBP3	4.170175415	0.00467656	0.011433403
NR1D1	4.057403961	7.1971E-41	1.82291E-39
CX3CL1	3.63220813	3.03893E-09	1.79707E-08
MMP9	2.957771215	6.92724E-08	3.54111E-07
PYCARD	2.53860212	5.67539E-06	2.2851E-05
LCN2	-2.559379565	0.000376477	0.001139594
KLF2	-3.19685483	4.86162E-10	3.09707E-09
CACTIN	-3.364997251	3.62205E-65	1.55397E-63
MMP2	-3.847672007	0.000699408	0.002018992
CCL7	-4.000530375	5.82763E-09	3.33773E-08
CCL20	-5.915846112	1.9432E-11	1.38713E-10
VLDLR	-5.985985359	4.88269E-35	1.05009E-33
ADAMTS12	-7.794156838	1.4102E-103	1.1569E-101
FAS	-11.03817713	8.77406E-13	6.97363E-12
PTGIS	-19.03097991	6.18366E-19	7.02681E-18
ABCC2	-25.18127774	1.7933E-12	1.39306E-11
EDN1	-25.85498871	6.83621E-27	1.12277E-25
FGG	-28.11222149	8.01409E-33	1.62387E-31
ANKRD1	-77.76368597	0	0

GO:0014068~positive regulation of phosphatidylinositol 3-kinase signaling

Gene	Fold change	P-value	FDR
RELN	448.6971861	0.000177033	0.000568086
KDR	128.8956701	0.003114572	0.007910128
HCLS1	52.11805983	0.017378135	0.036656902
GPER1	36.62157844	2.06866E-17	2.17987E-16
NRG1	11.78979842	8.3204E-99	6.30327E-97
ERBB4	11.63291009	1.75241E-31	3.40716E-30
FLT1	11.31314812	5.24162E-14	4.52766E-13
MYOC	9.483946143	0.018176762	0.03813107
NTRK2	5.439056267	0.001220677	0.003354468
SEMA4D	5.028204743	0.022900879	0.046775199
F2RL1	3.049234632	2.41321E-51	7.71585E-50
AGT	2.90888258	0.004462768	0.010959497
PDGFRB	-3.278026165	9.87446E-62	3.93528E-60
DCN	-3.895382864	0.002426153	0.006288032
TGFB2	-4.020839745	1.27254E-85	7.32933E-84
FYN	-4.347000726	0.000148877	0.000483267
TEK	-6.152991416	3.14036E-17	3.28653E-16
CD28	-6.42317603	2.21002E-49	6.79489E-48
ROR2	-10.13588218	1.91403E-66	8.38286E-65
PLXNB1	-28.79877068	5.83929E-58	2.17268E-56
PRR5L	-107.1772962	1.14343E-19	1.35139E-18

GO:0009615~response to virus

Gene	Fold change	P-value	FDR
CHRM2	87.94575893	0.006838213	0.016071137
MST1R	84.06641487	1.25205E-23	1.78752E-22
CYP1A1	73.88901434	1.09158E-16	1.10859E-15
IFI27L2B	11.84061258	2.01517E-24	2.95734E-23
OASL2	8.527704345	1.92004E-44	5.31981E-43
IFIT2	7.873298488	3.01626E-18	3.32639E-17
MX1	6.419599595	4.23072E-05	0.000149857
OAS1A	6.128225321	2.78951E-05	0.000101798
OASL	4.912241476	4.55706E-13	3.67934E-12
DHX58	4.789272588	3.57279E-08	1.88302E-07
RSAD2	4.429689508	0.001518081	0.00410072
IFIT3	3.667454137	0.000293472	0.000904787
DDX58	3.541654399	1.67437E-12	1.30308E-11
GATA3	2.585040044	1.1389E-15	1.08337E-14
CXCL10	2.529647431	3.34949E-26	5.37517E-25
LCN2	-2.559379565	0.000376477	0.001139594
NMI	-3.064066784	1.24131E-06	5.46071E-06
SRC	-10.28414552	1.1583E-182	2.1407E-180
IFITM1	-11.56019295	2.70311E-25	4.1479E-24
DUOX2	-31.12533221	1.18685E-25	1.85333E-24
ACTA2	-64.87987773	0	0
IFIH1	-126.4797195	6.42973E-30	1.17666E-28
ITGB8	-224.6733022	7.34897E-10	4.60488E-09

GO:0007219~Notch signaling pathway			
**Gene**	**Fold change**	**P-value**	**FDR**
JAG2	251.7688048	5.0403E-148	6.6225E-146
GRIP2	67.47253815	0.011134789	0.024721437
HHEX	48.96842577	0.021050806	0.043407608
TP63	42.27644375	4.2588E-183	7.9582E-181
ZFP423	27.23579794	8.32553E-06	3.2822E-05
HES3	13.74037632	0.005440967	0.013100384
DLL1	9.115179387	1.8478E-43	5.01231E-42
MESP2	5.487029088	6.25331E-06	2.50699E-05
APP	3.788256992	1.12742E-79	5.9253E-78
TGFB1	3.239683624	2.88078E-23	4.0543E-22
SNAI2	-2.531268578	6.72592E-26	1.06413E-24
TGFBR2	-2.564435378	2.7325E-37	6.24391E-36
SIX1	-2.735833083	7.64704E-41	1.93395E-39
DTX4	-3.086377057	2.07095E-08	1.11883E-07
SORBS2	-4.295336028	3.26492E-72	1.55111E-70
S1PR3	-5.761664073	6.54602E-10	4.12017E-09
RCAN2	-9.884328732	5.44844E-73	2.60318E-71
FOXA1	-9.908935836	1.37813E-06	6.02948E-06
NOTCH3	-12.97966901	9.51103E-26	1.49492E-24

TBX2	-14.24885327	5.29701E-52	1.73317E-50
PLN	-17.89090567	1.19968E-19	1.41687E-18
FOXC2	-22.84796664	1.21312E-56	4.39704E-55
SUSD5	-33.46564099	2.19788E-95	1.53377E-93
CDH6	-48.33112962	0.023115609	0.047122219
ANGPT4	-87.85014729	2.0657E-224	5.1851E-222
ONECUT1	-151.2059529	0.002833636	0.007253474

Table S3. Differentially expressed genes in MM cells treated with desamethasone

Cutoffs for differentially expressed genes: Fold change>2.5, P-value<0.05, FDR<0.05

Name	Log_2 fold change	Fold change	P-value	FDR p-value	Bonferroni	ENSEMBL	Biotype
Raet1l	-1.3402	-2.5319	0.00465	0.02114	1	ENSRNOG	protein_coding
Nup43	-1.4641	-2.7589	7.7E-06	0.00012	0.14805	ENSRNOG	protein_coding
Zc3h12d	2.24063	4.72604	0.00158	0.00901	1	ENSRNOG	protein_coding
Samd5	-2.3006	-4.9265	6.7E-08	1.7E-06	0.00128	ENSRNOG	protein_coding
Grm1	1.74392	3.34943	0.00012	0.00118	1	ENSRNOG	protein_coding
Adgrg6	-1.4219	-2.6793	2.9E-05	0.00036	0.55598	ENSRNOG	protein_coding
AABR070	1.84347	3.58872	0.00458	0.02096	1	ENSRNOG	lincRNA
Ccdc28a	1.65201	3.14271	1.4E-08	4.1E-07	0.00027	ENSRNOG	protein_coding
LOC1025	2.92989	7.62052	0.00345	0.01681	1	ENSRNOG	lincRNA
RGD1559	-1.7192	-3.2926	0.00047	0.00348	1	ENSRNOG	protein_coding
Slc2a12	2.53605	5.8	1.2E-10	4.8E-09	2.3E-06	ENSRNOG	protein_coding
AABR070	1.85457	3.61645	0.00223	0.01195	1	ENSRNOG	lincRNA
Soga3	3.04159	8.23401	3.8E-06	6.3E-05	0.07265	ENSRNOG	protein_coding
NEWGEN	1.8607	3.63184	0.00119	0.00712	1	ENSRNOG	protein_coding
Trip13	-1.9358	-3.8259	2.5E-05	0.00031	0.47454	ENSRNOG	protein_coding
Irx4	2.79811	6.95529	0.00044	0.00327	1	ENSRNOG	protein_coding
Zfp874b	1.52174	2.87138	0.00068	0.0046	1	ENSRNOG	protein_coding
Mthfd1l	-1.7961	-3.4728	0.00023	0.00196	1	ENSRNOG	protein_coding
Myct1	-1.3371	-2.5265	0.00013	0.00122	1	ENSRNOG	protein_coding
Vip	-2.5762	-5.9637	0.00493	0.02217	1	ENSRNOG	protein_coding
Fbxo5	-1.8899	-3.706	2.9E-06	5E-05	0.05604	ENSRNOG	protein_coding
AABR070	3.91479	15.0824	0.00373	0.01785	1	ENSRNOG	lincRNA
Tagap	4.41719	21.3651	0.00087	0.00553	1	ENSRNOG	protein_coding
Fndc1	2.37947	5.20344	3.3E-20	3.9E-18	6.3E-16	ENSRNOG	protein_coding
Acat2	1.71784	3.28943	0.01011	0.03895	1	ENSRNOG	protein_coding
AABR070	-1.6895	-3.2254	1.9E-05	0.00025	0.36068	ENSRNOG	protein_coding
AABR070	-2.423	-5.363	0.00448	0.02059	1	ENSRNOG	lincRNA
Rps6ka2	-2.2921	-4.8975	0.00186	0.01029	1	ENSRNOG	protein_coding
LOC1036	7.22949	150.069	0.0023	0.01223	1	ENSRNOG	lincRNA
Ermard	1.63027	3.0957	4.3E-06	7E-05	0.08282	ENSRNOG	protein_coding
Has1	1.97744	3.93794	0.00138	0.00805	1	ENSRNOG	protein_coding
Mboat7	-5.7254	-52.908	8.3E-06	0.00012	0.15982	ENSRNOG	protein_coding
Prkcg	1.55389	2.93608	0.00535	0.02371	1	ENSRNOG	protein_coding
Mzf1	2.3111	4.96262	4.2E-09	1.4E-07	8.1E-05	ENSRNOG	protein_coding
Zfp579	2.21329	4.63731	0.00188	0.01036	1	ENSRNOG	protein_coding
Ssc5d	1.87683	3.67268	0.00137	0.00798	1	ENSRNOG	protein_coding
Tmem190	-2.1397	-4.4068	0.00041	0.0031	1	ENSRNOG	protein_coding
Brsk1	2.46756	5.53107	0.00025	0.0021	1	ENSRNOG	protein_coding
Tnni3	1.71953	3.29329	0.00723	0.03015	1	ENSRNOG	protein_coding
Eps8l1	-1.6588	-3.1575	0.00568	0.02491	1	ENSRNOG	protein_coding
Lilrb4	-5.1029	-34.366	5.1E-05	0.00058	0.98687	ENSRNOG	protein_coding
LOC1009	-2.562	-5.9054	0.0076	0.03131	1	ENSRNOG	protein_coding
Lig1	-1.4896	-2.8082	2.2E-05	0.00028	0.42727	ENSRNOG	protein_coding
LOC1036	-2.3822	-5.2133	0.00542	0.02397	1	ENSRNOG	protein_coding

Ehd2	1.64246	3.12197	1.9E-11	8.7E-10	3.6E-07	ENSRNOG	protein_coding
Gltscr1	1.81491	3.51837	8.6E-11	3.6E-09	1.7E-06	ENSRNOG	protein_coding
Zfp541	2.34413	5.07755	0.00836	0.03373	1	ENSRNOG	protein_coding
Slc8a2	4.26785	19.2642	0.00479	0.02164	1	ENSRNOG	protein_coding
Gng8	1.48745	2.80393	6.7E-06	0.0001	0.12874	ENSRNOG	protein_coding
Hif3a	3.73403	13.3063	4.7E-05	0.00054	0.90099	ENSRNOG	protein_coding
Micb	1.7856	3.44762	0.00012	0.00115	1	ENSRNOG	protein_coding
Pglyrp1	2.34318	5.0742	0.00066	0.00449	1	ENSRNOG	protein_coding
Dmpk	1.83475	3.56708	4.8E-13	2.8E-11	9.3E-09	ENSRNOG	protein_coding
Fosb	1.93624	3.82706	2.3E-05	0.00029	0.44009	ENSRNOG	protein_coding
Ercc1	-3.2198	-9.3163	6.2E-42	2.6E-39	1.2E-37	ENSRNOG	protein_coding
Cd3eap	-1.5272	-2.8823	2.6E-06	4.5E-05	0.05007	ENSRNOG	protein_coding
Ppp1r13l	-1.7607	-3.3886	1.4E-11	6.8E-10	2.8E-07	ENSRNOG	protein_coding
Bloc1s3	1.43366	2.70131	0.00512	0.02285	1	ENSRNOG	protein_coding
Trappc6a	1.89497	3.71915	6.1E-05	0.00066	1	ENSRNOG	protein_coding
Bcl3	3.05907	8.33436	0.00052	0.00371	1	ENSRNOG	protein_coding
Kcnn4	-1.6805	-3.2053	7.8E-07	1.6E-05	0.01494	ENSRNOG	protein_coding
Plaur	-1.5958	-3.0225	1.6E-09	5.5E-08	3E-05	ENSRNOG	protein_coding
Cadm4	-1.4135	-2.6639	0.0025	0.0131	1	ENSRNOG	protein_coding
Tex101	1.45308	2.73793	0.00034	0.00267	1	ENSRNOG	protein_coding
LOC1036	2.69084	6.4569	1.7E-06	3.1E-05	0.03225	ENSRNOG	protein_coding
Lipe	1.71975	3.29378	0.0005	0.00362	1	ENSRNOG	protein_coding
Cxcl17	2.56263	5.90782	0.00014	0.00133	1	ENSRNOG	protein_coding
AC134759	2.63979	6.23239	0.00522	0.02323	1	ENSRNOG	lincRNA
Erich4	4.15475	17.8116	0.0004	0.00307	1	ENSRNOG	protein_coding
Bckdha	1.57375	2.97676	8.7E-08	2.2E-06	0.00167	ENSRNOG	protein_coding
Cyp2t1	2.64761	6.26627	0.0004	0.00306	1	ENSRNOG	protein_coding
Mia	2.44045	5.4281	0.00503	0.02257	1	ENSRNOG	protein_coding
Numbl	2.22191	4.66509	5.3E-13	3.1E-11	1E-08	ENSRNOG	protein_coding
Sptbn4	2.86627	7.29175	0.00082	0.0053	1	ENSRNOG	protein_coding
Dyrk1b	1.73921	3.33853	1.3E-05	0.00018	0.25332	ENSRNOG	protein_coding
Lrfn1	1.81902	3.52842	0.00099	0.00616	1	ENSRNOG	protein_coding
Zfp36	1.61471	3.06251	5.9E-12	3E-10	1.1E-07	ENSRNOG	protein_coding
Plekhg2	1.45254	2.73689	1.6E-07	3.8E-06	0.00307	ENSRNOG	protein_coding
Ech1	2.46691	5.52857	4.6E-17	4.2E-15	8.8E-13	ENSRNOG	protein_coding
Sipa1l3	-1.4429	-2.7187	9.1E-12	4.5E-10	1.7E-07	ENSRNOG	protein_coding
AABR070	2.771	6.82582	0.0078	0.03192	1	ENSRNOG	protein_coding
LOC1036	2.49516	5.63791	0.001	0.00618	1	ENSRNOG	protein_coding
Ggn	1.64624	3.13017	0.00127	0.00748	1	ENSRNOG	protein_coding
Catsperg	6.05393	66.4376	9.6E-11	4E-09	1.8E-06	ENSRNOG	protein_coding
LOC1025	6.74649	107.373	6.4E-05	0.0007	1	ENSRNOG	protein_coding
LOC1025	7.09067	136.303	0.00325	0.01608	1	ENSRNOG	protein_coding
Syne4l1	1.82172	3.53503	0.00307	0.0154	1	ENSRNOG	protein_coding
Igflr1	1.67828	3.20046	0.00387	0.01838	1	ENSRNOG	protein_coding
Lsr	1.4743	2.77849	0.00325	0.0161	1	ENSRNOG	protein_coding
Fxyd5	-1.7864	-3.4494	1.6E-14	1.2E-12	3.1E-10	ENSRNOG	protein_coding
Lgi4	2.36048	5.1354	0.00058	0.00404	1	ENSRNOG	protein_coding
Gramd1a	1.8024	3.48799	1.9E-06	3.5E-05	0.03671	ENSRNOG	protein_coding
RGD1563	-1.9453	-3.8512	1.1E-05	0.00016	0.21821	ENSRNOG	protein_coding

Clip3	1.63594	3.1079	4.9E-07	1E-05	0.0095	ENSRNOG	protein_coding
RGD1562	4.46833	22.1362	1.3E-09	4.6E-08	2.6E-05	ENSRNOG	protein_coding
Plekhf1	1.34928	2.54785	3E-07	6.7E-06	0.00575	ENSRNOG	protein_coding
Siglec10	-1.8516	-3.6089	1.9E-06	3.5E-05	0.03743	ENSRNOG	protein_coding
Klk6	-4.6859	-25.739	1.1E-06	2.1E-05	0.02099	ENSRNOG	protein_coding
Pold1	-1.3266	-2.5081	0.0008	0.00518	1	ENSRNOG	protein_coding
Fcgrt	1.4722	2.77445	6.3E-07	1.3E-05	0.01203	ENSRNOG	protein_coding
Rpl13a_2	2.42602	5.37411	0.00634	0.02719	1	ENSRNOG	protein_coding
Slc17a7	1.36077	2.56822	0.0055	0.02429	1	ENSRNOG	protein_coding
Ppfia3	2.01416	4.03944	1.4E-06	2.7E-05	0.02708	ENSRNOG	protein_coding
Plekha4	3.08734	8.4993	5.1E-05	0.00058	0.98798	ENSRNOG	protein_coding
Bcat2	1.32447	2.50442	6.9E-09	2.1E-07	0.00013	ENSRNOG	protein_coding
Car11	1.8268	3.54751	3.9E-05	0.00046	0.75844	ENSRNOG	protein_coding
E2f8	-1.8771	-3.6734	8.1E-05	0.00085	1	ENSRNOG	protein_coding
Slc17a6	-2.2132	-4.6369	0.01049	0.04007	1	ENSRNOG	protein_coding
Otud7a	2.74567	6.70703	0.00376	0.01794	1	ENSRNOG	protein_coding
Mcee	1.49583	2.82026	4.2E-06	6.9E-05	0.08092	ENSRNOG	protein_coding
AABR070	-1.5888	-3.008	0.00685	0.02892	1	ENSRNOG	pseudogene
Lysmd4	1.66905	3.18005	7.8E-09	2.4E-07	0.00015	ENSRNOG	protein_coding
Ttc23	1.38622	2.61393	6.5E-07	1.3E-05	0.01258	ENSRNOG	protein_coding
Arrdc4	1.87913	3.67853	0.00052	0.00371	1	ENSRNOG	protein_coding
Nr2f2	2.07661	4.21814	1.9E-10	7.4E-09	3.6E-06	ENSRNOG	protein_coding
AABR070	1.93196	3.81572	0.00115	0.00694	1	ENSRNOG	lincRNA
Sv2b	7.45848	175.883	6.5E-20	7.6E-18	1.2E-15	ENSRNOG	processed_transcript
Acan	-6.2017	-73.601	1.1E-53	1E-50	2.2E-49	ENSRNOG	protein_coding
Mfge8	-1.6569	-3.1534	4.2E-11	1.8E-09	8.1E-07	ENSRNOG	protein_coding
Fanci	-1.4418	-2.7166	0.0005	0.0036	1	ENSRNOG	protein_coding
Ticrr	-1.4978	-2.8242	6.5E-05	0.0007	1	ENSRNOG	protein_coding
Kif7	1.42944	2.69342	1.1E-05	0.00016	0.21403	ENSRNOG	protein_coding
Plin1	3.63005	12.3809	3E-05	0.00036	0.56745	ENSRNOG	protein_coding
Pex11a	2.22496	4.67497	1.5E-10	6E-09	2.9E-06	ENSRNOG	protein_coding
Anpep	2.98983	7.94382	2.9E-13	1.8E-11	5.6E-09	ENSRNOG	protein_coding
Ttll13	1.99591	3.98866	7.1E-06	0.00011	0.13621	ENSRNOG	protein_coding
Prc1	-1.4307	-2.6958	0.00131	0.00768	1	ENSRNOG	protein_coding
Blm	-1.4451	-2.7229	3.3E-05	0.0004	0.63133	ENSRNOG	protein_coding
Zscan2	1.58203	2.9939	0.00108	0.00658	1	ENSRNOG	protein_coding
Slc28a1	1.61444	3.06192	0.0029	0.01473	1	ENSRNOG	protein_coding
Fah	1.57104	2.97118	3.8E-06	6.3E-05	0.07273	ENSRNOG	protein_coding
Prss23	1.58345	2.99686	7E-12	3.6E-10	1.4E-07	ENSRNOG	protein_coding
Sytl2_2	7.56893	189.878	0.00308	0.01544	1	ENSRNOG	protein_coding
Dlg2	3.63722	12.4426	5.9E-06	9.1E-05	0.11252	ENSRNOG	protein_coding
Ddias	-1.5101	-2.8483	0.00028	0.00228	1	ENSRNOG	protein_coding
Tenm4	3.14253	8.83073	0.00927	0.03645	1	ENSRNOG	protein_coding
Usp35	2.08599	4.24566	1E-06	2E-05	0.01934	ENSRNOG	protein_coding
Aamdc	1.49188	2.81255	0.00422	0.01964	1	ENSRNOG	protein_coding
B3gnt6	3.15601	8.9136	0.00013	0.00125	1	ENSRNOG	protein_coding
Lrrc32	5.53178	46.2629	7.5E-42	3E-39	1.4E-37	ENSRNOG	protein_coding
Neu3	1.99094	3.97495	1.5E-11	7.1E-10	2.9E-07	ENSRNOG	protein_coding
Kcne3	5.44816	43.6576	6E-05	0.00066	1	ENSRNOG	protein_coding

Plekhb1	2.98057	7.89297	3.9E-08	1E-06	0.00075	ENSRNOG	protein_coding
Relt	-1.57	-2.969	4.9E-05	0.00056	0.95132	ENSRNOG	protein_coding
Il18bp	1.36013	2.56709	0.0017	0.00954	1	ENSRNOG	protein_coding
Trim68	-1.4041	-2.6464	7.5E-05	0.00079	1	ENSRNOG	protein_coding
Trim34	1.55627	2.94092	5.5E-05	0.00062	1	ENSRNOG	protein_coding
Hpx	1.58014	2.99	0.00967	0.03773	1	ENSRNOG	protein_coding
Scube2	1.90106	3.73487	0.00103	0.00635	1	ENSRNOG	protein_coding
AABR070	-1.7119	-3.276	0.00173	0.00971	1	ENSRNOG	pseudogene
Adm	2.75274	6.73997	2.6E-13	1.6E-11	5.1E-09	ENSRNOG	protein_coding
Mrvi1	2.28437	4.8715	0.00076	0.00499	1	ENSRNOG	protein_coding
Plekha7	2.21381	4.639	5.6E-14	3.7E-12	1.1E-09	ENSRNOG	protein_coding
Xylt1	-5.0823	-33.878	0.00628	0.02699	1	ENSRNOG	protein_coding
Knop1_1	-3.1745	-9.0284	0.00316	0.01573	1	ENSRNOG	protein_coding
Dcun1d3	1.5126	2.85324	2.8E-11	1.3E-09	5.4E-07	ENSRNOG	protein_coding
Tmem159	1.59108	3.01276	1.6E-08	4.6E-07	0.00031	ENSRNOG	protein_coding
Anks4b	2.11112	4.32026	0.00015	0.0014	1	ENSRNOG	protein_coding
Crym	2.82277	7.0752	0.00014	0.00135	1	ENSRNOG	protein_coding
Eef2k	1.4728	2.7756	2.1E-13	1.3E-11	3.9E-09	ENSRNOG	protein_coding
Plk1	-1.5614	-2.9513	0.00242	0.01273	1	ENSRNOG	protein_coding
Nupr1	2.08676	4.24792	1.1E-16	1E-14	2.2E-12	ENSRNOG	protein_coding
Sult1a1	6.29714	78.637	2.8E-56	2.8E-53	5.4E-52	ENSRNOG	protein_coding
Ypel3	3.89874	14.9155	2.4E-24	3.8E-22	4.6E-20	ENSRNOG	protein_coding
Tbx6	2.0811	4.23129	0.00253	0.0132	1	ENSRNOG	protein_coding
Fam57b	5.17545	36.1382	4.1E-06	6.8E-05	0.07878	ENSRNOG	protein_coding
Hirip3	-1.5855	-3.0011	1.9E-05	0.00025	0.3745	ENSRNOG	protein_coding
LOC3089	-1.4363	-2.7063	4.7E-05	0.00054	0.9092	ENSRNOG	protein_coding
Spn	-1.411	-2.6592	0.00085	0.00546	1	ENSRNOG	protein_coding
Ccdc189	1.38476	2.61128	0.00245	0.01285	1	ENSRNOG	protein_coding
Itgax	-2.2398	-4.7232	1.7E-07	4E-06	0.00322	ENSRNOG	protein_coding
AABR070	2.62797	6.18158	0.00165	0.00934	1	ENSRNOG	lincRNA
Acadsb	2.21245	4.63462	2.2E-10	8.6E-09	4.3E-06	ENSRNOG	protein_coding
Hmx2	-1.3529	-2.5542	0.00237	0.01256	1	ENSRNOG	protein_coding
Cpxm2	4.80532	27.9606	1E-14	7.3E-13	1.9E-10	ENSRNOG	protein_coding
Mki67	-1.8187	-3.5276	3.8E-05	0.00045	0.73737	ENSRNOG	protein_coding
Bnip3	1.43059	2.69558	2E-10	7.9E-09	3.9E-06	ENSRNOG	protein_coding
Stk32c	-1.4521	-2.7361	0.00882	0.0351	1	ENSRNOG	protein_coding
Ifitm2	1.69725	3.24281	6.6E-10	2.4E-08	1.3E-05	ENSRNOG	protein_coding
Ifitm1	2.17026	4.50105	3.2E-13	1.9E-11	6.1E-09	ENSRNOG	protein_coding
Ifitm3	3.21191	9.26577	1.2E-23	1.8E-21	2.3E-19	ENSRNOG	protein_coding
Lrrc56	1.64479	3.12701	0.00022	0.00188	1	ENSRNOG	protein_coding
Cracr2b	-4.9684	-31.306	0.01383	0.0499	1	ENSRNOG	protein_coding
Muc6	2.8619	7.26973	0.0018	0.01001	1	ENSRNOG	protein_coding
Dusp8	-1.4863	-2.8018	0.00331	0.01629	1	ENSRNOG	protein_coding
Ifitm10	-2.9686	-7.8275	5.3E-10	2E-08	1E-05	ENSRNOG	protein_coding
Dhcr7	1.43357	2.70115	0.00278	0.01424	1	ENSRNOG	protein_coding
Unc93b1	1.40556	2.6492	7.8E-06	0.00012	0.14935	ENSRNOG	protein_coding
Acy3	2.17672	4.52124	0.00013	0.00128	1	ENSRNOG	protein_coding
Doc2g	1.65321	3.14532	0.01376	0.04967	1	ENSRNOG	protein_coding
Carns1	1.36685	2.57908	0.00036	0.00281	1	ENSRNOG	protein_coding

Pc	2.89689	7.44818	4.6E-38	1.5E-35	8.7E-34	ENSRNOG	protein_coding
Lrfn4	-1.7192	-3.2926	3.5E-15	2.7E-13	6.8E-11	ENSRNOG	protein_coding
LOC1083	1.82941	3.55393	1.2E-12	6.6E-11	2.3E-08	ENSRNOG	protein_coding
Slc29a2	1.62367	3.08158	7.8E-09	2.4E-07	0.00015	ENSRNOG	protein_coding
LOC1083	-1.6427	-3.1225	1.8E-05	0.00023	0.3367	ENSRNOG	protein_coding
Fosl1	-3.0864	-8.4937	6.3E-16	5.2E-14	1.2E-11	ENSRNOG	protein_coding
Ctsw	-3.3121	-9.9324	1.2E-11	5.9E-10	2.4E-07	ENSRNOG	protein_coding
Snx32	2.46224	5.5107	1.1E-06	2.1E-05	0.0205	ENSRNOG	protein_coding
Rn60_1_2	1.78027	3.4349	3.2E-07	7E-06	0.00608	ENSRNOG	lincRNA
Cdc42ep2	2.48505	5.59853	6.6E-08	1.7E-06	0.00126	ENSRNOG	protein_coding
Pola2	-1.6475	-3.133	1.1E-06	2.1E-05	0.02031	ENSRNOG	protein_coding
LOC1009	-1.6828	-3.2105	0.00062	0.00427	1	ENSRNOG	protein_coding
Macrod1	1.61114	3.05492	0.00114	0.0069	1	ENSRNOG	protein_coding
RGD1560	1.46251	2.75588	0.00084	0.00536	1	ENSRNOG	protein_coding
Pla2g16	1.72843	3.31367	0.00445	0.0205	1	ENSRNOG	protein_coding
Lrrn4cl	1.89384	3.71624	0.00079	0.00516	1	ENSRNOG	protein_coding
Scgb2a2	10.7346	1703.87	7.6E-45	3.7E-42	1.5E-40	ENSRNOG	protein_coding
Scgb2a1	6.67081	101.886	1.5E-27	2.8E-25	3E-23	ENSRNOG	protein_coding
Incenp	-1.4726	-2.7753	4.3E-06	7E-05	0.08256	ENSRNOG	protein_coding
Best1	2.1366	4.39723	1E-09	3.6E-08	2E-05	ENSRNOG	protein_coding
AABR070	-1.9294	-3.8089	0.01032	0.03956	1	ENSRNOG	pseudogene
Rab3il1	2.23206	4.69806	7.9E-07	1.6E-05	0.01519	ENSRNOG	protein_coding
AABR070	-1.5593	-2.9471	0.00047	0.00343	1	ENSRNOG	protein_coding
AABR070	4.36933	20.668	3.8E-05	0.00045	0.7225	ENSRNOG	lincRNA
Ms4a6c	-1.6389	-3.1143	0.00024	0.00205	1	ENSRNOG	protein_coding
Ms4a6bl	3.57527	11.9197	9.3E-10	3.4E-08	1.8E-05	ENSRNOG	protein_coding
AABR070	3.36685	10.3163	3.7E-06	6.1E-05	0.07034	ENSRNOG	protein_coding
AABR070	6.52539	92.1169	9.7E-10	3.5E-08	1.9E-05	ENSRNOG	pseudogene
Dtx4	2.61685	6.1341	5.3E-26	8.9E-24	1E-21	ENSRNOG	protein_coding
LOC1009	5.4717	44.3758	6.3E-05	0.00068	1	ENSRNOG	protein_coding
Trpm6	-2.7198	-6.588	3.8E-07	8.3E-06	0.00735	ENSRNOG	protein_coding
Pcsk5	-2.3175	-4.9846	9.6E-16	7.8E-14	1.8E-11	ENSRNOG	protein_coding
Gda	2.29026	4.89145	0.00951	0.03727	1	ENSRNOG	protein_coding
Trpm3	4.877	29.3848	8E-19	8.5E-17	1.5E-14	ENSRNOG	protein_coding
Klf9	1.75575	3.37702	4.6E-15	3.6E-13	8.9E-11	ENSRNOG	protein_coding
Mamdc2	-3.2885	-9.7712	2.1E-08	5.9E-07	0.0004	ENSRNOG	protein_coding
Dmrt2	-2.5309	-5.7795	1.1E-07	2.6E-06	0.00207	ENSRNOG	protein_coding
Jak2	2.47083	5.54364	5.9E-18	5.9E-16	1.1E-13	ENSRNOG	protein_coding
NEWGEN	7.05231	132.727	0.00346	0.01687	1	ENSRNOG	protein_coding
Acta2	-2.7543	-6.7472	2.5E-27	4.4E-25	4.9E-23	ENSRNOG	protein_coding
Ch25h	2.72619	6.61704	0.00019	0.00168	1	ENSRNOG	protein_coding
Kif20b	-1.5223	-2.8724	0.0002	0.00178	1	ENSRNOG	protein_coding
Ankrd1	-2.4767	-5.566	1.8E-17	1.7E-15	3.4E-13	ENSRNOG	protein_coding
Ppp1r3c	2.44912	5.46082	9.9E-11	4.1E-09	1.9E-06	ENSRNOG	protein_coding
Kif11	-1.6415	-3.1198	0.00016	0.00145	1	ENSRNOG	protein_coding
Cyp26c1	-2.2433	-4.7347	0.00012	0.00116	1	ENSRNOG	protein_coding
Cep55	-1.559	-2.9465	0.0006	0.00419	1	ENSRNOG	protein_coding
Plce1	2.34662	5.08632	9.8E-05	0.00099	1	ENSRNOG	protein_coding
Hells	-1.3943	-2.6286	0.00068	0.0046	1	ENSRNOG	protein_coding

Aldh18a1	3.63338	12.4096	0.00051	0.0037	1	ENSRNOG	protein_coding
Sorbs1	3.42063	10.7081	7.5E-28	1.4E-25	1.4E-23	ENSRNOG	protein_coding
Pik3ap1	4.99789	31.9533	1.6E-24	2.6E-22	3.1E-20	ENSRNOG	protein_coding
AABR070	3.77092	13.6508	0.01014	0.03905	1	ENSRNOG	pseudogene
Rn60_1_2	1.93434	3.82203	0.00049	0.00354	1	ENSRNOG	antisense_RNA
LOC6814	1.72886	3.31466	0.0128	0.04687	1	ENSRNOG	protein_coding
Scd2	2.84085	7.16441	8.5E-17	7.7E-15	1.6E-12	ENSRNOG	protein_coding
Scd	1.56057	2.9497	0.01144	0.04287	1	ENSRNOG	protein_coding
Sema4g	1.71523	3.28349	0.00031	0.00246	1	ENSRNOG	protein_coding
Pdzd7	1.46548	2.76155	0.00253	0.01322	1	ENSRNOG	protein_coding
LOC1025	1.93643	3.82756	0.01143	0.04285	1	ENSRNOG	lincRNA
Npm3	-1.4833	-2.7958	0.00217	0.01167	1	ENSRNOG	protein_coding
LOC1036	3.18507	9.095	0.0004	0.00305	1	ENSRNOG	protein_coding
Cfap43	1.73979	3.33986	5.4E-07	1.1E-05	0.01034	ENSRNOG	protein_coding
LOC1036	6.60128	97.0919	0.00569	0.02494	1	ENSRNOG	protein_coding
RGD1561	1.9974	3.99281	0.00053	0.00378	1	ENSRNOG	protein_coding
Dusp5	-1.378	-2.5991	6.5E-06	0.0001	0.12549	ENSRNOG	protein_coding
Rbm20	1.78597	3.44851	0.00015	0.00137	1	ENSRNOG	protein_coding
Pdcd4	1.90382	3.74203	1E-11	5E-10	2E-07	ENSRNOG	protein_coding
Ablim1	2.14447	4.42131	6.6E-18	6.5E-16	1.3E-13	ENSRNOG	protein_coding
Snu13	-1.4671	-2.7647	9.6E-07	1.9E-05	0.01852	ENSRNOG	protein_coding
Nanos1	-1.9691	-3.9153	0.00222	0.01192	1	ENSRNOG	protein_coding
Grk5	1.61382	3.06062	1.1E-11	5.2E-10	2.1E-07	ENSRNOG	protein_coding
ENSRNO	1.38431	2.61047	1.9E-05	0.00024	0.3566	ENSRNOG	protein_coding
Ces2c	1.32515	2.50559	8.3E-05	0.00086	1	ENSRNOG	protein_coding
LOC1025	1.3929	2.62607	0.00326	0.0161	1	ENSRNOG	lincRNA
LOC1036	-1.6665	-3.1745	0.00029	0.00234	1	ENSRNOG	lincRNA
ENSRNO	6.23145	75.137	7.9E-15	5.8E-13	1.5E-10	ENSRNOG	protein_coding
Ciita	-2.5612	-5.9021	5.2E-09	1.7E-07	1E-04	ENSRNOG	protein_coding
Tnfrsf12a	-1.4264	-2.6877	2.5E-06	4.4E-05	0.04856	ENSRNOG	protein_coding
Cldn6	3.5136	11.4209	0.01369	0.04943	1	ENSRNOG	protein_coding
LOC1001	-1.3648	-2.5754	0.00067	0.00456	1	ENSRNOG	protein_coding
Ccnf	-1.9082	-3.7535	3E-05	0.00036	0.57181	ENSRNOG	protein_coding
Caskin1	1.52669	2.88123	0.00018	0.00159	1	ENSRNOG	protein_coding
Npw	-1.7623	-3.3924	0.00307	0.0154	1	ENSRNOG	protein_coding
AC11518	1.85275	3.61187	0.0051	0.02281	1	ENSRNOG	protein_coding
Mapk8ip3	1.61264	3.05812	2.6E-07	5.8E-06	0.00498	ENSRNOG	protein_coding
Tmem204	2.70211	6.50753	6.2E-21	7.7E-19	1.2E-16	ENSRNOG	protein_coding
Chtf18	-1.7176	-3.289	8.2E-06	0.00012	0.15793	ENSRNOG	protein_coding
Wfikkn1	1.70331	3.25646	0.00083	0.00534	1	ENSRNOG	protein_coding
Stc2	-1.6024	-3.0365	0.00015	0.00139	1	ENSRNOG	protein_coding
Crebrf	1.58516	3.00042	3.7E-06	6.2E-05	0.07162	ENSRNOG	protein_coding
Neurl1b	-1.7584	-3.3833	2.2E-05	0.00028	0.42614	ENSRNOG	protein_coding
Adra1b	4.8115	28.0806	5.7E-05	0.00063	1	ENSRNOG	protein_coding
AABR070	6.13083	70.075	0.01008	0.0389	1	ENSRNOG	lincRNA
Cyfip2	1.86474	3.64202	0.00015	0.00141	1	ENSRNOG	protein_coding
Ifi47	2.8151	7.0377	0.00043	0.00325	1	ENSRNOG	protein_coding
Flt4	-2.8036	-6.9816	2.5E-16	2.1E-14	4.8E-12	ENSRNOG	protein_coding
Sqstm1	1.41557	2.66766	2.4E-06	4.2E-05	0.04533	ENSRNOG	protein_coding

Zfp354c	1.72589	3.30784	1.9E-07	4.4E-06	0.00367	ENSRNOG	protein_coding
Phykpl	7.69594	207.353	0.00119	0.00712	1	ENSRNOG	protein_coding
Hnrnpab	-1.3663	-2.5781	0.00032	0.00254	1	ENSRNOG	protein_coding
N4bp3	-1.6155	-3.0641	0.01279	0.04686	1	ENSRNOG	protein_coding
Cdkl3	1.36184	2.57014	0.00019	0.0017	1	ENSRNOG	protein_coding
Tcf7	1.61063	3.05386	0.00383	0.01823	1	ENSRNOG	protein_coding
Shroom1	-1.5496	-2.9273	5E-14	3.4E-12	9.6E-10	ENSRNOG	protein_coding
Pdlim4	1.37283	2.58978	0.00239	0.01264	1	ENSRNOG	protein_coding
Gpx3	2.32967	5.02691	1.7E-06	3.1E-05	0.03183	ENSRNOG	protein_coding
Fat2	-4.4714	-22.184	1.6E-11	7.4E-10	3E-07	ENSRNOG	protein_coding
Sparc	1.50611	2.84042	4.6E-09	1.5E-07	8.9E-05	ENSRNOG	protein_coding
Glra1	-3.498	-11.298	1.6E-07	3.9E-06	0.00317	ENSRNOG	protein_coding
Faxdc2	1.86767	3.64942	0.00017	0.00156	1	ENSRNOG	protein_coding
Nlrp3	-2.6748	-6.3855	7.5E-07	1.5E-05	0.01447	ENSRNOG	protein_coding
Flcn	1.45524	2.74202	1.9E-07	4.4E-06	0.00359	ENSRNOG	protein_coding
Nt5m	1.48614	2.80138	0.00215	0.0116	1	ENSRNOG	protein_coding
Rasd1	1.90267	3.73904	0.0001	0.00101	1	ENSRNOG	protein_coding
Shmt1	-1.3677	-2.5806	0.00013	0.00124	1	ENSRNOG	protein_coding
Slc47a1	-5.9883	-63.483	1.8E-37	5.5E-35	3.4E-33	ENSRNOG	protein_coding
Trpv2	4.1662	17.9536	1.5E-06	2.8E-05	0.02874	ENSRNOG	protein_coding
Dnah9	3.37837	10.399	2.6E-05	0.00032	0.49143	ENSRNOG	protein_coding
Myh2	-6.4711	-88.716	3.3E-74	1.1E-70	6.3E-70	ENSRNOG	protein_coding
Myh8	-6.2429	-75.733	3.2E-07	7.1E-06	0.00616	ENSRNOG	protein_coding
Usp43	-2.6078	-6.0957	0.00059	0.00411	1	ENSRNOG	protein_coding
Aurkb	-1.6039	-3.0396	2.5E-05	0.00031	0.47267	ENSRNOG	protein_coding
Per1	3.29535	9.81743	1.1E-39	3.7E-37	2E-35	ENSRNOG	protein_coding
Chrnb1	-1.9973	-3.9924	1.1E-05	0.00016	0.21802	ENSRNOG	protein_coding
Fgf11	2.25633	4.77776	1.7E-05	0.00023	0.33631	ENSRNOG	protein_coding
Slc2a4	3.44075	10.8585	1.6E-07	3.7E-06	0.003	ENSRNOG	protein_coding
Dlg4	1.47732	2.78431	0.00077	0.00505	1	ENSRNOG	protein_coding
Slc16a11	-2.1273	-4.3691	7.3E-12	3.7E-10	1.4E-07	ENSRNOG	protein_coding
Vmo1	2.57778	5.97022	0.01311	0.04775	1	ENSRNOG	protein_coding
Chrne	3.06899	8.39185	0.00379	0.01806	1	ENSRNOG	protein_coding
Gp1ba	3.02231	8.12469	0.00049	0.00358	1	ENSRNOG	protein_coding
Tekt1	2.23452	4.70607	0.00443	0.02041	1	ENSRNOG	protein_coding
Itgae	1.64822	3.13447	0.00021	0.00181	1	ENSRNOG	protein_coding
Gsg2	-1.3382	-2.5283	1.1E-05	0.00015	0.20953	ENSRNOG	protein_coding
Aspa	1.51246	2.85296	1.5E-06	2.8E-05	0.02916	ENSRNOG	protein_coding
Rn60_10_	-2.973	-7.8519	0.00737	0.03053	1	ENSRNOG	unprocessed_pseudogene
Coro6	4.47799	22.2848	1E-34	2.6E-32	1.9E-30	ENSRNOG	protein_coding
Tusc5	4.59972	24.2467	0.00296	0.015	1	ENSRNOG	protein_coding
Inpp5k	1.54324	2.91448	1.5E-08	4.4E-07	0.0003	ENSRNOG	protein_coding
Pipox	8.34861	325.973	6E-09	1.9E-07	0.00012	ENSRNOG	protein_coding
Foxn1	-2.3725	-5.1783	3.8E-06	6.4E-05	0.0738	ENSRNOG	protein_coding
Sarm1	1.48313	2.79554	0.00052	0.00372	1	ENSRNOG	protein_coding
Lyrm9	-2.0945	-4.2707	0.00013	0.00123	1	ENSRNOG	protein_coding
LOC4979	-3.0589	-8.3335	3.9E-05	0.00046	0.74381	ENSRNOG	protein_coding
Evi2a	-4.1649	-17.937	1.5E-18	1.5E-16	2.8E-14	ENSRNOG	protein_coding
Rhbdl3	1.41291	2.66273	0.0054	0.02391	1	ENSRNOG	protein_coding

Ccl2	-2.7507	-6.7306	3.9E-09	1.3E-07	7.5E-05	ENSRNOG	protein_coding
Ccl7	-1.7722	-3.4157	0.00026	0.00218	1	ENSRNOG	protein_coding
Slfn5	3.57752	11.9383	1.4E-17	1.3E-15	2.6E-13	ENSRNOG	protein_coding
Slfn13	-1.3294	-2.513	4.9E-06	7.9E-05	0.09439	ENSRNOG	protein_coding
Rasl10b	3.97492	15.7242	0.00045	0.00336	1	ENSRNOG	protein_coding
LOC6890	2.25392	4.76977	2E-07	4.6E-06	0.00378	ENSRNOG	protein_coding
Mmp28	2.06538	4.18544	9.4E-10	3.4E-08	1.8E-05	ENSRNOG	protein_coding
Anos1	2.85291	7.22455	0.00251	0.01311	1	ENSRNOG	protein_coding
Wfdc18	5.99997	63.9987	3.2E-21	4.2E-19	6.2E-17	ENSRNOG	protein_coding
Brip1	-1.3963	-2.6323	0.00013	0.00126	1	ENSRNOG	protein_coding
Ypel2	2.48839	5.61151	4.7E-15	3.6E-13	9E-11	ENSRNOG	protein_coding
Prr11	-1.9308	-3.8128	1.5E-05	0.0002	0.28277	ENSRNOG	protein_coding
Sept4	4.26165	19.1816	1.6E-10	6.2E-09	3E-06	ENSRNOG	protein_coding
Mmd	-3.3834	-10.435	9.4E-51	5.8E-48	1.8E-46	ENSRNOG	protein_coding
Cacna1g	1.91681	3.77587	0.00058	0.00408	1	ENSRNOG	protein_coding
Chad	2.49254	5.62768	0.00024	0.002	1	ENSRNOG	protein_coding
Eme1	-1.8405	-3.5814	9.2E-05	0.00094	1	ENSRNOG	protein_coding
AABR070	2.98271	7.90467	1.2E-15	9.4E-14	2.2E-11	ENSRNOG	lincRNA
Sgca	-3.1887	-9.1177	2.6E-11	1.1E-09	4.9E-07	ENSRNOG	protein_coding
Pdk2	1.48493	2.79903	1.5E-07	3.5E-06	0.00284	ENSRNOG	protein_coding
Ngfr	-3.4609	-11.012	0.00133	0.00778	1	ENSRNOG	protein_coding
Ttll6	2.32914	5.02505	0.0024	0.01269	1	ENSRNOG	protein_coding
Pnpo	1.5896	3.00965	2.2E-06	4E-05	0.04316	ENSRNOG	protein_coding
Scrn2	1.69244	3.23203	9.9E-06	0.00014	0.19021	ENSRNOG	protein_coding
Tbkbp1	-3.0099	-8.0553	0.00247	0.01295	1	ENSRNOG	protein_coding
Srcin1	2.53986	5.81532	0.00552	0.02437	1	ENSRNOG	protein_coding
LOC6911	-1.3561	-2.5599	0.00878	0.03498	1	ENSRNOG	protein_coding
Cacnb1	2.85473	7.23366	1.5E-05	0.0002	0.2883	ENSRNOG	protein_coding
Fbxl20	1.4558	2.74308	2.3E-07	5.2E-06	0.00438	ENSRNOG	protein_coding
Ppp1r1b	5.01924	32.4296	3.3E-31	6.8E-29	6.3E-27	ENSRNOG	protein_coding
Thra	1.37751	2.59819	1.3E-07	3.1E-06	0.00247	ENSRNOG	protein_coding
Cdc6	-1.3427	-2.5362	0.00147	0.00848	1	ENSRNOG	protein_coding
Top2a	-1.7792	-3.4325	7.3E-05	0.00077	1	ENSRNOG	protein_coding
Krt222	2.21203	4.63326	0.00988	0.03834	1	ENSRNOG	protein_coding
Krt28	2.31591	4.97918	1E-05	0.00015	0.20124	ENSRNOG	protein_coding
Krt23	-3.3244	-10.017	8.3E-08	2.1E-06	0.00159	ENSRNOG	protein_coding
Krtap1-5	-3.5183	-11.458	0.00246	0.0129	1	ENSRNOG	protein_coding
Dhx58	3.26806	9.63351	6E-09	1.9E-07	0.00012	ENSRNOG	protein_coding
Psmc3ip	-1.6281	-3.0911	2.1E-05	0.00027	0.40167	ENSRNOG	protein_coding
Fam134c	1.71116	3.27425	6.5E-15	4.9E-13	1.3E-10	ENSRNOG	protein_coding
Rn50_10_	6.27503	77.4415	0.01148	0.04298	1	ENSRNOG	antisense_RNA
Arl4d	2.07654	4.21794	1.5E-08	4.2E-07	0.00028	ENSRNOG	protein_coding
Mpp2	2.90215	7.4754	0.00314	0.01567	1	ENSRNOG	protein_coding
LOC3035	-1.7435	-3.3485	0.0003	0.00242	1	ENSRNOG	protein_coding
Itga2b	2.77366	6.8384	0.00023	0.00197	1	ENSRNOG	protein_coding
Kif18b	-1.5101	-2.8484	0.00054	0.00385	1	ENSRNOG	protein_coding
Cyb561	1.65898	3.15794	2.2E-06	3.9E-05	0.04163	ENSRNOG	protein_coding
Ace	4.19666	18.3367	5.3E-12	2.8E-10	1E-07	ENSRNOG	protein_coding
Kcnh6	-4.7548	-26.998	1.1E-19	1.3E-17	2.1E-15	ENSRNOG	protein_coding

Kpna2	-1.4908	-2.8105	0.00041	0.0031	1	ENSRNOG	protein_coding
AABR070	2.16055	4.47085	0.00246	0.01292	1	ENSRNOG	lincRNA
Arsg	1.76706	3.4036	1.9E-09	6.4E-08	3.6E-05	ENSRNOG	protein_coding
Wipi1	1.35009	2.54928	2.4E-08	6.7E-07	0.00047	ENSRNOG	protein_coding
Kcnj16	5.14111	35.2881	7.5E-49	4.2E-46	1.4E-44	ENSRNOG	protein_coding
Kcnj2	1.55965	2.94781	5.4E-08	1.4E-06	0.00103	ENSRNOG	protein_coding
Itgb4	4.45806	21.979	1.3E-71	3.2E-68	2.6E-67	ENSRNOG	protein_coding
Unc13d	1.98337	3.95415	0.00512	0.02285	1	ENSRNOG	protein_coding
AABR070	3.77097	13.6513	0.00092	0.00579	1	ENSRNOG	lincRNA
Sphk1	1.32651	2.50795	9.8E-09	2.9E-07	0.00019	ENSRNOG	protein_coding
St6galnac	-5.6443	-50.015	2.5E-60	3.2E-57	4.8E-56	ENSRNOG	protein_coding
Tmc8	1.71156	3.27514	0.01241	0.0458	1	ENSRNOG	protein_coding
LOC6882	1.56652	2.9619	0.00777	0.03184	1	ENSRNOG	protein_coding
Cep295nl	2.40432	5.29387	1.3E-11	6.3E-10	2.5E-07	ENSRNOG	protein_coding
C1qtnf1	4.50435	22.6957	1.8E-37	5.7E-35	3.5E-33	ENSRNOG	protein_coding
Engase	1.52928	2.88642	1.2E-07	2.9E-06	0.00233	ENSRNOG	protein_coding
Ccdc40	1.99455	3.98491	0.0003	0.00243	1	ENSRNOG	protein_coding
LOC1003	-1.6484	-3.1348	1.9E-08	5.4E-07	0.00037	ENSRNOG	protein_coding
Notum	2.68864	6.44704	9.1E-11	3.8E-09	1.8E-06	ENSRNOG	protein_coding
Rac3	1.95346	3.87303	0.00019	0.00166	1	ENSRNOG	protein_coding
Dcxr	4.11692	17.3507	3.7E-33	8.9E-31	7.1E-29	ENSRNOG	protein_coding
LOC6883	6.96302	124.761	0.00287	0.01462	1	ENSRNOG	protein_coding
LOC1025	2.41596	5.33675	2.5E-07	5.7E-06	0.0048	ENSRNOG	lincRNA
Rab40b	2.56746	5.92765	2.3E-06	4.1E-05	0.04501	ENSRNOG	protein_coding
Jam2	-3	-8.0003	2.6E-05	0.00033	0.50477	ENSRNOG	protein_coding
Adamts1	2.07143	4.20304	1.4E-15	1.1E-13	2.6E-11	ENSRNOG	protein_coding
Map3k7cl	3.89116	14.8373	1.4E-53	1.2E-50	2.6E-49	ENSRNOG	protein_coding
Kcne2	2.20389	4.6072	0.00558	0.02456	1	ENSRNOG	protein_coding
Runx1	-1.5639	-2.9565	1.1E-07	2.7E-06	0.00216	ENSRNOG	protein_coding
Chaf1b	-1.5311	-2.8901	0.00015	0.00141	1	ENSRNOG	protein_coding
Kcnj15	4.78826	27.6319	5.3E-08	1.4E-06	0.00102	ENSRNOG	protein_coding
LOC1009	-8.0666	-268.1	3.4E-05	0.00041	0.65838	ENSRNOG	protein_coding
Ripk4	-4.6335	-24.82	0.00032	0.00257	1	ENSRNOG	protein_coding
Senp7	1.71741	3.28845	9.5E-08	2.3E-06	0.00182	ENSRNOG	protein_coding
Hmgb2	-1.4098	-2.657	0.0001	0.00101	1	ENSRNOG	protein_coding
Cblb	2.75713	6.76049	6E-22	8.1E-20	1.2E-17	ENSRNOG	protein_coding
RGD1310	-1.3511	-2.551	0.00544	0.02405	1	ENSRNOG	protein_coding
Plcxd2	-3.1501	-8.8772	6.5E-06	1E-04	0.12466	ENSRNOG	protein_coding
LOC1036	-1.6071	-3.0464	0.00555	0.02444	1	ENSRNOG	protein_coding
Btla	2.62192	6.1557	0.00759	0.03128	1	ENSRNOG	protein_coding
Hgd	7.52224	183.831	0.002	0.01092	1	ENSRNOG	protein_coding
Stxbp5l	2.83047	7.11304	0.00097	0.00602	1	ENSRNOG	protein_coding
Polq	-1.6891	-3.2246	4.1E-05	0.00048	0.79092	ENSRNOG	protein_coding
Mylk	1.85982	3.62962	0.00264	0.01367	1	ENSRNOG	protein_coding
Muc13	-3.4635	-11.031	0.00743	0.03075	1	ENSRNOG	protein_coding
Lmln	-1.9071	-3.7505	3.3E-07	7.2E-06	0.00625	ENSRNOG	protein_coding
LOC1009	6.52761	92.2583	5E-05	0.00057	0.96265	ENSRNOG	protein_coding
Fam43a	3.74186	13.3786	1.9E-38	6.5E-36	3.6E-34	ENSRNOG	protein_coding
Atp13a4	-2.7443	-6.7006	1E-07	2.6E-06	0.00201	ENSRNOG	protein_coding

Cldn1	1.85808	3.62526	0.01196	0.04441	1	ENSRNOG	protein_coding
Tp63	-1.3831	-2.6082	0.01048	0.04004	1	ENSRNOG	protein_coding
Bcl6	1.57279	2.9748	2E-06	3.7E-05	0.03936	ENSRNOG	protein_coding
Rtp4	2.87904	7.3566	0.00349	0.01696	1	ENSRNOG	protein_coding
Masp1	2.35775	5.1257	1.7E-16	1.5E-14	3.3E-12	ENSRNOG	protein_coding
Kng1	5.33481	40.3589	5.8E-27	1E-24	1.1E-22	ENSRNOG	protein_coding
Fetub	9.59301	772.293	7.5E-32	1.6E-29	1.4E-27	ENSRNOG	protein_coding
LOC1009	7.65042	200.912	2.6E-14	1.8E-12	5.1E-10	ENSRNOG	protein_coding
LOC1009	-7.7489	-215.11	3.7E-05	0.00044	0.70914	ENSRNOG	protein_coding
LOC1083	1.55471	2.93775	4.2E-06	6.9E-05	0.08028	ENSRNOG	protein_coding
Fam131a	2.40652	5.30194	6E-06	9.3E-05	0.11475	ENSRNOG	protein_coding
Klhl24	3.19524	9.15933	2.8E-17	2.7E-15	5.5E-13	ENSRNOG	protein_coding
Ranbp1	-1.5526	-2.9334	1.8E-06	3.3E-05	0.03465	ENSRNOG	protein_coding
Rtn4r	-1.3425	-2.536	0.00082	0.00527	1	ENSRNOG	protein_coding
Prodh1	4.406	21.2002	5.6E-12	2.9E-10	1.1E-07	ENSRNOG	protein_coding
Aifm3	1.71995	3.29424	0.00077	0.00505	1	ENSRNOG	protein_coding
RGD1308	-1.9785	-3.941	0.00359	0.01735	1	ENSRNOG	protein_coding
Ypel1	-1.8413	-3.5832	0.0001	0.00102	1	ENSRNOG	protein_coding
ENSRNO	-2.3542	-5.1133	1.3E-07	3.1E-06	0.00243	ENSRNOG	protein_coding
Cebpd	2.53592	5.79948	1.2E-12	6.9E-11	2.4E-08	ENSRNOG	protein_coding
Snai2	2.61342	6.11954	5.8E-11	2.4E-09	1.1E-06	ENSRNOG	protein_coding
Brca2	-1.4775	-2.7846	3.2E-07	7E-06	0.00612	ENSRNOG	protein_coding
N4bp2l1	1.96757	3.9111	0.00328	0.01616	1	ENSRNOG	protein_coding
Rfc3	-1.4266	-2.6882	0.00016	0.00143	1	ENSRNOG	protein_coding
Tgfbr3l	1.72865	3.31417	0.00668	0.02835	1	ENSRNOG	protein_coding
Fry	1.40355	2.64552	4.8E-06	7.7E-05	0.09221	ENSRNOG	protein_coding
Hsph1	-1.3744	-2.5925	2.1E-08	5.9E-07	0.0004	ENSRNOG	protein_coding
Tex26	2.78008	6.86892	0.00354	0.01717	1	ENSRNOG	protein_coding
Mtus2	1.33971	2.531	1.2E-05	0.00017	0.2327	ENSRNOG	protein_coding
Slc46a3	1.5929	3.01656	0.00068	0.00458	1	ENSRNOG	protein_coding
Flt1	1.8862	3.69662	0.00333	0.01635	1	ENSRNOG	protein_coding
Fscn1	-1.6505	-3.1394	5.3E-09	1.7E-07	0.0001	ENSRNOG	protein_coding
Card11	-3.5959	-12.091	0.00571	0.025	1	ENSRNOG	processed_transcript
AABR070	-2.5452	-5.8369	0.00039	0.003	1	ENSRNOG	lincRNA
Cyp3a9_2	-6.1889	-72.954	4.8E-24	7.3E-22	9.2E-20	ENSRNOG	protein_coding
Mcm7	-1.4989	-2.8262	6.4E-05	0.00069	1	ENSRNOG	protein_coding
LOC1025	2.36533	5.15272	4.1E-08	1.1E-06	0.00079	ENSRNOG	lincRNA
LOC1009	3.20743	9.23704	2.8E-13	1.7E-11	5.4E-09	ENSRNOG	protein_coding
Agfg2	1.57509	2.97954	1.2E-10	4.8E-09	2.3E-06	ENSRNOG	protein_coding
Irs3	2.89389	7.43272	2.2E-06	3.9E-05	0.04202	ENSRNOG	protein_coding
Cldn15	1.71629	3.28591	3.9E-05	0.00046	0.74734	ENSRNOG	protein_coding
Rasa4	2.06374	4.18067	0.00295	0.01492	1	ENSRNOG	protein_coding
Upk3b	2.59444	6.03953	0.00028	0.00233	1	ENSRNOG	protein_coding
Rhbdd2	-1.4175	-2.6712	7.6E-06	0.00012	0.14684	ENSRNOG	protein_coding
Mlxipl	5.76986	54.5633	1.6E-14	1.2E-12	3.1E-10	ENSRNOG	processed_transcript
Vps37d	1.70723	3.26534	0.00239	0.01261	1	ENSRNOG	protein_coding
Kctd7	1.60795	3.04818	4.7E-06	7.6E-05	0.09039	ENSRNOG	protein_coding
Phkg1	2.43534	5.4089	3.5E-05	0.00042	0.6823	ENSRNOG	protein_coding
Rflna	3.92044	15.1416	0.00368	0.01765	1	ENSRNOG	protein_coding

Rilpl2	1.41738	2.67099	1.1E-08	3.2E-07	0.00021	ENSRNOG	protein_coding
Abcb9	2.54257	5.82625	0.0011	0.00666	1	ENSRNOG	protein_coding
Ccdc62	1.3967	2.63299	1.5E-05	0.0002	0.28894	ENSRNOG	protein_coding
Kntc1	-1.4279	-2.6905	0.00049	0.00355	1	ENSRNOG	protein_coding
AABR070	1.83569	3.56941	0.00045	0.00335	1	ENSRNOG	lincRNA
P2rx7	3.14393	8.83926	4.7E-05	0.00054	0.90595	ENSRNOG	protein_coding
Oas1a	7.13974	141.018	1.1E-22	1.6E-20	2.1E-18	ENSRNOG	protein_coding
Oas1b	5.15066	35.5225	3.1E-22	4.3E-20	6E-18	ENSRNOG	protein_coding
Oas1e	5.21701	37.1942	0.00035	0.00277	1	ENSRNOG	protein_coding
Oas3	3.25375	9.53843	3E-06	5.2E-05	0.05846	ENSRNOG	protein_coding
Oas1i	7.0366	131.289	0.00453	0.02078	1	ENSRNOG	protein_coding
Rfc5	-1.5896	-3.0096	8.6E-06	0.00013	0.16462	ENSRNOG	protein_coding
Sirt4	1.53553	2.89895	1.8E-05	0.00024	0.34917	ENSRNOG	protein_coding
Hnf1a	3.50843	11.38	0.01367	0.04938	1	ENSRNOG	protein_coding
Acacb	2.03004	4.08417	0.00204	0.01108	1	ENSRNOG	protein_coding
Sez6l	1.9337	3.82034	0.00025	0.00208	1	ENSRNOG	protein_coding
Tpst2	-1.4615	-2.754	1.4E-09	4.8E-08	2.7E-05	ENSRNOG	protein_coding
Pole	-1.4911	-2.811	9.2E-05	0.00095	1	ENSRNOG	protein_coding
LOC6804	-1.9616	-3.8949	0.00016	0.00147	1	ENSRNOG	processed_pseudogene
Cntnap5c	2.50591	5.68007	0.0044	0.02031	1	ENSRNOG	protein_coding
Tfcp2l1	2.44648	5.45085	1.7E-15	1.3E-13	3.2E-11	ENSRNOG	protein_coding
Gli2	1.58073	2.99121	3.7E-11	1.6E-09	7.1E-07	ENSRNOG	protein_coding
Epb41l5	1.77352	3.41887	1.4E-12	7.9E-11	2.8E-08	ENSRNOG	protein_coding
Sctr	2.88116	7.36744	8.9E-05	0.00092	1	ENSRNOG	protein_coding
Slc35f5	1.4002	2.63939	2E-10	7.9E-09	3.9E-06	ENSRNOG	protein_coding
Nckap5	7.18385	145.397	1.3E-07	3.1E-06	0.00247	ENSRNOG	protein_coding
LOC1009	-6.7078	-104.53	0.00069	0.00465	1	ENSRNOG	protein_coding
Map3k19	-2.5584	-5.8905	6.3E-06	9.7E-05	0.1207	ENSRNOG	protein_coding
Mcm6	-1.3769	-2.5972	0.00025	0.00213	1	ENSRNOG	protein_coding
Cd55	-1.9712	-3.9209	0.00039	0.00298	1	ENSRNOG	protein_coding
Ikbke	2.74466	6.70232	6.3E-12	3.2E-10	1.2E-07	ENSRNOG	protein_coding
Pm20d1	2.90729	7.50206	0.01347	0.04882	1	ENSRNOG	protein_coding
Pik3c2b	-1.7001	-3.2492	3.1E-05	0.00038	0.60507	ENSRNOG	protein_coding
Sox13	4.9233	30.3432	3.4E-11	1.5E-09	6.6E-07	ENSRNOG	protein_coding
Prelp	3.72767	13.2477	8.4E-08	2.1E-06	0.00161	ENSRNOG	protein_coding
Kdm5b	1.83727	3.57332	1.6E-09	5.5E-08	3.1E-05	ENSRNOG	protein_coding
Ube2t	-1.6334	-3.1024	0.00126	0.00747	1	ENSRNOG	protein_coding
Ptprv	2.51863	5.73038	6.7E-06	0.0001	0.12949	ENSRNOG	protein_coding
Tnnt2	2.98648	7.9254	1.3E-09	4.5E-08	2.5E-05	ENSRNOG	protein_coding
Kif21b	1.82155	3.5346	5.7E-09	1.8E-07	0.00011	ENSRNOG	protein_coding
Kif14	-1.6865	-3.2188	0.00024	0.00206	1	ENSRNOG	protein_coding
Aspm	-1.3687	-2.5824	0.00088	0.0056	1	ENSRNOG	protein_coding
Rgs2	7.98761	253.81	3.1E-69	6.3E-66	6E-65	ENSRNOG	protein_coding
Rgs18	-2.4813	-5.584	0.00956	0.03739	1	ENSRNOG	protein_coding
Ptgs2	-2.1358	-4.3948	9.7E-23	1.4E-20	1.9E-18	ENSRNOG	protein_coding
Prg4	1.9818	3.94986	0.00457	0.02091	1	ENSRNOG	protein_coding
Hmcn1	-3.3702	-10.34	0.00174	0.00975	1	ENSRNOG	protein_coding
Glul	2.74733	6.71474	2.5E-24	3.9E-22	4.8E-20	ENSRNOG	protein_coding
Mr1	4.32913	20.1001	5.7E-12	2.9E-10	1.1E-07	ENSRNOG	protein_coding

Lhx4	2.44502	5.44533	0.00688	0.02903	1	ENSRNOG	protein_coding
Pappa2	2.25729	4.78093	0.00012	0.0012	1	ENSRNOG	protein_coding
Tnn	3.49658	11.2869	7.6E-36	2.2E-33	1.5E-31	ENSRNOG	protein_coding
Tnfsf18	-1.4343	-2.7025	0.00697	0.02935	1	ENSRNOG	protein_coding
Dnm3	2.01194	4.03324	1.4E-05	0.00019	0.26851	ENSRNOG	protein_coding
Mir199a2_	2.14424	4.4206	5E-06	8E-05	0.09686	ENSRNOG	protein_coding
Fmo4	4.63707	24.8827	1.1E-46	5.8E-44	2.1E-42	ENSRNOG	protein_coding
Fmo1	4.16958	17.9957	5.7E-35	1.5E-32	1.1E-30	ENSRNOG	protein_coding
Fmo2	9.25876	612.584	3.4E-69	6.3E-66	6.5E-65	ENSRNOG	protein_coding
Fmo6	6.63332	99.2721	7.8E-22	1E-19	1.5E-17	ENSRNOG	protein_coding
Fmo3	8.11398	277.045	3E-115	6E-111	7E-111	ENSRNOG	protein_coding
Mroh9	8.24182	302.716	0.00041	0.00312	1	ENSRNOG	protein_coding
Selp	-4.8464	-28.768	0.00798	0.0325	1	ENSRNOG	protein_coding
Rn50_13_	-5.8848	-59.088	0.00143	0.00826	1	ENSRNOG	lincRNA
Fam78b	-1.794	-3.4677	2.3E-05	0.00029	0.43911	ENSRNOG	protein_coding
Mgst3	1.92453	3.79613	8.3E-14	5.4E-12	1.6E-09	ENSRNOG	protein_coding
AABR070	-2.9449	-7.7002	0.00028	0.00231	1	ENSRNOG	lincRNA
Nuf2	-1.3643	-2.5745	0.00061	0.0042	1	ENSRNOG	protein_coding
Sh2d1b	-3.0479	-8.2699	1E-10	4.3E-09	2E-06	ENSRNOG	protein_coding
LOC1003	2.2529	4.76642	3.5E-06	5.9E-05	0.06817	ENSRNOG	protein_coding
Olfml2b	1.36807	2.58125	3.8E-07	8.3E-06	0.00727	ENSRNOG	protein_coding
Fcrlb	2.07408	4.21077	0.00271	0.01394	1	ENSRNOG	protein_coding
Adamts4	1.49802	2.82455	0.00409	0.01917	1	ENSRNOG	protein_coding
Tstd1	1.9899	3.9721	0.00245	0.01285	1	ENSRNOG	protein_coding
Ptma	-1.6587	-3.1574	0.00017	0.00153	1	ENSRNOG	protein_coding
Cd244	2.10672	4.30712	0.00112	0.0068	1	ENSRNOG	protein_coding
Atp1a2	5.83342	57.0211	0.01261	0.04633	1	ENSRNOG	protein_coding
Grem2	-3.1696	-8.9978	0.0041	0.01921	1	ENSRNOG	protein_coding
Exo1	-1.6181	-3.0697	2.3E-05	0.00029	0.43598	ENSRNOG	protein_coding
ENSRNO	-3.8291	-14.213	0.00326	0.0161	1	ENSRNOG	protein_coding
Kif26b	6.19324	73.1731	0.00931	0.03658	1	ENSRNOG	protein_coding
LOC1083	1.91471	3.77038	0.00027	0.00223	1	ENSRNOG	lincRNA
Coq8a	3.11853	8.68501	7.5E-09	2.3E-07	0.00014	ENSRNOG	protein_coding
Ephx1	2.07773	4.22143	1.1E-05	0.00016	0.21325	ENSRNOG	protein_coding
Capn8	-2.8979	-7.4534	0.00016	0.00147	1	ENSRNOG	protein_coding
AABR070	-2.9079	-7.5051	0.00128	0.00755	1	ENSRNOG	lincRNA
Dusp10	-1.5961	-3.0233	7.2E-05	0.00076	1	ENSRNOG	protein_coding
Vash2	1.62668	3.08802	0.00012	0.00113	1	ENSRNOG	protein_coding
Nek2	-1.6704	-3.1831	0.0027	0.01394	1	ENSRNOG	protein_coding
Sertad4	-1.9476	-3.8574	1.6E-18	1.6E-16	3E-14	ENSRNOG	protein_coding
Hsd11b1	2.80656	6.99615	9.5E-14	6.2E-12	1.8E-09	ENSRNOG	protein_coding
Lamb3	-3.6951	-12.952	6.7E-29	1.3E-26	1.3E-24	ENSRNOG	protein_coding
Cd34	4.4118	21.2856	0.0034	0.01664	1	ENSRNOG	protein_coding
Csf2ra	1.34279	2.53641	0.00952	0.03729	1	ENSRNOG	protein_coding
Mfsd7	2.15945	4.46745	8.8E-07	1.8E-05	0.01693	ENSRNOG	protein_coding
Gfi1	-3.512	-11.408	4.6E-06	7.5E-05	0.08841	ENSRNOG	protein_coding
LOC6899	2.40628	5.30105	0.01083	0.04097	1	ENSRNOG	protein_coding
Mepe	1.61171	3.05614	0.00639	0.0273	1	ENSRNOG	protein_coding
Ibsp	1.87235	3.6613	0.00562	0.02472	1	ENSRNOG	protein_coding

Dmp1	3.8026	13.9539	2E-13	1.2E-11	3.8E-09	ENSRNOG	protein_coding
Hsd17b11	1.63834	3.11308	4.2E-11	1.8E-09	8.1E-07	ENSRNOG	protein_coding
Hsd17b13	3.09232	8.52868	0.00035	0.00273	1	ENSRNOG	protein_coding
Slc10a6	4.64732	25.06	0.00058	0.00406	1	ENSRNOG	protein_coding
Prdm8	-1.3937	-2.6274	0.00282	0.01443	1	ENSRNOG	protein_coding
Cxcl13	8.52064	367.257	1.3E-22	1.8E-20	2.5E-18	ENSRNOG	protein_coding
AABR070	9.1192	556.1	0.00018	0.00164	1	ENSRNOG	lincRNA
RGD1565	7.09168	136.398	0.00319	0.01586	1	ENSRNOG	protein_coding
LOC5019	6.22473	74.7876	0.01156	0.04322	1	ENSRNOG	protein_coding
Sowahb	-2.941	-7.6794	2.3E-05	0.0003	0.45125	ENSRNOG	protein_coding
Shroom3	3.60445	12.1632	3.4E-11	1.5E-09	6.6E-07	ENSRNOG	protein_coding
Art3	6.73437	106.475	4.1E-19	4.6E-17	7.9E-15	ENSRNOG	protein_coding
Cxcl10	-1.8631	-3.6379	5.3E-06	8.3E-05	0.1013	ENSRNOG	protein_coding
Naaa	1.5779	2.98536	0.01324	0.04815	1	ENSRNOG	protein_coding
LOC1009	-4.1206	-17.395	0.0011	0.00668	1	ENSRNOG	protein_coding
Parm1	1.45642	2.74426	1.7E-10	6.7E-09	3.3E-06	ENSRNOG	protein_coding
Btc	1.4121	2.66125	0.00114	0.0069	1	ENSRNOG	protein_coding
Areg	-2.6164	-6.1324	9.7E-13	5.5E-11	1.9E-08	ENSRNOG	protein_coding
Ereg	-2.0229	-4.0641	2.9E-06	5E-05	0.05629	ENSRNOG	protein_coding
Cxcl1	-2.9782	-7.88	4.2E-23	6E-21	8E-19	ENSRNOG	protein_coding
Cxcl3	-3.875	-14.672	5.1E-12	2.7E-10	9.9E-08	ENSRNOG	protein_coding
Ugt2b10	3.71066	13.0924	0.01288	0.04711	1	ENSRNOG	protein_coding
LOC6898	2.1543	4.45153	5.1E-05	0.00058	0.98578	ENSRNOG	protein_coding
Pdgfra	1.92387	3.79439	9.5E-11	3.9E-09	1.8E-06	ENSRNOG	protein_coding
Spata18	-1.5418	-2.9115	0.00619	0.02667	1	ENSRNOG	protein_coding
Dcun1d4	1.72443	3.30449	9.4E-09	2.8E-07	0.00018	ENSRNOG	protein_coding
Tec	1.57527	2.97992	1E-09	3.6E-08	1.9E-05	ENSRNOG	protein_coding
Txk	3.66247	12.6623	4.6E-17	4.3E-15	8.9E-13	ENSRNOG	protein_coding
Nipal1	2.75633	6.75676	3.1E-09	1E-07	5.9E-05	ENSRNOG	protein_coding
Limch1	-2.2322	-4.6985	0.0101	0.03893	1	ENSRNOG	protein_coding
Smim14	2.04123	4.11595	2.3E-16	1.9E-14	4.3E-12	ENSRNOG	protein_coding
LOC4983	1.5328	2.89346	0.00146	0.0084	1	ENSRNOG	protein_coding
AABR070	2.08889	4.2542	0.00696	0.02928	1	ENSRNOG	lincRNA
AABR070	-1.4988	-2.826	2.8E-05	0.00035	0.53845	ENSRNOG	pseudogene
Pcdh7	-1.5104	-2.8489	0.0081	0.03289	1	ENSRNOG	protein_coding
Slit2	-1.7468	-3.3562	2.3E-08	6.3E-07	0.00043	ENSRNOG	protein_coding
Fam184b	-2.6805	-6.411	3.5E-06	5.9E-05	0.06745	ENSRNOG	protein_coding
Prom1	5.36927	41.3344	8.5E-05	0.00089	1	ENSRNOG	protein_coding
Bst1	1.58534	3.00078	7.9E-11	3.3E-09	1.5E-06	ENSRNOG	protein_coding
C1qtnf7	5.07143	33.6242	0.00032	0.00256	1	ENSRNOG	protein_coding
RGD1561	-2.8857	-7.3908	0.00957	0.03744	1	ENSRNOG	protein_coding
Wdr1	-1.5038	-2.8359	1.2E-08	3.6E-07	0.00023	ENSRNOG	protein_coding
Otop1	-4.0299	-16.335	0.00172	0.00962	1	ENSRNOG	protein_coding
Lyar	-2.1404	-4.4089	7.5E-10	2.7E-08	1.5E-05	ENSRNOG	protein_coding
Msx1	-1.9419	-3.8422	3.9E-08	1E-06	0.00075	ENSRNOG	protein_coding
Wfs1	-1.4314	-2.697	1.1E-09	3.7E-08	2E-05	ENSRNOG	protein_coding
Htra3	1.99205	3.97803	6.4E-05	0.00069	1	ENSRNOG	protein_coding
Acox3	1.40377	2.64592	1E-07	2.5E-06	0.00199	ENSRNOG	protein_coding
Dok7	-1.4496	-2.7312	1.8E-07	4.3E-06	0.00351	ENSRNOG	protein_coding

Msantd1	1.61826	3.07005	0.00313	0.0156	1	ENSRNOG	protein_coding
Zfyve28	1.85356	3.6139	0.00555	0.02446	1	ENSRNOG	protein_coding
Mxd4	2.88891	7.40713	1.5E-22	2.1E-20	2.9E-18	ENSRNOG	protein_coding
Tacc3	-1.5187	-2.8653	0.00022	0.00192	1	ENSRNOG	protein_coding
Pik3ip1	3.95411	15.4991	1.9E-35	5.1E-33	3.6E-31	ENSRNOG	protein_coding
Sec14l2	1.55503	2.9384	8.9E-07	1.8E-05	0.01709	ENSRNOG	protein_coding
Gatsl3	1.51873	2.86538	2.3E-06	4.1E-05	0.04411	ENSRNOG	protein_coding
Pgam2	1.36039	2.56754	0.00873	0.03484	1	ENSRNOG	protein_coding
AC10666:	-2.2348	-4.707	0.01322	0.04807	1	ENSRNOG	pseudogene
LOC1003	1.53719	2.90229	0.01352	0.04889	1	ENSRNOG	protein_coding
Nacad	1.54511	2.91826	4.3E-06	7.1E-05	0.08334	ENSRNOG	protein_coding
Ramp3	-1.3774	-2.598	0.00277	0.01423	1	ENSRNOG	protein_coding
Fignl1	-1.4385	-2.7103	0.00042	0.00315	1	ENSRNOG	protein_coding
Egfr	1.3643	2.57452	3.3E-06	5.6E-05	0.06412	ENSRNOG	protein_coding
Fbxo48	-1.7035	-3.257	0.00419	0.01955	1	ENSRNOG	protein_coding
Lgalsl	2.29675	4.9135	7.7E-15	5.7E-13	1.5E-10	ENSRNOG	protein_coding
AABR070	1.83148	3.55901	0.00127	0.00748	1	ENSRNOG	lincRNA
Acyp2	6.27784	77.5922	0.00825	0.03336	1	ENSRNOG	protein_coding
Psme4	1.36112	2.56884	1.8E-05	0.00024	0.35511	ENSRNOG	protein_coding
Gpr75	-1.7037	-3.2574	3.4E-05	0.00041	0.65726	ENSRNOG	protein_coding
Mss51	1.41099	2.65919	0.00087	0.00555	1	ENSRNOG	protein_coding
Nid2	-2.7145	-6.5638	3.4E-19	3.9E-17	6.5E-15	ENSRNOG	protein_coding
Rarb	2.52276	5.7468	0.00303	0.01526	1	ENSRNOG	protein_coding
LOC3610	-1.558	-2.9445	0.00043	0.00321	1	ENSRNOG	protein_coding
Fam107a	7.15419	142.438	2.7E-74	1.1E-70	5.1E-70	ENSRNOG	protein_coding
LOC1025	1.40577	2.64959	0.00475	0.02151	1	ENSRNOG	protein_coding
Pnp	1.55264	2.93353	1.4E-11	6.8E-10	2.8E-07	ENSRNOG	protein_coding
Rnase4	3.2594	9.57585	3.5E-20	4.2E-18	6.7E-16	ENSRNOG	protein_coding
AC11434:	3.7186	13.1647	0.00059	0.00412	1	ENSRNOG	protein_coding
Ang2	3.34716	10.1764	0.00284	0.01452	1	ENSRNOG	protein_coding
Rnase2	3.69815	12.9794	0.00379	0.01805	1	ENSRNOG	protein_coding
Ndrg2	3.52127	11.4817	3.2E-10	1.2E-08	6.1E-06	ENSRNOG	protein_coding
AABR070	-1.6932	-3.2338	0.0079	0.03225	1	ENSRNOG	protein_coding
Ajuba	-1.7249	-3.3055	4.8E-12	2.5E-10	9.2E-08	ENSRNOG	protein_coding
Carmil3	2.61396	6.1218	0.00073	0.0048	1	ENSRNOG	protein_coding
Tgm1	1.86969	3.65453	0.00396	0.01871	1	ENSRNOG	protein_coding
Nfatc4	1.77986	3.43393	5.1E-07	1.1E-05	0.00987	ENSRNOG	protein_coding
Nynrin	1.99661	3.99061	7E-09	2.2E-07	0.00013	ENSRNOG	protein_coding
Khnyn	1.49085	2.81054	2.4E-06	4.2E-05	0.046	ENSRNOG	protein_coding
Mcpt8l2	-3.5102	-11.394	1.9E-06	3.5E-05	0.03653	ENSRNOG	protein_coding
Mcpt8	-4.3586	-20.516	1.3E-08	3.7E-07	0.00025	ENSRNOG	protein_coding
Gzmc	-2.047	-4.1326	0.00152	0.00873	1	ENSRNOG	protein_coding
LOC6916	-5.6039	-48.635	0.00307	0.0154	1	ENSRNOG	protein_coding
Zmym5	1.55854	2.94555	6.6E-06	0.0001	0.12777	ENSRNOG	protein_coding
Setdb2	1.47746	2.78458	5.3E-07	1.1E-05	0.01021	ENSRNOG	protein_coding
Atp8a2	-1.3274	-2.5096	0.00266	0.01378	1	ENSRNOG	protein_coding
Spata13	1.34582	2.54174	1.7E-09	5.9E-08	3.3E-05	ENSRNOG	protein_coding
Pbk	-1.7064	-3.2635	2.4E-05	0.00031	0.46409	ENSRNOG	protein_coding
Esco2	-1.579	-2.9877	3.9E-05	0.00046	0.75672	ENSRNOG	protein_coding

Ptk2b	-2.2301	-4.6916	1.9E-07	4.5E-06	0.00369	ENSRNOG	protein_coding
Cdca2	-1.7739	-3.4197	5.5E-05	0.00061	1	ENSRNOG	protein_coding
Fam124a	4.09073	17.0385	3.5E-19	4E-17	6.7E-15	ENSRNOG	protein_coding
Wdfy2	1.55714	2.94271	1E-07	2.5E-06	0.00192	ENSRNOG	protein_coding
Kif13b	1.70793	3.26691	8.8E-08	2.2E-06	0.00169	ENSRNOG	protein_coding
Scara5	2.81866	7.05507	1.9E-13	1.2E-11	3.6E-09	ENSRNOG	protein_coding
AABR070	-2.6424	-6.2436	1.1E-05	0.00015	0.20758	ENSRNOG	lincRNA
Adam28	-3.2558	-9.5517	1.3E-06	2.6E-05	0.02589	ENSRNOG	protein_coding
Egr3	1.79648	3.47371	0.00148	0.00851	1	ENSRNOG	protein_coding
RGD1308	1.32855	2.51149	1.9E-06	3.4E-05	0.03574	ENSRNOG	protein_coding
Pdlim2	2.75892	6.76887	6.7E-32	1.4E-29	1.3E-27	ENSRNOG	protein_coding
Dok2	-1.6244	-3.0831	0.0001	0.00104	1	ENSRNOG	protein_coding
Itm2b	1.54725	2.9226	7.7E-07	1.5E-05	0.01483	ENSRNOG	protein_coding
Rubcnl	4.23786	18.8679	0.00229	0.0122	1	ENSRNOG	protein_coding
Lcp1	-2.0135	-4.0375	0.00063	0.00433	1	ENSRNOG	protein_coding
Enox1	2.33935	5.06074	0.00624	0.02684	1	ENSRNOG	protein_coding
Pcdh20	1.38545	2.61254	7.6E-05	0.00081	1	ENSRNOG	protein_coding
Klf5	-1.7537	-3.3721	6.3E-07	1.3E-05	0.01219	ENSRNOG	protein_coding
AABR070	3.93035	15.2459	0.00036	0.00283	1	ENSRNOG	lincRNA
Slc15a1	-3.621	-12.304	0.00143	0.00825	1	ENSRNOG	protein_coding
Clybl	2.81958	7.05959	1.1E-09	3.9E-08	2.1E-05	ENSRNOG	protein_coding
Nalcn	1.59311	3.017	0.0023	0.01223	1	ENSRNOG	protein_coding
Asb14	1.62008	3.07393	0.00993	0.03849	1	ENSRNOG	protein_coding
Il17rd	1.86855	3.65164	0.01172	0.04365	1	ENSRNOG	protein_coding
Duxbl1	4.09844	17.1299	0.00372	0.01782	1	ENSRNOG	protein_coding
Il17rb	2.39515	5.26031	0.01155	0.04318	1	ENSRNOG	protein_coding
Prkcd	1.75949	3.38578	1.9E-06	3.5E-05	0.03695	ENSRNOG	protein_coding
Ogdhl	1.91599	3.77374	3.1E-08	8.3E-07	0.00059	ENSRNOG	protein_coding
Arhgap22	-1.6136	-3.0602	7.4E-10	2.7E-08	1.4E-05	ENSRNOG	protein_coding
Gdf10	6.07473	67.4023	0.01335	0.04845	1	ENSRNOG	protein_coding
Fam25a	-1.7097	-3.2709	0.00048	0.00352	1	ENSRNOG	protein_coding
Sncg	1.99655	3.99044	1.2E-11	5.9E-10	2.4E-07	ENSRNOG	protein_coding
Ldb3	2.23173	4.69695	9.9E-11	4.1E-09	1.9E-06	ENSRNOG	protein_coding
Fam213a	4.0175	16.1953	2.6E-08	7.2E-07	0.0005	ENSRNOG	protein_coding
AABR070	2.15644	4.45814	0.01139	0.04273	1	ENSRNOG	lincRNA
Nwd1	5.41954	42.7999	2.7E-08	7.3E-07	0.00051	ENSRNOG	protein_coding
Cyp4f18	2.50372	5.67147	0.00061	0.00423	1	ENSRNOG	protein_coding
Haus8	-1.4669	-2.7642	6.9E-05	0.00074	1	ENSRNOG	protein_coding
Ushbp1	1.91991	3.78399	0.00421	0.0196	1	ENSRNOG	protein_coding
Ankle1	-1.4437	-2.7202	0.0027	0.01394	1	ENSRNOG	protein_coding
Bst2	1.45992	2.75093	0.00146	0.00844	1	ENSRNOG	protein_coding
Unc13a	2.0941	4.26959	4.5E-09	1.5E-07	8.7E-05	ENSRNOG	protein_coding
Jak3	1.35964	2.5662	0.00017	0.00152	1	ENSRNOG	protein_coding
B3gnt3	6.36413	82.3748	0.00776	0.03182	1	ENSRNOG	protein_coding
Fcho1	2.71063	6.54609	0.00472	0.02138	1	ENSRNOG	protein_coding
Kcnn1	3.88163	14.7396	1.8E-07	4.3E-06	0.00349	ENSRNOG	protein_coding
Arrdc2	2.47367	5.55454	9.9E-15	7.3E-13	1.9E-10	ENSRNOG	protein_coding
Rab3a	1.63795	3.11223	1.2E-05	0.00017	0.23817	ENSRNOG	protein_coding
Pde4c	4.68877	25.7906	6E-10	2.2E-08	1.2E-05	ENSRNOG	protein_coding

LOC1003	-3.3657	-10.308	0.00315	0.01569	1	ENSRNOG	protein_coding
Isyna1	2.86081	7.26423	1.8E-05	0.00024	0.35101	ENSRNOG	protein_coding
Crlf1	2.47568	5.56228	0.00023	0.00194	1	ENSRNOG	protein_coding
Tssk6	-1.7288	-3.3146	0.01321	0.04806	1	ENSRNOG	protein_coding
Pbx4	2.34595	5.08395	0.00879	0.03499	1	ENSRNOG	protein_coding
Lpl	2.35162	5.10396	2.4E-08	6.6E-07	0.00046	ENSRNOG	protein_coding
Ccdc94_1	-2.8637	-7.279	0.00094	0.0059	1	ENSRNOG	protein_coding
Tll1	-1.6429	-3.1229	0.00061	0.0042	1	ENSRNOG	protein_coding
AABR070	1.64663	3.13101	0.00838	0.0338	1	ENSRNOG	lincRNA
Cdkn2aip	1.98021	3.94549	1.3E-10	5.4E-09	2.6E-06	ENSRNOG	protein_coding
Enpp6	3.41318	10.6529	4.7E-07	1E-05	0.00911	ENSRNOG	protein_coding
Cenpu	-1.8018	-3.4867	0.00015	0.00139	1	ENSRNOG	protein_coding
LOC1009	-3.374	-10.367	0.00152	0.00871	1	ENSRNOG	protein_coding
Pdgfrl	3.43608	10.8234	7.9E-68	1.3E-64	1.5E-63	ENSRNOG	protein_coding
AABR070	2.78996	6.9161	0.00028	0.00228	1	ENSRNOG	protein_coding
Slc7a2	1.83581	3.56971	1.2E-15	9.9E-14	2.4E-11	ENSRNOG	protein_coding
Mtmr7	2.09235	4.26441	0.00063	0.00432	1	ENSRNOG	protein_coding
Ppp1r3b	3.24078	9.45304	2.5E-05	0.00031	0.48204	ENSRNOG	protein_coding
Rbpms	2.26619	4.81051	0.00019	0.00168	1	ENSRNOG	protein_coding
Nrg1	-2.2977	-4.9168	1.3E-06	2.5E-05	0.02485	ENSRNOG	protein_coding
Rnf122	2.38981	5.24089	3.5E-10	1.3E-08	6.6E-06	ENSRNOG	protein_coding
Got1l1	2.2744	4.83796	0.00541	0.02392	1	ENSRNOG	protein_coding
Fgfr1	-1.6335	-3.1027	0.00404	0.01901	1	ENSRNOG	protein_coding
Plekha2	2.32945	5.02614	3.4E-26	5.6E-24	6.4E-22	ENSRNOG	protein_coding
Htra4	3.03483	8.19549	6.2E-08	1.6E-06	0.0012	ENSRNOG	protein_coding
Ido2	-6.9659	-125.01	1.3E-24	2E-22	2.4E-20	ENSRNOG	protein_coding
LOC1009	2.20008	4.59504	0.00726	0.03022	1	ENSRNOG	protein_coding
Gins4	-1.4251	-2.6853	0.00013	0.00128	1	ENSRNOG	protein_coding
Ckap2	-1.329	-2.5124	0.00119	0.00712	1	ENSRNOG	protein_coding
Nek3	1.67209	3.18676	0.00204	0.0111	1	ENSRNOG	protein_coding
Myom2	2.78154	6.87586	8E-08	2E-06	0.00155	ENSRNOG	protein_coding
Dlgap2	-4.9677	-31.292	0.00659	0.02801	1	ENSRNOG	protein_coding
Tmco3	4.70039	25.999	1E-22	1.4E-20	1.9E-18	ENSRNOG	protein_coding
Dcun1d2	6.47673	89.0617	0.01292	0.04724	1	ENSRNOG	protein_coding
Gas6	3.09137	8.52305	6.5E-17	5.9E-15	1.2E-12	ENSRNOG	protein_coding
LOC2908	6.42495	85.9216	0.00805	0.03272	1	ENSRNOG	protein_coding
Grtp1_1	1.32256	2.5011	0.0002	0.00175	1	ENSRNOG	protein_coding
AC127756	-2.1479	-4.4319	0.00255	0.01326	1	ENSRNOG	lincRNA
Shcbp1	-1.5516	-2.9313	0.00083	0.00532	1	ENSRNOG	protein_coding
Slc35d2	2.13335	4.38734	1.5E-10	6.1E-09	2.9E-06	ENSRNOG	protein_coding
Gas1	1.52948	2.88683	0.00139	0.0081	1	ENSRNOG	protein_coding
Ntrk2	2.98396	7.91155	0.00016	0.00148	1	ENSRNOG	protein_coding
Lect2	1.97432	3.92942	0.00758	0.03127	1	ENSRNOG	protein_coding
RGD1562	3.36717	10.3186	1.5E-06	2.8E-05	0.02844	ENSRNOG	protein_coding
Prr7	-1.4677	-2.7659	0.00114	0.00689	1	ENSRNOG	protein_coding
Grk6	-1.4637	-2.7581	2.2E-05	0.00028	0.41639	ENSRNOG	protein_coding
Hk3	-2.6312	-6.1954	4.3E-05	0.0005	0.8258	ENSRNOG	protein_coding
Tspan17	1.43047	2.69535	6.1E-08	1.6E-06	0.00117	ENSRNOG	protein_coding
Cdhr2	2.23477	4.70689	0.00262	0.01357	1	ENSRNOG	protein_coding

Nfil3	1.72499	3.30577	2.8E-14	2E-12	5.4E-10	ENSRNOG	protein_coding
Cks2	-1.3625	-2.5714	0.00367	0.01764	1	ENSRNOG	protein_coding
LOC1036	-8.603	-388.84	4.1E-06	6.8E-05	0.07877	ENSRNOG	protein_coding
NEWGEN	2.95376	7.74764	2.4E-32	5.4E-30	4.5E-28	ENSRNOG	protein_coding
LOC1036	9.67788	819.09	0.00013	0.00124	1	ENSRNOG	protein_coding
Aspn	3.80653	13.992	1.8E-08	5.1E-07	0.00034	ENSRNOG	protein_coding
LOC1009	5.16594	35.9007	1.3E-06	2.5E-05	0.02505	ENSRNOG	protein_coding
LOC6793	-1.3537	-2.5557	0.00045	0.00331	1	ENSRNOG	protein_coding
Ogn	2.91562	7.54551	4.3E-49	2.5E-46	8.2E-45	ENSRNOG	protein_coding
Omd	5.79638	55.5757	4.3E-88	2.4E-84	8.2E-84	ENSRNOG	protein_coding
Aspnl1	2.62131	6.15308	2.9E-09	9.6E-08	5.5E-05	ENSRNOG	protein_coding
Ecm2	3.28872	9.77244	2.2E-09	7.4E-08	4.2E-05	ENSRNOG	protein_coding
Fam8a1	1.3337	2.52049	1.1E-05	0.00015	0.20482	ENSRNOG	protein_coding
Cap2	4.64166	24.9619	0.00111	0.00672	1	ENSRNOG	protein_coding
LOC1025	-3.232	-9.396	0.00029	0.00237	1	ENSRNOG	protein_coding
AABR070	1.59746	3.02611	0.00201	0.01093	1	ENSRNOG	pseudogene
Mylip	1.91934	3.7825	3.9E-09	1.3E-07	7.6E-05	ENSRNOG	protein_coding
AABR070	3.25067	9.51806	0.00272	0.01398	1	ENSRNOG	protein_coding
Edn1	2.03491	4.09798	7.1E-08	1.8E-06	0.00136	ENSRNOG	protein_coding
Phactr1	2.44866	5.45907	2.4E-05	0.0003	0.45614	ENSRNOG	protein_coding
Gfod1	-2.1359	-4.395	4.8E-07	1E-05	0.00931	ENSRNOG	protein_coding
Slc35b3	1.59613	3.02332	3.4E-08	9.1E-07	0.00065	ENSRNOG	protein_coding
AABR070	-2.8188	-7.0558	8.4E-06	0.00012	0.16122	ENSRNOG	lincRNA
Nrn1	-1.837	-3.5727	1.3E-16	1.1E-14	2.5E-12	ENSRNOG	protein_coding
F13a1	-5.5831	-47.939	1.2E-20	1.5E-18	2.4E-16	ENSRNOG	protein_coding
AABR070	-1.7034	-3.2567	5.2E-12	2.7E-10	1E-07	ENSRNOG	lincRNA
RGD1307	2.05856	4.1657	0.0008	0.00518	1	ENSRNOG	protein_coding
Fam217a	2.41074	5.31746	0.0004	0.00305	1	ENSRNOG	protein_coding
Slc22a23	-1.6999	-3.2488	1.2E-07	3E-06	0.00237	ENSRNOG	protein_coding
Bphl	1.35594	2.55964	5.7E-05	0.00063	1	ENSRNOG	protein_coding
Serpinb6b	-2.2	-4.5949	0.00616	0.02657	1	ENSRNOG	protein_coding
Exoc2_2	-2.8957	-7.4421	6.9E-05	0.00074	1	ENSRNOG	protein_coding
AABR070	1.63169	3.09875	4.8E-05	0.00054	0.91339	ENSRNOG	pseudogene
RGD1563	-2.8039	-6.9833	0.01262	0.04637	1	ENSRNOG	protein_coding
Cmah	2.26031	4.79096	0.00037	0.00287	1	ENSRNOG	protein_coding
Slc17a4	-3.7759	-13.698	0.00055	0.00391	1	ENSRNOG	protein_coding
Hist1h3a	-2.5995	-6.0608	0.00014	0.00131	1	ENSRNOG	protein_coding
Hist1h2ah	-1.934	-3.8212	0.01311	0.04775	1	ENSRNOG	protein_coding
Hist1h1c	1.53137	2.89059	3.5E-12	1.9E-10	6.8E-08	ENSRNOG	protein_coding
Hfe	1.68781	3.22167	5.2E-06	8.2E-05	0.09929	ENSRNOG	protein_coding
Hist1h2bc	1.39837	2.63603	1.5E-06	2.9E-05	0.02928	ENSRNOG	protein_coding
Hist1h1d	-2.4185	-5.3463	9.1E-05	0.00094	1	ENSRNOG	protein_coding
Btn1a1	2.43789	5.41847	3.2E-05	0.00038	0.60947	ENSRNOG	protein_coding
RGD1562	-1.9289	-3.8076	0.00767	0.0315	1	ENSRNOG	protein_coding
AABR070	2.29135	4.89513	0.00413	0.01934	1	ENSRNOG	lincRNA
Hist1h3b_	-2.6981	-6.4893	0.0016	0.00906	1	ENSRNOG	protein_coding
Zfp192	1.32778	2.51016	4.6E-05	0.00053	0.88102	ENSRNOG	protein_coding
Rn60_17_	1.78969	3.45741	0.00112	0.0068	1	ENSRNOG	unprocessed_pseudogene
ENSRNO	-1.6326	-3.1007	0.00149	0.00858	1	ENSRNOG	protein_coding

Inhba	-1.4039	-2.6461	4.2E-09	1.4E-07	8.1E-05	ENSRNOG	protein_coding
Hecw1	2.85129	7.21644	0.00303	0.01526	1	ENSRNOG	protein_coding
LOC1083	1.57109	2.97129	0.00934	0.03668	1	ENSRNOG	lincRNA
Map3k8	1.66151	3.16347	9.4E-07	1.8E-05	0.01799	ENSRNOG	protein_coding
Fzd8	-1.8965	-3.723	4.3E-07	9.2E-06	0.00817	ENSRNOG	protein_coding
LOC1009	-2.0271	-4.0759	6.8E-08	1.7E-06	0.00131	ENSRNOG	protein_coding
Chrm3	-1.3715	-2.5875	0.01052	0.0401	1	ENSRNOG	protein_coding
Ryr2	2.7916	6.92398	6.1E-05	0.00066	1	ENSRNOG	protein_coding
Akr1c13	2.47146	5.54604	0.00013	0.00121	1	ENSRNOG	protein_coding
Akr1c19	5.17101	36.027	6E-14	3.9E-12	1.1E-09	ENSRNOG	protein_coding
Akr1c12	1.48022	2.78991	2.2E-07	5E-06	0.00422	ENSRNOG	protein_coding
Akr1c3	-3.9676	-15.645	0.00043	0.00321	1	ENSRNOG	protein_coding
Akr1c14	3.47494	11.1189	1.7E-51	1.1E-48	3.2E-47	ENSRNOG	protein_coding
Akr1cl	6.77539	109.546	5E-43	2.3E-40	9.6E-39	ENSRNOG	protein_coding
Net1	1.47207	2.77421	1.9E-05	0.00025	0.3638	ENSRNOG	protein_coding
Calml3	6.7561	108.091	0.0065	0.02769	1	ENSRNOG	protein_coding
Dhtkd1_1	1.38531	2.61228	5.8E-06	9E-05	0.11127	ENSRNOG	protein_coding
Dhtkd1_2	1.98665	3.96317	0.00067	0.00452	1	ENSRNOG	protein_coding
Camk1d	-1.4002	-2.6394	0.00312	0.01559	1	ENSRNOG	protein_coding
Mcm10	-1.5075	-2.8431	7.9E-05	0.00083	1	ENSRNOG	protein_coding
Cdnf	1.67948	3.20312	0.00412	0.0193	1	ENSRNOG	protein_coding
Mt1	2.04233	4.11911	1E-15	8.1E-14	1.9E-11	ENSRNOG	protein_coding
Fam171a	-1.8335	-3.564	4.1E-06	6.8E-05	0.07948	ENSRNOG	protein_coding
Itga8	1.94841	3.85948	1.3E-05	0.00018	0.25628	ENSRNOG	protein_coding
Mrc1	-2.9029	-7.4791	0.00123	0.0073	1	ENSRNOG	protein_coding
Spag6	3.62998	12.3803	4.7E-05	0.00054	0.90378	ENSRNOG	protein_coding
Thnsl1	-2.2125	-4.6347	0.00012	0.00114	1	ENSRNOG	protein_coding
Gpr158	3.16865	8.99204	0.00439	0.02025	1	ENSRNOG	protein_coding
Mastl	-1.4217	-2.679	0.00057	0.00402	1	ENSRNOG	protein_coding
ENSRNO	2.06839	4.19418	1.3E-10	5.2E-09	2.5E-06	ENSRNOG	protein_coding
Nid1	1.52414	2.87615	1.3E-06	2.5E-05	0.02541	ENSRNOG	protein_coding
Greb1l	1.40704	2.65193	1.7E-05	0.00022	0.31775	ENSRNOG	protein_coding
Lama3	-4.047	-16.53	1.8E-43	8.5E-41	3.4E-39	ENSRNOG	protein_coding
Hrh4	4.41755	21.3705	6.1E-05	0.00067	1	ENSRNOG	protein_coding
Cdh2	-2.081	-4.2311	0.0012	0.00716	1	ENSRNOG	protein_coding
AABR070	1.80335	3.4903	0.00446	0.02051	1	ENSRNOG	lincRNA
Rnf125	2.06727	4.19093	1.2E-05	0.00017	0.23249	ENSRNOG	protein_coding
Lims2	2.86939	7.30755	1.5E-18	1.6E-16	2.9E-14	ENSRNOG	protein_coding
Myo7b	1.4699	2.77002	0.00431	0.02	1	ENSRNOG	protein_coding
Gypc	1.63557	3.10709	2.2E-10	8.5E-09	4.2E-06	ENSRNOG	protein_coding
Egr1	1.57252	2.97425	5.1E-06	8.2E-05	0.0989	ENSRNOG	protein_coding
Prob1	1.72121	3.29712	0.01128	0.04233	1	ENSRNOG	protein_coding
Igip	2.44147	5.43195	0.00039	0.003	1	ENSRNOG	protein_coding
Hbegf	-1.7321	-3.3222	4.9E-11	2.1E-09	9.3E-07	ENSRNOG	protein_coding
Apbb3	1.91242	3.7644	1.5E-08	4.2E-07	0.00028	ENSRNOG	protein_coding
AC12524	3.6686	12.7162	1.9E-56	2E-53	3.6E-52	ENSRNOG	lincRNA
Cd14	1.90334	3.74078	1.8E-16	1.5E-14	3.4E-12	ENSRNOG	protein_coding
ENSRNO	2.72524	6.61268	2.3E-06	4.1E-05	0.04422	ENSRNOG	protein_coding
Pcdhb4	2.62194	6.15577	9.8E-05	0.00099	1	ENSRNOG	protein_coding

Pcdhb5	1.88221	3.68641	0.00083	0.00534	1	ENSRNOG	protein_coding
Pcdhb6	4.10454	17.2024	0.00021	0.00182	1	ENSRNOG	pseudogene
Pcdhb7	2.79288	6.93012	0.00125	0.00742	1	ENSRNOG	protein_coding
Pcdhb9	3.9706	15.6772	0.00627	0.02696	1	ENSRNOG	protein_coding
Pcdhb12	1.64331	3.12383	9.7E-05	0.00098	1	ENSRNOG	protein_coding
LOC1083	1.75504	3.37536	0.00032	0.00257	1	ENSRNOG	protein_coding
Pcdhb19	2.88197	7.37154	5.4E-07	1.1E-05	0.01043	ENSRNOG	protein_coding
Pcdhb20	1.98208	3.95062	0.00094	0.00588	1	ENSRNOG	protein_coding
Pcdhga7	1.56502	2.95882	0.00554	0.02441	1	ENSRNOG	protein_coding
Pcdhgb6	1.86624	3.64581	0.00362	0.01744	1	ENSRNOG	protein_coding
Pcdhgb7	3.15237	8.89114	0.00102	0.00629	1	ENSRNOG	protein_coding
Pcdhgb8	1.67057	3.18341	1.9E-05	0.00025	0.37439	ENSRNOG	protein_coding
AABR070	2.87074	7.3144	0.0004	0.00306	1	ENSRNOG	lincRNA
Pcdh12	1.5538	2.9359	0.00893	0.03542	1	ENSRNOG	protein_coding
Lvrn	2.19481	4.5783	0.00044	0.00328	1	ENSRNOG	protein_coding
Sema6a	-2.0442	-4.1245	1.9E-18	1.9E-16	3.6E-14	ENSRNOG	protein_coding
Prdm6	-1.7749	-3.4222	0.00057	0.00402	1	ENSRNOG	protein_coding
Lmnb1	-1.5509	-2.9301	0.00031	0.00247	1	ENSRNOG	protein_coding
LOC1025	-5.0671	-33.523	0.00809	0.03287	1	ENSRNOG	protein_coding
Csf1r	-3.4181	-10.69	1.6E-08	4.6E-07	0.00031	ENSRNOG	protein_coding
Slc26a2	2.83619	7.14134	0.00086	0.00548	1	ENSRNOG	protein_coding
Arhgef37	4.19629	18.3319	4.7E-13	2.8E-11	9E-09	ENSRNOG	protein_coding
Sh3tc2	2.33116	5.03209	1.3E-21	1.7E-19	2.5E-17	ENSRNOG	protein_coding
Piezo2	2.91481	7.54127	1.4E-21	1.8E-19	2.6E-17	ENSRNOG	protein_coding
Ccbe1	-4.1435	-17.673	1.1E-17	1.1E-15	2.1E-13	ENSRNOG	protein_coding
Pmaip1	-2.0893	-4.2555	1.2E-09	4.3E-08	2.3E-05	ENSRNOG	protein_coding
Ska1	-1.5356	-2.899	0.00136	0.00794	1	ENSRNOG	protein_coding
Pstpip2	1.46835	2.76705	0.01252	0.0461	1	ENSRNOG	protein_coding
Mbp	-1.5543	-2.9369	0.00284	0.0145	1	ENSRNOG	protein_coding
AABR070	-1.8225	-3.537	0.0044	0.02029	1	ENSRNOG	pseudogene
Terb1	1.91189	3.76302	0.00656	0.02792	1	ENSRNOG	protein_coding
AABR070	3.88488	14.7729	0.002	0.01092	1	ENSRNOG	protein_coding
Setd6	-1.5633	-2.9554	1.8E-05	0.00024	0.34985	ENSRNOG	protein_coding
Kifc3	-1.3396	-2.5308	3.2E-11	1.4E-09	6.2E-07	ENSRNOG	protein_coding
Cx3cl1	2.36046	5.13533	7.8E-05	0.00082	1	ENSRNOG	protein_coding
Ccl22	-3.4588	-10.995	3.7E-05	0.00044	0.72017	ENSRNOG	protein_coding
Nlrc5	2.13784	4.40102	4.4E-11	1.9E-09	8.4E-07	ENSRNOG	protein_coding
Slc12a3	3.02838	8.15894	0.00572	0.02505	1	ENSRNOG	protein_coding
AC128848	3.55667	11.767	0.00021	0.00184	1	ENSRNOG	protein_coding
Mt2A	3.59668	12.0978	5.2E-06	8.2E-05	0.09914	ENSRNOG	protein_coding
Ces5a	-2.559	-5.8932	6.5E-09	2E-07	0.00013	ENSRNOG	protein_coding
Mcm5	-1.4455	-2.7235	0.00036	0.00282	1	ENSRNOG	protein_coding
Ces1d	8.54597	373.761	4.8E-21	6.1E-19	9.2E-17	ENSRNOG	protein_coding
Ces1f	4.89724	29.8001	5.1E-09	1.6E-07	9.7E-05	ENSRNOG	protein_coding
Il15	1.48425	2.79772	0.00841	0.03386	1	ENSRNOG	protein_coding
Asf1b	-1.4987	-2.8258	0.00087	0.00555	1	ENSRNOG	protein_coding
Zswim4	1.45211	2.73609	6.2E-07	1.3E-05	0.01184	ENSRNOG	protein_coding
AABR070	3.96444	15.6105	0.00403	0.01897	1	ENSRNOG	lincRNA
Orc6	-1.431	-2.6963	5.7E-05	0.00063	1	ENSRNOG	protein_coding

AABR070	-1.5384	-2.9046	0.00903	0.03574	1	ENSRNOG	lincRNA
AABR070	1.36885	2.58264	0.01027	0.03942	1	ENSRNOG	lincRNA
Inpp4b	-5.4118	-42.572	1.9E-07	4.5E-06	0.00373	ENSRNOG	protein_coding
Mmaa	-1.7421	-3.3451	1.9E-11	8.7E-10	3.7E-07	ENSRNOG	protein_coding
Exoc3l1	1.95207	3.86931	6.2E-08	1.6E-06	0.00119	ENSRNOG	protein_coding
Plekhg4	-1.3405	-2.5324	3.5E-09	1.2E-07	6.8E-05	ENSRNOG	protein_coding
Kctd19	-1.3892	-2.6194	4.2E-08	1.1E-06	0.0008	ENSRNOG	protein_coding
Rn60_19_	2.30884	4.95485	0.00029	0.00234	1	ENSRNOG	antisense_RNA
Zdhhc1	1.44061	2.71436	0.00013	0.00122	1	ENSRNOG	protein_coding
Rn60_19_	2.30152	4.92975	2.6E-06	4.5E-05	0.04936	ENSRNOG	antisense_RNA
Esrp2	-1.5576	-2.9436	0.00354	0.01714	1	ENSRNOG	protein_coding
AABR070	1.48605	2.80121	0.00425	0.01979	1	ENSRNOG	lincRNA
Hp	2.08728	4.24946	7.8E-05	0.00082	1	ENSRNOG	protein_coding
Exosc6	-1.4209	-2.6776	0.00047	0.00347	1	ENSRNOG	protein_coding
Fuk	1.50515	2.83854	1E-08	3E-07	0.00019	ENSRNOG	protein_coding
Wdr59	1.62234	3.07873	1.3E-06	2.6E-05	0.02587	ENSRNOG	protein_coding
Ldhd	1.44502	2.72267	2.8E-08	7.5E-07	0.00053	ENSRNOG	protein_coding
Ctrb1	-1.6295	-3.094	0.00488	0.02198	1	ENSRNOG	protein_coding
Pkd1l2	-1.691	-3.2288	0.00043	0.00321	1	ENSRNOG	protein_coding
Osgin1	2.11105	4.32006	5.8E-07	1.2E-05	0.01117	ENSRNOG	protein_coding
Wfdc1	1.80432	3.49264	0.00067	0.00454	1	ENSRNOG	protein_coding
Crispld2	5.10685	34.4599	4.6E-13	2.8E-11	8.9E-09	ENSRNOG	protein_coding
AABR070	-1.9208	-3.7864	0.00878	0.03498	1	ENSRNOG	lincRNA
Fam92b	6.50941	91.1018	0.0056	0.02462	1	ENSRNOG	protein_coding
Sult5a1	2.6272	6.17825	9.8E-08	2.4E-06	0.00188	ENSRNOG	protein_coding
Dpep1	7.4471	174.502	1.2E-57	1.4E-54	2.3E-53	ENSRNOG	protein_coding
Fanca	-1.3329	-2.5192	6.1E-05	0.00067	1	ENSRNOG	protein_coding
Spire2	-1.9555	-3.8784	0.00189	0.01041	1	ENSRNOG	protein_coding
Dbndd1	1.99148	3.97643	0.0042	0.01959	1	ENSRNOG	protein_coding
Acta1	-2.4184	-5.3458	3.1E-06	5.3E-05	0.06007	ENSRNOG	protein_coding
Sipa1l2	1.36437	2.57463	1.3E-06	2.5E-05	0.02488	ENSRNOG	protein_coding
Set_1	-1.5804	-2.9906	5.6E-07	1.2E-05	0.0107	ENSRNOG	protein_coding
AABR070	2.04432	4.12479	5.8E-05	0.00064	1	ENSRNOG	lincRNA
Nrp1	2.67089	6.3682	2.3E-11	1E-09	4.4E-07	ENSRNOG	protein_coding
Glrx	4.91435	30.1555	8.6E-66	1.3E-62	1.7E-61	ENSRNOG	protein_coding
Rhobtb3	1.37032	2.58529	1.4E-07	3.3E-06	0.00261	ENSRNOG	protein_coding
Gpr150	3.60025	12.1278	8.8E-13	5.1E-11	1.7E-08	ENSRNOG	protein_coding
Fam81b	3.4378	10.8363	0.01347	0.04882	1	ENSRNOG	protein_coding
Arrdc3	1.38514	2.61198	1.1E-09	3.8E-08	2.1E-05	ENSRNOG	protein_coding
AC127140	2.98105	7.89561	0.01024	0.03932	1	ENSRNOG	lincRNA
Adgrv1	6.93701	122.532	1.3E-11	6.1E-10	2.5E-07	ENSRNOG	protein_coding
Ckmt2	5.23559	37.6764	1.5E-13	9.5E-12	2.9E-09	ENSRNOG	protein_coding
Serinc5	1.41951	2.67494	1.4E-06	2.7E-05	0.02756	ENSRNOG	protein_coding
Thbs4	2.40789	5.30698	0.00163	0.00923	1	ENSRNOG	protein_coding
Arsb	-1.4855	-2.8002	2.6E-10	9.8E-09	4.9E-06	ENSRNOG	protein_coding
F2rl2	-3.9915	-15.906	1.1E-19	1.3E-17	2.2E-15	ENSRNOG	protein_coding
Arhgef26	1.8687	3.65204	4.2E-15	3.2E-13	8.1E-11	ENSRNOG	protein_coding
Mccc2	1.64335	3.12391	2.8E-07	6.2E-06	0.00532	ENSRNOG	protein_coding
Cenph	-1.4746	-2.779	9.2E-05	0.00094	1	ENSRNOG	protein_coding

AABR070	3.57581	11.9241	0.01082	0.04095	1	ENSRNOG	lincRNA
Mast4	-9.0413	-526.87	4.3E-07	9.2E-06	0.00818	ENSRNOG	protein_coding
Sgtb	1.53945	2.90684	9E-06	0.00013	0.17363	ENSRNOG	protein_coding
Depdc1b	-1.3396	-2.5308	0.00395	0.01868	1	ENSRNOG	protein_coding
Plk2	-3.8584	-14.504	2.2E-52	1.7E-49	4.2E-48	ENSRNOG	protein_coding
Plpp1	1.57091	2.97091	8.8E-17	7.8E-15	1.7E-12	ENSRNOG	protein_coding
Ccno	-2.0526	-4.1485	0.00713	0.02983	1	ENSRNOG	protein_coding
RGD1561	-2.0004	-4.0011	1.1E-05	0.00016	0.21276	ENSRNOG	protein_coding
Itga2	-1.8698	-3.6549	0.00312	0.01559	1	ENSRNOG	protein_coding
Itga1	2.48159	5.58513	5.4E-13	3.2E-11	1E-08	ENSRNOG	protein_coding
Hcn1	-1.3785	-2.6	0.00243	0.01278	1	ENSRNOG	protein_coding
Hmgcs1	1.5979	3.02703	0.00163	0.00922	1	ENSRNOG	protein_coding
Selenop	7.93801	245.234	7.4E-73	2.1E-69	1.4E-68	ENSRNOG	protein_coding
Ccdc152	5.25899	38.2924	3.6E-32	8.2E-30	7E-28	ENSRNOG	protein_coding
Ghr	4.03973	16.4468	9.4E-66	1.3E-62	1.8E-61	ENSRNOG	protein_coding
C6	8.16083	286.19	3.5E-11	1.5E-09	6.6E-07	ENSRNOG	protein_coding
Ptger4	3.39719	10.5355	1.2E-05	0.00016	0.22623	ENSRNOG	protein_coding
LOC1009	-6.8329	-114	0.00054	0.00381	1	ENSRNOG	protein_coding
Lifr	2.42079	5.35464	1.8E-11	8.1E-10	3.4E-07	ENSRNOG	protein_coding
Gdnf	-2.1329	-4.386	1.1E-06	2.1E-05	0.02111	ENSRNOG	protein_coding
Slc1a3	-2.0075	-4.0208	2.4E-08	6.7E-07	0.00047	ENSRNOG	protein_coding
Capsl	2.02832	4.07929	5.7E-05	0.00063	1	ENSRNOG	protein_coding
Spef2	4.47588	22.2523	3.2E-13	1.9E-11	6.1E-09	ENSRNOG	protein_coding
AABR070	4.92914	30.4662	2.5E-08	6.8E-07	0.00048	ENSRNOG	lincRNA
Mtmr12	1.60948	3.05141	5.6E-12	2.9E-10	1.1E-07	ENSRNOG	protein_coding
Cdh18	4.14021	17.633	1.1E-33	2.7E-31	2.1E-29	ENSRNOG	protein_coding
Ankh	-1.3401	-2.5317	2.6E-09	8.6E-08	4.9E-05	ENSRNOG	protein_coding
Fam105a	1.40093	2.64072	1.7E-07	4E-06	0.00327	ENSRNOG	protein_coding
Ctnnd2	-1.469	-2.7683	6.2E-08	1.6E-06	0.00118	ENSRNOG	protein_coding
Cmbl	2.31351	4.9709	7.9E-10	2.9E-08	1.5E-05	ENSRNOG	protein_coding
Car2	-2.2849	-4.8733	5.3E-06	8.4E-05	0.1019	ENSRNOG	protein_coding
Car3	-1.9365	-3.8277	4.1E-15	3.1E-13	7.8E-11	ENSRNOG	protein_coding
Car1	4.92574	30.3945	0.0004	0.00306	1	ENSRNOG	protein_coding
Fabp4	4.25735	19.1244	2.3E-13	1.4E-11	4.4E-09	ENSRNOG	protein_coding
Zfhx4	1.61191	3.05656	3.2E-06	5.4E-05	0.06161	ENSRNOG	protein_coding
Cp	6.03013	65.3505	1.4E-35	3.8E-33	2.6E-31	ENSRNOG	protein_coding
Agtr1b	3.21534	9.28783	0.00054	0.00383	1	ENSRNOG	protein_coding
Ect2	-1.4132	-2.6632	0.00174	0.00975	1	ENSRNOG	protein_coding
Pld1	1.3496	2.54842	8E-13	4.6E-11	1.5E-08	ENSRNOG	protein_coding
Gnb4	-2.3904	-5.243	0.00991	0.03841	1	ENSRNOG	protein_coding
Ccna2	-1.7581	-3.3825	0.00026	0.00219	1	ENSRNOG	protein_coding
Fat4	2.01215	4.03383	1.4E-13	8.8E-12	2.6E-09	ENSRNOG	protein_coding
LOC1083	1.56019	2.94893	0.00173	0.00967	1	ENSRNOG	lincRNA
Hspa4l	-1.5434	-2.9148	0.00015	0.00139	1	ENSRNOG	protein_coding
Plk4	-1.3442	-2.539	0.0005	0.00359	1	ENSRNOG	protein_coding
Abhd18	1.96866	3.91405	5E-07	1E-05	0.00959	ENSRNOG	protein_coding
LOC3657	4.65931	25.2692	0.00056	0.00395	1	ENSRNOG	protein_coding
RGD1565	-1.3408	-2.533	0.00234	0.01242	1	ENSRNOG	lincRNA
AABR070	-2.2002	-4.5954	0.01226	0.04533	1	ENSRNOG	lincRNA

Foxo1	1.33374	2.52054	2.8E-05	0.00035	0.54121	ENSRNOG	protein_coding
Postn	-3.4274	-10.758	5.6E-19	6.2E-17	1.1E-14	ENSRNOG	protein_coding
Rfxapl1	-2.9185	-7.5606	0.01192	0.04428	1	ENSRNOG	protein_coding
Dclk1	3.74349	13.3938	2E-29	3.8E-27	3.9E-25	ENSRNOG	protein_coding
AC12936!	-1.7358	-3.3307	0.00131	0.00771	1	ENSRNOG	pseudogene
Plch1	1.3858	2.61317	2.5E-08	7E-07	0.00049	ENSRNOG	protein_coding
RGD1565	1.34242	2.53576	0.00019	0.0017	1	ENSRNOG	protein_coding
Vom2r44	2.2845	4.87196	4.7E-05	0.00054	0.89739	ENSRNOG	protein_coding
AABR070	-2.522	-5.7438	0.00196	0.01075	1	ENSRNOG	protein_coding
Smc4	-1.4203	-2.6763	6.2E-05	0.00068	1	ENSRNOG	protein_coding
AABR070	1.68895	3.22421	0.00019	0.00169	1	ENSRNOG	lincRNA
Gucy1a3	8.48002	357.06	6.5E-42	2.7E-39	1.2E-37	ENSRNOG	protein_coding
Mnd1	-1.5134	-2.8549	0.00047	0.00343	1	ENSRNOG	protein_coding
Tigd4	1.33726	2.5267	0.0135	0.04887	1	ENSRNOG	protein_coding
Rrnad1	1.38223	2.60672	0.0003	0.00245	1	ENSRNOG	protein_coding
Rn60_2_1	1.62772	3.09024	0.00637	0.02727	1	ENSRNOG	lincRNA
Fdps	1.47254	2.7751	0.00382	0.01817	1	ENSRNOG	protein_coding
Efna3	1.39245	2.62524	0.00942	0.03694	1	ENSRNOG	protein_coding
Efna4	1.8937	3.71586	1E-05	0.00014	0.19149	ENSRNOG	protein_coding
Il6r	2.06301	4.17856	6.2E-17	5.6E-15	1.2E-12	ENSRNOG	protein_coding
Slc27a3	2.06123	4.17343	3.2E-06	5.4E-05	0.06226	ENSRNOG	protein_coding
Npr1	3.10249	8.58898	8.8E-06	0.00013	0.17011	ENSRNOG	protein_coding
S100a1	1.6262	3.08698	0.00035	0.00274	1	ENSRNOG	protein_coding
Sprr1a	-1.9563	-3.8806	0.00022	0.00189	1	ENSRNOG	protein_coding
Crnn	-3.0562	-8.3176	1.1E-05	0.00015	0.20531	ENSRNOG	protein_coding
AABR070	-6.8445	-114.92	0.00014	0.00133	1	ENSRNOG	protein_coding
LOC1036	6.85248	115.558	5.4E-09	1.7E-07	0.0001	ENSRNOG	protein_coding
Sema6c	3.08819	8.50428	6.8E-08	1.7E-06	0.0013	ENSRNOG	protein_coding
AABR070	2.63388	6.20695	4.1E-10	1.5E-08	7.8E-06	ENSRNOG	lincRNA
Ctsk	2.07251	4.20618	0.00042	0.00315	1	ENSRNOG	protein_coding
Adamtsl4	1.85737	3.62346	1.1E-12	6.2E-11	2.1E-08	ENSRNOG	protein_coding
ENSRNO	3.85324	14.4524	8.7E-06	0.00013	0.16777	ENSRNOG	protein_coding
Mtmr11	1.37382	2.59157	9.1E-06	0.00013	0.17529	ENSRNOG	protein_coding
Hfe2	2.45309	5.47588	0.00109	0.00663	1	ENSRNOG	protein_coding
Txnip	3.59841	12.1124	2.3E-14	1.6E-12	4.4E-10	ENSRNOG	protein_coding
Itga10	3.12942	8.75083	2.1E-12	1.1E-10	4E-08	ENSRNOG	protein_coding
Gja5	7.62243	197.052	1.4E-08	4.1E-07	0.00027	ENSRNOG	protein_coding
LOC1009	-1.3345	-2.5218	0.00036	0.00279	1	ENSRNOG	protein_coding
Hmgcs2	3.73451	13.3106	0.00784	0.03206	1	ENSRNOG	protein_coding
Cd2	3.20693	9.23385	2.5E-07	5.7E-06	0.00483	ENSRNOG	protein_coding
Casq2	1.81967	3.53	0.00024	0.00207	1	ENSRNOG	protein_coding
Ngf	-4.3656	-20.615	7.7E-07	1.5E-05	0.01476	ENSRNOG	protein_coding
Nr1h5	2.0428	4.12044	0.00504	0.0226	1	ENSRNOG	protein_coding
Dennd2c	1.53096	2.88978	9.6E-05	0.00097	1	ENSRNOG	protein_coding
Ppm1j	6.0571	66.5838	0.0117	0.04359	1	ENSRNOG	protein_coding
Cd53	-3.1102	-8.6351	7.4E-08	1.9E-06	0.00142	ENSRNOG	protein_coding
Slc16a4	1.986	3.96137	1.2E-17	1.1E-15	2.2E-13	ENSRNOG	protein_coding
Kcnc4	1.76112	3.38962	0.01027	0.03942	1	ENSRNOG	protein_coding
NEWGEN	1.98279	3.95256	7.1E-19	7.7E-17	1.4E-14	ENSRNOG	protein_coding

Gstm7	2.03222	4.09035	6.3E-26	1E-23	1.2E-21	ENSRNOG	protein_coding
Gnat2	-1.9873	-3.9648	5.9E-07	1.2E-05	0.0114	ENSRNOG	protein_coding
Aknad1	2.13057	4.3789	0.00025	0.00211	1	ENSRNOG	protein_coding
Amy1a	6.08912	68.0782	0.01228	0.04539	1	ENSRNOG	protein_coding
Col11a1	2.63346	6.20513	4.1E-19	4.6E-17	7.9E-15	ENSRNOG	protein_coding
S1pr1	3.8743	14.665	0.00131	0.00771	1	ENSRNOG	protein_coding
Vcam1	3.46738	11.0608	2.2E-08	6E-07	0.00041	ENSRNOG	protein_coding
Gpr88	4.51824	22.9153	1.2E-11	5.8E-10	2.3E-07	ENSRNOG	protein_coding
Lrrc39	1.68891	3.22413	0.00043	0.00321	1	ENSRNOG	protein_coding
Agl	1.64949	3.13722	8.9E-07	1.8E-05	0.01707	ENSRNOG	protein_coding
Frrs1	2.01276	4.03555	1.2E-21	1.6E-19	2.3E-17	ENSRNOG	protein_coding
RGD1306	3.21339	9.27531	0.00298	0.01505	1	ENSRNOG	protein_coding
LOC1025	2.63966	6.23183	1.1E-05	0.00016	0.21659	ENSRNOG	lincRNA
Ank2	1.62816	3.09119	3E-12	1.6E-10	5.8E-08	ENSRNOG	protein_coding
Alpk1	2.53342	5.78943	1.4E-16	1.2E-14	2.7E-12	ENSRNOG	protein_coding
Tifa	1.98931	3.97046	2.6E-06	4.5E-05	0.04967	ENSRNOG	protein_coding
LOC6919	1.34366	2.53795	8.2E-07	1.6E-05	0.01578	ENSRNOG	protein_coding
Col25a1	2.26486	4.80608	2E-06	3.6E-05	0.0386	ENSRNOG	protein_coding
AABR070	-1.3481	-2.5458	0.00733	0.03044	1	ENSRNOG	lincRNA
Sgms2	-1.517	-2.862	2.5E-06	4.3E-05	0.04731	ENSRNOG	protein_coding
Manba	1.48912	2.80718	9.3E-05	0.00095	1	ENSRNOG	protein_coding
Slc39a8	1.81814	3.52627	1.9E-17	1.8E-15	3.6E-13	ENSRNOG	protein_coding
Dapp1	1.48679	2.80265	0.00106	0.00651	1	ENSRNOG	protein_coding
Unc5c	-1.7892	-3.4561	5.1E-12	2.7E-10	9.7E-08	ENSRNOG	protein_coding
Gbp5	2.64662	6.26197	0.00207	0.01122	1	ENSRNOG	protein_coding
LOC6850	2.82219	7.07233	6.9E-06	0.0001	0.13169	ENSRNOG	protein_coding
Gbp3	3.62166	12.3091	4.4E-06	7.2E-05	0.08483	ENSRNOG	protein_coding
Kyat3	3.53387	11.5824	5.8E-40	2.2E-37	1.1E-35	ENSRNOG	protein_coding
Clca4	1.67765	3.19906	0.00081	0.00525	1	ENSRNOG	protein_coding
Clca2	3.57593	11.9251	4.6E-07	9.8E-06	0.0089	ENSRNOG	protein_coding
Cyr61	-2.0107	-4.0296	1.4E-15	1.1E-13	2.7E-11	ENSRNOG	protein_coding
Mcoln3	-2.3185	-4.9882	1E-04	0.001	1	ENSRNOG	protein_coding
Ptgfr	1.34284	2.5365	2.4E-07	5.4E-06	0.00458	ENSRNOG	protein_coding
LOC1036	3.23049	9.38587	4.7E-34	1.2E-31	9.1E-30	ENSRNOG	protein_coding
Depdc1	-1.3588	-2.5648	0.0014	0.00815	1	ENSRNOG	protein_coding
LOC1036	6.87357	117.26	0.00376	0.01796	1	ENSRNOG	protein_coding
RT1-M3-1	1.77305	3.41776	1.1E-06	2.2E-05	0.02158	ENSRNOG	protein_coding
Gabbr1	1.62313	3.08044	1.4E-07	3.2E-06	0.0026	ENSRNOG	protein_coding
Rn50_20_	1.65196	3.1426	0.00032	0.00256	1	ENSRNOG	antisense_RNA
Trim40	2.09645	4.27656	0.0026	0.01351	1	ENSRNOG	protein_coding
RT1-N2	1.81801	3.52595	3.2E-07	7.1E-06	0.00615	ENSRNOG	protein_coding
Rn60_20_	2.10635	4.30599	0.00197	0.01079	1	ENSRNOG	antisense_RNA
RT1-S3	2.9589	7.7753	8E-09	2.4E-07	0.00015	ENSRNOG	protein_coding
RT1-T24-	-1.3396	-2.5309	0.00661	0.0281	1	ENSRNOG	protein_coding
RT1-T24-	-2.2027	-4.6035	0.00316	0.01573	1	ENSRNOG	protein_coding
Atat1	1.90073	3.73402	5.1E-05	0.00058	0.987	ENSRNOG	protein_coding
Mdc1	-1.6882	-3.2226	1.9E-08	5.5E-07	0.00037	ENSRNOG	protein_coding
Flot1	1.73216	3.32225	1.2E-12	6.8E-11	2.4E-08	ENSRNOG	protein_coding
Tcf19	-1.5081	-2.8444	4.8E-06	7.7E-05	0.09229	ENSRNOG	protein_coding

Col11a2	1.90062	3.73375	0.00633	0.02716	1	ENSRNOG	protein_coding
LOC1083	1.62653	3.0877	8.9E-06	0.00013	0.1715	ENSRNOG	protein_coding
C4a_2	1.62098	3.07583	0.00321	0.0159	1	ENSRNOG	protein_coding
Atp6v1g2	1.36288	2.57198	0.011	0.04148	1	ENSRNOG	protein_coding
Lsm2	-1.5181	-2.8642	2.7E-05	0.00033	0.51571	ENSRNOG	protein_coding
Kifc1	-1.3412	-2.5337	0.002	0.01091	1	ENSRNOG	protein_coding
Phf1	1.56657	2.96199	3.2E-06	5.4E-05	0.06151	ENSRNOG	protein_coding
Rn60_20_	-1.3572	-2.5619	0.00436	0.02018	1	ENSRNOG	sense_intronic
Pi16	2.14463	4.42179	8.1E-06	0.00012	0.15643	ENSRNOG	protein_coding
Hmga1	-2.1978	-4.5878	2.5E-14	1.7E-12	4.7E-10	ENSRNOG	protein_coding
Fkbp5	3.52267	11.4929	4.4E-45	2.2E-42	8.4E-41	ENSRNOG	protein_coding
Abcg1	-1.5188	-2.8656	0.00168	0.00945	1	ENSRNOG	protein_coding
Sik1	-2.0617	-4.1747	4.1E-09	1.3E-07	7.9E-05	ENSRNOG	protein_coding
Icoslg	-3.4635	-11.031	0.00742	0.03071	1	ENSRNOG	protein_coding
Adarb1	1.82034	3.53163	1.6E-08	4.7E-07	0.00031	ENSRNOG	protein_coding
Pcbp3	-2.7185	-6.582	0.00786	0.0321	1	ENSRNOG	protein_coding
Lss	2.34976	5.09741	2.8E-16	2.4E-14	5.4E-12	ENSRNOG	protein_coding
RGD1564	3.14253	8.83072	0.00016	0.00147	1	ENSRNOG	protein_coding
RGD1563	-2.6983	-6.4903	0.0012	0.00718	1	ENSRNOG	protein_coding
Gstt2	2.50494	5.67626	3.3E-10	1.3E-08	6.4E-06	ENSRNOG	protein_coding
Gstt1	2.26419	4.80385	1.8E-05	0.00023	0.33851	ENSRNOG	protein_coding
AABR070	3.03594	8.20178	0.00014	0.00133	1	ENSRNOG	lincRNA
Ggt1	-3.0663	-8.3763	8E-28	1.5E-25	1.5E-23	ENSRNOG	protein_coding
Rn60_20_	-3.0233	-8.1302	2.3E-12	1.2E-10	4.3E-08	ENSRNOG	lincRNA
Upb1	-4.2015	-18.398	0.00015	0.00137	1	ENSRNOG	protein_coding
Fam13c	1.71784	3.28944	0.00218	0.01169	1	ENSRNOG	protein_coding
Rtkn2	-2.11	-4.3168	4.7E-07	1E-05	0.00907	ENSRNOG	protein_coding
AABR070	-1.7271	-3.3107	0.00036	0.00283	1	ENSRNOG	protein_coding
Fam26e	2.14205	4.41388	1.2E-07	2.9E-06	0.00232	ENSRNOG	protein_coding
Edar	4.95471	31.0111	5.1E-08	1.3E-06	0.00099	ENSRNOG	protein_coding
AABR070	-1.6874	-3.2207	0.00027	0.00227	1	ENSRNOG	pseudogene
Sh3rf3	-1.6973	-3.2429	4.3E-08	1.1E-06	0.00082	ENSRNOG	protein_coding
Ddit4	4.37502	20.7497	2E-35	5.3E-33	3.8E-31	ENSRNOG	protein_coding
Spock2	2.56585	5.92105	0.00101	0.00623	1	ENSRNOG	protein_coding
Chst3	1.99539	3.98723	6.3E-12	3.2E-10	1.2E-07	ENSRNOG	protein_coding
Vsir	3.5333	11.5779	5.8E-19	6.4E-17	1.1E-14	ENSRNOG	protein_coding
Vgll2	-3.461	-11.012	0.00148	0.0085	1	ENSRNOG	protein_coding
Ros1	-3.2524	-9.5293	0.00088	0.00559	1	ENSRNOG	protein_coding
Pln	2.75822	6.76559	5.1E-14	3.4E-12	9.7E-10	ENSRNOG	protein_coding
Fam184a	-1.4988	-2.826	2.6E-08	7.2E-07	0.00051	ENSRNOG	protein_coding
Pkib	3.76104	13.5577	0.00535	0.02369	1	ENSRNOG	protein_coding
Smpdl3a	2.34349	5.0753	2.6E-09	8.8E-08	5.1E-05	ENSRNOG	protein_coding
Tspyl4	1.67561	3.19455	0.00132	0.00773	1	ENSRNOG	protein_coding
Ppil6	1.8552	3.61801	0.00353	0.01712	1	ENSRNOG	protein_coding
Foxo3	2.62529	6.1701	3E-25	4.9E-23	5.7E-21	ENSRNOG	protein_coding
AABR070	3.72493	13.2226	2.6E-07	5.8E-06	0.00497	ENSRNOG	protein_coding
Sesn1	1.59208	3.01485	1.1E-12	6.4E-11	2.2E-08	ENSRNOG	protein_coding
AABR070	-5.3983	-42.175	0.00033	0.00261	1	ENSRNOG	pseudogene
AABR070	2.02848	4.07975	0.00177	0.00986	1	ENSRNOG	lincRNA

Il1rn	3.9606	15.569	1.6E-52	1.3E-49	3E-48	ENSRNOG	protein_coding
Pnpla7	2.10284	4.29555	1.2E-08	3.5E-07	0.00023	ENSRNOG	protein_coding
Nrarp	-1.4945	-2.8177	0.00066	0.00448	1	ENSRNOG	protein_coding
Tubb4b	-1.4769	-2.7835	0.00085	0.00542	1	ENSRNOG	protein_coding
Sapcd2	-2.1964	-4.5834	0.00013	0.00121	1	ENSRNOG	protein_coding
Lcn12	1.85122	3.60806	0.00624	0.02684	1	ENSRNOG	protein_coding
RGD1560	1.58603	3.00222	0.00043	0.00322	1	ENSRNOG	protein_coding
Adamts13	4.06264	16.7101	3.2E-06	5.4E-05	0.06107	ENSRNOG	protein_coding
Dbh	-2.3756	-5.1895	0.00015	0.00139	1	ENSRNOG	protein_coding
Olfm1	-2.8236	-7.0791	0.00867	0.03467	1	ENSRNOG	protein_coding
Cel	2.18973	4.56221	0.01284	0.04699	1	ENSRNOG	protein_coding
AABR070	3.03419	8.19188	0.004	0.01887	1	ENSRNOG	pseudogene
AC142138	5.35724	40.9911	7E-24	1E-21	1.3E-19	ENSRNOG	lincRNA
Lcn2	1.85122	3.60806	0.00061	0.0042	1	ENSRNOG	protein_coding
Ttc16	2.42298	5.36278	2E-07	4.6E-06	0.00387	ENSRNOG	protein_coding
Garnl3	2.50683	5.68368	4.3E-06	7.1E-05	0.08355	ENSRNOG	protein_coding
Phf19	-2.1228	-4.3554	4.7E-05	0.00054	0.90742	ENSRNOG	protein_coding
Rn60_3_0	6.87963	117.754	0.00465	0.02115	1	ENSRNOG	protein_coding
Ttll11	2.84299	7.17507	0.00871	0.0348	1	ENSRNOG	protein_coding
Lhx6	-2.3958	-5.2627	0.00215	0.01156	1	ENSRNOG	protein_coding
Crb2	1.86119	3.63307	0.00774	0.03177	1	ENSRNOG	protein_coding
Olfml2a	3.59078	12.0485	8.8E-12	4.4E-10	1.7E-07	ENSRNOG	protein_coding
Cacnb4	4.04529	16.5102	1.2E-06	2.3E-05	0.02333	ENSRNOG	protein_coding
Galnt5	3.44124	10.8622	2.8E-08	7.6E-07	0.00054	ENSRNOG	protein_coding
Ly75	1.52483	2.87753	0.00069	0.00464	1	ENSRNOG	protein_coding
Scn1a	2.21307	4.63661	2.1E-05	0.00027	0.39562	ENSRNOG	protein_coding
Nostrin	1.60461	3.04115	0.00527	0.02344	1	ENSRNOG	protein_coding
Spc25	-1.3693	-2.5835	0.00016	0.00148	1	ENSRNOG	protein_coding
Dhrs9	-1.327	-2.5088	2.9E-09	9.5E-08	5.5E-05	ENSRNOG	protein_coding
Klhl41	-1.7183	-3.2906	4.2E-06	6.9E-05	0.0808	ENSRNOG	protein_coding
Itga6	-3.0443	-8.2495	1.1E-09	4E-08	2.2E-05	ENSRNOG	protein_coding
Rapgef4	1.74415	3.34998	3.1E-17	2.8E-15	5.9E-13	ENSRNOG	protein_coding
Cdca7	-1.3703	-2.5852	0.00032	0.00254	1	ENSRNOG	protein_coding
Hoxd3	1.67129	3.18499	1E-05	0.00015	0.19793	ENSRNOG	protein_coding
Fkbp7	1.50761	2.84339	3.9E-08	1E-06	0.00075	ENSRNOG	protein_coding
Ccdc141	2.79925	6.96077	0.00038	0.00291	1	ENSRNOG	protein_coding
Frzb	5.00162	32.036	0.00084	0.00539	1	ENSRNOG	protein_coding
Nup35	-1.3373	-2.5267	3.1E-05	0.00038	0.59412	ENSRNOG	protein_coding
Calcrl	1.83286	3.56243	4.6E-09	1.5E-07	8.9E-05	ENSRNOG	protein_coding
Serping1	4.3787	20.8028	2.6E-05	0.00032	0.49469	ENSRNOG	protein_coding
Slc43a1	1.36952	2.58385	5.9E-05	0.00065	1	ENSRNOG	protein_coding
Rtn4rl2	1.68161	3.20785	0.01132	0.04249	1	ENSRNOG	protein_coding
Harbi1	1.45718	2.74572	3.1E-05	0.00038	0.6	ENSRNOG	protein_coding
RGD1564	1.89181	3.711	2.1E-05	0.00027	0.40794	ENSRNOG	protein_coding
Prr5l	-1.7198	-3.294	5.6E-05	0.00062	1	ENSRNOG	protein_coding
Fjx1	-2.1762	-4.5195	0.00042	0.00319	1	ENSRNOG	protein_coding
Slc1a2	2.75866	6.76766	0.00035	0.00273	1	ENSRNOG	protein_coding
Ehf	2.83248	7.12296	4.8E-07	1E-05	0.00918	ENSRNOG	protein_coding
LOC6910	3.14299	8.83352	1.8E-11	8.2E-10	3.4E-07	ENSRNOG	protein_coding

Pax6	2.88882	7.40665	0.00012	0.00118	1	ENSRNOG	protein_coding
Kif18a	-1.5129	-2.8539	0.00025	0.00208	1	ENSRNOG	protein_coding
Ccdc34	-1.5097	-2.8474	6.5E-05	0.0007	1	ENSRNOG	protein_coding
Bbox1	3.46562	11.0472	0.00019	0.00169	1	ENSRNOG	protein_coding
Fibin	3.7937	13.8681	9.6E-36	2.8E-33	1.8E-31	ENSRNOG	protein_coding
Grem1	-1.8949	-3.719	1.4E-17	1.4E-15	2.7E-13	ENSRNOG	protein_coding
Arhgap11	-1.702	-3.2535	0.00019	0.00167	1	ENSRNOG	protein_coding
Rasgrp1	-3.5846	-11.997	3.8E-09	1.2E-07	7.3E-05	ENSRNOG	protein_coding
Fsip1	-1.3804	-2.6035	0.00723	0.03015	1	ENSRNOG	protein_coding
Bmf	3.15716	8.92069	1.5E-13	9.6E-12	2.9E-09	ENSRNOG	protein_coding
Chac1	-1.6865	-3.2187	0.00149	0.00856	1	ENSRNOG	protein_coding
Oip5	-1.4716	-2.7734	0.00078	0.0051	1	ENSRNOG	protein_coding
Nusap1	-1.4163	-2.669	0.00065	0.00446	1	ENSRNOG	protein_coding
Itpka	2.05923	4.16764	0.00338	0.01654	1	ENSRNOG	protein_coding
Capn3	2.73544	6.65961	5.5E-07	1.2E-05	0.01064	ENSRNOG	protein_coding
LOC1009	-1.4624	-2.7557	0.00022	0.00188	1	ENSRNOG	protein_coding
LOC1009	7.64205	199.749	0.00192	0.01053	1	ENSRNOG	protein_coding
Ccndbp1	1.49131	2.81144	7.7E-08	1.9E-06	0.00148	ENSRNOG	protein_coding
Epb42	3.4465	10.9018	1.6E-10	6.3E-09	3E-06	ENSRNOG	protein_coding
Tgm7l1_1	4.29575	19.6404	2.6E-37	7.8E-35	4.9E-33	ENSRNOG	protein_coding
Tgm7l1_2	3.071	8.40355	0.00032	0.00254	1	ENSRNOG	protein_coding
Ckmt1	3.11639	8.67212	1.1E-05	0.00016	0.21999	ENSRNOG	protein_coding
Frmd5	-1.3902	-2.6211	0.0005	0.00361	1	ENSRNOG	protein_coding
AC112350	2.64989	6.27618	0.00956	0.0374	1	ENSRNOG	pseudogene
Slc28a2	2.18762	4.55552	8.6E-14	5.6E-12	1.7E-09	ENSRNOG	protein_coding
MGC1056	-1.6627	-3.1662	4E-05	0.00046	0.76062	ENSRNOG	protein_coding
Usp50	2.34105	5.06672	0.00357	0.01728	1	ENSRNOG	protein_coding
Ncaph	-1.5591	-2.9466	0.00032	0.00254	1	ENSRNOG	protein_coding
Adra2b	3.55186	11.7278	0.0016	0.00909	1	ENSRNOG	protein_coding
Fahd2a	1.53287	2.89362	7.9E-09	2.4E-07	0.00015	ENSRNOG	protein_coding
Prom2	-3.0268	-8.1501	6.4E-06	9.9E-05	0.12333	ENSRNOG	protein_coding
Acoxl	6.616	98.0877	0.00512	0.02285	1	ENSRNOG	protein_coding
Bcl2l11	3.05416	8.30604	1.4E-11	6.7E-10	2.7E-07	ENSRNOG	protein_coding
Mertk	3.64715	12.5286	3E-06	5.2E-05	0.05854	ENSRNOG	protein_coding
Ckap2l	-1.6003	-3.0321	0.00038	0.00295	1	ENSRNOG	protein_coding
Il1a	-3.3967	-10.532	0.00122	0.00727	1	ENSRNOG	protein_coding
Nop56	-1.4382	-2.7099	1.2E-06	2.3E-05	0.02262	ENSRNOG	protein_coding
Cpxm1	1.97331	3.92669	0.00329	0.01623	1	ENSRNOG	protein_coding
Slc4a11	-2.9196	-7.5665	1.1E-13	7.3E-12	2.2E-09	ENSRNOG	protein_coding
Rassf2	4.1397	17.6269	6E-43	2.7E-40	1.2E-38	ENSRNOG	protein_coding
Pcna	-1.3689	-2.5828	0.00019	0.00168	1	ENSRNOG	protein_coding
Plcb4	1.98782	3.96636	5.8E-12	3E-10	1.1E-07	ENSRNOG	protein_coding
Sptlc3	1.52775	2.88336	0.00065	0.00446	1	ENSRNOG	protein_coding
Nxt1	-1.3677	-2.5806	0.00014	0.00128	1	ENSRNOG	protein_coding
Cst3	3.35421	10.2263	1.2E-12	6.8E-11	2.3E-08	ENSRNOG	protein_coding
Dusp15	2.24267	4.73272	0.00077	0.00504	1	ENSRNOG	protein_coding
Hck	1.91469	3.77032	0.00049	0.00356	1	ENSRNOG	protein_coding
Nol4l	2.44328	5.43879	3.1E-05	0.00038	0.59524	ENSRNOG	protein_coding
Tp53inp2	1.83136	3.55872	5.2E-20	6.1E-18	1E-15	ENSRNOG	protein_coding

Acss2	2.02294	4.0641	4.6E-16	3.8E-14	8.8E-12	ENSRNOG	protein_coding
Myh7b	1.98364	3.95489	0.00012	0.00114	1	ENSRNOG	protein_coding
Rbm12_2	-1.3387	-2.5293	0.0015	0.00861	1	ENSRNOG	protein_coding
Dsn1	-1.5239	-2.8757	1.5E-05	0.00021	0.29516	ENSRNOG	protein_coding
Rbl1	-1.5535	-2.9352	8.6E-06	0.00013	0.1647	ENSRNOG	protein_coding
Adig	4.9352	30.5945	1.8E-08	5E-07	0.00034	ENSRNOG	protein_coding
Lbp	3.29547	9.81825	1.3E-12	7E-11	2.4E-08	ENSRNOG	protein_coding
LOC1009	4.83268	28.4958	9.8E-09	2.9E-07	0.00019	ENSRNOG	protein_coding
Fam83d	-1.8937	-3.7158	8.8E-05	0.00091	1	ENSRNOG	protein_coding
Lpin3	1.59815	3.02754	4.4E-08	1.2E-06	0.00085	ENSRNOG	protein_coding
Sgk2	-3.1377	-8.8013	1.8E-05	0.00024	0.35504	ENSRNOG	protein_coding
Mybl2	-2.1938	-4.575	9.2E-05	0.00095	1	ENSRNOG	protein_coding
R3hdml	-6.0575	-66.601	0.00097	0.00604	1	ENSRNOG	protein_coding
Rbpjl	-3.4599	-11.004	0.00064	0.00441	1	ENSRNOG	protein_coding
Sdc4	1.62175	3.07749	2E-11	9.1E-10	3.9E-07	ENSRNOG	protein_coding
Ube2c	-1.9584	-3.8863	0.0002	0.00178	1	ENSRNOG	protein_coding
Mmp9	-4.7073	-26.124	2.4E-12	1.3E-10	4.5E-08	ENSRNOG	protein_coding
Slc12a5	1.73685	3.33308	0.00935	0.03673	1	ENSRNOG	protein_coding
Sulf2	3.54315	11.6572	1.3E-12	7.1E-11	2.5E-08	ENSRNOG	protein_coding
Cebpb	2.1575	4.4614	2.3E-18	2.3E-16	4.4E-14	ENSRNOG	protein_coding
Kcng1	-2.43	-5.389	1E-05	0.00014	0.19266	ENSRNOG	protein_coding
Cyp24a1	-2.3077	-4.9509	0.00072	0.00476	1	ENSRNOG	protein_coding
Aurka	-1.4334	-2.7008	0.00108	0.0066	1	ENSRNOG	protein_coding
Bmp7	-4.9749	-31.449	4.1E-10	1.5E-08	7.8E-06	ENSRNOG	protein_coding
Rbm38	-1.5396	-2.9072	1.3E-06	2.4E-05	0.02412	ENSRNOG	protein_coding
RGD1561	-1.3988	-2.6368	0.00015	0.00137	1	ENSRNOG	protein_coding
Pmepa1	-2.4737	-5.5548	4.5E-11	1.9E-09	8.7E-07	ENSRNOG	protein_coding
AABR070	2.89757	7.45171	0.00907	0.03584	1	ENSRNOG	protein_coding
Rbbp8nl	2.63859	6.22721	0.0085	0.03411	1	ENSRNOG	protein_coding
Slco4a1	-2.7758	-6.8485	0.0002	0.00173	1	ENSRNOG	protein_coding
Col20a1	2.43865	5.42135	0.00326	0.01611	1	ENSRNOG	protein_coding
Pcmtd2	2.08193	4.23372	5.8E-09	1.8E-07	0.00011	ENSRNOG	protein_coding
Insig1	2.40404	5.29285	4.8E-05	0.00055	0.92186	ENSRNOG	protein_coding
Xrcc2	-1.5745	-2.9783	1E-04	0.001	1	ENSRNOG	protein_coding
Dnajc2	-1.3491	-2.5476	2.5E-06	4.3E-05	0.0471	ENSRNOG	protein_coding
Fbxl13	3.66267	12.6641	0.00867	0.03465	1	ENSRNOG	protein_coding
Ccdc146	2.90133	7.47112	2E-06	3.6E-05	0.0388	ENSRNOG	protein_coding
Fgl2	-1.669	-3.1799	0.00566	0.02485	1	ENSRNOG	protein_coding
Gnai1	-1.637	-3.1103	1.4E-08	4.1E-07	0.00027	ENSRNOG	protein_coding
Cd36	2.2663	4.81087	0.00127	0.00749	1	ENSRNOG	protein_coding
Sema3d	1.45405	2.73976	1.3E-06	2.6E-05	0.02594	ENSRNOG	protein_coding
Steap4	2.53671	5.80266	0.00015	0.00143	1	ENSRNOG	protein_coding
Steap2	1.67434	3.19174	2.1E-12	1.2E-10	4.1E-08	ENSRNOG	protein_coding
Cfap69	2.12402	4.35908	6.9E-07	1.4E-05	0.01324	ENSRNOG	protein_coding
Ppp1r9a	-4.6084	-24.393	5.8E-12	3E-10	1.1E-07	ENSRNOG	protein_coding
Pdk4	2.22739	4.68287	3.1E-10	1.2E-08	6E-06	ENSRNOG	protein_coding
ENSRNO	-1.606	-3.0441	4.3E-14	2.9E-12	8.2E-10	ENSRNOG	protein_coding
Dlx6as	1.76215	3.39203	0.00052	0.00375	1	ENSRNOG	antisense_RNA
Nxph1	2.30943	4.95688	2.1E-14	1.5E-12	4.1E-10	ENSRNOG	protein_coding

Cftr	-1.7284	-3.3135	0.00639	0.0273	1	ENSRNOG	protein_coding
Cped1	2.70617	6.52587	1.3E-16	1.2E-14	2.6E-12	ENSRNOG	protein_coding
Iqub	1.89586	3.72145	0.00075	0.00494	1	ENSRNOG	protein_coding
Gpr37	1.44267	2.71824	0.00152	0.0087	1	ENSRNOG	protein_coding
N5	3.07795	8.44412	2.8E-07	6.4E-06	0.00548	ENSRNOG	pseudogene
Agbl3	2.26482	4.80595	4E-10	1.5E-08	7.7E-06	ENSRNOG	protein_coding
Tmem140	3.02125	8.11868	4.7E-30	9.2E-28	9E-26	ENSRNOG	protein_coding
RGD1565	1.95169	3.86827	0.00149	0.00856	1	ENSRNOG	protein_coding
Slc13a4	4.15837	17.8565	1.4E-13	8.8E-12	2.6E-09	ENSRNOG	protein_coding
Fam180a	6.80977	112.188	1E-108	8E-105	2E-104	ENSRNOG	protein_coding
Ptn	2.72045	6.5908	2.9E-10	1.1E-08	5.5E-06	ENSRNOG	protein_coding
Hipk2	1.47624	2.78223	2.6E-11	1.1E-09	5E-07	ENSRNOG	protein_coding
Kdm7a	1.7561	3.37785	2E-06	3.6E-05	0.03804	ENSRNOG	protein_coding
AABR070	1.90723	3.75088	0.00036	0.00278	1	ENSRNOG	lincRNA
Dennd2a	1.49151	2.81183	1E-07	2.5E-06	0.00194	ENSRNOG	protein_coding
Tcaf2	5.06156	33.3951	8.7E-52	6.1E-49	1.7E-47	ENSRNOG	protein_coding
Rn50_4_1	7.3009	157.684	0.00226	0.01206	1	ENSRNOG	unprocessed_pseudogene
Fam131b	2.76639	6.80402	3.5E-10	1.3E-08	6.8E-06	ENSRNOG	protein_coding
Epha1	3.70758	13.0645	8.4E-11	3.5E-09	1.6E-06	ENSRNOG	protein_coding
Fam115c	5.03329	32.747	7.5E-51	4.8E-48	1.4E-46	ENSRNOG	protein_coding
Rn50_4_1	5.07911	33.8037	2.8E-27	4.9E-25	5.4E-23	ENSRNOG	unprocessed_pseudogene
AABR070	1.68585	3.2173	2E-07	4.7E-06	0.0039	ENSRNOG	lincRNA
Tmem176	3.07593	8.4323	1.1E-05	0.00015	0.20449	ENSRNOG	protein_coding
Tmem176	2.73525	6.65876	0.00184	0.01018	1	ENSRNOG	protein_coding
Creb5	-2.7192	-6.585	0.00072	0.00477	1	ENSRNOG	protein_coding
Nod1	1.43739	2.7083	1.3E-09	4.5E-08	2.5E-05	ENSRNOG	protein_coding
Neurod6	-1.6587	-3.1573	0.00026	0.00215	1	ENSRNOG	protein_coding
Herc6	1.75643	3.37861	2.7E-07	6.1E-06	0.00521	ENSRNOG	protein_coding
Nap1l5	1.9577	3.88442	0.00148	0.00851	1	ENSRNOG	protein_coding
Fam13a	4.9598	31.1207	6.9E-40	2.5E-37	1.3E-35	ENSRNOG	protein_coding
Mad2l1	-1.4791	-2.7877	0.00019	0.00167	1	ENSRNOG	protein_coding
AABR070	1.76821	3.40632	2E-05	0.00026	0.39134	ENSRNOG	lincRNA
Thnsl2	2.00889	4.02472	0.0002	0.00176	1	ENSRNOG	protein_coding
Tmem150	2.72208	6.59822	1.9E-11	8.7E-10	3.7E-07	ENSRNOG	protein_coding
Rnf181	1.5724	2.974	2.9E-09	9.5E-08	5.5E-05	ENSRNOG	protein_coding
Dnah6	-1.5083	-2.8447	0.00487	0.02195	1	ENSRNOG	protein_coding
AABR070	-1.8691	-3.653	0.01247	0.04596	1	ENSRNOG	lincRNA
Loxl3	2.47817	5.5719	4E-21	5.2E-19	7.7E-17	ENSRNOG	protein_coding
Dqx1	3.25741	9.56267	1.2E-09	4.1E-08	2.3E-05	ENSRNOG	protein_coding
LOC1036	2.6447	6.25366	3.5E-05	0.00041	0.66417	ENSRNOG	protein_coding
LOC1036	2.81929	7.05813	8.9E-06	0.00013	0.17181	ENSRNOG	protein_coding
LOC1036	2.2483	4.75124	0.002	0.01092	1	ENSRNOG	protein_coding
LOC5002	1.50889	2.84591	0.00269	0.01387	1	ENSRNOG	protein_coding
Actg2	-4.2767	-19.382	4.7E-13	2.8E-11	9E-09	ENSRNOG	protein_coding
Cd207	-4.7726	-27.334	4.3E-10	1.6E-08	8.3E-06	ENSRNOG	protein_coding
Cyp26b1	2.66753	6.35339	4.5E-06	7.3E-05	0.08639	ENSRNOG	protein_coding
RGD1560	-1.4359	-2.7056	3E-05	0.00036	0.57032	ENSRNOG	protein_coding
Fbxo41	4.81525	28.1537	3E-05	0.00037	0.58137	ENSRNOG	protein_coding
Nat8f5	4.07008	16.7964	0.00344	0.01678	1	ENSRNOG	protein_coding

LOC1036	7.07012	134.374	0.00351	0.01703	1	ENSRNOG	protein_coding
Antxr1	1.54747	2.92304	5.4E-05	0.00061	1	ENSRNOG	protein_coding
Abtb1	2.67407	6.38228	2.4E-14	1.7E-12	4.6E-10	ENSRNOG	protein_coding
Cfap100	2.60026	6.06395	1.1E-30	2.3E-28	2.2E-26	ENSRNOG	protein_coding
Klf15	3.84927	14.4127	3.3E-57	3.7E-54	6.4E-53	ENSRNOG	protein_coding
Nup210	-2.8305	-7.1131	4.5E-07	9.7E-06	0.00869	ENSRNOG	protein_coding
Fbln2	-2.382	-5.2125	2.8E-05	0.00034	0.53535	ENSRNOG	protein_coding
Aldh1l1	3.08685	8.49639	2.6E-07	5.9E-06	0.00505	ENSRNOG	protein_coding
Adamts9	1.82228	3.53639	1.7E-13	1.1E-11	3.2E-09	ENSRNOG	protein_coding
Lrig1	-2.0454	-4.1279	3.6E-07	7.8E-06	0.00683	ENSRNOG	protein_coding
Mitf	1.3751	2.59385	1.9E-06	3.5E-05	0.03654	ENSRNOG	protein_coding
Gxylt2	1.43262	2.69936	0.00797	0.03248	1	ENSRNOG	protein_coding
Lmcd1	2.60023	6.06384	3.2E-12	1.7E-10	6.2E-08	ENSRNOG	protein_coding
Camk1	1.80993	3.50625	6.3E-15	4.8E-13	1.2E-10	ENSRNOG	protein_coding
Ttll3	1.50744	2.84304	0.00531	0.02358	1	ENSRNOG	protein_coding
Il17re	2.43076	5.39178	6.1E-32	1.3E-29	1.2E-27	ENSRNOG	protein_coding
Ghrl	2.19971	4.59388	0.00497	0.02233	1	ENSRNOG	protein_coding
Hrh1	-5.5371	-46.432	0.00239	0.01262	1	ENSRNOG	protein_coding
Syn2	9.02279	520.153	0.00012	0.00116	1	ENSRNOG	protein_coding
Timp4	8.38168	333.532	2E-09	7E-08	3.9E-05	ENSRNOG	protein_coding
Pparg	1.4417	2.7164	7.5E-08	1.9E-06	0.00145	ENSRNOG	protein_coding
Mbd4	1.55738	2.9432	1E-05	0.00014	0.19177	ENSRNOG	protein_coding
Alox5	6.45533	87.7501	0.00649	0.02768	1	ENSRNOG	protein_coding
LOC5003	3.12886	8.74746	2.1E-26	3.5E-24	4E-22	ENSRNOG	protein_coding
A2m	6.46443	88.3056	3.7E-38	1.3E-35	7.1E-34	ENSRNOG	protein_coding
LOC1009	4.99359	31.8581	2.1E-32	5E-30	4.1E-28	ENSRNOG	protein_coding
M6pr	-1.6026	-3.0368	0.01222	0.04526	1	ENSRNOG	protein_coding
Mfap5	2.73515	6.65826	1.1E-05	0.00015	0.20452	ENSRNOG	protein_coding
C1rl	1.81095	3.50873	1.1E-11	5.3E-10	2.1E-07	ENSRNOG	protein_coding
C1r	1.47752	2.7847	5.7E-08	1.5E-06	0.0011	ENSRNOG	protein_coding
C1s	2.02388	4.06677	3.7E-08	9.8E-07	0.0007	ENSRNOG	protein_coding
Cdca3	-1.6668	-3.1752	0.00094	0.00586	1	ENSRNOG	protein_coding
Acrbp	2.197	4.58526	0.00016	0.00148	1	ENSRNOG	protein_coding
Iffo1	1.98775	3.96619	4.8E-07	1E-05	0.00926	ENSRNOG	protein_coding
Scnn1a	2.95629	7.76126	7.3E-08	1.8E-06	0.0014	ENSRNOG	protein_coding
Vwf	2.9007	7.46787	8.2E-05	0.00086	1	ENSRNOG	protein_coding
Ano2	-3.5836	-11.989	1.5E-31	3.1E-29	2.8E-27	ENSRNOG	protein_coding
Akap3	2.34858	5.09321	0.00117	0.00704	1	ENSRNOG	protein_coding
Rad51ap1	-1.5082	-2.8446	0.00011	0.00109	1	ENSRNOG	protein_coding
LOC6895	2.0855	4.24421	0.00367	0.01764	1	ENSRNOG	lincRNA
Tead4	1.35377	2.55579	1.7E-09	5.8E-08	3.3E-05	ENSRNOG	protein_coding
Foxm1	-1.5182	-2.8644	0.00091	0.00576	1	ENSRNOG	protein_coding
Fkbp4	-1.3989	-2.637	1.9E-06	3.5E-05	0.03635	ENSRNOG	protein_coding
Clec2d2	1.56978	2.9686	3.2E-07	7.1E-06	0.00619	ENSRNOG	protein_coding
Clec2d_3	3.03389	8.19018	7.4E-13	4.3E-11	1.4E-08	ENSRNOG	protein_coding
LOC1003	3.49104	11.2436	7.9E-06	0.00012	0.1518	ENSRNOG	protein_coding
Clec2e	2.47205	5.54832	0.00059	0.00411	1	ENSRNOG	protein_coding
Clec2g	1.33253	2.51844	1.4E-05	0.00019	0.27369	ENSRNOG	protein_coding
Clec12a	4.00982	16.1093	3.9E-07	8.5E-06	0.00749	ENSRNOG	protein_coding

Gabarapl1	1.92406	3.7949	6.2E-11	2.6E-09	1.2E-06	ENSRNOG	protein_coding
LOC6900	6.19792	73.4107	0.0082	0.0332	1	ENSRNOG	protein_coding
Gpr19	-1.6263	-3.0872	0.00036	0.00279	1	ENSRNOG	protein_coding
Gprc5d	-2.8224	-7.0732	0.00426	0.01982	1	ENSRNOG	protein_coding
Mgp	2.13293	4.38606	0.0001	0.00101	1	ENSRNOG	protein_coding
LOC1009	7.31845	159.614	0.00471	0.02138	1	ENSRNOG	protein_coding
Abcc9	-2.2721	-4.8302	5.7E-21	7.1E-19	1.1E-16	ENSRNOG	protein_coding
Etnk1	1.44357	2.71993	4.2E-07	9.1E-06	0.00814	ENSRNOG	protein_coding
Sox5	2.80766	7.00148	7.2E-05	0.00076	1	ENSRNOG	protein_coding
Casc1	2.3598	5.13299	2.1E-05	0.00027	0.41061	ENSRNOG	protein_coding
Sspn	3.44492	10.8899	0.00702	0.02948	1	ENSRNOG	protein_coding
AABR070	2.2401	4.7243	2E-06	3.6E-05	0.0381	ENSRNOG	protein_coding
Itpr2	2.21082	4.62937	3.3E-12	1.8E-10	6.3E-08	ENSRNOG	protein_coding
LOC6907	2.47695	5.56719	0.01152	0.0431	1	ENSRNOG	protein_coding
Tmtc1	4.96006	31.1263	4.7E-42	2E-39	9E-38	ENSRNOG	protein_coding
Etfbkmt	1.3866	2.61462	5.9E-06	9.2E-05	0.11343	ENSRNOG	protein_coding
AABR070	-2.0408	-4.1147	0.00667	0.02831	1	ENSRNOG	pseudogene
Mybl1	-1.8258	-3.5451	3.4E-13	2.1E-11	6.5E-09	ENSRNOG	protein_coding
Adhfe1	3.05135	8.28989	2.4E-09	8E-08	4.5E-05	ENSRNOG	protein_coding
Rrs1	-1.37	-2.5847	4.3E-06	7E-05	0.08195	ENSRNOG	protein_coding
Rp1	2.91898	7.56314	0.00021	0.00181	1	ENSRNOG	protein_coding
Plag1	2.37563	5.18963	4.6E-07	9.8E-06	0.00881	ENSRNOG	protein_coding
Sdr16c5	6.24659	75.9297	0.00823	0.0333	1	ENSRNOG	protein_coding
Ubxn2b	1.60506	3.04208	1.2E-06	2.4E-05	0.02377	ENSRNOG	protein_coding
Gdf6	3.39862	10.546	5E-08	1.3E-06	0.00096	ENSRNOG	protein_coding
Tp53inp1	4.16396	17.9258	1.7E-24	2.7E-22	3.3E-20	ENSRNOG	protein_coding
Gem	3.22841	9.37237	0.00044	0.00325	1	ENSRNOG	protein_coding
Cdh17	2.73399	6.65291	3.2E-14	2.2E-12	6.1E-10	ENSRNOG	protein_coding
Tmem67	1.6216	3.07716	1.8E-07	4.3E-06	0.00349	ENSRNOG	protein_coding
Slc26a7	3.26001	9.5799	8.1E-09	2.4E-07	0.00016	ENSRNOG	protein_coding
LOC1009	1.96994	3.91752	0.00817	0.03309	1	ENSRNOG	protein_coding
Mms22l	-1.549	-2.9262	9.5E-05	0.00097	1	ENSRNOG	protein_coding
Gabrr2	2.85386	7.22932	0.0104	0.03981	1	ENSRNOG	protein_coding
Srsf12	4.12208	17.4129	4.3E-08	1.1E-06	0.00083	ENSRNOG	protein_coding
Pnrc1	2.11528	4.33274	5.4E-14	3.6E-12	1E-09	ENSRNOG	protein_coding
AABR070	-5.2574	-38.251	0.00865	0.0346	1	ENSRNOG	pseudogene
Mob3b	1.62174	3.07746	0.0003	0.00242	1	ENSRNOG	protein_coding
LOC1025	1.61366	3.06027	0.00011	0.00111	1	ENSRNOG	lincRNA
Ddx58	1.78043	3.43529	9.7E-06	0.00014	0.18734	ENSRNOG	protein_coding
RGD1309	1.47317	2.77632	0.00368	0.01764	1	ENSRNOG	protein_coding
Il11ra1	1.65751	3.15471	2.9E-06	4.9E-05	0.05519	ENSRNOG	protein_coding
Phf24	-1.3622	-2.5708	0.00038	0.00292	1	ENSRNOG	protein_coding
LOC1003	2.52344	5.74953	0.00412	0.0193	1	ENSRNOG	protein_coding
Fam214b	1.61148	3.05565	2.5E-09	8.6E-08	4.9E-05	ENSRNOG	protein_coding
Npr2	1.45919	2.74955	4.1E-07	8.8E-06	0.0078	ENSRNOG	protein_coding
Tmem8b	1.53876	2.90545	0.00034	0.00266	1	ENSRNOG	protein_coding
Hrct1	3.35204	10.2109	1.1E-23	1.6E-21	2.1E-19	ENSRNOG	protein_coding
Reck	1.72215	3.29928	2E-08	5.6E-07	0.00038	ENSRNOG	protein_coding
Melk	-1.5236	-2.8752	0.00011	0.00112	1	ENSRNOG	protein_coding

Frmpd1	-2.5512	-5.8614	9.7E-06	0.00014	0.18559	ENSRNOG	protein_coding
Mcart1	-1.5207	-2.8693	0.00067	0.00456	1	ENSRNOG	protein_coding
Xpa	1.35094	2.55078	0.00266	0.01377	1	ENSRNOG	protein_coding
Tbc1d2	-1.4722	-2.7744	4.6E-05	0.00053	0.88979	ENSRNOG	protein_coding
Murc	-2.0962	-4.276	1.8E-17	1.8E-15	3.5E-13	ENSRNOG	protein_coding
Smc2	-1.6109	-3.0544	8.3E-05	0.00087	1	ENSRNOG	protein_coding
Nipsnap3l	1.49746	2.82346	9E-12	4.4E-10	1.7E-07	ENSRNOG	protein_coding
Abca1	3.19509	9.15839	5.8E-21	7.3E-19	1.1E-16	ENSRNOG	protein_coding
Ctnnal1	1.93839	3.83277	1E-11	5E-10	2E-07	ENSRNOG	protein_coding
Svep1	2.82935	7.10754	1.7E-14	1.2E-12	3.3E-10	ENSRNOG	protein_coding
Ptgr1	-1.4424	-2.7178	4.5E-07	9.6E-06	0.00865	ENSRNOG	protein_coding
LOC1009	1.41608	2.66859	0.00319	0.01586	1	ENSRNOG	protein_coding
Orm1	3.73073	13.2758	3.7E-22	5.1E-20	7.1E-18	ENSRNOG	protein_coding
Tnc	-3.3007	-9.8541	4.2E-19	4.7E-17	8E-15	ENSRNOG	protein_coding
Pappa	6.31117	79.4057	2.6E-12	1.4E-10	5E-08	ENSRNOG	protein_coding
Tle1	1.44842	2.72908	2.4E-06	4.2E-05	0.04624	ENSRNOG	protein_coding
Lurap1l	1.53255	2.89297	5.6E-08	1.4E-06	0.00107	ENSRNOG	protein_coding
Frem1	1.77579	3.42426	3.7E-11	1.6E-09	7.1E-07	ENSRNOG	protein_coding
Ccdc171	2.68886	6.44805	1.2E-16	1E-14	2.2E-12	ENSRNOG	processed_transcript
Sh3gl2	4.96383	31.2076	3.9E-13	2.3E-11	7.5E-09	ENSRNOG	protein_coding
Acer2	6.24715	75.9591	2.5E-49	1.5E-46	4.8E-45	ENSRNOG	protein_coding
Dmrta1	6.7007	104.019	4.4E-07	9.4E-06	0.00839	ENSRNOG	protein_coding
Usp1	-1.5226	-2.873	0.00032	0.00256	1	ENSRNOG	protein_coding
Lepr	2.02468	4.06903	4.5E-06	7.4E-05	0.08731	ENSRNOG	protein_coding
Sgip1	1.37583	2.59518	0.0001	0.00104	1	ENSRNOG	protein_coding
Plpp3	2.66657	6.34919	3.3E-18	3.3E-16	6.3E-14	ENSRNOG	protein_coding
Pcsk9	2.01171	4.0326	0.00728	0.03027	1	ENSRNOG	protein_coding
Ttc22	2.84737	7.19686	1.3E-29	2.5E-27	2.5E-25	ENSRNOG	protein_coding
Mroh7	6.06816	67.0965	7.7E-08	1.9E-06	0.00148	ENSRNOG	protein_coding
Fam151a	4.43397	21.6152	4.9E-36	1.5E-33	9.5E-32	ENSRNOG	protein_coding
Acot11	4.95933	31.1106	8.9E-48	4.8E-45	1.7E-43	ENSRNOG	protein_coding
Ldlrad1	2.93529	7.64911	1.8E-05	0.00024	0.34355	ENSRNOG	protein_coding
Podn	1.32562	2.5064	5.2E-11	2.2E-09	1E-06	ENSRNOG	protein_coding
Echdc2	6.03478	65.5616	0.01095	0.04132	1	ENSRNOG	protein_coding
Zyg11a	3.90929	15.025	4.7E-06	7.6E-05	0.09038	ENSRNOG	protein_coding
Zcchc11	1.60382	3.03947	4.2E-10	1.6E-08	8.1E-06	ENSRNOG	protein_coding
Orc1	-1.3378	-2.5276	0.00393	0.01858	1	ENSRNOG	protein_coding
Rab3b	-3.4122	-10.646	3.7E-19	4.2E-17	7E-15	ENSRNOG	protein_coding
Ttc39a	2.15475	4.45293	1.4E-05	0.00019	0.26904	ENSRNOG	protein_coding
AABR070	-1.4012	-2.6411	0.01144	0.04287	1	ENSRNOG	pseudogene
Skint10	2.72793	6.62506	0.00051	0.00366	1	ENSRNOG	protein_coding
AABR070	-1.788	-3.4534	0.00085	0.00542	1	ENSRNOG	lincRNA
Stil	-1.3434	-2.5375	0.00016	0.00147	1	ENSRNOG	protein_coding
Hpdl	-1.7962	-3.4731	0.00604	0.02613	1	ENSRNOG	protein_coding
Tmem53	1.38127	2.60498	0.00124	0.00736	1	ENSRNOG	protein_coding
Artn	-3.4764	-11.13	1.6E-07	3.7E-06	0.00298	ENSRNOG	protein_coding
Ccdc30	2.47398	5.55573	0.00522	0.02323	1	ENSRNOG	protein_coding
Col9a2	6.21592	74.3326	0.01004	0.0388	1	ENSRNOG	protein_coding
AC124205	-1.4335	-2.7011	0.00963	0.03763	1	ENSRNOG	pseudogene

Mfsd2a	1.73783	3.33534	0.00025	0.00211	1	ENSRNOG	protein_coding
Dnali1	1.99232	3.97877	9.5E-05	0.00097	1	ENSRNOG	protein_coding
Snip1	9.23855	604.062	7.2E-10	2.6E-08	1.4E-05	ENSRNOG	protein_coding
Eva1b	1.36469	2.57522	5E-07	1.1E-05	0.00964	ENSRNOG	protein_coding
Sh3d21	1.42191	2.67939	0.00112	0.00678	1	ENSRNOG	protein_coding
Col8a2	1.41726	2.67078	5.9E-09	1.8E-07	0.00011	ENSRNOG	protein_coding
Ago3	1.66128	3.16298	1.7E-10	6.7E-09	3.2E-06	ENSRNOG	protein_coding
Ago4	2.36537	5.15286	3.5E-08	9.4E-07	0.00067	ENSRNOG	protein_coding
Clspn	-1.6044	-3.0407	0.00022	0.00192	1	ENSRNOG	protein_coding
Gja4	-2.9652	-7.8091	2.1E-09	7.1E-08	4E-05	ENSRNOG	protein_coding
Gjb3	-1.39	-2.6207	0.00071	0.00471	1	ENSRNOG	protein_coding
AABR070	-1.8882	-3.7018	0.00183	0.01015	1	ENSRNOG	protein_coding
AABR070	-2.2053	-4.6116	0.00039	0.00297	1	ENSRNOG	lincRNA
LOC1009	-1.6833	-3.2116	0.00016	0.00146	1	ENSRNOG	lincRNA
LOC1009	1.50191	2.83217	1.3E-05	0.00018	0.25568	ENSRNOG	protein_coding
Azin2	1.95115	3.86683	0.00016	0.00145	1	ENSRNOG	protein_coding
Tssk3	1.7767	3.42643	0.01115	0.04195	1	ENSRNOG	protein_coding
Ccdc28b	3.97321	15.7056	7.4E-09	2.3E-07	0.00014	ENSRNOG	protein_coding
Fabp3	-1.7677	-3.405	7.2E-06	0.00011	0.13796	ENSRNOG	protein_coding
Ptpru	2.80498	6.98849	0.00041	0.00314	1	ENSRNOG	protein_coding
Ptafr	-1.6518	-3.1424	0.00043	0.00321	1	ENSRNOG	protein_coding
Smpdl3b	-1.5336	-2.895	0.00011	0.00109	1	ENSRNOG	protein_coding
Themis2	1.58079	2.99133	0.00044	0.0033	1	ENSRNOG	protein_coding
Sytl1	1.76706	3.40359	0.00265	0.01372	1	ENSRNOG	protein_coding
Fam46b	1.59663	3.02436	6.1E-09	1.9E-07	0.00012	ENSRNOG	protein_coding
Sfn	2.77486	6.8441	0.00027	0.00225	1	ENSRNOG	protein_coding
Hmgn2	-1.6594	-3.159	3.9E-07	8.5E-06	0.00753	ENSRNOG	protein_coding
Cnksr1	1.8041	3.49211	3E-05	0.00037	0.57833	ENSRNOG	protein_coding
Trim63	3.68094	12.8254	5.1E-16	4.3E-14	9.9E-12	ENSRNOG	protein_coding
Extl1	3.00367	8.02039	2.7E-06	4.7E-05	0.05231	ENSRNOG	protein_coding
Aunip	-1.3473	-2.5443	0.00252	0.01319	1	ENSRNOG	protein_coding
Rsrp1	1.47942	2.78836	6.4E-07	1.3E-05	0.01225	ENSRNOG	protein_coding
Myom3	2.58083	5.98285	0.00021	0.0018	1	ENSRNOG	protein_coding
Ephb2	-1.9245	-3.7961	0.00596	0.02587	1	ENSRNOG	protein_coding
Alpl	1.39223	2.62485	0.00029	0.00239	1	ENSRNOG	protein_coding
Pink1	2.48349	5.5925	1E-14	7.5E-13	2E-10	ENSRNOG	protein_coding
LOC1009	-2.112	-4.3228	0.00062	0.00431	1	ENSRNOG	protein_coding
Camk2n1	3.67711	12.7914	1.4E-05	0.00019	0.27152	ENSRNOG	protein_coding
Mrto4	-1.3972	-2.6338	9.1E-05	0.00094	1	ENSRNOG	protein_coding
Padi3	-3.2017	-9.2003	1.1E-06	2.1E-05	0.02122	ENSRNOG	protein_coding
Clcnkb	3.54151	11.6439	6.1E-10	2.2E-08	1.2E-05	ENSRNOG	protein_coding
Hspb7	3.59124	12.0524	3.9E-34	1E-31	7.6E-30	ENSRNOG	protein_coding
Slc25a34	3.6385	12.4536	0.01222	0.04523	1	ENSRNOG	protein_coding
Agmat	1.50028	2.82897	0.00414	0.01934	1	ENSRNOG	protein_coding
Tmem51	-1.5185	-2.865	7.2E-06	0.00011	0.13745	ENSRNOG	protein_coding
Dhrs3	3.16189	8.95004	5.3E-32	1.2E-29	1E-27	ENSRNOG	protein_coding
Srm	-7.8893	-237.1	2.3E-05	0.00029	0.44567	ENSRNOG	protein_coding
H6pd	1.35989	2.56666	4.1E-05	0.00048	0.79396	ENSRNOG	protein_coding
Slc45a1	2.64106	6.23792	0.0002	0.00175	1	ENSRNOG	protein_coding

Errfi1	1.84377	3.58947	2.5E-13	1.5E-11	4.7E-09	ENSRNOG	protein_coding
Tnfrsf9	2.00123	4.00342	0.00326	0.0161	1	ENSRNOG	protein_coding
Plekhg5	1.41004	2.65744	7.2E-09	2.2E-07	0.00014	ENSRNOG	protein_coding
Tnfrsf25	2.83998	7.16011	4.1E-11	1.8E-09	7.9E-07	ENSRNOG	protein_coding
Rnf207	2.42949	5.38705	0.00011	0.00105	1	ENSRNOG	protein_coding
Smim1	1.84306	3.58769	0.00036	0.00283	1	ENSRNOG	protein_coding
Ccdc27	1.90901	3.75551	0.00621	0.02674	1	ENSRNOG	protein_coding
Prkcz	1.39576	2.63128	6E-05	0.00066	1	ENSRNOG	protein_coding
Mib2	1.89269	3.71326	7.9E-09	2.4E-07	0.00015	ENSRNOG	protein_coding
LOC1003	2.12951	4.3757	0.00182	0.01014	1	ENSRNOG	protein_coding
Tnfrsf4	1.88595	3.69596	0.00036	0.00284	1	ENSRNOG	protein_coding
Perm1	2.73961	6.67891	0.00033	0.00259	1	ENSRNOG	protein_coding
Klhl17	1.54203	2.91204	0.00011	0.00105	1	ENSRNOG	protein_coding
Samd11	2.35536	5.11723	7.7E-07	1.5E-05	0.01476	ENSRNOG	protein_coding
Crim1	-1.4079	-2.6536	9.3E-07	1.8E-05	0.01793	ENSRNOG	protein_coding
Qpct	-1.5637	-2.9561	0.00077	0.00502	1	ENSRNOG	protein_coding
Cyp1b1	-1.7823	-3.4397	1.5E-13	9.9E-12	3E-09	ENSRNOG	protein_coding
Dhx57	1.36506	2.57588	2.1E-05	0.00027	0.39935	ENSRNOG	protein_coding
Slc8a1	-1.8121	-3.5115	4.5E-11	1.9E-09	8.6E-07	ENSRNOG	protein_coding
Plekhh2	1.96897	3.9149	1.9E-08	5.3E-07	0.00036	ENSRNOG	protein_coding
Rasgrp3	-2.6342	-6.2082	4.1E-10	1.5E-08	7.9E-06	ENSRNOG	protein_coding
Clip4	2.27506	4.84019	0.00022	0.00188	1	ENSRNOG	protein_coding
Fam179a	2.4415	5.43207	5.2E-05	0.00059	1	ENSRNOG	protein_coding
Ypel5	1.49832	2.82513	2E-07	4.6E-06	0.00386	ENSRNOG	protein_coding
Lclat1	2.03354	4.09408	5.2E-15	3.9E-13	1E-10	ENSRNOG	protein_coding
Xdh	3.92556	15.1954	1.1E-37	3.6E-35	2.2E-33	ENSRNOG	protein_coding
Trim54	9.22451	598.211	8.4E-05	0.00087	1	ENSRNOG	protein_coding
Tcf23	3.53265	11.5727	5.5E-08	1.4E-06	0.00106	ENSRNOG	protein_coding
Emilin1	1.66813	3.17803	5.4E-06	8.5E-05	0.1034	ENSRNOG	protein_coding
Adcy3	-1.5451	-2.9183	0.00012	0.00119	1	ENSRNOG	protein_coding
Cenpo	-1.5387	-2.9053	0.00019	0.0017	1	ENSRNOG	protein_coding
Atad2b	1.33715	2.52651	2.8E-05	0.00035	0.54553	ENSRNOG	protein_coding
Slc7a15	5.47416	44.4515	0.00031	0.00249	1	ENSRNOG	protein_coding
Sdc1	-2.0643	-4.1822	1.8E-11	8.4E-10	3.5E-07	ENSRNOG	protein_coding
Matn3	1.84809	3.60023	0.00289	0.01472	1	ENSRNOG	protein_coding
Ttc32	-1.3226	-2.5012	0.00038	0.00296	1	ENSRNOG	protein_coding
AABR070	3.86985	14.6198	1.3E-05	0.00018	0.25117	ENSRNOG	lincRNA
Nt5c1b	2.93449	7.64486	2.2E-08	6.2E-07	0.00043	ENSRNOG	protein_coding
Fam49a	1.57151	2.97216	0.00095	0.00591	1	ENSRNOG	protein_coding
Mycn	-1.5764	-2.9822	0.00297	0.01502	1	ENSRNOG	protein_coding
Fam84a	-3.7567	-13.517	1.4E-11	6.6E-10	2.7E-07	ENSRNOG	protein_coding
Trib2	1.92066	3.78595	1.1E-12	6.2E-11	2.1E-08	ENSRNOG	protein_coding
AABR070	4.44586	21.794	0.00061	0.0042	1	ENSRNOG	lincRNA
Lpin1	3.18074	9.06771	1.7E-35	4.7E-33	3.3E-31	ENSRNOG	protein_coding
Greb1	5.08582	33.9612	4.6E-21	5.9E-19	8.8E-17	ENSRNOG	protein_coding
Odc1	-1.6144	-3.0618	0.00364	0.01754	1	ENSRNOG	protein_coding
Cys1	1.49074	2.81033	0.01002	0.03874	1	ENSRNOG	protein_coding
Rrm2	-1.461	-2.753	0.00514	0.02292	1	ENSRNOG	protein_coding
Kidins220	1.61967	3.07305	1.2E-10	4.9E-09	2.3E-06	ENSRNOG	protein_coding

Sox11	-1.8852	-3.6941	0.0019	0.01045	1	ENSRNOG	protein_coding
Sh3yl1	1.38261	2.60739	0.0003	0.00246	1	ENSRNOG	protein_coding
Dus4l	-1.3334	-2.52	0.00027	0.00223	1	ENSRNOG	protein_coding
Hbp1	1.41802	2.67218	1.2E-08	3.5E-07	0.00023	ENSRNOG	protein_coding
AABR070	1.71963	3.29352	0.00079	0.00515	1	ENSRNOG	pseudogene
Nampt	1.35176	2.55223	7.6E-07	1.5E-05	0.01461	ENSRNOG	protein_coding
Tspan13	-1.4809	-2.7913	0.0003	0.00244	1	ENSRNOG	protein_coding
Agmo	2.4148	5.33246	0.00975	0.03795	1	ENSRNOG	protein_coding
Scin	2.83338	7.12743	7.2E-06	0.00011	0.13854	ENSRNOG	protein_coding
Lsmem1	4.22189	18.6602	4.3E-11	1.9E-09	8.3E-07	ENSRNOG	protein_coding
Lrrn3	-1.4737	-2.7773	0.00017	0.0015	1	ENSRNOG	protein_coding
AABR070	-3.0147	-8.0818	5.6E-09	1.8E-07	0.00011	ENSRNOG	protein_coding
Nfkbia	2.0374	4.10505	1.6E-14	1.1E-12	3E-10	ENSRNOG	protein_coding
Brms1l_2	1.34815	2.54586	0.0001	0.00102	1	ENSRNOG	protein_coding
Foxa1	3.09978	8.57287	3.5E-13	2.1E-11	6.8E-09	ENSRNOG	protein_coding
Ttc6	3.02384	8.13329	3.3E-06	5.5E-05	0.06297	ENSRNOG	protein_coding
Mis18bp1	-1.5449	-2.9179	2.7E-05	0.00033	0.51144	ENSRNOG	protein_coding
Mdga2	-2.0329	-4.0922	1.9E-05	0.00025	0.3614	ENSRNOG	protein_coding
Lrr1	-1.5388	-2.9056	0.00262	0.01357	1	ENSRNOG	protein_coding
Pole2	-1.4814	-2.7922	0.00048	0.00353	1	ENSRNOG	protein_coding
Trim9	-3.7512	-13.465	0.00022	0.00189	1	ENSRNOG	protein_coding
L3hypdh	1.76294	3.39388	0.0082	0.03319	1	ENSRNOG	protein_coding
Slc38a6	1.4507	2.73341	6.7E-06	0.0001	0.12844	ENSRNOG	protein_coding
Zbtb25	1.6745	3.1921	8.8E-05	0.00091	1	ENSRNOG	protein_coding
Tmem229	2.53718	5.80455	7.3E-08	1.9E-06	0.00141	ENSRNOG	protein_coding
Actn1	-1.6624	-3.1654	4.3E-08	1.1E-06	0.00083	ENSRNOG	protein_coding
AC118490	-3.1695	-8.9975	0.00414	0.01934	1	ENSRNOG	pseudogene
AABR070	-1.6444	-3.1261	0.00048	0.00353	1	ENSRNOG	protein_coding
LOC5006	-1.4856	-2.8003	2.8E-05	0.00035	0.54369	ENSRNOG	protein_coding
Smoc1	1.35129	2.5514	9E-07	1.8E-05	0.01725	ENSRNOG	protein_coding
Zfyve1	1.48904	2.80702	2E-06	3.7E-05	0.03918	ENSRNOG	protein_coding
Dnal1	1.5661	2.96103	1.6E-08	4.5E-07	0.0003	ENSRNOG	protein_coding
Aldh6a1	1.43621	2.7061	2.4E-11	1.1E-09	4.7E-07	ENSRNOG	protein_coding
Fos	1.48145	2.79228	0.00022	0.00192	1	ENSRNOG	protein_coding
Irf2bpl	1.67077	3.18385	7.7E-10	2.8E-08	1.5E-05	ENSRNOG	protein_coding
AABR070	3.21371	9.27734	0.00324	0.01602	1	ENSRNOG	lincRNA
Samd15	-1.4805	-2.7905	0.00134	0.00783	1	ENSRNOG	protein_coding
Cep128	-1.6625	-3.1656	2.5E-05	0.00031	0.4819	ENSRNOG	processed_transcript
Spata7	1.4919	2.81259	4.4E-07	9.5E-06	0.00854	ENSRNOG	protein_coding
Eml5	2.25819	4.7839	0.00076	0.00498	1	ENSRNOG	protein_coding
Efcab11	-1.3548	-2.5576	0.01111	0.0418	1	ENSRNOG	protein_coding
Tdp1	-1.7272	-3.3109	8.1E-08	2E-06	0.00156	ENSRNOG	protein_coding
Fbln5	2.18331	4.54193	8.5E-09	2.6E-07	0.00016	ENSRNOG	protein_coding
Moap1	9.77947	878.846	4.1E-05	0.00048	0.7805	ENSRNOG	protein_coding
Serpina12	8.9387	490.701	1.4E-12	7.6E-11	2.6E-08	ENSRNOG	protein_coding
LOC5007	3.78585	13.7929	1.1E-32	2.6E-30	2.2E-28	ENSRNOG	protein_coding
LOC1009	8.16992	287.999	0.00155	0.00886	1	ENSRNOG	protein_coding
Hhipl1	1.84	3.58011	1.5E-11	6.8E-10	2.8E-07	ENSRNOG	protein_coding
Cyp46a1	2.11446	4.33027	1.9E-06	3.5E-05	0.03613	ENSRNOG	protein_coding

Slc25a29	1.70424	3.25856	0.00462	0.02108	1	ENSRNOG	protein_coding
Hsp90aa1	-1.6673	-3.1762	4E-06	6.7E-05	0.07749	ENSRNOG	protein_coding
Mok	1.46533	2.76126	0.00058	0.00403	1	ENSRNOG	protein_coding
Ankrd9	-1.5338	-2.8955	5E-06	8E-05	0.0963	ENSRNOG	protein_coding
Aspg	-2.1058	-4.3045	0.00078	0.00511	1	ENSRNOG	protein_coding
LOC6914	3.62241	12.3155	9.5E-06	0.00014	0.18249	ENSRNOG	protein_coding
Tmem179	3.32738	10.0379	1.5E-06	2.8E-05	0.02868	ENSRNOG	protein_coding
Adssl1	2.92838	7.61257	9.1E-05	0.00094	1	ENSRNOG	protein_coding
Gpr132	-2.0349	-4.0981	8.4E-06	0.00012	0.16095	ENSRNOG	protein_coding
LOC1025	2.6036	6.07803	1.7E-07	3.9E-06	0.0032	ENSRNOG	protein_coding
Ptprn2	7.1609	143.102	1E-06	2E-05	0.0192	ENSRNOG	protein_coding
Rapgef5	4.89979	29.8528	1.6E-05	0.00021	0.30218	ENSRNOG	protein_coding
Itgb8	2.68066	6.41151	2.5E-11	1.1E-09	4.9E-07	ENSRNOG	protein_coding
Tmem196	1.58782	3.00594	0.00368	0.01767	1	ENSRNOG	protein_coding
AABR070	2.31367	4.97147	0.00136	0.00794	1	ENSRNOG	pseudogene
Pros1	1.71852	3.29099	2.2E-10	8.6E-09	4.3E-06	ENSRNOG	protein_coding
Timeless	-1.4752	-2.7802	0.00014	0.00129	1	ENSRNOG	protein_coding
Stat2	1.45367	2.73904	6.4E-13	3.7E-11	1.2E-08	ENSRNOG	protein_coding
Pa2g4	-1.4134	-2.6636	8.1E-06	0.00012	0.15496	ENSRNOG	protein_coding
Dgka	1.73222	3.32239	1E-07	2.5E-06	0.00192	ENSRNOG	protein_coding
Mmp19	1.56993	2.9689	4.8E-15	3.6E-13	9.2E-11	ENSRNOG	protein_coding
Mettl7b	-2.5763	-5.964	0.01061	0.04039	1	ENSRNOG	protein_coding
Zfp709l1	1.80547	3.49544	1.4E-07	3.4E-06	0.00273	ENSRNOG	protein_coding
AABR070	2.32932	5.02567	5.4E-11	2.3E-09	1E-06	ENSRNOG	protein_coding
Tle2	2.22003	4.65903	5.9E-08	1.5E-06	0.00113	ENSRNOG	protein_coding
Celf5	2.18532	4.54828	1.6E-05	0.00021	0.30678	ENSRNOG	protein_coding
Matk	1.96475	3.90345	0.00018	0.00159	1	ENSRNOG	protein_coding
Zfr2	3.38256	10.4292	7.7E-12	3.9E-10	1.5E-07	ENSRNOG	protein_coding
Gng7	2.17588	4.5186	0.00078	0.00511	1	ENSRNOG	protein_coding
Lmnb2	-1.6692	-3.1805	6.7E-05	0.00072	1	ENSRNOG	protein_coding
Atp8b3	2.23969	4.72297	1.1E-06	2.1E-05	0.02129	ENSRNOG	protein_coding
Pcsk4	1.7032	3.25623	0.00085	0.00543	1	ENSRNOG	protein_coding
Apc2	1.94272	3.8443	0.00297	0.01501	1	ENSRNOG	protein_coding
Arid3a	1.50416	2.8366	2.8E-05	0.00034	0.53456	ENSRNOG	protein_coding
Misp	6.37499	82.9971	0.00648	0.02761	1	ENSRNOG	protein_coding
Notch3	1.61887	3.07135	6.5E-06	0.0001	0.12562	ENSRNOG	protein_coding
Akap8l	1.89139	3.70994	2.4E-11	1.1E-09	4.7E-07	ENSRNOG	protein_coding
Rasal3	2.77211	6.83106	0.00433	0.02006	1	ENSRNOG	protein_coding
Cyp4f17	2.70903	6.53881	8.1E-12	4E-10	1.6E-07	ENSRNOG	protein_coding
Cyp4f1	4.75496	27.0014	4.7E-05	0.00054	0.90384	ENSRNOG	protein_coding
Cyp4f6	2.00503	4.01396	1.9E-06	3.5E-05	0.03655	ENSRNOG	protein_coding
Adamts10	2.77835	6.86069	1.2E-10	4.9E-09	2.3E-06	ENSRNOG	protein_coding
Myo1f	4.20621	18.4584	1.1E-09	3.8E-08	2.1E-05	ENSRNOG	protein_coding
Pram1	2.25537	4.77455	0.00711	0.02977	1	ENSRNOG	protein_coding
March2	1.33545	2.52354	1.9E-08	5.4E-07	0.00037	ENSRNOG	protein_coding
Angptl4	3.86806	14.6017	1.4E-06	2.7E-05	0.02748	ENSRNOG	protein_coding
Tcp11l2	4.30179	19.7228	1.5E-29	2.8E-27	2.8E-25	ENSRNOG	protein_coding
LOC1009	4.6011	24.27	2.5E-05	0.00031	0.47962	ENSRNOG	protein_coding
Appl2	1.57379	2.97685	2.2E-06	3.9E-05	0.04229	ENSRNOG	protein_coding

Glt8d2	2.07035	4.19988	0.01214	0.04499	1	ENSRNOG	protein_coding
LOC3628	1.36618	2.57788	0.00574	0.02508	1	ENSRNOG	protein_coding
Parpbp	-1.4548	-2.7411	0.00067	0.00452	1	ENSRNOG	protein_coding
Dram1	1.80117	3.48502	8.7E-21	1.1E-18	1.7E-16	ENSRNOG	protein_coding
Gas2l3	-1.389	-2.6189	0.00021	0.0018	1	ENSRNOG	protein_coding
Cfap54	1.67836	3.20064	0.01257	0.04623	1	ENSRNOG	protein_coding
AABR070	2.85073	7.21366	0.0032	0.01588	1	ENSRNOG	lincRNA
Socs2	1.41177	2.66063	0.00163	0.00922	1	ENSRNOG	protein_coding
Nudt4	2.1009	4.28976	6.3E-13	3.7E-11	1.2E-08	ENSRNOG	protein_coding
Btg1	1.49292	2.81459	6.8E-07	1.4E-05	0.01305	ENSRNOG	protein_coding
Acss3	6.26841	77.0865	1E-09	3.7E-08	2E-05	ENSRNOG	protein_coding
Ptprq	2.17604	4.51913	2E-15	1.6E-13	3.9E-11	ENSRNOG	protein_coding
E2f7	-1.5178	-2.8636	0.00063	0.00431	1	ENSRNOG	protein_coding
Phlda1	-2.4413	-5.4312	4.3E-16	3.6E-14	8.2E-12	ENSRNOG	protein_coding
Glipr1	-1.5612	-2.9511	5.1E-14	3.4E-12	9.8E-10	ENSRNOG	protein_coding
Caps2	1.36149	2.56951	0.009	0.03566	1	ENSRNOG	protein_coding
Lgr5	3.10949	8.63079	5.7E-09	1.8E-07	0.00011	ENSRNOG	protein_coding
Tspan8	2.16624	4.48852	1.9E-11	8.7E-10	3.7E-07	ENSRNOG	protein_coding
Ptprr	-2.6243	-6.166	0.00052	0.00371	1	ENSRNOG	protein_coding
Kcnmb4	6.07426	67.3803	2.4E-08	6.6E-07	0.00046	ENSRNOG	protein_coding
Cpm	-1.6714	-3.1852	2.6E-06	4.5E-05	0.05059	ENSRNOG	protein_coding
Mdm1	-1.4108	-2.6588	1.8E-07	4.2E-06	0.0034	ENSRNOG	protein_coding
Wif1	3.14291	8.83306	1.8E-07	4.2E-06	0.00342	ENSRNOG	protein_coding
RGD1561	2.574	5.95456	0.00355	0.0172	1	ENSRNOG	protein_coding
Irak3	1.56891	2.96681	3.9E-06	6.4E-05	0.0745	ENSRNOG	protein_coding
AABR070	3.53483	11.5902	0.01023	0.03928	1	ENSRNOG	lincRNA
Mettl21b	2.4098	5.31401	0.00807	0.03281	1	ENSRNOG	protein_coding
Agap2	2.68537	6.43246	2.4E-06	4.3E-05	0.04689	ENSRNOG	protein_coding
Slc26a10	2.97389	7.85654	0.00012	0.00118	1	ENSRNOG	protein_coding
Arhgef25	1.35971	2.56634	4.6E-07	9.8E-06	0.00889	ENSRNOG	protein_coding
Dtx3	1.38546	2.61254	5.4E-05	0.0006	1	ENSRNOG	protein_coding
Gpr182	1.57092	2.97093	0.00216	0.01161	1	ENSRNOG	protein_coding
Spag1	1.82589	3.54526	7.8E-09	2.4E-07	0.00015	ENSRNOG	protein_coding
Ncald	1.65466	3.14849	1.7E-10	6.8E-09	3.3E-06	ENSRNOG	protein_coding
Angpt1	4.56093	23.6036	5.8E-40	2.2E-37	1.1E-35	ENSRNOG	protein_coding
Pkhd1l1	3.00746	8.04145	4E-05	0.00047	0.77038	ENSRNOG	protein_coding
Tnfrsf11b	1.943	3.84505	2.2E-08	6E-07	0.00041	ENSRNOG	protein_coding
Enpp2	2.23016	4.69187	0.00467	0.02123	1	ENSRNOG	protein_coding
Dscc1	-1.3729	-2.5899	5.2E-06	8.2E-05	0.09994	ENSRNOG	protein_coding
Sntb1	-1.4889	-2.8067	5.5E-06	8.6E-05	0.10575	ENSRNOG	protein_coding
Atad2	-1.5942	-3.0193	4.9E-06	7.9E-05	0.09452	ENSRNOG	protein_coding
Fbxo32	3.70685	13.0579	1.6E-11	7.3E-10	3E-07	ENSRNOG	protein_coding
Klhl38	5.83218	56.9721	1.5E-11	7.1E-10	2.9E-07	ENSRNOG	protein_coding
Rn60_7_1	1.3371	2.52644	0.00144	0.00829	1	ENSRNOG	lincRNA
Slurp1	-2.6462	-6.2604	0.00191	0.01051	1	ENSRNOG	protein_coding
Psca	-1.5079	-2.8441	0.0036	0.0174	1	ENSRNOG	protein_coding
Ly6e	5.95766	62.1489	1.6E-07	3.8E-06	0.0031	ENSRNOG	protein_coding
Naprt	1.64704	3.1319	0.00033	0.00259	1	ENSRNOG	protein_coding
Nrbp2	2.22791	4.68454	1.2E-08	3.4E-07	0.00022	ENSRNOG	protein_coding

Spatc1	4.13986	17.6288	0.00227	0.01211	1	ENSRNOG	protein_coding
Oplah	1.48925	2.80744	2.1E-09	7.1E-08	4E-05	ENSRNOG	protein_coding
Wdr97	3.55112	11.7218	1.3E-05	0.00018	0.24862	ENSRNOG	protein_coding
Scx	-2.147	-4.4289	0.00011	0.00106	1	ENSRNOG	protein_coding
Foxh1	-2.1208	-4.3494	0.00589	0.02562	1	ENSRNOG	protein_coding
Mfsd3	1.54997	2.92811	2.7E-05	0.00034	0.52671	ENSRNOG	protein_coding
Recql4	-1.4384	-2.7101	0.00043	0.00324	1	ENSRNOG	protein_coding
LOC1083	7.56304	189.105	0.00311	0.01553	1	ENSRNOG	protein_coding
Apol3	2.64852	6.27023	0.0017	0.00954	1	ENSRNOG	protein_coding
Pole3	-1.4913	-2.8115	0.00027	0.00223	1	ENSRNOG	protein_coding
Tmprss6	2.10372	4.29817	0.00248	0.01299	1	ENSRNOG	protein_coding
Elfn2	-2.3015	-4.9296	0.00023	0.00196	1	ENSRNOG	protein_coding
Lgals2	-5.7965	-55.58	2.6E-06	4.5E-05	0.04999	ENSRNOG	protein_coding
Fam227a	4.51799	22.9114	1.6E-05	0.00022	0.31289	ENSRNOG	protein_coding
Nptxr	-3.0107	-8.0598	1.8E-27	3.2E-25	3.5E-23	ENSRNOG	protein_coding
Syngr1	-1.3381	-2.5281	0.00415	0.01938	1	ENSRNOG	protein_coding
Cyp2d4	5.38757	41.8619	0.00011	0.00108	1	ENSRNOG	protein_coding
Cyp2d1	1.72038	3.29523	0.00426	0.0198	1	ENSRNOG	protein_coding
Tspo	1.90936	3.75643	2.5E-14	1.8E-12	4.9E-10	ENSRNOG	protein_coding
Parvb	-1.3728	-2.5896	5.5E-05	0.00061	1	ENSRNOG	protein_coding
Fbln1	-1.9924	-3.979	2.7E-06	4.7E-05	0.05238	ENSRNOG	protein_coding
Wnt7b	-7.9127	-240.97	8.1E-06	0.00012	0.15484	ENSRNOG	protein_coding
Gtse1	-1.602	-3.0357	0.00014	0.00133	1	ENSRNOG	protein_coding
Mlc1	4.03915	16.4402	0.00781	0.03197	1	ENSRNOG	protein_coding
Syt10	-4.5681	-23.721	0.01225	0.04531	1	ENSRNOG	protein_coding
Abcd2	1.36423	2.57438	0.00014	0.00135	1	ENSRNOG	protein_coding
Prickle1	1.4478	2.72791	2.7E-10	1E-08	5.1E-06	ENSRNOG	protein_coding
Endou	2.65351	6.29198	0.00015	0.00139	1	ENSRNOG	protein_coding
Rapgef3	3.2714	9.65585	9.6E-17	8.5E-15	1.8E-12	ENSRNOG	protein_coding
Ccdc184	2.55405	5.87281	0.00129	0.00758	1	ENSRNOG	protein_coding
Rnd1	-2.7945	-6.9381	5.8E-09	1.8E-07	0.00011	ENSRNOG	protein_coding
Wnt10b	-1.7149	-3.2827	1.7E-06	3.2E-05	0.03351	ENSRNOG	protein_coding
Lmbr1l	2.2617	4.79557	3.8E-08	1E-06	0.00073	ENSRNOG	protein_coding
Troap	-1.4659	-2.7624	0.00197	0.01078	1	ENSRNOG	protein_coding
C1ql4	2.29568	4.90987	0.00216	0.01161	1	ENSRNOG	protein_coding
Prpf40b	1.37437	2.59255	7.9E-06	0.00012	0.15124	ENSRNOG	protein_coding
Nckap5l	1.72702	3.31043	2.5E-10	9.8E-09	4.9E-06	ENSRNOG	protein_coding
Mettl7a	2.41276	5.32492	6.6E-16	5.4E-14	1.3E-11	ENSRNOG	protein_coding
Pou6f1	1.34735	2.54444	5.4E-05	0.00061	1	ENSRNOG	protein_coding
AABR070	1.65271	3.14423	0.00309	0.01547	1	ENSRNOG	lincRNA
Grasp	1.88432	3.69178	4.7E-13	2.8E-11	9.1E-09	ENSRNOG	protein_coding
Rn50_7_1	2.96454	7.80575	0.0016	0.00911	1	ENSRNOG	lincRNA
Nr4a1	1.5684	2.96575	9.3E-13	5.3E-11	1.8E-08	ENSRNOG	protein_coding
Krt5	4.6179	24.5542	2E-05	0.00026	0.38591	ENSRNOG	protein_coding
Krt76	2.08291	4.23661	0.01328	0.04822	1	ENSRNOG	protein_coding
Krt79	-1.3364	-2.5252	0.00395	0.01867	1	ENSRNOG	protein_coding
Znf740	1.41639	2.66916	6.5E-08	1.7E-06	0.00124	ENSRNOG	protein_coding
Espl1	-1.5044	-2.837	0.00068	0.00458	1	ENSRNOG	protein_coding
Npff	2.11993	4.34674	0.00287	0.01463	1	ENSRNOG	protein_coding

LOC1009	3.11986	8.69306	3E-14	2.1E-12	5.8E-10	ENSRNOG	protein_coding	
Zfp385a	1.3706	2.58578	9.3E-09	2.8E-07	0.00018	ENSRNOG	protein_coding	
AABR070	-2.1053	-4.3029	0.00127	0.00749	1	ENSRNOG	lincRNA	
Calcoco1	3.27097	9.65294	1.5E-14	1.1E-12	2.8E-10	ENSRNOG	protein_coding	
Casp1	2.14895	4.43505	3.3E-05	0.0004	0.63746	ENSRNOG	protein_coding	
Casp4	2.29774	4.91688	7.3E-12	3.7E-10	1.4E-07	ENSRNOG	protein_coding	
Pdgfd	5.14211	35.3125	6.3E-10	2.3E-08	1.2E-05	ENSRNOG	protein_coding	
AABR070	2.84479	7.18403	0.01351	0.04889	1	ENSRNOG	lincRNA	
Kdm4d	2.83288	7.12497	0.00333	0.01635	1	ENSRNOG	protein_coding	
Zfp560	1.44691	2.72624	1.4E-06	2.7E-05	0.0269	ENSRNOG	protein_coding	
Col5a3	-1.7269	-3.3101	1.1E-08	3.3E-07	0.00022	ENSRNOG	protein_coding	
Rdh8	-2.9364	-7.6547	0.00878	0.03498	1	ENSRNOG	protein_coding	
Dnmt1	-1.3854	-2.6124	2.2E-05	0.00028	0.42711	ENSRNOG	protein_coding	
Pde4a	2.00844	4.02347	0.00107	0.00654	1	ENSRNOG	protein_coding	
Slc44a2	1.60495	3.04186	8.8E-09	2.6E-07	0.00017	ENSRNOG	protein_coding	
LOC6911	1.97613	3.93436	0.00357	0.01728	1	ENSRNOG	protein_coding	
Spc24	-1.3259	-2.507	0.00561	0.02466	1	ENSRNOG	protein_coding	
Tmem205	1.85609	3.62025	9.5E-06	0.00014	0.18341	ENSRNOG	protein_coding	
Rgl3	1.60372	3.03926	5.3E-06	8.3E-05	0.10104	ENSRNOG	protein_coding	
Ccdc151	1.88111	3.68359	0.00072	0.0048	1	ENSRNOG	protein_coding	
Cnn1	-3.3434	-10.15	3.4E-09	1.1E-07	6.5E-05	ENSRNOG	protein_coding	
AABR070	1.70131	3.25196	0.00142	0.0082	1	ENSRNOG	lincRNA	
LOC1036	1.58803	3.00638	0.00238	0.01257	1	ENSRNOG	protein_coding	
Anln	-1.4803	-2.7901	0.00098	0.00609	1	ENSRNOG	protein_coding	
Eepd1	2.17279	4.50895	2.2E-12	1.2E-10	4.2E-08	ENSRNOG	protein_coding	
Glb1l2	-3.9268	-15.208	1.5E-30	2.9E-28	2.8E-26	ENSRNOG	protein_coding	
Glb1l3	-3.3326	-10.074	0.00137	0.00798	1	ENSRNOG	protein_coding	
Igsf9b	-2.3922	-5.2496	7.9E-05	0.00083	1	ENSRNOG	protein_coding	
Ntm	-1.9396	-3.836	2.7E-06	4.7E-05	0.05281	ENSRNOG	protein_coding	
Adamts8	-6.8595	-116.12	8E-52	5.8E-49	1.5E-47	ENSRNOG	protein_coding	
Hyls1	-1.4486	-2.7293	9.9E-06	0.00014	0.19056	ENSRNOG	protein_coding	
Tmem218	1.77386	3.41968	2.7E-06	4.7E-05	0.05232	ENSRNOG	protein_coding	
Slc37a2	2.70883	6.53791	0.00014	0.00135	1	ENSRNOG	protein_coding	
Scn3b	2.01456	4.04057	0.00175	0.00979	1	ENSRNOG	protein_coding	
Gramd1b	3.75918	13.5402	5E-14	3.4E-12	9.6E-10	ENSRNOG	protein_coding	
Hspa8_2	-1.5877	-3.0058	0.00356	0.01725	1	ENSRNOG	protein_coding	
Ubash3b	-1.5197	-2.8674	9.7E-06	0.00014	0.18668	ENSRNOG	protein_coding	
Mfrp	5.83486	57.0777	3.7E-41	1.5E-38	7.2E-37	ENSRNOG	protein_coding	
C1qtnf5	1.88563	3.69514	4.4E-13	2.7E-11	8.5E-09	ENSRNOG	protein_coding	
Abcg4	1.47257	2.77516	0.0041	0.01921	1	ENSRNOG	protein_coding	
Tmem25	2.27019	4.82385	4.3E-05	0.0005	0.8263	ENSRNOG	protein_coding	
Mpzl2	1.84297	3.58747	4.1E-05	0.00048	0.78082	ENSRNOG	protein_coding	
Jaml	4.54785	23.3905	3.1E-17	2.8E-15	5.9E-13	ENSRNOG	protein_coding	
Scn4b	7.98086	252.626	4.6E-12	2.4E-10	8.9E-08	ENSRNOG	protein_coding	
Tagln	-2.7921	-6.9263	2.9E-08	7.8E-07	0.00055	ENSRNOG	protein_coding	
Sidt2	1.58931	3.00906	4.6E-08	1.2E-06	0.00088	ENSRNOG	protein_coding	
ENSRNO	2.01269	4.03533	0.00109	0.00661	1	ENSRNOG	protein_coding	
Nnmt	2.39082	5.24457	4.6E-16	3.8E-14	8.9E-12	ENSRNOG	protein_coding	
Zbtb16	4.63046	24.769	1E-35	2.8E-33	1.9E-31	ENSRNOG	protein_coding	

Plet1	4.80458	27.9461	1.9E-30	3.8E-28	3.7E-26	ENSRNOG	protein_coding
Tex12_1	3.09858	8.56573	0.00038	0.00295	1	ENSRNOG	protein_coding
Il18	2.94523	7.70198	0.00059	0.00413	1	ENSRNOG	protein_coding
Dixdc1	1.33343	2.52001	2.3E-05	0.00029	0.43405	ENSRNOG	protein_coding
Hspb2	2.24149	4.72884	0.00309	0.01548	1	ENSRNOG	protein_coding
Cryab	1.94893	3.86087	8.6E-13	4.9E-11	1.6E-08	ENSRNOG	protein_coding
Elmod1	-2.2039	-4.6072	0.00044	0.00329	1	ENSRNOG	protein_coding
Cyp19a1	-2.9189	-7.5627	9.4E-08	2.3E-06	0.0018	ENSRNOG	protein_coding
Acsbg1	6.47714	89.0867	0.00846	0.03401	1	ENSRNOG	protein_coding
Odf3l1	-1.9342	-3.8216	0.00462	0.02108	1	ENSRNOG	protein_coding
Ulk3	1.54665	2.92139	1.3E-05	0.00018	0.25134	ENSRNOG	protein_coding
Islr	4.24562	18.9697	4.6E-05	0.00053	0.88153	ENSRNOG	protein_coding
Tbc1d21	2.24649	4.74528	0.0107	0.04065	1	ENSRNOG	protein_coding
Hcn4	-1.4806	-2.7907	0.00126	0.00747	1	ENSRNOG	protein_coding
Uaca	-1.5482	-2.9245	4.6E-09	1.5E-07	8.8E-05	ENSRNOG	protein_coding
Kif23	-1.6666	-3.1747	0.00017	0.00152	1	ENSRNOG	protein_coding
Coro2b	-1.5772	-2.9839	3.5E-05	0.00042	0.68186	ENSRNOG	protein_coding
Itga11	1.71943	3.29306	5.6E-09	1.8E-07	0.00011	ENSRNOG	protein_coding
Calml4	3.75623	13.5125	7.7E-29	1.4E-26	1.5E-24	ENSRNOG	protein_coding
Iqch	2.89772	7.4525	0.0079	0.03225	1	ENSRNOG	protein_coding
Igdcc4	1.55602	2.94041	0.00044	0.00327	1	ENSRNOG	protein_coding
Cilp	4.66423	25.3556	9.1E-19	9.6E-17	1.7E-14	ENSRNOG	protein_coding
Rasl12	-1.7087	-3.2687	0.00073	0.00481	1	ENSRNOG	protein_coding
Slc51b	-5.2803	-38.861	0.00373	0.01783	1	ENSRNOG	protein_coding
Pif1	-1.5076	-2.8434	0.00727	0.03024	1	ENSRNOG	protein_coding
Ns5atp9	-1.3531	-2.5547	0.00123	0.0073	1	ENSRNOG	protein_coding
Dapk2	2.63795	6.22448	8.5E-12	4.2E-10	1.6E-07	ENSRNOG	protein_coding
Vps13c	1.33075	2.51533	6.7E-07	1.4E-05	0.01292	ENSRNOG	protein_coding
Fam81a	-1.6066	-3.0453	0.0002	0.00179	1	ENSRNOG	protein_coding
Cgnl1	-1.9967	-3.9909	0.00284	0.01452	1	ENSRNOG	protein_coding
Mns1	-1.3231	-2.502	0.00044	0.0033	1	ENSRNOG	protein_coding
Fam214a	3.22681	9.36198	4.5E-13	2.7E-11	8.6E-09	ENSRNOG	protein_coding
Myo5c	-2.0194	-4.0541	2.5E-05	0.00031	0.48256	ENSRNOG	protein_coding
Mapk6	-1.4609	-2.7529	1.9E-07	4.5E-06	0.00369	ENSRNOG	protein_coding
Bmp5	1.39954	2.63818	1.1E-10	4.5E-09	2.1E-06	ENSRNOG	protein_coding
Hmgcll1	1.46304	2.75688	1.2E-05	0.00016	0.22273	ENSRNOG	protein_coding
Gsta4	2.85987	7.25952	4E-10	1.5E-08	7.7E-06	ENSRNOG	protein_coding
Gsta5	-8.1983	-293.73	3.8E-06	6.3E-05	0.0731	ENSRNOG	protein_coding
Slc17a5	1.4227	2.68087	3.2E-09	1.1E-07	6.1E-05	ENSRNOG	protein_coding
Col12a1	2.21262	4.63517	7.5E-19	8.1E-17	1.4E-14	ENSRNOG	protein_coding
Mei4	6.86337	116.434	4.7E-07	1E-05	0.00904	ENSRNOG	protein_coding
Ttk	-1.3433	-2.5374	0.0007	0.00469	1	ENSRNOG	protein_coding
Trim43a	1.75532	3.376	0.00728	0.03026	1	ENSRNOG	protein_coding
LOC5010	3.34038	10.1287	0.00045	0.00335	1	ENSRNOG	protein_coding
Rasgrf1	-2.5354	-5.7974	0.00035	0.00277	1	ENSRNOG	protein_coding
Tbc1d2b	1.57705	2.9836	8.8E-09	2.6E-07	0.00017	ENSRNOG	protein_coding
Plod2	2.42418	5.36722	1.9E-18	1.9E-16	3.6E-14	ENSRNOG	protein_coding
RGD1309	1.95359	3.87338	8.8E-09	2.6E-07	0.00017	ENSRNOG	protein_coding
Pik3cb	-1.5798	-2.9893	3.7E-06	6.2E-05	0.07129	ENSRNOG	protein_coding

Il20rb	2.21147	4.63145	1.7E-07	4E-06	0.00326	ENSRNOG	protein_coding
Cdv3	-1.7729	-3.4173	1.4E-09	4.8E-08	2.6E-05	ENSRNOG	protein_coding
Nphp3	1.34263	2.53614	1.3E-06	2.4E-05	0.02446	ENSRNOG	protein_coding
Cish	1.40896	2.65545	3.4E-05	0.00041	0.6604	ENSRNOG	protein_coding
Zmynd10	1.57927	2.98818	0.00092	0.00579	1	ENSRNOG	protein_coding
Hyal1	2.69507	6.47587	5.4E-09	1.7E-07	0.0001	ENSRNOG	protein_coding
Hyal3	1.42257	2.68063	0.00045	0.00331	1	ENSRNOG	protein_coding
Ifrd2	-1.3783	-2.5997	0.00015	0.0014	1	ENSRNOG	protein_coding
Lsmem2	4.81093	28.0694	0.00101	0.00626	1	ENSRNOG	protein_coding
Uba7	1.50234	2.83301	1.4E-05	0.00019	0.26295	ENSRNOG	protein_coding
Fam212a	-2.8564	-7.2419	0.00122	0.00724	1	ENSRNOG	protein_coding
Mst1	2.8467	7.19353	3.9E-05	0.00046	0.75837	ENSRNOG	protein_coding
Bsn	1.76069	3.38861	0.00042	0.00318	1	ENSRNOG	protein_coding
Amt	1.82908	3.55309	0.00069	0.00462	1	ENSRNOG	protein_coding
Prkar2a	-1.3811	-2.6046	2.9E-06	4.9E-05	0.05479	ENSRNOG	protein_coding
Ip6k2	1.57669	2.98285	7.7E-08	1.9E-06	0.00147	ENSRNOG	protein_coding
Col7a1	-3.1346	-8.7825	7.5E-07	1.5E-05	0.01441	ENSRNOG	protein_coding
Kif9	2.44908	5.46069	4.8E-05	0.00054	0.91446	ENSRNOG	protein_coding
Prss42	6.81858	112.875	0.00565	0.0248	1	ENSRNOG	protein_coding
Als2cl	1.96851	3.91364	7.9E-12	4E-10	1.5E-07	ENSRNOG	protein_coding
Ccrl2	2.35141	5.10321	0.0001	0.00101	1	ENSRNOG	protein_coding
Stac	-1.8692	-3.6534	9.5E-05	0.00097	1	ENSRNOG	protein_coding
Gpd1l	1.70448	3.2591	4.6E-08	1.2E-06	0.00088	ENSRNOG	protein_coding
Acvr2b	1.5624	2.95344	0.00196	0.01073	1	ENSRNOG	protein_coding
Ttc21a	2.60631	6.08946	0.00018	0.00158	1	ENSRNOG	protein_coding
Entpd3	-1.7007	-3.2507	1.7E-05	0.00022	0.32091	ENSRNOG	protein_coding
Klhl40	-1.7921	-3.4631	0.00192	0.01054	1	ENSRNOG	protein_coding
Snrk	1.74516	3.35231	2.3E-08	6.4E-07	0.00044	ENSRNOG	protein_coding
Tmem158	4.20917	18.4963	1.7E-11	7.7E-10	3.2E-07	ENSRNOG	protein_coding
Ccr1	-5.0988	-34.268	1.7E-13	1.1E-11	3.2E-09	ENSRNOG	protein_coding
Sgo1	-1.4219	-2.6795	0.00026	0.00214	1	ENSRNOG	protein_coding
Pot1b	1.66041	3.16106	5.9E-07	1.2E-05	0.01132	ENSRNOG	protein_coding
LOC3161	5.63675	49.7543	2.5E-09	8.4E-08	4.8E-05	ENSRNOG	protein_coding
Kdm4b	1.83219	3.56078	5.5E-08	1.4E-06	0.00106	ENSRNOG	protein_coding
Uhrf1	-1.5136	-2.8552	0.00073	0.00481	1	ENSRNOG	protein_coding
Sema6b	1.79261	3.46442	0.00051	0.00365	1	ENSRNOG	protein_coding
Plin4	5.51386	45.6917	8.8E-43	3.9E-40	1.7E-38	ENSRNOG	protein_coding
Chaf1a	-1.4045	-2.6472	6.5E-05	0.0007	1	ENSRNOG	protein_coding
Stap2	1.684	3.21318	0.00123	0.0073	1	ENSRNOG	protein_coding
Fsd1	1.81401	3.51619	2.1E-07	4.8E-06	0.00407	ENSRNOG	protein_coding
Daam2	1.62163	3.07723	9.1E-05	0.00094	1	ENSRNOG	protein_coding
Prickle4	1.81844	3.52699	8.9E-05	0.00092	1	ENSRNOG	protein_coding
Klc4	1.88251	3.68715	7.1E-10	2.6E-08	1.4E-05	ENSRNOG	protein_coding
Cul9	1.40711	2.65205	3.7E-05	0.00044	0.70358	ENSRNOG	protein_coding
Dnph1	-1.5303	-2.8885	0.00038	0.00295	1	ENSRNOG	protein_coding
Tcte1	4.33607	20.197	0.00028	0.00231	1	ENSRNOG	protein_coding
Cyp39a1	2.51263	5.70659	2.6E-11	1.2E-09	5E-07	ENSRNOG	protein_coding
LOC1009	-2.419	-5.3478	0.00138	0.00805	1	ENSRNOG	protein_coding
LOC1009	3.24422	9.47561	0.0037	0.01773	1	ENSRNOG	protein_coding

Mut	1.3712	2.58686	6.2E-09	1.9E-07	0.00012	ENSRNOG	protein_coding
LOC6884	-2.0117	-4.0326	8.8E-16	7.2E-14	1.7E-11	ENSRNOG	protein_coding
Cyp2ac1	-1.6458	-3.1292	0.00019	0.00168	1	ENSRNOG	protein_coding
Crisp3	-3.9844	-15.828	1.3E-33	3.2E-31	2.5E-29	ENSRNOG	protein_coding
AABR070	-1.6309	-3.097	0.01185	0.0441	1	ENSRNOG	protein_coding
Il17f	-3.4663	-11.052	0.00161	0.00911	1	ENSRNOG	protein_coding
Gsta1	3.15039	8.87895	0.00076	0.005	1	ENSRNOG	protein_coding
LOC5011	2.97291	7.8512	9.5E-05	0.00097	1	ENSRNOG	protein_coding
Kcnq5	-1.8119	-3.511	3.9E-11	1.7E-09	7.5E-07	ENSRNOG	protein_coding
Lonrf2	-3.2596	-9.5772	2.1E-10	8.3E-09	4.1E-06	ENSRNOG	protein_coding
Il1r1	-1.322	-2.5002	1.3E-08	3.9E-07	0.00026	ENSRNOG	protein_coding
Il1rl2	-4.5024	-22.665	3.8E-14	2.6E-12	7.3E-10	ENSRNOG	protein_coding
Il1rl1	-3.6873	-12.882	9.7E-10	3.5E-08	1.9E-05	ENSRNOG	protein_coding
Ercc5	1.55834	2.94514	1.1E-05	0.00016	0.21435	ENSRNOG	protein_coding
AABR070	-1.4383	-2.71	0.01091	0.04121	1	ENSRNOG	pseudogene
Col3a1	1.92615	3.80038	4.2E-12	2.2E-10	8.2E-08	ENSRNOG	protein_coding
Slc40a1_	1.74065	3.34185	8.5E-08	2.1E-06	0.00164	ENSRNOG	protein_coding
Myo1b	-2.1753	-4.5167	1.9E-14	1.3E-12	3.6E-10	ENSRNOG	protein_coding
Sdpr	3.99756	15.9729	2.3E-20	2.8E-18	4.5E-16	ENSRNOG	protein_coding
AABR070	3.53951	11.6278	0.01099	0.04145	1	ENSRNOG	lincRNA
Rftn2	3.87698	14.6922	1.9E-11	8.7E-10	3.7E-07	ENSRNOG	protein_coding
Plcl1	2.37023	5.17023	1.3E-07	3.2E-06	0.00255	ENSRNOG	protein_coding
Aox1	3.74283	13.3877	6.3E-19	6.9E-17	1.2E-14	ENSRNOG	protein_coding
Clk1	1.49427	2.81721	6.3E-06	9.8E-05	0.12194	ENSRNOG	protein_coding
Fam117b	1.47924	2.78801	1.2E-08	3.5E-07	0.00023	ENSRNOG	protein_coding
Ica1l	2.10302	4.29609	0.00184	0.01018	1	ENSRNOG	protein_coding
Zdbf2	2.92703	7.60541	2.1E-15	1.6E-13	3.9E-11	ENSRNOG	protein_coding
Mdh1b	1.81374	3.51552	0.0016	0.00906	1	ENSRNOG	protein_coding
AC11131	-1.471	-2.7722	0.00309	0.01548	1	ENSRNOG	lincRNA
Ccnyl1	-1.3374	-2.527	1.1E-05	0.00015	0.20225	ENSRNOG	protein_coding
Plekhm3	1.53394	2.89575	2E-06	3.7E-05	0.03919	ENSRNOG	protein_coding
Idh1	1.94956	3.86256	7.9E-19	8.5E-17	1.5E-14	ENSRNOG	protein_coding
Pth2r	6.29915	78.7467	1.5E-17	1.4E-15	2.8E-13	ENSRNOG	protein_coding
Bard1	-1.3687	-2.5824	9.5E-05	0.00097	1	ENSRNOG	protein_coding
AABR070	2.73304	6.64857	0.00024	0.00201	1	ENSRNOG	lincRNA
Tmem16	2.75614	6.75585	2.2E-09	7.3E-08	4.1E-05	ENSRNOG	protein_coding
Tns1	3.31638	9.96161	4.4E-24	6.8E-22	8.5E-20	ENSRNOG	protein_coding
Pnkd	1.33966	2.53091	2.1E-07	4.7E-06	0.00396	ENSRNOG	protein_coding
Tmbim1	1.58637	3.00294	1.1E-10	4.6E-09	2.2E-06	ENSRNOG	protein_coding
Catip	3.77283	13.6689	1E-05	0.00015	0.19927	ENSRNOG	protein_coding
Slc11a1	3.02064	8.11529	0.0002	0.00174	1	ENSRNOG	protein_coding
Vil1	-3.3176	-9.9701	0.01064	0.04046	1	ENSRNOG	protein_coding
Plcd4	-3.5303	-11.554	9.9E-38	3.2E-35	1.9E-33	ENSRNOG	protein_coding
Cyp27a1	1.9143	3.76932	9.7E-11	4E-09	1.9E-06	ENSRNOG	protein_coding
Ihh	-2.7784	-6.8607	0.00024	0.00205	1	ENSRNOG	protein_coding
Tuba4a	-1.3257	-2.5066	0.00048	0.00351	1	ENSRNOG	protein_coding
Ptprn	-3.7471	-13.427	2.4E-23	3.5E-21	4.6E-19	ENSRNOG	protein_coding
Des	-1.8237	-3.5399	4.5E-09	1.5E-07	8.7E-05	ENSRNOG	protein_coding
Speg	1.52827	2.8844	0.0002	0.00173	1	ENSRNOG	protein_coding

Slc4a3	2.02254	4.06299	9.3E-08	2.3E-06	0.00179	ENSRNOG	protein_coding
Daw1	2.82913	7.10647	5.6E-09	1.8E-07	0.00011	ENSRNOG	protein_coding
LOC1009	-5.7969	-55.596	0.00376	0.01796	1	ENSRNOG	protein_coding
Dner	-1.4511	-2.7342	0.00011	0.00105	1	ENSRNOG	protein_coding
Fbxo36	4.26275	19.1963	0.00073	0.00483	1	ENSRNOG	protein_coding
Htr2b	3.60773	12.1909	4.4E-27	7.6E-25	8.4E-23	ENSRNOG	protein_coding
B3gnt7	-2.4688	-5.536	5.8E-09	1.8E-07	0.00011	ENSRNOG	protein_coding
Ncl	-1.4107	-2.6586	1.7E-05	0.00023	0.32937	ENSRNOG	protein_coding
Efhd1	-2.0144	-4.0401	1E-05	0.00014	0.19209	ENSRNOG	protein_coding
Mroh2a	2.25402	4.77009	2.3E-07	5.2E-06	0.00439	ENSRNOG	protein_coding
Hjurp	-1.7991	-3.4801	7.3E-06	0.00011	0.1398	ENSRNOG	protein_coding
AABR070	-1.8846	-3.6924	2.9E-06	4.9E-05	0.05524	ENSRNOG	pseudogene
Sh3bp4	-1.6878	-3.2217	1.2E-11	5.8E-10	2.3E-07	ENSRNOG	protein_coding
Espnl	-1.459	-2.7493	0.00228	0.01216	1	ENSRNOG	protein_coding
Erfe	-1.8824	-3.6869	0.00015	0.0014	1	ENSRNOG	protein_coding
Sned1	1.67293	3.18861	0.00267	0.01381	1	ENSRNOG	protein_coding
Bok	-1.6122	-3.0572	7.3E-11	3E-09	1.4E-06	ENSRNOG	protein_coding
Slco4c1	1.39321	2.62663	0.0082	0.0332	1	ENSRNOG	protein_coding
Ddx11	-2.2099	-4.6265	2.3E-08	6.5E-07	0.00045	ENSRNOG	protein_coding
Akain1	3.56017	11.7955	0.00186	0.01031	1	ENSRNOG	protein_coding
Ndc80	-1.3931	-2.6264	0.00066	0.00451	1	ENSRNOG	protein_coding
AABR070	1.79267	3.46456	0.01326	0.04819	1	ENSRNOG	protein_coding
Zfp300	3.09653	8.55358	3.1E-08	8.4E-07	0.0006	ENSRNOG	protein_coding
Spaca5	6.54014	93.0634	0.00645	0.02754	1	ENSRNOG	protein_coding
Zfp182	3.99255	15.9176	5.4E-10	2E-08	1E-05	ENSRNOG	protein_coding
Efhc2	-1.5348	-2.8975	0.0082	0.03319	1	ENSRNOG	protein_coding
Tspan7	-1.4482	-2.7287	1.9E-05	0.00024	0.35747	ENSRNOG	protein_coding
Lancl3	6.02415	65.0803	0.0111	0.04178	1	ENSRNOG	protein_coding
Suv39h1l	-1.5104	-2.8489	0.00016	0.00144	1	ENSRNOG	protein_coding
Hdac6	6.87789	117.612	0.00692	0.02916	1	ENSRNOG	protein_coding
LOC1083	5.64993	50.2109	0.00107	0.00656	1	ENSRNOG	protein_coding
AABR070	-3.9661	-15.628	3.5E-07	7.7E-06	0.00676	ENSRNOG	protein_coding
Wnk3	-2.1481	-4.4325	4.2E-06	6.9E-05	0.08072	ENSRNOG	protein_coding
Iqsec2	1.5657	2.96022	5.1E-07	1.1E-05	0.00988	ENSRNOG	protein_coding
Fancb	-1.4036	-2.6456	9.1E-05	0.00093	1	ENSRNOG	protein_coding
Vegfd	5.42854	43.068	5.5E-32	1.2E-29	1.1E-27	ENSRNOG	protein_coding
Grpr	3.77824	13.7203	6.8E-24	1E-21	1.3E-19	ENSRNOG	protein_coding
Reps2	2.02887	4.08084	0.00632	0.02713	1	ENSRNOG	protein_coding
Scml2	-1.5373	-2.9025	0.00154	0.00882	1	ENSRNOG	protein_coding
Adgrg2	5.49689	45.1573	1.7E-10	6.7E-09	3.2E-06	ENSRNOG	protein_coding
LOC1003	-5.6867	-51.506	0.00446	0.02052	1	ENSRNOG	protein_coding
Pola1	-1.7068	-3.2644	2.4E-07	5.5E-06	0.00469	ENSRNOG	protein_coding
Pcyt1b	2.35857	5.1286	1.5E-07	3.7E-06	0.00297	ENSRNOG	protein_coding
Zc3h12b	1.54454	2.9171	0.0031	0.01548	1	ENSRNOG	protein_coding
Stard8	1.46469	2.76004	5.1E-06	8.1E-05	0.09736	ENSRNOG	protein_coding
P2ry4	2.43754	5.41716	0.00276	0.01421	1	ENSRNOG	protein_coding
Arr3	2.88063	7.36472	0.00723	0.03015	1	ENSRNOG	protein_coding
Rn50_X_(	1.53733	2.90257	0.00885	0.03518	1	ENSRNOG	antisense_RNA
Kif4a	-1.5137	-2.8554	0.00052	0.00372	1	ENSRNOG	protein_coding

Nlgn3	3.10644	8.61252	3E-06	5.2E-05	0.05858	ENSRNOG	protein_coding
Cxcr3	-2.7156	-6.5685	3.2E-06	5.4E-05	0.06153	ENSRNOG	protein_coding
Ercc6l	-1.8859	-3.6958	9.8E-05	0.00099	1	ENSRNOG	protein_coding
RGD1561	1.8129	3.51347	4.2E-15	3.2E-13	8E-11	ENSRNOG	protein_coding
RGD1560	-1.8569	-3.6223	2.8E-06	4.8E-05	0.05347	ENSRNOG	protein_coding
Gpr174	3.37814	10.3973	0.003	0.01515	1	ENSRNOG	protein_coding
Dach2	2.35921	5.13088	0.00477	0.02158	1	ENSRNOG	protein_coding
Nox1	3.80757	14.0021	4.9E-09	1.6E-07	9.3E-05	ENSRNOG	protein_coding
Xkrx	2.1443	4.42077	5.9E-05	0.00066	1	ENSRNOG	protein_coding
Tmem35	2.85846	7.25238	1.7E-09	5.8E-08	3.3E-05	ENSRNOG	protein_coding
Cenpi	-1.4013	-2.6415	0.00022	0.0019	1	ENSRNOG	protein_coding
Drp2	2.33285	5.03799	0.00023	0.00199	1	ENSRNOG	protein_coding
Bhlhb9	-1.8381	-3.5753	0.00374	0.01789	1	ENSRNOG	protein_coding
Tceal1	1.63849	3.11341	0.00035	0.00275	1	ENSRNOG	protein_coding
Tsc22d3	4.33957	20.2461	2.2E-52	1.7E-49	4.3E-48	ENSRNOG	protein_coding
AABR070	4.53272	23.1464	1E-17	1E-15	2E-13	ENSRNOG	protein_coding
Atg4a	1.97784	3.93904	2.2E-18	2.3E-16	4.2E-14	ENSRNOG	protein_coding
Col4a6	2.1639	4.48123	2E-06	3.6E-05	0.0389	ENSRNOG	protein_coding
Tmem164	2.41735	5.34188	7.8E-17	7.1E-15	1.5E-12	ENSRNOG	protein_coding
Chrdl1	5.42743	43.0346	3.6E-07	7.8E-06	0.00683	ENSRNOG	protein_coding
Lhfpl1	2.80757	7.00104	0.00991	0.03841	1	ENSRNOG	protein_coding
Pls3	1.64481	3.12707	6.5E-11	2.7E-09	1.3E-06	ENSRNOG	protein_coding
AABR070	-2.6226	-6.1584	0.0084	0.03384	1	ENSRNOG	pseudogene
Akap17b	2.3833	5.21728	1.1E-09	3.9E-08	2.1E-05	ENSRNOG	protein_coding
Apln	-4.0267	-16.299	8.5E-12	4.2E-10	1.6E-07	ENSRNOG	protein_coding
Rbmx2	-1.4032	-2.6448	3.9E-05	0.00046	0.75025	ENSRNOG	protein_coding
Ccdc160	4.304	19.753	4.3E-05	0.0005	0.83552	ENSRNOG	protein_coding
Nsdhl	1.36808	2.58127	0.002	0.01092	1	ENSRNOG	protein_coding
Gdi1	1.35918	2.56539	0.00027	0.00223	1	ENSRNOG	protein_coding
LOC6860	1.68502	3.21545	0.0116	0.04332	1	ENSRNOG	protein_coding
LOC1036	-1.6143	-3.0617	0.00448	0.0206	1	ENSRNOG	protein_coding
LOC1025	-2.9279	-7.61	0.00564	0.02475	1	ENSRNOG	protein_coding

Table S4. Differentially expressed genes in KMM cells treated with desamethasone

Cutoffs for differentially expressed genes: Fold change>2.5, P-value<0.05, FDR<0.05

Name	Log_2 fold change	Fold change	P-value	FDR p-value	Bonferroni	ENSEMBL	Biotype
Polr2l	-8.9213	-484.81	8.6E-14	1.1E-12	1.73E-09	ENSRNO(	protein_coding
Rfxapl1	-8.7284	-424.15	4.9E-06	2.5E-05	0.098339	ENSRNO(	protein_coding
LOC1083	-7.4399	-173.63	1E-04	0.00042	1	ENSRNO(	protein_coding
Srm	-7.2887	-156.36	0.00014	0.00057	1	ENSRNO(	protein_coding
AABR070	-7.2362	-150.77	8.4E-05	0.00036	1	ENSRNO(	protein_coding
Snrpd2l	-6.8497	-115.34	0.00032	0.00124	1	ENSRNO(	protein_coding
LOC1036	-6.5097	-91.122	0.00066	0.00238	1	ENSRNO(	protein_coding
LOC1009	-6.4777	-89.123	7.3E-11	6.8E-10	1.46E-06	ENSRNO(	protein_coding
Cldn4	-6.402	-84.567	5.4E-08	3.6E-07	0.001088	ENSRNO(	protein_coding
AABR070	-6.1865	-72.832	0.00141	0.00476	1	ENSRNO(	protein_coding
Crnn	-6.0539	-66.436	4E-188	7E-185	8.2E-184	ENSRNO(	protein_coding
Chrdl2	-5.7856	-55.161	0.00125	0.00427	1	ENSRNO(	protein_coding
Ptpn5	-5.685	-51.445	0.00166	0.00552	1	ENSRNO(	protein_coding
Mmp9	-5.3513	-40.823	1.4E-34	7.2E-33	2.91E-30	ENSRNO(	protein_coding
Majin	-5.3337	-40.326	6.5E-08	4.3E-07	0.001316	ENSRNO(	protein_coding
Ovol2	-5.1978	-36.702	0.00349	0.01088	1	ENSRNO(	protein_coding
Pax5	-5.1848	-36.374	0.00413	0.01266	1	ENSRNO(	protein_coding
LOC1036	-5.1475	-35.444	4.8E-05	0.00021	0.965368	ENSRNO(	protein_coding
AC118841	-5.0703	-33.599	0.00643	0.01884	1	ENSRNO(	protein_coding
St6galnac	-5.0382	-32.858	8.9E-63	1.1E-60	1.79E-58	ENSRNO(	protein_coding
AC099294	-4.9787	-31.532	0.00696	0.02017	1	ENSRNO(	protein_coding
ENSRNO	-4.9658	-31.25	0.00827	0.02346	1	ENSRNO(	protein_coding
Scube1	-4.8324	-28.49	4.1E-13	4.8E-12	8.2E-09	ENSRNO(	protein_coding
Fibcd1	-4.8312	-28.468	0.00799	0.02279	1	ENSRNO(	protein_coding
Wdr88	-4.8311	-28.464	0.00694	0.02012	1	ENSRNO(	protein_coding
Lmx1a	-4.8219	-28.284	0.00813	0.02311	1	ENSRNO(	protein_coding
LOC1003	-4.8171	-28.19	0.00874	0.02458	1	ENSRNO(	protein_coding
Krtap3-2	-4.71	-26.173	0.01121	0.03069	1	ENSRNO(	protein_coding
Slco2a1	-4.6997	-25.986	1.5E-40	9.6E-39	3.07E-36	ENSRNO(	protein_coding
Ccl27	-4.6805	-25.642	0.00987	0.02742	1	ENSRNO(	protein_coding
AABR070	-4.6748	-25.542	0.01706	0.04463	1	ENSRNO(	pseudogene
LOC1083	-4.659	-25.264	0.01306	0.03523	1	ENSRNO(	protein_coding
Aldob	-4.6531	-25.161	1.4E-07	8.5E-07	0.002719	ENSRNO(	protein_coding
Lat2	-4.6526	-25.153	6.9E-06	3.4E-05	0.138735	ENSRNO(	protein_coding
Il1a	-4.6356	-24.858	2.9E-15	4.2E-14	5.91E-11	ENSRNO(	protein_coding
Mt1m	-4.5749	-23.833	7.1E-05	0.00031	1	ENSRNO(	protein_coding
LOC1025	-4.5433	-23.316	0.01536	0.04065	1	ENSRNO(	protein_coding
LOC1036	-4.5219	-22.973	0.01168	0.03186	1	ENSRNO(	lincRNA
Hes3	-4.5204	-22.949	0.0002	0.00078	1	ENSRNO(	protein_coding
Tmem163	-4.4484	-21.832	3.7E-07	2.2E-06	0.007445	ENSRNO(	protein_coding
Nppb	-4.4439	-21.765	0.00028	0.00108	1	ENSRNO(	protein_coding
Ido2	-4.3775	-20.786	3.7E-30	1.5E-28	7.42E-26	ENSRNO(	protein_coding
Tns4	-4.3705	-20.685	3.6E-13	4.3E-12	7.26E-09	ENSRNO(	protein_coding
Spib	-4.3632	-20.581	0.0003	0.00115	1	ENSRNO(	protein_coding

Trpm2	-4.3604	-20.54	2.3E-13	2.8E-12	4.65E-09	ENSRNO(	protein_coding
Apln	-4.3417	-20.275	9.7E-15	1.3E-13	1.95E-10	ENSRNO(	protein_coding
Ppp1r3g	-4.3382	-20.227	0.01595	0.04201	1	ENSRNO(	protein_coding
Ccl17	-4.3382	-20.226	0.01551	0.04099	1	ENSRNO(	protein_coding
Ttc29	-4.3382	-20.226	0.01553	0.04103	1	ENSRNO(	protein_coding
Krt26	-4.3282	-20.087	1.2E-68	1.7E-66	2.49E-64	ENSRNO(	protein_coding
Upb1	-4.2938	-19.614	9.4E-07	5.3E-06	0.018844	ENSRNO(	protein_coding
AABR070	-4.2882	-19.538	0.00144	0.00487	1	ENSRNO(	lincRNA
Glb1l3	-4.2776	-19.395	0.00038	0.00142	1	ENSRNO(	protein_coding
Wnt7b	-4.2025	-18.411	3.7E-05	0.00016	0.735346	ENSRNO(	protein_coding
Ccl2	-4.2022	-18.407	1.8E-28	6.4E-27	3.53E-24	ENSRNO(	protein_coding
Ubald1	-4.1681	-17.977	0.00032	0.00124	1	ENSRNO(	protein_coding
Fam83f	-4.1659	-17.95	1.9E-06	1E-05	0.03852	ENSRNO(	protein_coding
Fam19a2	-4.1205	-17.393	5.8E-06	2.9E-05	0.116685	ENSRNO(	protein_coding
Ccl7	-4.1199	-17.386	2.7E-08	1.9E-07	0.000551	ENSRNO(	protein_coding
Has2	-4.1136	-17.31	6.7E-13	7.8E-12	1.35E-08	ENSRNO(	protein_coding
Hcar2	-4.1099	-17.267	2E-17	3.3E-16	3.94E-13	ENSRNO(	protein_coding
Sh2d7	-4.1086	-17.251	1.6E-05	7.4E-05	0.314431	ENSRNO(	protein_coding
Ngfr	-4.0671	-16.761	0.00233	0.00755	1	ENSRNO(	protein_coding
Sfrp2	-4.0636	-16.721	7.7E-14	9.7E-13	1.55E-09	ENSRNO(	protein_coding
F2rl2	-4.0409	-16.46	1.8E-08	1.3E-07	0.000372	ENSRNO(	protein_coding
Prl7a3	-4.0136	-16.151	8.3E-08	5.3E-07	0.001671	ENSRNO(	protein_coding
Klk14	-4.0134	-16.149	4.1E-11	3.9E-10	8.16E-07	ENSRNO(	protein_coding
Krt14	-3.9886	-15.874	2.1E-14	2.8E-13	4.32E-10	ENSRNO(	protein_coding
Shc2	-3.9855	-15.84	0.00125	0.00427	1	ENSRNO(	protein_coding
Hnf4a	-3.9811	-15.792	1.5E-09	1.2E-08	2.96E-05	ENSRNO(	protein_coding
B4galnt3	-3.8921	-14.847	5.4E-14	6.9E-13	1.08E-09	ENSRNO(	protein_coding
Cd300lb	-3.875	-14.672	1.7E-08	1.2E-07	0.00035	ENSRNO(	protein_coding
Ndp	-3.8711	-14.633	0.00018	0.00072	1	ENSRNO(	protein_coding
Krt16	-3.8684	-14.605	1.7E-09	1.3E-08	3.34E-05	ENSRNO(	protein_coding
Evi2a	-3.8588	-14.508	1.9E-70	2.9E-68	3.83E-66	ENSRNO(	protein_coding
Il1rl1	-3.8108	-14.033	8.4E-64	1.1E-61	1.69E-59	ENSRNO(	protein_coding
Igfbp4	-3.7998	-13.927	1E-139	7E-137	2.4E-135	ENSRNO(	protein_coding
Rn60_10_	-3.7977	-13.907	3.4E-09	2.7E-08	6.91E-05	ENSRNO(	unprocessed_pseudogene
Prom2	-3.7614	-13.561	7.6E-30	3E-28	1.54E-25	ENSRNO(	protein_coding
Ecel1	-3.7517	-13.47	0.00295	0.00933	1	ENSRNO(	protein_coding
Adamts8	-3.6994	-12.99	3E-121	1E-118	5.2E-117	ENSRNO(	protein_coding
Gck	-3.6888	-12.896	0.00014	0.00058	1	ENSRNO(	protein_coding
Adgrg1	-3.6283	-12.366	0.00032	0.00122	1	ENSRNO(	protein_coding
Prl5a1	-3.6021	-12.144	7.3E-07	4.1E-06	0.014609	ENSRNO(	protein_coding
Plvap	-3.5373	-11.61	0.00063	0.0023	1	ENSRNO(	protein_coding
Fjx1	-3.5296	-11.548	8.5E-08	5.5E-07	0.001713	ENSRNO(	protein_coding
Gpr68	-3.5191	-11.464	8.8E-11	8.1E-10	1.77E-06	ENSRNO(	protein_coding
Traf3ip3	-3.4976	-11.295	0.00012	0.00051	1	ENSRNO(	protein_coding
LOC1025	-3.4947	-11.272	0.01222	0.03314	1	ENSRNO(	protein_coding
AABR070	-3.4919	-11.25	0.01079	0.02969	1	ENSRNO(	protein_coding
Krt79	-3.4742	-11.113	8.7E-47	6.8E-45	1.75E-42	ENSRNO(	protein_coding
Hpca	-3.4725	-11.1	0.00651	0.01905	1	ENSRNO(	protein_coding
Lce1f	-3.4724	-11.1	0.00687	0.01996	1	ENSRNO(	protein_coding

Fam167a	-3.468	-11.066	1.4E-23	3.7E-22	2.81E-19	ENSRNO0	protein_coding
Slc47a1	-3.4605	-11.008	1.3E-37	7.6E-36	2.66E-33	ENSRNO0	protein_coding
Tnc	-3.4491	-10.922	2E-108	6E-106	3.7E-104	ENSRNO0	protein_coding
Pex11g	-3.4462	-10.9	0.00206	0.00674	1	ENSRNO0	protein_coding
Ccdc94_1	-3.4228	-10.724	6.3E-10	5.3E-09	1.28E-05	ENSRNO0	protein_coding
AABR070	-3.4038	-10.584	5.6E-13	6.5E-12	1.12E-08	ENSRNO0	protein_coding
AABR070	-3.393	-10.505	3.7E-06	1.9E-05	0.075196	ENSRNO0	lincRNA
Haus1	-3.3429	-10.147	2.3E-06	1.2E-05	0.046127	ENSRNO0	protein_coding
Atp13a4	-3.338	-10.112	2.6E-79	4.8E-77	5.18E-75	ENSRNO0	protein_coding
Serpinb13	-3.3305	-10.06	0.00299	0.00946	1	ENSRNO0	protein_coding
Krtap3-1	-3.3298	-10.055	0.00312	0.00983	1	ENSRNO0	protein_coding
Aldh18a1	-3.3224	-10.003	0.00043	0.00162	1	ENSRNO0	protein_coding
Chi3l1	-3.3096	-9.9146	0.00996	0.02763	1	ENSRNO0	protein_coding
Pou4f2	-3.2996	-9.8464	0.01525	0.04039	1	ENSRNO0	protein_coding
Pcsk5	-3.2979	-9.8347	1.2E-63	1.5E-61	2.39E-59	ENSRNO0	protein_coding
AABR070	-3.2974	-9.8314	5.7E-20	1.2E-18	1.15E-15	ENSRNO0	protein_coding
Ifitm10	-3.2935	-9.8049	4E-21	8.9E-20	7.97E-17	ENSRNO0	protein_coding
Pla2g2a	-3.2922	-9.7963	8.7E-30	3.4E-28	1.75E-25	ENSRNO0	protein_coding
Nrap	-3.2642	-9.6077	0.0007	0.00252	1	ENSRNO0	protein_coding
Stac	-3.2472	-9.495	2.9E-09	2.2E-08	5.77E-05	ENSRNO0	protein_coding
Krt78	-3.2271	-9.3636	0.0038	0.01175	1	ENSRNO0	protein_coding
Ccl4	-3.224	-9.3437	0.0027	0.00862	1	ENSRNO0	protein_coding
Rn60_20_	-3.224	-9.3437	0.003	0.00949	1	ENSRNO0	antisense_RNA
Cxcl3	-3.2192	-9.313	1.8E-10	1.6E-09	3.68E-06	ENSRNO0	protein_coding
Klk6	-3.2171	-9.299	0.00447	0.01359	1	ENSRNO0	protein_coding
Postn	-3.2056	-9.2252	1.5E-85	3.2E-83	3.01E-81	ENSRNO0	protein_coding
AABR070	-3.2027	-9.207	0.00212	0.00692	1	ENSRNO0	protein_coding
Knop1_1	-3.1978	-9.1753	0.0002	0.00078	1	ENSRNO0	protein_coding
Crabp2_1	-3.1946	-9.155	7.2E-06	3.6E-05	0.14421	ENSRNO0	protein_coding
Unc13c	-3.1892	-9.121	2.1E-18	3.8E-17	4.21E-14	ENSRNO0	protein_coding
Pla2g3	-3.1864	-9.1034	0.00136	0.00462	1	ENSRNO0	protein_coding
RGD1561	-3.1814	-9.0721	1.9E-22	4.7E-21	3.88E-18	ENSRNO0	protein_coding
Nat8l	-3.1802	-9.0646	6.2E-10	5.2E-09	1.25E-05	ENSRNO0	protein_coding
LOC1009	-3.1464	-8.8543	0.00104	0.0036	1	ENSRNO0	protein_coding
Usp51	-3.1259	-8.7297	1.6E-09	1.2E-08	3.12E-05	ENSRNO0	protein_coding
LOC1025	-3.1259	-8.7293	0.01353	0.03633	1	ENSRNO0	protein_coding
AABR070	-3.1198	-8.6925	1.2E-11	1.2E-10	2.45E-07	ENSRNO0	lincRNA
Mmd	-3.1094	-8.6301	1E-110	5E-108	2.8E-106	ENSRNO0	protein_coding
Sema6d	-3.1024	-8.5882	0.00458	0.0139	1	ENSRNO0	protein_coding
Dlgap3	-3.1023	-8.5879	0.00377	0.01165	1	ENSRNO0	protein_coding
Syt2	-3.101	-8.5804	0.00552	0.01645	1	ENSRNO0	protein_coding
LOC4979	-3.0869	-8.4969	1E-17	1.7E-16	2.04E-13	ENSRNO0	protein_coding
Parvb	-3.0854	-8.4881	5.8E-11	5.5E-10	1.17E-06	ENSRNO0	protein_coding
Cyp24a1	-3.0818	-8.4665	2.5E-20	5.3E-19	5.06E-16	ENSRNO0	protein_coding
Vil1	-3.0624	-8.3535	1.5E-05	7.2E-05	0.305872	ENSRNO0	protein_coding
Capn8	-3.0496	-8.2796	5E-85	1E-82	1.01E-80	ENSRNO0	protein_coding
Dhrs2	-3.0367	-8.2062	0.00014	0.00055	1	ENSRNO0	protein_coding
Grip2	-3.0236	-8.1321	5.9E-05	0.00026	1	ENSRNO0	protein_coding
Cyp3a9_2	-3.0229	-8.1278	3.1E-16	4.9E-15	6.34E-12	ENSRNO0	protein_coding

Mmp2	-3.0208	-8.1159	0.00098	0.00342	1	ENSRNO(	protein_coding
Kank4	-3.0172	-8.0961	0.00036	0.00138	1	ENSRNO(	protein_coding
Sema7a	-3.0143	-8.0798	2.7E-37	1.5E-35	5.38E-33	ENSRNO(	protein_coding
Fcer2	-3.0082	-8.0457	0.00052	0.0019	1	ENSRNO(	protein_coding
Asb2	-3.0082	-8.0457	0.00079	0.00282	1	ENSRNO(	protein_coding
Lhfpl2	-3.0003	-8.0017	1.5E-50	1.3E-48	2.95E-46	ENSRNO(	protein_coding
Mettl7b	-2.994	-7.9668	0.00145	0.0049	1	ENSRNO(	protein_coding
LOC6853	-2.994	-7.9667	0.00135	0.0046	1	ENSRNO(	protein_coding
Kcnq5	-2.9807	-7.8938	1.6E-84	3.3E-82	3.28E-80	ENSRNO(	protein_coding
Angptl2	-2.9797	-7.888	2.1E-46	1.6E-44	4.3E-42	ENSRNO(	protein_coding
Ccl9	-2.9695	-7.8325	0.0062	0.01824	1	ENSRNO(	protein_coding
Cym	-2.9677	-7.823	8.8E-05	0.00037	1	ENSRNO(	protein_coding
Des	-2.9631	-7.7981	1.4E-24	4.1E-23	2.88E-20	ENSRNO(	protein_coding
Cxcl11	-2.9619	-7.7916	1.1E-07	6.8E-07	0.00216	ENSRNO(	protein_coding
Jam2	-2.9528	-7.7423	2.9E-11	2.8E-10	5.79E-07	ENSRNO(	protein_coding
Mab21l3	-2.9502	-7.7287	2E-10	1.8E-09	3.99E-06	ENSRNO(	protein_coding
Nptxr	-2.9445	-7.6981	1.4E-92	3.6E-90	2.84E-88	ENSRNO(	protein_coding
LOC1025	-2.9274	-7.6074	0.00106	0.00369	1	ENSRNO(	lincRNA
Itga2	-2.8985	-7.4567	4.9E-07	2.8E-06	0.009809	ENSRNO(	protein_coding
Rab3b	-2.8944	-7.4351	3.8E-08	2.5E-07	0.000757	ENSRNO(	protein_coding
Fam102a	-2.8878	-7.4017	2E-223	5E-220	3.4E-219	ENSRNO(	protein_coding
Itgb6	-2.8822	-7.3728	0.00034	0.00129	1	ENSRNO(	protein_coding
Mal	-2.8814	-7.3688	2.3E-08	1.6E-07	0.000467	ENSRNO(	protein_coding
Dhrs9	-2.8741	-7.3316	2E-65	2.6E-63	4.08E-61	ENSRNO(	protein_coding
Ccdc24	-2.8493	-7.2065	0.00028	0.00109	1	ENSRNO(	protein_coding
Ccl22	-2.8485	-7.2027	0.00173	0.00575	1	ENSRNO(	protein_coding
Tnip3	-2.8478	-7.1989	3.2E-52	3E-50	6.43E-48	ENSRNO(	protein_coding
LOC1009	-2.8355	-7.1378	0.01072	0.02952	1	ENSRNO(	protein_coding
Vom2r60	-2.8231	-7.0766	0.0119	0.03235	1	ENSRNO(	protein_coding
Csf2	-2.823	-7.0765	0.00796	0.0227	1	ENSRNO(	protein_coding
Gpbar1	-2.823	-7.0764	0.01092	0.03	1	ENSRNO(	protein_coding
Acan	-2.8213	-7.0679	4.5E-19	8.4E-18	8.97E-15	ENSRNO(	protein_coding
LOC1025	-2.8113	-7.0194	0.00148	0.00499	1	ENSRNO(	protein_coding
Kcnh6	-2.8088	-7.007	5.1E-47	4E-45	1.02E-42	ENSRNO(	protein_coding
Sez6	-2.8023	-6.9756	0.01804	0.04683	1	ENSRNO(	protein_coding
Wnt7a	-2.7969	-6.9494	1.5E-05	7.2E-05	0.306346	ENSRNO(	protein_coding
Prkg2	-2.7947	-6.9388	1E-34	5E-33	2.01E-30	ENSRNO(	protein_coding
R3hdml	-2.7778	-6.8582	0.00398	0.01223	1	ENSRNO(	protein_coding
Cylc2	-2.7777	-6.8576	0.00286	0.00909	1	ENSRNO(	protein_coding
AABR070	-2.7776	-6.8573	0.00491	0.01477	1	ENSRNO(	pseudogene
Sh2d1b2	-2.7572	-6.7606	5.9E-14	7.6E-13	1.19E-09	ENSRNO(	protein_coding
F13a1	-2.7526	-6.7394	8.5E-43	5.8E-41	1.72E-38	ENSRNO(	protein_coding
Ptafr	-2.7508	-6.7308	0.00283	0.00898	1	ENSRNO(	protein_coding
Nes	-2.7457	-6.707	4E-99	1.2E-96	8.24E-95	ENSRNO(	protein_coding
Milr1	-2.7456	-6.7065	0.00012	0.0005	1	ENSRNO(	protein_coding
Cacnb1	-2.7329	-6.6481	0.00074	0.00265	1	ENSRNO(	protein_coding
LOC5006	-2.7324	-6.6455	2.9E-39	1.8E-37	5.88E-35	ENSRNO(	protein_coding
Padi2	-2.7198	-6.588	3.6E-05	0.00016	0.722853	ENSRNO(	protein_coding
Samd5	-2.7168	-6.5743	2.5E-25	7.7E-24	5.1E-21	ENSRNO(	protein_coding

Gcnt3	-2.7147	-6.5644	5.2E-07	3E-06	0.010378	ENSRNO(	protein_coding
Dmtn	-2.6975	-6.4869	4.7E-05	0.00021	0.955166	ENSRNO(	protein_coding
Scube3	-2.6848	-6.4297	1.6E-11	1.6E-10	3.15E-07	ENSRNO(	protein_coding
Plcd4	-2.6703	-6.3655	1.4E-77	2.6E-75	2.86E-73	ENSRNO(	protein_coding
Ercc1	-2.6697	-6.363	1E-123	6E-121	2.8E-119	ENSRNO(	protein_coding
Slc4a11	-2.666	-6.3465	1E-26	3.4E-25	2.02E-22	ENSRNO(	protein_coding
AABR070	-2.656	-6.3029	0.00769	0.02205	1	ENSRNO(	lincRNA
AABR070	-2.6554	-6.3	0.00047	0.00175	1	ENSRNO(	lincRNA
Ngf	-2.6548	-6.2975	3.9E-31	1.7E-29	7.95E-27	ENSRNO(	protein_coding
Slurp1	-2.6496	-6.275	0.00016	0.00066	1	ENSRNO(	protein_coding
AABR070	-2.6464	-6.2612	2.4E-05	0.00011	0.478248	ENSRNO(	lincRNA
Gsta5	-2.6407	-6.2363	0.00229	0.00743	1	ENSRNO(	protein_coding
Arhgap22	-2.6238	-6.1636	2.5E-49	2.2E-47	5.09E-45	ENSRNO(	protein_coding
Myo5c	-2.6204	-6.1493	9.1E-06	4.5E-05	0.182574	ENSRNO(	protein_coding
Gpd1	-2.6121	-6.1139	1E-17	1.8E-16	2.09E-13	ENSRNO(	protein_coding
Hmga1	-2.608	-6.0968	1.2E-68	1.7E-66	2.48E-64	ENSRNO(	protein_coding
AABR070	-2.5985	-6.0567	1.8E-11	1.8E-10	3.62E-07	ENSRNO(	lincRNA
Myb	-2.5887	-6.0158	9.5E-13	1.1E-11	1.91E-08	ENSRNO(	protein_coding
Tnf	-2.5815	-5.9856	0.00932	0.02607	1	ENSRNO(	protein_coding
AABR070	-2.576	-5.9627	0.00175	0.00581	1	ENSRNO(	lincRNA
Fat2	-2.5498	-5.8554	0.0039	0.01201	1	ENSRNO(	protein_coding
LOC1009	-2.5474	-5.8456	0.00017	0.00068	1	ENSRNO(	protein_coding
Adamtsl2	-2.5445	-5.834	2.7E-10	2.4E-09	5.52E-06	ENSRNO(	protein_coding
Lexm	-2.5308	-5.7789	9E-05	0.00038	1	ENSRNO(	protein_coding
Iqsec3	-2.5289	-5.7714	4.9E-19	9.2E-18	9.91E-15	ENSRNO(	protein_coding
Cnga4	-2.5232	-5.7487	0.01158	0.03162	1	ENSRNO(	protein_coding
Slc51b	-2.5211	-5.74	0.00255	0.00816	1	ENSRNO(	protein_coding
Svop	-2.5206	-5.738	0.01882	0.0486	1	ENSRNO(	protein_coding
Sgca	-2.5075	-5.6865	4.5E-15	6.2E-14	8.99E-11	ENSRNO(	protein_coding
Mmp1	-2.5072	-5.6852	0.00071	0.00253	1	ENSRNO(	protein_coding
Creb3l3	-2.5071	-5.6849	0.01106	0.03033	1	ENSRNO(	protein_coding
Lama3	-2.4877	-5.6088	4.3E-64	5.5E-62	8.68E-60	ENSRNO(	protein_coding
Ccbe1	-2.4814	-5.5844	9.1E-24	2.5E-22	1.83E-19	ENSRNO(	protein_coding
Mlnr	-2.4773	-5.5685	0.00026	0.00102	1	ENSRNO(	protein_coding
Acod1	-2.4757	-5.5624	0.00316	0.00995	1	ENSRNO(	protein_coding
Gnat2	-2.4466	-5.4512	1.1E-06	5.9E-06	0.021155	ENSRNO(	protein_coding
Adgrg3	-2.4375	-5.417	0.00539	0.01607	1	ENSRNO(	protein_coding
Cx3cl1	-2.431	-5.3927	4.3E-07	2.5E-06	0.008622	ENSRNO(	protein_coding
Sema4a	-2.4286	-5.3839	0.00011	0.00046	1	ENSRNO(	protein_coding
Crisp3	-2.4282	-5.3824	0.00539	0.01607	1	ENSRNO(	protein_coding
Olfm1	-2.4251	-5.3707	1.4E-52	1.4E-50	2.87E-48	ENSRNO(	protein_coding
Fer1l4	-2.4211	-5.3559	0.01586	0.04181	1	ENSRNO(	protein_coding
Mamdc2	-2.4196	-5.3502	0.002	0.00655	1	ENSRNO(	protein_coding
Dok2	-2.4134	-5.3271	4E-27	1.4E-25	8.1E-23	ENSRNO(	protein_coding
Tmprss7	-2.4115	-5.3203	0.00061	0.00222	1	ENSRNO(	protein_coding
RGD1566	-2.4115	-5.3202	0.00194	0.00637	1	ENSRNO(	protein_coding
Cuzd1	-2.3983	-5.2718	0.00924	0.02586	1	ENSRNO(	protein_coding
AABR070	-2.3848	-5.2228	0.0079	0.02259	1	ENSRNO(	lincRNA
Mthfd1l	-2.3809	-5.2088	1.4E-72	2.2E-70	2.79E-68	ENSRNO(	protein_coding

Krt17	-2.3805	-5.2073	0.00714	0.02063	1	ENSRNO(	protein_coding
Dmrtc1c1	-2.3794	-5.203	0.00157	0.00526	1	ENSRNO(	protein_coding
AABR070	-2.3783	-5.1993	1.9E-07	1.2E-06	0.003829	ENSRNO(	protein_coding
Gpa33	-2.3768	-5.194	0.01441	0.0384	1	ENSRNO(	protein_coding
Wdr62	-2.3744	-5.1854	0.01548	0.04091	1	ENSRNO(	protein_coding
AABR070	-2.3668	-5.1579	0.00022	0.00086	1	ENSRNO(	protein_coding
Itln1	-2.3465	-5.0858	0.00102	0.00353	1	ENSRNO(	protein_coding
Fas	-2.3372	-5.0531	0.00559	0.01663	1	ENSRNO(	protein_coding
Prss22	-2.3356	-5.0478	0.00253	0.00813	1	ENSRNO(	protein_coding
Slc1a5	-2.335	-5.0454	8E-84	1.6E-81	1.61E-79	ENSRNO(	protein_coding
Ly49i5	-2.3292	-5.0253	1.8E-12	2E-11	3.72E-08	ENSRNO(	protein_coding
Cd82	-2.3286	-5.0232	1.4E-76	2.5E-74	2.87E-72	ENSRNO(	protein_coding
Fosl1	-2.3266	-5.0164	1.7E-76	2.9E-74	3.36E-72	ENSRNO(	protein_coding
Pbp2	-2.3159	-4.9792	0.01137	0.03108	1	ENSRNO(	protein_coding
Erfe	-2.3127	-4.9682	1.3E-12	1.5E-11	2.61E-08	ENSRNO(	protein_coding
Grik4	-2.3109	-4.9619	0.00013	0.00055	1	ENSRNO(	protein_coding
St3gal4	-2.3003	-4.9256	1.3E-87	2.9E-85	2.54E-83	ENSRNO(	protein_coding
Ccl20	-2.2989	-4.9209	0.00048	0.00176	1	ENSRNO(	protein_coding
Cd53	-2.2948	-4.907	0.00074	0.00263	1	ENSRNO(	protein_coding
Wnt5a	-2.2919	-4.897	1.9E-16	3.1E-15	3.91E-12	ENSRNO(	protein_coding
Hk3	-2.2877	-4.8828	8.4E-21	1.8E-19	1.69E-16	ENSRNO(	protein_coding
Sema6a	-2.2704	-4.8247	4.7E-37	2.6E-35	9.53E-33	ENSRNO(	protein_coding
Cyp1b1	-2.2668	-4.8127	1.1E-44	7.8E-43	2.18E-40	ENSRNO(	protein_coding
Evi2b	-2.2668	-4.8126	2.9E-09	2.3E-08	5.83E-05	ENSRNO(	protein_coding
LOC1009	-2.2519	-4.763	6.8E-17	1.1E-15	1.37E-12	ENSRNO(	protein_coding
Fam46c	-2.2517	-4.7624	9.3E-09	6.8E-08	0.000187	ENSRNO(	protein_coding
Hcn4	-2.2517	-4.7623	0.00035	0.00133	1	ENSRNO(	protein_coding
Mmaa	-2.2514	-4.7613	2.1E-47	1.7E-45	4.19E-43	ENSRNO(	protein_coding
hist1h2ail	-2.2502	-4.7574	0.00646	0.01891	1	ENSRNO(	protein_coding
RGD1565	-2.2471	-4.7471	4.2E-08	2.8E-07	0.000839	ENSRNO(	processed_pseudogene
Vstm4	-2.2368	-4.7136	0.00048	0.00179	1	ENSRNO(	protein_coding
Cd83	-2.2225	-4.6672	0.00811	0.02308	1	ENSRNO(	protein_coding
Fam78b	-2.2143	-4.6407	2.5E-14	3.3E-13	5.08E-10	ENSRNO(	protein_coding
Neto2	-2.211	-4.6301	4.9E-38	2.9E-36	9.87E-34	ENSRNO(	protein_coding
Ly6g6c	-2.2046	-4.6094	0.0043	0.01313	1	ENSRNO(	protein_coding
Csf1r	-2.1998	-4.5941	5.5E-06	2.8E-05	0.110781	ENSRNO(	protein_coding
Irx4	-2.1987	-4.5905	6.1E-07	3.5E-06	0.012337	ENSRNO(	protein_coding
Arc	-2.1942	-4.5763	3.5E-11	3.4E-10	7.04E-07	ENSRNO(	protein_coding
AABR070	-2.1875	-4.5553	0.00147	0.00496	1	ENSRNO(	protein_coding
Lef1	-2.183	-4.5411	6.3E-32	2.8E-30	1.26E-27	ENSRNO(	protein_coding
Krt1	-2.1795	-4.5299	0.00946	0.0264	1	ENSRNO(	protein_coding
AABR070	-2.1775	-4.5236	1.4E-08	1E-07	0.000283	ENSRNO(	lincRNA
Pycr1	-2.1712	-4.5039	3E-36	1.6E-34	6.09E-32	ENSRNO(	protein_coding
Lhx6	-2.1622	-4.476	0.01062	0.02926	1	ENSRNO(	protein_coding
Ly49s6	-2.1598	-4.4686	6.9E-07	4E-06	0.013922	ENSRNO(	protein_coding
Siglec10	-2.1583	-4.4638	1E-06	5.6E-06	0.020259	ENSRNO(	protein_coding
Dync1i1	-2.1477	-4.4313	0.01364	0.03659	1	ENSRNO(	protein_coding
Dusp4	-2.1417	-4.4128	2.2E-41	1.4E-39	4.47E-37	ENSRNO(	protein_coding
Hspa8_1	-2.1411	-4.4111	4.6E-09	3.5E-08	9.26E-05	ENSRNO(	protein_coding

Slc17a4	-2.1406	-4.4095	0.00011	0.00044	1	ENSRNO(	protein_coding
Krt23	-2.1158	-4.3344	3.4E-07	2E-06	0.006891	ENSRNO(	protein_coding
Pcdh1	-2.1123	-4.3238	2.3E-60	2.7E-58	4.58E-56	ENSRNO(	protein_coding
Cxcl2	-2.1095	-4.3153	0.00335	0.01051	1	ENSRNO(	protein_coding
Car8	-2.0999	-4.2869	2.3E-05	0.00011	0.464035	ENSRNO(	protein_coding
Mybl2	-2.0966	-4.2771	1.3E-31	5.8E-30	2.68E-27	ENSRNO(	protein_coding
Dlx3	-2.0783	-4.2231	3.7E-05	0.00017	0.753347	ENSRNO(	protein_coding
Rasl12	-2.0707	-4.2009	0.00173	0.00573	1	ENSRNO(	protein_coding
AABR070	-2.0692	-4.1966	1.1E-05	5.3E-05	0.22185	ENSRNO(	pseudogene
ENSRNO	-2.0659	-4.187	5.2E-08	3.5E-07	0.001049	ENSRNO(	protein_coding
Neurl3	-2.0584	-4.1654	0.00033	0.00124	1	ENSRNO(	protein_coding
Klhl40	-2.0578	-4.1635	8.3E-10	6.9E-09	1.68E-05	ENSRNO(	protein_coding
AABR070	-2.0576	-4.1629	1.4E-06	7.8E-06	0.028794	ENSRNO(	lincRNA
AC12260:	-2.0514	-4.1451	0.00135	0.0046	1	ENSRNO(	pseudogene
Mybpc1	-2.0472	-4.133	0.00794	0.02267	1	ENSRNO(	protein_coding
Pmfbp1	-2.0462	-4.1301	0.01348	0.03623	1	ENSRNO(	protein_coding
Tmem13C	-2.0435	-4.1224	0.01015	0.02806	1	ENSRNO(	protein_coding
Cyp19a1	-2.0424	-4.1194	4.8E-22	1.1E-20	9.62E-18	ENSRNO(	protein_coding
LOC1003	-2.0412	-4.1158	0.01609	0.04234	1	ENSRNO(	protein_coding
RGD1562	-2.04	-4.1124	1.1E-69	1.7E-67	2.3E-65	ENSRNO(	protein_coding
Car3	-2.0379	-4.1065	2.1E-23	5.6E-22	4.29E-19	ENSRNO(	protein_coding
Sdc1	-2.0378	-4.1061	2.1E-49	1.8E-47	4.18E-45	ENSRNO(	protein_coding
Dusp5	-2.0315	-4.0883	3.4E-45	2.5E-43	6.88E-41	ENSRNO(	protein_coding
Il33	-2.0308	-4.0863	0.00413	0.01265	1	ENSRNO(	protein_coding
Akap12	-2.0222	-4.0622	7.2E-41	4.5E-39	1.44E-36	ENSRNO(	protein_coding
Lypd8	-2.0195	-4.0543	4.6E-05	0.0002	0.928577	ENSRNO(	protein_coding
Hpdl	-2.0194	-4.0541	1.2E-06	6.6E-06	0.023998	ENSRNO(	protein_coding
Gdf15	-2.0187	-4.0523	3.9E-16	6E-15	7.85E-12	ENSRNO(	protein_coding
Pgm5	-2.0173	-4.0481	0.00059	0.00215	1	ENSRNO(	protein_coding
Sgk2	-2.0115	-4.032	2.2E-08	1.6E-07	0.000453	ENSRNO(	protein_coding
Hspa8_2	-2.006	-4.0167	6.4E-07	3.7E-06	0.012944	ENSRNO(	protein_coding
Ptgs2	-2.0058	-4.0162	5.4E-55	5.5E-53	1.08E-50	ENSRNO(	protein_coding
Cxcl10	-2.0048	-4.0132	3.1E-45	2.3E-43	6.32E-41	ENSRNO(	protein_coding
Pmepa1	-2.0028	-4.0079	9.2E-53	8.8E-51	1.85E-48	ENSRNO(	protein_coding
Rbpjl	-1.9949	-3.9859	5.1E-06	2.6E-05	0.102136	ENSRNO(	protein_coding
LOC1009	-1.9945	-3.9847	0.00024	0.00093	1	ENSRNO(	protein_coding
Nefh	-1.9933	-3.9813	0.00357	0.0111	1	ENSRNO(	protein_coding
Hist1h3a	-1.9921	-3.9782	0.0021	0.00686	1	ENSRNO(	protein_coding
AABR070	-1.9914	-3.9763	0.00029	0.00113	1	ENSRNO(	protein_coding
Adamts17	-1.9911	-3.9755	0.00252	0.00811	1	ENSRNO(	protein_coding
Fgf2	-1.9854	-3.9596	5.8E-09	4.3E-08	0.000116	ENSRNO(	protein_coding
LOC1009	-1.9828	-3.9525	2.3E-26	7.6E-25	4.69E-22	ENSRNO(	lincRNA
Vwa2	-1.9805	-3.9462	0.00998	0.02769	1	ENSRNO(	protein_coding
RGD1560	-1.9759	-3.9338	0.00681	0.0198	1	ENSRNO(	protein_coding
Rpp25	-1.9739	-3.9283	1.9E-20	4E-19	3.81E-16	ENSRNO(	protein_coding
Sik1	-1.9728	-3.9254	8.5E-24	2.3E-22	1.71E-19	ENSRNO(	protein_coding
Pou2f2	-1.9698	-3.9171	0.00085	0.00301	1	ENSRNO(	protein_coding
Foxa2	-1.9697	-3.9167	4.9E-35	2.5E-33	9.8E-31	ENSRNO(	protein_coding
Lrrc15	-1.9678	-3.9116	1E-05	4.9E-05	0.202985	ENSRNO(	protein_coding

Mob3b	-1.9639	-3.9011	0.00018	0.00072	1	ENSRNO0	protein_coding
Agbl2	-1.9619	-3.8956	0.0125	0.03387	1	ENSRNO0	protein_coding
AABR070	-1.9617	-3.8953	0.0101	0.02794	1	ENSRNO0	protein_coding
Ces5a	-1.9581	-3.8856	2.9E-06	1.5E-05	0.057855	ENSRNO0	protein_coding
Exo1	-1.9581	-3.8854	1.6E-43	1.1E-41	3.27E-39	ENSRNO0	protein_coding
Spata31d	-1.9486	-3.8601	0.01748	0.04557	1	ENSRNO0	protein_coding
Nkain1	-1.9467	-3.8549	2.4E-09	1.9E-08	4.79E-05	ENSRNO0	protein_coding
Eml1	-1.9449	-3.8502	8.5E-33	4E-31	1.72E-28	ENSRNO0	protein_coding
Nid2	-1.9424	-3.8435	5.9E-47	4.6E-45	1.18E-42	ENSRNO0	protein_coding
Ptprv	-1.9397	-3.8362	1.6E-26	5.2E-25	3.15E-22	ENSRNO0	protein_coding
Hhip	-1.9369	-3.8288	1.7E-05	8E-05	0.343097	ENSRNO0	protein_coding
Ggt1	-1.9364	-3.8274	3.5E-12	3.7E-11	6.98E-08	ENSRNO0	protein_coding
Sftpd	-1.9314	-3.8142	0.0166	0.04353	1	ENSRNO0	protein_coding
Slc4a8	-1.9308	-3.8126	9.4E-08	6E-07	0.001893	ENSRNO0	protein_coding
Dnph1	-1.93	-3.8105	2.5E-19	4.8E-18	5.05E-15	ENSRNO0	protein_coding
Tfap4	-1.9261	-3.8001	7.9E-23	2E-21	1.6E-18	ENSRNO0	protein_coding
Slc6a4	-1.9214	-3.7879	0.00415	0.01271	1	ENSRNO0	protein_coding
Actg2	-1.9121	-3.7636	1.5E-05	7E-05	0.295054	ENSRNO0	protein_coding
Chac1	-1.9108	-3.7602	5.9E-30	2.3E-28	1.19E-25	ENSRNO0	protein_coding
Kif17	-1.9079	-3.7526	0.00114	0.00393	1	ENSRNO0	protein_coding
Lsm2	-1.9038	-3.7421	3.2E-37	1.8E-35	6.36E-33	ENSRNO0	protein_coding
Myrip	-1.9035	-3.7413	1.4E-08	9.9E-08	0.00028	ENSRNO0	protein_coding
Sh2d1b	-1.9028	-3.7394	1.1E-15	1.6E-14	2.24E-11	ENSRNO0	protein_coding
AABR070	-1.9023	-3.7382	7.5E-07	4.3E-06	0.015035	ENSRNO0	protein_coding
Aspg	-1.8994	-3.7307	1E-24	2.9E-23	2.05E-20	ENSRNO0	protein_coding
Oacyl	-1.8969	-3.7242	0.00803	0.02288	1	ENSRNO0	protein_coding
Slc7a5	-1.8904	-3.7073	5E-102	2E-99	1.08E-97	ENSRNO0	protein_coding
Epb41l4b	-1.8815	-3.6845	1.9E-07	1.2E-06	0.003767	ENSRNO0	protein_coding
Sh2b2	-1.8733	-3.6638	7.8E-08	5E-07	0.001569	ENSRNO0	protein_coding
Phlda1	-1.873	-3.663	5.1E-36	2.7E-34	1.02E-31	ENSRNO0	protein_coding
Pnma3	-1.8709	-3.6576	0.00027	0.00106	1	ENSRNO0	protein_coding
LOC1025	-1.8709	-3.6576	0.01239	0.03359	1	ENSRNO0	protein_coding
Tuba8	-1.8708	-3.6573	0.01708	0.04467	1	ENSRNO0	protein_coding
Hist1h3b_	-1.8687	-3.6519	0.00092	0.00323	1	ENSRNO0	protein_coding
AABR070	-1.8666	-3.6466	4.6E-09	3.5E-08	9.3E-05	ENSRNO0	pseudogene
Map3k19	-1.8529	-3.6123	0.00207	0.00677	1	ENSRNO0	protein_coding
E2f8	-1.851	-3.6074	4.1E-35	2.1E-33	8.2E-31	ENSRNO0	protein_coding
Crabp2_2	-1.8475	-3.5988	5.9E-08	3.9E-07	0.001197	ENSRNO0	protein_coding
Spats1	-1.8445	-3.5912	0.01336	0.03595	1	ENSRNO0	protein_coding
Myo3a	-1.841	-3.5827	0.00424	0.01297	1	ENSRNO0	protein_coding
Slc16a6	-1.8393	-3.5784	0.00016	0.00066	1	ENSRNO0	protein_coding
Ckmt1	-1.8393	-3.5784	0.00254	0.00814	1	ENSRNO0	protein_coding
Syt7	-1.8342	-3.5657	0.0052	0.01557	1	ENSRNO0	protein_coding
LOC1009	-1.8342	-3.5657	2.5E-05	0.00011	0.493856	ENSRNO0	protein_coding
AABR070	-1.8323	-3.561	0.00016	0.00065	1	ENSRNO0	lincRNA
Mnd1	-1.8261	-3.5458	3.2E-15	4.5E-14	6.46E-11	ENSRNO0	protein_coding
Gnb3	-1.8239	-3.5405	0.00306	0.00964	1	ENSRNO0	protein_coding
Fam118a	-1.8238	-3.5402	0.003	0.00949	1	ENSRNO0	protein_coding
Ces2g	-1.8236	-3.5396	1.5E-06	8.3E-06	0.030596	ENSRNO0	protein_coding

Nup210	-1.8217	-3.5349	0.0014	0.00475	1	ENSRNO(	protein_coding	
Itga8	-1.82	-3.5309	1.5E-41	9.6E-40	2.96E-37	ENSRNO(	protein_coding	
Relt	-1.8182	-3.5264	2.2E-29	8.3E-28	4.35E-25	ENSRNO(	protein_coding	
Tmem19(	-1.8172	-3.524	0.00026	0.00102	1	ENSRNO(	protein_coding	
Cxcl1	-1.8108	-3.5085	2.6E-30	1.1E-28	5.31E-26	ENSRNO(	protein_coding	
ENSRNO	-1.809	-3.504	1.3E-06	7.2E-06	0.026498	ENSRNO(	protein_coding	
Gja4	-1.8069	-3.4989	4.8E-26	1.5E-24	9.66E-22	ENSRNO(	protein_coding	
Pla2g1b	-1.8035	-3.4907	0.00786	0.02249	1	ENSRNO(	protein_coding	
Fam184b	-1.799	-3.4797	5.4E-06	2.8E-05	0.109668	ENSRNO(	protein_coding	
Myo1b	-1.794	-3.4677	1.4E-20	3.1E-19	2.88E-16	ENSRNO(	protein_coding	
Tmem11(	-1.792	-3.4628	5.4E-21	1.2E-19	1.08E-16	ENSRNO(	protein_coding	
Hoxa4	-1.7856	-3.4475	0.00023	0.0009	1	ENSRNO(	protein_coding	
Flg2	-1.783	-3.4414	0.00809	0.02303	1	ENSRNO(	protein_coding	
Tnfrsf21	-1.7813	-3.4375	1.6E-30	6.7E-29	3.26E-26	ENSRNO(	protein_coding	
Plekhg1	-1.776	-3.4247	3.2E-28	1.1E-26	6.41E-24	ENSRNO(	protein_coding	
AABR070	-1.7757	-3.4241	8.4E-05	0.00036	1	ENSRNO(	protein_coding	
Pou5f1	-1.7685	-3.4069	8.1E-07	4.6E-06	0.016232	ENSRNO(	protein_coding	
Lcp1	-1.766	-3.4012	1.9E-29	7.4E-28	3.86E-25	ENSRNO(	protein_coding	
Rnd1	-1.7659	-3.4008	1.9E-15	2.7E-14	3.79E-11	ENSRNO(	protein_coding	
Myh2	-1.7653	-3.3995	0.00068	0.00246	1	ENSRNO(	protein_coding	
Fv1	-1.7644	-3.3974	1.9E-43	1.3E-41	3.87E-39	ENSRNO(	protein_coding	
P2ry2	-1.7627	-3.3934	1.3E-17	2.3E-16	2.71E-13	ENSRNO(	protein_coding	
Eme1	-1.7622	-3.3921	2E-41	1.3E-39	4.07E-37	ENSRNO(	protein_coding	
Kbtbd6_2	-1.7616	-3.3909	2.7E-05	0.00012	0.543872	ENSRNO(	protein_coding	
Atg9b	-1.761	-3.3894	1.6E-05	7.7E-05	0.326053	ENSRNO(	protein_coding	
Lhx2	-1.7567	-3.3792	1.8E-15	2.6E-14	3.63E-11	ENSRNO(	protein_coding	
Trip13	-1.7507	-3.3651	2.2E-30	9E-29	4.41E-26	ENSRNO(	protein_coding	
AABR070	-1.748	-3.359	2.7E-11	2.6E-10	5.35E-07	ENSRNO(	protein_coding	
AABR070	-1.7454	-3.3528	3.6E-08	2.4E-07	0.000717	ENSRNO(	protein_coding	
Il2rg	-1.7436	-3.3486	4.3E-09	3.3E-08	8.62E-05	ENSRNO(	protein_coding	
Trpa1	-1.7379	-3.3354	4.7E-07	2.8E-06	0.00953	ENSRNO(	protein_coding	
AABR070	-1.7377	-3.3351	0.00011	0.00047	1	ENSRNO(	lincRNA	
Rn60_20_	-1.7359	-3.3309	8.9E-05	0.00037	1	ENSRNO(	lincRNA	
Icam4	-1.7358	-3.3306	0.00167	0.00557	1	ENSRNO(	protein_coding	
Pelp1	-1.7338	-3.326	5.4E-86	1.2E-83	1.08E-81	ENSRNO(	protein_coding	
Adam33	-1.733	-3.3242	0.00437	0.01333	1	ENSRNO(	protein_coding	
Serpinb1a	-1.7283	-3.3135	4.3E-20	8.8E-19	8.6E-16	ENSRNO(	protein_coding	
Exoc3l4	-1.727	-3.3104	6.7E-07	3.8E-06	0.013423	ENSRNO(	protein_coding	
Dlx2	-1.7203	-3.295	1E-05	4.9E-05	0.203995	ENSRNO(	protein_coding	
Enpp7	-1.7192	-3.2925	0.00148	0.005	1	ENSRNO(	protein_coding	
Pde2a	-1.7186	-3.2912	0.00163	0.00544	1	ENSRNO(	protein_coding	
Cd28	-1.7177	-3.2892	6.8E-08	4.5E-07	0.001376	ENSRNO(	protein_coding	
Alox5ap	-1.7126	-3.2775	0.00998	0.02768	1	ENSRNO(	protein_coding	
Cdc6	-1.7123	-3.2769	3.6E-37	2E-35	7.33E-33	ENSRNO(	protein_coding	
Gper1	-1.7119	-3.276	4.5E-05	0.0002	0.916201	ENSRNO(	protein_coding	
Rrs1	-1.7079	-3.2669	1.1E-38	6.4E-37	2.16E-34	ENSRNO(	protein_coding	
Slc17a6	-1.6948	-3.2373	0.00928	0.02597	1	ENSRNO(	protein_coding	
Aox2	-1.6923	-3.2316	4.7E-08	3.1E-07	0.000938	ENSRNO(	protein_coding	
Acap1	-1.692	-3.2309	1.9E-07	1.1E-06	0.003738	ENSRNO(	protein_coding	

Lrfn4	-1.6911	-3.2291	1.7E-28	6.1E-27	3.35E-24	ENSRNO0	protein_coding
Fads6	-1.69	-3.2266	0.00011	0.00046	1	ENSRNO0	protein_coding
Pold1	-1.6895	-3.2254	8.5E-33	4E-31	1.71E-28	ENSRNO0	protein_coding
Rn50_8_0	-1.6876	-3.2212	0.00016	0.00066	1	ENSRNO0	processed_transcript
Ripk4	-1.6836	-3.2124	0.00432	0.01319	1	ENSRNO0	protein_coding
Ptma	-1.6812	-3.207	2.5E-07	1.5E-06	0.004941	ENSRNO0	protein_coding
RGD1564	-1.6811	-3.2068	0.00645	0.01889	1	ENSRNO0	protein_coding
Fbln1	-1.6797	-3.2037	0.00571	0.01696	1	ENSRNO0	protein_coding
Fxn	-1.677	-3.1976	7.3E-23	1.8E-21	1.47E-18	ENSRNO0	protein_coding
Lyar	-1.676	-3.1954	3.7E-34	1.8E-32	7.54E-30	ENSRNO0	protein_coding
Als2cr12	-1.6714	-3.1853	0.00336	0.01053	1	ENSRNO0	protein_coding
Ankrd23	-1.6709	-3.1842	7E-05	0.0003	1	ENSRNO0	protein_coding
Rn50_8_0	-1.6669	-3.1754	7.1E-10	5.9E-09	1.42E-05	ENSRNO0	processed_transcript
Rsph1	-1.6668	-3.175	0.00042	0.00159	1	ENSRNO0	protein_coding
Pole2	-1.6653	-3.1719	1.1E-26	3.6E-25	2.14E-22	ENSRNO0	protein_coding
AABR070	-1.6651	-3.1713	0.01745	0.04551	1	ENSRNO0	protein_coding
LOC3035	-1.6642	-3.1694	5.8E-23	1.5E-21	1.18E-18	ENSRNO0	protein_coding
Prdx5	-1.6642	-3.1693	7.9E-40	4.9E-38	1.59E-35	ENSRNO0	protein_coding
Slc20a1	-1.6632	-3.1673	4.7E-55	4.8E-53	9.39E-51	ENSRNO0	protein_coding
Tdp1	-1.663	-3.1668	3.6E-26	1.2E-24	7.32E-22	ENSRNO0	protein_coding
Blnk	-1.6622	-3.165	6.7E-07	3.9E-06	0.013553	ENSRNO0	protein_coding
Abca17	-1.662	-3.1645	0.01291	0.03486	1	ENSRNO0	protein_coding
Set_1	-1.6607	-3.1616	1.2E-16	1.9E-15	2.33E-12	ENSRNO0	protein_coding
Ncan	-1.6596	-3.1593	0.01111	0.03047	1	ENSRNO0	protein_coding
Sox12	-1.658	-3.1557	3.1E-06	1.6E-05	0.062307	ENSRNO0	protein_coding
Chtf18	-1.6578	-3.1555	6E-26	1.9E-24	1.21E-21	ENSRNO0	protein_coding
Pa2g4	-1.6555	-3.1504	3.2E-36	1.7E-34	6.36E-32	ENSRNO0	protein_coding
AABR070	-1.6555	-3.1503	0.00591	0.01748	1	ENSRNO0	protein_coding
Klhl30	-1.6554	-3.1502	4.1E-18	7.2E-17	8.16E-14	ENSRNO0	protein_coding
Ncf2	-1.6514	-3.1415	0.00019	0.00077	1	ENSRNO0	processed_transcript
Ncbp2_2	-1.6493	-3.1368	0.00037	0.0014	1	ENSRNO0	protein_coding
Gdnf	-1.6474	-3.1328	7E-06	3.5E-05	0.140702	ENSRNO0	protein_coding
Jph3	-1.6458	-3.1293	0.00017	0.00069	1	ENSRNO0	protein_coding
Cdca7	-1.6434	-3.1241	5.5E-20	1.1E-18	1.11E-15	ENSRNO0	protein_coding
Inhbe	-1.6424	-3.1219	0.01846	0.04777	1	ENSRNO0	protein_coding
Dscc1	-1.6423	-3.1217	2.1E-27	7.4E-26	4.32E-23	ENSRNO0	protein_coding
Ranbp1	-1.6417	-3.1203	6.3E-29	2.4E-27	1.27E-24	ENSRNO0	protein_coding
Dnd1	-1.6381	-3.1125	2.6E-06	1.4E-05	0.053056	ENSRNO0	protein_coding
LOC1009	-1.6355	-3.1069	1.7E-23	4.4E-22	3.37E-19	ENSRNO0	protein_coding
Mettl1	-1.6323	-3.1	4E-19	7.6E-18	8.05E-15	ENSRNO0	protein_coding
Syn3	-1.6301	-3.0952	0.00489	0.01472	1	ENSRNO0	protein_coding
Dlx1	-1.6289	-3.0928	6.7E-10	5.6E-09	1.35E-05	ENSRNO0	protein_coding
LOC1001	-1.6283	-3.0914	1.4E-20	3E-19	2.8E-16	ENSRNO0	protein_coding
Homer2	-1.6282	-3.0912	0.00658	0.0192	1	ENSRNO0	protein_coding
B3gat1	-1.6258	-3.0861	0.01331	0.03585	1	ENSRNO0	protein_coding
Mybl1	-1.6246	-3.0836	3.1E-67	4.1E-65	6.2E-63	ENSRNO0	protein_coding
LOC4982	-1.6236	-3.0815	0.01096	0.03009	1	ENSRNO0	protein_coding
Rab9b	-1.6233	-3.0807	0.00033	0.00126	1	ENSRNO0	protein_coding
Pole3	-1.6231	-3.0804	3.7E-10	3.2E-09	7.39E-06	ENSRNO0	protein_coding

Cacna1f	-1.6228	-3.0798	0.00061	0.00222	1	ENSRNO(	protein_coding
Cacna1e	-1.6224	-3.0788	0.01616	0.04251	1	ENSRNO(	protein_coding
Fxyd5	-1.6213	-3.0765	4E-32	1.8E-30	8E-28	ENSRNO(	protein_coding
RGD1560	-1.6212	-3.0762	3.9E-13	4.7E-12	7.95E-09	ENSRNO(	protein_coding
Enkd1	-1.6171	-3.0676	2.5E-11	2.4E-10	4.98E-07	ENSRNO(	protein_coding
RGD1563	-1.6168	-3.067	6.4E-07	3.7E-06	0.012818	ENSRNO(	protein_coding
Mlf1	-1.6166	-3.0665	3.1E-13	3.7E-12	6.3E-09	ENSRNO(	protein_coding
Fcna	-1.6157	-3.0647	6.3E-05	0.00027	1	ENSRNO(	protein_coding
Rassf4	-1.6156	-3.0643	0.01002	0.02777	1	ENSRNO(	protein_coding
Disp2	-1.6153	-3.0638	0.01121	0.03069	1	ENSRNO(	protein_coding
Mill1	-1.615	-3.0632	0.00016	0.00065	1	ENSRNO(	protein_coding
Ak4	-1.6143	-3.0616	9.6E-07	5.4E-06	0.019265	ENSRNO(	protein_coding
AABR070	-1.6139	-3.0607	0.00013	0.00055	1	ENSRNO(	protein_coding
Tnfaip3	-1.6134	-3.0598	2.3E-20	4.8E-19	4.59E-16	ENSRNO(	protein_coding
Nop56	-1.6131	-3.0591	6.1E-43	4.2E-41	1.23E-38	ENSRNO(	protein_coding
LOC1009	-1.6107	-3.054	2.6E-12	2.9E-11	5.31E-08	ENSRNO(	protein_coding
Phf11b	-1.6105	-3.0536	5.4E-20	1.1E-18	1.09E-15	ENSRNO(	protein_coding
Arsb	-1.6101	-3.0528	5.3E-47	4.2E-45	1.08E-42	ENSRNO(	protein_coding
Celf3	-1.6084	-3.0492	0.01386	0.03708	1	ENSRNO(	protein_coding
Dusp10	-1.6028	-3.0374	9.1E-17	1.5E-15	1.84E-12	ENSRNO(	protein_coding
M6pr	-1.6024	-3.0364	0.00045	0.00167	1	ENSRNO(	protein_coding
LOC1009	-1.5995	-3.0304	6.7E-31	2.8E-29	1.35E-26	ENSRNO(	protein_coding
Kctd13	-1.5985	-3.0282	2.2E-19	4.2E-18	4.41E-15	ENSRNO(	protein_coding
Prrx2	-1.5973	-3.0258	1.4E-10	1.3E-09	2.85E-06	ENSRNO(	protein_coding
Spns2	-1.5965	-3.024	0.00015	0.00061	1	ENSRNO(	protein_coding
Tnfaip2	-1.5948	-3.0205	4.8E-72	7.4E-70	9.67E-68	ENSRNO(	protein_coding
Colgalt2	-1.5927	-3.0161	0.00431	0.01314	1	ENSRNO(	protein_coding
Oasl	-1.591	-3.0126	1E-09	8.2E-09	2.02E-05	ENSRNO(	protein_coding
Adh7	-1.5881	-3.0065	0.00694	0.02013	1	ENSRNO(	protein_coding
Rab25	-1.5845	-2.999	0.00342	0.01068	1	ENSRNO(	protein_coding
AABR070	-1.5842	-2.9984	0.00676	0.01966	1	ENSRNO(	protein_coding
Areg	-1.5823	-2.9944	1.4E-17	2.4E-16	2.86E-13	ENSRNO(	protein_coding
Mcm10	-1.5799	-2.9896	5.4E-41	3.5E-39	1.09E-36	ENSRNO(	protein_coding
Colq	-1.5796	-2.9889	0.00327	0.01026	1	ENSRNO(	protein_coding
Rnf151	-1.5792	-2.9881	8.9E-05	0.00038	1	ENSRNO(	protein_coding
Ctrl	-1.5772	-2.9838	0.00019	0.00077	1	ENSRNO(	protein_coding
Mcm7	-1.5748	-2.979	1.1E-32	4.9E-31	2.12E-28	ENSRNO(	protein_coding
Mapk6	-1.5728	-2.9748	2.6E-45	1.9E-43	5.21E-41	ENSRNO(	protein_coding
Procr	-1.5717	-2.9726	0.00018	0.00073	1	ENSRNO(	protein_coding
Pola2	-1.5712	-2.9716	1.6E-30	6.6E-29	3.19E-26	ENSRNO(	protein_coding
Rbm12_2	-1.5706	-2.9703	2.4E-06	1.3E-05	0.049127	ENSRNO(	protein_coding
Fbxo5	-1.57	-2.9691	5E-40	3.1E-38	1.02E-35	ENSRNO(	protein_coding
Lmo1	-1.5659	-2.9607	7.8E-27	2.6E-25	1.57E-22	ENSRNO(	protein_coding
Ppan	-1.5638	-2.9564	3.3E-32	1.5E-30	6.7E-28	ENSRNO(	protein_coding
AABR070	-1.5621	-2.9528	0.00915	0.02563	1	ENSRNO(	pseudogene
Gpatch4	-1.5613	-2.9512	9.4E-30	3.6E-28	1.88E-25	ENSRNO(	protein_coding
Col7a1	-1.5612	-2.9509	0.00075	0.00268	1	ENSRNO(	protein_coding
Phgdh	-1.5568	-2.9419	4.9E-23	1.2E-21	9.78E-19	ENSRNO(	protein_coding
Naa10	-1.5535	-2.9353	7E-24	1.9E-22	1.41E-19	ENSRNO(	protein_coding

Zfp185	-1.5485	-2.9252	0.00813	0.02311	1	ENSRNO0	protein_coding
Dtnb	-1.5481	-2.9244	3.9E-17	6.5E-16	7.86E-13	ENSRNO0	protein_coding
Ankh	-1.5465	-2.921	7.9E-36	4.1E-34	1.58E-31	ENSRNO0	protein_coding
Mrto4	-1.5458	-2.9197	9.5E-29	3.5E-27	1.92E-24	ENSRNO0	protein_coding
Cdt1	-1.5455	-2.919	3.7E-35	1.9E-33	7.46E-31	ENSRNO0	protein_coding
Dagla	-1.5453	-2.9186	2E-40	1.3E-38	4.09E-36	ENSRNO0	protein_coding
LOC1036	-1.5445	-2.917	2.2E-44	1.6E-42	4.44E-40	ENSRNO0	protein_coding
St6galnac	-1.5442	-2.9163	3.1E-44	2.2E-42	6.33E-40	ENSRNO0	protein_coding
Adcy8	-1.5434	-2.9149	5.3E-14	6.8E-13	1.06E-09	ENSRNO0	protein_coding
Nop2	-1.5426	-2.9132	3.5E-44	2.4E-42	6.96E-40	ENSRNO0	protein_coding
Igsf9b	-1.5423	-2.9126	1.3E-14	1.8E-13	2.7E-10	ENSRNO0	protein_coding
Afmid	-1.5401	-2.9081	5.4E-12	5.7E-11	1.08E-07	ENSRNO0	protein_coding
Pou2af1	-1.5397	-2.9074	0.00546	0.01628	1	ENSRNO0	protein_coding
Uhrf1	-1.5385	-2.905	1.3E-31	5.7E-30	2.66E-27	ENSRNO0	protein_coding
Lrrn2	-1.5371	-2.9021	0.00375	0.01161	1	ENSRNO0	protein_coding
Hmcn1	-1.5364	-2.9006	0.00484	0.01458	1	ENSRNO0	protein_coding
Plxna3	-1.5352	-2.8984	4.3E-18	7.6E-17	8.63E-14	ENSRNO0	protein_coding
Plaur	-1.5347	-2.8972	4.7E-33	2.2E-31	9.54E-29	ENSRNO0	protein_coding
AC111319	-1.5327	-2.8933	1.9E-10	1.7E-09	3.77E-06	ENSRNO0	lincRNA
Pop1	-1.5314	-2.8906	4.6E-18	8.1E-17	9.3E-14	ENSRNO0	protein_coding
Lrrtm3	-1.5306	-2.8891	0.00944	0.02635	1	ENSRNO0	protein_coding
Enthd1	-1.5273	-2.8825	0.0012	0.00411	1	ENSRNO0	protein_coding
Ppp1r13l	-1.5268	-2.8815	1.7E-26	5.5E-25	3.38E-22	ENSRNO0	protein_coding
Odc1	-1.5227	-2.8732	1.1E-24	3.1E-23	2.21E-20	ENSRNO0	protein_coding
Mybbp1a	-1.5193	-2.8665	3.8E-40	2.4E-38	7.68E-36	ENSRNO0	protein_coding
Ttc39c	-1.5145	-2.857	5.1E-15	7.1E-14	1.03E-10	ENSRNO0	protein_coding
Il1rl2	-1.513	-2.854	0.01907	0.04915	1	ENSRNO0	protein_coding
Ncl	-1.513	-2.854	7.3E-33	3.4E-31	1.47E-28	ENSRNO0	protein_coding
Blm	-1.5127	-2.8534	7.5E-33	3.5E-31	1.52E-28	ENSRNO0	protein_coding
Wipf1	-1.5114	-2.851	0.0029	0.0092	1	ENSRNO0	protein_coding
Gpr19	-1.5098	-2.8477	3.9E-21	8.7E-20	7.77E-17	ENSRNO0	protein_coding
Pold2	-1.5097	-2.8476	4.5E-30	1.8E-28	8.99E-26	ENSRNO0	protein_coding
Glb1l2	-1.5083	-2.8447	1.1E-20	2.4E-19	2.25E-16	ENSRNO0	protein_coding
Cst6	-1.5077	-2.8435	0.00275	0.00876	1	ENSRNO0	protein_coding
Laptm5	-1.5049	-2.838	0.01825	0.04734	1	ENSRNO0	protein_coding
Orc1	-1.5032	-2.8347	5E-20	1E-18	1.01E-15	ENSRNO0	protein_coding
Chaf1b	-1.5022	-2.8327	1.1E-26	3.6E-25	2.17E-22	ENSRNO0	protein_coding
Clspn	-1.5015	-2.8313	1.4E-41	9.2E-40	2.84E-37	ENSRNO0	protein_coding
Ccdc134	-1.5003	-2.829	7.6E-18	1.3E-16	1.53E-13	ENSRNO0	protein_coding
H2afx	-1.4982	-2.8249	8.2E-22	1.9E-20	1.65E-17	ENSRNO0	protein_coding
Rn50_8_0	-1.497	-2.8225	0.0035	0.01091	1	ENSRNO0	processed_transcript
AABR070	-1.4921	-2.813	6.4E-07	3.7E-06	0.012977	ENSRNO0	lincRNA
Ttc32	-1.4917	-2.8123	3.4E-10	3E-09	6.94E-06	ENSRNO0	protein_coding
Wbp11l1	-1.4898	-2.8085	4.8E-06	2.4E-05	0.096093	ENSRNO0	protein_coding
Usp1	-1.4885	-2.806	9.8E-40	6E-38	1.97E-35	ENSRNO0	protein_coding
Fgf9	-1.4874	-2.8038	4.9E-06	2.5E-05	0.098774	ENSRNO0	protein_coding
Dnajc2	-1.4862	-2.8014	3.5E-33	1.7E-31	7.04E-29	ENSRNO0	protein_coding
Cd44	-1.4841	-2.7974	5.9E-26	1.9E-24	1.19E-21	ENSRNO0	protein_coding
Kctd4	-1.4838	-2.7969	0.00026	0.00102	1	ENSRNO0	protein_coding

Thop1	-1.4831	-2.7954	2E-33	9.7E-32	4.08E-29	ENSRNO(	protein_coding
Rad51ap1	-1.4827	-2.7948	1.4E-32	6.5E-31	2.85E-28	ENSRNO(	protein_coding
Tonsl	-1.4773	-2.7844	1.3E-28	4.8E-27	2.64E-24	ENSRNO(	protein_coding
Runx1	-1.4721	-2.7743	6.6E-21	1.5E-19	1.32E-16	ENSRNO(	protein_coding
RGD1565	-1.4701	-2.7704	7.8E-22	1.8E-20	1.57E-17	ENSRNO(	protein_coding
AABR070	-1.4686	-2.7676	0.0007	0.00252	1	ENSRNO(	lincRNA
Gar1	-1.4671	-2.7647	1.2E-21	2.9E-20	2.49E-17	ENSRNO(	protein_coding
LOC1083	-1.467	-2.7645	4.9E-34	2.4E-32	9.85E-30	ENSRNO(	protein_coding
Itih2	-1.466	-2.7625	0.00322	0.01012	1	ENSRNO(	protein_coding
Nxph3	-1.466	-2.7625	3.1E-09	2.4E-08	6.3E-05	ENSRNO(	protein_coding
Ifrd2	-1.4652	-2.761	1.3E-26	4.2E-25	2.53E-22	ENSRNO(	protein_coding
LOC4985	-1.464	-2.7588	0.00082	0.00291	1	ENSRNO(	protein_coding
Cenpu	-1.4638	-2.7583	4.4E-21	9.8E-20	8.8E-17	ENSRNO(	protein_coding
Fkbp4	-1.4629	-2.7567	6.3E-63	7.8E-61	1.27E-58	ENSRNO(	protein_coding
Gemin6	-1.4627	-2.7563	4.5E-15	6.3E-14	9.1E-11	ENSRNO(	protein_coding
AABR070	-1.4623	-2.7554	5.4E-09	4.1E-08	0.000109	ENSRNO(	protein_coding
Cenpm	-1.4599	-2.7509	5.6E-16	8.6E-15	1.13E-11	ENSRNO(	protein_coding
Exosc2	-1.4599	-2.7509	1.8E-30	7.4E-29	3.6E-26	ENSRNO(	protein_coding
Dctd	-1.4553	-2.7421	2.5E-31	1.1E-29	5.11E-27	ENSRNO(	protein_coding
Ctrb1	-1.4547	-2.741	7.6E-06	3.7E-05	0.152226	ENSRNO(	protein_coding
Nolc1	-1.4537	-2.7392	7.6E-32	3.4E-30	1.54E-27	ENSRNO(	protein_coding
Espnl	-1.4509	-2.7339	0.00033	0.00128	1	ENSRNO(	protein_coding
Dtl	-1.4508	-2.7336	8.8E-28	3.1E-26	1.77E-23	ENSRNO(	protein_coding
Mmp10	-1.4507	-2.7333	2.8E-19	5.4E-18	5.63E-15	ENSRNO(	protein_coding
Mrps10	-1.4501	-2.7323	2E-26	6.5E-25	4E-22	ENSRNO(	protein_coding
Mcm5	-1.4476	-2.7275	1.5E-23	4.1E-22	3.08E-19	ENSRNO(	protein_coding
Celf4	-1.4466	-2.7256	0.01183	0.03219	1	ENSRNO(	protein_coding
Ppargc1b	-1.4428	-2.7184	1.1E-05	5.2E-05	0.216748	ENSRNO(	protein_coding
Npas2	-1.44	-2.7131	8.2E-16	1.2E-14	1.64E-11	ENSRNO(	protein_coding
Pcdh18	-1.4372	-2.708	2E-29	7.8E-28	4.08E-25	ENSRNO(	protein_coding
Ly6l	-1.4336	-2.7012	4.9E-05	0.00021	0.982992	ENSRNO(	lincRNA
Ly49s4	-1.4326	-2.6993	0.01651	0.04331	1	ENSRNO(	protein_coding
Rrp8	-1.4309	-2.6961	2E-33	9.8E-32	4.11E-29	ENSRNO(	protein_coding
Plekho1	-1.4305	-2.6954	1.3E-10	1.2E-09	2.62E-06	ENSRNO(	protein_coding
Rrp9	-1.4288	-2.6922	2E-24	5.8E-23	4.11E-20	ENSRNO(	protein_coding
Gins4	-1.4264	-2.6877	1.3E-20	2.9E-19	2.69E-16	ENSRNO(	protein_coding
Tfrc	-1.4263	-2.6875	2.4E-18	4.4E-17	4.9E-14	ENSRNO(	protein_coding
Npm3	-1.4257	-2.6864	3.6E-11	3.5E-10	7.25E-07	ENSRNO(	protein_coding
Rtn4r	-1.4241	-2.6835	0.00019	0.00077	1	ENSRNO(	protein_coding
Chaf1a	-1.4234	-2.6821	8.5E-39	5E-37	1.7E-34	ENSRNO(	protein_coding
Rad54b	-1.4231	-2.6815	2.6E-15	3.7E-14	5.17E-11	ENSRNO(	protein_coding
Xrcc2	-1.4212	-2.6781	2.6E-28	9.2E-27	5.14E-24	ENSRNO(	protein_coding
Fcer1g	-1.4209	-2.6776	0.00855	0.02414	1	ENSRNO(	protein_coding
Tnfsf9	-1.4206	-2.6769	5.8E-12	6.1E-11	1.17E-07	ENSRNO(	protein_coding
Cep41	-1.4203	-2.6764	8.6E-24	2.3E-22	1.74E-19	ENSRNO(	protein_coding
Snrpa	-1.4201	-2.6761	1.4E-28	5E-27	2.74E-24	ENSRNO(	protein_coding
Morc4	-1.4193	-2.6745	1.1E-27	3.8E-26	2.21E-23	ENSRNO(	protein_coding
Ska1	-1.4189	-2.6738	5.9E-25	1.7E-23	1.18E-20	ENSRNO(	protein_coding
Epsti1	-1.4186	-2.6732	0.00397	0.01221	1	ENSRNO(	protein_coding

Entpd3	-1.4185	-2.6731	0.00053	0.00195	1	ENSRNO(	protein_coding
Hirip3	-1.4177	-2.6716	2.7E-36	1.4E-34	5.36E-32	ENSRNO(	protein_coding
Cck	-1.4163	-2.669	0.00028	0.00108	1	ENSRNO(	protein_coding
Spn	-1.4151	-2.6667	9E-14	1.1E-12	1.8E-09	ENSRNO(	protein_coding
Ihh	-1.4139	-2.6645	0.01547	0.04089	1	ENSRNO(	protein_coding
Ust5r	-1.4114	-2.66	6E-09	4.5E-08	0.000122	ENSRNO(	protein_coding
Recql4	-1.4086	-2.6548	3.1E-22	7.5E-21	6.21E-18	ENSRNO(	protein_coding
Nhp2	-1.4077	-2.6531	7.1E-10	5.9E-09	1.43E-05	ENSRNO(	protein_coding
Eng	-1.4049	-2.648	0.00261	0.00835	1	ENSRNO(	protein_coding
Gfi1	-1.4033	-2.6451	6.8E-06	3.4E-05	0.136221	ENSRNO(	protein_coding
Sipa1l3	-1.4024	-2.6434	2.3E-24	6.4E-23	4.6E-20	ENSRNO(	protein_coding
Zmynd15	-1.4024	-2.6433	6.7E-05	0.00029	1	ENSRNO(	protein_coding
Cndp1	-1.4022	-2.6431	0.00066	0.00239	1	ENSRNO(	protein_coding
Tp53	-1.4021	-2.6429	9.8E-32	4.3E-30	1.98E-27	ENSRNO(	protein_coding
Ldlrad3	-1.4013	-2.6414	0.00016	0.00066	1	ENSRNO(	protein_coding
AABR070	-1.3988	-2.6369	0.01754	0.04569	1	ENSRNO(	pseudogene
RGD1305	-1.3967	-2.633	3.5E-29	1.3E-27	7.06E-25	ENSRNO(	protein_coding
Lrp8	-1.3955	-2.6308	3E-36	1.6E-34	5.99E-32	ENSRNO(	protein_coding
Bmp3	-1.3909	-2.6224	2E-05	9.1E-05	0.392694	ENSRNO(	protein_coding
Haus8	-1.3906	-2.6218	1.1E-24	3.2E-23	2.24E-20	ENSRNO(	protein_coding
Cenph	-1.3899	-2.6206	4.1E-26	1.3E-24	8.35E-22	ENSRNO(	protein_coding
Mcpt8l2	-1.389	-2.619	0.01183	0.0322	1	ENSRNO(	protein_coding
Cd81	-1.3886	-2.6183	0.00238	0.0077	1	ENSRNO(	protein_coding
Cetn4	-1.3883	-2.6177	4.1E-10	3.5E-09	8.27E-06	ENSRNO(	protein_coding
Rn50_16_	-1.3874	-2.6161	2.1E-07	1.3E-06	0.004226	ENSRNO(	processed_pseudogene
Cbr3	-1.3869	-2.6152	1.8E-09	1.5E-08	3.71E-05	ENSRNO(	protein_coding
Dlg3	-1.3846	-2.6109	0.00091	0.00318	1	ENSRNO(	protein_coding
Ccdc86	-1.3836	-2.6093	2.5E-24	7.1E-23	5.11E-20	ENSRNO(	protein_coding
Cenpo	-1.3835	-2.6091	1.9E-36	1E-34	3.81E-32	ENSRNO(	protein_coding
Sts	-1.3812	-2.6048	1.9E-12	2.1E-11	3.83E-08	ENSRNO(	protein_coding
LOC1009	-1.3808	-2.6042	0.01172	0.03195	1	ENSRNO(	protein_coding
Lamb3	-1.3802	-2.603	1.9E-23	5E-22	3.82E-19	ENSRNO(	protein_coding
LOC1009	-1.38	-2.6026	7.5E-26	2.3E-24	1.52E-21	ENSRNO(	protein_coding
Nrn1	-1.3792	-2.6012	6.6E-14	8.4E-13	1.33E-09	ENSRNO(	protein_coding
Dnai2	-1.3783	-2.5997	0.00935	0.02614	1	ENSRNO(	protein_coding
Asns	-1.3772	-2.5977	1.1E-53	1.1E-51	2.16E-49	ENSRNO(	protein_coding
Snx31	-1.3765	-2.5965	0.01774	0.04612	1	ENSRNO(	protein_coding
Mgme1	-1.3761	-2.5956	1.8E-15	2.6E-14	3.63E-11	ENSRNO(	protein_coding
Cdh5	-1.3736	-2.5912	0.00034	0.00129	1	ENSRNO(	protein_coding
Ipcef1	-1.3722	-2.5887	0.00044	0.00165	1	ENSRNO(	protein_coding
Smco4	-1.3721	-2.5885	0.00337	0.01056	1	ENSRNO(	protein_coding
Lig1	-1.3719	-2.5882	8.1E-25	2.3E-23	1.64E-20	ENSRNO(	protein_coding
Rfc5	-1.3711	-2.5867	2.8E-53	2.7E-51	5.63E-49	ENSRNO(	protein_coding
Rad54l	-1.3704	-2.5854	2.8E-24	7.7E-23	5.59E-20	ENSRNO(	protein_coding
Rundc3b	-1.3692	-2.5833	0.01423	0.03798	1	ENSRNO(	protein_coding
Orc6	-1.3682	-2.5815	1.1E-18	2E-17	2.24E-14	ENSRNO(	protein_coding
AC11788!	-1.3676	-2.5805	1.4E-05	6.6E-05	0.278533	ENSRNO(	protein_coding
Gipr	-1.367	-2.5794	0.00103	0.00359	1	ENSRNO(	protein_coding
Abcb1a	-1.3663	-2.578	2E-34	9.7E-33	3.95E-30	ENSRNO(	protein_coding

Tpd52l1	-1.3657	-2.577	0.00421	0.01289	1	ENSRNO(	protein_coding
Nmnat2	-1.3654	-2.5765	1.9E-05	8.7E-05	0.374008	ENSRNO(	protein_coding
Cdv3	-1.3645	-2.5748	3.4E-18	6.1E-17	6.91E-14	ENSRNO(	protein_coding
Rfc3	-1.3641	-2.5742	7.8E-14	9.8E-13	1.58E-09	ENSRNO(	protein_coding
Ns5atp9	-1.3638	-2.5736	1.2E-15	1.8E-14	2.48E-11	ENSRNO(	protein_coding
Set_2	-1.3621	-2.5706	2.4E-28	8.8E-27	4.88E-24	ENSRNO(	protein_coding
Rrh	-1.3603	-2.5675	0.00931	0.02605	1	ENSRNO(	protein_coding
Ddias	-1.36	-2.5668	4.9E-23	1.2E-21	9.9E-19	ENSRNO(	protein_coding
F2rl1	-1.3583	-2.5638	1.2E-20	2.7E-19	2.48E-16	ENSRNO(	protein_coding
Kcnt2	-1.3574	-2.5623	0.00657	0.01918	1	ENSRNO(	protein_coding
Shmt2	-1.3563	-2.5602	5.8E-53	5.6E-51	1.18E-48	ENSRNO(	protein_coding
Pmf1	-1.3562	-2.5601	4.2E-26	1.3E-24	8.38E-22	ENSRNO(	protein_coding
Jag2	-1.3558	-2.5594	2.8E-24	7.7E-23	5.61E-20	ENSRNO(	protein_coding
Cad	-1.3558	-2.5594	1.3E-26	4.4E-25	2.65E-22	ENSRNO(	protein_coding
Nutm1	-1.3554	-2.5587	0.00193	0.00635	1	ENSRNO(	protein_coding
Tssk6	-1.3552	-2.5584	0.0006	0.00218	1	ENSRNO(	protein_coding
Ercc6l	-1.3541	-2.5564	4.9E-26	1.5E-24	9.87E-22	ENSRNO(	protein_coding
Nme6	-1.3535	-2.5552	5.4E-13	6.3E-12	1.09E-08	ENSRNO(	protein_coding
Gins3	-1.3502	-2.5495	2.1E-24	5.9E-23	4.24E-20	ENSRNO(	protein_coding
Sapcd2	-1.3502	-2.5494	4.1E-13	4.9E-12	8.35E-09	ENSRNO(	protein_coding
Mark1	-1.3499	-2.5489	1.5E-07	9.3E-07	0.002982	ENSRNO(	protein_coding
Tube1	-1.3495	-2.5482	1.7E-15	2.5E-14	3.42E-11	ENSRNO(	protein_coding
Traip	-1.3489	-2.5472	4.4E-26	1.4E-24	8.89E-22	ENSRNO(	protein_coding
Ccna2	-1.3479	-2.5454	1.8E-23	4.7E-22	3.6E-19	ENSRNO(	protein_coding
AC124205	-1.3474	-2.5445	0.01756	0.04573	1	ENSRNO(	pseudogene
Cox6a2	-1.3469	-2.5436	0.0011	0.00378	1	ENSRNO(	protein_coding
Fancb	-1.346	-2.542	3.8E-15	5.3E-14	7.63E-11	ENSRNO(	protein_coding
Chchd6	-1.3434	-2.5375	8E-15	1.1E-13	1.61E-10	ENSRNO(	protein_coding
Iqcd	-1.3424	-2.5357	4.4E-11	4.2E-10	8.76E-07	ENSRNO(	protein_coding
Rgs14	-1.3404	-2.5322	0.01427	0.03808	1	ENSRNO(	protein_coding
Wdr46	-1.339	-2.5298	1.4E-20	3E-19	2.8E-16	ENSRNO(	protein_coding
LOC1003	-1.3389	-2.5296	7.6E-05	0.00033	1	ENSRNO(	protein_coding
Mcm3	-1.335	-2.5227	7E-25	2.1E-23	1.41E-20	ENSRNO(	protein_coding
Spc25	-1.3348	-2.5224	5.1E-20	1E-18	1.02E-15	ENSRNO(	protein_coding
Hlx	-1.3344	-2.5217	2.3E-11	2.3E-10	4.57E-07	ENSRNO(	protein_coding
Suv39h1l	-1.3339	-2.5209	3.3E-26	1.1E-24	6.7E-22	ENSRNO(	protein_coding
Mylk3	-1.3325	-2.5184	0.0083	0.02354	1	ENSRNO(	protein_coding
Adcy3	-1.3312	-2.5161	5.9E-15	8.1E-14	1.18E-10	ENSRNO(	protein_coding
AABR070	-1.3311	-2.5159	3.5E-09	2.7E-08	7.12E-05	ENSRNO(	protein_coding
Fam25a	-1.3299	-2.5138	1.5E-19	3E-18	3.07E-15	ENSRNO(	protein_coding
Pwp2	-1.3296	-2.5133	1.1E-25	3.3E-24	2.12E-21	ENSRNO(	protein_coding
Mlh3	-1.3294	-2.5129	2.4E-06	1.3E-05	0.049314	ENSRNO(	protein_coding
Wbscr22	-1.3279	-2.5104	5.2E-24	1.4E-22	1.05E-19	ENSRNO(	protein_coding
Mb21d1	-1.3271	-2.509	2.9E-10	2.5E-09	5.87E-06	ENSRNO(	protein_coding
Fpgs	-1.327	-2.5087	5E-24	1.4E-22	1.01E-19	ENSRNO(	protein_coding
Pak1ip1	-1.3269	-2.5086	1.7E-24	4.8E-23	3.42E-20	ENSRNO(	protein_coding
Cxcl16	-1.3268	-2.5084	6.3E-20	1.3E-18	1.26E-15	ENSRNO(	protein_coding
LOC1003	-1.3257	-2.5065	3.7E-25	1.1E-23	7.52E-21	ENSRNO(	protein_coding
Pbk	-1.3257	-2.5065	4.5E-23	1.2E-21	9.11E-19	ENSRNO(	protein_coding

Lrr1	-1.3229	-2.5017	6.8E-09	5.1E-08	0.000138	ENSRNO0	protein_coding
AABR070	-1.3229	-2.5016	6.3E-08	4.1E-07	0.001267	ENSRNO0	protein_coding
Nelfe	-1.3228	-2.5015	1E-22	2.5E-21	2.06E-18	ENSRNO0	protein_coding
Nxt1	-1.3228	-2.5014	4.1E-20	8.5E-19	8.27E-16	ENSRNO0	protein_coding
Chchd4	-1.3226	-2.5011	0.00058	0.00211	1	ENSRNO0	protein_coding
Mgst1	1.32357	2.50285	6.4E-09	4.7E-08	0.000128	ENSRNO0	protein_coding
Zbtb10	1.32443	2.50433	7.5E-13	8.7E-12	1.52E-08	ENSRNO0	protein_coding
AABR070	1.32472	2.50484	0.00103	0.00357	1	ENSRNO0	pseudogene
Tmem8b	1.32488	2.50513	6.8E-05	0.00029	1	ENSRNO0	protein_coding
Gpr157	1.32775	2.51011	1.9E-06	1E-05	0.03807	ENSRNO0	protein_coding
Asl	1.32798	2.51051	9.6E-26	3E-24	1.93E-21	ENSRNO0	protein_coding
Spsb1	1.329	2.51228	2.5E-30	1E-28	4.96E-26	ENSRNO0	protein_coding
Usp35	1.32916	2.51257	9.1E-10	7.5E-09	1.83E-05	ENSRNO0	protein_coding
Nt5dc3	1.33135	2.51638	2.9E-26	9.5E-25	5.91E-22	ENSRNO0	protein_coding
Rab11fip5	1.33172	2.51703	9.1E-50	8E-48	1.83E-45	ENSRNO0	protein_coding
Nuak1	1.33177	2.51712	1.6E-49	1.4E-47	3.21E-45	ENSRNO0	protein_coding
Ophn1	1.33331	2.5198	1.6E-11	1.6E-10	3.23E-07	ENSRNO0	protein_coding
RGD1563	1.33423	2.52141	0.00648	0.01896	1	ENSRNO0	protein_coding
Afap1l1	1.33714	2.5265	0.00015	0.00061	1	ENSRNO0	protein_coding
AABR070	1.33797	2.52795	8.9E-07	5.1E-06	0.018018	ENSRNO0	protein_coding
Ulk3	1.33803	2.52807	3.1E-09	2.4E-08	6.24E-05	ENSRNO0	protein_coding
C1ql3	1.33853	2.52893	0.00723	0.02087	1	ENSRNO0	protein_coding
Sh3bgrl2	1.33863	2.52911	5.4E-10	4.5E-09	1.08E-05	ENSRNO0	protein_coding
Tenm3	1.33867	2.52918	1.5E-22	3.8E-21	3.1E-18	ENSRNO0	protein_coding
AABR070	1.3387	2.52924	2.5E-47	2E-45	5.07E-43	ENSRNO0	lincRNA
Sort1	1.33971	2.531	1.5E-24	4.1E-23	2.94E-20	ENSRNO0	protein_coding
Tmem106	1.34003	2.53157	4.6E-51	4.3E-49	9.32E-47	ENSRNO0	protein_coding
Zmiz2	1.34065	2.53265	2.9E-09	2.2E-08	5.8E-05	ENSRNO0	protein_coding
Bphl	1.34434	2.53914	1.5E-08	1.1E-07	0.000299	ENSRNO0	protein_coding
Agfg2	1.34445	2.53933	2.4E-50	2.2E-48	4.88E-46	ENSRNO0	protein_coding
Tmem173	1.34468	2.53974	5.9E-14	7.5E-13	1.18E-09	ENSRNO0	protein_coding
Nans	1.34505	2.54038	4.1E-24	1.1E-22	8.33E-20	ENSRNO0	protein_coding
Aldh3a2	1.34584	2.54179	2.6E-20	5.4E-19	5.21E-16	ENSRNO0	protein_coding
AABR070	1.34623	2.54246	6.2E-08	4.1E-07	0.001256	ENSRNO0	lincRNA
Bmp4	1.34757	2.54483	3.6E-21	8.1E-20	7.24E-17	ENSRNO0	protein_coding
Dnase1l3	1.34779	2.54521	0.0087	0.02451	1	ENSRNO0	protein_coding
Klf7_1	1.34783	2.54528	1E-18	1.9E-17	2.08E-14	ENSRNO0	protein_coding
Pdha1	1.34917	2.54766	3.9E-26	1.3E-24	7.94E-22	ENSRNO0	protein_coding
Frrs1	1.34976	2.5487	4.3E-37	2.4E-35	8.71E-33	ENSRNO0	protein_coding
Notch3	1.34988	2.5489	0.00122	0.00417	1	ENSRNO0	protein_coding
Nod1	1.35042	2.54987	1.1E-31	4.9E-30	2.26E-27	ENSRNO0	protein_coding
Crebl2	1.35144	2.55166	1.6E-05	7.6E-05	0.322663	ENSRNO0	protein_coding
Bmf	1.35145	2.55168	2.2E-16	3.5E-15	4.41E-12	ENSRNO0	protein_coding
LOC1083	1.35172	2.55216	2.7E-21	6.1E-20	5.4E-17	ENSRNO0	protein_coding
Slpil2	1.35395	2.55611	0.00123	0.00422	1	ENSRNO0	protein_coding
Gpr165	1.35417	2.5565	6.4E-05	0.00028	1	ENSRNO0	protein_coding
Lifr	1.35506	2.55808	4.8E-08	3.2E-07	0.000973	ENSRNO0	protein_coding
LOC3657	1.35558	2.55901	0.01833	0.0475	1	ENSRNO0	protein_coding
Wdr35	1.35655	2.56073	1E-25	3.1E-24	2.04E-21	ENSRNO0	protein_coding

Nalcn	1.35676	2.5611	0.00023	0.0009	1	ENSRNO(	protein_coding
Zfp560	1.35689	2.56133	8E-20	1.6E-18	1.61E-15	ENSRNO(	protein_coding
Ehf	1.36294	2.57209	0.0126	0.03411	1	ENSRNO(	protein_coding
Rhbdl3	1.36305	2.57229	0.01004	0.02783	1	ENSRNO(	protein_coding
Lmo4	1.36478	2.57536	7.4E-24	2E-22	1.49E-19	ENSRNO(	protein_coding
Evpl	1.36482	2.57544	1E-37	6E-36	2.08E-33	ENSRNO(	protein_coding
Cmbl	1.36489	2.57557	8.3E-14	1E-12	1.67E-09	ENSRNO(	protein_coding
Slc24a1	1.36647	2.57839	0.0117	0.0319	1	ENSRNO(	protein_coding
AABR070	1.36679	2.57896	0.00313	0.00985	1	ENSRNO(	lincRNA
Haghl	1.36904	2.58299	0.00028	0.00108	1	ENSRNO(	protein_coding
Dcp2_1	1.37101	2.58651	1.3E-13	1.6E-12	2.65E-09	ENSRNO(	protein_coding
Fam105a	1.37236	2.58893	5.9E-23	1.5E-21	1.18E-18	ENSRNO(	protein_coding
Slc8a3	1.37327	2.59057	0.00014	0.00056	1	ENSRNO(	protein_coding
Enkur	1.37363	2.59122	0.00073	0.00262	1	ENSRNO(	protein_coding
Ctla2a	1.37616	2.59577	0.00713	0.02059	1	ENSRNO(	protein_coding
Herc6	1.37767	2.59848	3E-15	4.2E-14	6E-11	ENSRNO(	protein_coding
Hoxc13	1.379	2.60088	0.00806	0.02294	1	ENSRNO(	protein_coding
Tom1l2	1.37971	2.60216	7.9E-30	3.1E-28	1.58E-25	ENSRNO(	protein_coding
Arrdc3	1.3816	2.60557	5.1E-18	9E-17	1.02E-13	ENSRNO(	protein_coding
Pcdhga2	1.3826	2.60739	4.7E-18	8.3E-17	9.44E-14	ENSRNO(	protein_coding
P2rx1	1.383	2.6081	0.00245	0.00791	1	ENSRNO(	protein_coding
AABR070	1.38315	2.60838	0.00139	0.0047	1	ENSRNO(	protein_coding
Lrriq1	1.38326	2.60858	0.00479	0.01444	1	ENSRNO(	protein_coding
Pgam2	1.38579	2.61316	1.4E-05	6.7E-05	0.280953	ENSRNO(	protein_coding
Micb	1.38782	2.61683	0.00015	0.00059	1	ENSRNO(	protein_coding
Trdn	1.39297	2.62618	2.1E-06	1.1E-05	0.042559	ENSRNO(	protein_coding
Plekhm3	1.39306	2.62635	2E-09	1.6E-08	4.1E-05	ENSRNO(	protein_coding
Vps13d	1.3934	2.62697	1.1E-32	4.9E-31	2.13E-28	ENSRNO(	protein_coding
Bach2	1.39389	2.62786	0.00474	0.0143	1	ENSRNO(	protein_coding
Dmpk	1.39417	2.62838	8.6E-14	1.1E-12	1.73E-09	ENSRNO(	protein_coding
Rn50_20_	1.39606	2.63182	0.00081	0.00286	1	ENSRNO(	antisense_RNA
Mlana	1.39819	2.63571	7.2E-09	5.3E-08	0.000145	ENSRNO(	protein_coding
Col8a1	1.39822	2.63576	1.7E-26	5.7E-25	3.52E-22	ENSRNO(	protein_coding
Fntb	1.40061	2.64013	1.5E-53	1.4E-51	2.94E-49	ENSRNO(	protein_coding
Fam198b	1.40302	2.64454	4.6E-14	6E-13	9.32E-10	ENSRNO(	protein_coding
Gdpd2	1.40533	2.64878	0.00024	0.00093	1	ENSRNO(	protein_coding
Nucb2	1.40674	2.65138	1.2E-24	3.5E-23	2.44E-20	ENSRNO(	protein_coding
Hip1	1.4078	2.65332	2.5E-31	1.1E-29	5.13E-27	ENSRNO(	protein_coding
Ppm1k	1.40797	2.65364	6.6E-15	9.1E-14	1.34E-10	ENSRNO(	protein_coding
Abcd2	1.40839	2.65441	0.00011	0.00047	1	ENSRNO(	protein_coding
Bnip3	1.40871	2.655	1.6E-30	6.7E-29	3.28E-26	ENSRNO(	protein_coding
Hsd17b11	1.40894	2.65542	3.8E-27	1.3E-25	7.64E-23	ENSRNO(	protein_coding
Slc22a13	1.40952	2.65648	0.00821	0.02331	1	ENSRNO(	protein_coding
Abtb1	1.41226	2.66154	5.9E-12	6.2E-11	1.18E-07	ENSRNO(	protein_coding
AABR070	1.41264	2.66225	0.00063	0.00229	1	ENSRNO(	lincRNA
Cyp3a9_1	1.41303	2.66295	0.00033	0.00126	1	ENSRNO(	protein_coding
Lgr4	1.41339	2.66363	1.1E-32	4.9E-31	2.12E-28	ENSRNO(	protein_coding
Prickle1	1.41642	2.66923	5.8E-33	2.7E-31	1.18E-28	ENSRNO(	protein_coding
Srsf12	1.41646	2.66929	0.01131	0.03094	1	ENSRNO(	protein_coding

Rnase4	1.41786	2.67188	7.2E-26	2.3E-24	1.46E-21	ENSRNO0	protein_coding
AABR070	1.41885	2.67373	0.01861	0.04812	1	ENSRNO0	protein_coding
Soga3	1.42052	2.67682	0.00056	0.00206	1	ENSRNO0	protein_coding
Rab3a	1.4207	2.67715	6.5E-07	3.7E-06	0.013051	ENSRNO0	protein_coding
Ppara	1.42123	2.67814	1.1E-06	6.4E-06	0.023138	ENSRNO0	protein_coding
Plcb4	1.42139	2.67843	1E-55	1.1E-53	2.03E-51	ENSRNO0	protein_coding
Cpeb3	1.42188	2.67934	2.7E-09	2.1E-08	5.38E-05	ENSRNO0	protein_coding
Btn1a1	1.42327	2.68192	0.00454	0.01377	1	ENSRNO0	protein_coding
AABR070	1.42403	2.68334	5.8E-11	5.5E-10	1.16E-06	ENSRNO0	lincRNA
Gramd1c	1.42652	2.68798	6E-09	4.5E-08	0.000122	ENSRNO0	protein_coding
Xrra1	1.42795	2.69065	0.01917	0.0494	1	ENSRNO0	protein_coding
Greb1	1.42908	2.69276	5.2E-05	0.00023	1	ENSRNO0	protein_coding
Foxp2	1.42983	2.69416	1.2E-53	1.2E-51	2.37E-49	ENSRNO0	protein_coding
Mzf1	1.43042	2.69526	2E-08	1.4E-07	0.000394	ENSRNO0	protein_coding
Kcnk6	1.43247	2.69908	3.3E-15	4.7E-14	6.67E-11	ENSRNO0	protein_coding
Bsn	1.43349	2.70099	6.7E-06	3.4E-05	0.135339	ENSRNO0	protein_coding
Dnajb4	1.43392	2.7018	3.1E-39	1.9E-37	6.28E-35	ENSRNO0	protein_coding
Meis1	1.43416	2.70224	3.1E-30	1.2E-28	6.18E-26	ENSRNO0	protein_coding
Doc2a	1.43487	2.70359	2E-12	2.2E-11	4.07E-08	ENSRNO0	protein_coding
Acss2	1.43657	2.70676	1.6E-55	1.7E-53	3.29E-51	ENSRNO0	protein_coding
Zfp709l1	1.43718	2.70791	6.1E-15	8.4E-14	1.22E-10	ENSRNO0	protein_coding
RT1-M3-1	1.43772	2.70893	6.3E-24	1.7E-22	1.28E-19	ENSRNO0	protein_coding
Tcaf1	1.44418	2.72107	2.5E-32	1.1E-30	5.13E-28	ENSRNO0	protein_coding
RT1-DMb	1.44424	2.72119	0.00017	0.00068	1	ENSRNO0	protein_coding
Fbxl20	1.44587	2.72428	3.6E-21	8.2E-20	7.3E-17	ENSRNO0	protein_coding
Sema6c	1.4466	2.72565	4.3E-05	0.00019	0.867435	ENSRNO0	protein_coding
Sox6	1.44764	2.72762	4.3E-09	3.3E-08	8.74E-05	ENSRNO0	protein_coding
Me3	1.44983	2.73176	0.00025	0.00097	1	ENSRNO0	protein_coding
Plce1	1.45234	2.73652	7.7E-20	1.5E-18	1.55E-15	ENSRNO0	protein_coding
Slc16a4	1.45238	2.73659	1.1E-07	6.9E-07	0.002194	ENSRNO0	protein_coding
Fam102b	1.45443	2.74048	1.3E-29	5.1E-28	2.66E-25	ENSRNO0	protein_coding
Aldh1l1	1.45833	2.7479	1.5E-06	8.1E-06	0.029805	ENSRNO0	protein_coding
Csf3	1.45837	2.74798	0.00025	0.00098	1	ENSRNO0	protein_coding
Sec14l4	1.45899	2.74916	0.00935	0.02612	1	ENSRNO0	protein_coding
Iqub	1.45921	2.74958	1.9E-05	9E-05	0.385224	ENSRNO0	protein_coding
Rilp	1.46034	2.75173	5.9E-05	0.00026	1	ENSRNO0	protein_coding
LOC4994	1.46208	2.75506	4.6E-05	0.0002	0.934207	ENSRNO0	protein_coding
Ampd3	1.46388	2.75849	1.6E-22	3.9E-21	3.16E-18	ENSRNO0	protein_coding
Dmd	1.46422	2.75915	2.1E-23	5.6E-22	4.27E-19	ENSRNO0	protein_coding
Psmd10	1.46602	2.76259	6.7E-16	1E-14	1.34E-11	ENSRNO0	protein_coding
Pinlyp	1.46678	2.76405	0.0061	0.01799	1	ENSRNO0	protein_coding
Syde2	1.46842	2.76718	1.5E-31	6.4E-30	2.97E-27	ENSRNO0	protein_coding
Smim14	1.46903	2.76835	3.2E-25	9.5E-24	6.39E-21	ENSRNO0	protein_coding
Klhl13	1.46996	2.77014	7.6E-20	1.5E-18	1.53E-15	ENSRNO0	protein_coding
Ccrl2	1.47085	2.77185	5.2E-05	0.00023	1	ENSRNO0	protein_coding
Cemip	1.47087	2.7719	0.00014	0.00056	1	ENSRNO0	protein_coding
Zfp14	1.471	2.77214	0.0104	0.0287	1	ENSRNO0	protein_coding
Ginm1	1.47132	2.77275	1.7E-57	1.9E-55	3.49E-53	ENSRNO0	protein_coding
Mylip	1.47133	2.77278	9.3E-15	1.3E-13	1.87E-10	ENSRNO0	protein_coding

Mras	1.47237	2.77477	3.8E-42	2.5E-40	7.64E-38	ENSRNO(	protein_coding
Car11	1.47266	2.77534	0.01269	0.03432	1	ENSRNO(	protein_coding
Sectm1a	1.47319	2.77636	0.00081	0.00287	1	ENSRNO(	protein_coding
Hapln1	1.47345	2.77686	3.3E-18	6E-17	6.71E-14	ENSRNO(	protein_coding
Antxr1	1.47471	2.77928	6.1E-59	7E-57	1.23E-54	ENSRNO(	protein_coding
Susd5	1.47718	2.78404	6.8E-06	3.4E-05	0.137413	ENSRNO(	protein_coding
C1qtnf6	1.48073	2.7909	7E-17	1.1E-15	1.41E-12	ENSRNO(	protein_coding
Slc35d2	1.48109	2.79159	2.2E-19	4.2E-18	4.42E-15	ENSRNO(	protein_coding
Tlcd2	1.48272	2.79476	0.00229	0.00743	1	ENSRNO(	protein_coding
Zhx3	1.48352	2.79629	1.5E-31	6.6E-30	3.11E-27	ENSRNO(	protein_coding
Ccdc28a	1.48447	2.79813	1.7E-11	1.7E-10	3.41E-07	ENSRNO(	protein_coding
LOC1036	1.48468	2.79855	0.00263	0.00839	1	ENSRNO(	antisense_RNA
Dock10	1.48708	2.80321	6.2E-09	4.7E-08	0.000125	ENSRNO(	processed_transcript
Pgr	1.48867	2.8063	8.8E-30	3.4E-28	1.76E-25	ENSRNO(	protein_coding
Clec12a	1.49014	2.80916	3.3E-07	2E-06	0.00673	ENSRNO(	protein_coding
Sema3b	1.49111	2.81105	9.6E-13	1.1E-11	1.93E-08	ENSRNO(	protein_coding
Idh1	1.49415	2.81699	1.4E-47	1.1E-45	2.77E-43	ENSRNO(	protein_coding
Arl4d	1.49485	2.81834	2.4E-24	6.8E-23	4.88E-20	ENSRNO(	protein_coding
Lrrc39	1.49631	2.82121	2.7E-09	2.1E-08	5.49E-05	ENSRNO(	protein_coding
AABR070	1.49697	2.82249	0.00654	0.01913	1	ENSRNO(	lincRNA
Fcgrt	1.4977	2.82392	1E-25	3.1E-24	2.02E-21	ENSRNO(	protein_coding
Alpk1	1.49862	2.82573	1.1E-15	1.6E-14	2.21E-11	ENSRNO(	protein_coding
RT1-DMb	1.49884	2.82615	0.00021	0.00085	1	ENSRNO(	protein_coding
Spock2	1.50067	2.82975	0.0083	0.02352	1	ENSRNO(	protein_coding
Mertk	1.50168	2.83172	3.6E-11	3.5E-10	7.34E-07	ENSRNO(	protein_coding
Pot1b	1.50175	2.83187	1.4E-10	1.3E-09	2.82E-06	ENSRNO(	protein_coding
Lgalsl	1.50184	2.83204	3.6E-53	3.4E-51	7.19E-49	ENSRNO(	protein_coding
AABR070	1.50403	2.83635	0.00205	0.00671	1	ENSRNO(	lincRNA
A3galt2	1.50787	2.84391	5.5E-62	6.8E-60	1.12E-57	ENSRNO(	protein_coding
Tspan8	1.5087	2.84553	2.4E-10	2.1E-09	4.88E-06	ENSRNO(	protein_coding
Nfkbia	1.51088	2.84984	1.3E-61	1.6E-59	2.67E-57	ENSRNO(	protein_coding
Vamp5	1.51239	2.85283	0.00042	0.00156	1	ENSRNO(	protein_coding
Vcan	1.51241	2.85285	4.3E-12	4.6E-11	8.68E-08	ENSRNO(	protein_coding
LOC6883	1.51549	2.85895	5E-07	2.9E-06	0.009986	ENSRNO(	protein_coding
Col6a4	1.51661	2.86117	0.00507	0.01522	1	ENSRNO(	protein_coding
AC13274(	1.51676	2.86147	0.00602	0.01779	1	ENSRNO(	protein_coding
Itga1	1.51703	2.86201	2.9E-21	6.7E-20	5.89E-17	ENSRNO(	protein_coding
Fn1	1.51804	2.86402	1.7E-25	5.3E-24	3.48E-21	ENSRNO(	protein_coding
Msln	1.51806	2.86406	5E-26	1.6E-24	1.01E-21	ENSRNO(	protein_coding
Eef2k	1.51845	2.86484	4.1E-55	4.2E-53	8.23E-51	ENSRNO(	protein_coding
Ednrb	1.51906	2.86604	0.00187	0.00618	1	ENSRNO(	protein_coding
Ppfia3	1.51932	2.86655	7.9E-05	0.00034	1	ENSRNO(	protein_coding
Cdh16	1.52045	2.86881	0.0008	0.00283	1	ENSRNO(	protein_coding
Tmem178	1.52155	2.871	1.7E-06	9.4E-06	0.03504	ENSRNO(	protein_coding
Dhtkd1_1	1.52229	2.87247	2.4E-29	9.3E-28	4.91E-25	ENSRNO(	protein_coding
Adssl1	1.5242	2.87627	1.5E-05	7.3E-05	0.307651	ENSRNO(	protein_coding
Sesn3	1.5248	2.87747	6.5E-31	2.8E-29	1.32E-26	ENSRNO(	protein_coding
AABR070	1.52503	2.87792	9.1E-05	0.00038	1	ENSRNO(	lincRNA
Spp1	1.52651	2.88089	1.9E-36	1E-34	3.8E-32	ENSRNO(	protein_coding

Krt7	1.53034	2.88853	7.5E-06	3.7E-05	0.151651	ENSRNO(	protein_coding
Cr1l	1.53065	2.88915	6.8E-41	4.3E-39	1.36E-36	ENSRNO(	protein_coding
Foxo1	1.53219	2.89225	6.5E-20	1.3E-18	1.32E-15	ENSRNO(	protein_coding
AABR070	1.53412	2.89612	0.00749	0.02153	1	ENSRNO(	lincRNA
Pdlim4	1.53645	2.9008	9.8E-05	0.00041	1	ENSRNO(	protein_coding
Ptpn18	1.53916	2.90625	0.01384	0.03705	1	ENSRNO(	protein_coding
Atf6	1.54003	2.90801	2.1E-38	1.2E-36	4.18E-34	ENSRNO(	protein_coding
Rtn4rl1	1.54187	2.91173	1.4E-19	2.8E-18	2.85E-15	ENSRNO(	protein_coding
AABR070	1.54294	2.91387	1.1E-17	1.8E-16	2.12E-13	ENSRNO(	lincRNA
Smarca1	1.54361	2.91523	0.00119	0.0041	1	ENSRNO(	protein_coding
LOC1083	1.54464	2.91732	0.00034	0.00131	1	ENSRNO(	protein_coding
Mtmr12	1.54543	2.91892	3.8E-36	2E-34	7.57E-32	ENSRNO(	protein_coding
AABR070	1.54804	2.92419	4.9E-07	2.8E-06	0.009804	ENSRNO(	lincRNA
LOC3003	1.54908	2.9263	0.00239	0.00771	1	ENSRNO(	protein_coding
Zrsr1	1.54931	2.92678	5.2E-25	1.5E-23	1.06E-20	ENSRNO(	protein_coding
Fam214a	1.55108	2.93037	1.9E-21	4.4E-20	3.84E-17	ENSRNO(	protein_coding
Ctsk	1.55491	2.93815	0.00118	0.00406	1	ENSRNO(	protein_coding
Gstt2	1.55816	2.94478	1.4E-10	1.3E-09	2.9E-06	ENSRNO(	protein_coding
Prob1	1.56018	2.9489	0.00847	0.02397	1	ENSRNO(	protein_coding
Sparc	1.56183	2.95228	2.3E-36	1.3E-34	4.66E-32	ENSRNO(	protein_coding
C1qtnf1	1.56421	2.95715	3.8E-66	5.1E-64	7.74E-62	ENSRNO(	protein_coding
Rabac1	1.56534	2.95946	1.6E-29	6.3E-28	3.28E-25	ENSRNO(	protein_coding
LOC2576	1.56934	2.96769	7E-06	3.5E-05	0.140371	ENSRNO(	protein_coding
Gna14	1.56949	2.968	0.01785	0.04639	1	ENSRNO(	protein_coding
Ttc30b	1.57078	2.97065	5.9E-17	9.6E-16	1.18E-12	ENSRNO(	protein_coding
Htra4	1.57482	2.97898	2.7E-07	1.6E-06	0.005447	ENSRNO(	protein_coding
Pdgfrb	1.5767	2.98287	4.9E-30	2E-28	9.81E-26	ENSRNO(	protein_coding
Rn60_7_1	1.57863	2.98685	2.5E-08	1.8E-07	0.000512	ENSRNO(	lincRNA
Tspan12	1.57869	2.98698	0.00015	0.0006	1	ENSRNO(	protein_coding
Pcyox1	1.58292	2.99576	7.1E-28	2.5E-26	1.43E-23	ENSRNO(	protein_coding
Dnah9	1.58332	2.99658	0.00779	0.0223	1	ENSRNO(	protein_coding
Ache	1.58597	3.00209	0.00015	0.00059	1	ENSRNO(	protein_coding
Scn2a	1.58653	3.00326	5.7E-27	1.9E-25	1.16E-22	ENSRNO(	protein_coding
Slc4a4	1.59116	3.01292	5.4E-20	1.1E-18	1.09E-15	ENSRNO(	protein_coding
AABR070	1.59142	3.01345	0.00108	0.00372	1	ENSRNO(	lincRNA
Msi1	1.59477	3.02047	0.01345	0.03615	1	ENSRNO(	protein_coding
Wfdc18	1.596	3.02303	0.00504	0.01512	1	ENSRNO(	protein_coding
Usp53	1.6012	3.03395	2.5E-70	3.7E-68	4.94E-66	ENSRNO(	protein_coding
Snrpn	1.60133	3.03424	0.00113	0.00387	1	ENSRNO(	protein_coding
Epgn	1.60176	3.03514	1.4E-06	7.5E-06	0.027572	ENSRNO(	protein_coding
Fam134b	1.60193	3.03549	6.4E-10	5.4E-09	1.29E-05	ENSRNO(	protein_coding
Hacd4	1.60253	3.03675	1.2E-07	7.8E-07	0.002501	ENSRNO(	protein_coding
Ptpn22	1.60321	3.03819	0.00307	0.00967	1	ENSRNO(	protein_coding
Kcnip4	1.60433	3.04055	5.9E-13	6.8E-12	1.18E-08	ENSRNO(	protein_coding
Pik3ap1	1.60639	3.04489	2.6E-06	1.4E-05	0.051369	ENSRNO(	protein_coding
Pxk	1.60722	3.04664	2E-69	2.8E-67	3.98E-65	ENSRNO(	protein_coding
Sema3e	1.60808	3.04845	2.3E-29	9E-28	4.73E-25	ENSRNO(	protein_coding
Bcl2l11	1.60977	3.05202	3.6E-13	4.3E-12	7.19E-09	ENSRNO(	protein_coding
Vhl	1.61144	3.05557	1.2E-46	9.2E-45	2.39E-42	ENSRNO(	protein_coding

Sec31b	1.61158	3.05586	0.00718	0.02074	1	ENSRNO(	protein_coding
AABR070	1.61188	3.0565	0.00732	0.02109	1	ENSRNO(	protein_coding
Abhd6	1.61604	3.06533	2.6E-37	1.5E-35	5.31E-33	ENSRNO(	protein_coding
LOC6875	1.6176	3.06863	0.0012	0.00412	1	ENSRNO(	protein_coding
Pparg	1.61816	3.06984	1.1E-30	4.6E-29	2.23E-26	ENSRNO(	protein_coding
AABR070	1.61878	3.07115	0.00565	0.0168	1	ENSRNO(	protein_coding
Ccpg1	1.6193	3.07227	3.5E-60	4.1E-58	7.03E-56	ENSRNO(	protein_coding
Ccdc28b	1.62032	3.07443	3.8E-05	0.00017	0.75536	ENSRNO(	protein_coding
Tnxb	1.62058	3.07499	1.2E-27	4.4E-26	2.51E-23	ENSRNO(	processed_transcript
Col6a3	1.62148	3.0769	2.4E-19	4.6E-18	4.8E-15	ENSRNO(	processed_transcript
Jade2	1.62148	3.07691	1.8E-55	1.8E-53	3.55E-51	ENSRNO(	protein_coding
AABR070	1.62216	3.07836	0.00144	0.00486	1	ENSRNO(	lincRNA
Rrn3	1.62279	3.07971	1.2E-40	7.8E-39	2.5E-36	ENSRNO(	protein_coding
LOC1009	1.62336	3.08093	6.7E-14	8.4E-13	1.34E-09	ENSRNO(	protein_coding
Wfikkn2	1.62427	3.08287	0.00142	0.0048	1	ENSRNO(	protein_coding
Esyt3	1.62689	3.08846	1.4E-08	1E-07	0.000288	ENSRNO(	protein_coding
Lvrn	1.62689	3.08847	3.9E-05	0.00018	0.790173	ENSRNO(	protein_coding
Cyp4f17	1.62715	3.08902	6.3E-08	4.1E-07	0.001266	ENSRNO(	protein_coding
LOC6860	1.6277	3.0902	0.00011	0.00044	1	ENSRNO(	protein_coding
Pag1	1.62859	3.09211	1.4E-07	8.5E-07	0.002736	ENSRNO(	protein_coding
Ypel5	1.6297	3.09449	2.1E-23	5.6E-22	4.27E-19	ENSRNO(	protein_coding
Rab40b	1.62997	3.09507	2.8E-05	0.00013	0.55881	ENSRNO(	protein_coding
Tmem136	1.63086	3.09698	0.00156	0.00523	1	ENSRNO(	protein_coding
Clybl	1.6322	3.09986	4.1E-16	6.3E-15	8.31E-12	ENSRNO(	protein_coding
Cnpy4	1.63383	3.10335	1.3E-45	9.7E-44	2.6E-41	ENSRNO(	protein_coding
Siae	1.63468	3.10519	7.6E-28	2.7E-26	1.53E-23	ENSRNO(	protein_coding
Pfkfb3	1.63547	3.10688	2.1E-24	5.9E-23	4.22E-20	ENSRNO(	protein_coding
Fat4	1.63618	3.10841	9.1E-30	3.5E-28	1.83E-25	ENSRNO(	protein_coding
Ugt1a5	1.63822	3.11282	1.7E-76	2.9E-74	3.5E-72	ENSRNO(	protein_coding
Olr1675	1.63893	3.11434	0.00092	0.00322	1	ENSRNO(	protein_coding
ENSRNO	1.63914	3.1148	0.00048	0.00179	1	ENSRNO(	protein_coding
Thsd7a	1.64144	3.11977	0.00408	0.01251	1	ENSRNO(	protein_coding
Mapt	1.64416	3.12566	1.9E-17	3.2E-16	3.88E-13	ENSRNO(	protein_coding
Lpar6	1.64526	3.12804	4.7E-25	1.4E-23	9.54E-21	ENSRNO(	protein_coding
Ecm2	1.65271	3.14424	0.00544	0.01622	1	ENSRNO(	protein_coding
Patj	1.65302	3.14491	9.3E-49	7.9E-47	1.86E-44	ENSRNO(	protein_coding
Anpep	1.65322	3.14535	3.5E-17	5.8E-16	7.03E-13	ENSRNO(	protein_coding
Pold4	1.6535	3.14597	2.4E-13	2.9E-12	4.79E-09	ENSRNO(	protein_coding
Faxdc2	1.65512	3.14949	3E-11	3E-10	6.08E-07	ENSRNO(	protein_coding
RGD1564	1.65586	3.15112	4.7E-13	5.5E-12	9.46E-09	ENSRNO(	lincRNA
Ifitm3	1.65748	3.15465	9.4E-17	1.5E-15	1.89E-12	ENSRNO(	protein_coding
Nynrin	1.66097	3.16229	3.8E-10	3.3E-09	7.7E-06	ENSRNO(	protein_coding
Bckdha	1.66101	3.16237	1.3E-15	1.9E-14	2.57E-11	ENSRNO(	protein_coding
Gng11	1.66252	3.16569	2.3E-14	3.1E-13	4.69E-10	ENSRNO(	protein_coding
Gpsm1	1.6627	3.16609	2.2E-37	1.2E-35	4.34E-33	ENSRNO(	protein_coding
Zp2	1.66292	3.16656	0.00084	0.00298	1	ENSRNO(	protein_coding
Cdc42ep3	1.66396	3.16885	3.3E-72	5.2E-70	6.67E-68	ENSRNO(	protein_coding
AABR070	1.66546	3.17215	0.00155	0.00522	1	ENSRNO(	lincRNA
Pappa2	1.66738	3.17637	6.3E-06	3.2E-05	0.126972	ENSRNO(	protein_coding

Lsr	1.66797	3.17768	9.4E-06	4.6E-05	0.189909	ENSRNO(	protein_coding
Ikbke	1.67036	3.18294	8.8E-29	3.3E-27	1.77E-24	ENSRNO(	protein_coding
Socs1	1.67249	3.18764	3.2E-06	1.7E-05	0.065308	ENSRNO(	protein_coding
Lss	1.67361	3.19012	5.3E-56	5.7E-54	1.06E-51	ENSRNO(	protein_coding
AABR070	1.67681	3.1972	0.00965	0.02687	1	ENSRNO(	lincRNA
Pnrc1	1.67844	3.20082	1.1E-27	3.8E-26	2.19E-23	ENSRNO(	protein_coding
Hbp1	1.67874	3.20147	1.8E-35	9.6E-34	3.71E-31	ENSRNO(	protein_coding
Rapgef4	1.6793	3.20273	3.1E-37	1.7E-35	6.17E-33	ENSRNO(	protein_coding
Apc2	1.68353	3.21212	6.2E-05	0.00027	1	ENSRNO(	protein_coding
Adprhl1	1.68378	3.21269	3.7E-37	2.1E-35	7.39E-33	ENSRNO(	protein_coding
Pls3	1.68392	3.21301	4.5E-36	2.4E-34	9.01E-32	ENSRNO(	protein_coding
Fa2h	1.68451	3.2143	1.6E-06	8.8E-06	0.03273	ENSRNO(	protein_coding
Tcf23	1.6848	3.21496	0.00425	0.01297	1	ENSRNO(	protein_coding
Rgs2	1.68657	3.21891	1.4E-14	1.9E-13	2.8E-10	ENSRNO(	protein_coding
LOC4996	1.68782	3.22169	0.01925	0.04955	1	ENSRNO(	protein_coding
Asb15	1.68818	3.22249	1.6E-10	1.4E-09	3.21E-06	ENSRNO(	protein_coding
Arsg	1.68834	3.22286	3E-29	1.1E-27	5.95E-25	ENSRNO(	protein_coding
Cebpd	1.68865	3.22355	1.2E-15	1.8E-14	2.41E-11	ENSRNO(	protein_coding
Nckap5l	1.68936	3.22513	2.3E-24	6.4E-23	4.6E-20	ENSRNO(	protein_coding
Nos1	1.69717	3.24263	0.00537	0.01603	1	ENSRNO(	protein_coding
LOC3628	1.69858	3.2458	6E-06	3E-05	0.120374	ENSRNO(	protein_coding
Zcchc24	1.69879	3.24628	1.4E-26	4.6E-25	2.79E-22	ENSRNO(	protein_coding
Plekhh2	1.6998	3.24856	3.6E-34	1.8E-32	7.19E-30	ENSRNO(	protein_coding
Rbm24	1.70187	3.25323	0.00011	0.00044	1	ENSRNO(	protein_coding
LOC2576	1.70251	3.25467	1.5E-05	7.1E-05	0.300036	ENSRNO(	protein_coding
Svbp	1.70348	3.25686	4.4E-23	1.1E-21	8.92E-19	ENSRNO(	protein_coding
Ephx1	1.70413	3.25832	2.5E-09	2E-08	5.02E-05	ENSRNO(	protein_coding
Tinagl1	1.70447	3.2591	1.4E-56	1.5E-54	2.85E-52	ENSRNO(	protein_coding
Iqcb1	1.7045	3.25917	2.7E-26	8.9E-25	5.53E-22	ENSRNO(	protein_coding
AABR070	1.70537	3.26112	0.00072	0.00259	1	ENSRNO(	protein_coding
Dram1	1.70912	3.26961	5.1E-77	9.1E-75	1.02E-72	ENSRNO(	protein_coding
AABR070	1.70937	3.27017	6.4E-05	0.00028	1	ENSRNO(	lincRNA
Mansc1	1.71515	3.28332	0.00083	0.00294	1	ENSRNO(	protein_coding
Ago4	1.72054	3.2956	7.7E-08	5E-07	0.001543	ENSRNO(	protein_coding
Npr3	1.72614	3.3084	1.7E-13	2.1E-12	3.42E-09	ENSRNO(	protein_coding
Nckap5	1.72679	3.3099	9.6E-07	5.4E-06	0.019309	ENSRNO(	protein_coding
Slc25a33	1.72742	3.31136	2.4E-32	1.1E-30	4.93E-28	ENSRNO(	protein_coding
LOC1003	1.72767	3.31192	0.00906	0.02541	1	ENSRNO(	protein_coding
Tmco3	1.73146	3.32064	2.2E-08	1.5E-07	0.000449	ENSRNO(	protein_coding
Tle1	1.7344	3.3274	2.3E-50	2.1E-48	4.71E-46	ENSRNO(	protein_coding
Ldb3	1.73554	3.33004	2.5E-15	3.5E-14	4.97E-11	ENSRNO(	protein_coding
Fndc1	1.73804	3.33581	1.4E-10	1.3E-09	2.84E-06	ENSRNO(	protein_coding
Ptn	1.7391	3.33828	2E-14	2.7E-13	4.09E-10	ENSRNO(	protein_coding
Coq8a	1.74106	3.34282	4.2E-07	2.5E-06	0.008556	ENSRNO(	protein_coding
Coro2a	1.74157	3.34398	1.3E-06	7E-06	0.025474	ENSRNO(	protein_coding
Srgap3	1.74242	3.34596	2.1E-27	7.3E-26	4.23E-23	ENSRNO(	protein_coding
Mgst3	1.74389	3.34936	2.4E-78	4.4E-76	4.76E-74	ENSRNO(	protein_coding
Rxrg	1.74564	3.35343	2.2E-05	0.0001	0.44716	ENSRNO(	protein_coding
ENSRNO	1.74732	3.35735	7.8E-24	2.1E-22	1.56E-19	ENSRNO(	protein_coding

Slc9a9	1.74866	3.36046	6.7E-41	4.3E-39	1.34E-36	ENSRNO0	protein_coding
Sh2d4a	1.74912	3.36153	5.1E-29	1.9E-27	1.02E-24	ENSRNO0	protein_coding
Tstd1	1.75031	3.3643	0.001	0.00347	1	ENSRNO0	protein_coding
Wipi1	1.75316	3.37095	3.9E-32	1.7E-30	7.76E-28	ENSRNO0	protein_coding
AABR070	1.75532	3.37601	0.00251	0.00807	1	ENSRNO0	protein_coding
LOC1083	1.7579	3.38205	0.01443	0.03844	1	ENSRNO0	lincRNA
Kctd7	1.7606	3.38839	4E-22	9.7E-21	8.12E-18	ENSRNO0	protein_coding
Dlc1	1.76142	3.39031	6.9E-41	4.4E-39	1.39E-36	ENSRNO0	protein_coding
Nlgn3	1.76143	3.39035	2.3E-05	0.00011	0.461875	ENSRNO0	protein_coding
Ccdc71l	1.76262	3.39313	1.8E-08	1.3E-07	0.000372	ENSRNO0	protein_coding
Mettl7a	1.76464	3.3979	1.2E-17	2.1E-16	2.42E-13	ENSRNO0	protein_coding
AABR070	1.76465	3.39792	0.00676	0.01966	1	ENSRNO0	protein_coding
AABR070	1.76887	3.40787	0.00253	0.00812	1	ENSRNO0	protein_coding
Fbxl7	1.7702	3.41102	4.4E-17	7.3E-16	8.86E-13	ENSRNO0	protein_coding
Tlr6	1.77067	3.41212	6.2E-29	2.3E-27	1.24E-24	ENSRNO0	protein_coding
Mxra7	1.77376	3.41945	0.0038	0.01174	1	ENSRNO0	protein_coding
Cfap69	1.77477	3.42183	4.8E-08	3.2E-07	0.000957	ENSRNO0	protein_coding
Jade1	1.77498	3.42234	2.9E-53	2.8E-51	5.91E-49	ENSRNO0	protein_coding
Fgf11	1.77963	3.43337	0.01048	0.0289	1	ENSRNO0	protein_coding
Irak2	1.78202	3.43908	3.9E-13	4.6E-12	7.88E-09	ENSRNO0	protein_coding
Vps37d	1.7845	3.445	0.00035	0.00133	1	ENSRNO0	protein_coding
Il20rb	1.78458	3.44518	6.2E-14	7.9E-13	1.24E-09	ENSRNO0	protein_coding
Clec1a	1.78622	3.4491	0.00162	0.00541	1	ENSRNO0	protein_coding
ENSRNO	1.78715	3.45133	0.0006	0.00217	1	ENSRNO0	protein_coding
Pcmtd2	1.78837	3.45425	5E-40	3.1E-38	1.02E-35	ENSRNO0	protein_coding
Pde7b	1.78961	3.45721	0.00123	0.0042	1	ENSRNO0	protein_coding
AABR070	1.79162	3.46203	5.1E-06	2.6E-05	0.103008	ENSRNO0	lincRNA
Ppfia2	1.79277	3.46479	0.01618	0.04256	1	ENSRNO0	protein_coding
Rcsd1	1.79335	3.46618	3E-07	1.8E-06	0.006022	ENSRNO0	protein_coding
Rxra	1.79528	3.47083	2.7E-50	2.4E-48	5.49E-46	ENSRNO0	protein_coding
Dhx58	1.79567	3.47176	1.8E-15	2.6E-14	3.65E-11	ENSRNO0	protein_coding
Plpp1	1.80031	3.48294	1.6E-87	3.6E-85	3.22E-83	ENSRNO0	protein_coding
Psme4	1.80418	3.49231	5.6E-49	4.8E-47	1.13E-44	ENSRNO0	protein_coding
Ccdc189	1.80438	3.49278	2E-06	1.1E-05	0.040143	ENSRNO0	protein_coding
Rgs7	1.80469	3.49354	8.6E-13	9.9E-12	1.74E-08	ENSRNO0	protein_coding
P4ha2	1.80537	3.49518	1.6E-57	1.8E-55	3.24E-53	ENSRNO0	protein_coding
Oplah	1.80737	3.50005	1.9E-35	9.9E-34	3.84E-31	ENSRNO0	protein_coding
Ccdc27	1.80799	3.50155	0.00099	0.00346	1	ENSRNO0	protein_coding
Slc31a2	1.80843	3.50262	1.9E-47	1.5E-45	3.82E-43	ENSRNO0	protein_coding
Cxcr4	1.81087	3.50854	0.00661	0.01929	1	ENSRNO0	protein_coding
Gdf6	1.81445	3.51726	1.3E-06	6.9E-06	0.025252	ENSRNO0	protein_coding
Grm1	1.81522	3.51913	5.3E-14	6.8E-13	1.08E-09	ENSRNO0	protein_coding
Cd84	1.81584	3.52064	0.00079	0.00281	1	ENSRNO0	protein_coding
AABR070	1.81674	3.52283	0.01321	0.0356	1	ENSRNO0	lincRNA
Zcchc11	1.81874	3.52772	9.6E-62	1.2E-59	1.93E-57	ENSRNO0	protein_coding
Fhod3	1.81909	3.52859	7.5E-05	0.00032	1	ENSRNO0	protein_coding
RGD1564	1.8232	3.53866	2E-05	9.4E-05	0.40571	ENSRNO0	protein_coding
Plxdc2	1.82742	3.54902	1.1E-18	2E-17	2.21E-14	ENSRNO0	protein_coding
Sh3bgr	1.82952	3.55418	0.01303	0.03516	1	ENSRNO0	protein_coding

Adamtsl4	1.83163	3.5594	3.3E-76	5.5E-74	6.67E-72	ENSRNO(	protein_coding
Cebpb	1.83249	3.56151	4.8E-20	9.9E-19	9.73E-16	ENSRNO(	protein_coding
Acadsb	1.83658	3.57163	3E-32	1.3E-30	6.05E-28	ENSRNO(	protein_coding
Nkx3-2	1.83869	3.57684	0.00892	0.02504	1	ENSRNO(	protein_coding
Epb41l5	1.84071	3.58186	1E-18	1.9E-17	2.04E-14	ENSRNO(	protein_coding
Zfr2	1.84223	3.58564	2.3E-06	1.2E-05	0.045858	ENSRNO(	protein_coding
Csgalnact	1.84534	3.59338	1.3E-41	8.4E-40	2.55E-37	ENSRNO(	protein_coding
Numbl	1.84645	3.59614	2.9E-23	7.5E-22	5.84E-19	ENSRNO(	protein_coding
Edar	1.84681	3.59703	0.0003	0.00115	1	ENSRNO(	protein_coding
Tepp	1.84731	3.59828	0.01157	0.03159	1	ENSRNO(	protein_coding
AABR070	1.84769	3.59924	0.01095	0.03008	1	ENSRNO(	protein_coding
LOC1025	1.8484	3.601	0.00037	0.0014	1	ENSRNO(	protein_coding
B4gat1	1.85337	3.61344	9.1E-50	8E-48	1.84E-45	ENSRNO(	protein_coding
AC114343	1.85452	3.61631	9E-05	0.00038	1	ENSRNO(	protein_coding
Crebrf	1.85612	3.62033	1.7E-35	8.6E-34	3.32E-31	ENSRNO(	protein_coding
Tlr10	1.85661	3.62155	0.00067	0.00241	1	ENSRNO(	protein_coding
AABR070	1.85829	3.62578	0.00134	0.00455	1	ENSRNO(	protein_coding
Nudt4	1.8613	3.63334	3E-76	5.1E-74	6.07E-72	ENSRNO(	protein_coding
Alcam	1.86655	3.64661	1.8E-98	5.1E-96	3.68E-94	ENSRNO(	protein_coding
Nhs	1.86714	3.64808	1.3E-44	9.6E-43	2.7E-40	ENSRNO(	protein_coding
Acy3	1.86731	3.64851	0.00042	0.00159	1	ENSRNO(	protein_coding
AABR070	1.87009	3.65556	0.00457	0.01386	1	ENSRNO(	lincRNA
LOC5007	1.87014	3.65569	5.7E-23	1.4E-21	1.14E-18	ENSRNO(	protein_coding
Ces2c	1.8707	3.65709	0.00015	0.00061	1	ENSRNO(	protein_coding
Ppargc1a	1.87554	3.6694	1.2E-45	9.2E-44	2.47E-41	ENSRNO(	protein_coding
AABR070	1.87653	3.6719	0.00877	0.02467	1	ENSRNO(	lincRNA
Eppin	1.88156	3.68474	7.9E-08	5.1E-07	0.001597	ENSRNO(	protein_coding
F11r	1.88457	3.69242	1.7E-69	2.5E-67	3.49E-65	ENSRNO(	protein_coding
Rnf122	1.88519	3.69402	7.9E-12	8.3E-11	1.59E-07	ENSRNO(	protein_coding
Aqp11	1.88572	3.69538	1.1E-06	6.4E-06	0.02312	ENSRNO(	protein_coding
Col4a4	1.88682	3.69819	2.4E-13	2.9E-12	4.81E-09	ENSRNO(	protein_coding
Emb	1.88743	3.69976	1.5E-77	2.7E-75	3.05E-73	ENSRNO(	protein_coding
Rerg	1.89025	3.70701	9.6E-05	0.0004	1	ENSRNO(	protein_coding
Plekha7	1.89213	3.71182	1E-24	2.9E-23	2.04E-20	ENSRNO(	protein_coding
Ctsc	1.89657	3.72328	4E-31	1.7E-29	8.03E-27	ENSRNO(	protein_coding
RGD1560	1.89672	3.72367	0.00024	0.00096	1	ENSRNO(	protein_coding
LOC1009	1.90018	3.7326	0.0138	0.03696	1	ENSRNO(	protein_coding
AABR070	1.9004	3.73316	0.01341	0.03606	1	ENSRNO(	pseudogene
Slc35f3	1.90328	3.74064	0.00898	0.02522	1	ENSRNO(	protein_coding
Inpp5k	1.90343	3.74101	1.5E-88	3.5E-86	3.02E-84	ENSRNO(	protein_coding
Trpt1	1.90355	3.74133	0.01754	0.04569	1	ENSRNO(	protein_coding
Nfil3	1.90676	3.74965	7.9E-43	5.4E-41	1.59E-38	ENSRNO(	protein_coding
Gpr156	1.90684	3.74986	1.2E-06	6.5E-06	0.023692	ENSRNO(	protein_coding
Nrbp2	1.90688	3.74998	4.3E-11	4.2E-10	8.73E-07	ENSRNO(	protein_coding
Acpp	1.90753	3.75167	9.2E-49	7.9E-47	1.85E-44	ENSRNO(	protein_coding
Sult1d1	1.90813	3.75322	4.7E-07	2.7E-06	0.009372	ENSRNO(	protein_coding
Aqp7	1.90815	3.75328	0.01592	0.04193	1	ENSRNO(	protein_coding
AABR070	1.91137	3.76166	0.00637	0.01869	1	ENSRNO(	lincRNA
Edem1	1.91204	3.76342	1.9E-66	2.6E-64	3.86E-62	ENSRNO(	protein_coding

Bex2	1.91244	3.76445	0.01016	0.0281	1	ENSRNO(	protein_coding
Ccdc171	1.91306	3.76606	2E-13	2.5E-12	4.11E-09	ENSRNO(	processed_transcript
Cxxc4	1.91504	3.77125	7.9E-09	5.8E-08	0.00016	ENSRNO(	protein_coding
Paqr6	1.9161	3.77402	4.3E-15	6.1E-14	8.74E-11	ENSRNO(	protein_coding
Rn50_13_	1.9182	3.77951	1.1E-72	1.7E-70	2.15E-68	ENSRNO(	lincRNA
AABR070	1.91921	3.78216	0.00127	0.00435	1	ENSRNO(	protein_coding
Lpin1	1.92071	3.7861	1.3E-29	5E-28	2.61E-25	ENSRNO(	protein_coding
Arrdc2	1.92157	3.78836	2E-21	4.6E-20	4.03E-17	ENSRNO(	protein_coding
Ypel2	1.92218	3.78994	2.7E-30	1.1E-28	5.46E-26	ENSRNO(	protein_coding
Plpp3	1.92401	3.79477	5.2E-19	9.8E-18	1.06E-14	ENSRNO(	protein_coding
Gbp2	1.92531	3.79819	4.1E-49	3.6E-47	8.33E-45	ENSRNO(	protein_coding
Slc12a1	1.93093	3.81301	4.6E-07	2.7E-06	0.009251	ENSRNO(	protein_coding
Slc2a5	1.93264	3.81752	9.1E-42	6.1E-40	1.84E-37	ENSRNO(	protein_coding
AC13475(	1.93527	3.8245	0.0079	0.02259	1	ENSRNO(	lincRNA
Hfe2	1.94176	3.84175	8.2E-05	0.00035	1	ENSRNO(	protein_coding
Sgk1	1.9451	3.85065	9.3E-58	1E-55	1.87E-53	ENSRNO(	protein_coding
Arhgef25	1.9516	3.86804	1.4E-19	2.8E-18	2.88E-15	ENSRNO(	protein_coding
Snx24	1.95177	3.86849	1.5E-94	3.9E-92	3.03E-90	ENSRNO(	protein_coding
Rubcnl	1.95591	3.87961	0.00047	0.00173	1	ENSRNO(	protein_coding
Pdha1l1	1.95647	3.88112	4.3E-28	1.5E-26	8.59E-24	ENSRNO(	protein_coding
Dyrk1b	1.95763	3.88423	1.7E-22	4.2E-21	3.47E-18	ENSRNO(	protein_coding
Ces1f	1.96663	3.90854	0.00193	0.00635	1	ENSRNO(	protein_coding
Rn60_16_	1.96919	3.91549	1.2E-32	5.7E-31	2.49E-28	ENSRNO(	unprocessed_pseudogene
Pink1	1.9719	3.92284	6.8E-44	4.8E-42	1.37E-39	ENSRNO(	protein_coding
Art3	1.97205	3.92326	5.3E-05	0.00023	1	ENSRNO(	protein_coding
Foxo3	1.97515	3.93168	4.8E-48	4E-46	9.62E-44	ENSRNO(	protein_coding
Olr907	1.97562	3.93297	0.00626	0.0184	1	ENSRNO(	pseudogene
Col12a1	1.97632	3.93487	5.9E-55	6E-53	1.18E-50	ENSRNO(	protein_coding
Cxcl17	1.97664	3.93575	1.5E-06	8.1E-06	0.029722	ENSRNO(	protein_coding
Fkbp14	1.97815	3.93987	5E-60	5.8E-58	1.02E-55	ENSRNO(	protein_coding
Gap43	1.98097	3.94758	2.1E-06	1.1E-05	0.04159	ENSRNO(	protein_coding
Ehhadh	1.98155	3.94916	0.00011	0.00046	1	ENSRNO(	protein_coding
Npr2	1.985	3.95863	6.3E-09	4.7E-08	0.000126	ENSRNO(	protein_coding
Sorcs1	1.98953	3.97109	1.3E-41	8.4E-40	2.56E-37	ENSRNO(	protein_coding
AABR070	1.9907	3.9743	0.00047	0.00173	1	ENSRNO(	protein_coding
Txk	1.99085	3.97471	2.1E-19	4E-18	4.15E-15	ENSRNO(	protein_coding
Lamb2	1.99223	3.97852	5.1E-57	5.6E-55	1.03E-52	ENSRNO(	protein_coding
Slc23a1	1.99627	3.98968	0.008	0.02281	1	ENSRNO(	protein_coding
Hpse	1.99781	3.99394	4.1E-57	4.5E-55	8.3E-53	ENSRNO(	protein_coding
RGD1559	1.9982	3.99502	1.2E-06	6.4E-06	0.02332	ENSRNO(	protein_coding
Gab2	2.00012	4.00033	2.5E-36	1.3E-34	4.98E-32	ENSRNO(	protein_coding
Fmo4	2.00084	4.00233	2.1E-22	5E-21	4.18E-18	ENSRNO(	protein_coding
Dusp13_1	2.00229	4.00636	4.7E-06	2.4E-05	0.094979	ENSRNO(	protein_coding
Hipk1	2.0061	4.01696	9.3E-48	7.7E-46	1.86E-43	ENSRNO(	protein_coding
AABR070	2.00695	4.01931	1.5E-23	4E-22	3.04E-19	ENSRNO(	protein_coding
NEWGEN	2.01225	4.03412	0.00271	0.00864	1	ENSRNO(	protein_coding
Tbx22	2.0192	4.05359	0.00139	0.0047	1	ENSRNO(	protein_coding
Stard8	2.01925	4.05374	0.01014	0.02806	1	ENSRNO(	protein_coding
Nacad	2.0203	4.05668	4.9E-10	4.2E-09	9.86E-06	ENSRNO(	protein_coding

Trpv4	2.02079	4.05806	0.0011	0.0038	1	ENSRNO(	protein_coding
AABR070	2.02125	4.05936	1.2E-05	5.9E-05	0.245631	ENSRNO(	protein_coding
Ang2	2.02621	4.07333	0.00201	0.00659	1	ENSRNO(	protein_coding
ENSRNO	2.03033	4.08497	0.0007	0.00253	1	ENSRNO(	protein_coding
Gabarapl1	2.03104	4.08699	4.6E-73	7.4E-71	9.24E-69	ENSRNO(	protein_coding
Arid3a	2.03246	4.09103	4.7E-12	5E-11	9.52E-08	ENSRNO(	protein_coding
AABR070	2.03386	4.09498	4.8E-20	9.9E-19	9.67E-16	ENSRNO(	protein_coding
LOC1009	2.04071	4.11447	0.00849	0.024	1	ENSRNO(	protein_coding
Rn60_1_2	2.04466	4.12576	7.2E-22	1.7E-20	1.46E-17	ENSRNO(	lincRNA
Dcxr	2.04789	4.135	0.00245	0.0079	1	ENSRNO(	protein_coding
AABR070	2.04983	4.14058	0.0001	0.00043	1	ENSRNO(	lincRNA
RGD1560	2.05264	4.14864	0.01455	0.03873	1	ENSRNO(	protein_coding
Ankrd44	2.05286	4.14927	9.2E-48	7.7E-46	1.85E-43	ENSRNO(	protein_coding
Nptx1	2.05965	4.16886	0.01369	0.03671	1	ENSRNO(	protein_coding
Dact2	2.06098	4.17269	0.00101	0.0035	1	ENSRNO(	protein_coding
Nipal1	2.0616	4.17448	2.2E-54	2.2E-52	4.33E-50	ENSRNO(	protein_coding
Adamtsl3	2.06406	4.18161	8E-107	2E-104	1.5E-102	ENSRNO(	protein_coding
Acad11	2.06467	4.18339	4E-112	1E-109	7.5E-108	ENSRNO(	protein_coding
Elmo1	2.06652	4.18874	8.6E-48	7.2E-46	1.74E-43	ENSRNO(	protein_coding
AABR070	2.06703	4.19023	7.8E-05	0.00033	1	ENSRNO(	protein_coding
Nupr1	2.06727	4.19092	2.5E-50	2.3E-48	5.06E-46	ENSRNO(	protein_coding
Tnfrsf25	2.07042	4.2001	1E-07	6.7E-07	0.002107	ENSRNO(	protein_coding
AABR070	2.07145	4.20308	0.00196	0.00644	1	ENSRNO(	lincRNA
Mslnl	2.07755	4.22089	0.00276	0.00878	1	ENSRNO(	protein_coding
Ptprq	2.08181	4.23337	1.5E-23	4.1E-22	3.08E-19	ENSRNO(	protein_coding
Txnip	2.0825	4.23539	6.6E-21	1.5E-19	1.33E-16	ENSRNO(	protein_coding
Gstm7	2.0872	4.24924	1.1E-56	1.2E-54	2.19E-52	ENSRNO(	protein_coding
LOC6885	2.09356	4.26801	7.3E-07	4.2E-06	0.014664	ENSRNO(	protein_coding
Zfp473	2.09395	4.26914	2.3E-07	1.4E-06	0.00458	ENSRNO(	protein_coding
AABR070	2.09677	4.2775	1.4E-07	8.8E-07	0.002831	ENSRNO(	lincRNA
Xirp1	2.10016	4.28757	2.8E-05	0.00013	0.565278	ENSRNO(	protein_coding
ENSRNO	2.10073	4.28926	6.8E-55	6.9E-53	1.37E-50	ENSRNO(	protein_coding
AABR070	2.10597	4.30487	3.2E-06	1.7E-05	0.064008	ENSRNO(	lincRNA
Errfi1	2.10678	4.30728	1.3E-54	1.3E-52	2.69E-50	ENSRNO(	protein_coding
Map3k8	2.10745	4.30928	1.5E-32	6.9E-31	3.07E-28	ENSRNO(	protein_coding
Aldh3a1	2.10832	4.31188	4E-23	1E-21	8.14E-19	ENSRNO(	protein_coding
Mpp2	2.11114	4.32032	0.00505	0.01515	1	ENSRNO(	protein_coding
Phykpl	2.11177	4.32222	0.00101	0.00352	1	ENSRNO(	protein_coding
Tnfsf18	2.11448	4.33035	2E-08	1.4E-07	0.0004	ENSRNO(	protein_coding
Scn1a	2.11488	4.33154	2.8E-20	5.8E-19	5.59E-16	ENSRNO(	protein_coding
Mturn	2.12171	4.35211	9.4E-18	1.6E-16	1.9E-13	ENSRNO(	protein_coding
Zfp36	2.12457	4.36072	1.1E-63	1.4E-61	2.3E-59	ENSRNO(	protein_coding
Gpr146	2.12692	4.36783	1.1E-51	1E-49	2.25E-47	ENSRNO(	protein_coding
AABR070	2.127	4.36809	0.00804	0.02291	1	ENSRNO(	protein_coding
Clip1	2.12872	4.3733	1.1E-67	1.5E-65	2.18E-63	ENSRNO(	protein_coding
Pdcd4	2.13076	4.37947	1.2E-56	1.3E-54	2.33E-52	ENSRNO(	protein_coding
Npvf	2.13201	4.38328	0.01924	0.04955	1	ENSRNO(	protein_coding
Iqch	2.13283	4.38578	1.7E-05	7.9E-05	0.33682	ENSRNO(	protein_coding
Dclk1	2.1375	4.39999	3E-28	1.1E-26	5.97E-24	ENSRNO(	protein_coding

Golm1	2.13781	4.40094	1.4E-19	2.8E-18	2.82E-15	ENSRNO(	protein_coding
Rnf128	2.13871	4.40369	0.00047	0.00175	1	ENSRNO(	protein_coding
Sdc4	2.14575	4.42523	8.1E-69	1.1E-66	1.63E-64	ENSRNO(	protein_coding
AABR070	2.14737	4.43021	9.7E-06	4.7E-05	0.194772	ENSRNO(	lincRNA
AABR070	2.14935	4.43628	0.005	0.01503	1	ENSRNO(	lincRNA
Xirp2	2.15164	4.44332	0.00621	0.01827	1	ENSRNO(	protein_coding
Pla1a	2.1531	4.44784	0.00553	0.01646	1	ENSRNO(	protein_coding
Extl1	2.17005	4.50039	3.9E-07	2.3E-06	0.007755	ENSRNO(	protein_coding
Ypel3	2.17035	4.50131	8.3E-29	3.1E-27	1.68E-24	ENSRNO(	protein_coding
LOC1003	2.17197	4.5064	2.7E-05	0.00013	0.553554	ENSRNO(	protein_coding
Cebpa	2.17453	4.51438	1.8E-46	1.4E-44	3.58E-42	ENSRNO(	protein_coding
LOC1083	2.17528	4.51673	1.7E-47	1.4E-45	3.47E-43	ENSRNO(	protein_coding
Slc25a48	2.17548	4.51736	1.8E-30	7.4E-29	3.62E-26	ENSRNO(	protein_coding
Dtx4	2.17818	4.52582	1.6E-18	3E-17	3.29E-14	ENSRNO(	protein_coding
Upk3bl	2.17828	4.52614	0.01551	0.04098	1	ENSRNO(	protein_coding
Nsun7	2.18151	4.53628	7.3E-06	3.6E-05	0.147324	ENSRNO(	protein_coding
Cdnf	2.1844	4.54539	2.5E-10	2.2E-09	4.97E-06	ENSRNO(	protein_coding
Wisp2	2.18695	4.55341	9.1E-97	2.5E-94	1.83E-92	ENSRNO(	protein_coding
Mroh2a	2.1877	4.5558	8.1E-20	1.6E-18	1.63E-15	ENSRNO(	protein_coding
Znrf2	2.1881	4.55704	1.4E-67	1.9E-65	2.86E-63	ENSRNO(	protein_coding
Angpt1	2.18812	4.55711	8.3E-25	2.4E-23	1.68E-20	ENSRNO(	protein_coding
Sulf2	2.20379	4.60687	1.5E-12	1.7E-11	2.98E-08	ENSRNO(	protein_coding
Cblb	2.20489	4.6104	3.1E-86	7E-84	6.3E-82	ENSRNO(	protein_coding
Grtp1_1	2.21017	4.62729	3.6E-70	5.4E-68	7.34E-66	ENSRNO(	protein_coding
Il17re	2.21042	4.62809	1.1E-45	8.5E-44	2.27E-41	ENSRNO(	protein_coding
Boc	2.21073	4.6291	3.8E-05	0.00017	0.763889	ENSRNO(	protein_coding
Pik3c2a	2.21228	4.63406	5.3E-85	1.1E-82	1.06E-80	ENSRNO(	protein_coding
Pc	2.21954	4.65744	2.6E-91	6.5E-89	5.22E-87	ENSRNO(	protein_coding
Gucy2g	2.22048	4.66049	0.00017	0.0007	1	ENSRNO(	protein_coding
Slc25a54	2.22075	4.66135	0.0047	0.01419	1	ENSRNO(	protein_coding
Tspan17	2.22181	4.66479	3.1E-21	7.1E-20	6.25E-17	ENSRNO(	protein_coding
Calcoco1	2.22223	4.66616	5.1E-42	3.4E-40	1.03E-37	ENSRNO(	protein_coding
AABR070	2.2299	4.69102	0.01858	0.04805	1	ENSRNO(	lincRNA
Kidins220	2.23497	4.70754	5.2E-76	8.6E-74	1.05E-71	ENSRNO(	protein_coding
Slitrk6	2.25038	4.75808	3.3E-21	7.6E-20	6.74E-17	ENSRNO(	protein_coding
Tnr	2.25407	4.77026	1.1E-10	1E-09	2.28E-06	ENSRNO(	protein_coding
Slc28a2	2.25557	4.77524	8.3E-21	1.8E-19	1.67E-16	ENSRNO(	protein_coding
Irs3	2.2593	4.78759	1.5E-09	1.2E-08	2.93E-05	ENSRNO(	protein_coding
Gp1ba	2.26032	4.79096	0.00038	0.00144	1	ENSRNO(	protein_coding
LOC5003	2.26442	4.8046	9.2E-40	5.6E-38	1.86E-35	ENSRNO(	protein_coding
Il6r	2.26618	4.81047	3.7E-65	4.7E-63	7.41E-61	ENSRNO(	protein_coding
Asb9	2.26687	4.81276	9E-11	8.3E-10	1.81E-06	ENSRNO(	protein_coding
Loxl3	2.26951	4.82159	2E-79	3.8E-77	4.04E-75	ENSRNO(	protein_coding
Sphk1	2.2714	4.82791	1.2E-45	8.8E-44	2.35E-41	ENSRNO(	protein_coding
Kng1	2.27275	4.83242	3.7E-07	2.2E-06	0.007414	ENSRNO(	protein_coding
Tmem150	2.27314	4.83375	5E-105	2E-102	1.1E-100	ENSRNO(	protein_coding
Ifi47	2.28158	4.86209	8.1E-06	4E-05	0.163669	ENSRNO(	protein_coding
Impg2	2.28352	4.86865	0.00045	0.00168	1	ENSRNO(	protein_coding
AABR070	2.28428	4.87119	8E-08	5.1E-07	0.001604	ENSRNO(	protein_coding

Plod2	2.2881	4.88413	5.9E-73	9.4E-71	1.18E-68	ENSRNO(	protein_coding
AABR070	2.29799	4.91773	0.00499	0.015	1	ENSRNO(	pseudogene
Gabra3	2.29996	4.92445	8.2E-19	1.5E-17	1.66E-14	ENSRNO(	protein_coding
Itga10	2.30581	4.94444	5.8E-62	7E-60	1.16E-57	ENSRNO(	protein_coding
Mrc1	2.30685	4.94803	7.5E-05	0.00032	1	ENSRNO(	protein_coding
Cryab	2.30694	4.94834	5E-126	2E-123	1.1E-121	ENSRNO(	protein_coding
Slc29a2	2.30706	4.94875	3.8E-29	1.4E-27	7.72E-25	ENSRNO(	protein_coding
Zfp583	2.30861	4.95405	0.0109	0.02994	1	ENSRNO(	protein_coding
Mcc_2	2.31326	4.97003	3E-107	9E-105	5.3E-103	ENSRNO(	protein_coding
Pde1b	2.32226	5.00113	3.2E-08	2.2E-07	0.00064	ENSRNO(	protein_coding
Pou2f3	2.32299	5.00368	0.01622	0.04264	1	ENSRNO(	protein_coding
Adh1	2.32325	5.00459	9.2E-60	1.1E-57	1.86E-55	ENSRNO(	protein_coding
Clca4	2.32699	5.01757	6.7E-09	5E-08	0.000134	ENSRNO(	protein_coding
Dusp1	2.33388	5.04159	1E-143	8E-141	2.5E-139	ENSRNO(	protein_coding
Mir199a2_	2.336	5.04901	1E-06	5.6E-06	0.020153	ENSRNO(	protein_coding
LOC6917	2.33771	5.05501	0.01628	0.04278	1	ENSRNO(	protein_coding
Serpinb11	2.34485	5.08006	2E-06	1.1E-05	0.040405	ENSRNO(	protein_coding
Arr3	2.34549	5.08234	0.00047	0.00175	1	ENSRNO(	protein_coding
RGD1309	2.34774	5.09025	3.4E-86	7.6E-84	6.87E-82	ENSRNO(	protein_coding
Tmod2	2.349	5.09471	1.2E-05	5.9E-05	0.246771	ENSRNO(	protein_coding
Ikzf2	2.34968	5.09713	3.9E-14	5.1E-13	7.9E-10	ENSRNO(	protein_coding
LOC1083	2.35007	5.09848	7E-66	9.3E-64	1.42E-61	ENSRNO(	protein_coding
Mst1	2.35485	5.1154	6.9E-05	0.0003	1	ENSRNO(	protein_coding
Phactr2	2.35805	5.12676	0.00077	0.00274	1	ENSRNO(	protein_coding
Scarf1	2.3645	5.14975	0.00303	0.00956	1	ENSRNO(	protein_coding
Tmem38b	2.36757	5.16071	1E-142	1E-139	3E-138	ENSRNO(	protein_coding
Cys1	2.36908	5.1661	2.5E-12	2.8E-11	5.1E-08	ENSRNO(	protein_coding
Rasal3	2.37105	5.17317	0.01382	0.03701	1	ENSRNO(	protein_coding
Psd3	2.37704	5.19471	2.8E-47	2.2E-45	5.55E-43	ENSRNO(	protein_coding
Nhsl2	2.38012	5.20579	1.1E-33	5.4E-32	2.26E-29	ENSRNO(	processed_transcript
Hipk2	2.38161	5.2112	9E-66	1.2E-63	1.82E-61	ENSRNO(	protein_coding
Sgcg	2.38201	5.21264	3.5E-54	3.5E-52	7E-50	ENSRNO(	protein_coding
Fat3	2.38231	5.21372	1E-148	1E-145	2.3E-144	ENSRNO(	protein_coding
Syt13	2.38665	5.22943	1.2E-48	1E-46	2.46E-44	ENSRNO(	protein_coding
Ush1c	2.38912	5.23836	0.00733	0.02111	1	ENSRNO(	protein_coding
Tmprss6	2.39076	5.24432	0.01145	0.03127	1	ENSRNO(	protein_coding
Atp1b1	2.39247	5.25057	1.1E-88	2.5E-86	2.15E-84	ENSRNO(	protein_coding
Cyp2d4	2.39983	5.27741	6.6E-05	0.00029	1	ENSRNO(	protein_coding
Rnf207	2.40111	5.28208	0.00016	0.00066	1	ENSRNO(	protein_coding
Clvs2	2.40601	5.30007	1.1E-05	5.2E-05	0.213962	ENSRNO(	protein_coding
Anxa3	2.40697	5.30358	2.2E-89	5.3E-87	4.37E-85	ENSRNO(	protein_coding
Itpr2	2.40979	5.31398	6.2E-31	2.6E-29	1.25E-26	ENSRNO(	protein_coding
N4bp2l1	2.41572	5.33586	5.3E-11	5.1E-10	1.08E-06	ENSRNO(	protein_coding
Angptl4	2.42085	5.35487	1.8E-09	1.5E-08	3.72E-05	ENSRNO(	protein_coding
Per1	2.4245	5.36843	2E-145	1E-142	3.3E-141	ENSRNO(	protein_coding
Lrrn4cl	2.42537	5.37166	9.6E-32	4.2E-30	1.93E-27	ENSRNO(	protein_coding
Cxcl13	2.42696	5.37759	4.7E-05	0.00021	0.952746	ENSRNO(	protein_coding
AC128830	2.42805	5.38167	0.00797	0.02275	1	ENSRNO(	lincRNA
Mr1	2.4292	5.38595	6.5E-16	9.8E-15	1.3E-11	ENSRNO(	protein_coding

Matn2	2.44448	5.4433	0.00684	0.01988	1	ENSRNO(	protein_coding
Abcb9	2.44676	5.4519	3.3E-05	0.00015	0.673989	ENSRNO(	protein_coding
Fgd4	2.44988	5.4637	0.00428	0.01308	1	ENSRNO(	protein_coding
Itgb4	2.45291	5.47521	7.2E-18	1.3E-16	1.46E-13	ENSRNO(	protein_coding
Atg4a	2.45324	5.47646	4.5E-83	8.9E-81	9.09E-79	ENSRNO(	protein_coding
Tshr	2.46424	5.51838	7.5E-09	5.6E-08	0.000151	ENSRNO(	protein_coding
AABR070	2.46435	5.51878	3.7E-06	1.9E-05	0.074176	ENSRNO(	lincRNA
Wwp1	2.47115	5.54486	2.8E-61	3.3E-59	5.65E-57	ENSRNO(	protein_coding
Cfap70	2.4739	5.55544	2.7E-12	2.9E-11	5.38E-08	ENSRNO(	protein_coding
Slc2a12	2.48072	5.58177	4.3E-16	6.6E-15	8.64E-12	ENSRNO(	protein_coding
Ech1	2.48116	5.58348	1E-94	2.8E-92	2.11E-90	ENSRNO(	protein_coding
Dixdc1	2.48146	5.58463	1E-50	9.6E-49	2.11E-46	ENSRNO(	protein_coding
AABR070	2.48241	5.58832	1.2E-05	5.7E-05	0.237804	ENSRNO(	lincRNA
RT1-S3	2.48365	5.59309	2.6E-11	2.5E-10	5.16E-07	ENSRNO(	protein_coding
Ppp1r3c	2.48369	5.59325	9.9E-29	3.7E-27	2E-24	ENSRNO(	protein_coding
Th	2.48696	5.60594	4.7E-14	6.1E-13	9.51E-10	ENSRNO(	protein_coding
AABR070	2.49713	5.64562	8.2E-07	4.6E-06	0.016477	ENSRNO(	protein_coding
RGD1563	2.5001	5.65725	0.00401	0.01232	1	ENSRNO(	protein_coding
Mgp	2.50099	5.66072	4.5E-16	6.9E-15	9.05E-12	ENSRNO(	protein_coding
Cmah	2.50388	5.67208	1.6E-08	1.1E-07	0.000315	ENSRNO(	protein_coding
Cped1	2.50725	5.68537	1.3E-76	2.3E-74	2.66E-72	ENSRNO(	protein_coding
Angpt4	2.51108	5.70046	1.7E-19	3.3E-18	3.45E-15	ENSRNO(	protein_coding
C1qtnf5	2.51224	5.70507	1.5E-17	2.6E-16	3.02E-13	ENSRNO(	protein_coding
Gbp3	2.51715	5.72451	5.4E-10	4.6E-09	1.09E-05	ENSRNO(	protein_coding
Hspb2	2.51734	5.72526	5E-24	1.4E-22	1E-19	ENSRNO(	protein_coding
Pdk4	2.5175	5.72589	1.2E-06	6.5E-06	0.023591	ENSRNO(	protein_coding
Lox	2.5181	5.72829	5.1E-96	1.4E-93	1.02E-91	ENSRNO(	protein_coding
Htr1b	2.51893	5.73158	1.4E-18	2.5E-17	2.8E-14	ENSRNO(	protein_coding
Mx1	2.52366	5.7504	3.6E-12	3.9E-11	7.34E-08	ENSRNO(	protein_coding
AABR070	2.52456	5.75397	1.6E-25	5E-24	3.28E-21	ENSRNO(	pseudogene
Scd2	2.52771	5.76657	7.3E-64	9.3E-62	1.47E-59	ENSRNO(	protein_coding
Gas6	2.53712	5.80429	2.5E-16	4E-15	5.13E-12	ENSRNO(	protein_coding
Fam43a	2.53716	5.80445	3.5E-70	5.2E-68	7.08E-66	ENSRNO(	protein_coding
AABR070	2.5401	5.81629	0.01938	0.04983	1	ENSRNO(	pseudogene
Abcc2	2.54333	5.82931	0.0007	0.00252	1	ENSRNO(	protein_coding
Tnfrsf11b	2.54509	5.83645	4.7E-47	3.8E-45	9.49E-43	ENSRNO(	protein_coding
Ckmt2	2.55265	5.8671	0.01907	0.04915	1	ENSRNO(	protein_coding
Ctxn3	2.55627	5.88185	1.9E-11	1.9E-10	3.79E-07	ENSRNO(	protein_coding
Calml4	2.56206	5.90552	5E-41	3.2E-39	1.02E-36	ENSRNO(	protein_coding
Lrrc31	2.56234	5.90666	0.00163	0.00544	1	ENSRNO(	protein_coding
Prodh1	2.5665	5.9237	3.6E-10	3.1E-09	7.3E-06	ENSRNO(	protein_coding
Serpinb7	2.56665	5.92431	1.2E-71	1.9E-69	2.47E-67	ENSRNO(	protein_coding
Slc45a3	2.57322	5.95136	0.00449	0.01364	1	ENSRNO(	protein_coding
Cav3	2.57725	5.96801	0.00055	0.00203	1	ENSRNO(	protein_coding
Ablim1	2.58078	5.98261	6E-113	2E-110	1.2E-108	ENSRNO(	protein_coding
Spic	2.58459	5.99845	0.0116	0.03166	1	ENSRNO(	protein_coding
Coro6	2.58675	6.00745	2.2E-07	1.4E-06	0.004491	ENSRNO(	protein_coding
Cyfip2	2.58851	6.01477	0.00199	0.00652	1	ENSRNO(	protein_coding
Pdzd2	2.5965	6.04816	5.3E-30	2.1E-28	1.06E-25	ENSRNO(	protein_coding

Tgfb2	2.60083	6.06637	4E-113	2E-110	8.7E-109	ENSRNO(	protein_coding
AABR070	2.60526	6.08501	1.8E-05	8.3E-05	0.354929	ENSRNO(	protein_coding
AABR070	2.60733	6.09377	0.0041	0.01257	1	ENSRNO(	lincRNA
Rn50_7_1	2.61035	6.10653	0.00028	0.00109	1	ENSRNO(	lincRNA
Ripply1	2.6213	6.15304	0.00105	0.00364	1	ENSRNO(	protein_coding
Nr2f2	2.62305	6.16052	1.5E-89	3.8E-87	3.11E-85	ENSRNO(	protein_coding
Exoc7	2.63117	6.19528	5.7E-05	0.00025	1	ENSRNO(	protein_coding
Itga2b	2.63223	6.19983	0.006	0.01773	1	ENSRNO(	protein_coding
Ddit4	2.6388	6.22814	5.6E-30	2.2E-28	1.12E-25	ENSRNO(	protein_coding
RGD1562	2.64054	6.23564	0.00034	0.00131	1	ENSRNO(	protein_coding
Dhh	2.64079	6.23672	0.01055	0.0291	1	ENSRNO(	protein_coding
AC09757!	2.64092	6.23728	0.00351	0.01094	1	ENSRNO(	lincRNA
AABR070	2.64695	6.26341	6.4E-22	1.5E-20	1.28E-17	ENSRNO(	protein_coding
Ch25h	2.64919	6.27316	7.5E-06	3.7E-05	0.151219	ENSRNO(	protein_coding
AABR070	2.65133	6.28246	3.8E-06	2E-05	0.077073	ENSRNO(	lincRNA
Adamts9	2.66037	6.32193	8.3E-68	1.1E-65	1.67E-63	ENSRNO(	protein_coding
Eepd1	2.66321	6.33443	1.2E-76	2.1E-74	2.44E-72	ENSRNO(	protein_coding
Fam13a	2.66462	6.34058	3.7E-61	4.4E-59	7.47E-57	ENSRNO(	protein_coding
Islr	2.6683	6.35682	8.1E-06	4E-05	0.163961	ENSRNO(	protein_coding
Tmbim1	2.67098	6.36862	4E-120	2E-117	8.1E-116	ENSRNO(	protein_coding
Trpm6	2.68133	6.41449	8.8E-10	7.2E-09	1.77E-05	ENSRNO(	protein_coding
Sult1c2_1	2.68574	6.43409	7.5E-05	0.00032	1	ENSRNO(	protein_coding
Slpi	2.68575	6.43416	1.1E-15	1.6E-14	2.24E-11	ENSRNO(	protein_coding
LOC1083	2.68689	6.43925	0.01037	0.02863	1	ENSRNO(	lincRNA
Gabrg1	2.69155	6.46005	0.00247	0.00796	1	ENSRNO(	protein_coding
Klf9	2.69187	6.46149	4.4E-69	6.2E-67	8.87E-65	ENSRNO(	protein_coding
Pcdh20	2.69515	6.47622	7E-116	3E-113	1.3E-111	ENSRNO(	protein_coding
Atp8b3	2.69567	6.47856	1.9E-17	3.2E-16	3.86E-13	ENSRNO(	protein_coding
P2rx5	2.70212	6.50758	2.7E-25	8.1E-24	5.41E-21	ENSRNO(	protein_coding
Clca2	2.70271	6.51024	2.8E-08	1.9E-07	0.000563	ENSRNO(	protein_coding
Akap13	2.70496	6.52042	2E-106	5E-104	3.1E-102	ENSRNO(	protein_coding
Slc2a4	2.70925	6.53982	2.2E-06	1.2E-05	0.043839	ENSRNO(	protein_coding
Vtcn1	2.71118	6.54855	7.5E-05	0.00032	1	ENSRNO(	protein_coding
Clec2d_3	2.72162	6.59614	5.2E-33	2.5E-31	1.05E-28	ENSRNO(	protein_coding
Krt32	2.72187	6.59729	0.00143	0.00485	1	ENSRNO(	protein_coding
LOC4982	2.72485	6.61092	0.00118	0.00404	1	ENSRNO(	protein_coding
Mxd4	2.72539	6.61337	2E-171	3E-168	4.2E-167	ENSRNO(	protein_coding
Fhl1	2.72629	6.61752	1E-149	1E-146	2.9E-145	ENSRNO(	protein_coding
Hck	2.72958	6.63265	1E-07	6.6E-07	0.002079	ENSRNO(	protein_coding
Itgbl1	2.73072	6.63789	1E-99	4.1E-97	2.83E-95	ENSRNO(	protein_coding
Clip3	2.73168	6.64229	3.6E-05	0.00016	0.717997	ENSRNO(	protein_coding
LOC2908	2.73379	6.65202	0.01215	0.03299	1	ENSRNO(	protein_coding
Gli2	2.73855	6.67401	4.5E-15	6.3E-14	9.08E-11	ENSRNO(	protein_coding
Klrd1	2.74154	6.68782	0.00363	0.01128	1	ENSRNO(	protein_coding
LOC6910	2.74525	6.70507	2.5E-06	1.3E-05	0.051012	ENSRNO(	protein_coding
Cst3	2.75851	6.76699	4.4E-58	4.9E-56	8.76E-54	ENSRNO(	protein_coding
AABR070	2.76518	6.79832	0.00271	0.00865	1	ENSRNO(	protein_coding
Pak3	2.76905	6.81657	3.5E-14	4.6E-13	7.12E-10	ENSRNO(	protein_coding
Usp2	2.76923	6.81742	2.3E-25	7E-24	4.67E-21	ENSRNO(	protein_coding

LOC1036	2.77032	6.82258	3.4E-19	6.5E-18	6.83E-15	ENSRNO(	protein_coding
Mroh9	2.77853	6.86152	3.1E-07	1.9E-06	0.006238	ENSRNO(	protein_coding
Hsd17b13	2.78834	6.90836	2.7E-06	1.4E-05	0.054612	ENSRNO(	protein_coding
Dmp1	2.79725	6.95114	0.00011	0.00045	1	ENSRNO(	protein_coding
Col14a1	2.81178	7.02149	0.00265	0.00846	1	ENSRNO(	protein_coding
Fam46a	2.82641	7.09306	7E-148	5E-145	1.4E-143	ENSRNO(	protein_coding
ENSRNO	2.84316	7.17588	0.01184	0.03222	1	ENSRNO(	protein_coding
Ttc22	2.85041	7.21207	2.3E-07	1.4E-06	0.004575	ENSRNO(	protein_coding
Mlph	2.85314	7.22569	3.5E-12	3.8E-11	7.07E-08	ENSRNO(	protein_coding
Tnn	2.85842	7.2522	3.8E-55	3.9E-53	7.64E-51	ENSRNO(	protein_coding
Fam131a	2.86877	7.30441	7.2E-36	3.8E-34	1.45E-31	ENSRNO(	protein_coding
Mcf2	2.87122	7.31686	2.2E-18	4E-17	4.44E-14	ENSRNO(	protein_coding
Pdzrn3	2.8768	7.3452	5.2E-23	1.3E-21	1.05E-18	ENSRNO(	protein_coding
Tph2	2.88071	7.36512	2.4E-10	2.1E-09	4.74E-06	ENSRNO(	protein_coding
Cox8b	2.88346	7.37917	0.0018	0.00597	1	ENSRNO(	protein_coding
Prickle2	2.88484	7.38625	1.1E-19	2.2E-18	2.19E-15	ENSRNO(	protein_coding
Hoxa7	2.89255	7.42582	1.8E-05	8.7E-05	0.371753	ENSRNO(	protein_coding
Adamts1	2.89419	7.43429	2.9E-88	6.8E-86	5.88E-84	ENSRNO(	protein_coding
Mmp27	2.89795	7.45365	0.00508	0.01524	1	ENSRNO(	protein_coding
LOC6900	2.89914	7.45982	0.00598	0.01768	1	ENSRNO(	protein_coding
Fam26e	2.90053	7.46703	2E-28	7.1E-27	3.94E-24	ENSRNO(	protein_coding
LOC1009	2.90082	7.46853	0.00671	0.01954	1	ENSRNO(	protein_coding
Fbxo32	2.90174	7.47325	4.5E-25	1.4E-23	9.16E-21	ENSRNO(	protein_coding
Zcchc5	2.90998	7.51607	7.6E-11	7.1E-10	1.52E-06	ENSRNO(	protein_coding
Sctr	2.91199	7.52653	3.9E-05	0.00017	0.783594	ENSRNO(	protein_coding
Mcam	2.91508	7.54267	1.5E-51	1.4E-49	3E-47	ENSRNO(	protein_coding
AC125248	2.92776	7.6093	5.6E-89	1.3E-86	1.12E-84	ENSRNO(	lincRNA
Lmod1	2.93045	7.62346	1.9E-07	1.2E-06	0.003858	ENSRNO(	protein_coding
Adgrv1	2.93372	7.64078	8.9E-10	7.4E-09	1.8E-05	ENSRNO(	protein_coding
Trnp1	2.93709	7.65866	4.6E-06	2.4E-05	0.093141	ENSRNO(	protein_coding
Otud7a	2.93806	7.66378	0.00534	0.01596	1	ENSRNO(	protein_coding
LOC1003	2.94569	7.70445	0.00565	0.0168	1	ENSRNO(	protein_coding
Adhfe1	2.94852	7.71957	3.1E-33	1.5E-31	6.28E-29	ENSRNO(	protein_coding
Adamts12	2.96106	7.78694	7.8E-47	6.1E-45	1.56E-42	ENSRNO(	protein_coding
Tns1	2.96446	7.80535	1E-118	4E-116	2.2E-114	ENSRNO(	protein_coding
Lcn2	2.96576	7.81239	1.6E-13	2E-12	3.31E-09	ENSRNO(	protein_coding
Nwd1	2.96592	7.81323	0.00711	0.02056	1	ENSRNO(	protein_coding
Hyal1	2.96735	7.82099	5.7E-11	5.4E-10	1.15E-06	ENSRNO(	protein_coding
Trib2	3.00019	8.00106	4E-146	3E-143	8.3E-142	ENSRNO(	protein_coding
Lurap1l	3.00409	8.02273	6.8E-52	6.3E-50	1.36E-47	ENSRNO(	protein_coding
Gpx3	3.02496	8.1396	0.00106	0.00369	1	ENSRNO(	protein_coding
Akap2	3.02698	8.15104	1E-129	5E-127	2.2E-125	ENSRNO(	protein_coding
LOC1009	3.0288	8.1613	1.3E-13	1.6E-12	2.61E-09	ENSRNO(	protein_coding
Edn1	3.0297	8.16637	3.7E-09	2.8E-08	7.46E-05	ENSRNO(	protein_coding
Lsmem1	3.04239	8.23854	2.5E-10	2.2E-09	5.1E-06	ENSRNO(	protein_coding
Slc11a1	3.04268	8.24019	5.6E-06	2.8E-05	0.112682	ENSRNO(	protein_coding
LOC1003	3.05183	8.29264	9.7E-18	1.7E-16	1.95E-13	ENSRNO(	protein_coding
Akr1cl	3.05355	8.30251	4.2E-25	1.2E-23	8.39E-21	ENSRNO(	protein_coding
Klhl24	3.0545	8.30797	4.4E-82	8.6E-80	8.85E-78	ENSRNO(	protein_coding

Ccr3	3.06253	8.35435	0.00701	0.0203	1	ENSRNO0	protein_coding
Casq2	3.06541	8.37109	9.4E-20	1.9E-18	1.89E-15	ENSRNO0	protein_coding
LOC6813	3.06618	8.37555	0.00074	0.00264	1	ENSRNO0	protein_coding
Galnt15	3.07009	8.39825	0.00117	0.00403	1	ENSRNO0	protein_coding
Cecr2	3.08179	8.46667	2.2E-08	1.5E-07	0.000442	ENSRNO0	protein_coding
LOC5011	3.08638	8.4936	3.1E-11	3E-10	6.27E-07	ENSRNO0	protein_coding
Wfdc1	3.09063	8.51866	5.8E-13	6.8E-12	1.17E-08	ENSRNO0	protein_coding
Col4a5	3.10711	8.61655	7E-127	3E-124	1.5E-122	ENSRNO0	protein_coding
Gbp5	3.10987	8.63303	8.7E-12	9E-11	1.75E-07	ENSRNO0	protein_coding
Lpar1	3.10996	8.63358	9E-136	5E-133	1.9E-131	ENSRNO0	protein_coding
Dnajb13	3.11274	8.65024	1.8E-05	8.3E-05	0.354388	ENSRNO0	protein_coding
Dpyd	3.11391	8.65728	2.3E-30	9.3E-29	4.57E-26	ENSRNO0	protein_coding
Pdgfrl	3.1308	8.7592	1.2E-32	5.3E-31	2.33E-28	ENSRNO0	protein_coding
Trim63	3.14002	8.81534	1.7E-13	2.1E-12	3.43E-09	ENSRNO0	protein_coding
Cd200	3.14742	8.86069	1.2E-13	1.5E-12	2.46E-09	ENSRNO0	protein_coding
Ebf2	3.15433	8.90324	0.00065	0.00237	1	ENSRNO0	protein_coding
Mpzl2	3.15636	8.91576	5.5E-92	1.4E-89	1.11E-87	ENSRNO0	protein_coding
Zbtb16	3.15655	8.91694	4E-142	3E-139	8.6E-138	ENSRNO0	protein_coding
Adm	3.16024	8.93981	4.4E-28	1.6E-26	8.91E-24	ENSRNO0	protein_coding
Best2	3.16178	8.94934	5.5E-07	3.2E-06	0.011019	ENSRNO0	protein_coding
Calml3	3.17753	9.04759	0.01755	0.04571	1	ENSRNO0	protein_coding
Vipr2	3.18402	9.08835	0.0004	0.00149	1	ENSRNO0	protein_coding
Tmem207	3.19598	9.16404	2.3E-11	2.3E-10	4.7E-07	ENSRNO0	protein_coding
Gpr141	3.19621	9.16547	1.4E-15	2E-14	2.8E-11	ENSRNO0	protein_coding
Rnf152	3.19741	9.17307	8.3E-39	5E-37	1.67E-34	ENSRNO0	protein_coding
Rftn2	3.21677	9.29705	0.00061	0.00221	1	ENSRNO0	protein_coding
Rn50_20_	3.21929	9.3133	2.8E-10	2.5E-09	5.67E-06	ENSRNO0	lincRNA
Sprr1a	3.2261	9.35736	4.9E-85	1E-82	9.94E-81	ENSRNO0	protein_coding
Abhd15	3.22668	9.36108	4.6E-38	2.7E-36	9.18E-34	ENSRNO0	protein_coding
AC121639	3.23031	9.38469	0.00441	0.01342	1	ENSRNO0	protein_coding
Epb42	3.2325	9.39896	1E-05	5E-05	0.20879	ENSRNO0	protein_coding
AC142138	3.23278	9.40076	0.01657	0.04345	1	ENSRNO0	pseudogene
Erich4	3.236	9.42177	3.4E-05	0.00015	0.683604	ENSRNO0	protein_coding
AABR070	3.24484	9.47966	0.00181	0.00597	1	ENSRNO0	lincRNA
Car13	3.25334	9.53574	1.4E-08	9.8E-08	0.000275	ENSRNO0	protein_coding
Cyp4b1	3.26599	9.61968	2.2E-09	1.7E-08	4.37E-05	ENSRNO0	protein_coding
Oas3	3.27231	9.66192	3.1E-16	4.8E-15	6.22E-12	ENSRNO0	protein_coding
RGD1564	3.29316	9.80256	0.00267	0.00854	1	ENSRNO0	protein_coding
Capsl	3.29472	9.81318	0.01664	0.04363	1	ENSRNO0	protein_coding
Scara5	3.30205	9.86314	3E-117	1E-114	5.3E-113	ENSRNO0	protein_coding
Fam151a	3.30214	9.86378	0.00038	0.00145	1	ENSRNO0	protein_coding
Piezo2	3.31677	9.96433	5E-09	3.8E-08	0.000101	ENSRNO0	protein_coding
Adarb1	3.3331	10.0777	6E-147	4E-144	1.1E-142	ENSRNO0	protein_coding
Pglyrp1	3.33567	10.0957	6E-10	5E-09	1.21E-05	ENSRNO0	protein_coding
Comp	3.34277	10.1455	4.3E-05	0.00019	0.875945	ENSRNO0	protein_coding
Cdc42ep2	3.34752	10.179	2.6E-45	1.9E-43	5.3E-41	ENSRNO0	protein_coding
Plcl1	3.3503	10.1986	5.8E-30	2.3E-28	1.16E-25	ENSRNO0	protein_coding
Pappa	3.3517	10.2085	6.4E-07	3.7E-06	0.012972	ENSRNO0	protein_coding
AABR070	3.35859	10.2574	1.7E-17	2.9E-16	3.4E-13	ENSRNO0	protein_coding

Dnmt3l	3.37478	10.3731	0.00198	0.00649	1	ENSRNO(	protein_coding
Rasl10b	3.3857	10.452	0.01069	0.02943	1	ENSRNO(	protein_coding
Ttpa	3.38806	10.4691	1.8E-24	5E-23	3.55E-20	ENSRNO(	protein_coding
LOC2925	3.39526	10.5214	2.4E-32	1.1E-30	4.86E-28	ENSRNO(	protein_coding
Cpne7	3.39554	10.5234	0.01715	0.04483	1	ENSRNO(	protein_coding
Plagl1	3.39569	10.5246	1.9E-15	2.8E-14	3.92E-11	ENSRNO(	protein_coding
Il18	3.39671	10.5321	1.2E-06	6.7E-06	0.024402	ENSRNO(	protein_coding
Npy1r	3.3971	10.5349	1.4E-05	6.8E-05	0.285252	ENSRNO(	protein_coding
Fetub	3.40097	10.5631	2.4E-08	1.6E-07	0.000476	ENSRNO(	protein_coding
Olr807	3.4017	10.5685	0.0016	0.00534	1	ENSRNO(	protein_coding
Glul	3.40183	10.5695	7E-130	4E-127	1.5E-125	ENSRNO(	protein_coding
AABR070	3.40668	10.605	6.1E-07	3.5E-06	0.012343	ENSRNO(	lincRNA
Hspb7	3.42379	10.7315	4.8E-29	1.8E-27	9.77E-25	ENSRNO(	protein_coding
Corin	3.43542	10.8184	0.00024	0.00095	1	ENSRNO(	protein_coding
Map7d3	3.4393	10.8475	0.01091	0.02996	1	ENSRNO(	protein_coding
Btla	3.44983	10.927	0.00097	0.00339	1	ENSRNO(	protein_coding
AABR070	3.45249	10.9472	0.00103	0.00358	1	ENSRNO(	lincRNA
Hrh4	3.45919	10.9981	0.01114	0.03051	1	ENSRNO(	protein_coding
AABR070	3.46152	11.0159	4.1E-07	2.4E-06	0.008316	ENSRNO(	lincRNA
Acot11	3.46376	11.033	1.6E-05	7.4E-05	0.314107	ENSRNO(	protein_coding
Oas1b	3.46838	11.0685	1.6E-23	4.2E-22	3.2E-19	ENSRNO(	protein_coding
Slc38a4	3.46856	11.0698	5.2E-50	4.6E-48	1.05E-45	ENSRNO(	protein_coding
LOC1003	3.47215	11.0974	0.01024	0.02831	1	ENSRNO(	protein_coding
Col3a1	3.47321	11.1055	5.5E-71	8.3E-69	1.11E-66	ENSRNO(	protein_coding
Pak1	3.47719	11.1363	1E-168	1E-165	2.3E-164	ENSRNO(	protein_coding
Macrod1	3.47954	11.1544	0.00868	0.02447	1	ENSRNO(	protein_coding
Aspa	3.48031	11.1603	2.3E-42	1.6E-40	4.71E-38	ENSRNO(	protein_coding
Grpr	3.48221	11.175	1.1E-08	8.2E-08	0.000228	ENSRNO(	protein_coding
Nt5c1b	3.49172	11.2489	4.8E-12	5.1E-11	9.73E-08	ENSRNO(	protein_coding
Map3k7cl	3.49453	11.2709	5.9E-21	1.3E-19	1.19E-16	ENSRNO(	protein_coding
Oas1a	3.50099	11.3215	4.1E-19	7.7E-18	8.18E-15	ENSRNO(	protein_coding
Acoxl	3.51306	11.4166	0.00994	0.02758	1	ENSRNO(	protein_coding
Pm20d1	3.52425	11.5055	0.00745	0.02142	1	ENSRNO(	protein_coding
Cdh18	3.53038	11.5545	5.7E-86	1.2E-83	1.15E-81	ENSRNO(	protein_coding
Mrvi1	3.5334	11.5787	0.00082	0.0029	1	ENSRNO(	protein_coding
Ctgf	3.53398	11.5833	1E-155	1E-152	2.9E-151	ENSRNO(	protein_coding
Ptchd3	3.53861	11.6206	0.00906	0.02541	1	ENSRNO(	protein_coding
Steap4	3.55337	11.7401	1.2E-11	1.3E-10	2.47E-07	ENSRNO(	protein_coding
Fkbp5	3.56401	11.8269	1E-193	2E-190	2E-189	ENSRNO(	protein_coding
Rnase10	3.56825	11.8618	0.00016	0.00064	1	ENSRNO(	protein_coding
Mrgpre	3.57456	11.9138	0.00054	0.00199	1	ENSRNO(	protein_coding
Tmem169	3.59073	12.0481	9.4E-08	6E-07	0.0019	ENSRNO(	protein_coding
Tsc22d3	3.61896	12.2861	1E-139	7E-137	2.5E-135	ENSRNO(	protein_coding
Lect2	3.62325	12.3227	2.3E-20	4.8E-19	4.56E-16	ENSRNO(	protein_coding
Tp53inp1	3.6473	12.5299	2.3E-75	3.7E-73	4.62E-71	ENSRNO(	protein_coding
Smim1	3.65388	12.5872	4.2E-18	7.5E-17	8.51E-14	ENSRNO(	protein_coding
Trpc6	3.65941	12.6355	1.2E-09	9.5E-09	2.34E-05	ENSRNO(	protein_coding
Akr1c14	3.65943	12.6357	9E-99	2.5E-96	1.8E-94	ENSRNO(	protein_coding
AABR070	3.66936	12.7229	0.00042	0.00158	1	ENSRNO(	pseudogene

Masp1	3.68404	12.8531	6.6E-98	1.8E-95	1.34E-93	ENSRNO(	protein_coding
Hsd11b2	3.68812	12.8895	1.9E-07	1.2E-06	0.003905	ENSRNO(	protein_coding
Nox1	3.70017	12.9976	3.7E-31	1.6E-29	7.41E-27	ENSRNO(	protein_coding
Trpm3	3.70615	13.0515	3.8E-13	4.5E-12	7.59E-09	ENSRNO(	protein_coding
Xdh	3.72213	13.1969	4E-218	1E-214	8.8E-214	ENSRNO(	protein_coding
Tcp11l2	3.72402	13.2142	4E-119	2E-116	8.8E-115	ENSRNO(	protein_coding
Il36b	3.72641	13.2361	0.00514	0.0154	1	ENSRNO(	protein_coding
Fmo1	3.73212	13.2886	1.3E-09	1.1E-08	2.64E-05	ENSRNO(	protein_coding
Mmp1b	3.73677	13.3316	0.00034	0.00128	1	ENSRNO(	protein_coding
Grb10	3.74805	13.4362	0.00036	0.00138	1	ENSRNO(	protein_coding
Glrx	3.75255	13.4782	5E-192	1E-188	1E-187	ENSRNO(	protein_coding
Myoz2	3.7553	13.5039	1.3E-05	6.4E-05	0.269228	ENSRNO(	protein_coding
Cp	3.75969	13.545	2.3E-45	1.7E-43	4.63E-41	ENSRNO(	protein_coding
Syn2	3.78018	13.7387	0.00026	0.00102	1	ENSRNO(	protein_coding
Nyx	3.81397	14.0643	0.00567	0.01685	1	ENSRNO(	protein_coding
Sorbs1	3.81894	14.1129	2E-175	3E-172	4.7E-171	ENSRNO(	protein_coding
Fam46b	3.83111	14.2325	3E-180	4E-177	5.9E-176	ENSRNO(	protein_coding
Hrct1	3.83149	14.2362	1.5E-75	2.5E-73	3.04E-71	ENSRNO(	protein_coding
LOC1036	3.84393	14.3594	2.9E-05	0.00013	0.578182	ENSRNO(	protein_coding
Fzd9	3.84725	14.3925	1.6E-08	1.1E-07	0.000325	ENSRNO(	protein_coding
Ankrd33b	3.8505	14.425	0.00389	0.012	1	ENSRNO(	protein_coding
Fam180a	3.85346	14.4546	4.1E-34	2E-32	8.26E-30	ENSRNO(	protein_coding
Mybphl	3.86486	14.5694	1.3E-06	7.3E-06	0.026884	ENSRNO(	protein_coding
LOC6814	3.92441	15.1832	2.2E-16	3.4E-15	4.37E-12	ENSRNO(	protein_coding
Pdk2	3.92895	15.2311	4E-101	1E-98	7.06E-97	ENSRNO(	protein_coding
Tagap	3.92959	15.2378	4.7E-14	6E-13	9.38E-10	ENSRNO(	protein_coding
Cpb1	3.9421	15.3706	0.00271	0.00864	1	ENSRNO(	protein_coding
LOC1025	3.94556	15.4075	0.00288	0.00912	1	ENSRNO(	lincRNA
Kprp	3.97092	15.6807	2.5E-19	4.8E-18	5.02E-15	ENSRNO(	protein_coding
Sult1b1	3.98807	15.8683	2E-113	9E-111	4.7E-109	ENSRNO(	protein_coding
Otol1	3.99739	15.9711	7.4E-05	0.00032	1	ENSRNO(	protein_coding
Oas1e	4.01641	16.183	1.4E-05	6.7E-05	0.281809	ENSRNO(	protein_coding
Reps2	4.02072	16.2315	8.6E-05	0.00036	1	ENSRNO(	protein_coding
Tmem14(	4.02327	16.2602	5E-165	5E-162	9.2E-161	ENSRNO(	protein_coding
Adgrg2	4.03758	16.4222	0.00226	0.00734	1	ENSRNO(	protein_coding
AABR070	4.04023	16.4525	0.00011	0.00046	1	ENSRNO(	pseudogene
Jaml	4.04879	16.5504	3.9E-22	9.4E-21	7.85E-18	ENSRNO(	protein_coding
Kcnj15	4.06779	16.7698	9.2E-15	1.2E-13	1.85E-10	ENSRNO(	protein_coding
Tmem204	4.06781	16.77	7.8E-15	1.1E-13	1.56E-10	ENSRNO(	protein_coding
Pdgfd	4.06803	16.7725	0.00208	0.00678	1	ENSRNO(	protein_coding
Gabra1	4.07818	16.891	2.5E-06	1.3E-05	0.050159	ENSRNO(	protein_coding
Gda	4.0818	16.9334	2.1E-06	1.1E-05	0.042775	ENSRNO(	protein_coding
Lims2	4.11196	17.2911	7E-127	3E-124	1.5E-122	ENSRNO(	protein_coding
Fras1	4.12929	17.5001	2.3E-20	4.8E-19	4.54E-16	ENSRNO(	protein_coding
Ugt2a1	4.13377	17.5546	2E-33	9.7E-32	4.1E-29	ENSRNO(	protein_coding
Abca1	4.13729	17.5974	0	0	0	ENSRNO(	protein_coding
AABR070	4.15581	17.8248	0.00156	0.00525	1	ENSRNO(	lincRNA
Il23r	4.15889	17.8628	0.00218	0.0071	1	ENSRNO(	protein_coding
Penk	4.15958	17.8714	1.5E-08	1.1E-07	0.000307	ENSRNO(	protein_coding

Nat8f1	4.16084	17.887	0.00218	0.0071	1	ENSRNO0	protein_coding
Ppp1r1b	4.17073	18.01	5.2E-47	4.1E-45	1.05E-42	ENSRNO0	protein_coding
Il1rn	4.17221	18.0285	2.7E-26	8.6E-25	5.34E-22	ENSRNO0	protein_coding
LOC1025	4.19937	18.3712	0.00224	0.00729	1	ENSRNO0	protein_coding
Kyat3	4.21122	18.5227	4E-129	2E-126	7.9E-125	ENSRNO0	protein_coding
Eaf2	4.21188	18.5311	7.1E-21	1.6E-19	1.42E-16	ENSRNO0	protein_coding
Cpz	4.24203	18.9224	2.5E-07	1.5E-06	0.00507	ENSRNO0	protein_coding
Chchd2	4.24981	19.0248	0.00064	0.00232	1	ENSRNO0	protein_coding
Pik3ip1	4.2511	19.0419	8E-142	5E-139	1.6E-137	ENSRNO0	protein_coding
Plet1	4.25771	19.1293	4.1E-71	6.2E-69	8.16E-67	ENSRNO0	protein_coding
Yipf7	4.27901	19.4138	1.9E-05	9E-05	0.385328	ENSRNO0	protein_coding
Robo2	4.3015	19.7188	0.00155	0.0052	1	ENSRNO0	protein_coding
Rapgef5	4.33186	20.1382	4.6E-19	8.7E-18	9.34E-15	ENSRNO0	protein_coding
Atp6v0d2	4.3375	20.2171	7.5E-21	1.6E-19	1.51E-16	ENSRNO0	protein_coding
Vegfd	4.33804	20.2246	0	0	0	ENSRNO0	protein_coding
Lsmem2	4.37753	20.7858	7.8E-07	4.4E-06	0.015685	ENSRNO0	protein_coding
AABR070	4.39019	20.9691	0.00313	0.00986	1	ENSRNO0	pseudogene
Kcnmb4	4.41854	21.3852	4.1E-12	4.4E-11	8.27E-08	ENSRNO0	protein_coding
S100a7a	4.42383	21.4637	4.6E-27	1.6E-25	9.22E-23	ENSRNO0	protein_coding
Tmprss15	4.43911	21.6923	0.00071	0.00256	1	ENSRNO0	protein_coding
Mtnr1a	4.43956	21.699	0.00089	0.00313	1	ENSRNO0	protein_coding
Acer2	4.44204	21.7363	3E-107	1E-104	6.2E-103	ENSRNO0	protein_coding
Romo1	4.47236	22.198	1.2E-07	7.7E-07	0.002441	ENSRNO0	protein_coding
AABR070	4.47671	22.2651	0.0008	0.00283	1	ENSRNO0	lincRNA
Scgb2a1	4.49993	22.6263	6.2E-56	6.6E-54	1.24E-51	ENSRNO0	protein_coding
Lama2	4.50174	22.6548	1.4E-17	2.4E-16	2.87E-13	ENSRNO0	protein_coding
Aqp1	4.51331	22.8371	2E-129	1E-126	4.1E-125	ENSRNO0	protein_coding
Aspn	4.51891	22.926	1.3E-18	2.3E-17	2.58E-14	ENSRNO0	protein_coding
Cldn23	4.52864	23.0811	7.3E-15	9.9E-14	1.46E-10	ENSRNO0	protein_coding
Tmtc1	4.55699	23.5391	1.2E-61	1.5E-59	2.45E-57	ENSRNO0	protein_coding
Zyg11a	4.60343	24.3092	1.4E-26	4.6E-25	2.81E-22	ENSRNO0	protein_coding
Tnnt2	4.62513	24.6776	9.7E-20	1.9E-18	1.96E-15	ENSRNO0	protein_coding
Serpinb10	4.63144	24.7857	3E-09	2.3E-08	5.94E-05	ENSRNO0	protein_coding
Dusp13_3	4.63414	24.8322	0.00055	0.002	1	ENSRNO0	protein_coding
Ackr2	4.69514	25.9047	0.00031	0.00118	1	ENSRNO0	protein_coding
Rxfp3	4.69535	25.9084	7.6E-42	5E-40	1.52E-37	ENSRNO0	protein_coding
NEWGEN	4.72065	26.3667	4E-232	2E-228	8.4E-228	ENSRNO0	protein_coding
LOC6883	4.75949	27.0863	3.2E-06	1.7E-05	0.063935	ENSRNO0	protein_coding
Il22ra2	4.76201	27.1336	0.00042	0.00157	1	ENSRNO0	protein_coding
Dhrs3	4.77031	27.2903	2.7E-69	3.9E-67	5.49E-65	ENSRNO0	protein_coding
Fmo6	4.78705	27.6086	0.0003	0.00117	1	ENSRNO0	protein_coding
Klf15	4.82044	28.2552	2E-130	1E-127	4.5E-126	ENSRNO0	protein_coding
Klhl38	4.8338	28.5179	2.9E-39	1.7E-37	5.81E-35	ENSRNO0	protein_coding
Ogn	4.8384	28.609	3E-164	3E-161	5.9E-160	ENSRNO0	protein_coding
Ms4a6bl	4.85298	28.8997	3.6E-26	1.2E-24	7.24E-22	ENSRNO0	protein_coding
Enpp6	4.87215	29.2862	0.00018	0.00071	1	ENSRNO0	protein_coding
AABR070	4.89695	29.794	1.2E-06	6.8E-06	0.024888	ENSRNO0	lincRNA
Rapgef3	4.90192	29.8968	6.3E-28	2.2E-26	1.27E-23	ENSRNO0	protein_coding
AABR070	4.90433	29.9468	3.2E-10	2.8E-09	6.51E-06	ENSRNO0	lincRNA

Dlg2	4.92927	30.4689	0.00012	0.00051	1	ENSRNO(	protein_coding
Pdha2	4.97389	31.4262	1.1E-06	6.4E-06	0.02303	ENSRNO(	protein_coding
Mfrp	4.97572	31.466	6.9E-13	8E-12	1.39E-08	ENSRNO(	protein_coding
Tgm7l1_1	4.99168	31.8159	1.8E-32	8.1E-31	3.6E-28	ENSRNO(	protein_coding
Ccdc152	5.01394	32.3107	3.1E-43	2.1E-41	6.21E-39	ENSRNO(	protein_coding
Gbp1	5.02819	32.6315	1.5E-06	8.3E-06	0.030772	ENSRNO(	protein_coding
Cldn2	5.05372	33.214	2.7E-25	8.2E-24	5.5E-21	ENSRNO(	protein_coding
Echdc2	5.10012	34.2996	1.8E-10	1.6E-09	3.64E-06	ENSRNO(	protein_coding
Btc	5.1122	34.588	2E-185	3E-182	3.1E-181	ENSRNO(	protein_coding
Bbox1	5.14358	35.3486	2.2E-09	1.7E-08	4.35E-05	ENSRNO(	protein_coding
Ghr	5.21923	37.2515	7E-17	1.1E-15	1.4E-12	ENSRNO(	protein_coding
Aspnl1	5.22602	37.4274	4.8E-43	3.3E-41	9.59E-39	ENSRNO(	protein_coding
LOC3161	5.31053	39.6851	7.5E-16	1.1E-14	1.52E-11	ENSRNO(	protein_coding
LOC1083	5.35392	40.8969	0.00145	0.0049	1	ENSRNO(	protein_coding
Igf2bp1	5.40737	42.4404	4.5E-30	1.8E-28	9.04E-26	ENSRNO(	protein_coding
Upk3b	5.47188	44.3814	4.4E-08	2.9E-07	0.000878	ENSRNO(	protein_coding
Col4a6	5.48516	44.7917	7.2E-15	9.9E-14	1.45E-10	ENSRNO(	protein_coding
Tcaf2	5.49906	45.2254	5E-137	3E-134	9.2E-133	ENSRNO(	protein_coding
AABR070	5.52617	46.0833	1.8E-05	8.5E-05	0.362685	ENSRNO(	lincRNA
ENSRNO	5.53828	46.4717	2.9E-42	1.9E-40	5.77E-38	ENSRNO(	protein_coding
Selenop	5.53844	46.4768	1E-105	3E-103	2.2E-101	ENSRNO(	protein_coding
Abca8	5.54829	46.7952	2.5E-08	1.7E-07	0.000509	ENSRNO(	protein_coding
Fam115c	5.55707	47.0809	2E-160	2E-157	4.9E-156	ENSRNO(	protein_coding
Fmo3	5.60677	48.7312	8.7E-59	9.9E-57	1.75E-54	ENSRNO(	protein_coding
Fabp4	5.66211	50.6367	2.9E-65	3.7E-63	5.75E-61	ENSRNO(	protein_coding
Rasd1	5.6906	51.6464	0	0	0	ENSRNO(	protein_coding
Sdpr	5.69104	51.6622	7.1E-85	1.4E-82	1.43E-80	ENSRNO(	protein_coding
Slc7a13	5.75249	53.9103	0.01825	0.04734	1	ENSRNO(	protein_coding
Nov	5.762	54.2671	6E-253	3E-249	1.2E-248	ENSRNO(	protein_coding
LOC6914	5.78402	55.1015	6.1E-09	4.6E-08	0.000124	ENSRNO(	protein_coding
Tmem179	5.79114	55.3742	6.4E-12	6.7E-11	1.29E-07	ENSRNO(	protein_coding
Timp4	5.80297	55.8301	8.7E-08	5.6E-07	0.001746	ENSRNO(	protein_coding
Cd2	5.84247	57.3798	0.01587	0.04182	1	ENSRNO(	protein_coding
LOC1036	5.8516	57.7441	8E-09	5.9E-08	0.000162	ENSRNO(	protein_coding
Trim54	5.90702	60.0055	0.01475	0.03921	1	ENSRNO(	protein_coding
Mfn2	5.95804	62.1653	0.0194	0.04988	1	ENSRNO(	protein_coding
Cd36	5.98567	63.3676	6E-26	1.9E-24	1.2E-21	ENSRNO(	protein_coding
Cldn1	5.98983	63.5503	9E-56	9.6E-54	1.82E-51	ENSRNO(	protein_coding
Dpep1	6.01714	64.7647	2.1E-12	2.3E-11	4.16E-08	ENSRNO(	protein_coding
AABR070	6.06378	66.8928	0.01272	0.0344	1	ENSRNO(	lincRNA
Rn50_20_	6.06766	67.0732	0.01909	0.0492	1	ENSRNO(	processed_transcript
Snip1	6.11662	69.3884	1.6E-06	8.5E-06	0.03154	ENSRNO(	protein_coding
Cfap52	6.16153	71.5823	1.5E-06	8E-06	0.029601	ENSRNO(	protein_coding
Tfcp2l1	6.18855	72.9357	2E-46	1.5E-44	3.94E-42	ENSRNO(	protein_coding
Sned1	6.19853	73.4419	4.4E-25	1.3E-23	8.78E-21	ENSRNO(	protein_coding
Slc10a6	6.20083	73.5592	2E-06	1.1E-05	0.039808	ENSRNO(	protein_coding
Impg1	6.21094	74.0763	0.01133	0.03099	1	ENSRNO(	protein_coding
MGC9419	6.21812	74.4457	0.01707	0.04464	1	ENSRNO(	protein_coding
Crispld2	6.2355	75.348	1.6E-78	2.9E-76	3.16E-74	ENSRNO(	protein_coding

Nat8f5	6.31497	79.6152	0.01008	0.02791	1	ENSRNO0	protein_coding
Oas2	6.32617	80.2357	0.01008	0.02791	1	ENSRNO0	protein_coding
Fam92b	6.36066	82.1767	1E-10	9.6E-10	2.11E-06	ENSRNO0	protein_coding
Endou	6.40254	84.5971	0.00868	0.02447	1	ENSRNO0	protein_coding
C6	6.43687	86.6345	0.00816	0.02319	1	ENSRNO0	protein_coding
Fam107a	6.48532	89.5935	3.5E-86	7.6E-84	6.97E-82	ENSRNO0	protein_coding
Kcnc4	6.49382	90.1226	7.5E-11	7E-10	1.5E-06	ENSRNO0	protein_coding
AABR070	6.53821	92.9388	0.00736	0.02119	1	ENSRNO0	lincRNA
Fmo2	6.58974	96.3185	4.5E-43	3.1E-41	9.06E-39	ENSRNO0	protein_coding
Gpr17	6.62384	98.6219	0.00643	0.01885	1	ENSRNO0	protein_coding
LOC1009	6.63166	99.1584	0.00685	0.01989	1	ENSRNO0	protein_coding
RGD1559	6.662	101.266	0.00641	0.01878	1	ENSRNO0	protein_coding
Cfap100	6.66647	101.58	5E-132	2E-129	9.3E-128	ENSRNO0	protein_coding
Rgs6	6.6905	103.286	0.00565	0.01679	1	ENSRNO0	protein_coding
Trpc4	6.70304	104.188	1.5E-11	1.5E-10	3.04E-07	ENSRNO0	protein_coding
Rpl13a_2	6.73592	106.59	0.00823	0.02335	1	ENSRNO0	protein_coding
Slc30a2	6.75677	108.141	0.00531	0.01585	1	ENSRNO0	protein_coding
Plin4	6.79579	111.105	2.4E-79	4.5E-77	4.83E-75	ENSRNO0	protein_coding
Spink5	6.81651	112.713	0.00642	0.01882	1	ENSRNO0	protein_coding
Nr1h5	6.8285	113.653	0.00473	0.01427	1	ENSRNO0	protein_coding
Oas1i	6.85473	115.739	0.01022	0.02826	1	ENSRNO0	protein_coding
LOC1025	6.89143	118.721	0.00858	0.02423	1	ENSRNO0	protein_coding
LOC6900	6.91915	121.024	5.7E-12	6E-11	1.15E-07	ENSRNO0	protein_coding
Gucy1a3	7.04154	131.739	1.1E-25	3.3E-24	2.13E-21	ENSRNO0	protein_coding
Lamp3	7.09789	136.986	4.9E-13	5.7E-12	9.78E-09	ENSRNO0	protein_coding
Olr806	7.11184	138.317	0.00363	0.01127	1	ENSRNO0	protein_coding
Scrg1	7.16568	143.577	2.6E-40	1.6E-38	5.28E-36	ENSRNO0	protein_coding
AC111231	7.20797	147.848	0.00274	0.00873	1	ENSRNO0	lincRNA
Abra	7.25733	152.994	0.00262	0.00838	1	ENSRNO0	protein_coding
Pld6	7.27866	155.272	0.00261	0.00835	1	ENSRNO0	protein_coding
Omd	7.45409	175.35	1E-225	4E-222	2.8E-221	ENSRNO0	protein_coding
Tmeff2	7.56949	189.951	6.2E-20	1.3E-18	1.25E-15	ENSRNO0	protein_coding
Mlxipl	7.80769	224.052	9.8E-25	2.8E-23	1.98E-20	ENSRNO0	processed_transcript
Spef2	7.81486	225.168	8.4E-15	1.1E-13	1.68E-10	ENSRNO0	protein_coding
Rn50_4_1	7.88242	235.963	9.3E-54	9.2E-52	1.87E-49	ENSRNO0	unprocessed_pseudogene
Lrrc32	7.93577	244.852	7.6E-77	1.4E-74	1.54E-72	ENSRNO0	protein_coding
LOC1083	7.97466	251.544	0.00123	0.00422	1	ENSRNO0	protein_coding
Srcin1	8.01958	259.498	2E-10	1.8E-09	3.97E-06	ENSRNO0	protein_coding
Sult1a1	8.09726	273.853	1E-162	1E-159	2.3E-158	ENSRNO0	protein_coding
Cetn3	8.17835	289.686	0.00135	0.00458	1	ENSRNO0	protein_coding
Fibin	8.24717	303.841	6.6E-81	1.3E-78	1.34E-76	ENSRNO0	protein_coding
Fam213a	8.49188	360.006	7.5E-51	6.9E-49	1.51E-46	ENSRNO0	protein_coding
Arhgef37	8.50867	364.222	1.2E-11	1.2E-10	2.37E-07	ENSRNO0	protein_coding
LOC1036	8.57267	380.743	0.00063	0.00228	1	ENSRNO0	protein_coding
Cyp2c22	8.71768	421.002	0.00029	0.00111	1	ENSRNO0	protein_coding
Orm1	8.72336	422.663	7E-148	5E-145	1.3E-143	ENSRNO0	protein_coding
AABR070	8.79901	445.417	4.5E-12	4.8E-11	9.1E-08	ENSRNO0	lincRNA
LOC1036	8.82463	453.396	0.00115	0.00395	1	ENSRNO0	protein_coding
Ces1d	9.42959	689.59	6.9E-13	8E-12	1.4E-08	ENSRNO0	protein_coding

Rn50_4_[illegible]	9.62026	787.021	6.7E-05	0.00029	1	ENSRNO[illegible]	unprocessed_pseudogene
AC14213[illegible]	10.0382	1051.49	2.8E-05	0.00013	0.565363	ENSRNO[illegible]	lincRNA
LOC1009[illegible]	10.0616	1068.68	4.7E-05	0.00021	0.949621	ENSRNO[illegible]	protein_coding
Scn3b	11.6285	3166.12	1.3E-06	7.1E-06	0.026093	ENSRNO[illegible]	protein_coding
Scgb2a2	11.735	3408.62	3.8E-21	8.5E-20	7.57E-17	ENSRNO[illegible]	protein_coding
Hif3a	12.202	4711.7	3.8E-07	2.2E-06	0.007574	ENSRNO[illegible]	protein_coding

Table 5. Genes in the enriched pathways in MM cells treated dexamethasone analyzed using GOBP database

Differentially expressed genes: Fold change>2.5, P-value<0.05, FDR<0.05

**GO:0030198~extracellular matrix organization**			
**Gene**	**Fold change**	**P-value**	**FDR**
COL9A2	74.33264086	0.010040746	0.03879516
LOC100910978	35.90074097	1.30344E-06	2.49276E-05
CRISPLD2	34.45986282	4.61442E-13	2.75777E-11
ADAMTS13	16.71005471	3.17737E-06	5.3653E-05
NOX1	14.00205381	4.86059E-09	1.5552E-07
DMP1	13.9539088	1.95242E-13	1.22826E-11
OLFML2A	12.04845434	8.81456E-12	4.35111E-10
ECM2	9.772444347	2.17147E-09	7.35576E-08
BCL3	8.334361204	0.000518746	0.003714578
ADAMTS10	6.860687838	1.20555E-10	4.87286E-09
COL11A1	6.205130065	4.1219E-19	4.62998E-17
SPOCK2	5.921050948	0.001006919	0.006229127
MIA	5.428095653	0.005031762	0.022568079
COL25A1	4.806080608	2.00816E-06	3.62901E-05
FBLN5	4.541930675	8.53244E-09	2.56802E-07
COL4A6	4.481232093	2.02408E-06	3.64988E-05
ADAMTS1	4.203043095	1.35719E-15	1.08148E-13
MMP28	4.185437196	9.40123E-10	3.38323E-08
ITGA8	3.859477337	1.33335E-05	0.000183339
TNFRSF11B	3.845052422	2.15659E-08	6.04496E-07
COL3A1	3.800384345	4.24835E-12	2.24974E-10
PDGFRA	3.794389859	9.48726E-11	3.9297E-09
COL11A2	3.733746153	0.006332673	0.027158347
IBSP	3.661296895	0.005624184	0.0247151
ADAMTSL4	3.623455453	1.10503E-12	6.24716E-11
ADAMTS9	3.536385635	1.65455E-13	1.05275E-11
SLC39A8	3.526268739	1.86163E-17	1.76003E-15
RECK	3.299282464	1.98056E-08	5.58898E-07
EMILIN1	3.178032173	5.37977E-06	8.45306E-05
MMP19	2.968901161	4.77367E-15	3.61459E-13
NID1	2.876154303	1.32191E-06	2.52243E-05
ADAMTS4	2.824551454	0.004087187	0.019174375
COL8A2	2.67078481	5.8558E-09	1.82479E-07
OLFML2B	2.581252617	3.78222E-07	8.25185E-06
SMOC1	2.551403819	8.97478E-07	1.77523E-05
NPHP3	2.53613594	1.27277E-06	2.44249E-05
COL5A3	-3.310142054	1.12195E-08	3.31709E-07
FBLN1	-3.97903708	2.72529E-06	4.67743E-05
FBLN2	-5.212455158	2.78524E-05	0.000344481
POSTN	-10.75813069	5.63119E-19	6.24055E-17

MMP9	-26.12383289	2.35527E-12	1.27139E-10
ADAMTS8	-116.1246731	7.97619E-52	5.8032E-49

GO:0007160~cell-matrix adhesion

Gene	Fold change	P-value	FDR
ITGB4	21.97904544	1.33411E-71	3.18928E-68
CD34	21.28555811	0.003403236	0.016637381
TNN	11.2868835	7.59812E-36	2.23065E-33
VCAM1	11.06079892	2.1563E-08	6.04496E-07
ITGA10	8.750829206	2.08967E-12	1.14276E-10
BCL2L11	8.30604115	1.41389E-11	6.70255E-10
ITGA2B	6.838402241	0.000230483	0.001966805
ITGA1	5.585127571	5.43166E-13	3.17809E-11
MIA	5.428095653	0.005031762	0.022568079
ITGA8	3.859477337	1.33335E-05	0.000183339
COL3A1	3.800384345	4.24835E-12	2.24974E-10
FREM1	3.424260328	3.70785E-11	1.61581E-09
ITGA11	3.293056794	5.5835E-09	1.76624E-07
SNED1	3.18861164	0.002672702	0.013812242
HOXD3	3.184985192	1.02974E-05	0.000147911
EMILIN1	3.178032173	5.37977E-06	8.45306E-05
ITGAE	3.13447253	0.000207929	0.001809149
TMEM8B	2.905454342	0.000336357	0.00265876
NID1	2.876154303	1.32191E-06	2.52243E-05
COL5A3	-3.310142054	1.12195E-08	3.31709E-07
ITGA2	-3.654925134	0.00312372	0.015586036
ITGAX	-4.723163952	1.67432E-07	3.96294E-06
NID2	-6.563760667	3.37457E-19	3.89449E-17
ITGA6	-8.249493906	1.14766E-09	4.03465E-08

GO:0032496~response to lipopolysaccharide

Gene	Fold change	P-value	FDR
TIMP4	333.5321154	2.04447E-09	6.98208E-08
ACE	18.33667856	5.30093E-12	2.75484E-10
FMO1	17.99574195	5.74905E-35	1.5032E-32
IL1RN	15.56895813	1.55198E-52	1.29855E-49
XDH	15.19540187	1.12216E-37	3.6112E-35
ORM1	13.27578618	3.71443E-22	5.05344E-20
VCAM1	11.06079892	2.1563E-08	6.04496E-07
PTGER4	10.53550646	1.17699E-05	0.000164818
LBP	9.81824809	1.26432E-12	7.00568E-11
P2RX7	8.8392647	4.71336E-05	0.00054097
SLC11A1	8.115286748	0.000198425	0.001741187
ADM	6.739969308	2.63291E-13	1.63182E-11
JAK2	5.543637988	5.86425E-18	5.87619E-16
CEBPB	4.461398102	2.28409E-18	2.31648E-16

CASP1	4.435047836	3.31646E-05	0.000399264
HP	4.249456655	7.80517E-05	0.000821457
IL6R	4.178561724	6.1505E-17	5.62418E-15
NFKBIA	4.105047256	1.55194E-14	1.12425E-12
EDN1	4.097984932	7.08842E-08	1.79996E-06
CD14	3.740775575	1.76923E-16	1.51827E-14
CLDN1	3.625255925	0.011960804	0.044409163
PROS1	3.290988653	2.22663E-10	8.60518E-09
IRAK3	2.966806359	3.87579E-06	6.41517E-05
NR4A1	2.965746368	9.33046E-13	5.32887E-11
SPARC	2.840424273	4.60737E-09	1.48269E-07
FOS	2.79228439	0.000224218	0.001920321
ALPL	2.624848043	0.000293149	0.002387129
IL18BP	2.567089983	0.001695686	0.009536291
PTGFR	2.536498044	2.38496E-07	5.43731E-06
DNMT1	-2.612355154	2.22212E-05	0.000284395
HMGB2	-2.656975887	0.000101191	0.001013363
DUSP10	-3.023265716	7.18389E-05	0.000763756
PTAFR	-3.142351206	0.000425935	0.003205616
RGD1559962	-3.292609166	0.000474833	0.003475875
CXCL10	-3.637949626	5.27043E-06	8.31247E-05
PTGS2	-4.394808806	9.66212E-23	1.38193E-20
CCL2	-6.730641109	3.9246E-09	1.28521E-07
RGD1561694	-7.390782382	0.009573854	0.037440726
CXCL1	-7.880005028	4.17608E-23	6.02435E-21
GGT1	-8.376314475	7.99338E-28	1.45393E-25
IL1A	-10.5317494	0.001220676	0.007266735
NGFR	-11.01158209	0.001327466	0.007779615
CXCL3	-14.67204551	5.13074E-12	2.68305E-10
NGF	-20.61539137	7.68043E-07	1.54291E-05
MMP9	-26.12383289	2.35527E-12	1.27139E-10
SELP	-28.76787923	0.007979283	0.03249578

GO:0007229~integrin-mediated signaling pathway

Gene	Fold change	P-value	FDR
ITGB4	21.97904544	1.33411E-71	3.18928E-68
TXK	12.66232124	4.63238E-17	4.25924E-15
ITGA10	8.750829206	2.08967E-12	1.14276E-10
ITGA2B	6.838402241	0.000230483	0.001966805
CDH17	6.652908101	3.18217E-14	2.1824E-12
PTN	6.59080324	2.86774E-10	1.09814E-08
ITGB8	6.41151039	2.5274E-11	1.13084E-09
NRP1	6.368201026	2.28851E-11	1.03224E-09
PLPP3	6.349189828	3.25454E-18	3.28081E-16
ITGA1	5.585127571	5.43166E-13	3.17809E-11
PRAM1	4.774552152	0.007111298	0.029772444

ADAMTS1	4.203043095	1.35719E-15	1.08148E-13
ITGA8	3.859477337	1.33335E-05	0.000183339
COL3A1	3.800384345	4.24835E-12	2.24974E-10
IBSP	3.661296895	0.005624184	0.0247151
ADAMTS9	3.536385635	1.65455E-13	1.05275E-11
ITGA11	3.293056794	5.5835E-09	1.76624E-07
ITGAE	3.13447253	0.000207929	0.001809149
TEC	2.979918112	1.01159E-09	3.61708E-08
ITGA2	-3.654925134	0.00312372	0.015586036
PTK2B	-4.691592856	1.92145E-07	4.46577E-06
ITGAX	-4.723163952	1.67432E-07	3.96294E-06
ITGA6	-8.249493906	1.14766E-09	4.03465E-08

GO:0051384~response to glucocorticoid

Gene	Fold change	P-value	FDR
BCKDHA	2.976765	8.67E-08	2.16E-06
PTPRU	6.988494	0.000414	0.003138
IL1RN	15.56896	1.55E-52	1.3E-49
TRIM63	12.82543	5.13E-16	4.25E-14
SPARC	2.840424	4.61E-09	1.48E-07
AGL	3.137221	8.88E-07	1.76E-05
WNT7B	-240.965	8.06E-06	0.00012
HP	4.249457	7.81E-05	0.000821
ADM	6.739969	2.63E-13	1.63E-11
CXCL1	-7.88001	4.18E-23	6.02E-21
NGF	-20.6154	7.68E-07	1.54E-05
PTGS2	-4.39481	9.66E-23	1.38E-20
AREG	-6.13237	9.65E-13	5.49E-11
SULT1A1	78.63697	2.81E-56	2.77E-53
GHR	16.44679	9.43E-66	1.31E-62
FABP4	19.12444	2.29E-13	1.43E-11
MGP	4.386064	0.000101	0.001013
SDC1	-4.18219	1.84E-11	8.44E-10
CCL2	-6.73064	3.92E-09	1.29E-07
ALPL	2.624848	0.000293	0.002387
ACSBG1	89.08671	0.008461	0.034012
HMGCS2	13.31064	0.00784	0.032062
A2M	88.30558	3.69E-38	1.26E-35
IL6R	4.178562	6.15E-17	5.62E-15

GO:0019221~cytokine-mediated signaling pathway

Gene	Fold change	P-value	FDR
GHR	16.44678977	9.42804E-66	1.31474E-62
CRLF1	5.562276634	0.000226311	0.001937132
JAK2	5.543637988	5.86425E-18	5.87619E-16
IL17RE	5.391782409	6.05023E-32	1.31486E-29

LIFR	5.354635193	1.75286E-11	8.10287E-10
IL17RB	5.260308446	0.011546967	0.043179205
CX3CL1	5.135326239	7.75013E-05	0.000816692
NUMBL	4.665091369	5.28272E-13	3.10179E-11
IL20RB	4.631453523	1.69551E-07	4.00743E-06
IL6R	4.178561724	6.1505E-17	5.62418E-15
LEPR	4.069028339	4.54248E-06	7.36568E-05
IL17RD	3.6516445	0.011718504	0.043654821
IL11RA1	3.154711901	2.87136E-06	4.90251E-05
IRAK3	2.966806359	3.87579E-06	6.41517E-05
STAT2	2.739044235	6.40794E-13	3.72328E-11
CISH	2.655452137	3.4358E-05	0.000411853
JAK3	2.566203511	0.000168367	0.00151966
CSF2RA	2.536409626	0.009521207	0.037293354
IL1R1	-2.500178651	1.33092E-08	3.88007E-07
EREG	-4.064137379	2.92877E-06	4.98575E-05
CCL2	-6.730641109	3.9246E-09	1.28521E-07
GREM2	-8.997841201	0.004100356	0.019214604
IL1A	-10.5317494	0.001220676	0.007266735
CSF1R	-10.68964158	1.59739E-08	4.59292E-07
IL1RL1	-12.88243722	9.69986E-10	3.47575E-08
PTPRN	-13.42716006	2.41561E-23	3.51503E-21
IL1RL2	-22.66495205	3.79661E-14	2.59316E-12

GO:0006954~inflammatory response

**Gene**	**Fold change**	**P-value**	**FDR**
CXCL13	367.2566322	1.30017E-22	1.81309E-20
DPEP1	174.5015187	1.20176E-57	1.43645E-54
ALOX5	87.75006723	0.006493376	0.027684117
KNG1	40.3588965	5.83663E-27	9.96634E-25
HRH4	21.37051029	6.13673E-05	0.000669876
IL1RN	15.56895813	1.55198E-52	1.29855E-49
PTGER4	10.53550646	1.17699E-05	0.000164818
AGTR1B	9.287827725	0.000540082	0.003834419
P2RX7	8.8392647	4.71336E-05	0.00054097
SLC11A1	8.115286748	0.000198425	0.001741187
NTRK2	7.911554894	0.000162525	0.001478278
IL18	7.70197651	0.000593478	0.004133245
HYAL1	6.475870401	5.42805E-09	1.72032E-07
CYP26B1	6.353388214	4.49432E-06	7.30174E-05
LOXL3	5.571903274	4.02793E-21	5.18487E-19
IL17RE	5.391782409	6.05023E-32	1.31486E-29
CX3CL1	5.135326239	7.75013E-05	0.000816692
CCRL2	5.103212505	0.000100771	0.001012184
CASP4	4.916879294	7.27542E-12	3.6628E-10
CYP4F6	4.013958597	1.90147E-06	3.46992E-05

HCK	3.770317637	0.000490422	0.003558853
CD14	3.740775575	1.76923E-16	1.51827E-14
TNFRSF4	3.695955158	0.000363915	0.00283508
IL15	2.797719696	0.008409822	0.033862756
HYAL3	2.68062852	0.000445919	0.003309092
PRKCZ	2.631277695	6.03778E-05	0.000661665
PTGFR	2.536498044	2.38496E-07	5.43731E-06
SPHK1	2.50795428	9.83415E-09	2.923E-07
IL1R1	-2.500178651	1.33092E-08	3.88007E-07
HCN1	-2.599954543	0.002426334	0.012780067
PTAFR	-3.142351206	0.000425935	0.003205616
CCL7	-3.415708823	0.00026115	0.002176337
CXCL10	-3.637949626	5.27043E-06	8.31247E-05
SDC1	-4.182189945	1.83597E-11	8.44042E-10
PTGS2	-4.394808806	9.66212E-23	1.38193E-20
CIITA	-5.902145113	5.20135E-09	1.65789E-07
NLRP3	-6.385487139	7.52657E-07	1.5193E-05
CXCR3	-6.568523839	3.20134E-06	5.39489E-05
CCL2	-6.730641109	3.9246E-09	1.28521E-07
CXCL1	-7.880005028	4.17608E-23	6.02435E-21
IL1A	-10.5317494	0.001220676	0.007266735
CSF1R	-10.68964158	1.59739E-08	4.59292E-07
CCL22	-10.99487225	3.74679E-05	0.000444357
IL17F	-11.05216582	0.001606534	0.009113131
CXCL3	-14.67204551	5.13074E-12	2.68305E-10
NGF	-20.61539137	7.68043E-07	1.54291E-05
SELP	-28.76787923	0.007979283	0.03249578
CCR1	-34.26757538	1.67739E-13	1.06323E-11

**GO:0007155~cell adhesion**			
**Gene**	**Fold change**	**P-value**	**FDR**
OMD	55.57568901	4.27937E-88	2.38703E-84
ITGB4	21.97904544	1.33411E-71	3.18928E-68
PCDHB9	15.67723801	0.006272675	0.02695607
CLDN6	11.42085015	0.013691558	0.049431399
VCAM1	11.06079892	2.1563E-08	6.04496E-07
PCDHGB7	8.891139572	0.001020035	0.006289337
NLGN3	8.612521856	3.04759E-06	5.167E-05
GP1BA	8.124688574	0.000493411	0.003575891
VWF	7.467865984	8.20758E-05	0.000857874
PCDHB19	7.371544521	5.42889E-07	1.12994E-05
SVEP1	7.107537354	1.73731E-14	1.2424E-12
CCDC141	6.960768244	0.000375833	0.002911843
PCDHB7	6.930124611	0.001253541	0.007420148
PLPP3	6.349189828	3.25454E-18	3.28081E-16
PCDHB4	6.155774107	9.79284E-05	0.000989967

CNTNAP5C	5.680066182	0.004401456	0.020307132
ITGA1	5.585127571	5.43166E-13	3.17809E-11
THBS4	5.306981441	0.001629956	0.009230349
CX3CL1	5.135326239	7.75013E-05	0.000816692
CD36	4.810874909	0.00127109	0.007494863
CDHR2	4.706890662	0.00261676	0.013570849
COL12A1	4.63516912	7.53467E-19	8.13452E-17
FBLN5	4.541930675	8.53244E-09	2.56802E-07
PCDHB20	3.950621797	0.000941219	0.005883401
CTNNAL1	3.832767106	1.01727E-11	4.97747E-10
PCDHB5	3.686405787	0.000829857	0.005339033
IBSP	3.661296895	0.005624184	0.0247151
PCDHGB6	3.645811835	0.003616315	0.01744463
CLDN1	3.625255925	0.011960804	0.044409163
CLDN15	3.285910398	3.88816E-05	0.0004582
PCDHGB8	3.183406141	1.94783E-05	0.000253727
ITGAE	3.13447253	0.000207929	0.001809149
PCDHB12	3.123826389	9.71598E-05	0.000984781
SDC4	3.077490194	2.00444E-11	9.06549E-10
PCDHGA7	2.958824521	0.005535221	0.024413912
PCDH12	2.935896114	0.00893496	0.035424753
PCDH20	2.612539355	7.6333E-05	0.000806412
PARVB	-2.589639552	5.4908E-05	0.000612963
CADM4	-2.663870637	0.002503753	0.013101253
TNFRSF12A	-2.687729185	2.52653E-06	4.4054E-05
PCDH7	-2.848945521	0.008102406	0.032893175
MFGE8	-3.153407673	4.18895E-11	1.80841E-09
CYP1B1	-3.439671435	1.54783E-13	9.88605E-12
SIGLEC10	-3.608891649	1.94757E-06	3.53095E-05
LMLN	-3.750545805	3.25347E-07	7.16363E-06
NTM	-3.836011991	2.74758E-06	4.71087E-05
SDC1	-4.182189945	1.83597E-11	8.44042E-10
CDH2	-4.231110982	0.001199653	0.007161964
PTK2B	-4.691592856	1.92145E-07	4.46577E-06
TNC	-9.854142456	4.1736E-19	4.65607E-17
POSTN	-10.75813069	5.63119E-19	6.24055E-17
LAMA3	-16.53014643	1.78245E-43	8.52214E-41
SELP	-28.76787923	0.007979283	0.03249578
ACAN	-73.60127095	1.12637E-53	1.04715E-50

GO:0006260~DNA replication

Gene	Fold change	P-value	FDR
LPIN1	9.067708563	1.70719E-35	4.68329E-33
BTC	2.66125095	0.001141876	0.00689576
POLD1	-2.508124421	0.000796551	0.005176608
ORC1	-2.527584332	0.003929129	0.018584201

DNAJC2	-2.547565522	2.45063E-06	4.29412E-05
DSCC1	-2.589854358	5.19978E-06	8.23987E-05
CHAF1A	-2.647241168	6.45163E-05	0.000696976
RFC3	-2.688206603	0.00015618	0.001433632
RECQL4	-2.710148453	0.000433024	0.003237815
BLM	-2.722864006	3.28461E-05	0.000395999
RRM2	-2.753013895	0.005137557	0.022919722
LIG1	-2.808192083	2.22295E-05	0.000284395
POLE	-2.810957015	9.23731E-05	0.000946768
TICRR	-2.824202127	6.46695E-05	0.00069818
MCM7	-2.826190083	6.38032E-05	0.000691505
PIF1	-2.843365276	0.007268512	0.030241493
POLA2	-3.13299364	1.05682E-06	2.06357E-05
POLA1	-3.264389792	2.44006E-07	5.54782E-06

Table 6. Genes in the enriched pathways in KMM cells treated dexamethasone analyzed using GOBP database

Differentially expressed genes: Fold change>2.5, P-value<0.05, FDR<0.05

GO:0071356~cellular response to tumor necrosis factor			
**Gene**	**Fold change**	**P-value**	**FDR**
CLDN1	63.55025517	9.02413E-56	9.57931E-54
FABP4	50.63673663	2.85568E-65	3.69385E-63
GBP1	32.63150504	1.52806E-06	8.31829E-06
XDH	13.196887	4.3512E-218	1.0624E-214
IL18	10.53205028	1.21175E-06	6.71445E-06
EDN1	8.166373417	3.7056E-09	2.84169E-08
HYAL1	7.820988606	5.68686E-11	5.39727E-10
LCN2	7.812386598	1.64348E-13	2.01384E-12
ADAMTS12	7.786943834	7.75056E-47	6.07968E-45
SLC2A4	6.539815433	2.17691E-06	1.163E-05
ABCC2	5.829312112	0.000703311	0.002524268
GBP3	5.724507607	5.41592E-10	4.58733E-09
CEBPA	4.514381893	1.77823E-46	1.37282E-44
ZFP36	4.360724755	1.14087E-63	1.42843E-61
GBP2	3.798185765	4.13418E-49	3.57295E-47
INPP5K	3.741012445	1.50182E-88	3.53417E-86
PPARGC1A	3.669396788	1.22775E-45	9.22326E-44
NFKBIA	2.849843924	1.32761E-61	1.58115E-59
WDR35	2.560725359	1.01513E-25	3.14225E-24
ASL	2.51050983	9.56175E-26	2.96916E-24
ABCB1A	-2.578008541	1.96385E-34	9.71085E-33
GPER1	-3.275958632	4.54961E-05	0.000201002
TNFRSF21	-3.437459125	1.6206E-30	6.70626E-29
AKAP12	-4.062154915	7.1603E-41	4.52605E-39
CYP1B1	-4.812667213	1.08327E-44	7.80752E-43
CCL20	-4.920922792	0.00047568	0.001762327
FAS	-5.053111527	0.005588637	0.016629687
CX3CL1	-5.392660965	4.28155E-07	2.52096E-06
ACOD1	-5.562370058	0.003162844	0.009947934
MMP1	-5.685226154	0.000706431	0.002534068
GPD1	-6.113889371	1.03713E-17	1.79268E-16
CCL22	-7.202685389	0.001731857	0.005750954
CCL9	-7.832509209	0.006198726	0.018244955
POSTN	-9.225154356	1.49222E-85	3.16805E-83
CCL4	-9.343700352	0.002702413	0.008621944
CHI3L1	-9.914624681	0.009960386	0.027630487
HAS2	-17.31046368	6.71678E-13	7.78589E-12
CCL7	-17.38644385	2.73857E-08	1.88212E-07
CCL2	-18.40749723	1.753E-28	6.39993E-27
CCL17	-20.22632162	0.01551371	0.040992126

MMP9	-40.82298252	1.4443E-34	7.1599E-33

GO:0042060~wound healing

Gene	Fold change	P-value	FDR
AQP1	22.83707641	2.032E-129	9.6804E-127
PAK1	11.1362534	1.1338E-168	1.3841E-165
COL3A1	11.10552649	5.49365E-71	8.31798E-69
SLC11A1	8.240193163	5.59548E-06	2.83431E-05
TGFB2	6.066368676	4.3026E-113	1.5563E-110
LOX	5.728288788	5.0649E-96	1.35517E-93
POU2F3	5.003683918	0.016219328	0.042643143
SDC4	4.425229874	8.09961E-69	1.13814E-66
HPSE	3.993944397	4.11983E-57	4.54624E-55
PDGFRB	2.982869205	4.8726E-30	1.95024E-28
SPARC	2.952283781	2.31168E-36	1.26122E-34
FN1	2.864021286	1.72818E-25	5.27418E-24
PPARA	2.678141165	1.14899E-06	6.4029E-06
FNTB	2.640134719	1.46019E-53	1.42602E-51
EVPL	2.575443753	1.0322E-37	6.01817E-36
NOTCH3	2.548901511	0.001216761	0.004166512
BMP4	2.544828188	3.59282E-21	8.14095E-20
JAG2	-2.559443991	2.78365E-24	7.73404E-23
TP53	-2.642949239	9.82166E-32	4.28207E-30
ENG	-2.647981695	0.002609895	0.008351517
P2RY2	-3.39340619	1.34599E-17	2.30613E-16
EPB41L4B	-3.684536664	1.87054E-07	1.15509E-06
FGF2	-3.959613205	5.7836E-09	4.34649E-08
IL33	-4.086287631	0.00413016	0.012654162
SDC1	-4.106093497	2.07568E-49	1.80992E-47
CXCL2	-4.315348319	0.003353109	0.010509133
WNT5A	-4.896998873	1.94119E-16	3.07755E-15
CCL20	-4.920922792	0.00047568	0.001762327
CX3CL1	-5.392660965	4.28155E-07	2.52096E-06
F13A1	-6.73940661	8.51725E-43	5.77635E-41
WNT7A	-6.949355437	1.52123E-05	7.2417E-05
ITGB6	-7.372834983	0.000337301	0.001287002
ITGA2	-7.456719638	4.87071E-07	2.84409E-06
POSTN	-9.225154356	1.49222E-85	3.16805E-83
TNC	-10.92177789	1.8362E-108	6.1836E-106
IL1A	-24.85776899	2.93621E-15	4.16486E-14

GO:0051384~response to glucocorticoid

Gene	Fold change	P-value	FDR
SULT1A1	273.8534017	1.1373E-162	1.1692E-159
FABP4	50.63673663	2.85568E-65	3.69385E-63
GHR	37.2515478	6.96644E-17	1.13296E-15

IL1RN	18.02852394	2.65404E-26	8.63978E-25
HSD11B2	12.88947143	1.939E-07	1.19396E-06
ADM	8.939814786	4.42695E-28	1.57213E-26
TRIM63	8.815335461	1.70532E-13	2.08437E-12
DPYD	8.657280374	2.26806E-30	9.26773E-29
TPH2	7.365115303	2.35343E-10	2.07246E-09
MGP	5.660717971	4.49287E-16	6.8612E-15
ANXA3	5.303575335	2.16898E-89	5.29556E-87
DUSP1	5.041587953	1.2274E-143	8.2669E-141
IL6R	4.8104698	3.68087E-65	4.72992E-63
ALDH3A1	4.311878303	4.04389E-23	1.03791E-21
RXRA	3.470830687	2.72761E-50	2.43268E-48
BCKDHA	3.162372363	1.27788E-15	1.87665E-14
SPARC	2.952283781	2.31168E-36	1.26122E-34
FN1	2.864021286	1.72818E-25	5.27418E-24
BMP4	2.544828188	3.59282E-21	8.14095E-20
PPARGC1B	-2.718426785	1.07632E-05	5.2256E-05
AREG	-2.994446717	1.42175E-17	2.43167E-16
CXCL1	-3.508465465	2.63682E-30	1.07074E-28
SFTPD	-3.814245199	0.016597185	0.043531116
PTGS2	-4.016172181	5.36428E-55	5.48561E-53
SDC1	-4.106093497	2.07568E-49	1.80992E-47
CXCL2	-4.315348319	0.003353109	0.010509133
WNT5A	-4.896998873	1.94119E-16	3.07755E-15
FAS	-5.053111527	0.005588637	0.016629687
TNF	-5.985567167	0.009320519	0.026066493
NGF	-6.297470936	3.94758E-31	1.67618E-29
CCL2	-18.40749723	1.753E-28	6.39993E-27
WNT7B	-18.4111899	3.65154E-05	0.000163883
ALDOB	-25.16072471	1.35028E-07	8.49396E-07

GO:0030198~extracellular matrix organization

Gene	Fold change	P-value	FDR
CRISPLD2	75.34795575	1.56751E-78	2.91587E-76
IMPG1	74.07631342	0.011331622	0.030994152
COL4A6	44.79169965	7.2206E-15	9.87624E-14
OTOL1	15.97110747	7.44472E-05	0.000318463
MMP1B	13.33155365	0.000335808	0.001281592
NOX1	12.99757673	3.68144E-31	1.56658E-29
COL3A1	11.10552649	5.49365E-71	8.31798E-69
COL4A5	8.616547201	7.4768E-127	3.319E-124
ADAMTS12	7.786943834	7.75056E-47	6.07968E-45
MMP27	7.45364635	0.005081157	0.015238009
ADAMTS1	7.434285854	2.9198E-88	6.78922E-86
DMP1	6.951140703	0.000108117	0.000450359
ADAMTS9	6.321932318	8.2948E-68	1.14094E-65

TGFB2	6.066368676	4.3026E-113	1.5563E-110
TNFRSF11B	5.836451193	4.71271E-47	3.77248E-45
IMPG2	4.86865465	0.000451387	0.001680293
TNR	4.770258257	1.13052E-10	1.03766E-09
ADAMTSL3	4.181613817	7.6598E-107	2.4526E-104
COL4A4	3.698192582	2.38743E-13	2.87493E-12
CSGALNACT1	3.593381708	1.26654E-41	8.35746E-40
ADAMTSL4	3.559398688	3.31262E-76	5.5301E-74
ECM2	3.144241045	0.005440388	0.016218202
TNXB	3.074989161	1.24626E-27	4.35455E-26
VHL	3.055572302	1.18902E-46	9.21584E-45
FN1	2.864021286	1.72818E-25	5.27418E-24
COL6A4	2.861170764	0.005072113	0.015215559
SPOCK2	2.829746223	0.008295004	0.023518364
COL8A1	2.635756683	1.74618E-26	5.74182E-25
MMP10	-2.733325999	2.79701E-19	5.37709E-18
COLQ	-2.988866964	0.003269743	0.010264324
FBLN1	-3.203683131	0.005711728	0.016964943
ITGA8	-3.530900117	1.46987E-41	9.60182E-40
ADAMTS17	-3.975472955	0.002523947	0.008105513
ADAMTSL2	-5.834002656	2.7422E-10	2.39323E-09
TNF	-5.985567167	0.009320519	0.026066493
MMP2	-8.115936975	0.000981426	0.003418191
POSTN	-9.225154356	1.49222E-85	3.16805E-83
ADAMTS8	-12.99020482	2.5785E-121	1.0716E-118
MMP9	-40.82298252	1.4443E-34	7.1599E-33

GO:0071347~cellular response to interleukin-1

Gene	Fold change	P-value	FDR
GBP1	32.63150504	1.52806E-06	8.31829E-06
XDH	13.196887	4.3512E-218	1.0624E-214
EDN1	8.166373417	3.7056E-09	2.84169E-08
HYAL1	7.820988606	5.68686E-11	5.39727E-10
LCN2	7.812386598	1.64348E-13	2.01384E-12
ADAMTS12	7.786943834	7.75056E-47	6.07968E-45
ABCC2	5.829312112	0.000703311	0.002524268
GBP3	5.724507607	5.41592E-10	4.58733E-09
GBP2	3.798185765	4.13418E-49	3.57295E-47
CEBPB	3.561506936	4.83226E-20	9.9456E-19
FN1	2.864021286	1.72818E-25	5.27418E-24
MYLK3	-2.518387902	0.008303773	0.023536393
P2RY2	-3.39340619	1.34599E-17	2.30613E-16
AKAP12	-4.062154915	7.1603E-41	4.52605E-39
CXCL2	-4.315348319	0.003353109	0.010509133
CCL20	-4.920922792	0.00047568	0.001762327
FAS	-5.053111527	0.005588637	0.016629687

CX3CL1	-5.392660965	4.28155E-07	2.52096E-06
ACOD1	-5.562370058	0.003162844	0.009947934
MMP1	-5.685226154	0.000706431	0.002534068
CCL22	-7.202685389	0.001731857	0.005750954
CCL9	-7.832509209	0.006198726	0.018244955
MMP2	-8.115936975	0.000981426	0.003418191
CCL4	-9.343700352	0.002702413	0.008621944
CHI3L1	-9.914624681	0.009960386	0.027630487
HAS2	-17.31046368	6.71678E-13	7.78589E-12
CCL7	-17.38644385	2.73857E-08	1.88212E-07
CCL2	-18.40749723	1.753E-28	6.39993E-27
CCL17	-20.22632162	0.01551371	0.040992126
MMP9	-40.82298252	1.4443E-34	7.1599E-33

GO:0006954~inflammatory response

Gene	Fold change	P-value	FDR
DPEP1	64.76466865	2.06795E-12	2.27556E-11
ACKR2	25.90469331	0.000308356	0.001184661
S100A7A	21.46367886	4.57788E-27	1.55504E-25
IL1RN	18.02852394	2.65404E-26	8.63978E-25
IL36B	13.23609385	0.005141186	0.015399117
HRH4	10.9981346	0.011135375	0.030512926
IL18	10.53205028	1.21175E-06	6.71445E-06
CCR3	8.35434946	0.007014455	0.020300242
SLC11A1	8.240193163	5.59548E-06	2.83431E-05
HYAL1	7.820988606	5.68686E-11	5.39727E-10
HCK	6.632645229	1.03259E-07	6.58672E-07
ABCC2	5.829312112	0.000703311	0.002524268
CXCL13	5.377593331	4.73109E-05	0.000208313
KNG1	4.8324197	3.68138E-07	2.19021E-06
SPHK1	4.827906592	1.16669E-45	8.79841E-44
LOXL3	4.82159245	2.00858E-79	3.84623E-77
IL17RE	4.628092509	1.12789E-45	8.53877E-44
HPSE	3.993944397	4.11983E-57	4.54624E-55
TLR10	3.621550894	0.000667983	0.00240898
RXRA	3.470830687	2.72761E-50	2.43268E-48
IRAK2	3.439084103	3.91398E-13	4.64447E-12
TLR6	3.412123521	6.1797E-29	2.31673E-27
PXK	3.04663927	1.97567E-69	2.83741E-67
SPP1	2.880890372	1.88848E-36	1.03872E-34
CCRL2	2.771846627	5.17602E-05	0.000226576
F2RL1	-2.563783346	1.23162E-20	2.65226E-19
CD44	-2.797376349	5.91219E-26	1.85356E-24
GPER1	-3.275958632	4.54961E-05	0.000201002
SERPINB1A	-3.31348557	4.27116E-20	8.80933E-19
CXCL1	-3.508465465	2.63682E-30	1.07074E-28

CXCL10	-4.013234564	3.14062E-45	2.30611E-43
PTGS2	-4.016172181	5.36428E-55	5.48561E-53
SDC1	-4.106093497	2.07568E-49	1.80992E-47
CXCL2	-4.315348319	0.003353109	0.010509133
CSF1R	-4.594053425	5.50108E-06	2.78866E-05
CCL20	-4.920922792	0.00047568	0.001762327
CX3CL1	-5.392660965	4.28155E-07	2.52096E-06
TNF	-5.985567167	0.009320519	0.026066493
NGF	-6.297470936	3.94758E-31	1.67618E-29
PTAFR	-6.730764272	0.002826419	0.008980903
CCL22	-7.202685389	0.001731857	0.005750954
ITGB6	-7.372834983	0.000337301	0.001287002
CXCL11	-7.791583827	1.07262E-07	6.82165E-07
CCL9	-7.832509209	0.006198726	0.018244955
CXCL3	-9.313019911	1.82871E-10	1.63621E-09
CCL4	-9.343700352	0.002702413	0.008621944
PLA2G2A	-9.796287149	8.68165E-30	3.3982E-28
CHI3L1	-9.914624681	0.009960386	0.027630487
IGFBP4	-13.92685716	1.1695E-139	6.9223E-137
KRT16	-14.60480622	1.65852E-09	1.33091E-08
CCL7	-17.38644385	2.73857E-08	1.88212E-07
CCL2	-18.40749723	1.753E-28	6.39993E-27
CCL17	-20.22632162	0.01551371	0.040992126
NPPB	-21.76453569	0.000279197	0.001080072
IL1A	-24.85776899	2.93621E-15	4.16486E-14

GO:0070098~chemokine-mediated signaling pathway

**Gene**	**Fold change**	**P-value**	**FDR**
ACKR2	25.90469331	0.000308356	0.001184661
CCR3	8.35434946	0.007014455	0.020300242
CXCL13	5.377593331	4.73109E-05	0.000208313
CXCR4	3.508540219	0.006612558	0.019285724
CCRL2	2.771846627	5.17602E-05	0.000226576
CXCL1	-3.508465465	2.63682E-30	1.07074E-28
CXCL10	-4.013234564	3.14062E-45	2.30611E-43
CXCL2	-4.315348319	0.003353109	0.010509133
CCL20	-4.920922792	0.00047568	0.001762327
CX3CL1	-5.392660965	4.28155E-07	2.52096E-06
CCL22	-7.202685389	0.001731857	0.005750954
CXCL11	-7.791583827	1.07262E-07	6.82165E-07
CCL9	-7.832509209	0.006198726	0.018244955
CXCL3	-9.313019911	1.82871E-10	1.63621E-09
CCL4	-9.343700352	0.002702413	0.008621944
CCL7	-17.38644385	2.73857E-08	1.88212E-07
CCL2	-18.40749723	1.753E-28	6.39993E-27
CCL17	-20.22632162	0.01551371	0.040992126

GO:0032496~response to lipopolysaccharide			
**Gene**	**Fold change**	**P-value**	**FDR**
ORM1	422.662634	6.5569E-148	5.3362E-145
CLDN1	63.55025517	9.02413E-56	9.57931E-54
TIMP4	55.83013294	8.66868E-08	5.58064E-07
IL1RN	18.02852394	2.65404E-26	8.63978E-25
PENK	17.87143023	1.52408E-08	1.08248E-07
IL23R	17.86278992	0.002181475	0.007096697
FMO1	13.28859544	1.31088E-09	1.06153E-08
XDH	13.196887	4.3512E-218	1.0624E-214
ADM	8.939814786	4.42695E-28	1.57213E-26
SLC11A1	8.240193163	5.59548E-06	2.83431E-05
EDN1	8.166373417	3.7056E-09	2.84169E-08
SLPI	6.434156847	1.11151E-15	1.64615E-14
ABCC2	5.829312112	0.000703311	0.002524268
TH	5.605941587	4.72241E-14	6.07629E-13
IL6R	4.8104698	3.68087E-65	4.72992E-63
CEBPB	3.561506936	4.83226E-20	9.9456E-19
NOS1	3.242633106	0.00537046	0.016026864
SOCS1	3.187643133	3.24305E-06	1.69639E-05
PTPN22	3.038188336	0.003065265	0.009670612
SPARC	2.952283781	2.31168E-36	1.26122E-34
EDNRB	2.866041718	0.001874512	0.00617836
NFKBIA	2.849843924	1.32761E-61	1.58115E-59
WDR35	2.560725359	1.01513E-25	3.14225E-24
ASL	2.51050983	9.56175E-26	2.96916E-24
MGST1	2.5028495	6.35576E-09	4.74181E-08
ABCB1A	-2.578008541	1.96385E-34	9.71085E-33
DUSP10	-3.037411995	9.13369E-17	1.47559E-15
NCF2	-3.141483474	0.000194179	0.000771814
CXCL1	-3.508465465	2.63682E-30	1.07074E-28
GGT1	-3.82744968	3.46546E-12	3.74377E-11
CXCL10	-4.013234564	3.14062E-45	2.30611E-43
PTGS2	-4.016172181	5.36428E-55	5.48561E-53
AKAP12	-4.062154915	7.1603E-41	4.52605E-39
FAS	-5.053111527	0.005588637	0.016629687
ACOD1	-5.562370058	0.003162844	0.009947934
TNF	-5.985567167	0.009320519	0.026066493
NGF	-6.297470936	3.94758E-31	1.67618E-29
PTAFR	-6.730764272	0.002826419	0.008980903
CXCL3	-9.313019911	1.82871E-10	1.63621E-09
HNF4A	-15.79219858	1.47103E-09	1.1863E-08
NGFR	-16.76141294	0.00233363	0.007550183
CCL2	-18.40749723	1.753E-28	6.39993E-27
NPPB	-21.76453569	0.000279197	0.001080072

IL1A	-24.85776899	2.93621E-15	4.16486E-14
MMP9	-40.82298252	1.4443E-34	7.1599E-33

GO:0035025~positive regulation of Rho protein signal transduction

Gene	Fold change	P-value	FDR
ABRA	152.9936594	0.002619623	0.008376961
GPR17	98.6218968	0.006431682	0.018845427
COL3A1	11.10552649	5.49365E-71	8.31798E-69
LPAR1	8.633583306	9.4615E-136	5.1334E-133
AKAP13	6.520417539	1.5641E-106	4.9273E-104
F11R	3.692423416	1.73343E-69	2.50795E-67
LPAR6	3.128044943	4.73942E-25	1.40258E-23
PDGFRB	2.982869205	4.8726E-30	1.95024E-28
F2RL1	-2.563783346	1.23162E-20	2.65226E-19
RTN4R	-2.683535262	0.000193739	0.000770224
ADGRG1	-12.36568243	0.000317967	0.001218469
F2RL2	-16.46021954	1.84899E-08	1.30095E-07
NGFR	-16.76141294	0.00233363	0.007550183

GO:0007411~axon guidance

Gene	Fold change	P-value	FDR
LAMA2	22.65476546	1.42703E-17	2.43856E-16
ROBO2	19.71882805	0.001548446	0.005201073
GLI2	6.674008784	4.50877E-15	6.29488E-14
TGFB2	6.066368676	4.3026E-113	1.5563E-110
ABLIM1	5.982613783	6.1221E-113	2.1741E-110
MATN2	5.443296138	0.006840011	0.01987786
BOC	4.629100824	3.79327E-05	0.000169854
LAMB2	3.978523668	5.13036E-57	5.62956E-55
GAP43	3.947584261	2.06526E-06	1.10608E-05
ARHGEF25	3.868042394	1.42902E-19	2.80237E-18
EMB	3.69976228	1.51552E-77	2.74085E-75
ALCAM	3.646607588	1.82905E-98	5.10357E-96
B4GAT1	3.613436709	9.1289E-50	8.03178E-48
SEMA3E	3.048452173	2.35E-29	8.98242E-28
RTN4RL1	2.911725404	1.41392E-19	2.77834E-18
SEMA3B	2.811049833	9.58061E-13	1.08922E-11
SEMA6C	2.72565413	4.30745E-05	0.000190995
LGR4	2.663632015	1.05469E-32	4.86574E-31
NOTCH3	2.548901511	0.001216761	0.004166512
RTN4R	-2.683535262	0.000193739	0.000770224
PLXNA3	-2.898356731	4.28729E-18	7.58509E-17
LHX2	-3.379192868	1.802E-15	2.60901E-14
CSF1R	-4.594053425	5.50108E-06	2.78866E-05
SEMA6A	-4.824734701	4.73366E-37	2.63413E-35
WNT5A	-4.896998873	1.94119E-16	3.07755E-15

SEMA4A	-5.383870703	0.000110072	0.000457712
LAMA3	-5.608822088	4.30849E-64	5.50023E-62
SEMA7A	-8.079844176	2.67263E-37	1.52192E-35
SEMA6D	-8.588210297	0.004582016	0.013896883
POU4F2	-9.846404374	0.015252928	0.040390481
NGFR	-16.76141294	0.00233363	0.007550183
LMX1A	-28.28371363	0.008128469	0.023113299

GO:0071346~cellular response to interferon-gamma

Gene	Fold change	P-value	FDR
CLDN1	63.55025517	9.02413E-56	9.57931E-54
GBP1	32.63150504	1.52806E-06	8.31829E-06
IL18	10.53205028	1.21175E-06	6.71445E-06
CDC42EP2	10.1789857	2.63165E-45	1.93968E-43
GBP5	8.633032253	8.68239E-12	9.0445E-11
EDN1	8.166373417	3.7056E-09	2.84169E-08
GBP3	5.724507607	5.41592E-10	4.58733E-09
MRC1	4.948026325	7.47195E-05	0.000319558
GBP2	3.798185765	4.13418E-49	3.57295E-47
RAB11FIP5	2.517031967	9.07426E-50	8.01984E-48
ASL	2.51050983	9.56175E-26	2.96916E-24
WNT5A	-4.896998873	1.94119E-16	3.07755E-15
CCL20	-4.920922792	0.00047568	0.001762327
FAS	-5.053111527	0.005588637	0.016629687
CX3CL1	-5.392660965	4.28155E-07	2.52096E-06
ACOD1	-5.562370058	0.003162844	0.009947934
TNF	-5.985567167	0.009320519	0.026066493
CCL22	-7.202685389	0.001731857	0.005750954
CCL9	-7.832509209	0.006198726	0.018244955
CCL4	-9.343700352	0.002702413	0.008621944
CCL7	-17.38644385	2.73857E-08	1.88212E-07
CCL2	-18.40749723	1.753E-28	6.39993E-27
CCL17	-20.22632162	0.01551371	0.040992126

GO:0007155~cell adhesion

Gene	Fold change	P-value	FDR
OMD	175.3499228	1.3665E-225	4.4484E-222
CLDN1	63.55025517	9.02413E-56	9.57931E-54
CD36	63.36757761	5.97993E-26	1.87179E-24
CLDN2	33.21400852	2.73082E-25	8.20591E-24
CLDN23	23.08105049	7.26662E-15	9.93223E-14
LAMA2	22.65476546	1.42703E-17	2.43856E-16
RNASE10	11.86181496	0.000157134	0.000635563
COMP	10.14549594	4.34971E-05	0.000192737
MCAM	7.5426732	1.48816E-51	1.38413E-49
PCDH20	6.476222925	6.6919E-116	2.5136E-113

PDZD2	6.04815935	5.25494E-30	2.09897E-28
ITGB4	5.475206339	7.23333E-18	1.25919E-16
ATP1B1	5.25057053	1.06963E-88	2.5478E-86
GP1BA	4.790961498	0.000381225	0.001438859
TNR	4.770258257	1.13052E-10	1.03766E-09
SDC4	4.425229874	8.09961E-69	1.13814E-66
COL12A1	3.934873866	5.85802E-55	5.95932E-53
PLPP3	3.794766825	5.24163E-19	9.82528E-18
EMB	3.69976228	1.51552E-77	2.74085E-75
F11R	3.692423416	1.73343E-69	2.50795E-67
ALCAM	3.646607588	1.82905E-98	5.10357E-96
NLGN3	3.390345456	2.29355E-05	0.000106181
ACHE	3.002090741	0.000145143	0.000590488
SPP1	2.880890372	1.88848E-36	1.03872E-34
CDH16	2.8688062	0.00079756	0.002831838
FN1	2.864021286	1.72818E-25	5.27418E-24
ITGA1	2.862009692	2.92504E-21	6.65873E-20
VCAN	2.852854748	4.31146E-12	4.60423E-11
SUSD5	2.78404235	6.82355E-06	3.4095E-05
HAPLN1	2.776860482	3.33117E-18	5.97469E-17
PCDHGA2	2.60738736	4.68598E-18	8.25306E-17
PCDH18	-2.708011766	2.02759E-29	7.78052E-28
CD44	-2.797376349	5.91219E-26	1.85356E-24
NCAN	-3.159348384	0.011112215	0.030466561
ICAM4	-3.330640251	0.00167271	0.005568666
FLG2	-3.441401723	0.008093069	0.023026049
SDC1	-4.106093497	2.07568E-49	1.80992E-47
MYBPC1	-4.133037573	0.007942026	0.022668953
PCDH1	-4.323761431	2.27669E-60	2.66277E-58
SIGLEC10	-4.463786492	1.006E-06	5.64469E-06
LEF1	-4.541124418	6.25319E-32	2.76955E-30
CYP1B1	-4.812667213	1.08327E-44	7.80752E-43
CX3CL1	-5.392660965	4.28155E-07	2.52096E-06
LAMA3	-5.608822088	4.30849E-64	5.50023E-62
ACAN	-7.067938187	4.45216E-19	8.4019E-18
PARVB	-8.488093633	5.81942E-11	5.51236E-10
POSTN	-9.225154356	1.49222E-85	3.16805E-83
TNC	-10.92177789	1.8362E-108	6.1836E-106
ADGRG1	-12.36568243	0.000317967	0.001218469
FIBCD1	-28.46755505	0.007992287	0.02279243
CLDN4	-84.56732295	5.40067E-08	3.57095E-07

GO:0007165~signal transduction

Gene	Fold change	P-value	FDR
OGN	28.60901529	2.937E-164	3.187E-161
ACKR2	25.90469331	0.000308356	0.001184661

IL1RN	18.02852394	2.65404E-26	8.63978E-25
GABRA1	16.890988	2.49077E-06	1.32201E-05
TAGAP	15.23784578	4.65766E-14	6.00089E-13
GRB10	13.43618375	0.000363308	0.001376821
TCP11L2	13.21418756	4.3761E-119	1.7444E-116
NOX1	12.99757673	3.68144E-31	1.56658E-29
RASL10B	10.45196413	0.010686415	0.029427189
GABRG1	6.46005403	0.002474497	0.007958485
TGFB2	6.066368676	4.3026E-113	1.5563E-110
TNFRSF11B	5.836451193	4.71271E-47	3.77248E-45
GAS6	5.804290955	2.54719E-16	3.99933E-15
ANGPT4	5.700460405	1.71303E-19	3.33922E-18
RASAL3	5.17317085	0.013820861	0.037009742
ARR3	5.082343002	0.000472602	0.001752251
PDE1B	5.001133751	3.17935E-08	2.16599E-07
GABRA3	4.924445025	8.22587E-19	1.52003E-17
CALCOCO1	4.666156763	5.09954E-42	3.39946E-40
GUCY2G	4.660486774	0.000173619	0.000696471
CBLB	4.610398969	3.12984E-86	7.02667E-84
CDNF	4.545387141	2.4672E-10	2.16582E-09
TNFRSF25	4.200100654	1.04638E-07	6.66379E-07
STARD8	4.053737058	0.010140913	0.028055569
NPR2	3.958632693	6.27327E-09	4.68564E-08
PLPP3	3.794766825	5.24163E-19	9.82528E-18
BEX2	3.764450837	0.010158366	0.028095893
PLPP1	3.482944355	1.59653E-87	3.62599E-85
PDE7B	3.457214	0.001226665	0.004197479
FGF11	3.433374213	0.010476691	0.028898563
DLC1	3.390306201	6.89826E-41	4.37457E-39
SRGAP3	3.345956051	2.10229E-27	7.26761E-26
RAPGEF4	3.202729067	3.06347E-37	1.73437E-35
ANPEP	3.145352025	3.49116E-17	5.8063E-16
PAG1	3.09211414	1.35858E-07	8.54067E-07
LVRN	3.088469141	3.92379E-05	0.000175256
PPARG	3.069839698	1.10499E-30	4.60184E-29
PDGFRB	2.982869205	4.8726E-30	1.95024E-28
SPP1	2.880890372	1.88848E-36	1.03872E-34
LRRC39	2.821211766	2.72593E-09	2.12971E-08
SECTM1A	2.776356343	0.000809585	0.002870882
SYDE2	2.767180962	1.47434E-31	6.37097E-30
CSF3	2.747984981	0.000251872	0.000982537
TOM1L2	2.602162458	7.8548E-30	3.08692E-28
LOC365791	2.559005793	0.018332635	0.047502391
TENM3	2.529184449	1.53786E-22	3.78307E-21
OPHN1	2.519797176	1.60511E-11	1.6177E-10
CXCL16	-2.508371219	6.26088E-20	1.2725E-18

TRAIP	-2.547249123	4.41493E-26	1.40215E-24
BMP3	-2.622399084	1.95002E-05	9.12718E-05
TNFSF9	-2.67691872	5.80472E-12	6.13184E-11
FCER1G	-2.677601267	0.008549604	0.024138604
RASSF4	-3.064300346	0.010017259	0.027772476
INHBE	-3.121934152	0.018459404	0.047773828
GDNF	-3.132789339	6.98691E-06	3.484E-05
PDE2A	-3.291232376	0.001629555	0.005439836
CXCL1	-3.508465465	2.63682E-30	1.07074E-28
GNB3	-3.540468826	0.003055126	0.009640182
HHIP	-3.828806735	1.70373E-05	8.0438E-05
MOB3B	-3.90107182	0.000180704	0.000722667
AKAP12	-4.062154915	7.1603E-41	4.52605E-39
RASL12	-4.200892569	0.001725249	0.005732827
ERFE	-4.968243469	1.29369E-12	1.45176E-11
FAS	-5.053111527	0.005588637	0.016629687
OLFM1	-5.370660979	1.42679E-52	1.35282E-50
TNF	-5.985567167	0.009320519	0.026066493
ARHGAP22	-6.163564101	2.527E-49	2.19366E-47
SCUBE3	-6.429696144	1.56184E-11	1.57572E-10
CSF2	-7.076501952	0.00795664	0.022704436
CCL4	-9.343700352	0.002702413	0.008621944
PRL5A1	-12.14366513	7.25455E-07	4.14801E-06
PROM2	-13.56136518	7.64342E-30	3.0099E-28
PRL7A3	-16.15121527	8.29835E-08	5.34576E-07
NGFR	-16.76141294	0.00233363	0.007550183
FAM83F	-17.94998333	1.91282E-06	1.02838E-05
IL1A	-24.85776899	2.93621E-15	4.16486E-14
CCL27	-25.64232281	0.009872465	0.027421643
SCUBE1	-28.49045798	4.07096E-13	4.81319E-12

GO:0001666~response to hypoxia

Gene	Fold change	P-value	FDR
HIF3A	4711.700063	3.76104E-07	2.23489E-06
VEGFD	20.22458158	0	0
PENK	17.87143023	1.52408E-08	1.08248E-07
HSD11B2	12.88947143	1.939E-07	1.19396E-06
PAK1	11.1362534	1.1338E-168	1.3841E-165
IL18	10.53205028	1.21175E-06	6.71445E-06
ADM	8.939814786	4.42695E-28	1.57213E-26
EDN1	8.166373417	3.7056E-09	2.84169E-08
CST3	6.766986695	4.35119E-58	4.91257E-56
DDIT4	6.228135268	5.55631E-30	2.21481E-28
TGFB2	6.066368676	4.3026E-113	1.5563E-110
CAV3	5.968006614	0.000553643	0.002026185
TH	5.605941587	4.72241E-14	6.07629E-13

ANGPTL4	5.35487	1.84807E-09	1.47574E-08
ITPR2	5.313983124	6.21511E-31	2.62757E-29
ATP1B1	5.25057053	1.06963E-88	2.5478E-86
CRYAB	4.948338726	5.4056E-126	2.3463E-123
PLOD2	4.884128026	5.86095E-73	9.38328E-71
ANGPT1	4.557105734	8.32302E-25	2.40126E-23
ALDH3A1	4.311878303	4.04389E-23	1.03791E-21
PPARGC1A	3.669396788	1.22775E-45	9.22326E-44
CXCR4	3.508540219	0.006612558	0.019285724
NOS1	3.242633106	0.00537046	0.016026864
VHL	3.055572302	1.18902E-46	9.21584E-45
PPARA	2.678141165	1.14899E-06	6.4029E-06
BNIP3	2.655003611	1.62925E-30	6.72781E-29
JAG2	-2.559443991	2.78365E-24	7.73404E-23
ABCB1A	-2.578008541	1.96385E-34	9.71085E-33
ENG	-2.647981695	0.002609895	0.008351517
TFRC	-2.687521724	2.43312E-18	4.38006E-17
SLC6A4	-3.787907365	0.004149754	0.012708214
FAS	-5.053111527	0.005588637	0.016629687
CX3CL1	-5.392660965	4.28155E-07	2.52096E-06
TNF	-5.985567167	0.009320519	0.026066493
MYB	-6.015750593	9.47921E-13	1.07832E-11
ITGA2	-7.456719638	4.87071E-07	2.84409E-06
MMP2	-8.115936975	0.000981426	0.003418191
POSTN	-9.225154356	1.49222E-85	3.16805E-83
CCL2	-18.40749723	1.753E-28	6.39993E-27
NPPB	-21.76453569	0.000279197	0.001080072
IL1A	-24.85776899	2.93621E-15	4.16486E-14
MMP9	-40.82298252	1.4443E-34	7.1599E-33

GO:0060700~regulation of ribonuclease activity

Gene	Fold change	P-value	FDR
OAS1I	115.7387443	0.010222326	0.02826479
OAS2	80.23566493	0.010077492	0.02790599
OAS1E	16.18303053	1.39939E-05	6.69268E-05
OAS1A	11.32151074	4.06312E-19	7.69E-18
OAS1B	11.06845749	1.58753E-23	4.21872E-22
OAS3	9.661916955	3.08978E-16	4.80873E-15
OASL	-3.012552837	1.0034E-09	8.23466E-09

GO:0001525~angiogenesis

Gene	Fold change	P-value	FDR
HIF3A	4711.700063	3.76104E-07	2.23489E-06
RAPGEF3	29.89681013	6.3268E-28	2.23868E-26
VEGFD	20.22458158	0	0
NOX1	12.99757673	3.68144E-31	1.56658E-29

GLUL	10.56946795	7.3253E-130	3.6687E-127
IL18	10.53205028	1.21175E-06	6.71445E-06
MCAM	7.5426732	1.48816E-51	1.38413E-49
HOXA7	7.425822742	1.84603E-05	8.67372E-05
ITGA2B	6.199832243	0.006001284	0.017730614
ANGPT4	5.700460405	1.71303E-19	3.33922E-18
ANGPTL4	5.35487	1.84807E-09	1.47574E-08
ANGPT1	4.557105734	8.32302E-25	2.40126E-23
ANG2	4.073326584	0.002011636	0.006590285
ANPEP	3.145352025	3.49116E-17	5.8063E-16
VHL	3.055572302	1.18902E-46	9.21584E-45
EPGN	3.035136074	1.36917E-06	7.51832E-06
TSPAN12	2.986977033	0.000146812	0.000596657
PDGFRB	2.982869205	4.8726E-30	1.95024E-28
FN1	2.864021286	1.72818E-25	5.27418E-24
MEIS1	2.702240988	3.06729E-30	1.24038E-28
BMP4	2.544828188	3.59282E-21	8.14095E-20
ENG	-2.647981695	0.002609895	0.008351517
FGF9	-2.803763346	4.90486E-06	2.50397E-05
CCDC134	-2.829012627	7.61245E-18	1.32283E-16
NCL	-2.853977573	7.30304E-33	3.42069E-31
PTGS2	-4.016172181	5.36428E-55	5.48561E-53
CYP1B1	-4.812667213	1.08327E-44	7.80752E-43
WNT7A	-6.949355437	1.52123E-05	7.2417E-05
ANGPTL2	-7.888012654	2.13561E-46	1.6294E-44
MMP2	-8.115936975	0.000981426	0.003418191
ADGRG1	-12.36568243	0.000317967	0.001218469
WNT7B	-18.4111899	3.65154E-05	0.000163883
APLN	-20.2753939	9.66027E-15	1.30577E-13
OVOL2	-36.70225212	0.003488668	0.010883352

GO:0050919~negative chemotaxis

Gene	Fold change	P-value	FDR
SEMA3E	3.048452173	2.35E-29	8.98242E-28
RTN4RL1	2.911725404	1.41392E-19	2.77834E-18
SEMA3B	2.811049833	9.58061E-13	1.08922E-11
SEMA6C	2.72565413	4.30745E-05	0.000190995
LGR4	2.663632015	1.05469E-32	4.86574E-31
RTN4R	-2.683535262	0.000193739	0.000770224
PLXNA3	-2.898356731	4.28729E-18	7.58509E-17
SEMA6A	-4.824734701	4.73366E-37	2.63413E-35
WNT5A	-4.896998873	1.94119E-16	3.07755E-15
SEMA4A	-5.383870703	0.000110072	0.000457712
SEMA7A	-8.079844176	2.67263E-37	1.52192E-35
SEMA6D	-8.588210297	0.004582016	0.013896883

GO:0006261~DNA-dependent DNA replication			
**Gene**	**Fold change**	**P-value**	**FDR**
POLD4	3.145970019	2.3798E-13	2.8675E-12
RFC3	-2.574163586	7.83055E-14	9.83577E-13
ORC6	-2.581500487	1.11439E-18	2.03804E-17
RFC5	-2.586743773	2.79508E-53	2.7161E-51
GINS4	-2.687706407	1.33709E-20	2.86361E-19
POLD2	-2.847590281	4.46441E-30	1.79422E-28
POLE3	-3.080425463	3.66867E-10	3.15251E-09
POLE2	-3.171903076	1.06381E-26	3.55794E-25
POLD1	-3.225442207	8.51608E-33	3.96806E-31

GO:0070374~positive regulation of ERK1 and ERK2 cascade			
**Gene**	**Fold change**	**P-value**	**FDR**
CD36	63.36757761	5.97993E-26	1.87179E-24
PDGFD	16.77251409	0.002076458	0.006777638
GAS6	5.804290955	2.54719E-16	3.99933E-15
ANGPT1	4.557105734	8.32302E-25	2.40126E-23
MTURN	4.352109159	9.41756E-18	1.63215E-16
TRPV4	4.058056014	0.001102152	0.003801382
CXCL17	3.935752884	1.47593E-06	8.05696E-06
CXCR4	3.508540219	0.006612558	0.019285724
PTPN22	3.038188336	0.003065265	0.009670612
PDGFRB	2.982869205	4.8726E-30	1.95024E-28
NOD1	2.549869571	1.12392E-31	4.88918E-30
BMP4	2.544828188	3.59282E-21	8.14095E-20
NELFE	-2.501540163	1.02128E-22	2.54109E-21
F2RL1	-2.563783346	1.23162E-20	2.65226E-19
CD44	-2.797376349	5.91219E-26	1.85356E-24
GPER1	-3.275958632	4.54961E-05	0.000201002
FGF2	-3.959613205	5.7836E-09	4.34649E-08
AKAP12	-4.062154915	7.1603E-41	4.52605E-39
CSF1R	-4.594053425	5.50108E-06	2.78866E-05
CCL20	-4.920922792	0.00047568	0.001762327
CX3CL1	-5.392660965	4.28155E-07	2.52096E-06
NGF	-6.297470936	3.94758E-31	1.67618E-29
GPBAR1	-7.076420696	0.010922405	0.029999319
CCL22	-7.202685389	0.001731857	0.005750954
CCL9	-7.832509209	0.006198726	0.018244955
SEMA7A	-8.079844176	2.67263E-37	1.52192E-35
CCL4	-9.343700352	0.002702413	0.008621944
PLA2G2A	-9.796287149	8.68165E-30	3.3982E-28
CHI3L1	-9.914624681	0.009960386	0.027630487
CCL7	-17.38644385	2.73857E-08	1.88212E-07
CCL2	-18.40749723	1.753E-28	6.39993E-27
CCL17	-20.22632162	0.01551371	0.040992126

IL1A	-24.85776899	2.93621E-15	4.16486E-14
